# Implications for a Wireless, External Device System to Study Electrocorticography

**DOI:** 10.3390/s17040761

**Published:** 2017-04-04

**Authors:** David Rotermund, Jonas Pistor, Janpeter Hoeffmann, Tim Schellenberg, Dmitriy Boll, Elena Tolstosheeva, Dieter Gauck, Heiko Stemmann, Dagmar Peters-Drolshagen, Andreas Kurt Kreiter, Martin Schneider, Steffen Paul, Walter Lang, Klaus Richard Pawelzik

**Affiliations:** 1 Institute for Theoretical Physics, University of Bremen, 28359 Bremen, Germany; davrot@neuro.uni-bremen.de (D.R.); pawelzik@neuro.uni-bremen.de (K.R.P.); 2 Institute of Electrodynamics and Microelectronics, University of Bremen, 28359 Bremen, Germany; jpistor@uni-bremen.de (J.P.); j.hoeffmann@gmx.net (J.H.); peters@me.uni-bremen.de (D.P.-D.); steffen.paul@me.uni-bremen.de (S.P.); 3 RF and Microwave Engineering Laboratory, University of Bremen, 28359 Bremen, Germany; trschell@gmail.com (T.S.); martin.schneider@hf.uni-bremen.de (M.S.); 4 Institute for Microsensors, -Actuators and -Systems, University of Bremen, 28359 Bremen, Germany; dboll@imsas.uni-bremen.de (D.B.); etolstosheeva@imsas.uni-bremen.de (E.T.); wlang@imsas.uni-bremen.de (W.L.); 5 Institute for Brain Research, University of Bremen, 28359 Bremen, Germany; dieter@brain.uni-bremen.de (D.G.); stemmann@brain.uni-bremen.de (H.S.); kreiter@brain.uni-bremen.de (A.K.K.)

**Keywords:** neuro-implant, ECoG, wireless implant, open hardware, neuro-prosthetic

## Abstract

Implantable neuronal interfaces to the brain are an important keystone for future medical applications. However, entering this field of research is difficult since such an implant requires components from many different areas of technology. Since the complete avoidance of wires is important due to the risk of infections and other long-term problems, means for wirelessly transmitting data and energy are a necessity which adds to the requirements. In recent literature, many high-tech components for such implants are presented with remarkable properties. However, these components are typically not freely available for such a system. Every group needs to re-develop their own solution. This raises the question if it is possible to create a reusable design for an implant and its external base-station, such that it allows other groups to use it as a starting point. In this article, we try to answer this question by presenting a design based exclusively on commercial off-the-shelf components and studying the properties of the resulting system. Following this idea, we present a fully wireless neuronal implant for simultaneously measuring electrocorticography signals at 128 locations from the surface of the brain. All design files are available as open source.

## 1. Introduction

There is nothing more drastic in a person’s life than losing control over the own body (e.g., through a neuro-degenerative disease, stroke or paraplegia). One remedy could be to use invasive brain-computer interfaces (BCIs), which allow for controlling robot-arms with cortical activity patterns (e.g., [1,2,3,4,5,6,7,8,9,10]). Effective control of external devices with invasive BCIs requires recording of neuronal data with high temporal and spatial resolution, which would be best achieved with intracortical recordings. However, this collides with the requirement of long-term stability (up to several decades). An applicable compromise are electrocorticography (ECoG) signals, recorded from the surface of the brain or the dura mater, which still contain detailed information usable for BCIs [11]. Further requirements for an implantable interface are bio-compatibility and humidity persistence.

For recording ECoG signals, a neuro-implant on top of the brain is required, preferably inside the human skull (e.g., for keeping the fluidic environment around the brain intact). Due to a plethora of reasons (e.g., preventing tissue damage [12,13,14] and infection [15,16,17]), wireless connections for energy and data are necessary.

There are many publications concerning wireless data exchange and implants (e.g., [18,19,20,21,22,23,24,25,26,27]). However, it is nearly impossible for other groups to obtain and (re-)use these systems. We would like to provide other researchers with a reusable wireless energy and data interface. Therefore, we present a neuro-implant for sub-cranial implantation that is based on commercial off-the-shelf components and we make all design files (circuit diagrams, board designs, test boards, firmware and software) available as open source. Furthermore, we present (besides an Application-specific integrated circuit (ASIC)) a firmware for a Microsemi IGLOO nano (Aliso Viejo, CA, USA) Field Programmable Gate Array (FPGA) with support for the Intan Technologies RHA2116 (Los Angeles, CA, USA) and the newer Intan RHD2132. This allows us to present a design that can be built completely from commercial off-the-shelf components and make it available as open-source. Since these integrated circuits (ICs) are all available as bare dies, the size of the system is suitable for an implant usable for human medical applications.

We tested our implant-design on a flexible printed circuit board(PCB)-foil. We analyze the results and report which problems arose. Furthermore, we examined the temperature distribution around the implant.

## 2. Results

### 2.1. System Concept

**Figure 1:**
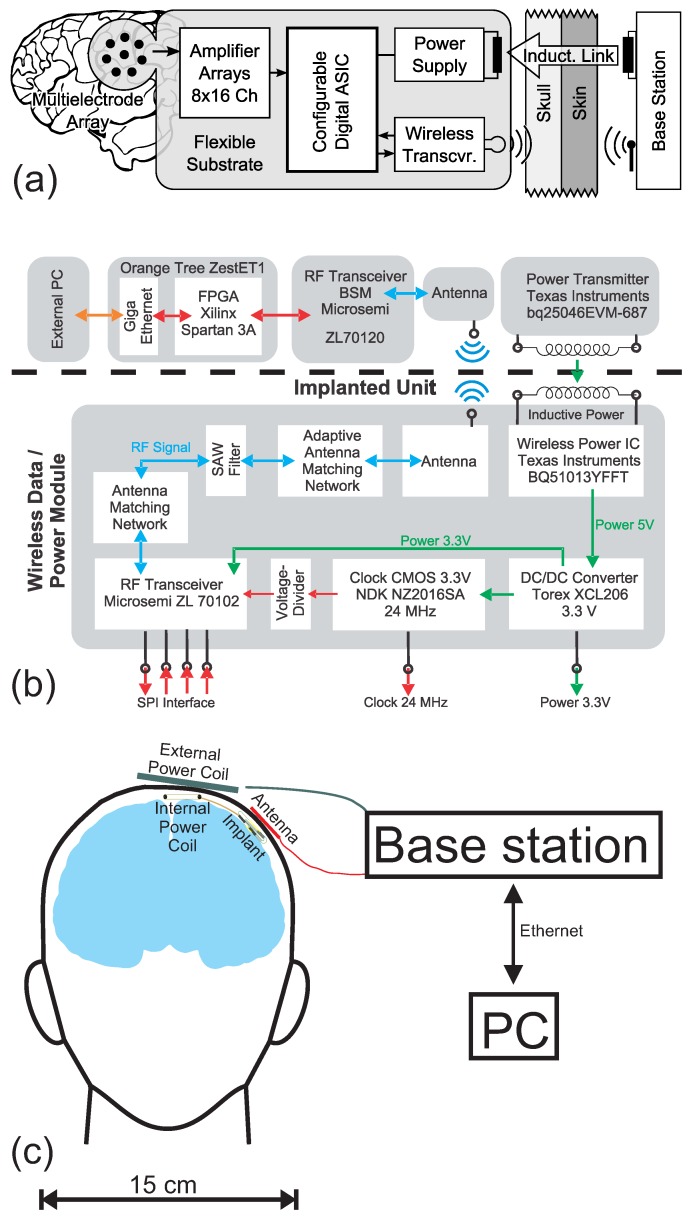
(**a**) concept of the implant with its base station; (**b**) overview of the components required for realizing the presented system concept of the wireless energy and data link; (**c**) proportions and positions of the implant, its power coil as well as the external power coil and antenna.

Our design goal was to build a system that can be implanted completely subcranially, which is supplied with energy via a wireless link (without any implanted batteries) and which exchanges data wirelessly with an external base station. Figure 1 shows the functional blocks necessary for such a system.

An array of electrodes serves as an interface between the brain tissue and a set of integrated analog signal amplifiers. analog digital converters (ADCs) digitize the amplified and band-pass filtered incoming signals. An ASIC (or FPGA) optimizes and condenses these data streams for a minimal bandwidth and transmits them via an RF transceiver data-link to an external base station. The base station processes that data and sends it via Ethernet to an external PC. User defined parameters for the data processing can be controlled via a bi-directional wireless data-link. Furthermore, the implant collects energy from an inductive wireless power link.

### 2.2. The Wireless Module

**Figure 2:**
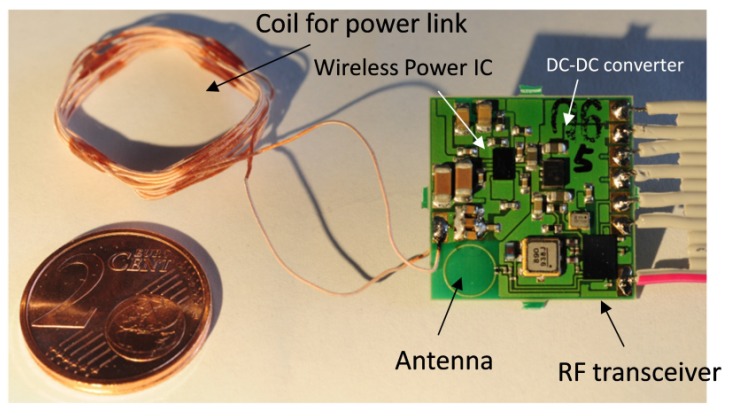
Realization of the wireless module on a 0.15 mm FR4 board with its hand wound coil for the inductive power link.

The presented wireless module incorporates two connected sub-segments: one which supplies the implant wirelessly with energy and the other one for wireless communication. Figure 1 visualizes all the necessary components plus its external counterparts. Figure 2 shows a PCB realization of that block diagram with a total size of 20mm×20mm×1.6mm.

**The power supply:** The wireless power link is based on the Texas Instruments bqTESLA system (Dallas, TX, USA) [28]. This Qi power link can deliver up to five watts. On the primary side (base station), we used the Texas Instruments bqTESLA wireless power evaluation kit (bq25046EVM-687) (Dallas, TX, USA) [29]. On the secondary side, a BQ51013YFFT as power receiver [30] was used. The power receiver’s output is too high for operating the other components. We applied a Torex XCL206 step-down micro DC/DC converter (San Jose, CA, USA) with built-in inductor [31].

**Data Transfer:** The wireless data transfer is based on Microsemi ZL70102 transceivers (Aliso Viejo, CA, USA) [32]. The RF transceiver operates in the Medical Implant Communication Service (MICS) frequency band (401-406 MHz) and is commercially available for medical applications including implants. The ZL70102 requires a 24 MHz clock. We used a very small (2mm×1.6mm×0.7mm) clock from Nihon Dempa Kogyo (NDK) (NZ2016SA) (Tokyo, Japan) [33]. It also provides a clock signal for data processing. Between the ZL70102 and the antenna (circular loop antenna with 5 mm diameter), we installed an adaptive antenna-matching circuit based on a SAW (surface acoustic wave) filter (RF Monolithics RF3607D, 403.5 MHz SAW-filter, (Dallas, TX, USA)) [34] and the two tunable ZL70102 capacitors (automatically optimized by the transceiver). The base-station is based on a Microsemi ZL70120 [35]. The FPGA is part of the Orange-Tree-Technologies ZestET1 (Oxfordshire, UK) with Gigabit Ethernet connectivity [36], which allows for streaming the data to an external PC.

### 2.3. The Implant Prototype

**Figure 3:**
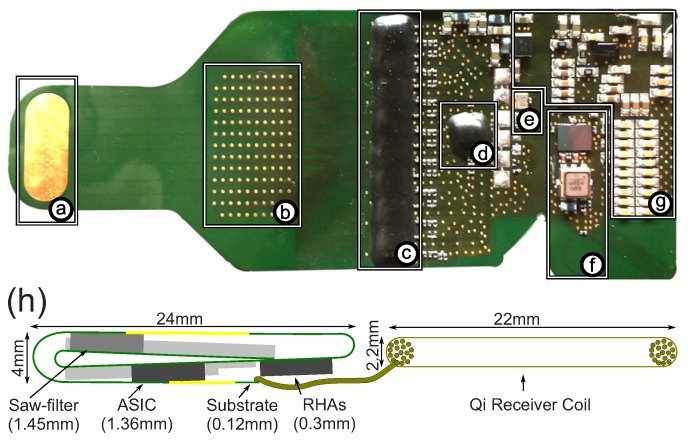
(top) implant prototype: (**a**) reference electrode; (**b**) 128 electrodes; (**c**) 8x Intan Technologies RHA2116; (**d**) ASIC (Application-specific integrated circuit); (**e**) 24 MHz clock; (**f**) radio frequency (RF)-transceiver; (**g**) inductive energy link. Implant has a weight of 1.72 g and is 32 mm wide; (**h**) drawing of the folded implant. The coil has a square shape.

The described system for the implant is realized (see Figure 3) with 128 gold electrodes on a flexible 50 μm thick PCB-foil (DuPont Pyralux AP, (Conterm, Luxembourg)).

**Analog front-end:** For the analog front-end, 8 Intan RHA2116 chips are used, which include the neural amplifiers and an (undocumented) ADC. The ADC allows for sampling all its individual channels at 10 kHz and 16 bit resolution.

**ASIC/nano FPGA:** The individual ADC data streams are collected by an ASIC [37]. The ASIC also significantly reduces the incoming data according to user-defined parameters in order to utilize the limited RF data bandwidth in an optimal way. The ASIC also controls the RF transceiver as well as provides and caches the outgoing data for achieving a high and continuous data transmission rate. We re-implemented the ASIC on a Microsemi IGLOO AGLN250 FPGA. Besides implementing the firmware for the Intan RHA analog-front end, we also wrote a second version for the newer Intan RHD2132. Table 1 shows the required resources on the FPGA.

### 2.4. Performance of the Wireless Module

Test boards with the wireless module were produced on 150 μm thick FR4 and 50 μm thick flexible PCB-foil substrates. Both versions were tested successfully. However, due to the required very fine resolution (50 μm strip width and distance between elements) of the PCBs, most of the flexible PCB-foils were produced with faults (e.g., shortcuts). Fortunately, we were able to fix some of them by manual cutting and grinding. As a result, the implant prototype used for testing was equipped with one RHA.

**Figure 4:**
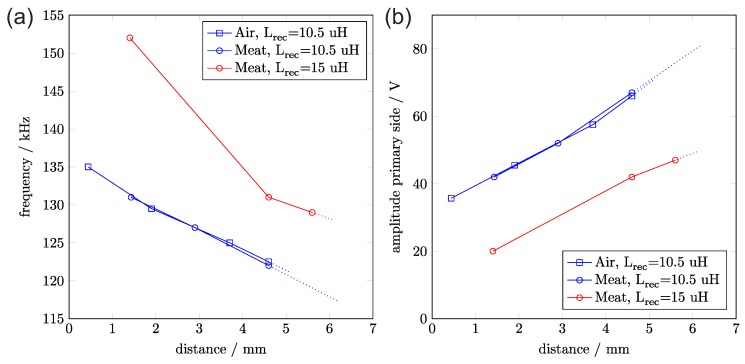
(**a**) wireless operation distances and according frequencies and (**b**) primary voltages (shown for two different coils and 100 mW DC transferred power).

**Power link:** For the secondary side, we used a handwound coil (see Figure 2). Our transmitter can bridge a distance of of 4.5 mm with the described coil (L = 10.5 μH, Q = 1). With a modified receiver coil, we reached 5.5 mm (L = 15 μH, Q = 0.76). Figure 4 shows results of a range measurement and how the transmitter’s parameters adapt accordingly.

An update of the Qi standard [38] was announced, which will work over distances between 12 mm and 45 mm while being backwards compatible with the existing receivers. Furthermore, the ’Rezence’ standard from the alliance for wireless power was also announced to have similar properties. These new standards are based on magnetic resonance, which will permit thick obstacles between the primary and secondary coil. It is expected that an update of our implant to these new standards will allow for placing the secondary coil also under the skull.

**Figure 5:**
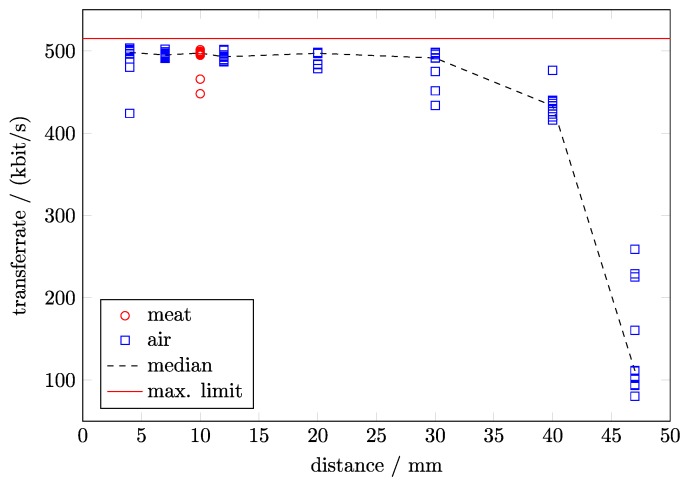
Data transfer rates for different distances.

**Data link:** We measured data transfer of the wireless data link (Figure 5) and found that the data can be transmitted with almost maximum transceiver speed through 10 mm thick stacks of sliced meat. We also tested the implant in air and observed comparable transmission rates at similar distances. A data transmission was possible up to 47 mm, but with a strongly reduced data rate due to re-transmissions of corrupted packets. Even under good conditions, some samples are lost because the transceiver is not optimized for real-time transfer but for good data integrity. The reason for the data loss lies in the limited amount of ZL70102’s buffer. Even small transmission pauses will fill the ZL70102 buffer quickly. Then, data needs to be discarded if it cannot be buffered elsewhere. Thus, time-stamps were included in the data packages to reconstruct if packets are lost.

### 2.5. Performance of the Analog Front End

**Figure 6:**
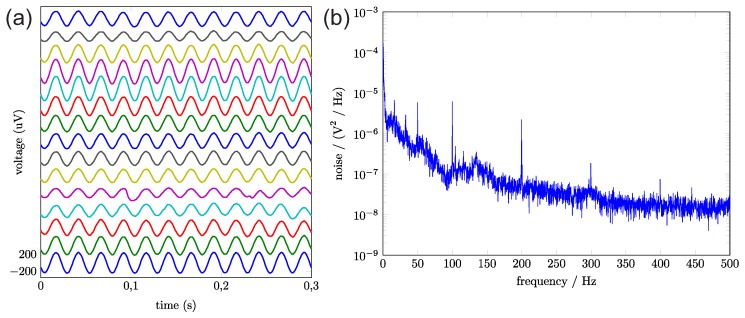
(**a**) received signals for a 40 Hz test signal. The amplitude depends on the distance between the stimulating wire and the channel electrode. The different channels are depicted in offset steps of 400 μV; (**b**) noise spectrum for open inputs in the Ringer solution.

After placing the implant’s electrodes in Ringer solution, we analyzed the signals for an root mean square (rms)-noise test. The prototype was sampling 16 channels with 1 kHz and a resolution of 10 bits. The rms noise of the measurement is 7.9 μV. Figure 6 shows the noise spectrum and what sinusoidal waves look like when recorded by the implant.

In preparation for another test, we soldered cables onto the individual electrodes of the implant. We found that when the reference electrode of the wireless system was not grounded, the amplitude of the recorded signal was strongly reduced and the neuronal signal nearly vanishes from the recorded time series. One hypothesis as to why this problem occurs is that this configuration allows the power supply to induce a strong ≈100 kHz sinusoidal signal on top of the neuronal signal at the inputs of the amplifier. Measurements with an oscilloscope revealed such voltages. These superimposed signals are now larger than the threshold voltages of the RHA’s ESD (Electrostatic discharge) protection diodes. As result, the diodes open a direct connection to ground, which eradicates the signal.

A larger distance between the RHAs and the power coils may reduce the problem or switching to a different kind of wireless energy link system might also remove this problem. This problem could also be an artifact of the several 10 cm long test-cables soldered onto the electrodes, which may act as an antenna. Unfortunately, the project ended before we could pinpoint the reason for the problem.

### 2.6. Examining the Implant’s Thermal Properties

**Estimated power consumption of the implant:** An estimate for the main electrical loads of the components of the implant is shown in Table 2. Including the losses of the power supply ICs, the fully equipped implant will dissipate about 110 mW–140 mW, while the intensively tested prototype with one RHA consumes about 73 mW–103 mW. For safety reasons, the power receiver is limited to max 200 mW.

**Tissue Heating:** A major concern for neural implants is the heating of the tissue, as proteins start denaturation at approximately $40\;{}^{\circ }$C. The IEEE Standard [39] states that a brain temperature of $40.5\;{}^{\circ }$C is critical for a heat stroke. Due to the folded structure of our implant, all active components are embedded inside the implant, which strongly increases the contact area to the tissue.

Another heat source are eddy currents from the inductive field of the power and data transmission in the conductive tissue and in the implant. The eddy currents are expected to be negligibly small. The whole RF transceiver only consumes 17 mW, and only a percentage of it is transformed into field energy.

Finally, the joule heating of the base station coil on top of the head’s skin increases the temperature of the tissue, but this can be counter-measured by an external cooling system.

**Figure 7:**
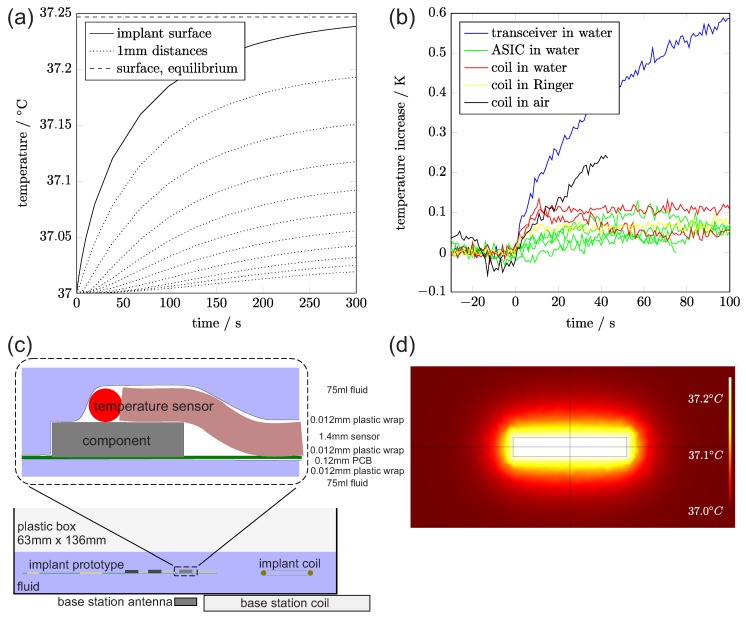
(**a**) simulated heat-up; (**b**) measured temperature increase after power on; (**c**) measurement setup for testing the heating up of the implant; (**d**) temperature distribution after 300 s, calculated with a simple FEM (Finite element method) model (COMSOL (Berlin, Germany)). Rectangle shows the 24mm×4mm implant cross section dimensions.

**Simulation of joule heating:** We used a simple FEM model to evaluate the heating of the fully assembled and folded implant and modeled it as a heat source with 100 mW (distributed over the volume of the implant). As a result from our simulations, Figure 7d shows the expected heat-up curves at the surface of the implant and at different distances within living tissue. The temperature at the surface in thermal equilibrium is calculated to be 0.25 K above the starting temperature of $37\;{}^{\circ }C$, in a sphere with 10 cm diameter and a border temperature of $37\;{}^{\circ }C$. Additionally, Figure 7 shows a temperature map taken after 300 seconds.

**Measurement of total heating:** We also made an experiment to observe the heating. The measurement setup is shown in Figure 7c. The implant prototype was isolated with a thin PCB-foil of plastic wrap against a liquid medium (Ringer solution) with a volume of 150 mL. For the temperature measurement, we contacted a thermocouple to different parts of the implant. We expected the surface temperature to be saturated after a few minutes. Longer tests are not expected to provide meaningful results due to the small volume and lack cooling by blood perfusion.

The black curve in Figure 7b shows a rapid joule heating of the coil in air, while the heating saturates at approximately $0.1\;{}^{\circ }C$ in water (red) and at even lower values in the conductive Ringer solution (yellow). Close to the ASIC, which is covered under a 0.75 mm plastic housing and has a power dissipation of 9.44 mW, we also measured a temperature increase below $0.1\;{}^{\circ }C$.

In contrast to the low heating at the ASIC, the blue curve shows the temperature on top of the unencapsulated RF transceiver, which has a power dissipation of 17 mW. The transceiver is located behind the saw filter, which is higher than the transceiver. After folding, the transceiver has no direct contact with the tissue. In our experiment, the PCB-foil was not folded and we saw a strong temperature increase at the contact between the transceiver and the fluid. The ground planes are expected to distribute the thermal power more equally to the outer implant surface.

In summary, we find that the temperature rise caused by the implant in the brain is far lower than $1\;{}^{\circ }C$. This way we can exclude the danger of thermal damage to the brain. This has been investigated by modelling and experiment.

## 3. Discussion

This article started with the question of if it is possible to design a fully wireless neuro-implant and its external base-station such that the results can be reused by other groups as starting points for their own technology development activities. As an answer, we present a system concept which can be transferred into real hardware by using only commercial off-the-shelf components. All design files (circuit diagrams, boards, firmware, and software as well as documentation concerning the development process) are made open source in the supplemental materials. We deliver two versions of firmware for the Microsemi IGLOO nano FPGA. One was written for the Intan RHA2116, using undocumented ADC functionality, like the ASIC which we used for the measurements with the flexible implant prototype and another firmware version for the newer Intan RHD2132. In the long run, we mainly aim at systems for medical applications where the implant is fully placed under the skull. We also expect the system to be usable as research equipment for mammalians with suitable large heads, where possibly only parts of the implant (e.g., the electrodes) can be positioned under the skull.

In contrast to many other systems, we developed an implant that can be placed completely under the skull, avoids energy storage elements with a limited lifetime (e.g., batteries) and has 128 channels for measuring ECoG signals. The fact that our wireless system can be completely implanted is crucial for long-term stability, keeps the natural barrier against germs intact and prevents cerebral fluid leakages [15]. The thickness of the prototype is, with its ≈4 mm, on the high side for implanting it between skull and brain. This could, depending on the individual width of the cerebrospinal fluid layers, create harmful pressure on the brain tissue. There are several ways to prevent this problem: replace the pre-assembled SAW-filter (1.45 mm thick) by thinner individual components, make the bone on the inside of the skull thinner to make head-space, or only fold the implant twice. The last option trades in a reduction of thickness for more occupied area.

In literature, many components, system parts, and system designs are presented here with remarkable performances (e.g., [18,19,20,21,22,23,24,25,26,27]), but they are not freely available. The implant we present has lower specs compared to these highly optimized solutions, but our system can be re-built by everybody and then be modified to one’s heart’s content.

One remaining challenge is finding long-term stable bio-compatible coatings which can protect the electronics from the harsh fluidic environment in the body as well as the body from toxic materials used in the implant. This very thin and flexible coating has to stay intact over many years. We designed the implant to be completely coated in a first processing step (including the power coil which is made from cooper litz wires (Menting Mikroelektrik (Gummersback, Germany)) coated with a polyurethan based insulation). In a second step, the coating must be removed from the electrodes and then the implant is folded at three folding lines for reducing the required area. Therefore, it is required that the coating is not only flexible but has a good adhesion to all the components. We expect that the adhesion between the coating (e.g., Parylene C) and the used material for the PCB-foil (DuPont Pyralux AP and insulating resist) might cause problems. This requires changing the substrate of the PCB-foil to something more suitable (e.g., Parylene C as well) and will hopefully give us the opportunity to reduce the thickness of the substrate for improving the bending radius of the PCB-foil. Currently, we are testing several promising candidates for coatings and substrates [40].

Two aspects of the implant need improvement: (1) By exchanging the power harvesting to magnetic resonance technology (the new Qi standard or the Rezence wireless power charging standard), the maximal operating distance between the primary and secondary coil can be increased up to 40 mm. In the actual state, our implant requires an energy harvesting coil between the skin and outside of the skull which is connected with two small wires to the implant under the skull. (2) The effective data transmission rate is limited to 515 kbit/s. For many applications, this transmission rate is too low. We looked into the possibility of optical data transmission via infra-red light. Together with the BIAS (Bremen Institute of Applied Beam Technology), we tested the feasibility of this idea by sending high-frequency signals through muscle, skin and bones. We expect that data transfer rates of over 100 MBit/s should be possible with optimized micro-optics and a vertical-cavity surface-emitting laser (VCSEL) on the implant as well as a suitable external receiver. If code division multiple access (CDMA) is used, even several implants can send information on the same wavelength. For the channel from the external base-station to the implant, the slow RF connection still can be used or also replaced by an IR data transmission (which is more challenging) on a different wavelength.

Many tests have proven the feasibility of our system design. However, in some special test setups, we ran into problems (for details, see results). As a result, the amplitude of the measured neuronal signals nearly vanishes when the reference of the Intan RHA2116 and the base-station do not have a low impedance connection. Such a cable is not compatible with the idea of a wireless system. The reason for this is still unclear and may be an artifact of the very special measurement setup (e.g., long cables soldered onto the electrodes of the flexible implant) or the close distance between the energy coil placement and the rest of the implant. After solving this problem, in vivo tests need to be performed in order to verify the system performance under real measurement conditions. This information is required to estimate the development steps that have to be taken for making the system safe enough for using it for human patients.

In summary, the actual state of the implant is not yet ready for implantation, especially not for long-term implantation in medical applications. Several problems still have to be solved in future development. Nevertheless, we deliver an open source tool kit completely based on commercial off-the-shelf components. This collection contains all design files, which allows interested researchers to develop their own wireless neuro-implant without starting from scratch.

## Figures and Tables

**Table 1:** Usage of the IGLOO nano FPGA (Field Programmable Gate Array) resources for an implant with Intan Technologies RHA or RHD analog front-end. A large portion (up to 33% in the case with Intan RHAs) of these core resources are by optional virtual RHAs/RHDs for testing purposes.

Resource	Usage (RHA)	Usage (RHD)
CORE	5859 of 6144 (95%)	5236 of 6144 (85%)
IO (W/clocks)	38 of 68 (56%)	38 of 68 (56%)
GLOBAL (Chip + Quadrant)	6 of 18 (33%)	6 of 18 (33%)
PLL	0 of 1 (0%)	0 of 1 (0%)
RAM/FIFO	8 of 8 (100%)	8 of 8 (100%)

**Table 2:** Estimated power consumption of the implant’s components.

Component	Power Consumption
Microsemi ZL70102 transceiver	17 mW (measured)
ASIC (Application-specific integrated circuit)	up to 9.44 mW (measured)
Clock quartz	16.5 mW (measured)
Intan RHA amplifier arrays	5 mW (each IC) (measured)
DC (direct current)/DC-Converter	8.5 mW (for 1 RHA), 15 mW (for 8 RHAs), (data-sheet)
Texas Instruments inductive power receiver	10–40 mW (data-sheet)

## Supplementary Materials

The following are available online at http://www.mdpi.com/1424-8220/17/4/761/s1. In the supplemental data, we present the design files for the firmware, software and PCB designs as open source as well as documentation concerning the development process.
