# Supplementary material for: Implications for a Wireless, External Device System to Study Electrocorticography
Source: Sensors (Basel). 2017 Apr 4;17(4):761. doi: 10.3390/s17040761 (PMC5421721; doi:10.3390/s17040761)
Supplement: Supplementary file 1 [file sensors-17-00761-s001.zip › Supplement/PCBs/Describe_DR.pdf]

Personal notes on the RF / energy module and its basestation

David Rotermund
Institute for theoretical physics
University of Bremen

January 21, 2014

Contents

1 Hardware of the base station **1**

1.1 The heart of the base station 4

1.2 Zarlink modules 7

1.2.1 Base station module 9

1.2.2 Implant module 15

1.3 Trigger channels 18

1.3.1 Trigger-In channels 18

1.3.2 Trigger-Out channels 21

1.3.3 Alternatives 22

1.4 Digital-to-analogue converter (DAC) 25

1.5 Analogue-to-digital conversion (ADC) 28

1.5.1 Version 1 30

1.5.2 Version 2 35

1.5.3 Version 3 43

1.5.4 Version 4 55

1.5.5 Version 5 85

2 Firmware of the base station **89**

2.1 Structure of the firmware 90

2.1.1 Orange Tree - Top level module 90
2.1.2 'Packet Frontend' - Our top level module 90
2.1.3 Network commands modules - Protocol 93
2.1.4 Network commands modules - Firmware 120
2.1.5 Zarlink modules 127
2.1.6 Implant data handling modules 135
2.1.7 Electro-physiological modules 152
2.1.8 Trigger channels 165

3 Software of the base station 167

3.1 Programming the FPGA 167
3.2 Monolithic software 168
3.3 Command line tools 173
3.3.1 List of the tools 174

4 The implant 181

4.1 Energy-data-buoy 184
4.1.1 Energy 187
4.1.2 Data 191
4.2 The implant's design 195
4.2.1 Pi - filter 200

Chapter 1

Hardware of the base station

A perfect wireless brain interface implant alone is not enough. It needs something outside of the body where it can send to the collected data. This external counter part can be a base station (see figure 1.1). In a minimal version the base station needs to bi-directionally communicate with the implant and also provided the necessary power for operating the implant, both wirelessly. In the case of the presented base station, data is exchanged via a 400MHz RF link and the energy is send via a dynamic inductive link. In a later part of this chapter, the base station part of the data link will be explained in more detail (see section 1.2). It is based on Zarlink ZL70102 ICs. The implant side of the RF link will be discussed in section 4.

The energy link is based on the bqTesla solution from Texas Instruments. For the base station side of the power link there is not much to report because the company Texas Instruments delivers a complete energy transmitter on a test board for around 100Euro. This module was designed for loading MP3 players and mobile phones. It works according to the new QI-wireless standard for wirelessly loading consumer products with up to 5Watt. 5Watt is more than one order of magnitude larger than the amount of power necessary (and allowed) for operating an implant. Due to the dynamic nature of the inductive energy link, the implant only collects the required amount of energy from the field and does not need to 'burn' the un-required amount of energy. This is possible because the external TI power transmitter and the energy receiver communicate via the inductive link. According the actual power requirements of the implant, the transmitter changes its frequency (between 110kHz and 200kHz) and thus changes the efficiency of the receiver's resonator (which has a fixed resonating frequency of around 100kHz). This power solution works fine as long as its communication channel is working. The communication channel may fail if the distance between the coil of the transmitter and the coil of the receiver gets too large. For increasing the maximal distance between the transmitter and the receiver it is necessary to improve the involved coils. Due to the larger available space for the external energy transmitter, it makes sense to optimize this coil first. However, we didn't looked into this problem during the project and tested the system with the standard external coil delivered by Texas

Instruments.

Beside the minimal features a base station needs to have, our base station has some additional features. A complete overview of the base station is shown in figure 1.1.

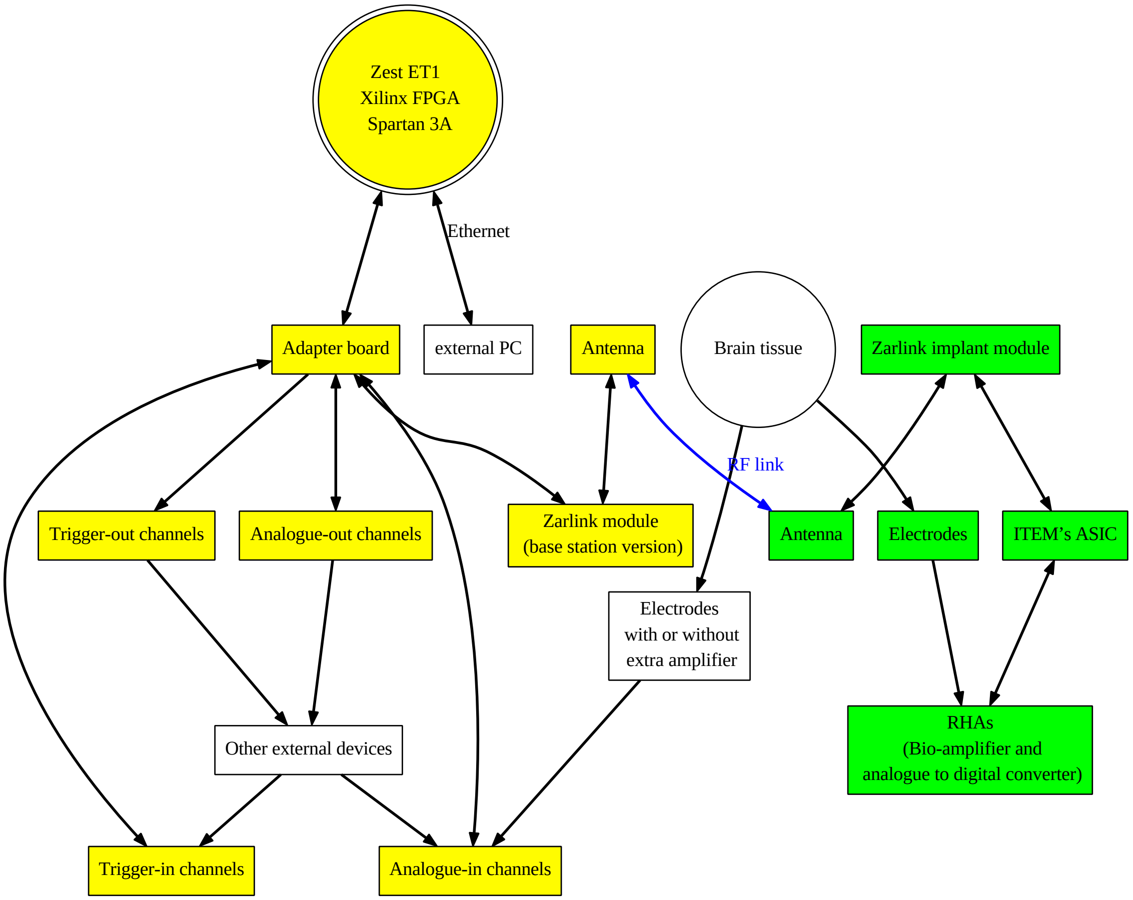

Figure 1.1: Overview of the complete system and the information flow through it. The yellow marked components are part of the base station and the implant consists out of the green parts. Power link not shown.

The core of our base station is the Orange Tree Zest ET 1 FPGA board (Xilinx Spartan 3A XC3S1400A FPGA) with an easy to use 1GBit Ethernet hardware stack (see section 1.1). All components (except the TI power transmitter) are connected to this FPGA board. It orchestrates the communication between the other components and exchanges data via Ethernet with an external PC, which allows user to control the base station and the implant as well as analyse the collected electro-physiological data. Beside the necessary software on the external PC (see chapter 3), the FPGA needs a custom firmware for talking to the connected hardware and establishing the necessary communication procedures (see chapter 2).

Talking to those who would perform the tests with the implant, showed very fast which extra features were required. One major issue was the synchronisation between

the base station and other external systems like visual stimulator computer or eye position measurement systems. The communication to these system made it necessary to provide fast binary input / output channels. In the following these binary channels are called input / output trigger channels (see section 1.3).

Another important and required feature was to have analogue-to-digital converter (ADC) channels (see the sections beginning with section 1.5) inside the base station. These channels are necessary for recording electro-physiological data from wirebound electrodes, which can be used for comparing them with the neuronal activities measured by the implant. Furthermore, these ADC channels can be used to record the analogue eyes' positions, measured by the eye tracker. Requirements for these ADC channels are that they can record signals within μV resolution but with an ignorable DC offset with some 100mV. The input impedance has to be as high as possible, the channels need a sampling rate of 25kHz, the number of channels scalable and the recording as much as possible in synchronicity with the data stream of the implant.

Last and least, the base station needs a way to produce analogue waveforms as test signals and as test-bed for low-latency closed-loop electrical stimulation of brain tissue. For providing this feature, a multi-channel digital-to-analogue converter was included as optional accessory for the base station (see section 1.4).

Most of boards that are shown in the following section were designed by myself using Eagle Version 5.11.0 (under Linux). Most of these designs were produced and assembled by PCB-Pool ($http://www.pcb-pool.com$). In the cases where this was not the case, I will note it in the text. Regarding the design of the circuitry, I followed the advice of Martin Schneider and tried to stick as close as possible to reference designs and manuals from the IC manufacturers. However, this was not possible for all cases. Especially the ADC channels required more creativity. Due to the complex nature of electro-physiological recordings, a close cooperation between Andreas Kreiter and myself for laying out the parameters of the analogue front-end (amplification factors, low-pass, high-pass, and band-pass filters) and testing it was essential. For a rough simulation of the analogue front-end, I used TINA 9 and Filter Pro from Texas Instruments. For drawing the showed circuit diagrams I used qucs which I edited with inkscape and the bubble graphs I used Graphviz.

Using the described procedure plus a huge amount of missing knowledge, it is astonishing that nearly all my designed boards worked.

1.1 The heart of the base station

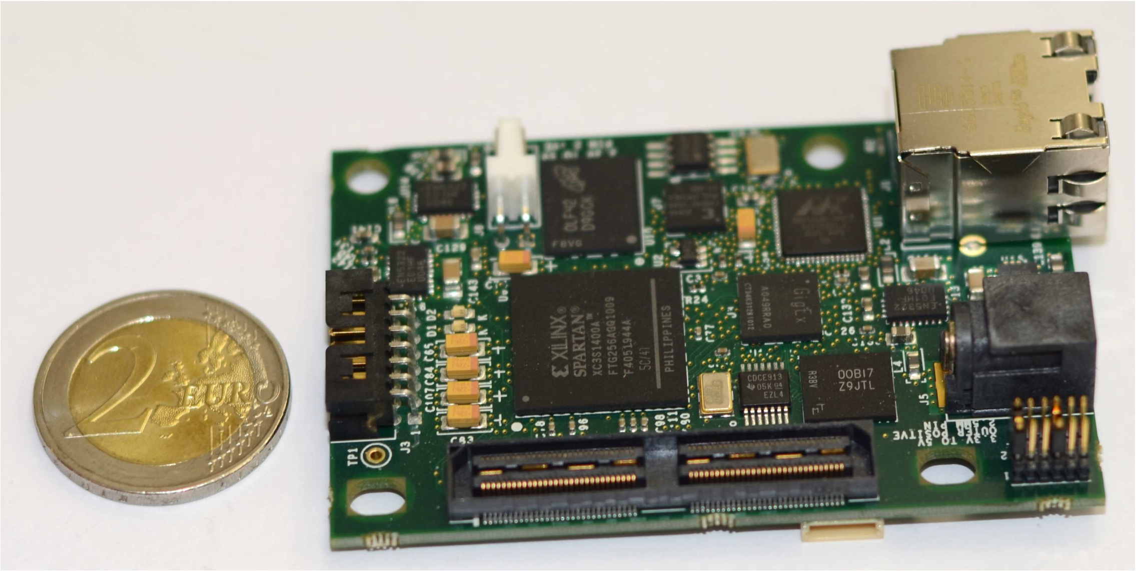

Figure 1.2: The heart of the base station is a Xilinx Spartan 3A XC3S1400A FPGA on a board from Orange Tree, called Zest ET1. Beside the FPGA, the ZestET1 contains an IC providing a Gigabit Ethernet stack which can easily accessed from the FPGA.

The base station was build around a Xilinx Spartan 3A XC3S1400A FPGA. This FPGA will run the firmware for controlling and interacting with all the other parts of the base station (for more information about the firmware see chapter 2). The FPGA is housed on a board (see figure 1.2), called ZestET1, produced by the British company Orange Tree (http : //www.orangetreetech.com/). The board was selected because it provides an easily accessible GigaBit Ethernet stack via a special IC for communicating with an external PC. Furthermore it contains 64 MByte of RAM, which we will not using in this project. For connecting our other components, the FPGA board has a high density Samtec QSH-060-01-L-D-A connector allowing to access 80 user IO pins of the FPGA.

Connecting the other parts of the base station directly to the ZestET1 via the Samtec connector plug is problematic due to its high density nature (0.5mm pitch; up to 9.5GHz signals) and it's relatively high price of around 10 Euro per connector plug. Furthermore, the corresponding connector cables (cost roughly 40 Euro) consist out of micro-coaxial wires between the two connector plugs. And, most importantly, the IO pins of the FPGA can not be distributed among several accessory boards.

My first idea was to use one Samtec connector cable and cut off one of the plugs, split the package of wires, un-isolate them and finally solder them on the individual PCBs. This procedure failed. Each individual wire consists out of a very thin copper conductor which is surrounded by a flexible isolator. The flexible isolator is wrapped

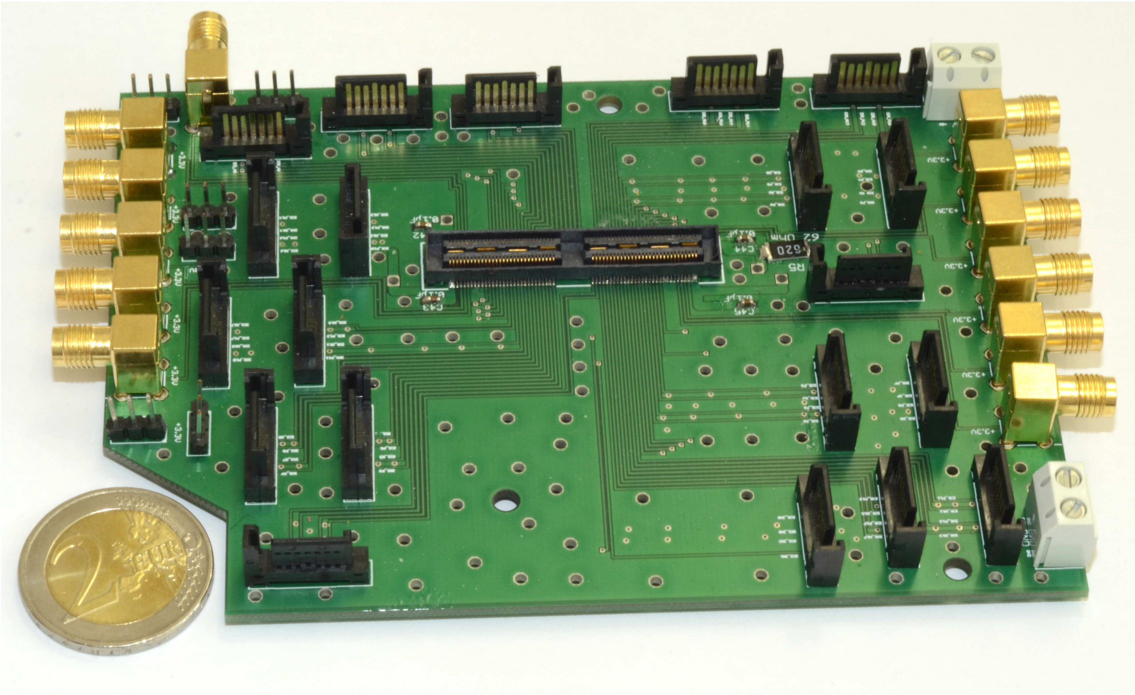

Figure 1.3: Large adapter board for converting the Samtec connector from the ZestET1 to SATA connectors. The SMA connectors deliver 3.3V from the DC/DC converter on the ZestET1 board to the external parts. The grey screw-able connectors can be used to supply the main 5V power power rail to the FPGA board.

within a metal mesh for grounding. And this is again covered with another layer of isolator. This structure is very fragile when worked on. The result was that with normal tools it takes many attempts to make the inner metal conductor accessible (and removing the ground mesh cleanly), which makes it hard to control the length of the wire. Furthermore, these type of connections break very easily. Thus this approach is not suitable for connecting many hundreds of wires to external components.

A second approach was to use a Samtec breakout cable. It converts the QSH connector plug into many individual SMA connectors. One problem is the extremely high price which is in the region of 400 Euro per adapter. Another problem is that the available versions of this adapter provide only a low number of pins from the total 80 available user IO pins of the FPGA. Furthermore, it is (for mechanical reasons due to the ZestET1 layout) necessary to use an additional adapter cable to connect the breakout cable to the Zest ET1. Theoretically it is possible to ask Samtec for a custom cable but alone the costs for the special design are beyond the 1000 Euro price tag.

As a solution, I decided to design an adapter board. The results was a board that splits the 80 user IO pins of the Zest ET1 into packages of four pins using SATA cables and plugs. A normal SATA cable contains four flexible conductors for I/O plus 3 ground lines which are all shielded with several layers of metal foil. The cables are used for

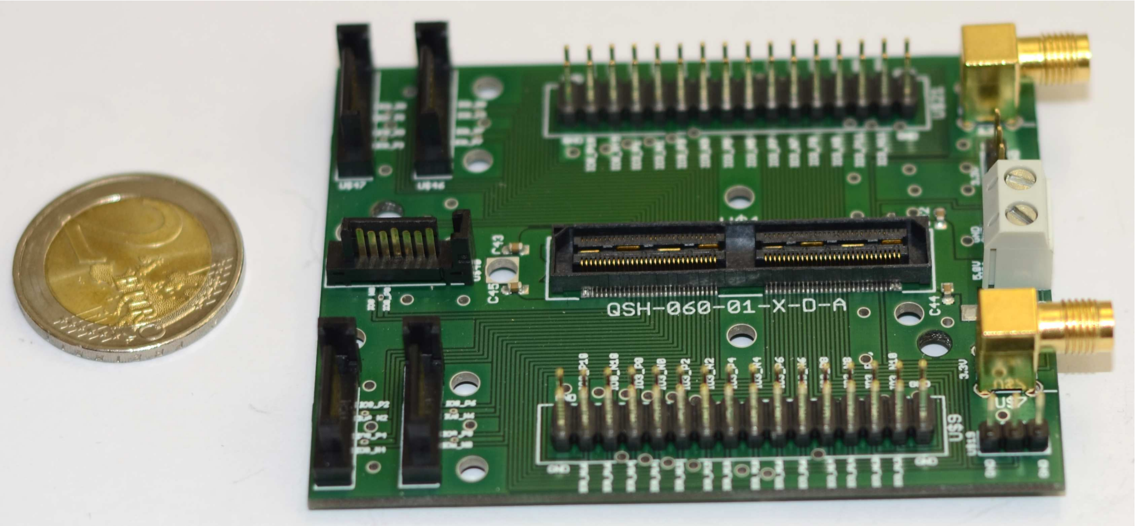

Figure 1.4: Smaller version of the adapter board shown in figure 1.3. This version has a lower number of SATA plugs and distributes the remaining user IO pins of the FPGA board via two pin headers. Furthermore, this board has a lower number of SMA connectors providing 3.3V DC supply voltage from the Zest ET1 to the other components.

differential signal transmission (Low-voltage differential signaling) with data-transfer-rate with, depending on the SATA version, much more than 1 gigabit per second. For the base station a transfer rate of 50MBit per second is sufficient. These SATA cables are available in large amounts and the plugs cost below 2 Euro each. Only the small pitch of the SATA plug is slightly demanding for the PCB manufacturer but can be realized with the typical 5mil (or better) standard production processes.

It turned out that the soldering of the Samtec connector onto the adapter boards was much more demanding than expected. The surface tension of the solder pulls the molten solder along the connector's pin into a region which is not accessible by normal tools any more. Thus too much solder can result in a short-cut between two pins that can not be removed any more. This problem occurs with reflow soldering (using a stencil) as well as during soldering with a soldering iron. I was able to assemble one board with reflow soldering in an oven and sheer luck but lost several ones during that process (especially when using a soldering iron). In the end, the IMAS (Ole Woitschach and Dmitriy Boll) soldered successfully some of these connectors to boards. After that, all other versions of these adapter boards were assembled by PCB pool, which also produced the PCBs for us.

In the end two versions were produced: The larger one (see figure 1.3) which externalizes all the user IO pins of the FPGA to SATA plugs as well as a smaller version (see figure 1.4) with only 20 user IO pins connected to SATA plugs but with accessibility to the remaining pins over two pin headers. Both boards contain SMA plugs, which

are connected to the 3.3V DC/DC converters of the ZestET1 board, converting the 5V supply voltage of the FPGA.

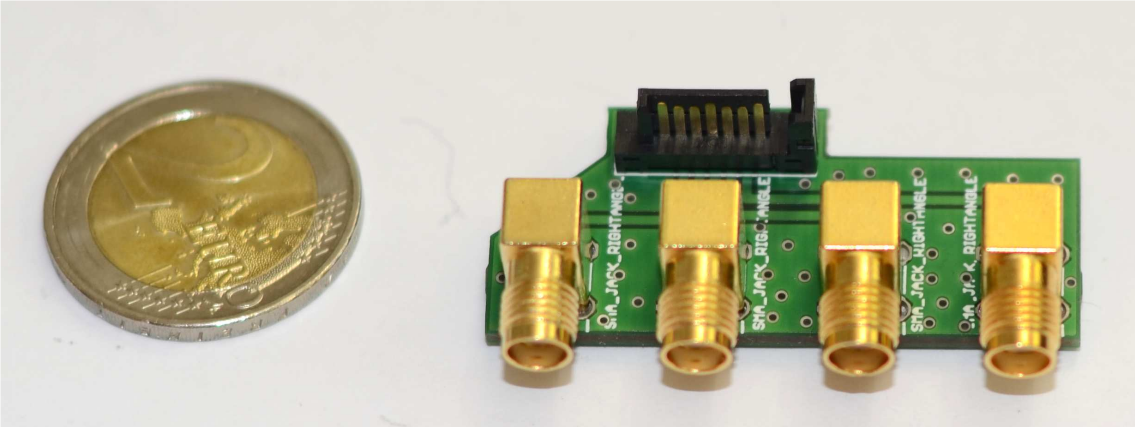

Figure 1.5: Small SATA to SMA adapter board.

One disadvantage of using SATA cables is accessing the signals for test purposes. For solving this problem, I designed and assembled small adapter boards (the PCBs were produced by PCB-Pool). The adapter boards convert each of the SATA plugs into four individual SMA connector. This adapter (see figure 1.5) was not designed for high speed signals. Thus the length of the individual lines are not optimized as well as the impedance of the lines. A similar adapter, but optimized for faster data transfers, is available from Xilinx for 395USD each.

1.2 Zarlink modules

Building a fully implantable system requires the capability of transferring data from outside into the body (e.g. control data or data for electrical stimulation) as well as the ability to transmit data from inside of the body to an external base station (e.g. measurements of the neuronal activity). One way is to use radio frequency (RF) signals. The electro-magnetic wave travels through the tissue & bones and carries the data modulated onto a carrier-wave with it.

After many discussions and searching (by Anne Vogel from Brain Products) through all the complex European standards for legally using frequency bands for wireless data and energy transmission, Tim Schellenberg (RF & Microwave Engineering Laboratory of the University Bremen) reviewed the technical specifications (especially the values for power consumption and data transfer rate) all the available RF transceiver solutions. In the end, he suggested to use a Zarlink ZL70102 IC for our project.

The ZL70102 is a medical implantable RF transceiver which works on the MICS (Medical Implant Communication Service; 402-405 MHz) band frequencies and is produced

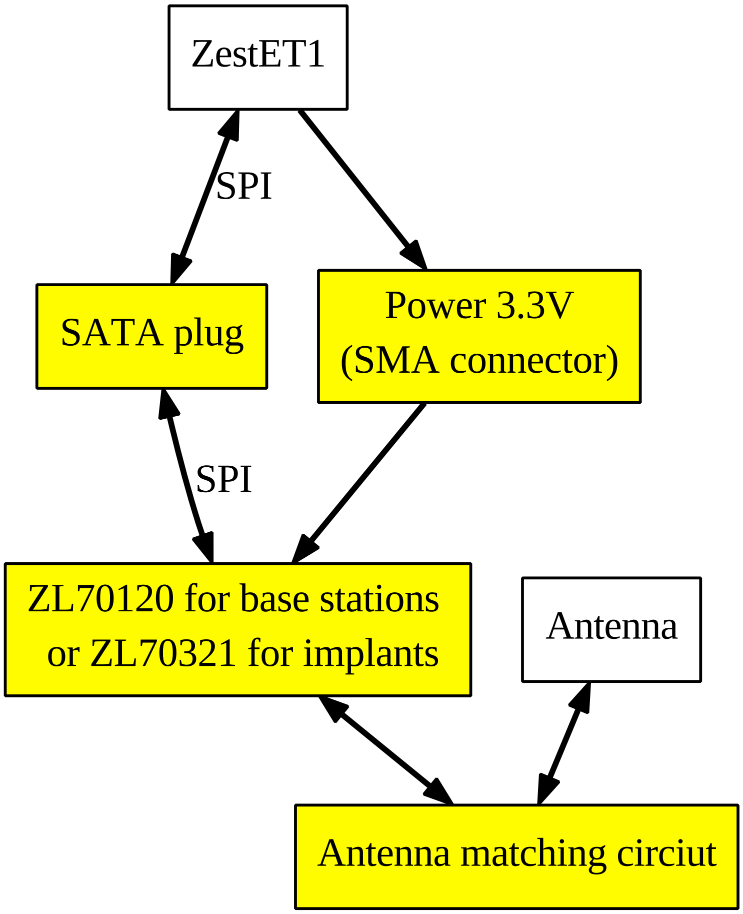

Figure 1.6: Structure of the base station module pcb or implant module pcb. The yellow components are part of this RF transceiver board.

by the company Zarlink Semiconductor (which is now part of the company Microsemi). This RF transceiver IC is a very complex piece of technology. The ZL70102 is able to use 4-FSK (frequency shift keying) or 2-FSK coding and it can – theoretically – produce a raw data transfer rate with up to 800kbit/sec. This translates, again theoretically, in up to 505 kbit/sec. The energy consumption is very low with around 15mW. Commands and data are interchanged with the IC via the SPI protocol and it has more than 100 registers for configurations. The ZL70102 has also the possibility to use an additional RF link (at 2.45GHz) for waking it up from an energy saving mode but we don't use this wakeup link in the project. This is because we aim for an application with a continuous wireless data transfers which doesn't allow pauses by definition. If a kind of energy saving mode is required than the wireless energy to the implant can simply be stopped by turning of the energy transmitter.

Using a ZL70102 for a base station or a implant requires a different set of external components. Thus, if possible, it is very helpful to use the ZL70120 MICS-band RF base station module or the ZL70321 MICS-Band RF standard implant module instead

of a pure ZL70102 (however, these modules have a price tag roughly 5 times those of a simple ZL70102). Both modules contain a, by the ZL70102 required, crystal clock (24 MHz) as well as the necessary filter-banks (which are different for the external module and the implant). They allow to realise the required designs in a much simpler way but they require extra care while soldering them. It is important to use as low as possible temperatures for the soldering process (I recommend a solder paste with lead if possible). The ZL70321 requires an assembling process with reflow soldering, because all the connections are on the flip-side of the module. This is similar to a BGA (ball grid array) with flat pads. In the case of the ZL70120, most of the connections can be soldered from the IC's frame with a simple soldering iron but it has also one large connection on its back side. This can be soldered via reflow soldering or, if additional holes / vias are placed below the module on the PCB, with the soldering iron.

For the base station and the FPGA based prototype version of the implant (designed and build by Karl-Heinz Open, Jonas Pistor, and Janpeter Hoeffmann from the ITEM) we required PCBs for these two types of Zarlink modules (see figure 1.6). In the following I will present my designs for these PCBs:

1.2.1 Base station module

Since most of the effort for the complex high frequency design was saved by using the ZL70120 (see figure 1.7) instead of a simple bare ZL70102, the main task would have been the antenna matching circuit. However, the manual of the ZL70120 states that the RF400 pin has an impedance of 50 Ω. Thus everything is already matched for a dedicated 50 Ω antenna for the 400 MHz transceiver part. In the case of a combined 400MHz and 2.45GHz (for the wakeup part) antenna, the handbook notes that it is a bit more complicated. To keep the design flexible regarding the putative use of the wake-up link, we decided to keep the 2.45GHz parts usable.

Zarlink produces a special test kit, containing a base station unit (BSM200 = Base Station Module with a ZL70102) based on the ZL70120 and an implant unit (AIM200 = Application Implant Module with a ZL70102) based on the ZL70321. We took the BSM200 reference design for the antenna matching circuit (see figure 1.8) and re-used it for our own design. For determining the parameters of the pcb cooper stripe between the RF pins of the ZL70120 and the antenna, I used the transmission line calculator TX-Line 2003 (as suggested by Tim Schellenberg; see figure 1.9).

In the first design (see figure 1.7), I had the idea to separate the RF transceiver from the FPGA, which are connected via the SPI lines. I had the apprehension that the 400 MHz signals could contaminate the rest of the base station's signals. For that reason it tested ADUM 1401 digital isolators from Analog Devices (see figure 1.10). The isolators alone for worked in these tests fine and I included them in the design.

However, when the whole board was tested, we had problems with the isolators (e.g. it

drained power from the signal pins in the case when supplied power was too low) and we removed them again (note the orange wires in figure 1.7). Another problem with the first design was that the manual of the ZL70120 was not completely clear about the purpose of one (very important) pin. Thus we had, without knowing, a non-connected supply pin for the digital processing on the ZL70102, which caused in some of the assembled boards strange behaviours (thanks to the Zarlink support team we found that error). Luckily the pin was available on a debug pin header on the board, thus the problem could be fixed. Strangely some of the boards worked fine even without the correctly connected pin, which created some extra confusion. And in addition we had some problems with the configuration of the FPGA pin. Tests showed that the Zarlink required these pins to be configured for pullup / pulldown mode. All this problems were occurring simultaneously and made debugging very hard.

In the end, I simplified the design and we produced the second (final) version of this board (see figure 1.11). This version showed no problems so far and works fine.

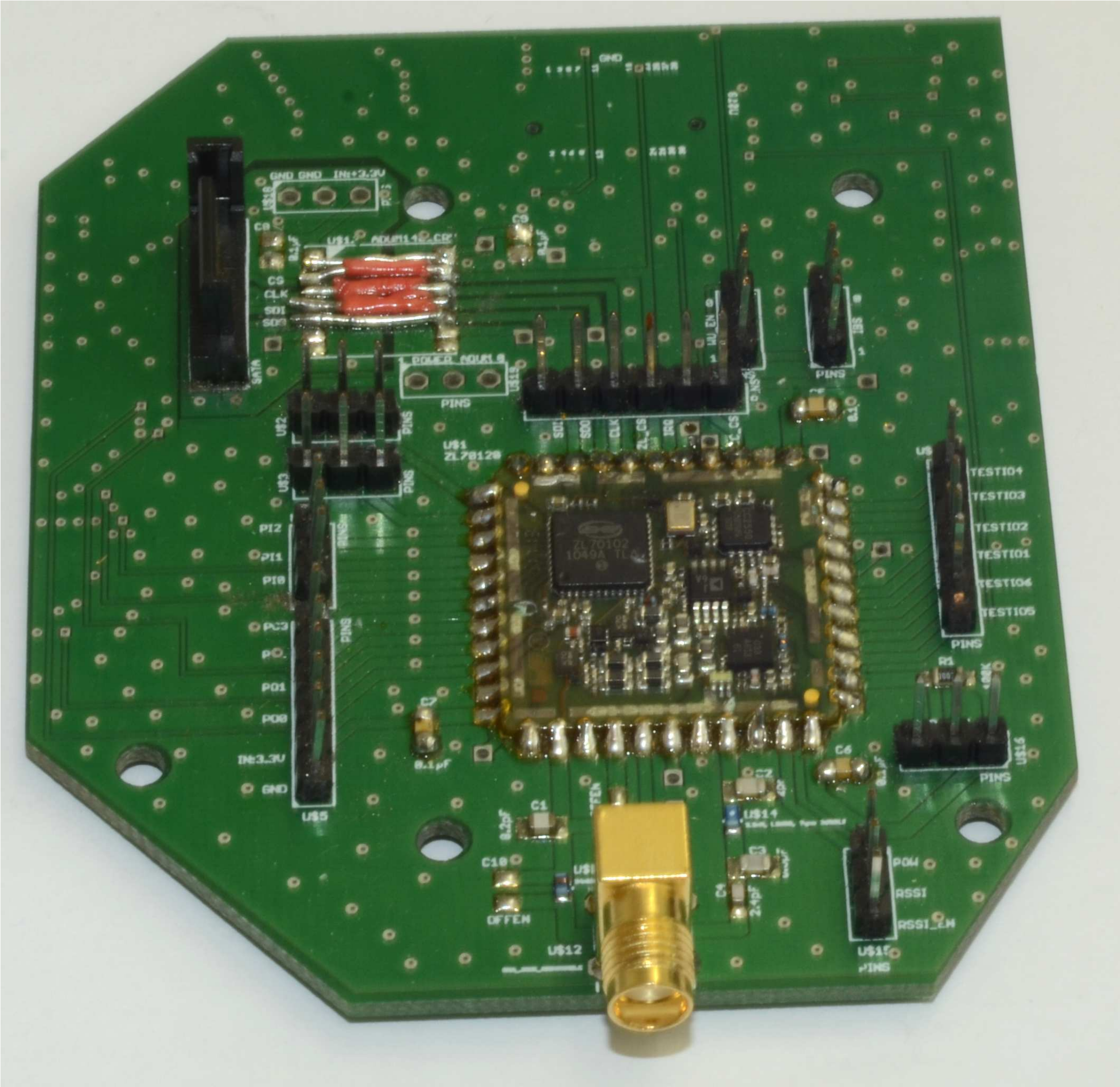

Figure 1.7: First version of the Zarlink base station board. In the middle of the PCB the Zarlink ZL70120 base station module is shown with an open shielding can.

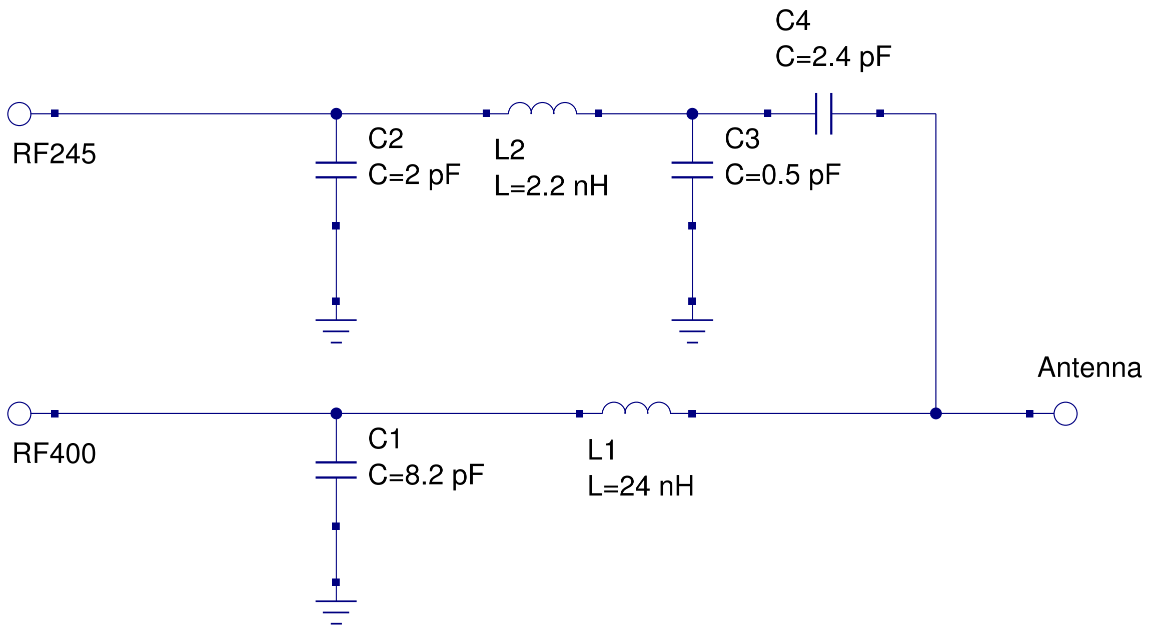

Figure 1.8: Antenna matching circuit taken from the Zarlink BSM200 reference design.

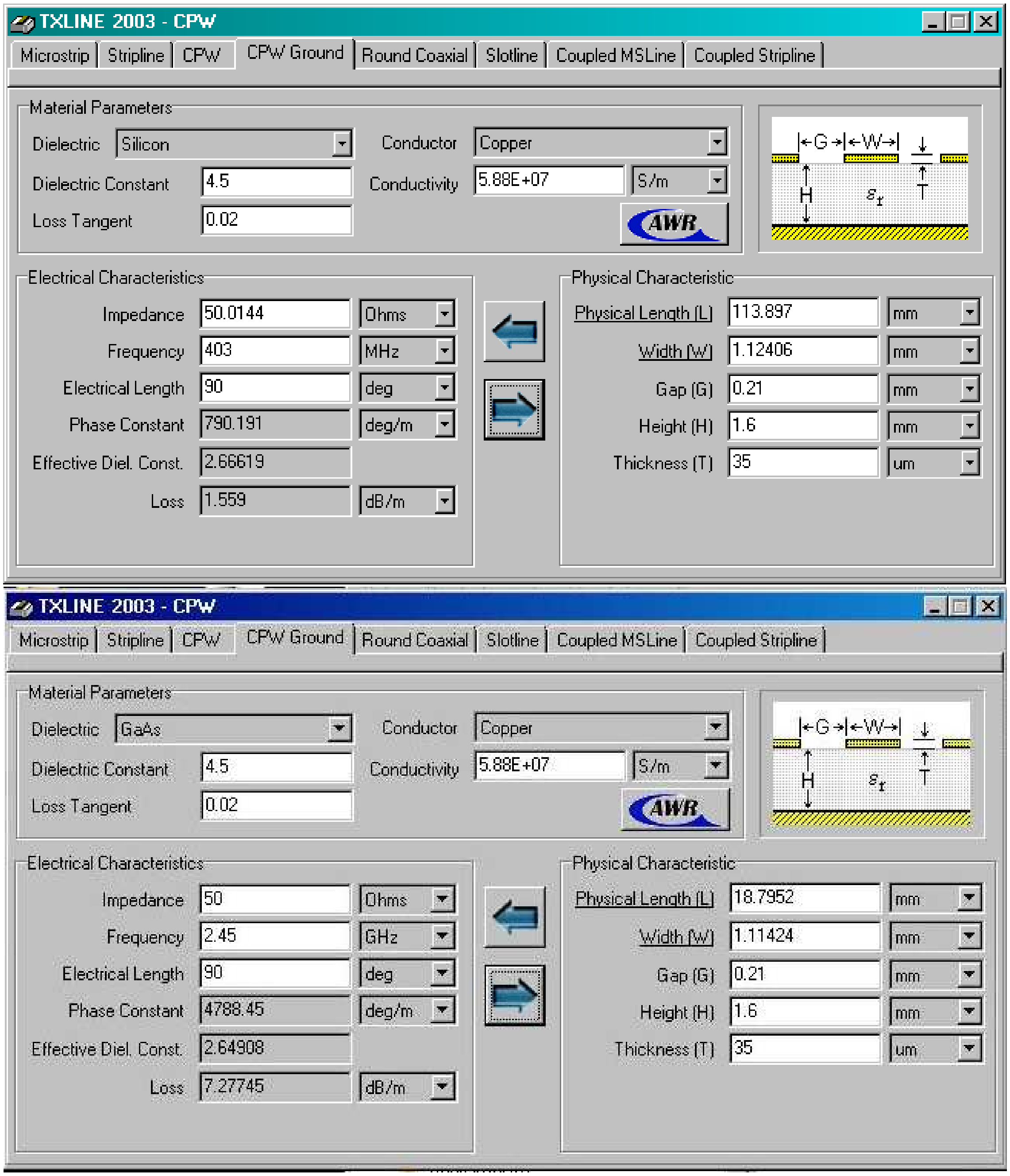

Figure 1.9: Calculation of the parameters for the cooper stripes between Zarlink and antenna.

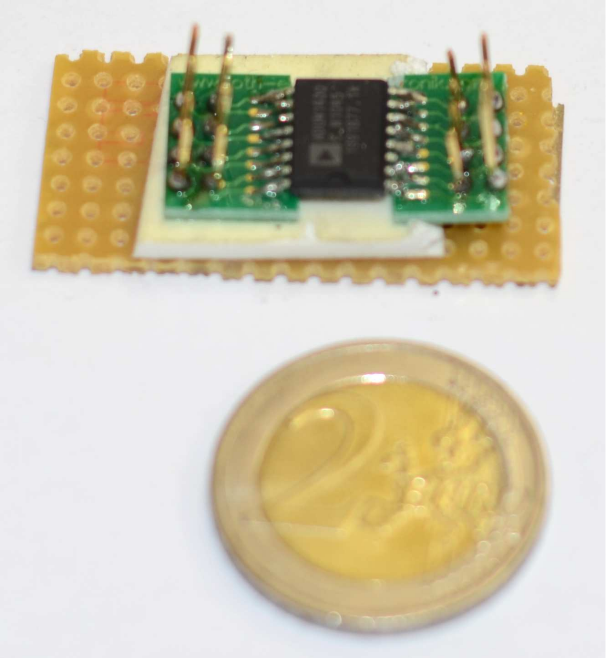

Figure 1.10: Digital isolator IC ADUM 1401 from Analog Devices in a 16 SOIC wide package on an improvised adapter (based on a 16 SOIC non-wide PCB).

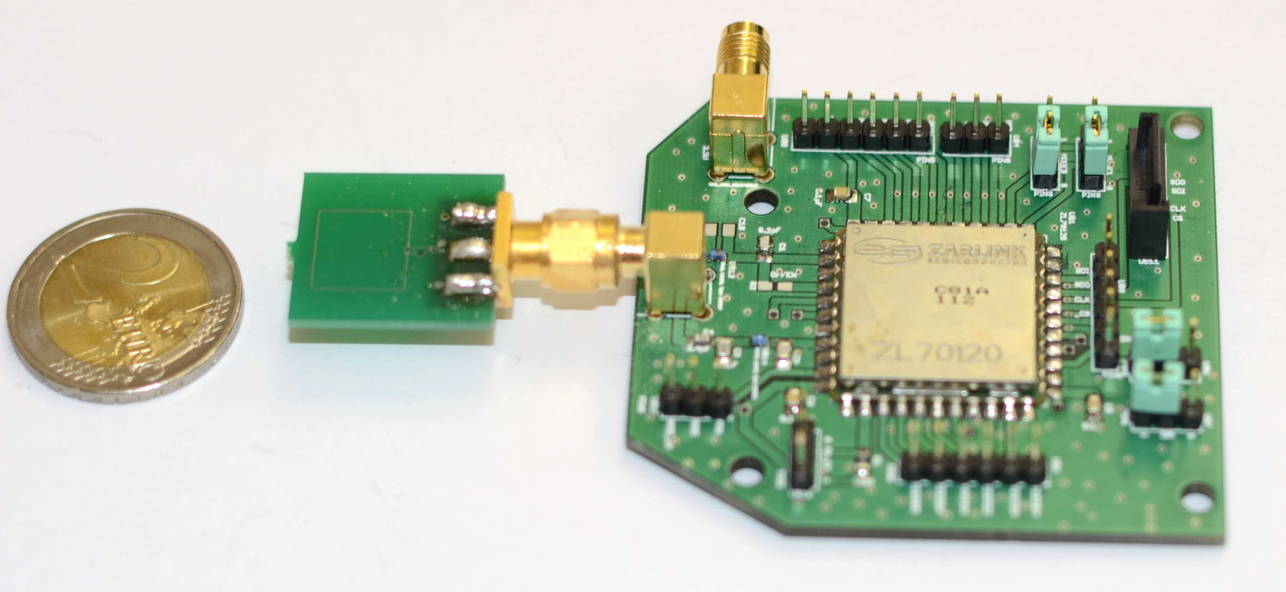

Figure 1.11: Second version of the Zarlink base station board with the base station antenna from Tim Schellenberg.

1.2.2 Implant module

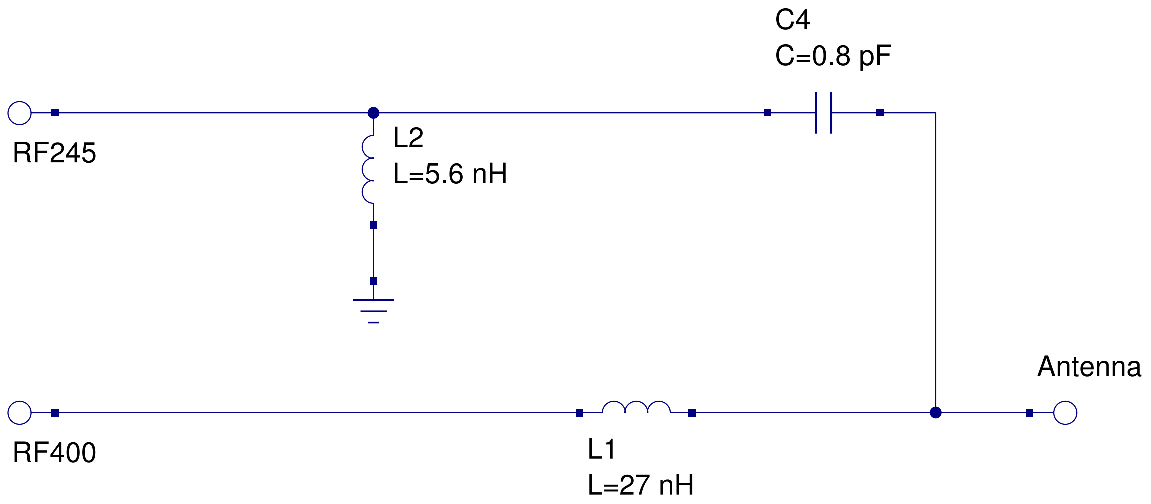

Figure 1.12: Antenna matching circuit taken from the Zarlink AIM200 reference design.

For the Zarlink implant module pcb, I used the same approach like with the base station module. The ZL70321 is optimized for antennas with $100 + j150\Omega$ for the 400 MHz link. For the antenna matching network (see 1.12), I used the reference design from Zarlink's AIM200 board. The main application for this board is to be connected to the ITEM's FPGA implant prototype via a Hirose DF 15 6-2 30 DP connector plug. Furthermore, the board had to be directly stacked and screwed on top the ITEM's FPGA board. This resulted in (hard) boundary conditions for the dimension of the board and positions of the holes (which were kind of meet) on the board.

The first version of this board looked like it is shown in the figures 1.13 and 1.14. A second version became necessary because I accidentally mirrored the Hirose connector plug and thus it was impossible to use the first version with the ITEM FPGA implant prototype. For the second version (not shown here), I added a SATA plug for connecting this module board to the base station FPGA for test purposes and an extra SMA plug for providing an external 3.3V supply voltage.

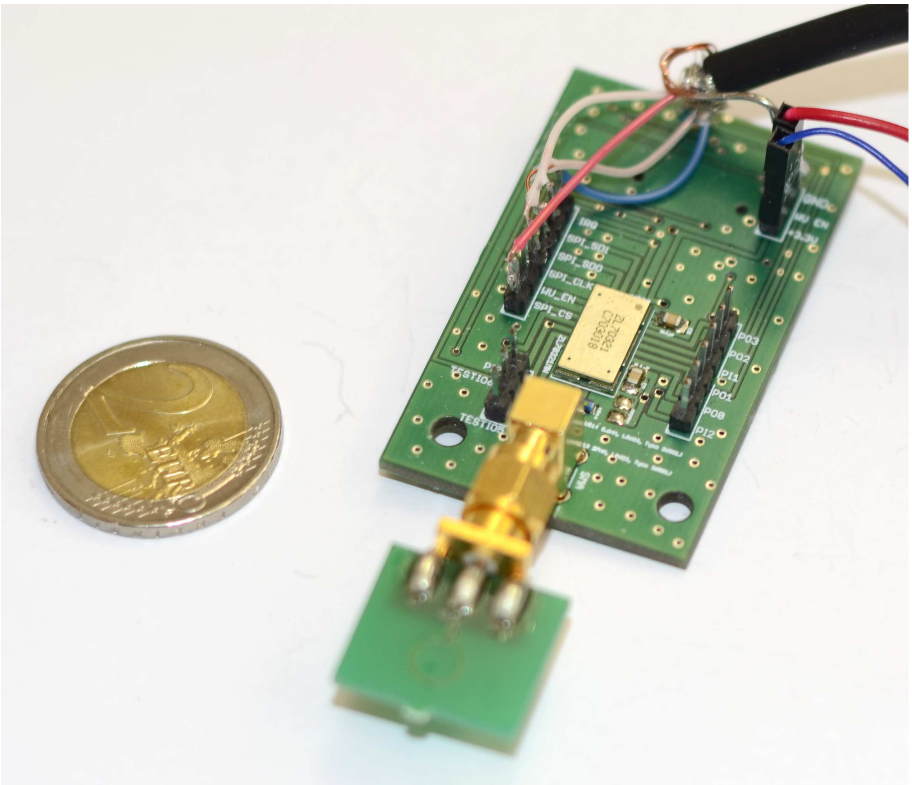

Figure 1.13: First version of the Zarlink implant module board with a ZL70321 MICS-Band RF standard implant module. For connecting this board to the base station FPGA, a SATA connector cable is soldered to a pin header with connections to the SPI pins of the ZL70321 (The Board was produced by PCB Pool and assembled by Janpeter Hoeffmann). Also shown is the implant loop antenna from Tim Schellenberg.

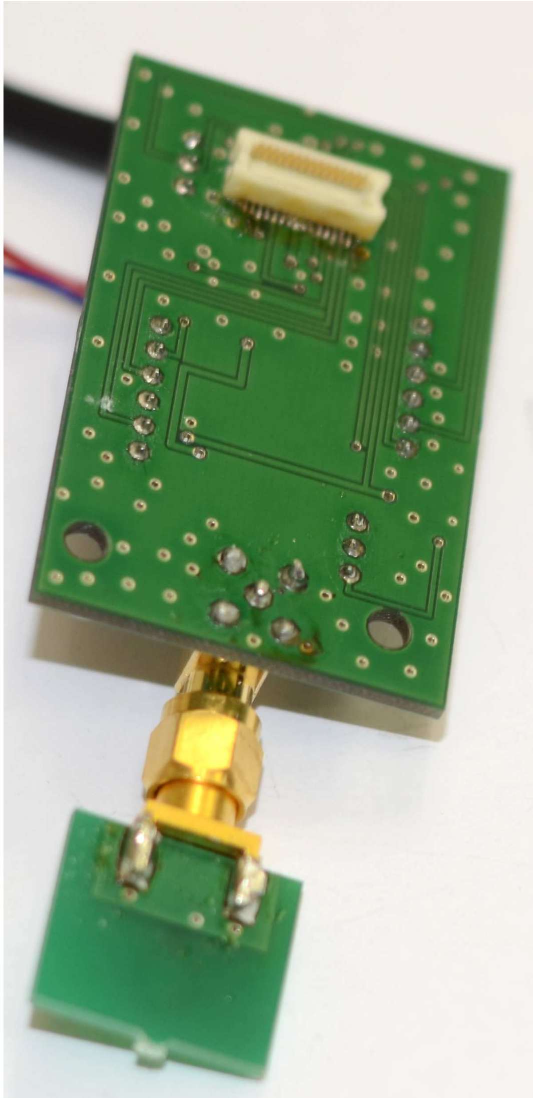

Figure 1.14: Flip side of my Zarlink implant module board with the (mirrored) Hirose DF 15 6-2 30 DP connector plug.

1.3 Trigger channels

In an experimental setup it is extremely important to know exactly what happened when. When more then only one device are part of an experimental measurement system (like e.g. an electro-physiological recording system and/ or a second computer, responsible for creating the visual stimulation) then the data streams from this equipment need to be synchronized. This can be archived by exchanging digital signals via so called trigger channels.

It would be possible to realize the required trigger-in and trigger-out channels with the base station FPGA's pins without any other components. However, there are two reasons why I decided to design additional trigger channel boards. First, the FPGA pins are not 5V tolerant and I wanted a solution that can be modified for 5V use if necessary. Second, the number of available user IO pins of the base station FPGA is very limited. A typical number for these trigger channels are 32 lines per direction. Hence we would lose 64 FPGA pins.

Thus it is desirable to find a solution that reduces the amount of required pins. I developed such solutions for the trigger-in and the trigger-out channels and realized them as modular extension cards for the base station. My solutions are mainly based on the 32 channel ADG732 analogue multiplexer ICs from Analog Devices and 74AC11074D dual D-flip flop ICs from Texas Instruments.

Both extension cards were designed for logic signals with logic level of up to 3.3V. Thus the trigger-out channels deliver only a maximum of 3.3V for a logic high. If higher voltages are required, an extra layer of drivers or logic level shifters are required to deliver TTL signals with 5V. The trigger-in channels are in a similar situation. One analogue multiplexer just routes the signal through to the FPGA. The used FPGA (Spartan 3A) can only survive (signal) voltages up to 3.3V. In a case where TTL 5V signals will be used, it is required to use a logic level shifter, voltage divider (if the resistances of the input sources are known and constant) or a 5V tolerant CPLD as mediator between the input pins and the FPGA.

As connector for the input / output of the cards, I used 2x20 pin headers (where the upper and lower 2x2 pin-blocks are just ground pins). In the middle of the header there are 16 signal and ground pairs. These pairs are in a 2x1 constellation. I designed it in this way because now it is possible to take a 2x20 ribbon cable and cut off one connector plug. Then we can singularise the wires of the ribbon cable and we will find in the adjacent wires a signal and ground pair.

1.3.1 Trigger-In channels

For the trigger-in channel card, I found a very simple solution (see figure 1.15 and 1.16). It is possible to sample the logic states of the trigger-in channels by selecting

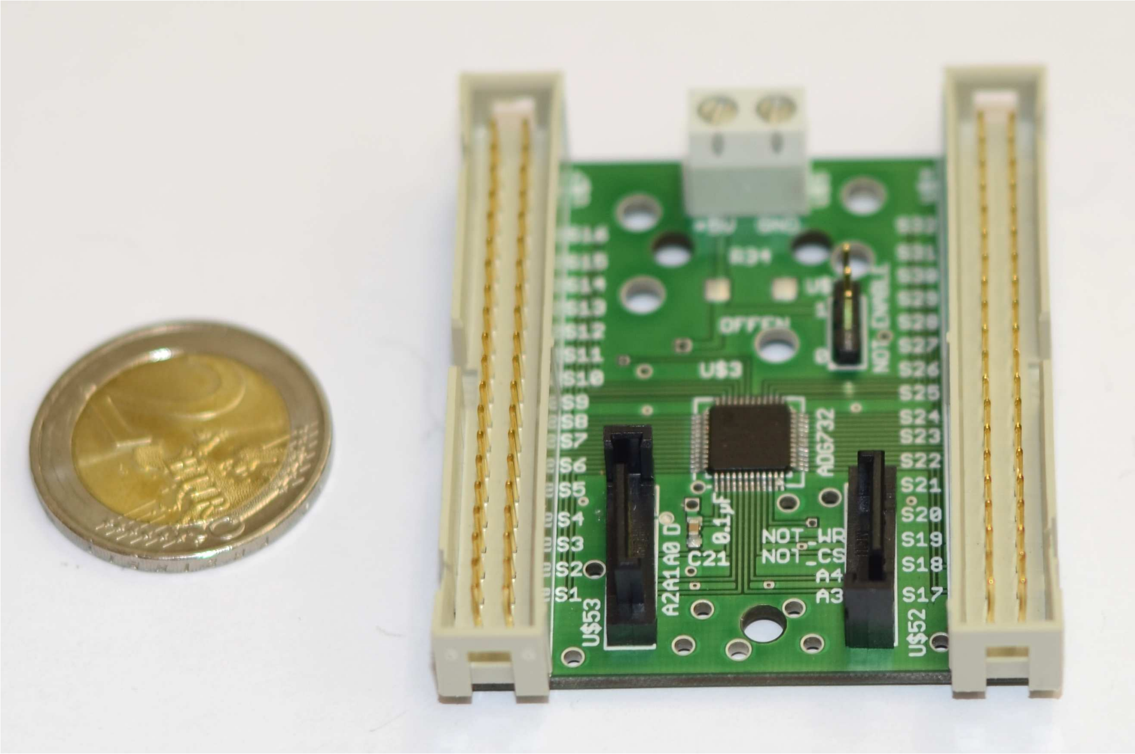

Figure 1.15: The 32 trigger-in channels card. Hardware realization of the concept shown in figure 1.16. The grey plug has to be connected to a 5V power supply.

(controlled by the base station FPGA) them with a multiplexer and feeding that signal to one input pin of the FPGA. As multiplexer I haven chosen an ADG732 analogue multiplexer from Analog Devices. This 32:1 multiplexer is driven by a 5V supply voltage and thus allows to route up to 5V signals (rail-to-rail operation). However, the FPGA's pins of the base station are not 5V tolerant. Hence only 3.3V logic level signals can be used as logical input.

Designing an input protection (protection against the wrong polarity or over-voltages) was postponed because the required parameters (e.g. source impedance) couldn't be determined. One idea for an input protection would be to put logic level shifters in sockets and use them as sacrificial elements.

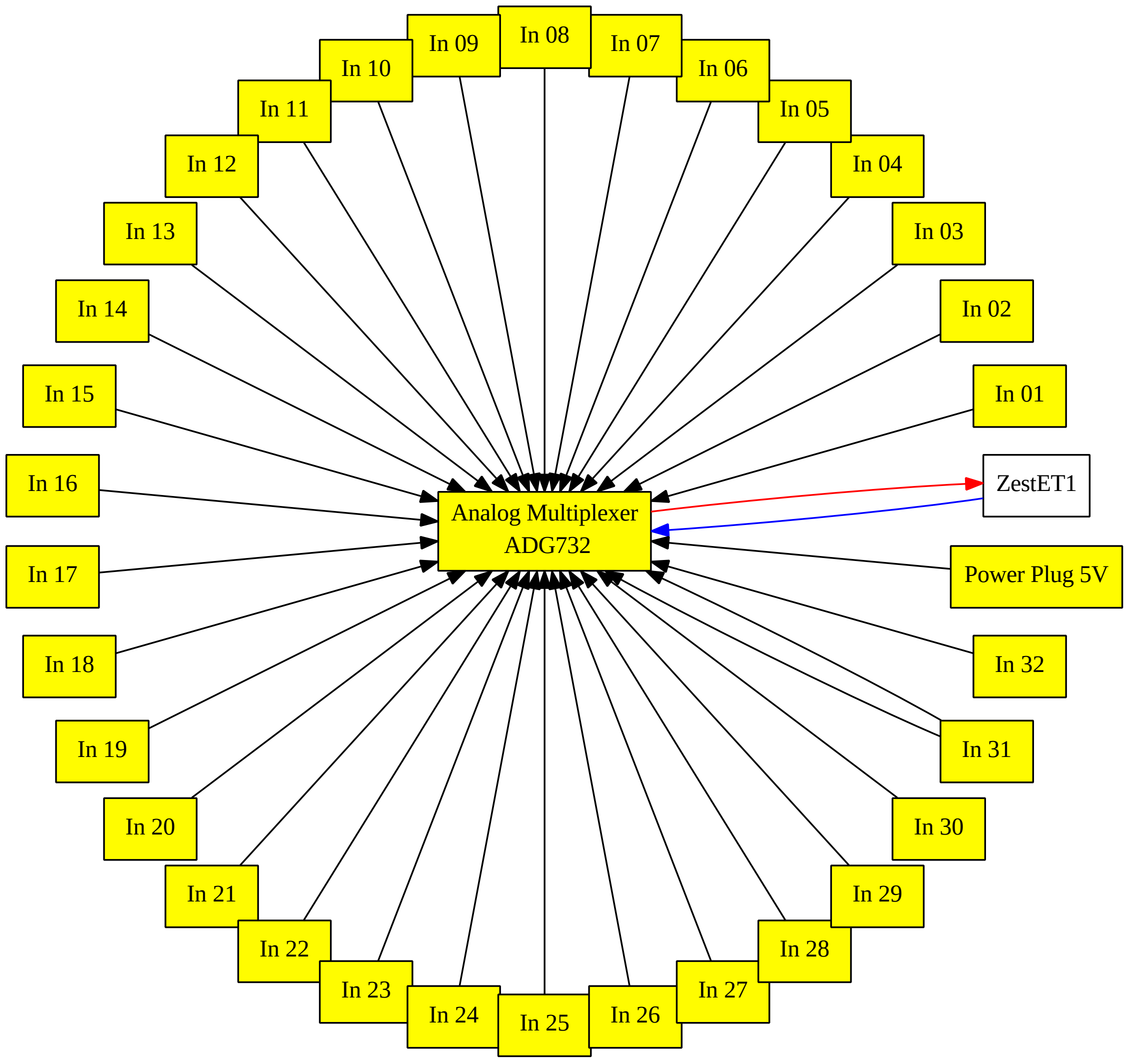

Figure 1.16: Structure of the the trigger-in channels card. An analogue multiplexer switches between 32 input channels. The selected channel is connected to the base station FPGA and there the channel's logic value is read out. The yellow elements are part of the trigger-in channels card. The blue line represents the connections, between the FPGA and the multiplexer, for selecting the channel. The red line symbolizes the data coming out of the multiplexer and going to the FPGA.

1.3.2 Trigger-Out channels

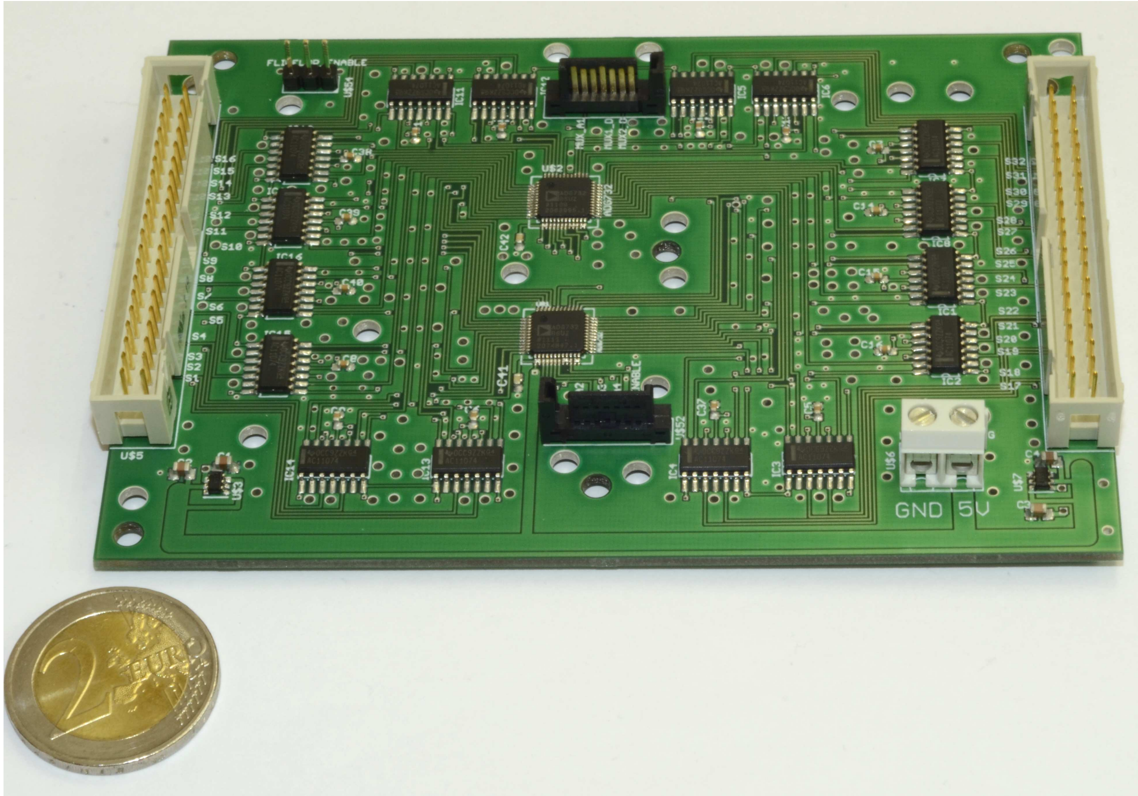

Figure 1.17: The 32 trigger-out channels card. Hardware realization of the concept shown in figure 1.18. The grey plug has to be connected to a 5V power supply rail.

For the trigger-out channels, I used the unusual combination of two ADG732 analogue multiplexer and 16 dual D-type flip flops 74AC11074D ICs from Texas Instruments. One multiplexer is used to connect the data-in pin of the selected flip flops to the base station FPGA's pins. The second multiplexer is used to route the clock pin to the selected flip flop. The concept behind this approach is that the output of the D-type flip flop stays constant until the next update of its logic state. This also works fine if no clock signal is applied for a very long time. Thus it is possible to select one of the 32 flip flops and update it individually, while all the logic states of all the other flip flops are kept untouched.

Two errors found their way into the produced design. For unknown reasons, I connected the negated output instead of the non-negated output of the D-type flip flops with the pin header for the outputs. I fixed this problem by simply negate the logic data output of the FPGA for compensating it. The second error concerns the ADP3330-3.3V DC/DC converters (from Analog Design) producing the 3.3V supply voltage for the flip flop ICs. Due to the high number of these ICs, I decided to have two of these DC/DC converters on the board. While creating the design, I assumed that I am allowed to connect the outputs of both converts. Retrospectively, I found no

hint in it's manual that this is possible (and that the feedback loops of the converters would survive it). Thus I decided, before I started with testing the board, to cut this unnecessary connection by grinding away parts of the cooper stripe on the flip side of the board.

Tests showed that the card works without any problems now. Nevertheless, it is not capable of producing TTL 5V outputs. One way would have been to drive the flip flops with 5V supply voltage. But in this case it is not clear if the output pins of the FPGA pins are capable of delivering the required logic levels voltages for the flip flops in 5V mode.

It may have been possible to use only one multiplexer for the clock line and connect from the FPGA a common data line to all flip flops. I haven't done that because it wasn't clear for me how strong the delay, created by the multiplexer in the signal path, really is and I wanted to prevent runtime difference between the data and clock signal.

1.3.3 Alternatives

The presented trigger-in and trigger-out cards work fine. Nevertheless, there is a lot of potential for improving the design. One way would be the use of a 5V tolerant CPLD (like e.g. Xilinx XC9572XL which would provide 72 user IO pins). Not only would this reduce the number of components, especially for the trigger-out card, it would also allow to change the input/output properties of the channels dynamically. Depending on the experimental setup and the requirements, the pins could be distributed freely to the trigger-in and trigger-out channels (which easly can create confusion and destroyed hardware). However, the trigger-in channels would be 5V tolerant in when using this type of CPLD but the trigger-out channel would still deliver a maximal voltage of 3.3V for a logic high. Thus for real TTL 5V outputs, there are sill logic level shifters or drivers necessary.

Another way of realizing real TTL 5V outputs is the application of 74HCT595D buffered 8-bit shift register ICs. Given a 5V supply voltage, 5V signal for a logic high are generated (I have not checked if the 3.3V for a logic high from the base station FPGA is able to drive the input of these shift register in 5V mode). These buffered shift register ICs can be daisy chained easily, allowing to drive many trigger-out channels with only 3 IO pins from the base station's FPGA. The disadvantage is that the update rate of an individual output channel is limited by the maximal frequency (30-108 MHz depending on the supply voltage) divided by the number of trigger-out channels. The reason for this is that the data has to be shifted through all the daisy chained ICs before the update of the output can be initiated. In this respect the solution with the two multiplexer is better because it allows to select one individual D-flip flop with maximally 50MHz and update it with up to 100MHz. At least in theory.

For protecting the base station FPGA from too high logic signals or reversed polarisa-

tions, it would be possible to use digital isolators (see figure 1.10) as sacrificial element within the signal lines of the FPGA. But due to the bad experience with the digital isolators in the context of the Zarlink base station module, I didn't used them in my later designs.

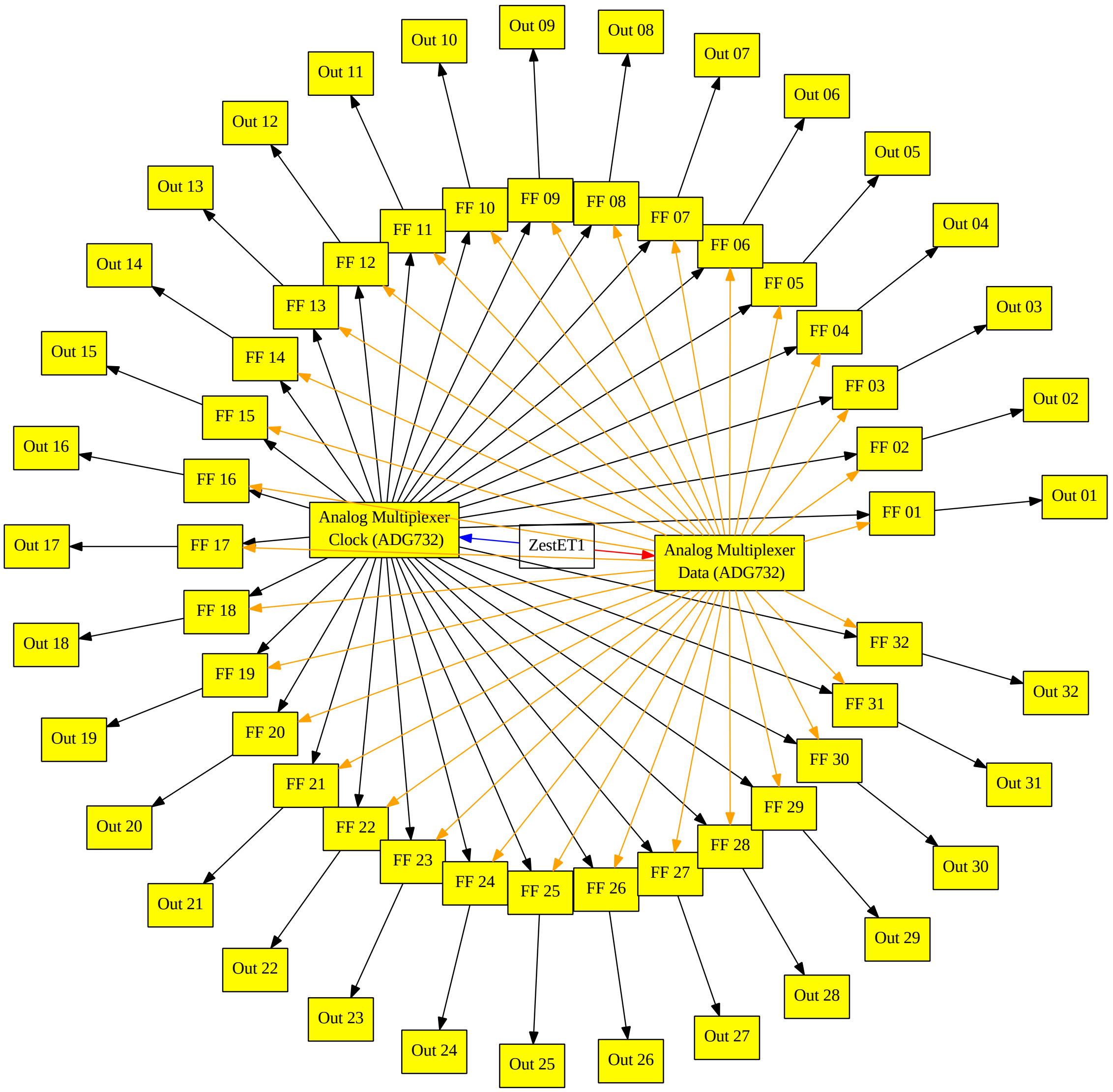

Figure 1.18: Structure of the trigger-out channels card. The FPGA on the base station controls two 32 channel multiplexers. With the use of these multiplexers, the FPGA is able to select one of the 32 D-type flip flops (FF). After selection, the FPGA provides the clock and data signal to the selected flip flop and updates its logic state. The 32 flip flops are provided by 16 dual D-type flip flops 74AC11074D ICs from Texas Instrument, which are driven by a 3.3V supply voltage. The two 32 channel ADG732 analogue multiplexers are driven by a 5V supply voltage.

1.4 Digital-to-analogue converter (DAC)

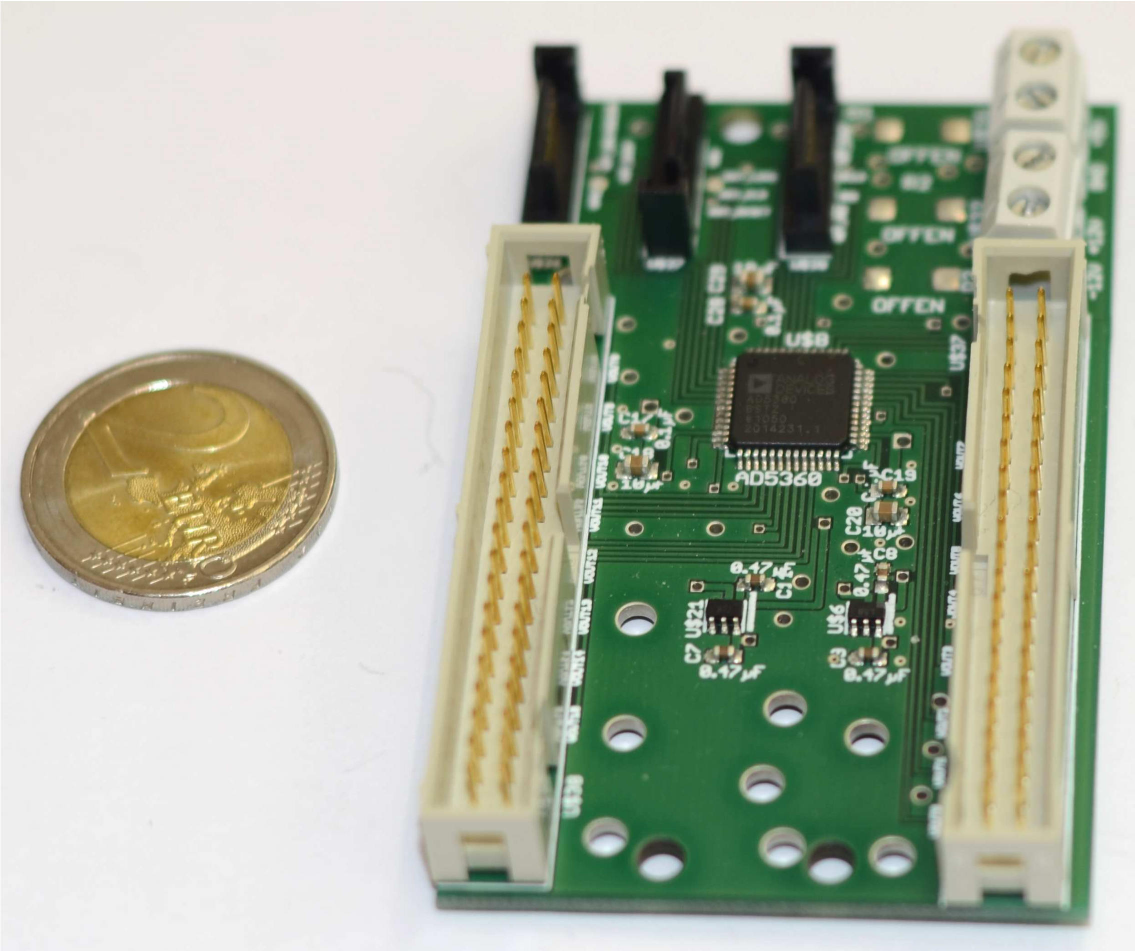

Figure 1.19: Extension card that provides digital-to-analogue conversion capabilities for the base station. It implements 16 DAC channels, allowing to produce analogue waveforms with amplitudes between -5 and 5V in 65536 steps. In figure 1.20 the structure of the board is shown. For supply voltage, the board needs 5V, 12V and -12V rails.

It is very important to note that this DAC design is NOT intended for medical applications! For example, it doesn't provide the necessary safety measures.

Besides emitting binary encoded information via the trigger-out channels, some times it is necessary to produce time series of analogue voltage values. One application is to control another device with the generated waveform (e.g. speaker). But more interesting is a different range of use: The (closed-loop) electrical stimulation of brain tissues.

The heart of this extension card (see figures 1.19 and 1.20) is an AD5360 from Analog Devices. This DAC provides 16 channel with 16-bit resolution. For the board, a

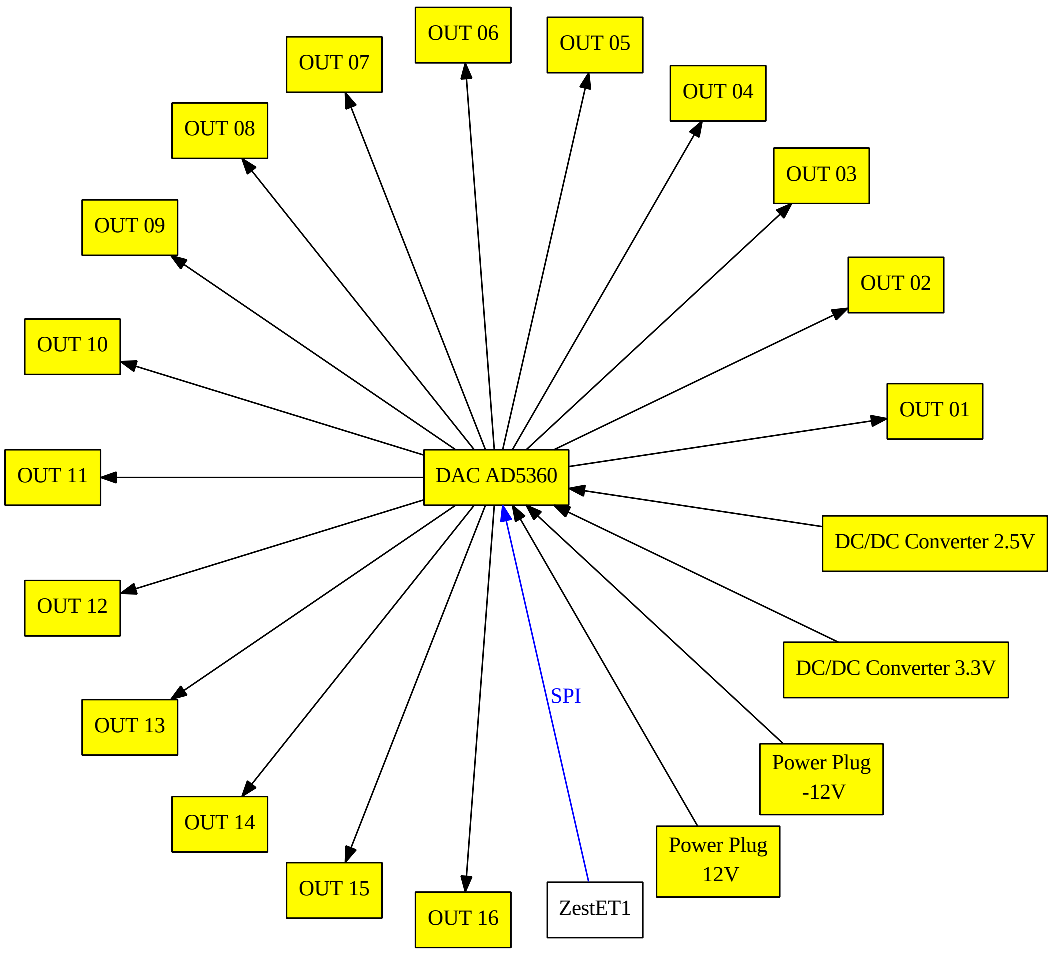

Figure 1.20: Structure of the DAC extension card. The heart of the board is an AD5360 from Analog Devices. This is a 16 channel 16-bit digital to analogue converter IC which can be programmed by the base station's FPGA via the SPI protocol.

reference voltage of 2.5V is used. This allows to generate voltages between -5V and +5V as output, with approximately 0.15mV resolution. Removing the DC/DC converter, which generates the 2.5V reference voltage from the 5V supply voltage, and connection the reference pin with the 5V power supply, would allow to generate voltages between -10V and 10V. However, it is important to provide a stable and clean reference voltage. Depending on the application, it can be necessary to use a reference voltage source IC.

The DAC is controllable via SPI protocol. The digital circuitry is driven by 3.3V. The 3.3V voltage is provided by a second DC/DC converter which is also powered by the 5V power rail.

Due to time constrains in the project, it wasn't possible to include this accessory card within the functionality of the base station firmware yet and hence I haven't tested it yet. I expect a work time of 30 hours to introduce the missing part to the firmware and

test the hardware. The manual of the DAC IC claims that it is possible to program at least one million new analogue value per second. Furthermore, the handbook states that it takes at least 30 μs for the new analogue value until it is settled.

1.5 Analogue-to-digital conversion (ADC)

The goal was (and still is) to create analogue input channels as addition for the base station, e.g. usable for electro-physiological measurements or analogue eye positions measured by an eye tracker. The first question is: Why do we need our own ADC channels for the base station and can not use commercially available models? The answers are: latency, accessibility and scalability.

1.) Latency: An important issue is delay between different signals. If we want to characterize the delay between the 'real' neuronal signals and the data stream which was measured by the implant and later received by the base station then it is very important to remove all extra sources of timing jitters (e.g. by an extra operation system or USB interface). In the case of a commercial electro-physiological recording system, the data is collected by an external PC. This data stream has to be reliably integrated (with a fixed and known latency) into the data stream from the implant in a synchronous fashion. It has to be noted that the base station and such an external recording system have two independent imperfect clocks plus an operation system and some interface structures in between. This doesn't make such an ongoing synchronisation an easy task.

If the ADC channels are operated by the FPGA of the base station then timing delays between the neuronal signal and the recorded values are known with high precision and reliability. Thus the data from the ADCs can be integrated into the data stream of the implant as comparison. In addition, this low latency allows, as a kind of side-effect, to build close-loop systems that allow to react to a measured neuronal activity pattern nearly instantaneously (if the incoming data can be directly processed on the FPGA of the base station) with a defined electrical stimulation response. This feature is very important for functional neuronal prostheses (e.g. artificial visual cortex prostheses) and newer research approaches.

2.) Accessibility: A lot of the commercially available recording systems don't even allow to export the data in real-time. This makes it necessary to reverse engineer and hack their software and drivers. Our own system allows us to access the recorded data in real-time with low and defined latency. We can define and implement the necessary interfaces ourself and change it later if it gets necessary.

3.) Scalability: In the future, due to improvements of the implant, the number of it's electrodes will rise. Allowing simultaneous wirebound recordings of these electrodes require a recording system which also can be scaled in the number of channels while keeping the latency and accessibility problems under control at the same time.

Beside these three main requirements, other boundary conditions are defined by recording electro-physiological signals which contain slow local field potentials as well as fast single action potentials. Relevant analogue signals like e.g. single individual action potentials show a band-width with up to several kHz. Thus we decided to go for an

analogue-to-digital conversion rate of 25k samples per second and per channel. For measuring the relevant components of the neuronal signals, we need to measure signals with a resolution of sub μV precision. On the other side, we have to expect DC components on top of the signals with several 100mV, which the analogue front-end needs to be able to ignore. For making the design of the analogue front-end (amplifier, low-pass, high- and band-pass filter) a bit more easier, we decided to base our ADC channel design on a 24 bit ADC IC from Analog Devices (the AD7766 model with maximal 32k sample per second). This is due to the fact that a 24 bit ADC can resolve a time series finer in voltage space than a 16 bit ADC. Thus a lower pre-amplification of the analogue signal is necessary. Furthermore, we decided to place the cut-off frequency for the high-pass filter at 1Hz and the cut-off frequency of the low-pass filter at 10kHz. Another requirement for the analogue front-end was that the phase response of the combined filter front-end shows as less as possible dispersion on the phase within the relevant frequency range.

It is important to note that the AD7766 has an additionally build-in 4-stage FIR digital low-pass filter which makes the filter design a bit easier. The number of stages depends on the IC model type. The lower the sample rate of the IC, the more efficient the low-pass filter can does its work. When the 32k samples per second - model type is driven with it's maximal sample rate, then it's low-pass filter cuts off at around 15kHz and drops to -110dB at around 17kHz. In this case, the IC needs to be driven by a 1.024MHz sampling clock and produces from 32 internal collected samples one sample which can be read out by the SPI bus. Since we aim for a 25kHz sample rate, we need to drive the ADC with a 800kHz clock signal.

We decided against using a faster ADC in combination with an analogue multiplexer. Such a combination would have allowed to reuse the same ADC for several input channels and would also have allowed to reduce the number of ADCs. The reason why we choose to go the way with the more hardware components was the fear of signal distortion and pollution by the switching process of the multiplexer on our weak electro-physiological signals.

An important parameter for the analogue front-end is the amplification factor. This factor is determined by the amplitude of the relevant signal component and the effective voltage resolution of the ADC. On the brain tissue / electrode side, the available amplitude is determined by the realised interface. It's properties depend on many parameters like e.g. the shape of the electrode, how much fluid is between the electrode and the brain tissue, and how many scare tissue grew around the electrode. On the other side, the voltage resolution of the 24 bit ADC depends also on many factors like supply voltage stabilities, the quality of the analogue ground connection and the influence of noise (and 50Hz mains pollution) collected by the recording system. In a real world application, the 24 bit ADC loses several meaningful lower bits. At the end of the day, it is not possible to name 'the' optimal amplification factor. Thus we aim for a recording system with programmable amplification factor (independent for each individual input channel with its individual, over time changing biological interface)

with the rough range of 400x - 5000x. Typically in close proximity of the recording site a so-called headstage is used. This is a low-noise pre-amplifier which is placed very close to the electrodes (directly on the head). Such a pre-amplifier already provides an amplification factor of 4x - 10x.

I developed the requirements for the filters of the analogue front-end and tested the complete system in close cooperation with Andreas Kreiter. Dieter Gauck helped us with many test measurements. When finished we plan to publish the design of the ADC channels as open source hardware.

In the following, I will present different evolutionary states of the ADC channels that I have designed over the project time. For me it is important to note that the measurements which I will present, were made in a completely non-optimal environment (e.g. no shielding and many sources of inference). Since I am far from an expert in operating a measurement system, the results have to be understood as rough characterisations instead of a real analysis of the recording system's properties. Even the generation of the test signal, using a Hameg HM8131-2 arbitrary waveform generator, in combination with BNC signal attenuator connector pieces was far from optimal. I also had to use the waveform generator at its minimal amplitude value (sinusoidal waveform with 20mV peak-to-peak), which sometimes caused strange behaviours (e.g. waveform distortions). Also the waveform generator used the same mains as the recording system, which is a bad idea in itself. However, my goal with the measurements was roughly to test the functions of the designs. Doing real meaningful measurements, I reserved for an experimentalist who really knows what he/she is doing.

Despite all the effort we haven't found the optimal analogue front-end yet but we have already achieved promising intermediate results.

1.5.1 Version 1

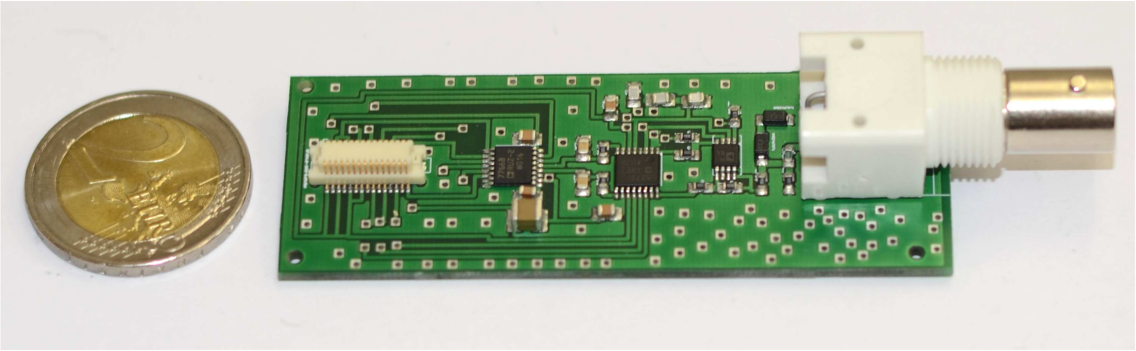

Figure 1.21: First version of the ADC channel as a small plug-in module with one channel.

The design of the first ADC channel module was mainly driven by naivety (see figure

1.21). For me it was clear, that the analogue-to-digital converter IC should have as much bits per sample as possible. This would allow me to skip the highly complex design of a real analogue front-end with all its filters. As a security margin, if the voltage resolution of the ADC would not have been good enough on its own, I wanted an extra digitally programmable amplifier. Thus I checked the main vendors for ADCs with as high as possible numbers of bits and a sample rate of around 30k samples per second. First, I found some 32 bit ADC which were much too slow but in the end, I choose a 24 bit ADC from Analog Devices. After four versions of ADC channels, I can claim that this AD7766-2 (32k samples per second version) was and is still the right choice. Via SPI, it can easily be connected to the base station FPGA with only a few connection lines. Multiple IC can be daisy chained which saves FPGA pins and brings its own highly efficient low-pass filter. However, the AD7766 has differential analogue inputs which was new to me.

Thus I thought that I need a fully differential and programmable amplifier (variable gain amplifier = VGA). The number of options for such ICs are very limited. Most of them are not usable for low frequency application and are aimed at high speed telecommunication systems. At the end of my research, I found the AD8370, which has a working range of LF to 750MHz. However, LF is not explicitly defined in its manual. The manual of the AD8370 explains in one example how to convert a single line input into a fully differential signal via an AD8138 fully differential driver. Thus I included the driver into my design. I also got the idea that I have to protect the components against too high input voltages. I found the 'usual' standard input protection for limiting the amplitude within a defined voltage range, based on one resistor and two Zener diodes. As result I got the structure shown in figure 1.22. The other required components I copied from the three manuals. As final 'analogue front-end' I got the circuit diagrams shown in figure 1.23.

The next important design decision was to create slim modules with single ADC channels which simply can be plugged on top of a motherboard - construction and easily exchained when broken. Thus I decided to employ the Hirose connectors that are used in the Zarlink test kit. The connectors are available in a version that show the necessary board-to-board distance. I made the decision that I want to test the ADC modules without constructing the corresponding mainboard.

After the PCBs were produced and assembled by PCB pool, I wanted to start with the first tests. This was the moment when I realized that the pitch of the connector was such small that it was very problematic to handle them without a motherboard. Luckily, Robert Huetten was able to build an adapter for me. In summary, the test was a small disaster. First of all, the 'input protection' worked to good. Thus no signal reached the driver because the 50 Ω input of the driver circuit wasn't suitable for the input protection (A simulation of the circuit would have shown the problem in advance). As a workaround, I had to de-solder the two Zener diodes and to short-cut the resistor with solder. Without that input protection, I was able to record my first signals from the signal generator. Together with Andreas Kreiter, we found that the

VGA was distorting the analogue signal. It increased the slope of the test sinusoidal wave (like it is seen for $sin(\alpha t)^{\beta}$ with $\beta > 1$). This distortion was depended on the amplification factor. Furthermore, we figured out that to combine the digital and analogue ground plane everywhere on the module was not a good idea (e.g. due to noise pollution and debugging options).

As a result, we decided to learn from the mistakes and thus I redesigned the PCB without the input protection and some other improvements.

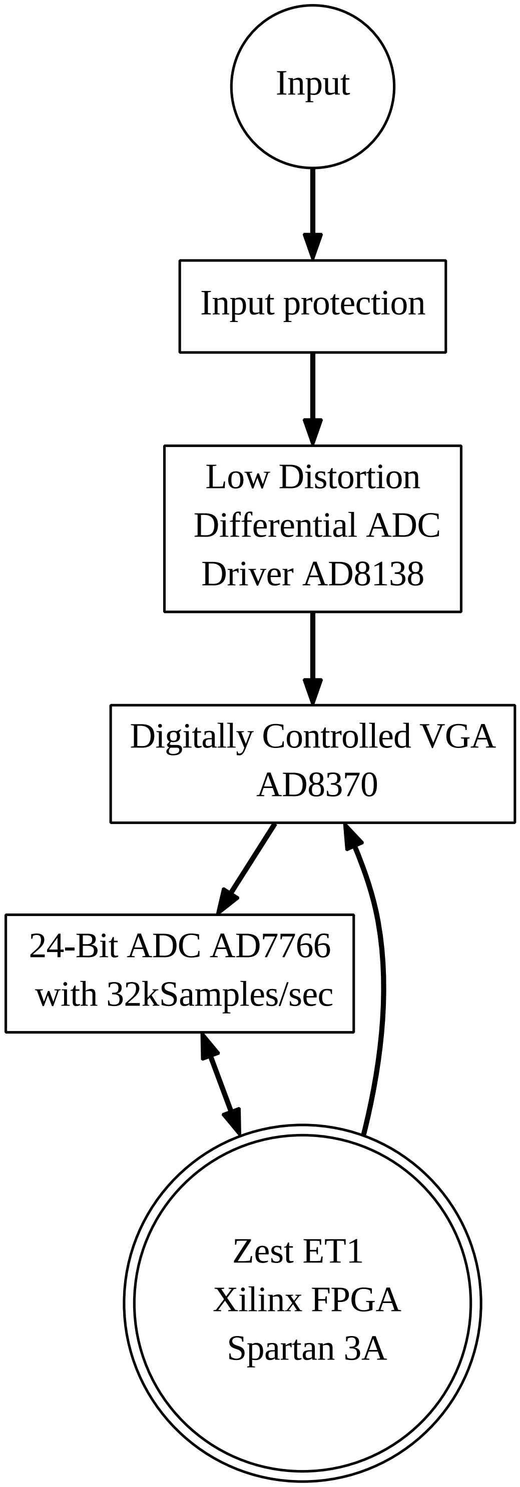

Figure 1.22: Information flow in the original planned ADC channel (version 1). The module has no optimized analogue-front end and consists out of a very low number of components. The idea was to have an input protection, followed by a fully differential driver. After this driver, a digitally programmable fully differential variable gain amplifier boosts the signal strength and delivers the analogue signal to the ADC.

Input-stage with fully differential driver:

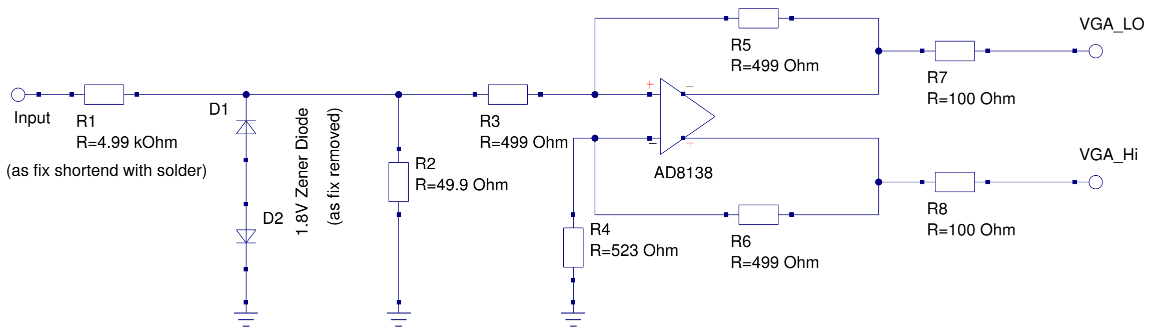

Variable gain amplifier stage:

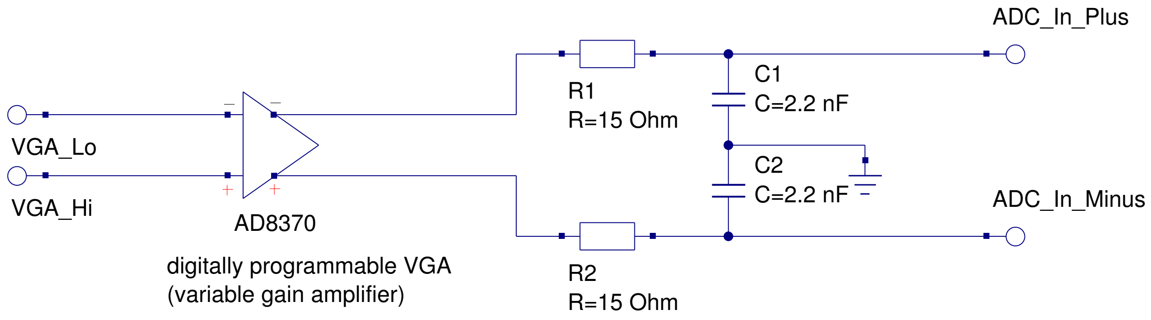

Figure 1.23: Circuit diagram of the 'analogue front-end' of the first ADC design. The input protection part (mainly the two diodes and the single 4.99 kΩ resistor before them) is a standard setup. The following parts were frankensteined together from the AD8138, AD8370 and the AD7766 manual.

1.5.2 Version 2

Based on the experiences with version one, I re-designed the ADC modules and prototyped a first connector board. There are several changes in design strategy. First of all, the module was conceptualize as stackable module. The idea was to daisy chain 16 of these modules via pin headers but keep the VGAs individually programmable. In addition, the power supply was reduced to one main DC power rail with 10V and a secondary power rail for the digital parts of the ADC and VGA ICs.

ADC channel

Input stage with driver:

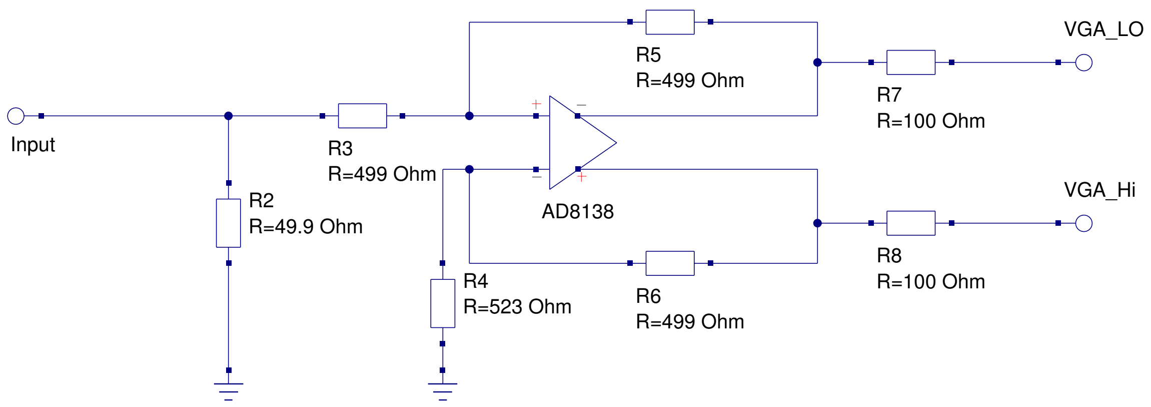

Programmable amplifier stage:

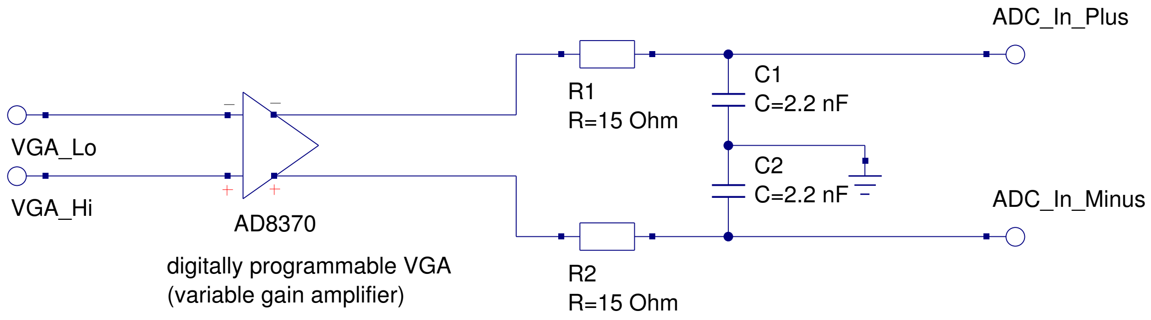

Figure 1.24: Improved version of the analogue front-end of the first version (see figure 1.23). The main difference lies in the removal of the 'input protection' part.

The analogue front-end of the ADC channel (see figure 1.24) of the second version stayed, with exception of the input protection, the same. The input protection components were completely removed. Placing and routing the ground plains and connections was done with much more care. I tried to keep them separate as long as possible.

Two major changes were introduced into the design:

1.) The power supply was reduced to a common 10V DC power rail. This rail supplies power to all the components except the digital IO parts of the ADC IC and VGA IC. It would have been also possible to generate this 3.3V supply voltage from the 10V power rail but I decided to get this voltage directly from the ZestET1 board. My reason behind that was, that I wanted to ensure that the logic levels of the FPGA and the ADC ICs as well as VGA ICs are the same. With the 10V power rail, I operate directly two reference voltage sources and a 5.0V DC/DC converter. One reference voltage (2.5V) is used for the driver IC and the other one provides the necessary reverence voltage for the ADC IC (5.0V). The output of the 5.0V DC/DC converter is used to drive the three main ICs, using an additional 2.5V DC/DC converter. Figure 1.25 shows the structure behind the module.

2.) In version one the module was designed to be individually stacked on a motherboard. In version two, my idea was to directly daisy chain the modules via pin headers. This reduces the size of the motherboard, which is more or less degraded to an simple adapter board. The ADC ICs are prepared to be daisy chained, under the cost of increasing the SPI clock frequency. I decided that 16 channels is a good number for one chain. First of all, this keep the SPI clock frequency within reasonable limits. Second, the length of the daisy chained modules are still below the 19" mark which can be found in a typical mounting rack.

However, the VGA IC doesn't provide such a simple possibility for stacking them. It is possible to daisy chain them but then all the VGAs are programmed with the same amplification setting. In contradiction to that, I wanted the channels to be individually programmable. This made it necessary to connect the individual chip select pins of the VGA ICs directly to FPGA pins. Now it was possible to select and program only one (or if desired more) VGAs at one instance.

Since it was not possible to design and produce 16 different modules, I came up with the following trick for the chip select (CS) line routing: CS line one of the modules was always connected to the VGA on the module. The rest of the CS lines between the input and the output header were shifted by one. Or in other words: CS line two enters the module and leaves as CS line one. CS line three goes in and leaves as CS line two, and so forth. This was done for all the 16 CS lines.

For reducing the time for this re-design cycle, I assembled the modules myself and PCB pool only produced the PCBs. First I produced a module with the VGA (see figure 1.26). The test of that ADC channel showed that the signal-to-noise was improved but the distortion of the signal shape was still present. Thus I started to assemble the ADC modules without the VGAs and closing the generated gap in the signal lines with mod-wires (see figure 1.27). We also tested different reference voltages for the ADC ICs as well as different amplification factors of the driver by replacing the resistors within the feedback loop of its amplifier. The results without the VGA were promising and where the basis for the third version of the ADC module. It was also possible to

confirm that stacking 16 channel modules can be expected to work.

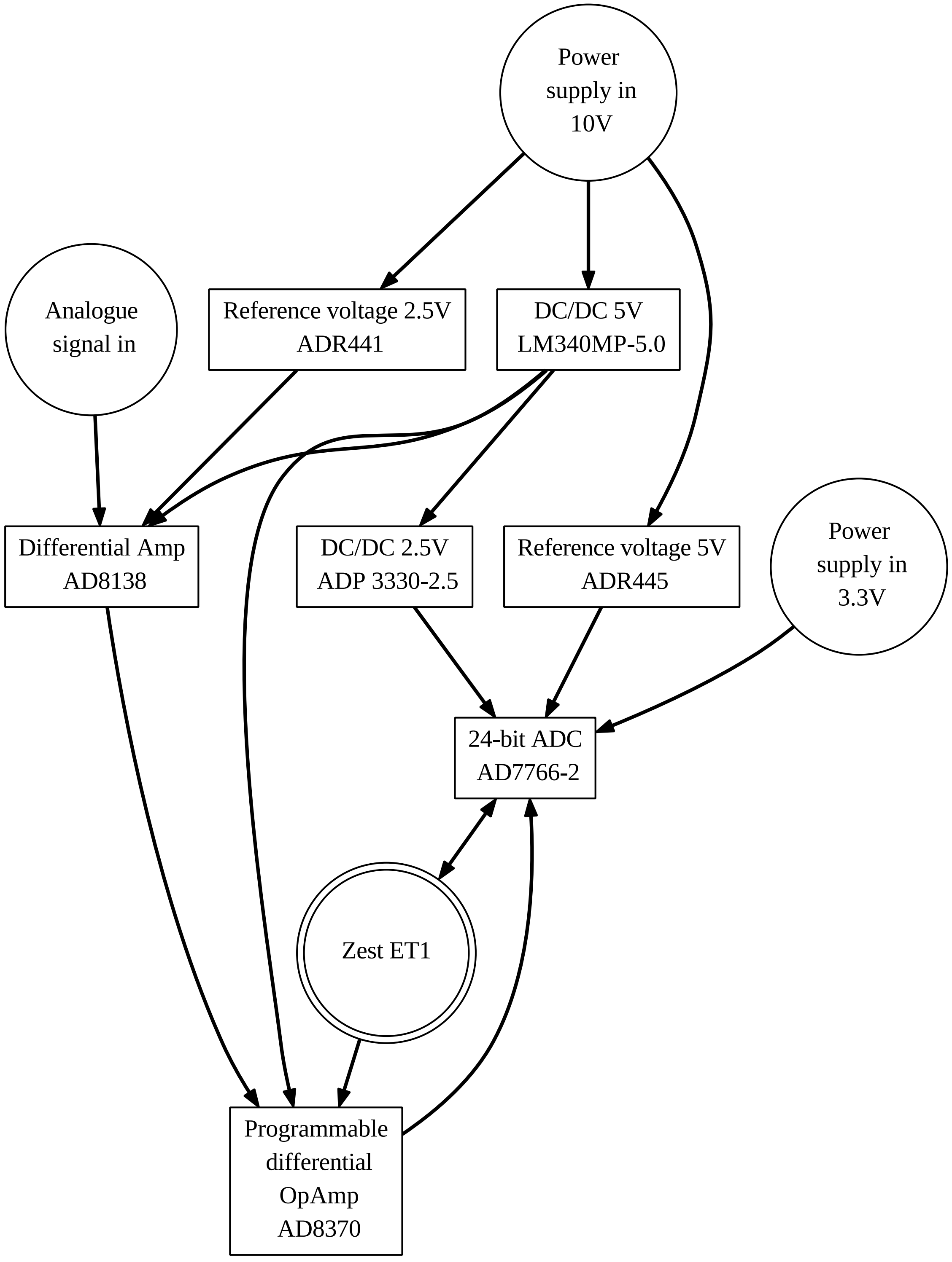

Figure 1.25: Structure of the second version of the ADC module. The biggest change in design lies in the power management. In this new version, the module gets power over a 10V rail for all the components except the 3.3V for the digital IO part of the ADC IC and the VGA IC. Furthermore, now the module hosts two reference voltage sources. One is for the driver IC (2.5V) and one for the ADC IC (5.0V).

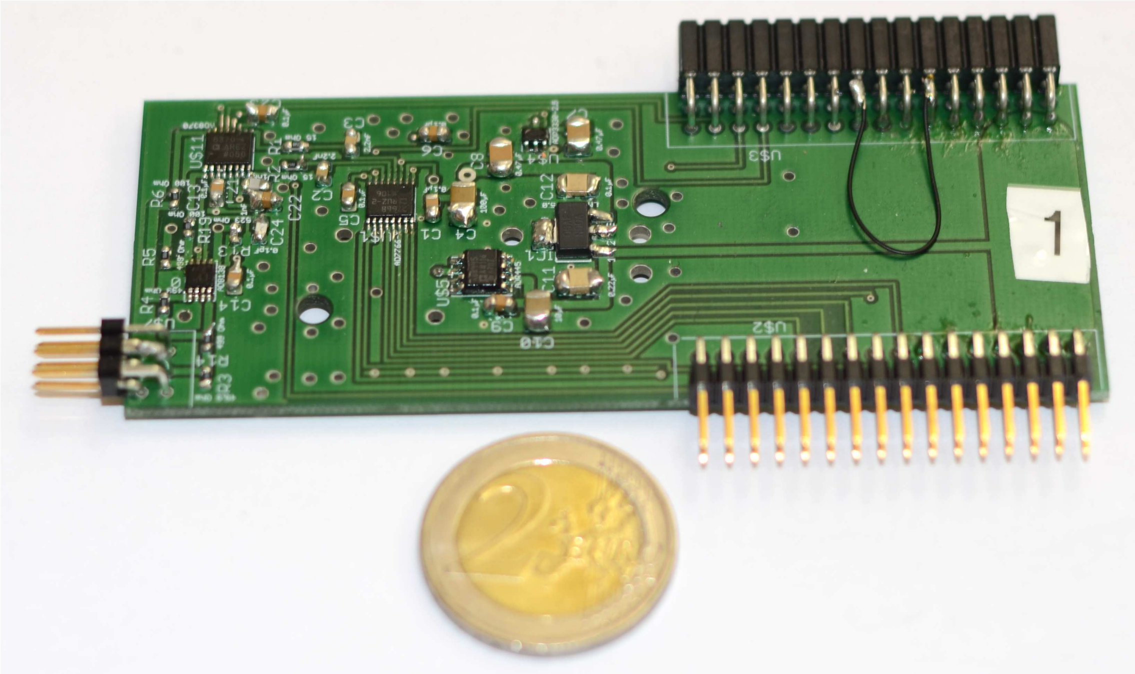

Figure 1.26: Realization of the module with the structure shown in figure 1.25.

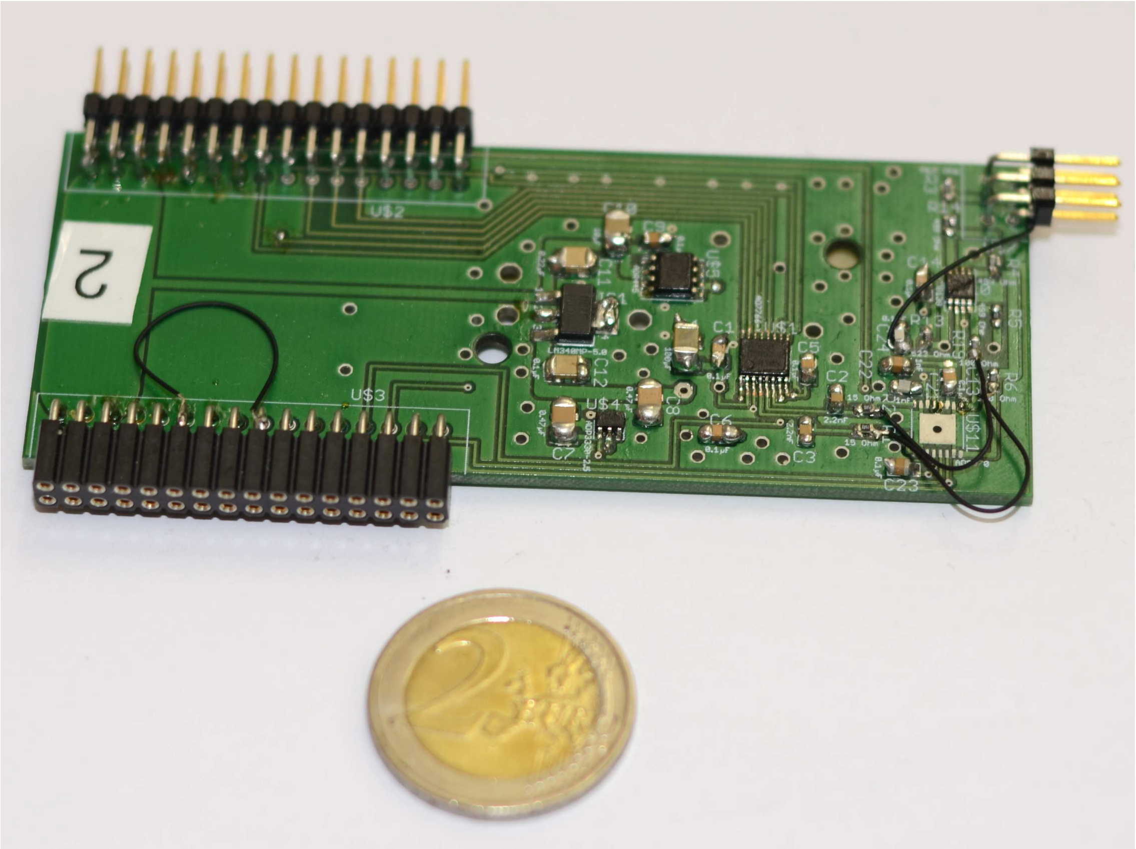

Figure 1.27: Picture of the ADC module in version 2 without the variable gain amplifier (lower right corner of the module). Two mod-wires bridge the missing VGA IC. The third mod-wire allows to inject a different ADC's reference voltage for test purposes.

Connector board

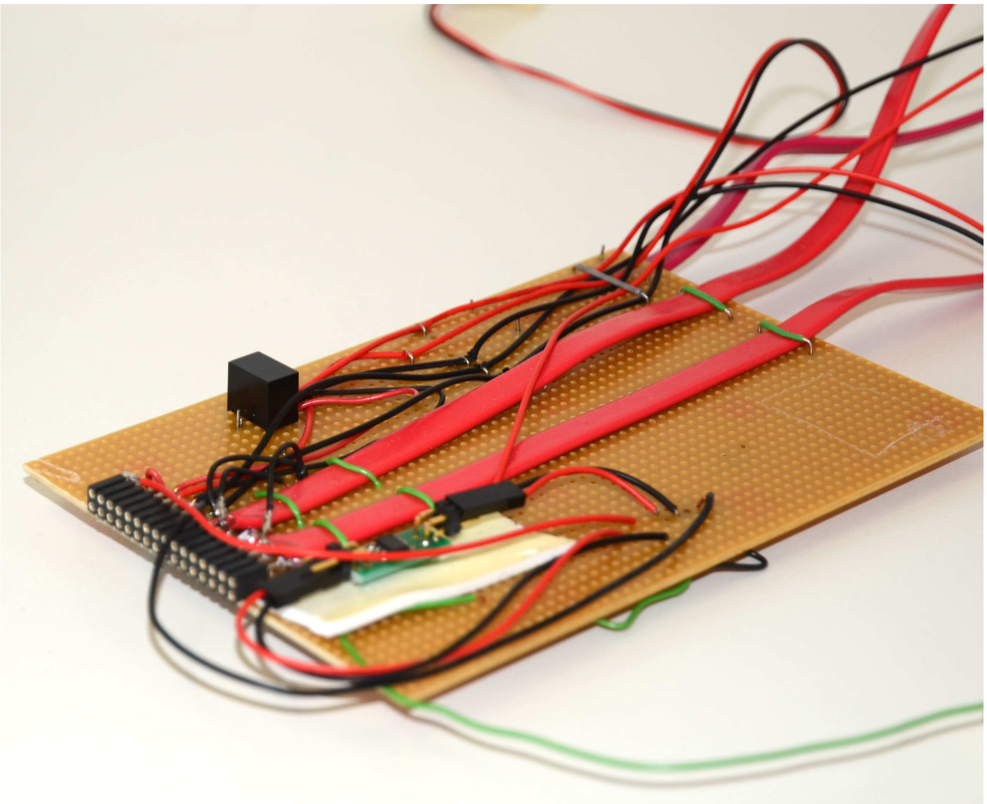

Figure 1.28: Picture of the prototyped connector board for the ADC modules version two. The SATA plugs are connected to the FPGA connector boards. The small green PCB can be exchanged and carries an additional reference voltage source for test purposes.

For performing the necessary experiments with the ADC modules, it was important to have a reliable adapter between the FPGA ZestET1 board and the ADC module stack. Thus I rapid-prototyped such a connector board (see figure 1.28). The first main task of the connector is to transfer the digital signal lines from the pin header of the module stack into SATA cables which can be plugged into the connector board of the FPGA (e.g. see figure 1.3). The second main task is to convert the 10V DC power rail into a 5.0V supply voltage for the FPGA board. This is very important because the FPGA board is not 10V tolerant. In the case that the FPGA's digital signal lines are supplied with 10V, then the FPGA gets a hunch, generates a bang, and the ICs on the ADC modules release their 'magic' smoke. I observed this behaviour once when my oscilloscope tip slipped. However, the ADC modules require the 10V supply rail for operating their reference voltage sources. For testing the behaviour of the ADC IC under different reference voltages, I included an extra exchangeable reference voltage source on the connector board.

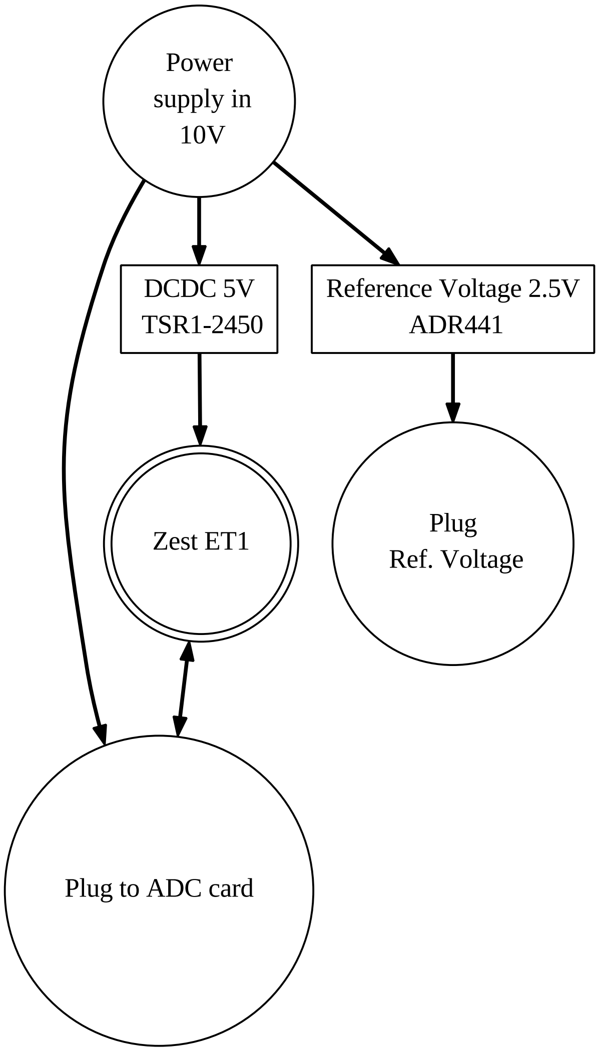

Figure 1.29: Structural overview of the connector board (see figure 1.28). One main 10V DC rail is used to power the whole setup. While the ADC modules have their own power management, it is necessary to generate 5.0V for the FPGA board from 10V rail. In addition, the connector board houses an extra reference voltage supply for test purposes.

1.5.3 Version 3

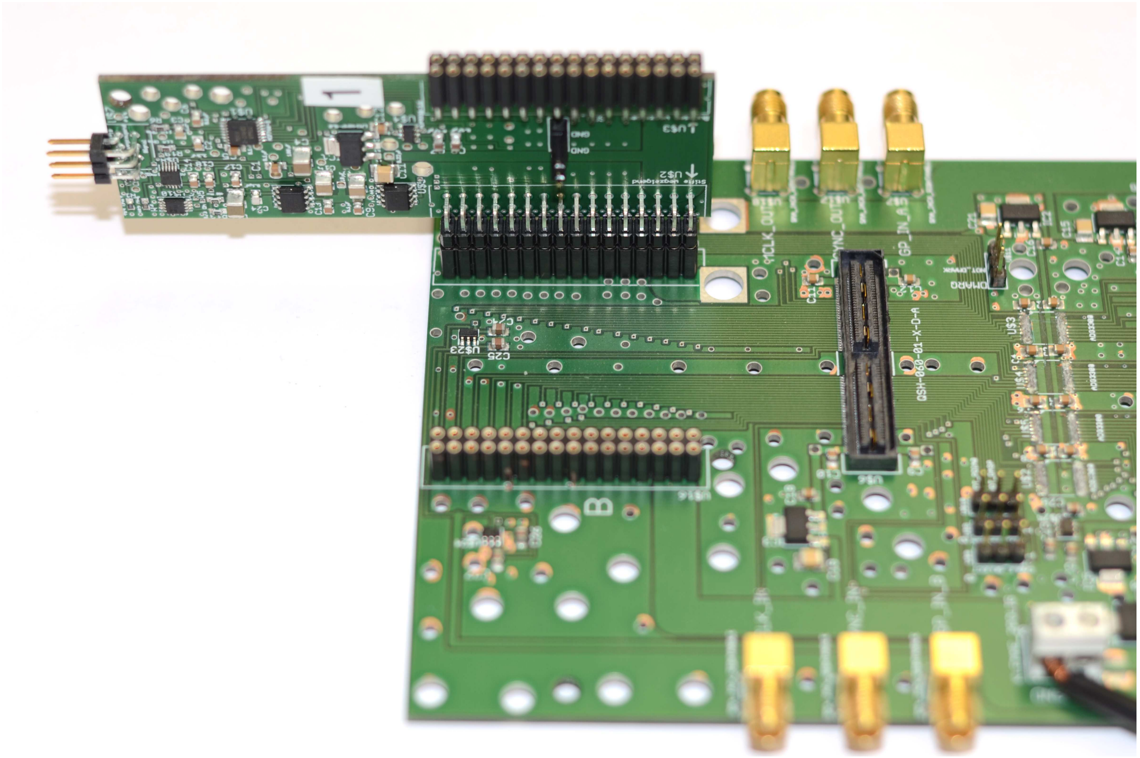

Figure 1.30: Third version of the ADC channels. One channel card on the corresponding connector board.

The second version of the ADC channel showed us that the results when using such a fully differential digitally programmable variable gain amplifier (VGA) are extremely unsatisfactory. Furthermore, options for other suitable versions of these kind of amplifiers are (still) not existing (e.g. they typically don't work at these low frequencies). Thus the development of the ADC modules ran into a dead end. Luckily, Mario Kaiser from Brain Products mentioned that digitally programmable are normally not used because they are typically too expensive. Instead digital potentiometers / resistors with simple amplifiers are often used. And this allowed the development of version 3 (see figure 1.30).

Version three was shortly tested with an external head-stage amplifier within an animal experiment (results are not shown). The system worked but it drew too much current from the head-stage amplifier such that it's voltage output broke down. The experimentalists found hints in the manual of these amplifiers which suggest that devices need at their output a high impedance. Version three of the ADC channels was designed to have a 50 Ω impedance, which goes into the opposite direction. In version four we tried to fix this problem as well as tried to further improve the signal-to-noise ratio of the analogue front-end.

ADC channel

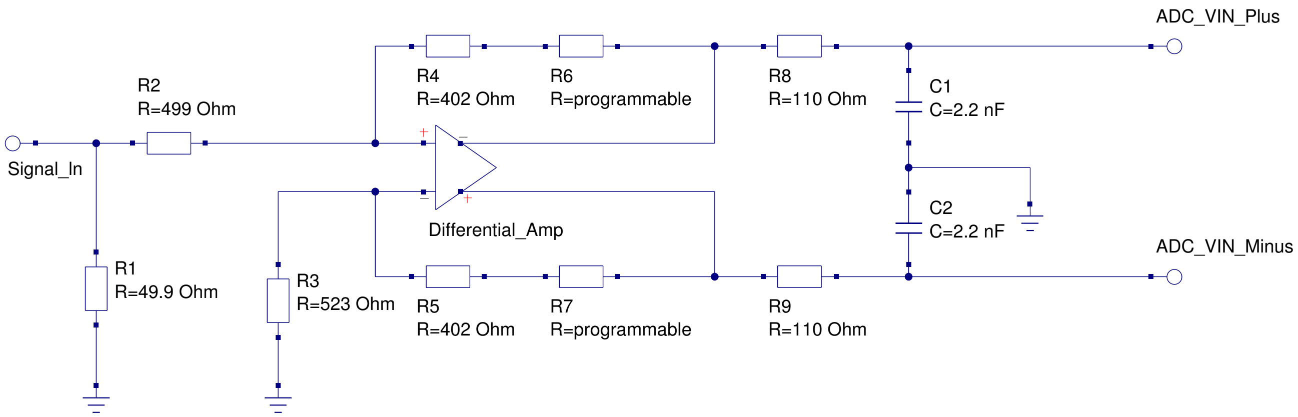

Figure 1.31: Analogue front-end of the recording channel. The circuit is strongly inspired by the manual of the differential amplifier.

Based on this digital potentiometer-hint, I re-examined the circuit diagram of ADC channel version two. The tests with version two had shown that the system worked fine without the VGA. Exchanging the resistors of the driver's amplifiers feedback loop also showed that the system works well even with high amplification factors. The simple conclusion was to add digitally programmable resistors (AD5162-10kΩ , a SPI controllable digital dual potentiometer with 256 setting on a linear scale from Analog Devices) into the feedback loop of the driver's amplifier (see figure 1.32 for the new structure of the ADC module and figure 1.31 for the diagram of the analogue front-end). The chip select lines of the VGAs were re-used as chip select lines for the digital resistors. I also optimized the ground planes further and was able to shrink the width of the module. The resulting design (see figure 1.33) was manufactured and assembled by PCB-Pool.

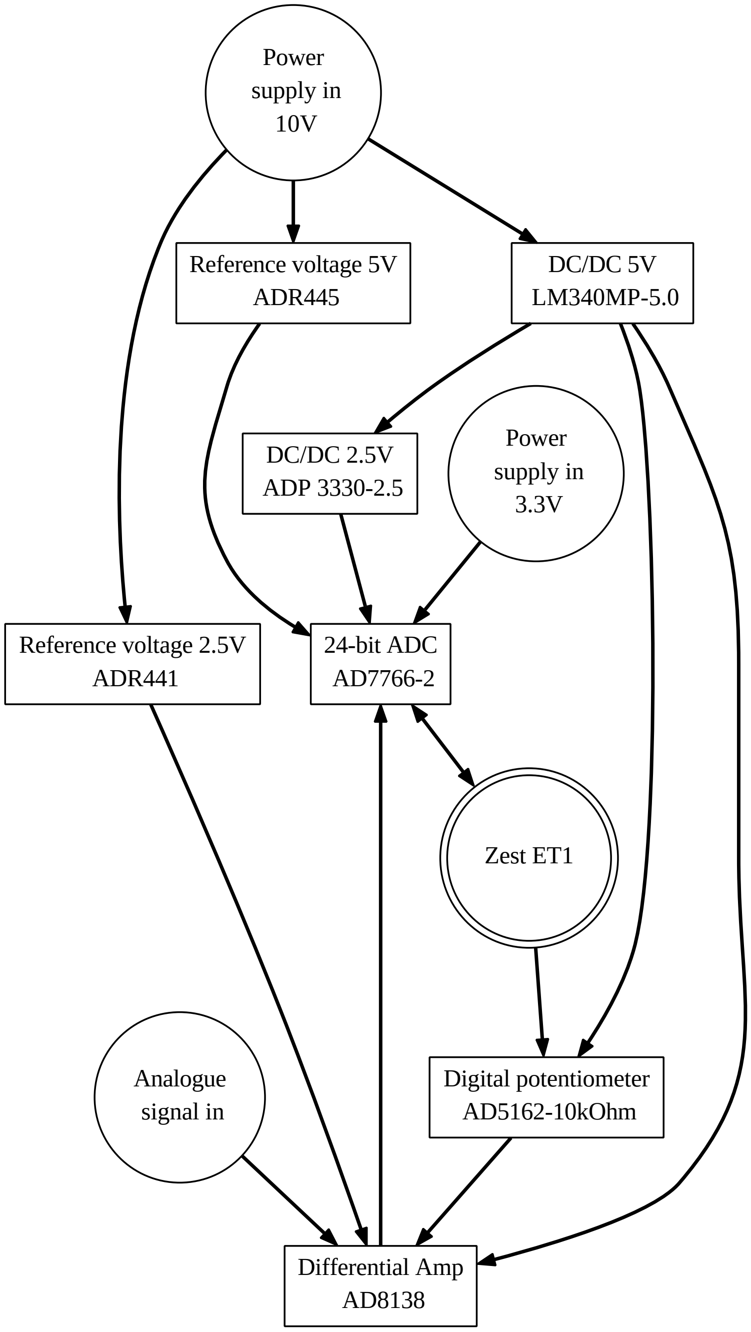

Figure 1.32: Structure of one recording channel module in version three (see figure 1.33).

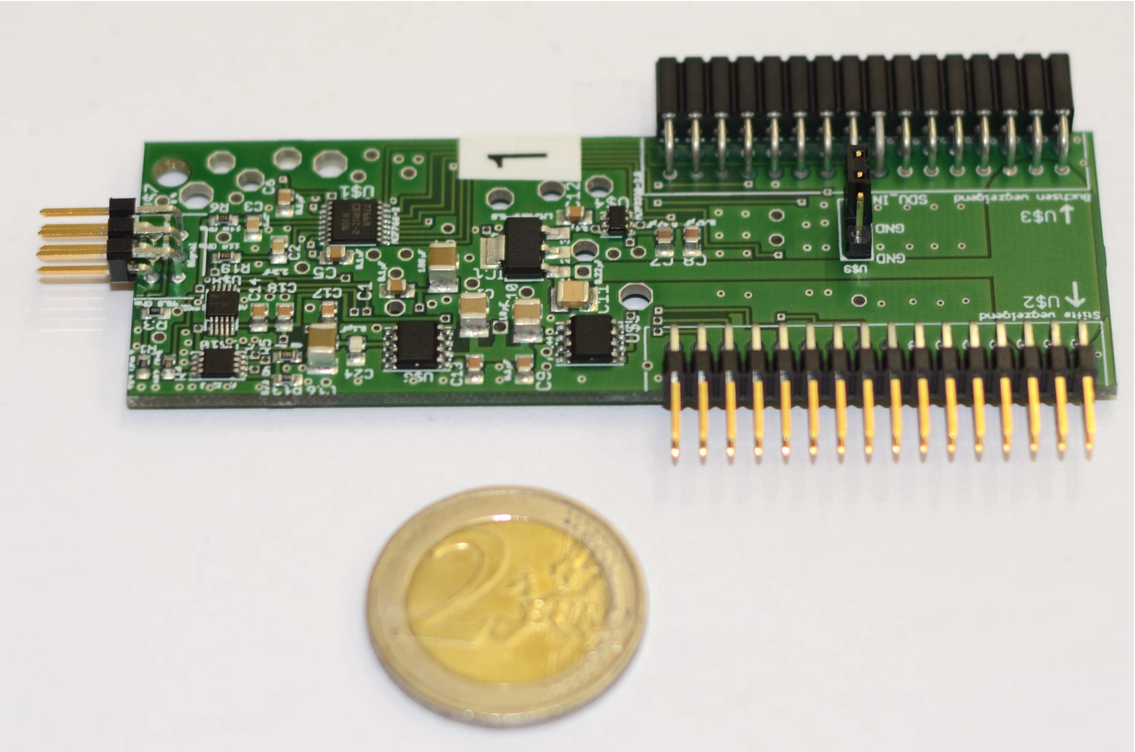

Figure 1.33: Single recording channel in version three. 16 of these channels can be stacked and plugged into one slot of the connector board (see figure 1.34). These type of channels have a programmable amplifier based on digitally programmable resistors allowing to control the amplification factor dynamically.

Connector board

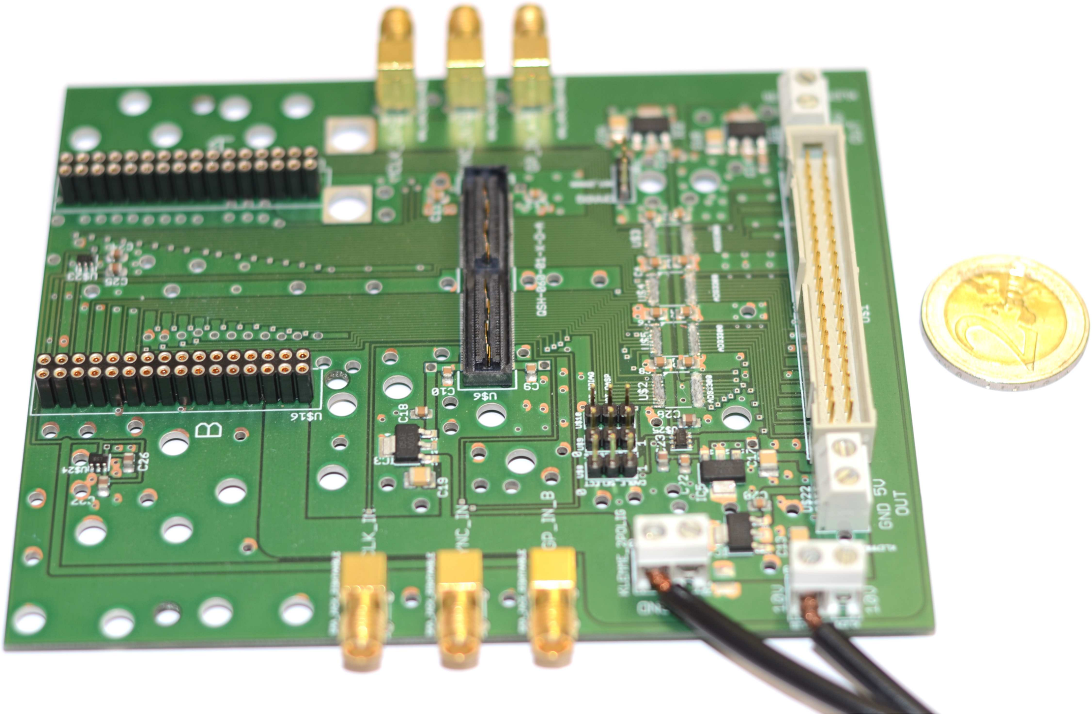

Figure 1.34: Professional realisation of the connector board for ADC channels in version three. The board can host up to 32 channels in two 16 module stacks.

I expected intensive tests with the ADC modules in version three. Thus a robust realisation and a not rapid prototyped version of connector board with flying cables was necessary. Instead of the SATA cables which are used for the rest of the base station, I added a Samtec connector plug for the FPGA board to be directly connected to the connector board.

In most respects, the concept of the new connector board (see figure 1.35) is very similar to its old version (see figure 1.29). The main changes are that I replaced the 5V DC/DC converter by another model, added 3.3V DC/DC converters for unburden the DC/DC converters on the Zest ET1 board, and I added an extra pin header plug for another set of 16 channels. Especially due to the difficult Samtec connector plug, the board was assembled by PCB-Pool. See figure 1.34 for the result.

This version of the connector board had another new feature which didn't worked out as expected. The idea was to cache all the recorded channels onto an IDE (Integrated Drive Electronics) hard-drive. Such a feature is required if scalability in number of channels is taken seriously. One ADC channel produces around 25k samples per second with 24 bit per sample. We have to assume that the samples are converted into 32 bit values before they are send to the external PC because this takes significant computational burdens from that computer. This results in approximately 100kByte per

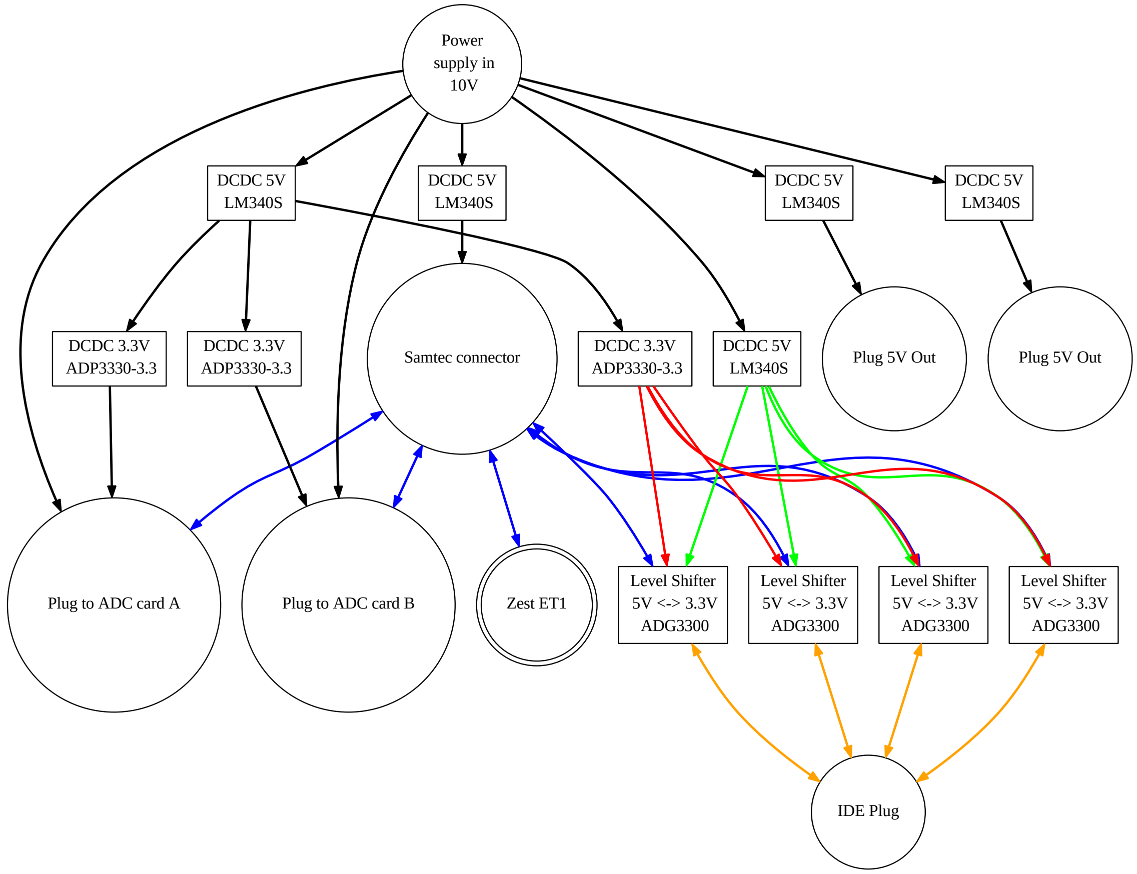

Figure 1.35: Structure of the new connector board. The design is based on version two's connector board (see figure 1.28 and 1.29). A new feature is the (non working) IDE interface for caching the recorded samples.

second of continuous data stream per channel. Looking into a typical electro-physiology lab like at the University of Bremen, we see that several hundreds of individual electrodes are already the de facto standard. Reaching one thousand electrodes can be expected soon which results in 100MByte per second. However, the system design was aimed as long term platform. As result the concept needs to hold for electrode number significantly beyond 1000 electrodes. During a running experiment this amount of data is produced continuously. An interruption of the continuous data transfer from the recording system to the external PC can be disastrous. One pause created by the multi-task nature of the operation system on the computer system in the size of parts of one second would cause a buffer overrun (leq 64 MByte) on the recording system and result in data loss.

The conclusion is that a continuous data stream from the recording system to the PC needs to be avoided for larger number of electrodes. Thus we need a storage medium that can hold the collected data and which allows to recover the data AFTER the experiment was done from the recording system. However, the recording system needs

the additional means to stream selected ADC channels in real-time onto the PC for monitoring the neuronal activity during setting up the electrodes and the experiments.

A simple calculation shows that we can expect in 10 hours (36000 seconds) of experiments around 2.4GByte of 24 bit samples. If we assume mobs of 16 channels, we need storage devices with at least 38.4GByte. Putative options are USB sticks, USB hard-drives, SD cards, SATA hard-drives, and IDE (=PATA) hard-drives.

USB: Regarding the USB devices, it is necessary to include an USB 2.0 (or newer) High-Speed host controller (e.g. from the company Cypress) to the connector board. It is problematic to get suitable USB host ICs for non-industrial-scale projects. Furthermore, it requires 480MBit per second high frequency connections between the USB host IC and the USB plug. And finally, the data and protocol handling of a typical industrial USB host controller IC is not end-user friendly.

SD-Card: In that stage of development, I dismissed the use of SD cards too. First of all, the size of a typical SD card was at that time at 32GByte. Some very expensive version with 64GByte were available. Furthermore, the data transfer protocol for these cards is closed source. At that time, I didn't knew that the SD cards have a SPI-based low speed fall-back protocol, which should be fast enough for this application and nowadays SDXC cards with 128GByte are available. However, there are leaks of the SD card full-speed protocol available on the internet as well as the website www.opencores.org provides the corresponding modules for FPGAs. It is not clear if these sources should be used for an open source project. In summary, this option is much more suitable than I thought at that time.

SATA: SATA (Serial Advanced Technology Attachment) uses four signal lines for exchanging data between two devices. Due to its serial nature, the signals have a data rates geq 1.5GBit per seconds. This speed requires a high frequency design of all the involved PBC lines, which is beyond the scope of this project. To make it even worse multi-gigabit transceiver with clock recovery circuits are required. Some new types of FPGAs provide this kind of transceivers. Furthermore, the SATA protocol and its 8b/10b encoding for the clock recovery makes the use for a non-industrial user nearly impossible. In addition, the documentation of the standard is closed source (but leaked to the internet). In summary, the direct use of SATA is not possible. One possibility is to use a SATA-to-PATA converter IC. However, these IC are normally designed for connecting PATA devices to a SATA controller. An intensive investigation showed that there seems to be one(!) IC that works in the required opposite direction but it looks like that it is not available for non-industrial users. Another possibility is to use a chip-set IC for modern computers (e.g. a south-bridge IC from Intel). However, the complexity of the documentation is much too high for the scope of this project. Taken all this facts into account, we have to dismiss SATA as an option.

PATA (formerly called IDE): PATA (Parallel Advanced Technology Attachment) is the predecessor technology of SATA and is in use for over an decade. PATA hard-drives are cheaply available, are still in production and hopefully purchasable for many years. On

the internet, tutorial from hobbyists are available and explain how to use PATA drives for your own designs (e.g. http : //www.pjrc.com/tech/8051/ide/wesley.html and http : //www.mikrocontroller.net/articles/Festplatte (in German)). Since the drives are cheap, in large quantities available for tests, have large storage capacities, are fast enough, and are relative easy to use in an own design, I decided to use them for the ADC connector board. However, it is not possible to connect a PATA drive directly to the base station FPGA. The drive uses 5V logic level signals and the FPGA 3.3V logic level signal. Furthermore, the FPGA is not 5V tolerant and would be destroyed by signals from the PATA drive. Thus it is necessary to use a so called level shifter for making both devices compatible.

I decided to use four 8-channel bi-directional logic level translators from Analog Devices of the type ADG3300 on the connector board (see figure 1.35) for interfacing the PATA drive. The first test of these level translators showed severe problems. The eight channels' logic states couldn't be switched reliable by the FPGA and also some highly non-understandable oscillations of the level translator's output occurred during the test measurements. During debugging, I found a note in the ADG3300 manual that explains that these translator IC need to be driven by a signal with a minimum of 36mA current. The FGPA on the base board is not even able to produce half of this current with its output pins. The default setting for that FPGA is 8mA. I found an alternative in the Texas Instruments TO TBX0108 8-channel bi-directional voltage level translator. This IC has the same footprint as the ADG3300 and needs, according the manual, only 2mA current. I designed corresponding test PCBs which were produced. I also started to assemble the test module but ran out of time before I could finish that step. In retrospective, a 5V Xilinx CLPD may have also done this job. However, I decided to use SD cards for new designs in the future.

Test measurements

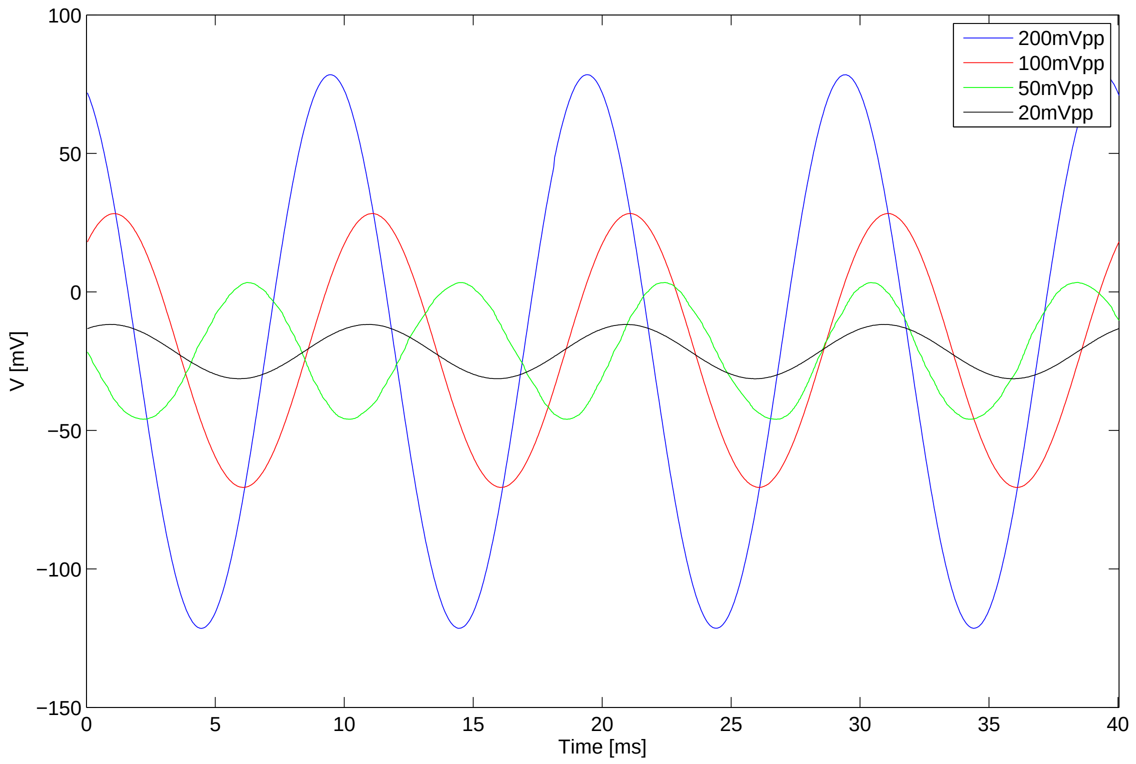

Figure 1.36: Recording of sinusoidal waves with 100 Hz and large amplitudes (from 200mV peak-to-peak down to 20mV peak-to-peak). The amplification factor of the channel is set to its minimum.

As advertised earlier, I performed some very simple test measurements with the ADC channels. The main goal was to check that everything functionally works and to get a first impression about the signal-to-noise ratio of the recorded signals. For that, I connected the waveform generator's output to a non-shielded approximately 1m long cable (with two cooper leads and a BNC plug on the generators side). On the other side of the cable, its two clamps where connected to a so called 2-pin jumper cable with approximately 1cm leads. The jumper cable was plugged onto the pin header of the ADC channel's input. For power supply, I used a Peak Tech 6080 DC power supply to generate the necessary 10V for operating the whole system.

In the first run I recorded sinusoidal waves with 100Hz and rather large amplitudes. Figure 1.36 shows the result of these measurements. During the data acquisition, the amplification was programmed to its minimum. For calculating the y-axis, I used the 200mV peak-to-peak values and calibrated the scale of that axis with it. I used this calibration for all the figures shown in this section. The recorded curves look clean. A check with an oscilloscope revealed that the offset seems to be a result of the recording system (maybe due to mismatches by production tolerances of the used resistors in the feedback loops of the fully differential driver) and not the function generator.

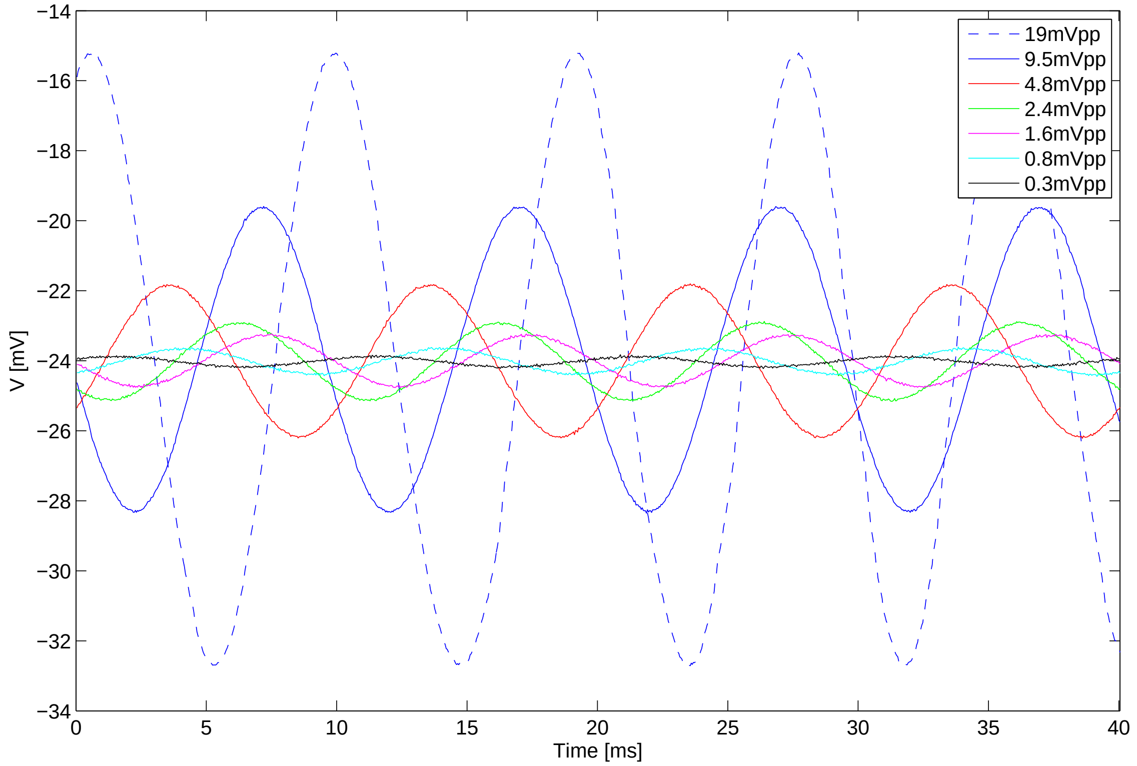

Figure 1.37: Recording of sinusoidal waves with 100 Hz and small amplitudes. Three damping elements (-6dB, -10dB and -20dB) were used. The amplification factor of the channel is set to its minimum.

Since 20mV peak-to-peak (pp) is the minimal amplitude of the used waveform generator and it is required that the ADC channel operate in μV range, it was necessary to reduce the amplitude further. An attenuation was introduced by three BNC damping elements with -6dB, -10dB, and -20dB between the waveform generators output and used the cable. Together the signal was reduced by -36dB, which corresponds to a reduction of 1/63.1 of it's amplitude. This results in a 317 μV peak-to-peak (= 158.5 μV amplitude) sine waveforms.

In figure 1.37 the results with the reduced amplitudes are shown. The largest one is 19mVpp (1200mV peak-to-peak on the waveform generator) and is shown as blue dashed line for reference. For the smaller amplitudes, the curves show still a fair signal-to-noise ratio. In figure 1.38 the curve with the smallest amplitude (black curve) is shown alone for better visibility. Close inspection of this curve reveals strange random spikes on top of the signal which seem to be bigger than the 'normal' noise level. We found that when the sample rate is increased from 25k samples per second to 32k samples per second (the maximum of the ADC IC) then the problem is much more severe. Furthermore, we found that this problem can be available on one day and gone for another day or time. Thus our guess is that this problem is due to an external electro-magnetic pollution.

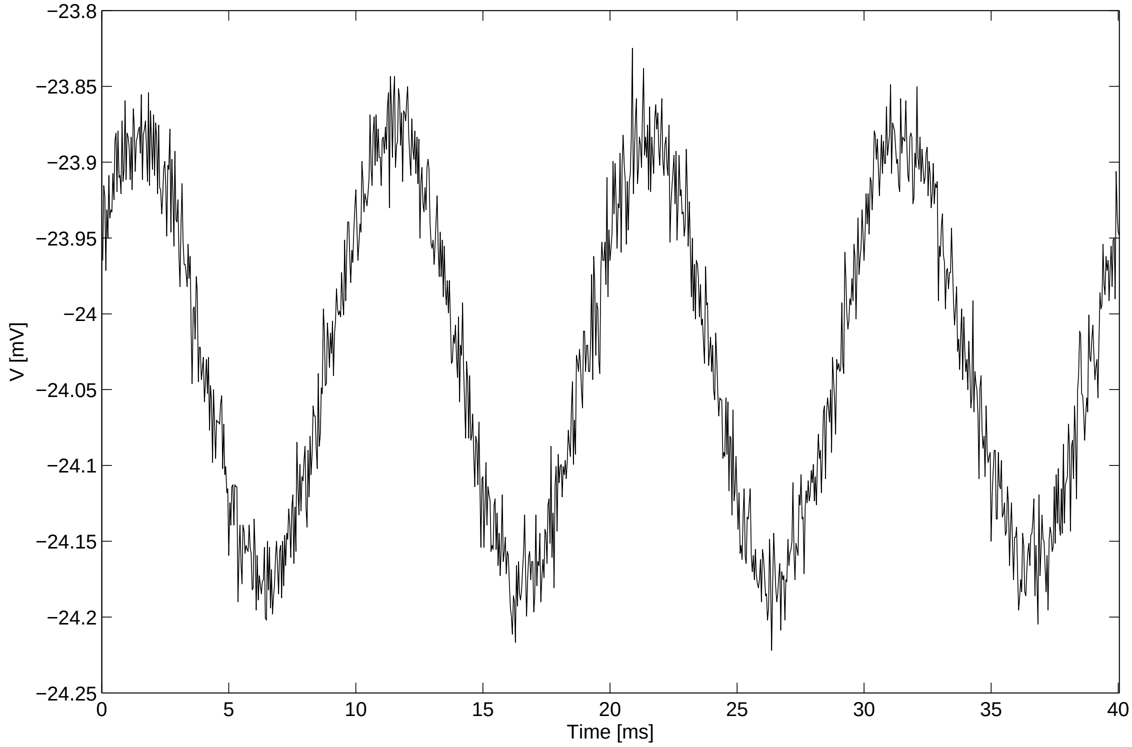

Figure 1.38: Recording of a sinusoidal wave with 100 Hz and the smallest possible amplitudes (317 μV peak-to-peak). The amplification factor of the channel is set to its minimum.

All the three previously shown figures were recorded with the amplification factor programmed to its minimum. In figure 1.39 (The units of the y-axis are now 'arbitrary'. I used the same calibration as before but for larger amplifications this doesn't correspond to mV any more.) I varied the amplification setting from its minimum to its maximum. The signal-to-noise ratio visually looks rather constant. However, the number of used elements ($= \mathsf{abs}(\mathsf{max}(\mathsf{Waveform}(t)) - \mathsf{min}(\mathsf{Waveform}(t))))$ of the 24-bit sample differ as expected. We find for the different amplification settings (AS): 298(AS=0), 1452 (AS=50), 2508 (AS=100), 3674 (AS=150), 4606(AS=200), and 5825 (AS=255).

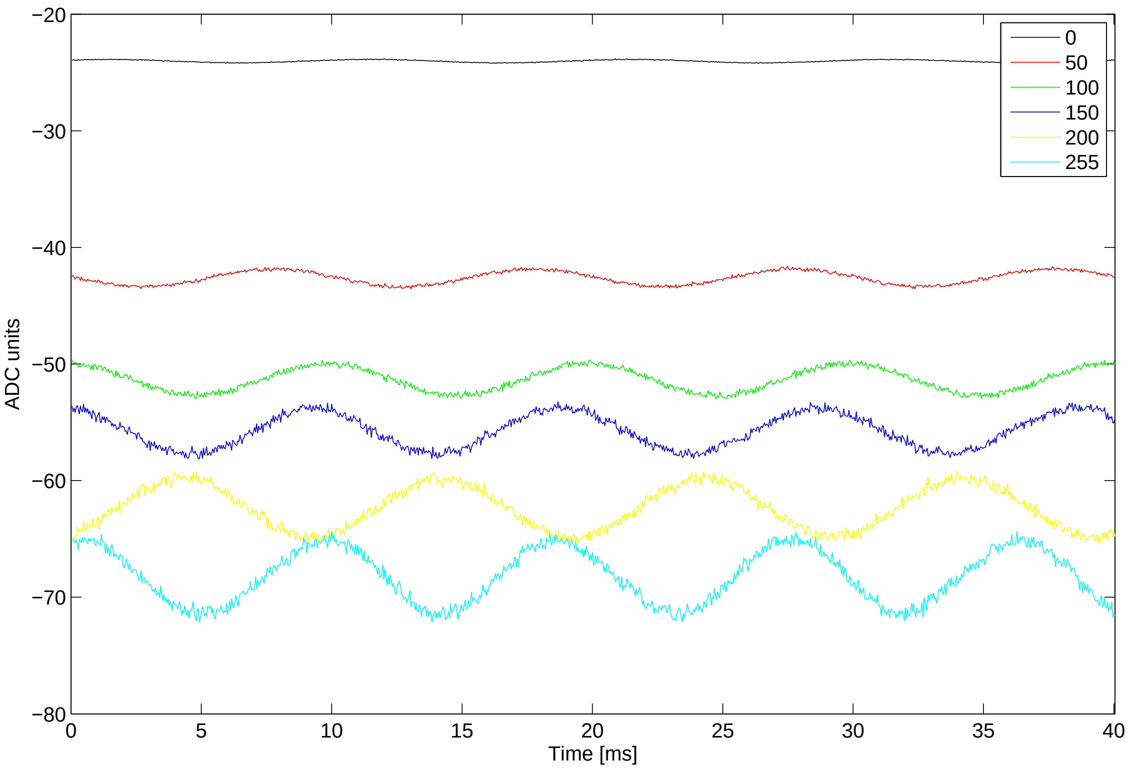

Figure 1.39: Effect of the programmable amplifier. Recording sinusoidal waves with 100 Hz and the smallest possible amplitudes (317 μV peak-to-peak). The black line is the waveform acquired with the smallest amplification (see figure 1.38 for a larger version). The cyan one was recorded with the highest amplification.

1.5.4 Version 4

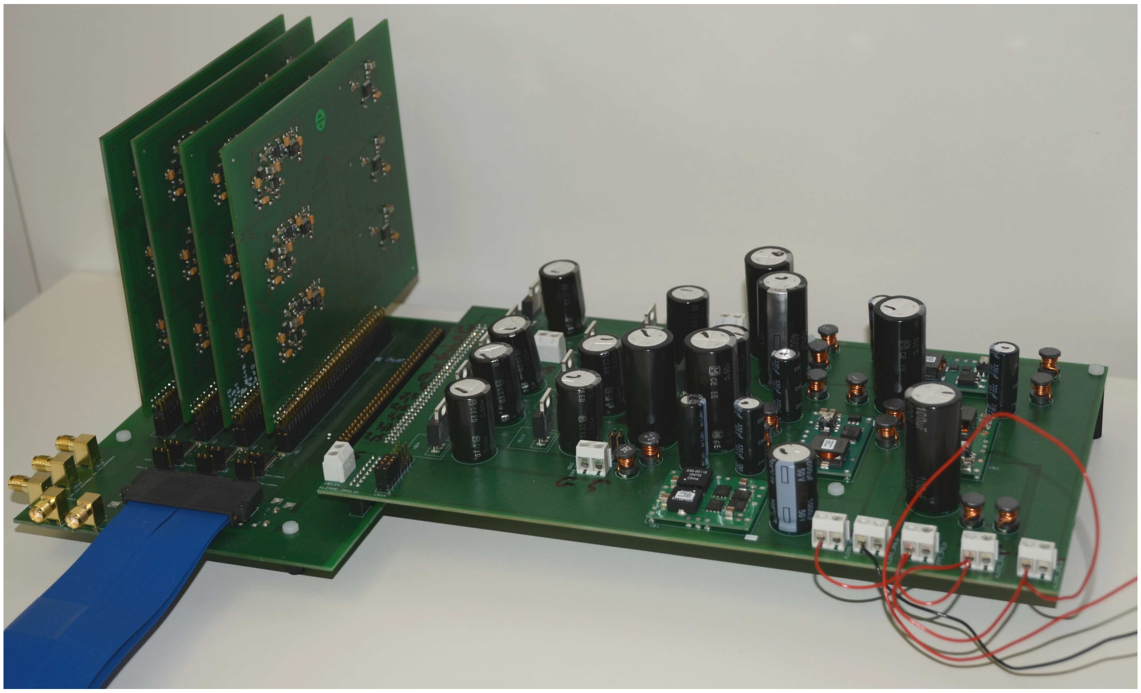

Figure 1.40: Version 4 of the electro-physiological recording sub-system.

The results of version 3 were very promising but the 50Ω input impedance drained to much current from the head-stage amplifier, which is a pre-amplifier located very close to the electrodes. Thus it was necessary to re-design the analogue front-end of the ADC channels. See figure 1.40 for the whole version 4 system.

Together with Andreas Kreiter und Dieter Gauck, I planned the low-, high-, and band-pass filters for version 4 using the Texas Instrument tool TINA 9. This made it necessary to replace the fully differential driver from Analog Devices with a similar component from Texas Instrument, for which a simulation model was available. We also added other TI amplifiers to the analogue signal path. One very low-noise pre-amplifier and two programmable amplifiers. We optimized the signal-to-noise ratio (e.g. how much amplification at which amplifier stage, values of the resistors in the feedback loop as well the component selection) and the filter banks (e.g. cut-off frequencies and phase properties) based on the TINA simulation. Based on an intuition from Andreas Kreiter, two version of card were designed: One simple version without the programmable amplifier stages and a second version with them. The simple version are working fine. The other version has severe problems. For adjusting the two amplifier stages, we used the same digitally programmable resistors as in version 3 but here this resulted in a non-working system. After an intensive investigation, Andreas Kreiter and myself found the reason for the failure in this digital resistors. These components are only able to conduct voltages between ground and its supply voltage. The direction doesn't

matter but negative voltages relative to ground are not allowed. In version 3 these resistors 'see' only positive voltages due to the fully differential nature of the driver. In the traditional setting with a normal operational amplifier as in version 4, negative voltages occur regularly if the analogue signal (e.g. sine wave input without offset) what results in very strange behaviours (non-linear resistor in an amplifier feed-back loop). After forcefully removing the programmable resistors from the card and replacing them with normal resistors, the ADC channel worked but wasn't able to meet the expectation from the simulation. Thus the development of a version 5 is necessary.

All these different ICs created the need for a clean and highly stable power supply with many different power rails. Furthermore, we wanted to test the daisy chaining of the 24 bit-ADCs and their interaction in more detail. Thus we decided to put 6 ADC channels on one card module. As result a new connector board was required. The new connector board is designed for four cards with 6 channels each. I also tested the use of buffered shift registers, as signal distribution system for programming the resistors, for saving many FPGA pins.

I also designed a FPGA board with over 130 user IO pins, two SD card slots, a complex power management, flash data storage for the firmware and an USB interface to an external PC. Due to cost saving measures and risk management, we didn't produced the system although the design was finished. Instead I changed the connector board for connecting it to the Zest ET1 board.

Power supply

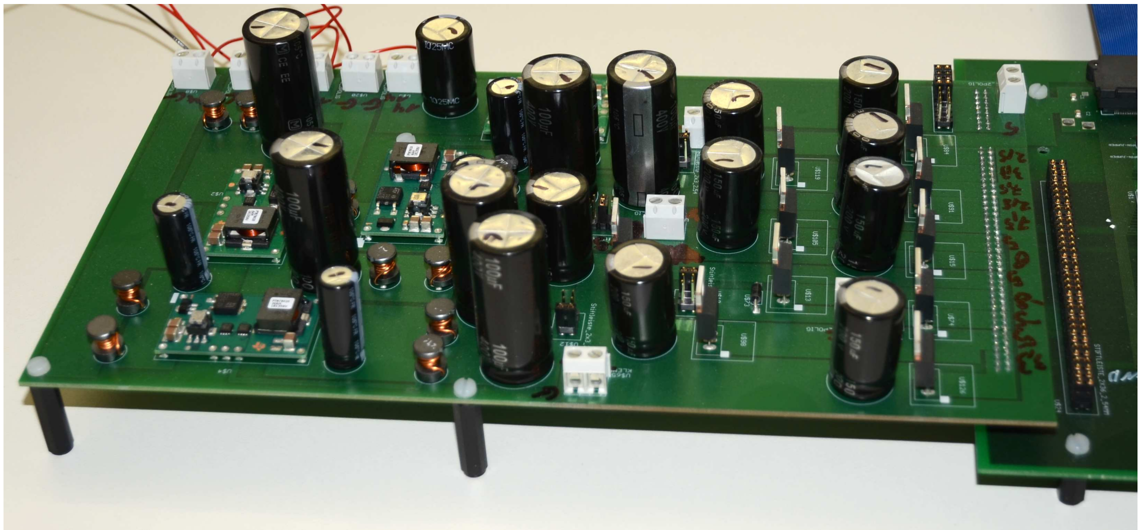

Figure 1.41: Power board for the ADC recording system. The board delivers 13 individual power rails for different voltages and different purposes. The idea was to generate as clean as possible supply voltages for the operational amplifiers and the ADCs.

Recording small voltages requires a system of amplifiers and analogue-digital-converters,

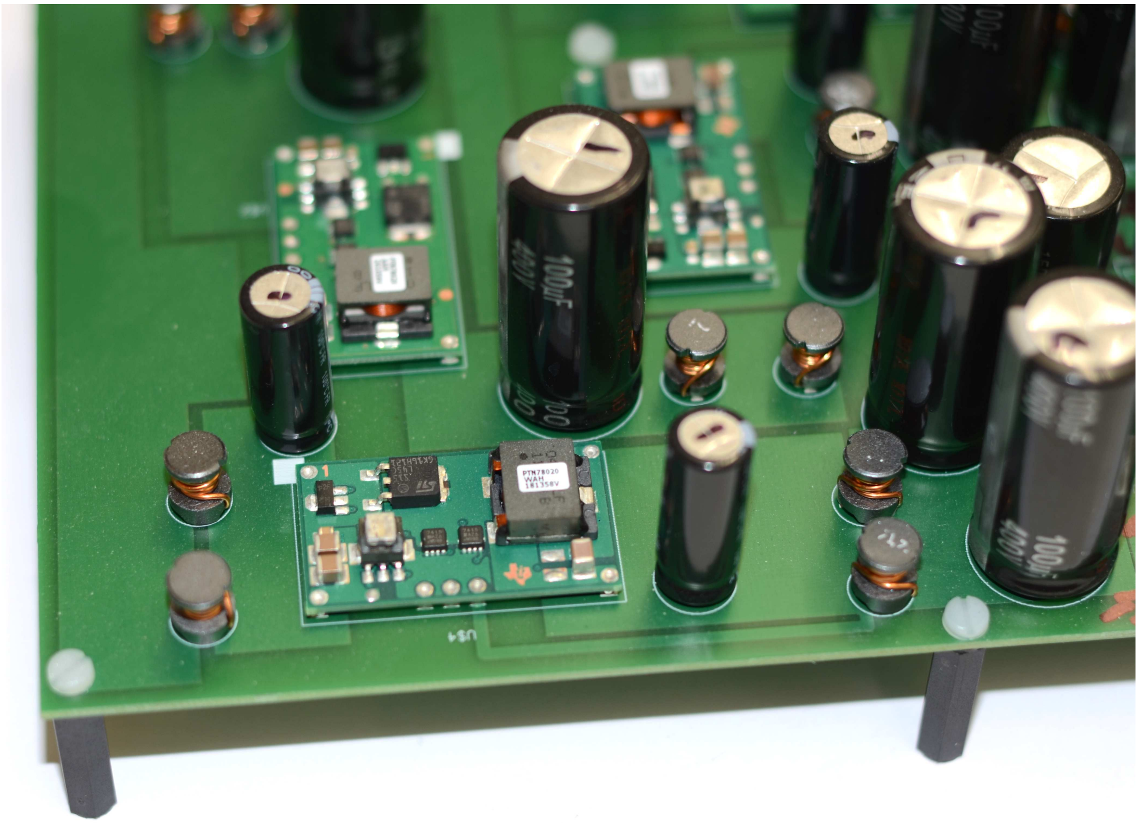

Figure 1.42: Close up of the installed power modules.

that are able to process the signals reliably and reproducibly over time. One requirement for that is a stable and clean power supply for operating the equipment. The envisioned design required many different voltages. Furthermore, is it important to protect the analogue components from high-frequency pollution produced by the digital components. This power board was designed to drive at least 128 electrodes and one (big) FPGA.

In the end, 13 individual power rails are required. The power rail with the most current is used for the FPGA board, for controlling the other components. A modern FPGA requires many different supply voltages. For keeping this power board universal, it provides 5V with up to 25A for the FPGA board. It not possible to reliably estimate the power requirements for a modern FPGA with high precision without the finished firmware. The requirement depends e.g. on the used resources, the voltages of the IO banks and all the used clock frequencies. The FPGA board itself should contain DC/DC converters for generating the required voltages (which are $\leq$ 5V), which will depend on the specific type of the FPGA.

The ADC IC requires separate +2.5V for analogue and digital parts as well as +3.3V for its digital IO banks. Furthermore, the ADC require a very precise reference voltage of +5.0V. The reference voltage source should be located near the ADC and requires

itself a clean supply voltage of +7.5V.

For the analogue signal pathway two normal operational amplifier (OpAmp) ICs and one differential OpAmp are used. The first OpAmp is driven with +/- 7.7V, allowing much room for amplifying signals with DC offsets. The second OpAmp IC is only operated by +/- 5V. The fully differential OpAmp converts the preprocessed analogue single-ended signal into fully differential signals before it is digitized by the ADC. This special OpAmp is driven by +5V and requires its own reference voltage of +2.5V, which is provided by a second precise reference voltage source using another +7.5V power rail.

Finally, there is a +5V power rail for driving the digital potentiometers/ resistors and a +3.3V one for the buffered shift registers. This power supply board joins the ground connections of all these supply voltages and thus allows a star-shapes distribution of ground to all the connected energy consumers. This is necessary for a clean measurement because it keeps the digital and analogue ground planes as long as possible separate.

In figure 1.43 the structure of the board's design is shown. The power board assumes an external 14V DC power supply. The first conversion layer consists out of non-isolating adjustable power modules. One module type is for positive voltages (+5V) with up to 26A mainly for supplying the FPGA board, one type is for negative voltages (-11.5V with 2A) for the OpAmps and the last type is for positive supply voltages (+11.5V and +7.5V) with up to 6A. Before the input and after the output of the modules, it is suggested by their manuals (except the module for the FPGA) to install PI filter for removing non-DC components from the power rails. This is necessary because these power modules are switching power supplies which run with around 550kHz switching frequencies. Without these filters the DC voltage would be strongly contaminated by these frequencies.

For improving the ripple rejection, the manual of the low drop-out regulators (LDOs) suggest to add an additional capacitor between the adjust and the voltage out pin. I also added these capacitors to the design. The normal ripple rejection of these LDOs lies in the region of -75dB.

The question is: How good is the quality of the supply voltage produced by the power board? The answer is: I don't know it. We don't have the necessary equipment at hand for testing it in all its details. Figure 1.44 shows some measurements that give a feeling of the quality. However, during tests we found that the oscilloscope's probe and it's cables (1x probe Hameg HZ 154 on an Agilent DSO6102A oscilloscope) work as an antenna which collects emissions of the switching power modules very efficiently. Thus we can not say if the $\approx$ 280kHz (half of the switching frequencies of the power modules) component measured on top of the DC voltages is really there or a result of this electro-magnetic induction into the measurement equipment. Just connecting ground of the oscilloscope and the power board's ground as well as connecting the probe's tip of the oscilloscope with the power boards ground, allows to detect the $\approx$

280kHz component with an amplitude reading up to 100mV (the measurement is very strongly depending on the orientation, position, and distance of the measurement tip).

The used external power supply (PeakTech 6080 DC) produced without load a clean (in comparison to typical switching power supplies) 14V supply power with only +/- 6mV noise. When connected to the power board, the power modules drain power with (different) switching frequencies around 550kHz, creating complex oscillations on the output of the external power supply. It is very important that the connections between the external power supply and the power board never lie over or in the proximity of the switching power modules. If they do then this can cause very strong feedback effects that can be even found in the recorded signal.

At the +11.5V output of the power module (after its PI filter), we find an oscillating voltage component (with $\approx$ 280kHz and an amplitude of 35mV) as well as some noise ($\approx$ +/- 2mV) on top of the DC voltage. After the LDO (+8V power rail) the oscillation on top of the DC voltage has still an amplitude of $\approx$ 25mV and the noise stayed similar to the earlier stage. It is important to note, that an unknown part this noise is produced by the measurement setup (e.g. cables, oscilloscope and probe).

The power supply ripple rejection (PSRR) of the most sensitive component (the first OpAmp), is $\approx$ -100dB ($10^{-}5$) at 280kHz. In the worst case we would expect a perturbation of 0.25μV. However, at all of the vital components, the power supply is stabilized with ferrite beads as well as 0.47μF, 2.2μF ceramic capacitors and 10μF tantalum capacitors. In addition to that, the rest of the analogue pathway as well as the ADC itself have low-pass filter that also remove these high frequencies.

In the case that it turns out that these power rails are not clean enough, it is easily possible to reduce the amplitudes of these 280kHz oscillations with additional PI filters. Furthermore, it is recommended to install heat sinks for the LDOs. For this reason, the required space was left empty around the components on the board.

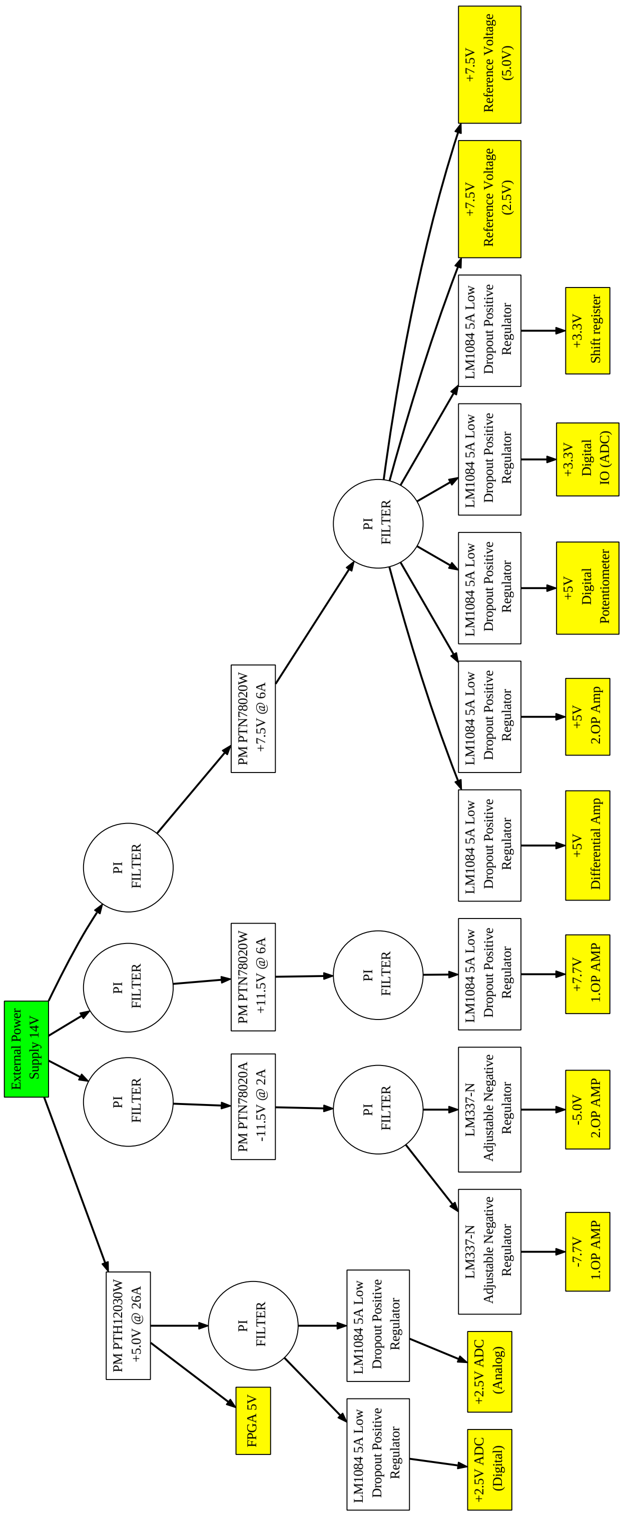

Figure 1.43: Structural overview of the power supply board (see figure 1.41). Green represents the incoming 14V DC supply voltage. Yellow marks the individual outgoing power rails.

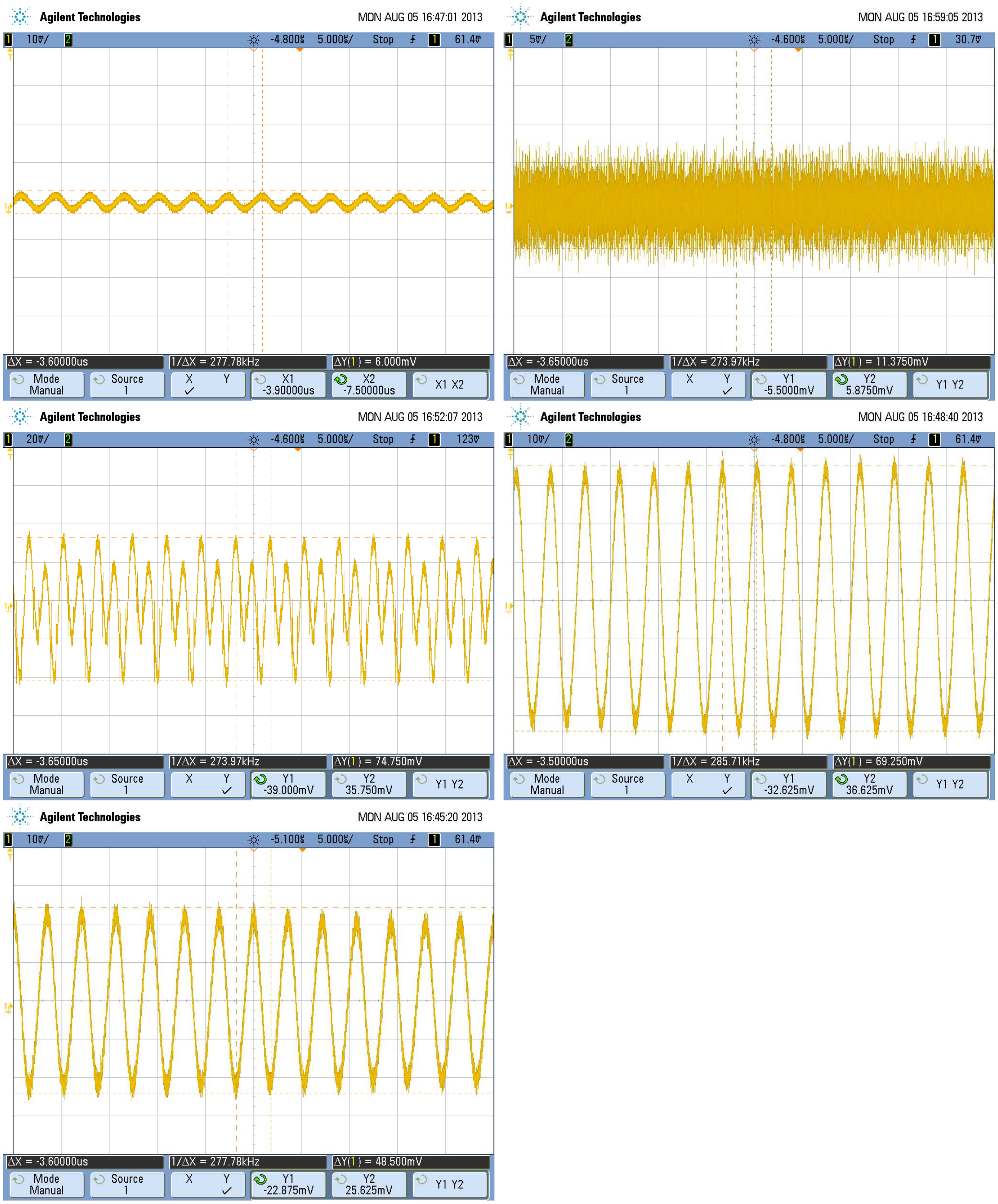

Figure 1.44: Upper row, left: oscilloscope probe connected to ground of the power board while the unconnected tip of the probe is lying 2cm away from the pin header of the board. Upper row, right: measurement of the output of the AC/DC power supply at 14V output voltage and no load. Middle row, left: measurement of the output of the AC/DC power supply at 14V with connected power board. Middle row, right: output of the +11.5V power module after it's PI filter. Lowest row: Output of the 7.7V power rail for driving the first OpAmp.

Connector board

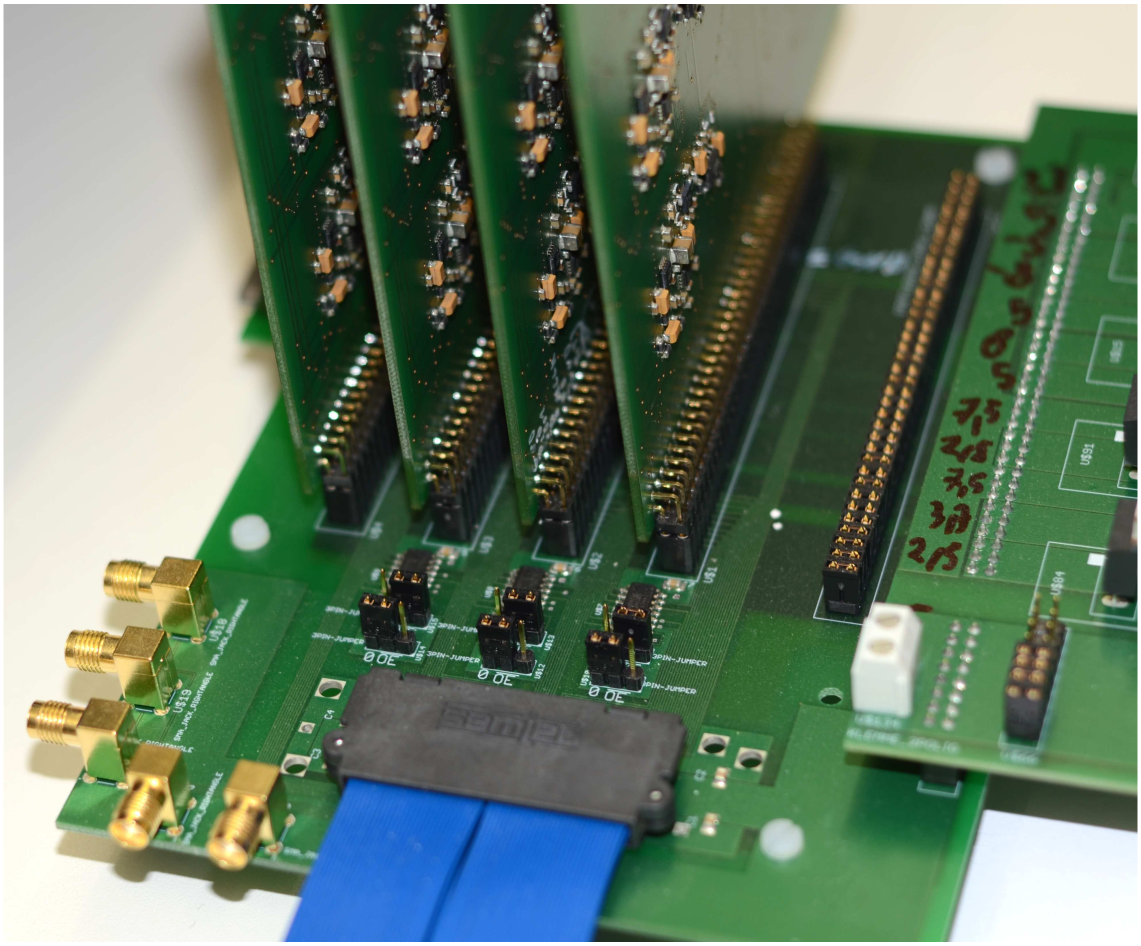

Figure 1.45: Connector board for ADC channels in version 4. The board can be populated with four cards with six ADC channels each.

The plan for the new design was to build non-stackable ADC channel cards with six channels. On the negative side this reduces the flexibility of replacing one single defect channel. On the positive side, this more compact design improves the stability and reduces the required space of the installation. Daisy chaining six ADC channels instead of 16 also allowed to reduce the SPI clock frequency from 16 MHz down to 4 MHz (with softer slopes) based on the hope to reduce high frequency pollution on the cards.

Four slots for the six channel ADC cards are provided by the new connector board. For reducing the number of FPGA pins for programming the digital resistors used by the amplifiers, we tested for three of these slots buffered shift registers for distributing the signals. Now it is possible to use only 3 FPGA pins instead of 20 FPGA pins for programming if the signals are relayed over daisy chained buffered shift registers. The data, chip select, and clock signals are transferred to the shift registers in a serial fashion. Then the new output of the shift registers is exported. This procedure is repeated

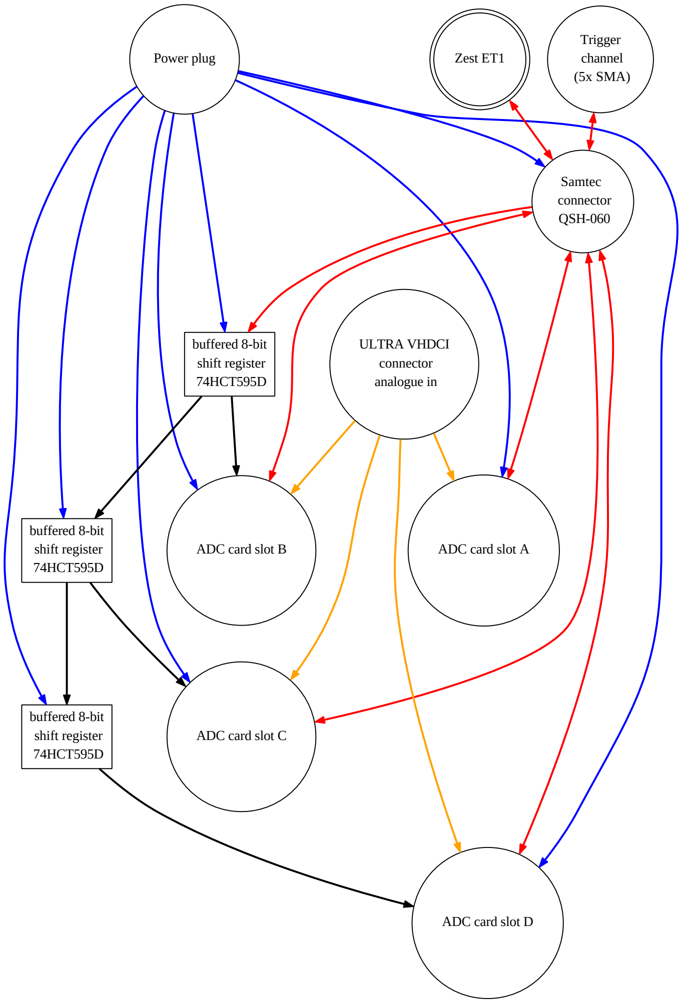

Figure 1.46: Structure of the connector board for ADC channels in version 4.

until all resistors are programmed as desired. Another positive effect is that these shift registers generate a digital barrier on the connector card after the programming was finished. The output of the shift registers will stay fixed and thus no additional extra digital noise is transmitted over this digital signal lines to the ADC cards.

Figure 1.46 shows the structure of the board (see figure 1.45) with its four card slots and its connector plugs for the power board and the Zest ET1. Between the power board and the rest of the connector board, a pin header is available for opening the power rails. This allows to insert a multimeter for measuring the current flowing into the ADC cards for the individual power rails.

FPGA controller board

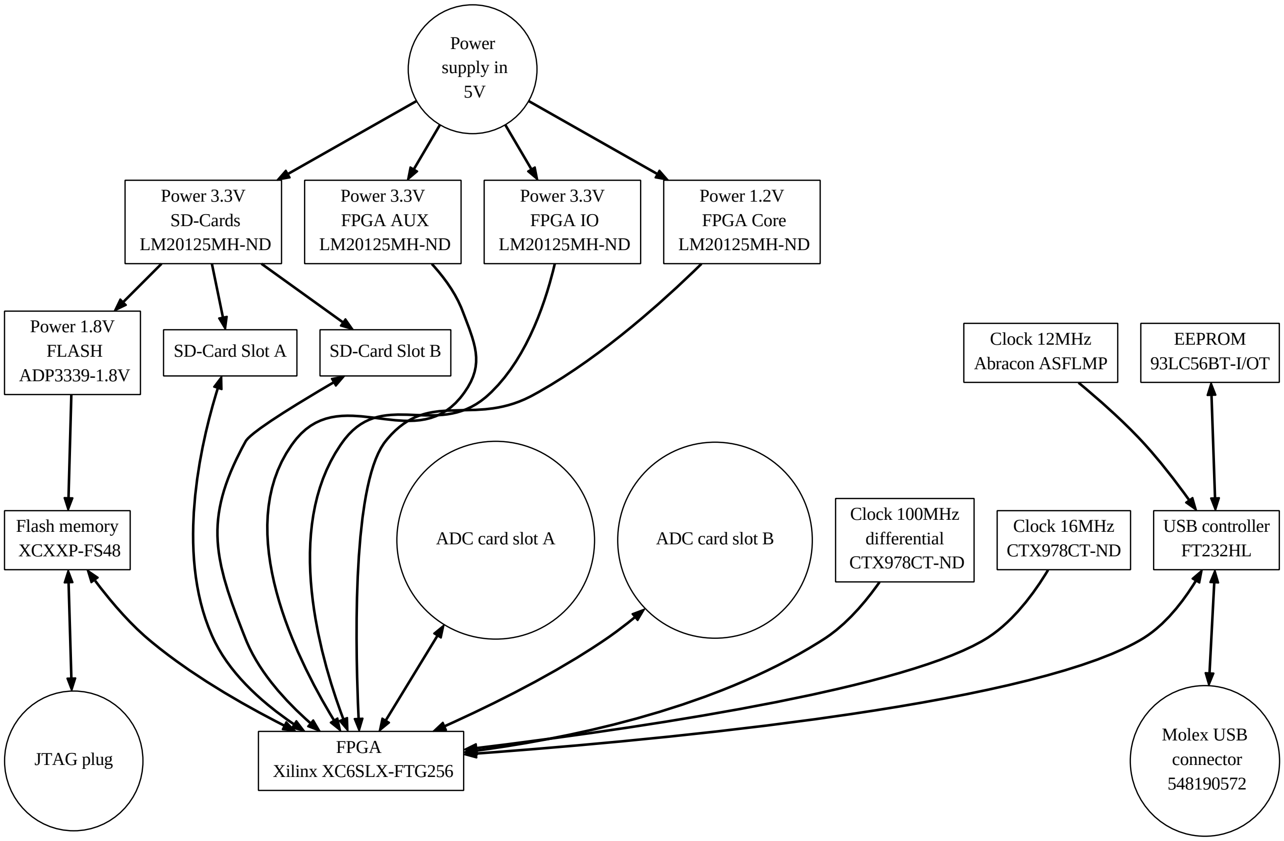

Figure 1.47: Structure of the planned FPGA board.

The plan was to design a cheaper replacement for the ZestET1 board with more user IOs and a more modern FPGA. The first question was which FPGA should be the heart of the board. Due to my experience with using Xilinx FPGA and the corresponding tool chain, I looked through their cheap Spartan 6 series. This series contains FPGAs with 0.5, 0.8 and 1.0mm lead/ball spacing. For keeping the production costs of the PCB low by using a standard process, I decided that 1.0mm ball spacing is a good value. For routing out the pins of the FPGA it is necessary to place an interlayer connection (also called VIA) into the centre of the space between four adjacent pins. From this VIA the connection is routed out on one of the available layer without crossing another VIA or connection. This requires several PCB layers. The number of necessary layers depend on the number of pins. In the 1.0mm spacing are FPGA with 256 (16 x 16), 484 (22 x 22) , 676 (26 x 26), and 900 (30 x 30) pins available. The more rows or columns of pins a FPGA has, the more PCB layers are required for routing them out. For keeping it cheep, I choose a FPGA (Xilinx XC6SLX25-2FTG256C for roughly 30Euro) with 16 x 16 pins which require a 16 layer PCB in the worst case and has 186 user IO pins. Such a modern FPGA requires a lot of capacitors to keep its many different supply voltages stable, which makes a compact design even more complicated. With a lot of

effort I was able to reduce the number of required layers to 10, which is below the recommended number of layers.

I connected 138 of the 186 user IO pins to two 36x2 pin headers for connecting them with base station components. In addition, I connected to a 100MHz (differential) clock and a 16MHz clock to the FPGA. For data storage, I provided two SD card slots which are connected for full speed (not only the SPI fall-back mode) data transfer. For connecting the board to an external PC, I envisioned a USB interface. The USB interface is based on a FTDI chip (FT232H - Hi-Speed Single Channel USB UART/FIFO IC) which allows very easily to exchange data (with up to 40MByte per second) with a connected computer, just by operating a simple FIFO. Even the necessary USB drivers are delivered by FTDI. The USB IC is cheap and costs below 5Euro. It is important to note that this USB interfaces can be connected to an external computer but it can't be used to connect an external USB drive to the FPGA board. For this direction other chips are necessary.

For programming the FPGA on power-on, a special flash memory is required. I choose to use Xilinx Plattfom Flash XL memory and included it on the board.

Another important issue is the power supply of a modern FPGA. First of all, it has to be very stable. Second, it needs to show a monotone voltage increase within a given time-window when power is turned on. Thus the individual power rails (3.3V for IO banks, 3.3V for AUX and 1.2V for the core) need to be synchronized. For the rest on the board an additional 1.8V for the flash is required and 3.3V for the SD cards.

The whole structure of the FPGA board is shown in figure 1.47. Due to time and money constrains as well as risk management, we decided not to produce the FPGA board. However, I complete the design before that decision. Figure 1.48 and 1.49 shows only the upper and lower layers of that 10 layer design.

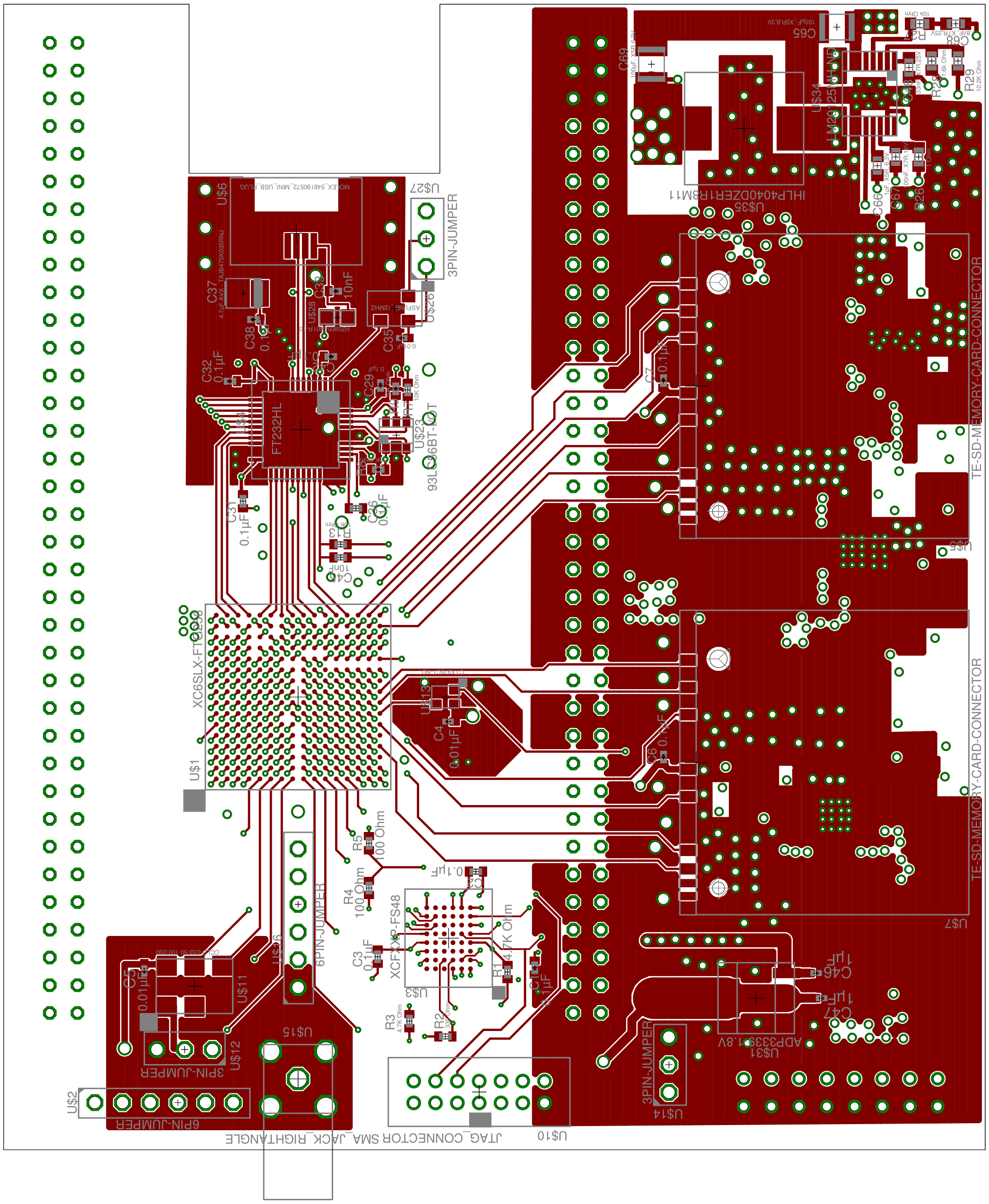

Figure 1.48: Design of the upper side of the FPGA board.

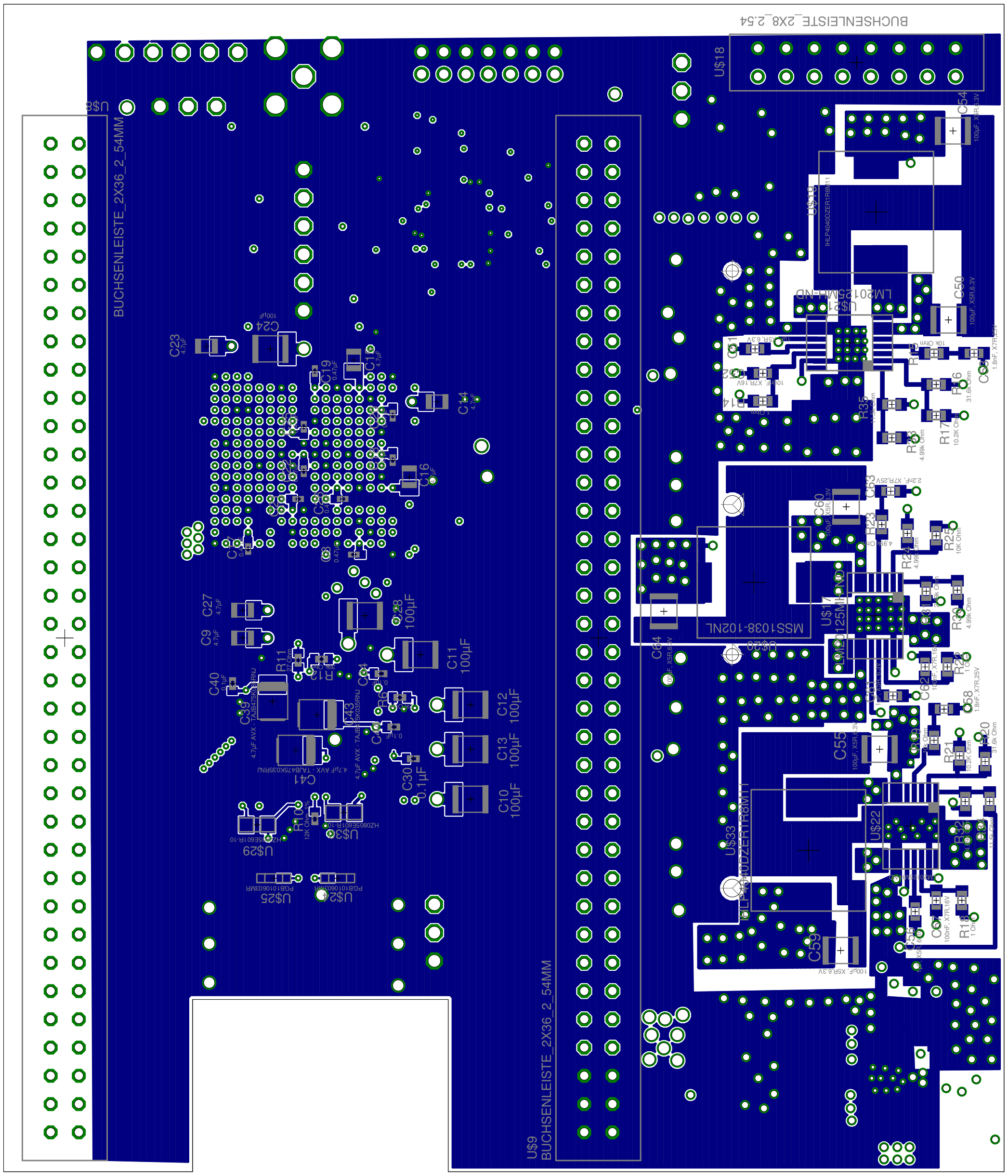

Figure 1.49: Design of the flip side of the FPGA board.

ADC channel cards (non-programmable)

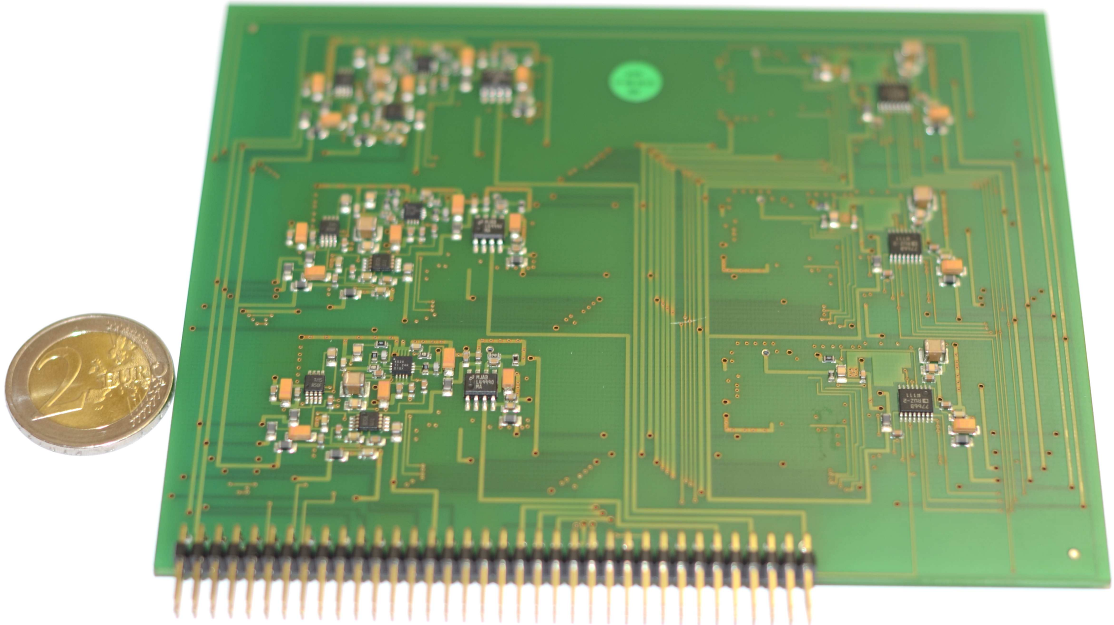

Figure 1.50: Version 4 (non-programmable amplification) of the analogue-signal recording channels. One card contains six recording channels.

The fourth version of the ADC channels were produced in two formats. Here I describe the simpler version without programmable amplifier stages. The analogue front-end (see figure 1.51) contains analogue filters for conditioning the frequency components' amplitudes below 1Hz and above 10kHz. The first amplifier, chosen for its extremely low-noise behaviour, amplifies the incoming signal $1+\frac{1070\Omega}{60.4\Omega}=18.7$ times. It's feedback path contains a low-pass filter construction, that decreases the amplification factor for frequency components above 10kHz. This is followed by a high-pass filter with a cut-off frequency of 1Hz. The first amplifier is operated with ±7V supply voltage, allowing some 100mV DC offset before they are removed by the high-pass filter. Then a fully differential driver converts the signal into a differential signal which is feed into the ADC IC.

Six of these ADC channels were bundled onto one card module (see figure 1.50). Daisy-chaining only six ADC allowed to reduce the SPI clock down to 4MHz as well as reducing the signal slope steepness. This reduces over- and undershoots which can cause distortions for the measurement. These PCBs were produced and assembled by kwi-electronic.de (a company in Bremen).

For operating the ADC channels two different reference voltages are required. Figure 1.52 shows the structure behind the ADC channels.

Input stage:

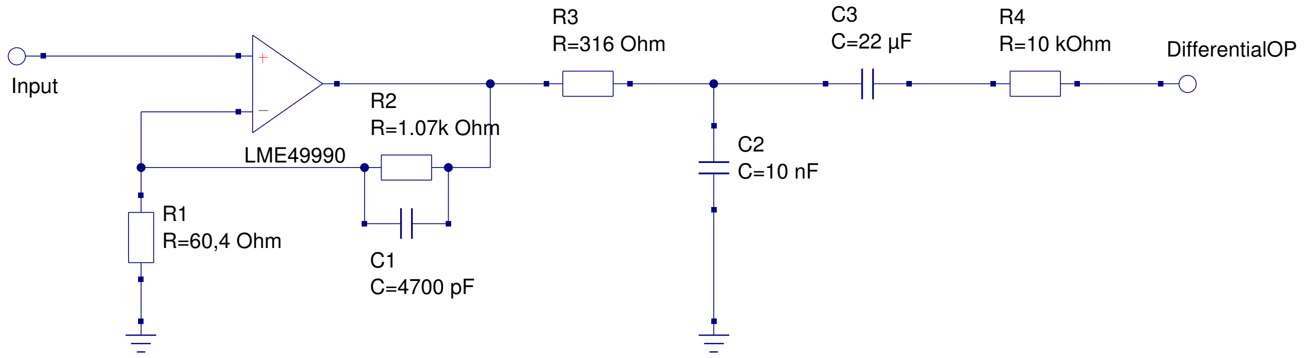

Differential stage:

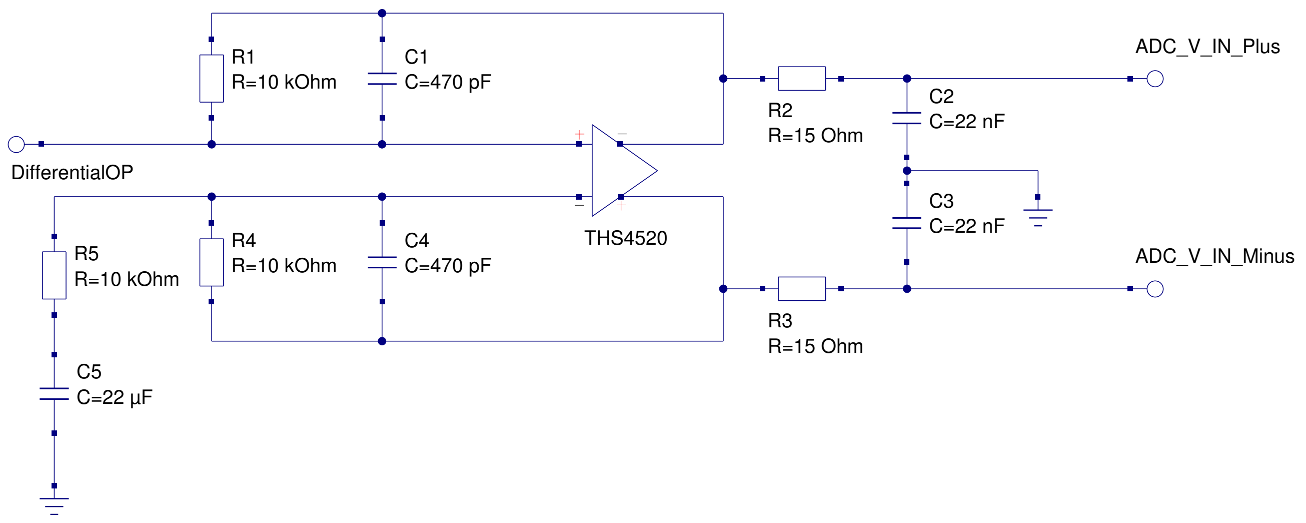

Figure 1.51: Analogue front-end of version 4 (non-programmable amplification) (see figure 1.50 for the realisation).

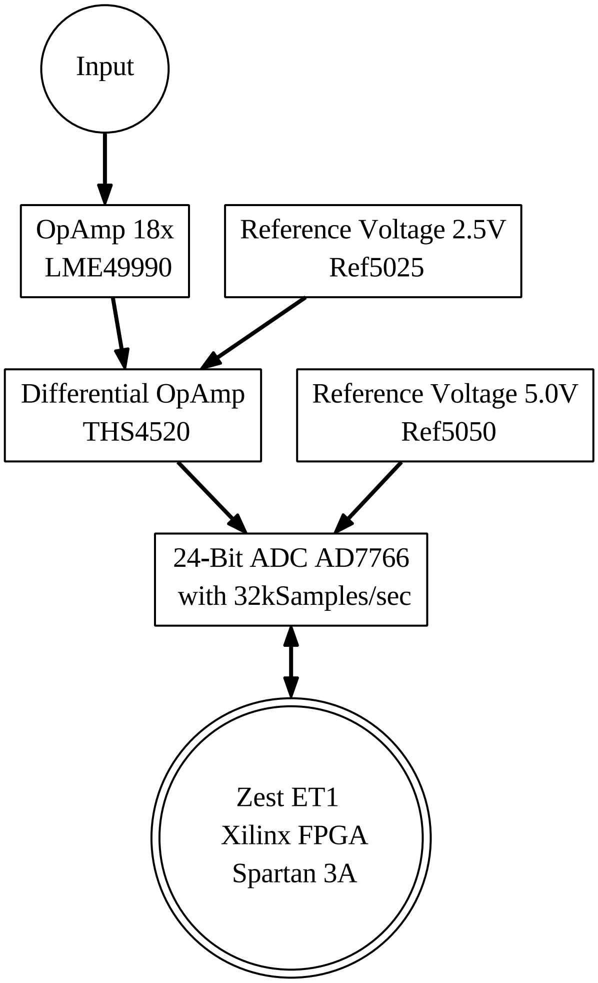

Figure 1.52: Structure of version 4 (non-programmable amplification) of the analogue signal recording cards.

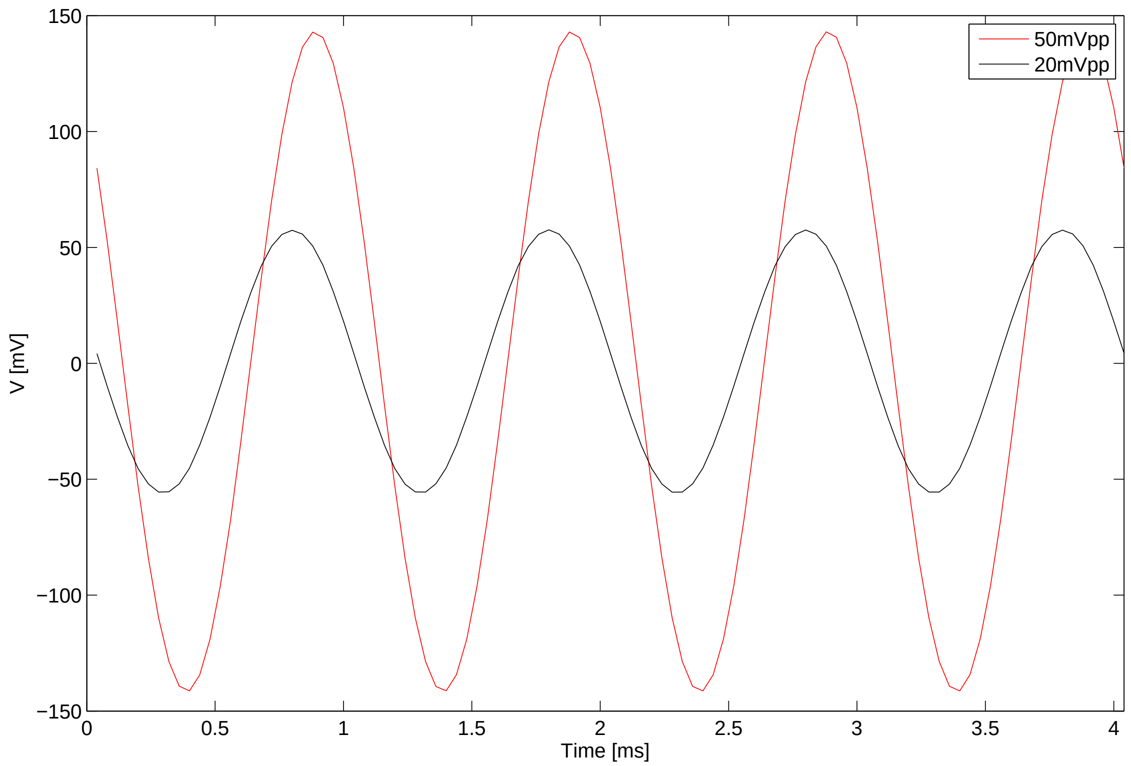

Figure 1.53: Measurement of a 1kHz sinusoidal wave with 50mV and 20mV peak-to-peak.

Similar to the test measurements that I have done for version 3 of the card, I did recordings with the simple version 4. The figures 1.53 (1kHz sine wave) and 1.54 (100Hz sine wave) show the acquired data with large signal amplitudes. For all the plots, the y-axis is calibrated to a 500mV peak-to-peak sinusoidal waveform. All the curves with high signal amplitudes look very clean.

For figure 1.55 and its close-up for the smallest amplitude shown in figure 1.56, again the three BNC damping elements (together -36dB) were installed between the signal generator and the input plug of the recording system. Compared with the curves from version 3 (see figure 1.37), the acquired data for version 4 looks a bit more noisy. One reason could be that the signal has to travel over longer cables. The signal flows as follows: The signal leaves the output BNC plug of the waveform generator and then runs through the stacked damping elements. After these reduction elements, an unshielded 1m cable follows where it's clamps are connected to 10cm wire pieces. These wire pieces are screwed into a breakout box. This breakout box is connected via a 1m SCSI cable to the recording system. All this together allows to collect more noise on the way.

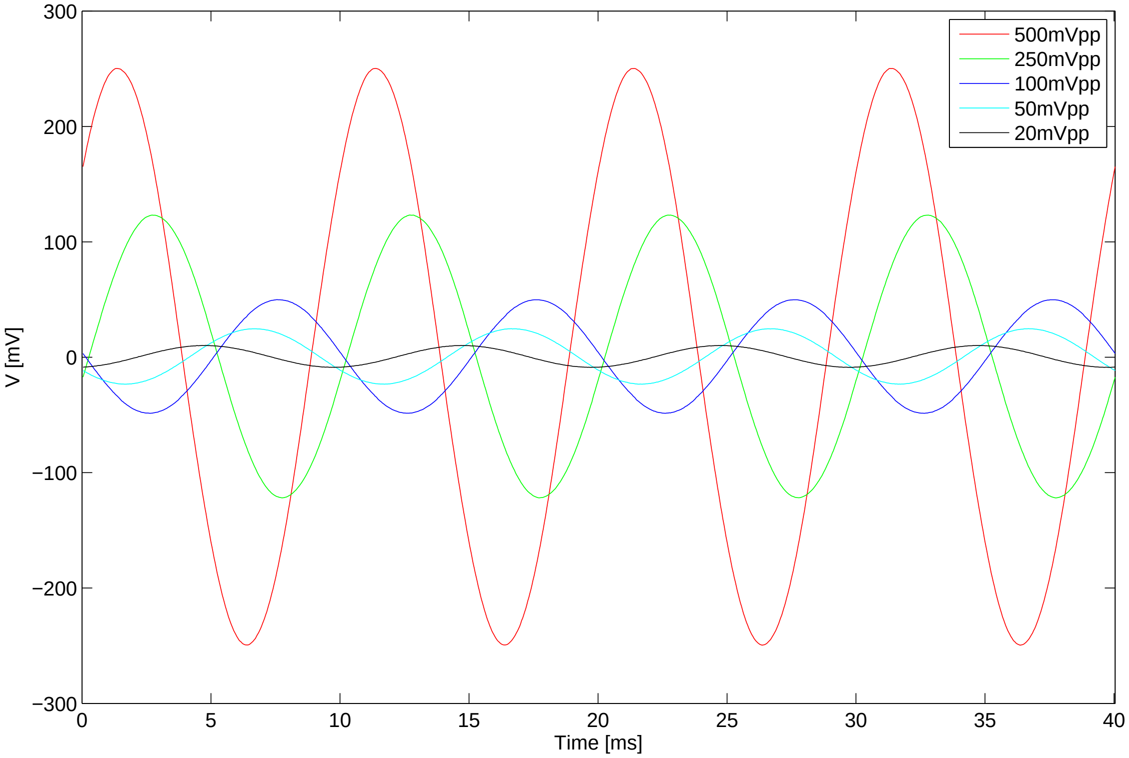

Figure 1.54: Measurement of a 100Hz sinusoidal wave with large amplitude (from 500mV peak-to-peak down to 20mV peak-to-peak).

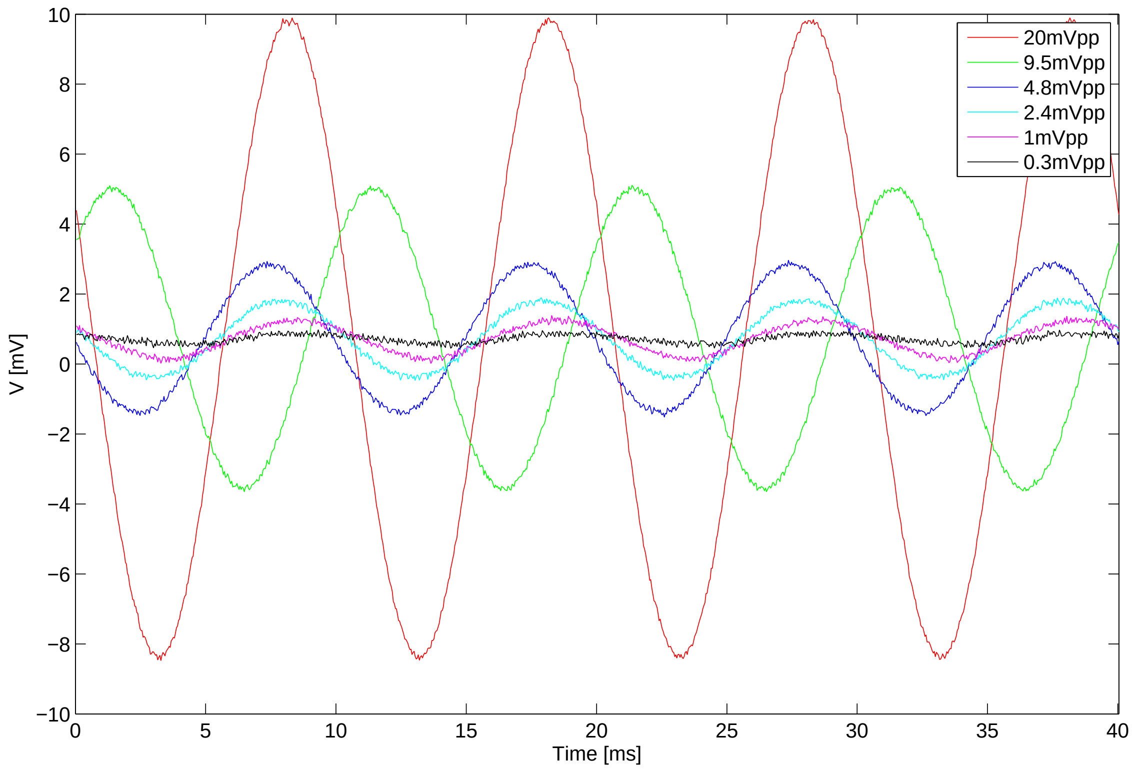

Figure 1.55: Measurement of a 100Hz sinusoidal wave with small amplitude (from 20mV peak-to-peak down to 300μV peak-to-peak).

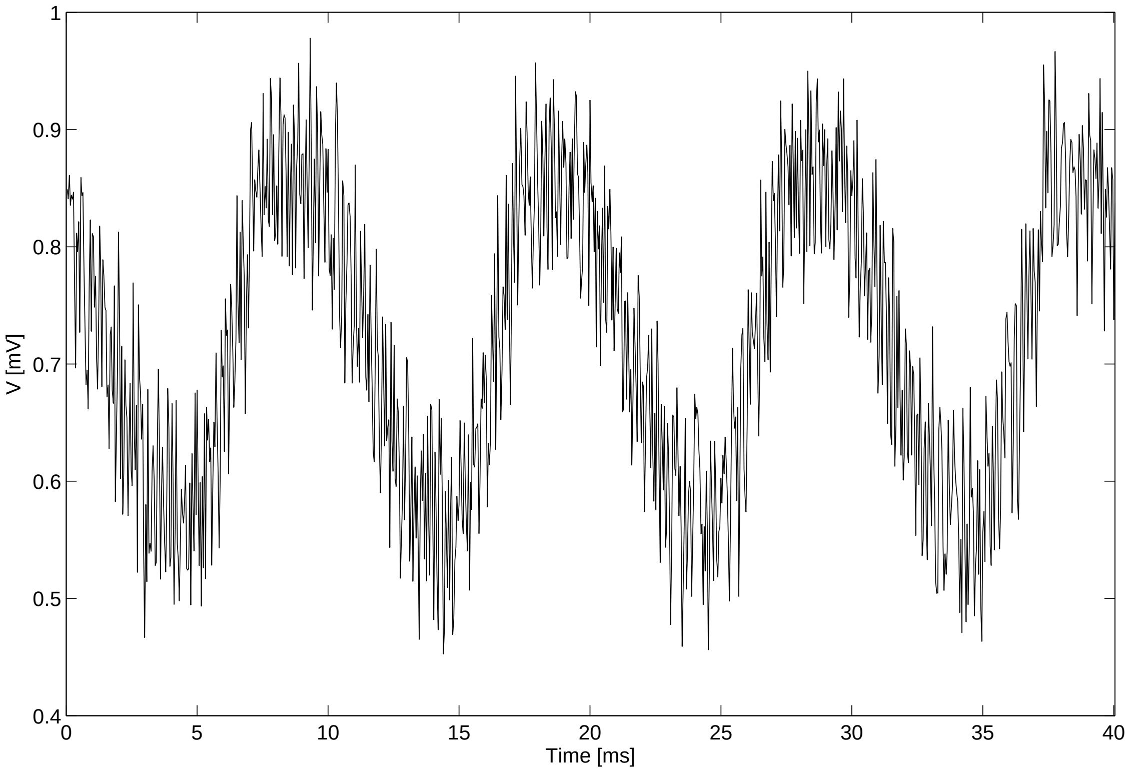

Figure 1.56: Close up of the waveform with the smallest amplitude from figure 1.55.

ADC channel cards (programmable, not working)

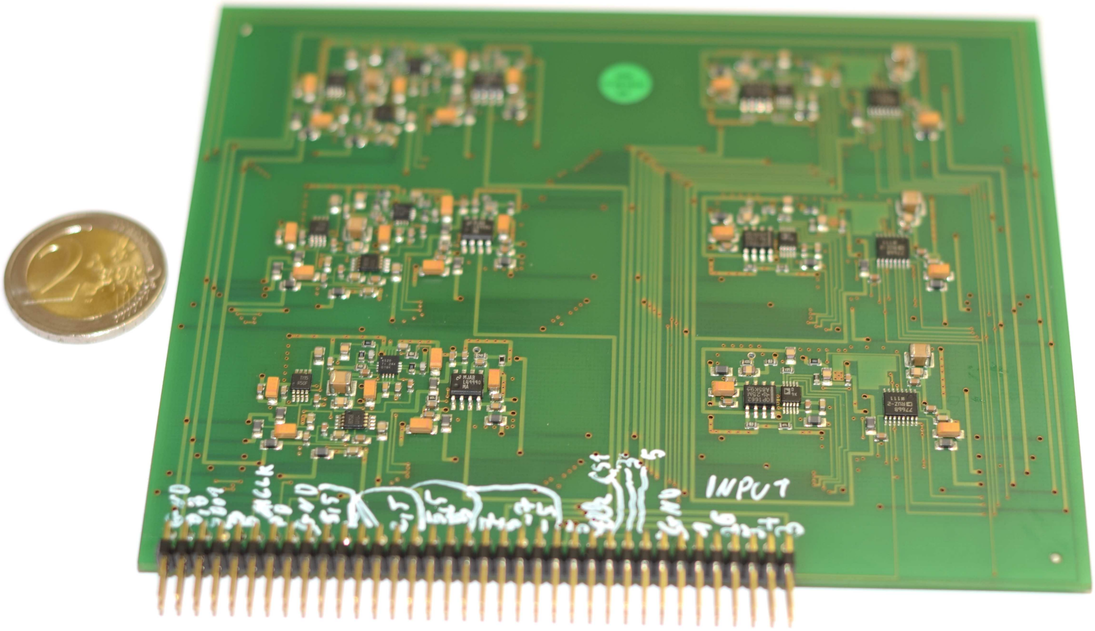

Figure 1.57: Version 4 of the analogue signal recording cards. One card contains six recording channels with individually programmable amplification. The PCB were produced and assembled by kwi-electronic.de (Bremen).

Beside the simple realisation of version 4, I designed another one with two programmable amplifier stages (see figure 1.57). The first fixed pre-amplifying state with the very low-noise amplifier and the final fully differential driver stage are the same as in the simple version. In between I placed two additional amplifiers with high-pass filters (with a cut-off frequency of 1Hz). Within the feedback loop of these amplifiers, digitally programmable resistors are integrated. This allows to individually control the amplification factor of these two stages. See figure 1.59 and 1.60 for the analogue front-end. Figure 1.61 shows the organisation of these ADC channels.

Before production the whole circuit and especially the filters, everything was carefully planned and simulated. However, when we tested these ADC channels they produced strange results. It took Andreas Kreiter and me several hours to localize the source of the problem. We had to remove many components on the board before we could isolate the responsible IC. Figure 1.58 shows the recording with only the last programmable amplifier stage and the fully differential driver stage. Since we knew from the simpler version, that the driver stage works fine, the amplifier and digital resistor combination had to cause the problem. After some more debugging, we found that the digitally programmable resistors are the problem. This was hard to believe at first, because we used them in version 3 very successfully. However, in version 3 these resistors had only to conduct positive voltages. In version 4, they have to transmit negative and

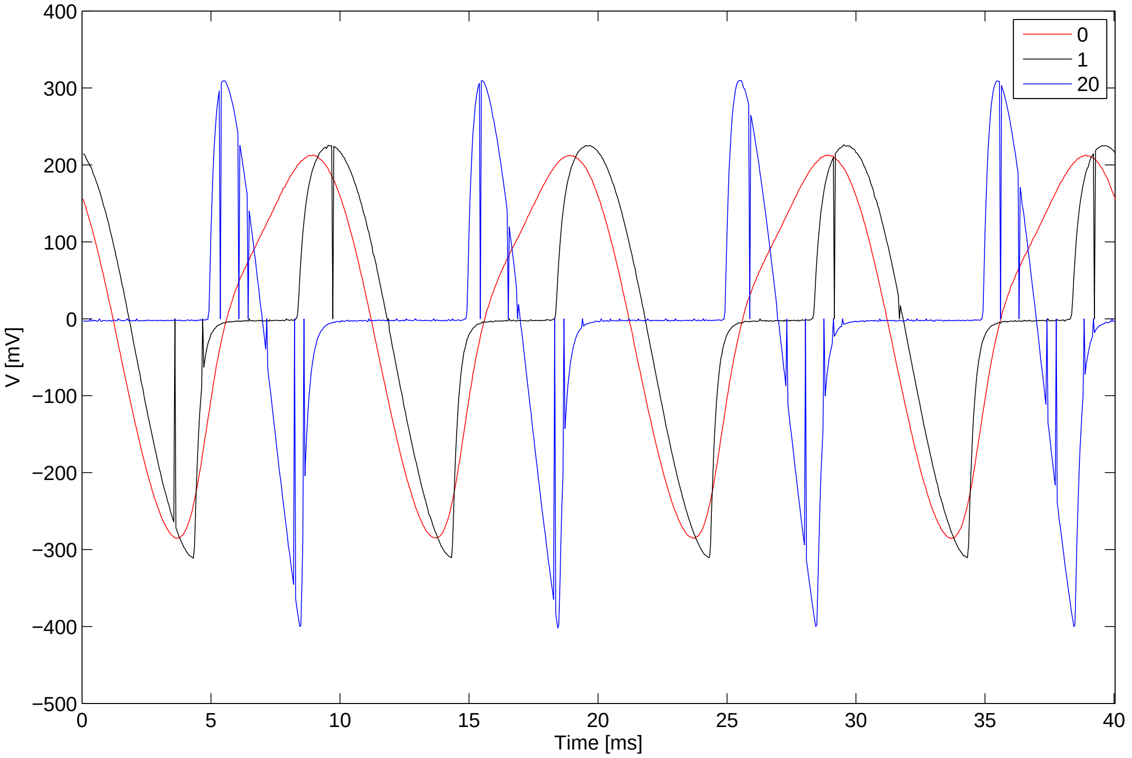

Figure 1.58: Measurement of a 100Hz sinusoidal wave with 500mV peak-to-peak. The test signal was injected into the input pin of the third amplification stage (the OpAmp before the differential amplifier.) The connection from this OpAmp to the two prior OpAmp was removed before the measurement. With the smallest amplification setting (0), the signal looks already deformed. Increasing the amplification (1 and 10), destroys the signal integrity completely.

positive voltages. In the manual of these resistors there is a not very obvious note, which explains that signals between ground and supply voltage are only allowed and that the direction of flow doesn't matter. In retrospect, this doesn't include negative voltages. In a next step we replaced the programmable resistors by normal leaded resistors and then the ADC channel worked with fixed amplifications.

In the following, I will show some measurement with differently modified programmable version 4 channels for better understanding the involved components for future designs.

Input stage:

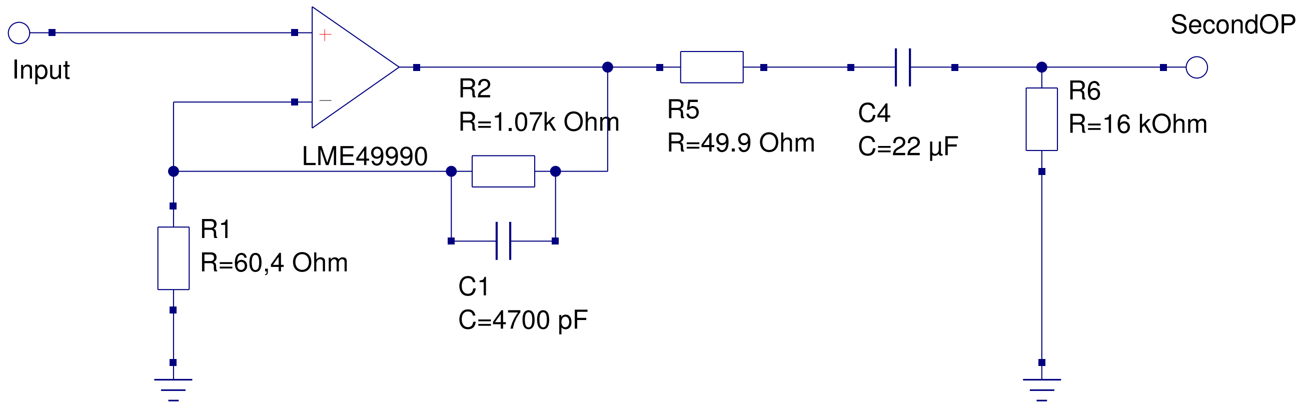

Programmable amplifier 1:

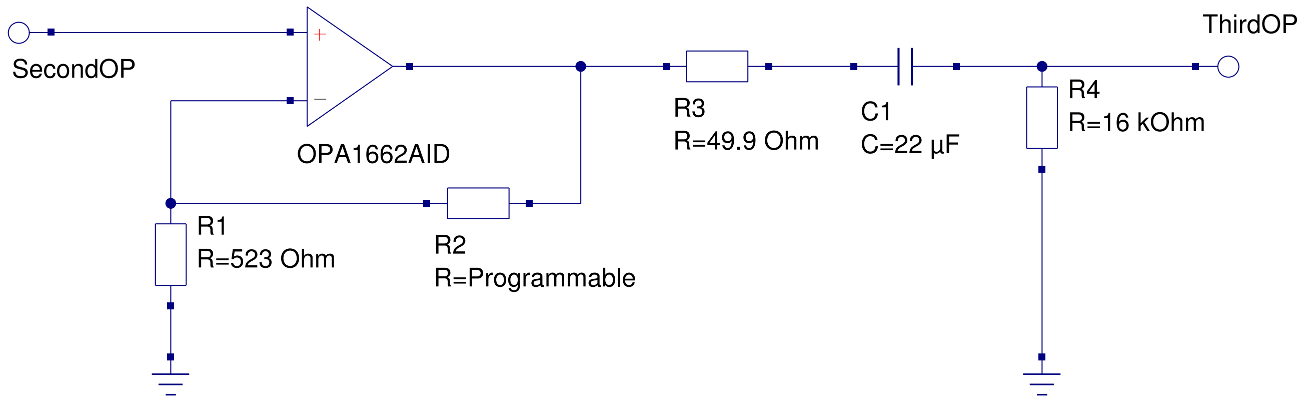

Figure 1.59: Analogue front-end of version 4 (programmable amplification) (first two stages).

Programmable amplifier 2:

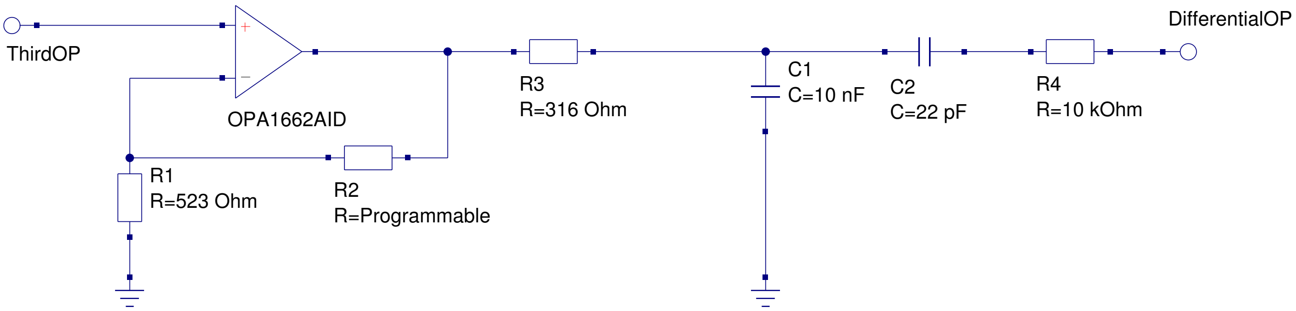

Differential stage:

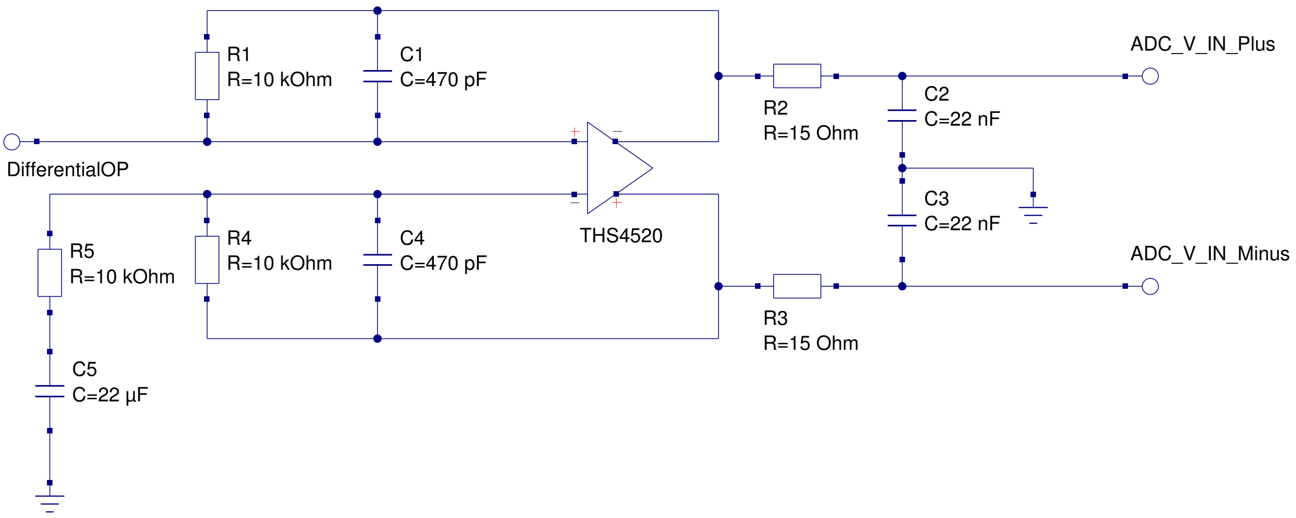

Figure 1.60: Analogue front-end of version 4 (programmable amplification) (second two stages).

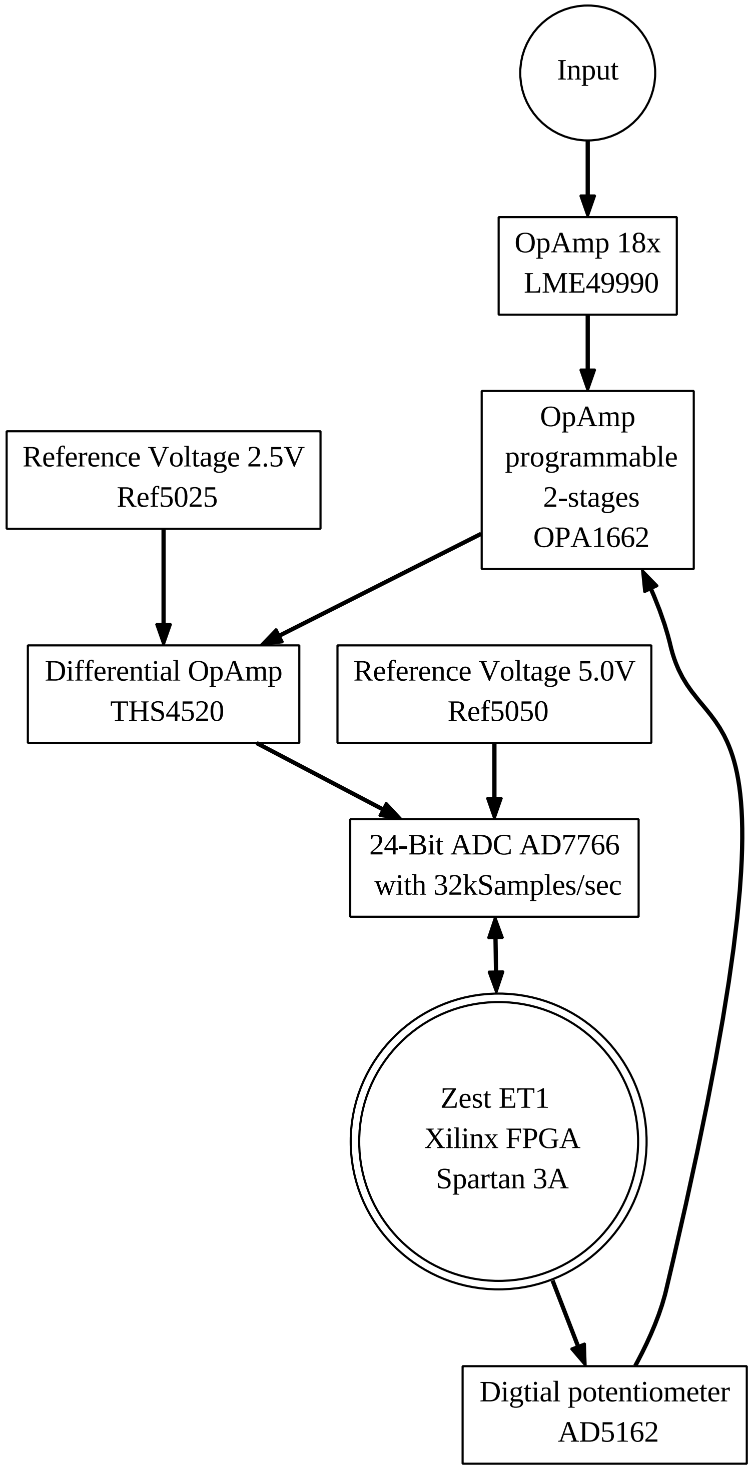

Figure 1.61: Structure of version 4 (programmable amplification) of the analogue signal recording cards.

ADC channel cards (replace programmable by fixed resistors)

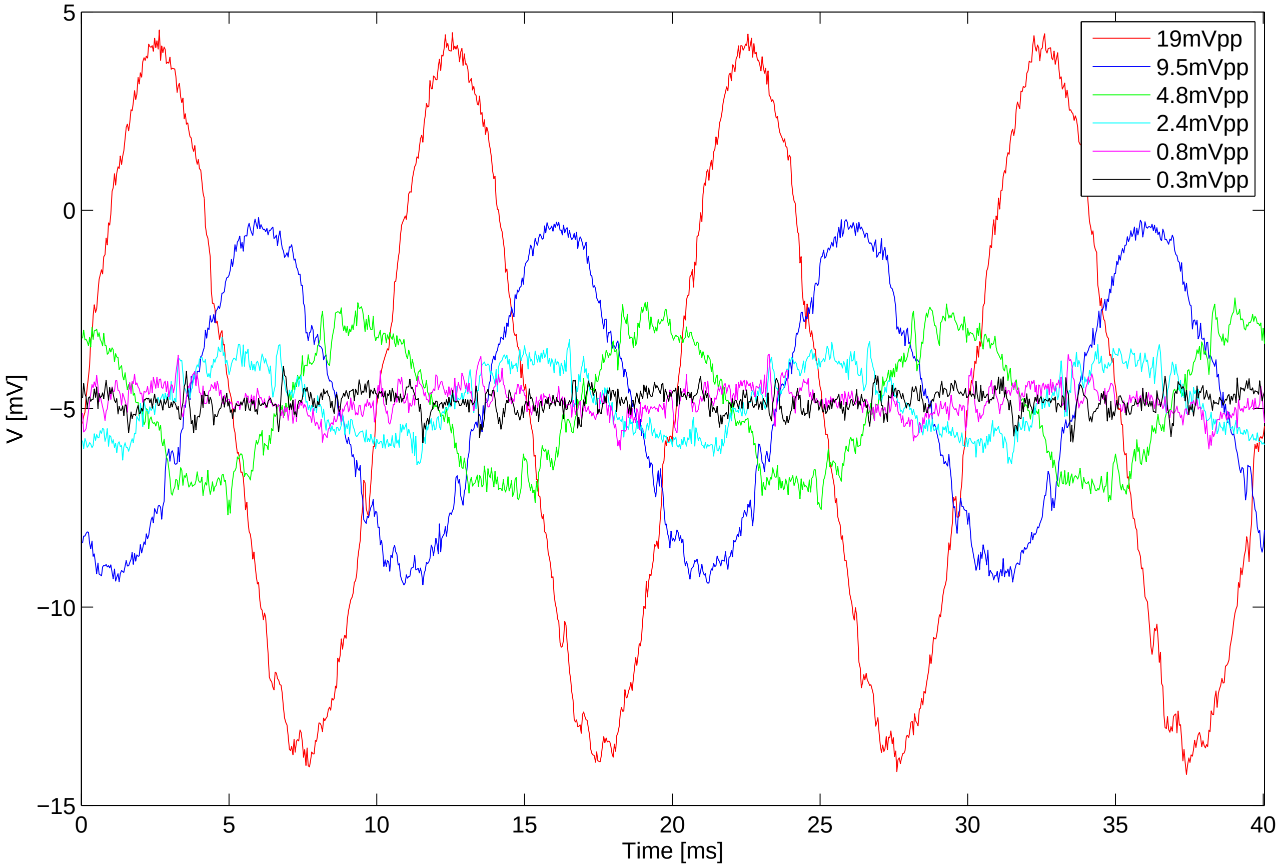

Figure 1.62: The digitally programmable resistors are replaced by 510Ω resistors.

In a first step we removed the digital resistor IC forcefully from the analogue front-end of one channel. Then we soldered leaded resistors directly on top of the amplifiers legs. We used as short as possible leads for the new resistors. Nevertheless, the resistors are creating a kind of loop antenna.

For the figure 1.62 we installed 510Ω resistors. This results in $1 + \frac{510}{523} = 1.98$ times amplification for each of these two amplifying stages. The result looks very noise compared to the usual curves. The we increased the resistors to 5kΩ which results in $1 + \frac{5000}{523} = 10.6$ times amplification for each of these stages. The horrible result of this modification is shown in figure 1.63. It is unclear if this is a result of the antenna property combined with the high resistance (= low currents) or if it is a problem with the operational amplifier ICs. However, I will avoid the use of these amplifiers in the next version of ADC channels.

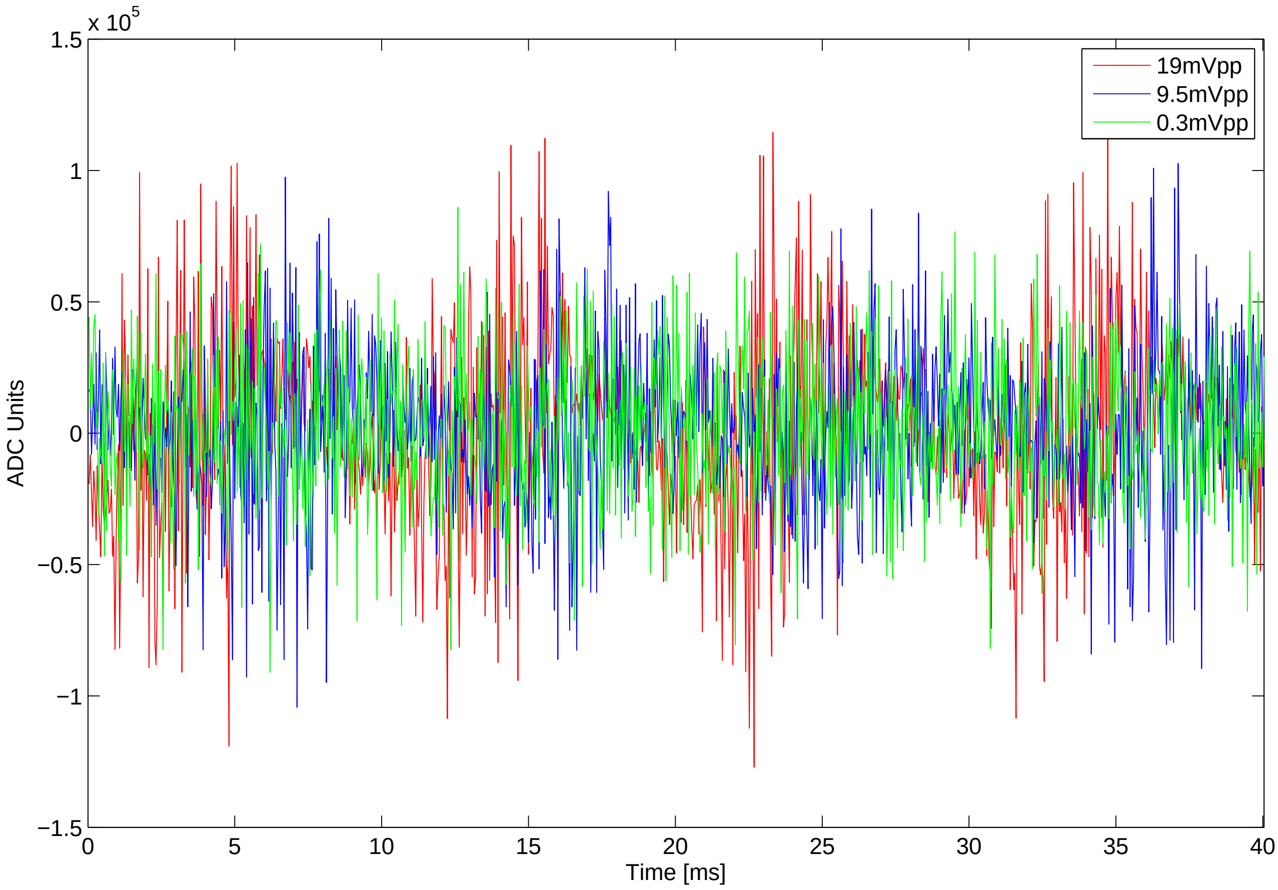

Figure 1.63: The digitally programmable resistors are replaced by 5kΩ resistors.

ADC channel cards (programmable stages are removed)

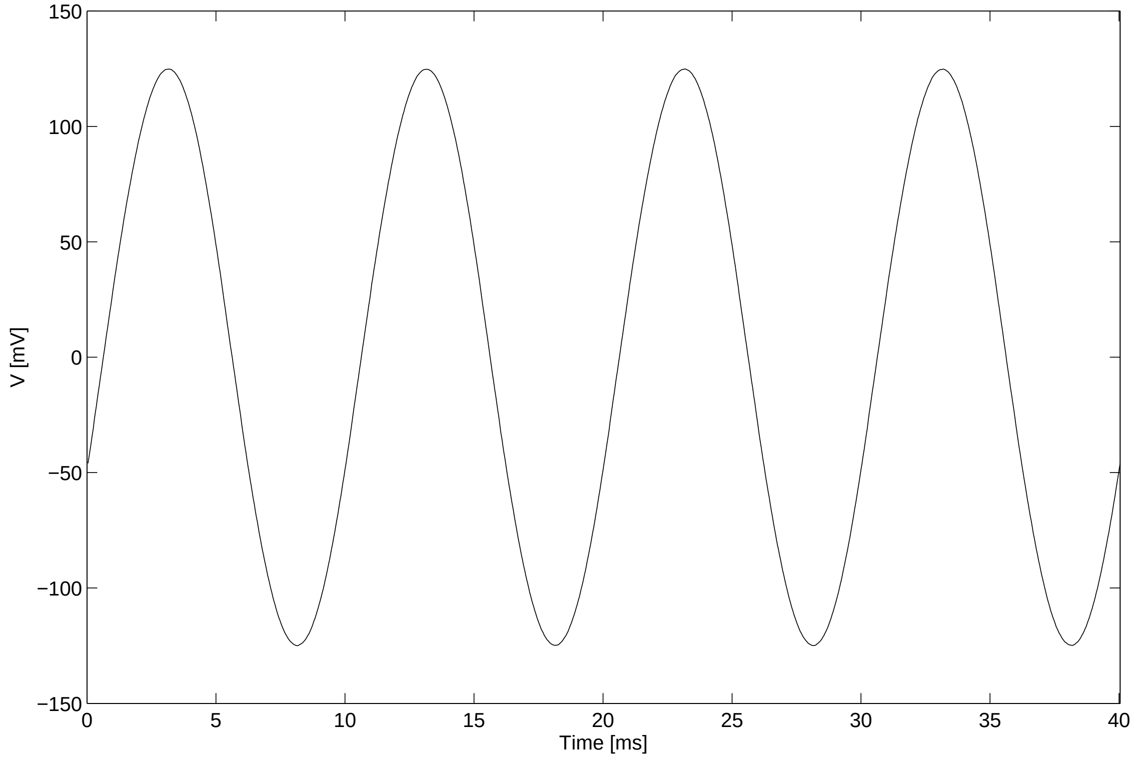

Figure 1.64: By removing components both programmable amplifiers were removed. With a leaded resistor, a connection between the first amplifier and the differential amplifier was established. Now the signal path is similar to the non-programmable version of this card. The recorded signal (250mV peak-to-peak sinusoidal wave) looks regular.

For another test, I removed the 49.9Ω resistor after the first amplifier and the 316Ω resistor after the second programmable resistors. Then I soldered a leaded 310Ω resistor as bridge over these two removed components. As result, the circuit looks now like the simple non-programmable version of these ADC channels. Figure 1.64 shows the result of this operation. For a large amplitude this procedure worked fine.

In a second step, I replaced the 1.07kΩ SMD resistor of the first amplifier by a leaded 11kΩ resistor. This modifies its amplification to $1+\frac{11k\Omega}{60.4\Omega} = 183$. Figure 1.65 shows the results with this setup. For larger amplitudes the recording looks clean and normal. However, for smaller amplitudes the curves look strangely deformed. Again it is unclear if this is the result of the antenna loop created by the two leaded resistors (plus the low current flow due to the high resistance in the amplifier's feedback loop) or if it is a result of a too high amplification factor. For future designs it may be important not to use too high amplification factors in the individual stages.

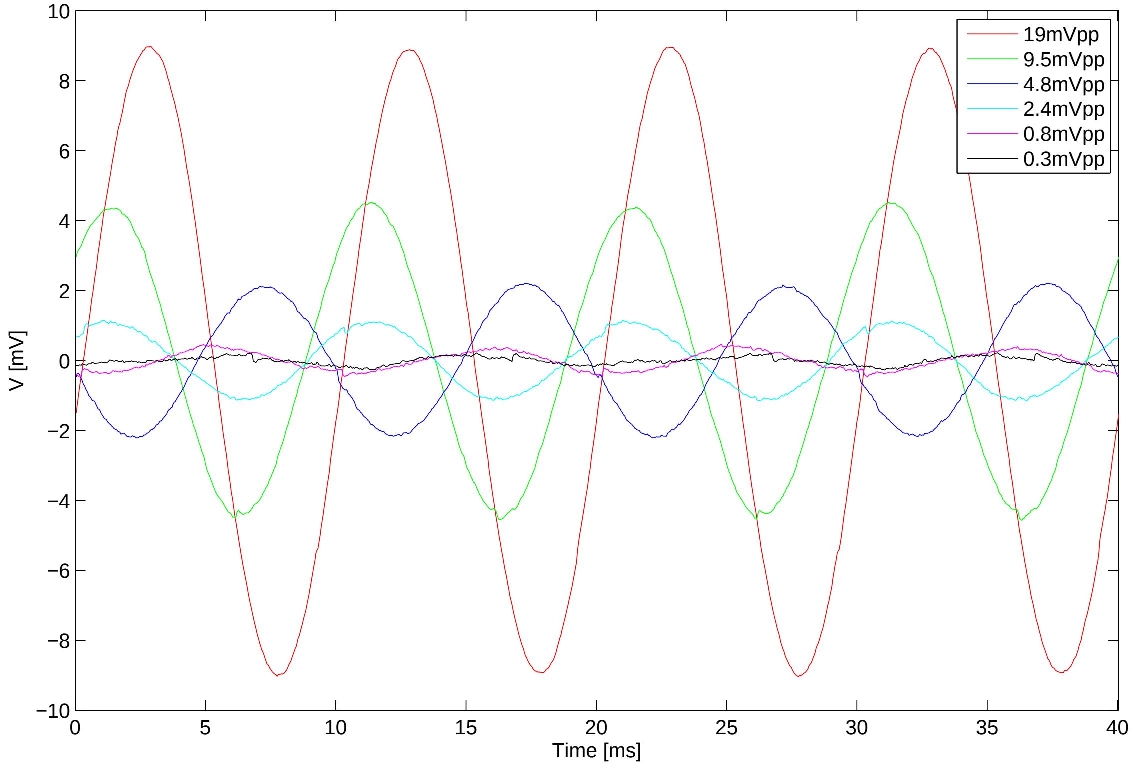

Figure 1.65: Continuing the modification after removing the programmable stages. The amplification factor of the first OpAmp is changed by replacing the 1.07kΩ (SMD) resistor by a 11kΩ (leaded) resistor.

ADC channel cards (comparison)

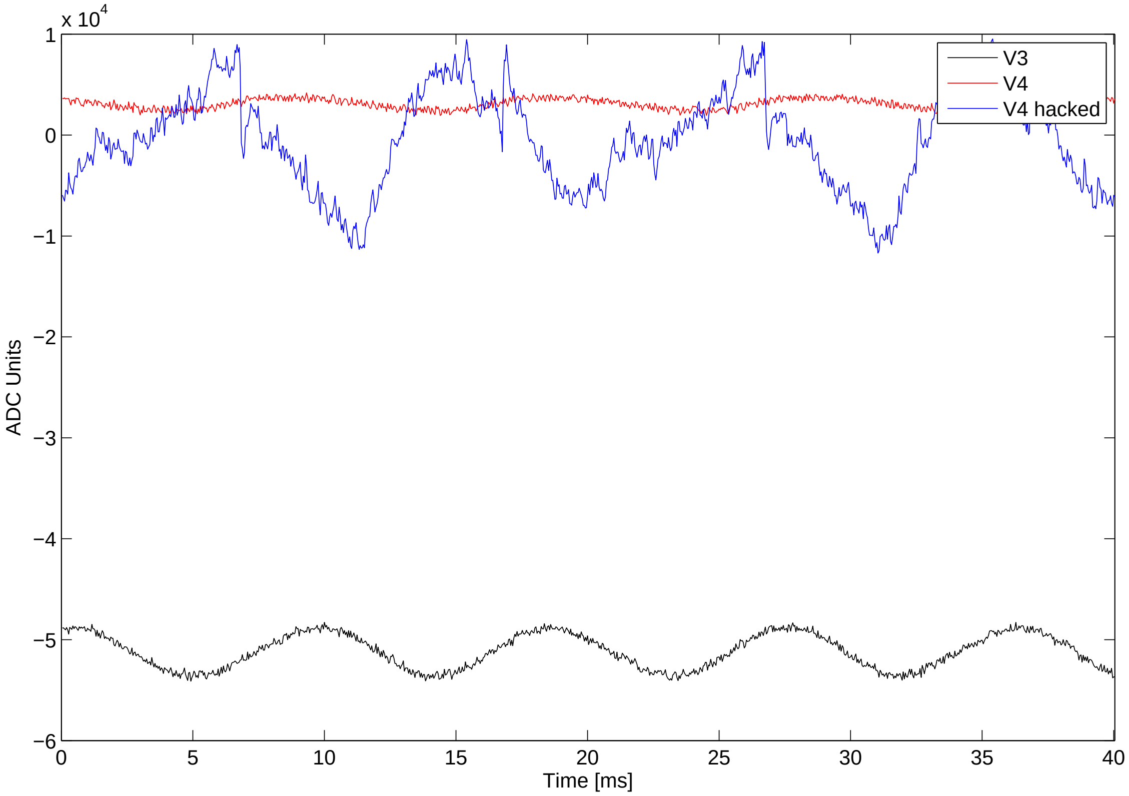

Figure 1.66: Comparison of the ADC channels version 3, version 4 (simple, non-programmable with 18.7x) and version 4 (programmable stages removed, modified to 183x). All three curves show the result of recording a 100Hz sine wave with a 0.3mV peak-to-peak amplitude

In figure 1.66 results of the recording with version 3, version 4 (simple, non-programmable) and version 4 (modified version, see figure 1.65) are compared. The comparison shows that version 3 still delivers (noise-wise) the best recording results. However, the simple version 4 does also a good job especially when the extra long cables for feeding in the input are taken into consideration.

With this information, now a new version can be designed.

1.5.5 Version 5

Based on the gained knowledge from the last four versions, I started with the design of version 5. As result from the earlier versions, the new version will be rapid-prototyped and iteratively and intensively tested during its assembly. My plan is to build one channel based on adapter boards, 0805 SMD components and breadboards. This allows faster changes of the components. The rough design will look like follows:

The first stage will amplify everything below 10kHz with approximately 20x (low-pass filter in the feedback loop). Then a high-pass follows with a cut-off frequency with 1Hz. A second amplification with 20x for the components below 10kHz is applied to separate the high-pass from the fully differential stage. This is important because without this second amplifier the fully differential stage would shift the cut-off frequency of that high-pass filter to much larger frequencies. Keeping the cut-off frequency down would require extremely large capacitors. The high-pass filter with 1Hz cut-off frequency is absolutely necessary, otherwise DC offsets would drive the amplifier into saturation. Thus this construct with the second 20x amplifier seems to be the lesser evil. Figure 1.67 shows the plan for the first two stages.

After these two stage, one fully differential driver with a low-pass filter (10kHz cut-off frequency) follows (see figure 1.68). This driver converts the single-end signal into a fully differential signal. Furthermore, within the feedback loop of the driver, I plan to install digitally programmable resistors for controlling the amplification. Depending on the performance of these three stages, I also prepared an optional programmable fully differential amplification stage (see figure 1.68).

Stage 1:

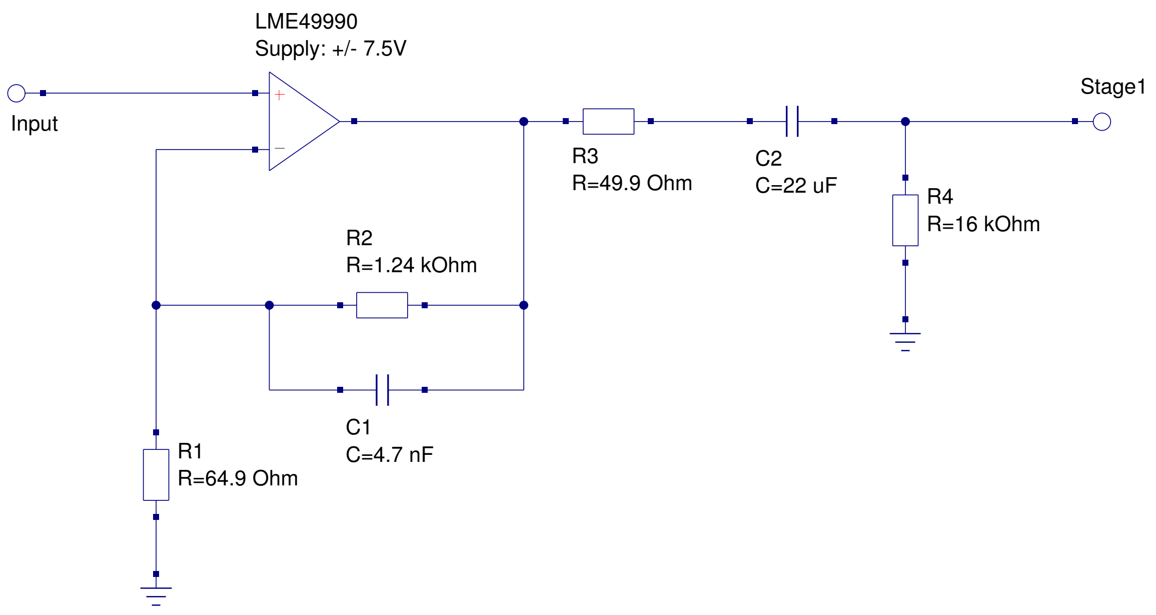

Stage 2:

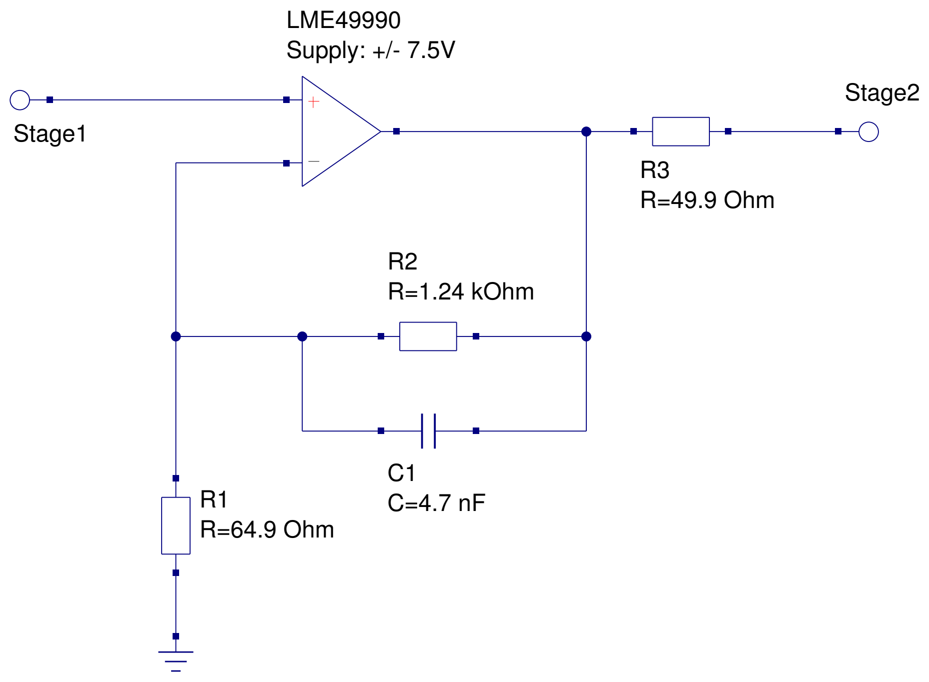

Figure 1.67: First two amplification stages with 20x amplification each and a high-pass filter with 1Hz cut-off frequency in between. Within the feedback loop a low-pass filter is installed for reducing the frequency components beyond 10kHz.

Stage 3:

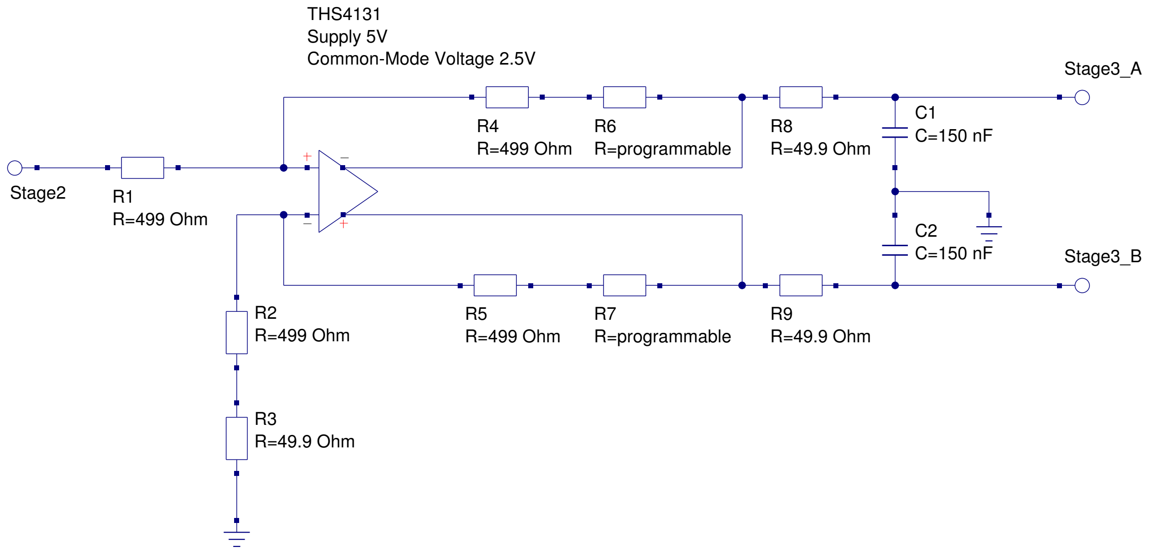

Stage 4:

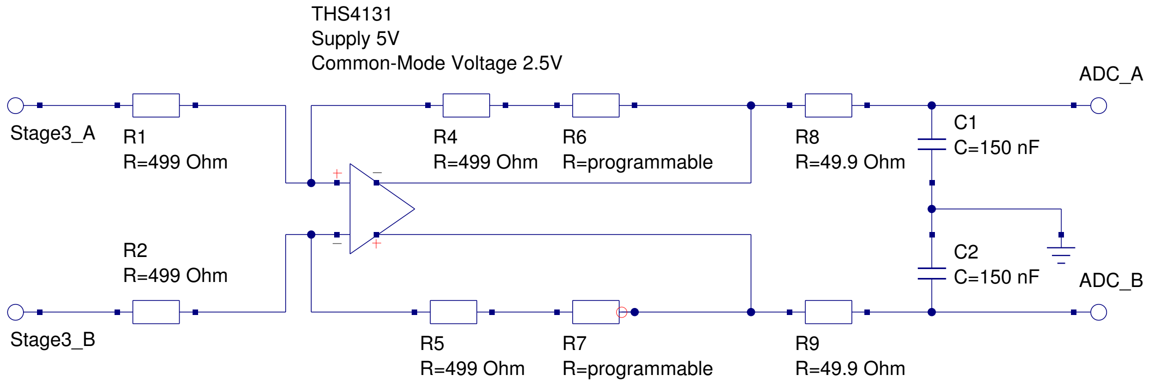

Figure 1.68: Fully differential driver converts the single-end signal into a fully differential signal. The amplification of this driver can be programmed by digital resistors and possesses a low-pass filter (10kHz cut-off frequency). Another optional fully differential and programmable amplification stage is possible when the amplification by the earlier three stages was not enough.

Chapter 2

Firmware of the base station

The actual version of the base station, and thus its firmware, has two major functions. One is to create a link between the implant and an external PC via an Ethernet network connection. The second one is to create a platform for electro-physiological wire-bond measurements which are also delivered to an external PC via an Ethernet network connection. The firmware of the base station run on an Orange Tree ZestET1 FPGA board. The ZestET1 contains a gigabit Ethernet hardware network stack, which allows to interchange information, via different network protocols, between the FPGA and connected network devices. Furthermore, the ZestET1 provides many unused FPGA IC pins allowing us to connect custom hardware to it.

The task of the firmware is to interface these custom hardware devices and provide a simple standardized interface via network to an external PC. In addition, we want that the FPGA of the base station performs all the tasks which have to be done in real time. Implementing these real time tasks on a PC would make software development unnecessary hard.

The written firmware is capable of controlling a Zarlink ZL70102 RF transceiver IC, interact with the Neuro-ASIC from the ITEM (via Zarlink RF link), service ingoing and outgoing trigger channels, set the amplification of the wire-bond electro-physiological recording channels (via SPI- interface controlling AD5162 digital resistors which are placed within the feedback loop of the op amps) as well as collecting the electro-physiological data from AD766 24-bit ADC ICs via SPI.

The hand-crafted parts of the VHDL source code for this firmware (excluding the test-benches, automatically generated VHDL files for FIFOs and source code from Orange Tree) has a size of approximately 800000 Byte. The size of the source code shows that the firmware is very complex. In the following, I want to give an overview over its structure. The intention of the overview is that a person who looks in the VHDL source code can understand where to find the different parts and how they interact. Furthermore, I want a user, who wants to interface the base station with his own

software, to know how to design the network packages send to the base station and how to decode the responses from the base station. It is important to understand that this overview will not replace the original source. Thus in the following description many details will be missing and to keep it simple, the shown finite state machine diagrams will only explain the idea behind the modules.

For developing the firmware, I used the Xilinx ISE design software and its tools.

2.1 Structure of the firmware

Amoung building the hardware for the basestation, a lot of time went into writing the firmware for the FPGA on the ZestET1 board, which is the heart of the base station. The information about the control sequences and package structure concerning the ASIC were provided by Jonas Pistor and Janpeter Hoeffmann (both ITEM, University of Bremen). For the software side, I developed together with Norbert Hauser (Brain Products) the network package structure.

2.1.1 Orange Tree - Top level module

The top level of the firmware is based on example '2' of the documentation which is delivered with the ZestET1 board. This example illustrates a finite state machine, that services one defined TCP network port. It accepts ingoing data stream from the hardware gigabit Ethernet network stack of the ZestET1 and drops it without further processing. And it supplies a counter which, simultaneously to processing the ingoing data, produces a test output which is sent out via the Ethernet network stack to an external PC.

Based on this example, I created a modified version where instead of dropping the incoming data and generating test data for the outgoing data, I connected these data pathways to one incoming and one outgoing 16 bit FIFO. Now it is possible to communicate via a TCP/IP network connection simply by servicing these two FIFOs.

The rest of our firmware is encapsulated in the 'packet fronted' module, which is our real top level module for the base station. Using a digital clock manager on the FPGA, I reduced the 125MHz clock of the Ethernet clock to 95.8333 MHz, which I passed to the 'packet frontend' module.

2.1.2 'Packet Frontend' - Our top level module

Within the 'packet frontend' module all the functionality of the firmware is encapsulated. If required, this allows to transfer the base station firmware easily to another

FPGA hardware platform. On the new system it would just be necessary to service one 16 bit input FIFO and one 16 bit output FIFO as well as to provide a 95.833 MHz clock signal and to pipe the other in / output port through to the FPGA pins. This design decision was made to ensure portability.

Figure 2.1 visualizes how the different sub-modules of the 'packet frontend' module interact with each other. In the following these sub-modules will be discussed in more detail. The firmware contains different groups of sub-modules.

The first group is all about the different defined network commands which the firmware is able to process. It also processes the incoming network commands.

The second group is concerned with interchanging data with the wireless RF transceiver.

Group number three handles the data coming from the implant. Its job is to unpack the incoming data stream from the implant in real time and send it out to the external PC via network.

Group four was designed to manage the hardware for the wire-bound electro-physiological data acquisition.

The last group is servicing the incoming and outgoing trigger channels allowing other external devices to synchronize or the be synchronize their activities with the base station.

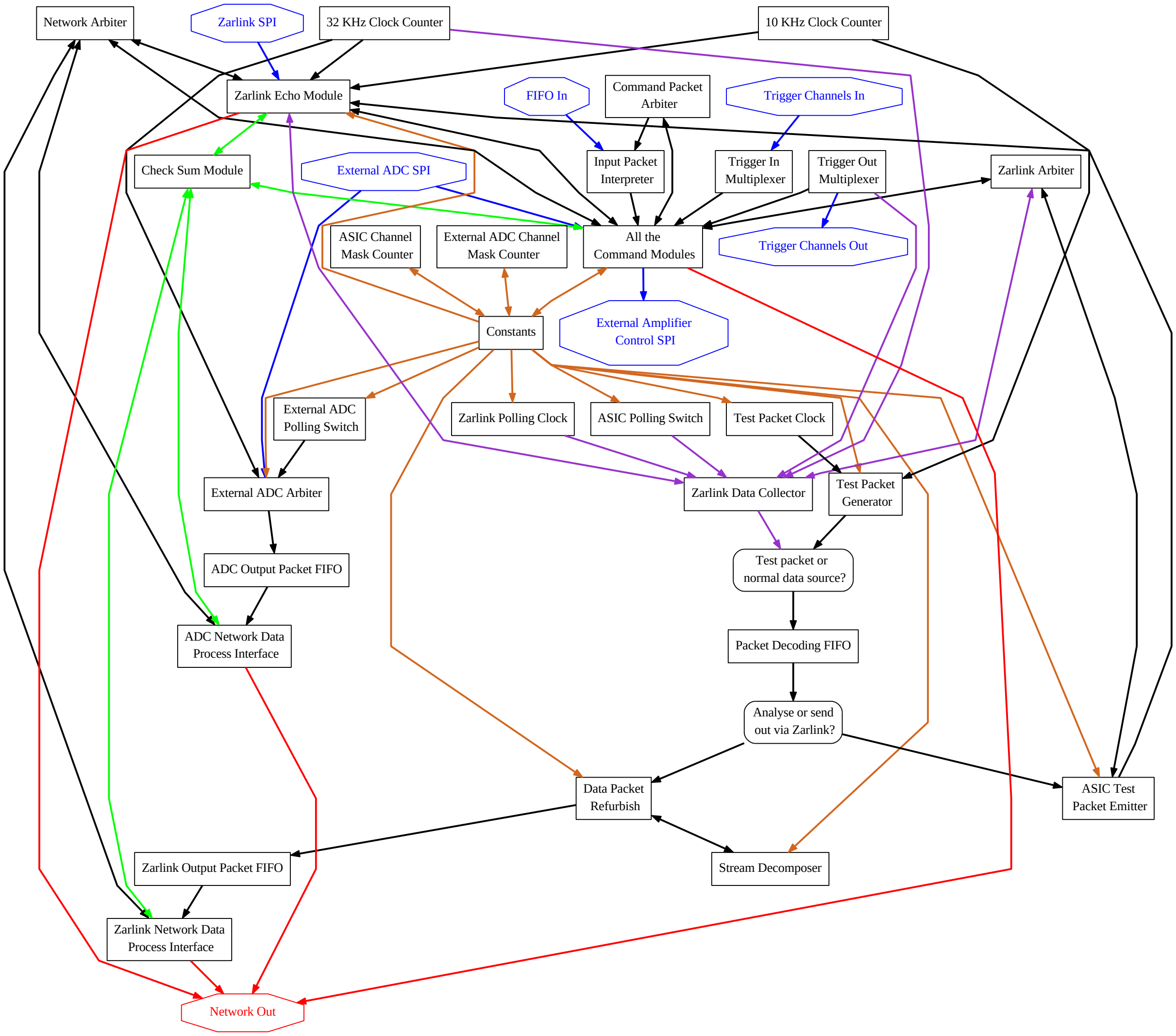

Figure 2.1: Overview of the sub-modules combined in the 'Packet Frontend' module as well as their interconnections.

2.1.3 Network commands modules - Protocol

Together with Norbert Hauser from the project partner Brain Products, I developed the communication protocol between the PC and the Orange Tree Zest ET1 FPGA board. The firmware supports 24 commands (for an overview see 2.2) allowing to perform a variety of tasks. In the following, we will discuss these commands in more detail and then give a description about how these commands are realized in firmware. This allows software designers to write their own software for interfacing the base station.

An important design decision was that the number of commands can change over the development. Thus the number of commands was assumed to be variable as well as that it was necessary to the split the modules such that their common complex parts can be used by all the modules. Furthermore, for increasing the development speed, I wrote a so called 'generalized command' template. This template was the base for most of the network commands within the firmware. This generic module has already the ability to communicate with the Zarlink IC and with the network FIFO. Only the specific functionality for the new network command needs to be added to this template. This procedure also reduced the necessary time for debugging these type of modules and to write a test-bench template too.

General packet structures

0x55AA	Length of the whole package (unit16)	[PAY LOAD]	Check sum

Structure of all packages send via network communication.

Every network command which is send from the PC to the FPGA begins with a fixed synchronisation value (0x55AA), followed by the length of the whole command packet (unsigned integer with 16 bits). Then the pay load is transmitted. The packet ends with a 8 byte long check sum. All integers are in big endian format.

The firmware of the FPGA can respond to requests with three different types of packages. Acknowledgement package, Zarlink debug package and data package. The basic structure (sync value, length, pay load and CRC) is the same as for all the command packages. Regarding the pay load there are differences.

Acknowledgement package: 0x8000 & internal process ID (uint16) & error code (uint16) & data (n x uint16, n can be zero)

Zarlink debug package: 0x8002 & internal process ID (uint16) & error code (uint16) & copy of the data stream (n x uint16)

Data package (ASIC): 0x8001 & time stamp ASIC (10kHz clock) (uint16) & time stamp

Packet - Types

**Unknown Packet**<br>Detects unknown packs and sends a message back to the PC.	**Set Constants** (0x00FF)<br>Allows to configure 23 constants which are used in the firmware.	**Get Version and Status** (0x0101)<br>The user can request information about the version and status of the firmware.
**Open RF** (0x0102)<br>Opens the RF connection via the Zarlink ZL70102 to the implant.	**Close RF** (0x0103)<br>Closes the RF connection to the implant.	**Set ASIC Channel Mask** (0x0263)<br>Sets the channel selection mask of the ASIC via the RF connection.
**Set ASIC AD Resolution** (0x0272)<br>Sets the number of bits for one sample in the ASIC (1-16 bit).	**Set ASIC Samplerate** (0x0273)<br>Sets the number of samples per second (n/10KHz) in the ASIC.	**Set ASIC Filter Parameters** (0x0266)<br>Configures the high- and low-pass setting of the ASIC.
**Set 2. Channel Mask** (0x027A)<br>Sets the secondary channel mask for an external recording system.	**Start ASCI Data Acquisition** (0x0301)<br>Starts the data acquisition process in the ASIC.	**Stops ASIC Data Acquisition** (0x0302)<br>Stops the data acquisition process in the ASIC.
**Read Zarlink Register P0** (0x0400)<br>Reads a Zarlink ZL70102 register from memory page 0.	**Read Zarlink Register P1** (0x0401)<br>Reads a Zarlink ZL70102 register from memory page 1.	**Write Zarlink Register P0** (0x0402)<br>Writes into a Zarlink ZL70102 register from memory page 0.
**Write Zarlink Register P1** (0x0403)<br>Writes into a Zarlink ZL70102 register from memory page 1.	**Write Zarlink Data Block** (0x0404)<br>Writes a block of 14 bytes to the Zarlink ZL70102 for RF transmission.	**Read Zarlink Data Block** (0x0405)<br>Reads a block of 14 bytes from the Zarlink ZL70102 via RF transmission.
**Set Trigger Channels** (0x0500)<br>Sets the trigger channels of the basestation.	**Set Amplification** (0x0279)<br>Controls the amplifier of the external recording system.	**Selected ASCI Channels** (0x0279)<br>Reports the selected channels of the ASIC and delivers the order found in the data.
**Start Data Acquisition** (0x0303)<br>Starts the data acquisition process of the external recording system.	**Stop Data Acquisition** (0x0304)<br>Stops the data acquisition process of the external recording system.	**Get Input Trigger Channels** (0x0503)<br>Reads out the status of the input trigger channels of the base station.

Commands for the ASIC
Commands for the external recording system
Commands for the Zarlink ZL70102
Commands for the trigger channels

Figure 2.2: List of all commands that can be send from the PC to the FPGA.

base station (32kHz clock) (uint16) & first 16 trigger-in channel (uint16) & recorded data (16 bit samples, n x uint16) & status bit (uint16)

Data package (base station ADC): 0x8003 & time stamp base station (32kHz clock) (uint16) & first 16 trigger-in channel (uint16) & 32 bit counter & recorded data (32 bit samples, n x 2 x uint16) & status bit (uint16)

If no error occurred, then the error code is zero. Otherwise a error code is delivered which allows to locate the problem in the VHDL source code. Since there are very many different error codes (in the order of magnitude of 100), it makes no sense to list them in this document.

For an acknowledgement package, the pay load begins with an ID value (0x8000, unsigned integer 16 bits). This is followed by a copy of the command packet ID allowing us to check which of the sent command packets were received and processed. In the case of an unknown command IDs, these are copied into the acknowledgement package too. The last mandatory part of the packet is an error code describing what went wrong if some thing went wrong. An error value of 0 represents the non-error case. Finally, there can be an optional return value in multiples of 2 bytes length.

Implemented are: The version of the firmware in the case of a firmware information request (high-byte = major firmware version, low-byte = minor firmware version), the answer to a Zarlink 70102 register readout (2 byte with bit[7-0] = register value), the result of a 14 byte Zarlink block read command, the status of the 'data repacking unit' (ID is 0x00FE) as well as the list of the activated channels on the ASIC (command ID 0x0502). In the last case, the FPGA sends to the PC a set of value tuples with three unsigned integer 16 bit values (ASIC channel number, RHA chip number and RHA channel number). This set has a length equal to the number of activated channels. This list is important for the PC to identify the order of channels in the data stream from the FPGA.

The data in an acknowledgement package can be:

Version information after command 0x0101: high-byte = major firmware version, low-byte = minor firmware version (uint16)

Zarlink register read out after command 0x0400 or 0x0401: content from the requested register (Bit[7-0] of an uint16)

Zarlink 14 byte data block after command 0x0405: 14 bytes of data from the RF link (7 x uint16)

Data repacking unit (internal process ID = 0x00FE) after command 0x0301: status information ($\mathfrak{n}$ x uint16)

Channel information after command 0x0502: $\mathfrak{n}$ x 3 x 16 bit unsigned integer with $\mathfrak{n}$ as the number of selected ASIC channels ($\mathfrak{n}$ x tuples of channel number (uint16) & RHA chip ID (uint16) & RHA input channel ID (uint16))

If there is a problem with establishing the RF connection or the data transfer via the RF link, it is possible to enable a Zarlink debug mode in the firmware. This will create a copy of the whole data transfer between the Zarlink IC and the FPGA. This copy will send as a Zarlink debug package to the external PC. The pay load of such

a debug package is: First a constant ID (0x8002) and a ID (unsigned integer 16 bits) of the part of the firmware which was responsible for the data transfer. This allows to identify which module of the firmware tried to interact with the Zarlink IC. Then an error code (unsigned integer 16 bits) is send where 0 shows that there was no error in communicating with the RF IC via SPI. Finally the pay load ends with a copy of the data stream between the Zarlink and the FPGA.

During data acquisition, the FPGA transfers the recorded data into data packages and sends them to the external PC. There are two versions of data package types: The version with the ID 0x8001 from acquiring data from the ASIC and packages with the ID 0x8003 from the cable bound data recording system.

In the case of the ASIC data package, after the ID follows a time stamp from inside the ASIC (unsigned integer 16 bits). This time stamp is created by a 10kHz counter inside the ASIC, allowing to check for the timing problems concerned with the ASIC, its data acquisition process and the RF link. Then a second time stamp, produced by a 32kHz in the base station firmware, is included in the pay load. This second time stamp allows to check how the timing (data collection from the RF link and data processing within the FPGA) develops over time. After the time stamps, the binary states of the first 16 trigger input channels, in the moment when the data entered the FPGA via the RF link, is included as a 16 bit string. Next the measured data from the ASIC can be found in the pay load. The measured data is composed out of a list of 16 bit unsigned integers. The number of these values corresponds to the number of selected channels in the ASIC. As the last 16 bit unsigned integer value of the pay load, we get status information signalling whether the data within this data package is valid (= 1) or not.

Except the ID, there are only three other differences between the packages with the data from the ASIC compared to cable based recording system. Each data sample in the case of the ASIC is 16 bit, where the data from the other recording system is 32 bits, due to the applied 24 bit analogue digital converter. Second there is no 10KHz counter and before the measured data there is one 32 bit counter, counting up with each package of acquired data.

In the following the different network commands are discussed in more detail. The settings were selected such that it is not necessary to intervene for a normal mode of operation.

Command 1. - Set debug constants

ID 0x00FF allows to adjust parameters within the firmware. These kind of network package contain 64 bit of information. It consists of an ID (unsigned integer with 16 bits) identifying the constant which needs to be changed. The ID is followed by three unsigned integer values with 16 bits each.

Input: 0x00FF & 64 bit pay load (consists of constant ID (uint16) & Value for the constant (3x uint16))

Constant ID 1 : Zarlink debug mode
If Bit[16] is 1 then the firmware starts to create Zarlink debug package and sends them to the external PC. These package contain the complete data transfer between the firmware and the Zarlink RF transceiver IC. In the case of problems with connecting and controlling the Zarlink IC as well as in the case where the ASIC produces defect packages, this mode allows a very detailed debugging of the incoming and outgoing data stream of the base station FPGA. When Bit[16] is set to 0, then Zarlink debug mode is disabled.

Constant ID 2: Zarlink block operation timeout
Before a 14 byte data block operation with the Zarlink IC can be performed, the firmware checks if the tx- buffer is too full for writing into it or if the rx- buffer is too empty for reading from it. If the actual status of the buffer shows that the requested operation is not possible in the moment, then the firmware waits and tries again. The value (Bit[43-16]) which is given to the firmware defines the number of clock cycles (referenced to a 95.833MHz clock) which the firmware waits between two buffer status checks. Default is 1 sec.

Constant ID 3: Zarlink connection timeout
There are several reasons why a connection between the Zarlink base station module and the Zarlink implant module may fail. For regaining control of the base station firmware and its network stack when a RF link can not be established, it is necessary to limit the time in which the base station waits for the connection to be completed. The default for this time interval is set to 5 min. This debug constant allows to change this time interval (Bit[43-16]). The debug command sets a 48 bit unsigned integer as limit on the number of clock cycles (referenced to a 95.833MHz clock).

Constant ID 4: Zarlink wait for reset
During the complex process which establishes the connection between the Zarlink base station module and the Zarlink implant module, it is necessary to reset the Zarlink IC. For some time after the reset, the IC ignores all incoming commands. Thus it is important that the FPGA does not send any information to the Zarlink IC in that time window. This debug constant configures this time interval using a 28 bit unsigned integer containing the number of clock cycles (referenced to a 95.833MHz clock) which the firmware waits until it starts to communicate with the Zarlink IC again. Default is 0.5 sec.

Constant ID 5: Implant device ID
During the communication with the implant's ASIC a device ID is used. Normally this device ID is hard coded into the ASIC design but for test purposes it is possible to change the implant device ID on the base station firmware. The device ID is 8 bits long (Bit[23-16]). Default is 53.

Constant ID 6: Zarlink check buffer fill status
In the ASIC data acquisition mode, the base station checks (in idle mode) approximately every 13450 clock cycles (referenced to a 95.833MHz clock) the rx- buffer status of the base station's Zarlink and collects the data from its rx- buffer. With this debug constant it is possible to change this period defined by a 20 bit unsigned integer value (Bit[35-16]).

Constant ID 7: Zarlink clean up counter
After stopping the ASIC data acquisition process, the firmware cleans the rx- buffer of the Zarlink base station IC. But between stopping and cleaning, the firmware waits for some time. It is helpful to wait because this allows the tx- buffer of the Zarlink implant IC to be emptied into the rx- buffer of the Zarlink base station IC. This time interval is defined by a 19 bit unsigned integer value (Bit[34-16]) which represents the number of clock cycles (referenced to a 95.833MHz clock) to be waited for. Default is 250000;

Constant ID 8: Test packet generator
For testing the data processing on the FPGA as well as allowing an easy way of testing the PC base station software, it is possible to convert the firmware into a test packet generator. If Bit[16] is set to 1 the firmware starts to produce test packets in a way and in speed similar to what the normal implant delivers. Bit[16] is set to 0, the firmware stops producing these test packets.

Constant ID 9: Test packet redirection to Zarlink IC
In combination with debug constant 8 and 10, it is possible to convert the base station into a test platform emulating an implant. Setting Bit[16] to 1 reconfigures the firmware such that, instead of analysing and processing the (test) packages, the (test) packages are directly send out via the Zarlink RF link. Setting Bit[16] back to 0, disables this redirection and feeds the packages back into the analysis data path.

Constant ID 10: Zarlink implant mode
In the case that two base station are available, it is possible to use one of them to emulate an implant for test purposes. Using this test mode makes it necessary to replace the Zarlink base station hardware module with a Zarlink implant hardware module. If Bit[16] is 1 then the firmware uses the Zarlink implant connection protocol for establishing the RF link and if Bit[16] is 0 then is uses the normal Zarlink base station connection protocol.

Constant ID 11: Wait until next retry in Zarlink communication
Checking the rx- buffer fill status of the Zarlink base station IC may result in the information that the buffer is empty. To keep the RF link from a buffer overrun, it is necessary to repeat the check about the buffer status very soon. On the other hand it is important that this check isn't done too soon because this interferes with the Zarlink IC from getting data via the RF link. This debug constant allows to set the number of clock cycle which the FPGA waits until it checks the buffer again. This time period is defined by a 19 bit unsigned integer value (Bit[35-16]). Default is 3000 clock cycles.

Constant ID 12: Wait until next Zarlink interaction
Tests showed that it is necessary to allow the Zarlink IC a cool down time after the every SPI interaction. This debug constant regulates this time interval with a 8 bit unsigned integer (Bit[24-16]). Default is 30 clock cycles (referenced to a 95.833MHz clock).

Constant ID 13: Data processing dump mode
Bit[16] = 1 enables a data processing dump mode. Instead of analysing and processing the incoming packages and reshaping them into outgoing network data packages, the RAW data is directly sent to the network interface. The FPGA appends additional information about the status of the firmware (see figure 2.3, 2.4 and 2.5 for details. Ignore the extra '0' or '1' in the FIFO-out output for the expected response.). Setting this bit to 0 disables this mode and switches the normal package processing on again.

Constant ID 14: Zarlink connection mode
Bit[19-16] define the mode of RF connection or more precise: its encoding schema via the Zarlink MAC Moduser register. Default is 4FSK ('1111') for both directions (FSK means frequency shift keying). Possible are '00' for 2FSK fall back (200kbit/sec), '10' for 2FSK (400kbit/sec) and '11' for 4FSK (800kbit/sec). The bits[17-16] are for the transmission modulation and the bits[19-18] are for the receiver modulation.

Constant ID 15: Zarlink link quality
Bit[16] = 1 enables a special debug mode, that reports the number of error detected and error corrected blocks as well as the number of total blocks. Default is off. Using this mode can produce problem with the active RF link because the additional SPI traffic disturbs the RF transmission.

Constant ID 16: Test packet emitter retry time
Number of clock cycles (Bit[47-16]) which the 'Test Packet Emitter' module waits between two Zarlink TX buffer checks (referenced to a 95.833MHz clock). Default is 300000.

Constant ID 17: Definition of a 1/10 ms for the test packet generator
Bit[45-16] tells the 'Test Packet Generator' module how long a 1/10 of a millisecond in clock cycles is (referenced to a 95.833MHz clock). Default is 9583. This value allows to control the number of test packet generated per second.

Constant ID 18: Zarlink maximum buffer fill
Bit[23-16] defines the number of Zarlink data blocks that can be written by the test packet generator (as maximum) before the status of the TX buffer is checked again. Default is 60.

Constant ID 19: Half cycle length for the trigger-out
Bit[32-16] allow to adjust the waveform length of the bits send to the multiplexer hardware for the trigger-out channels. Default is 100, which corresponds to approximately to 500kHz.

Constant ID 20: Wait for signal settling for trigger-in
Bit[32-16] sets the time for the signals of the trigger-in channels, allowing them to settle before reading them out. Default = 200.

Constant ID 21: Zarlink length of the SPI signals
During tests we found that the Zarlink SPI communication should be done with the lowest speed possible because too fast changes in the signal cause perturbations in the RF transmission. Bit[23-16] allows it to change the waveform length of the bits. The length can be calculated by $(n + 1) * 2$ clock cycles of a 95.833MHz clock. Default is 30.

Constant ID 22: Zarlink SPI speed flag
Bit[17-16] sets the SPI speed flag during the connection initialization procedure of the ZL70102 IC (register 43). Default is '01'. '00' sets a maximal SPI speed of 1MHz, '01' a maximum of 2MHz and '11'/'10' a maximum of 4MHz.

Constant ID 23: Zarlink max packet size
With Bit[23-16], the maximal packet size for the Zarlink connection process (register 5) is defined. Default is 31 and also the maximal selectable value.

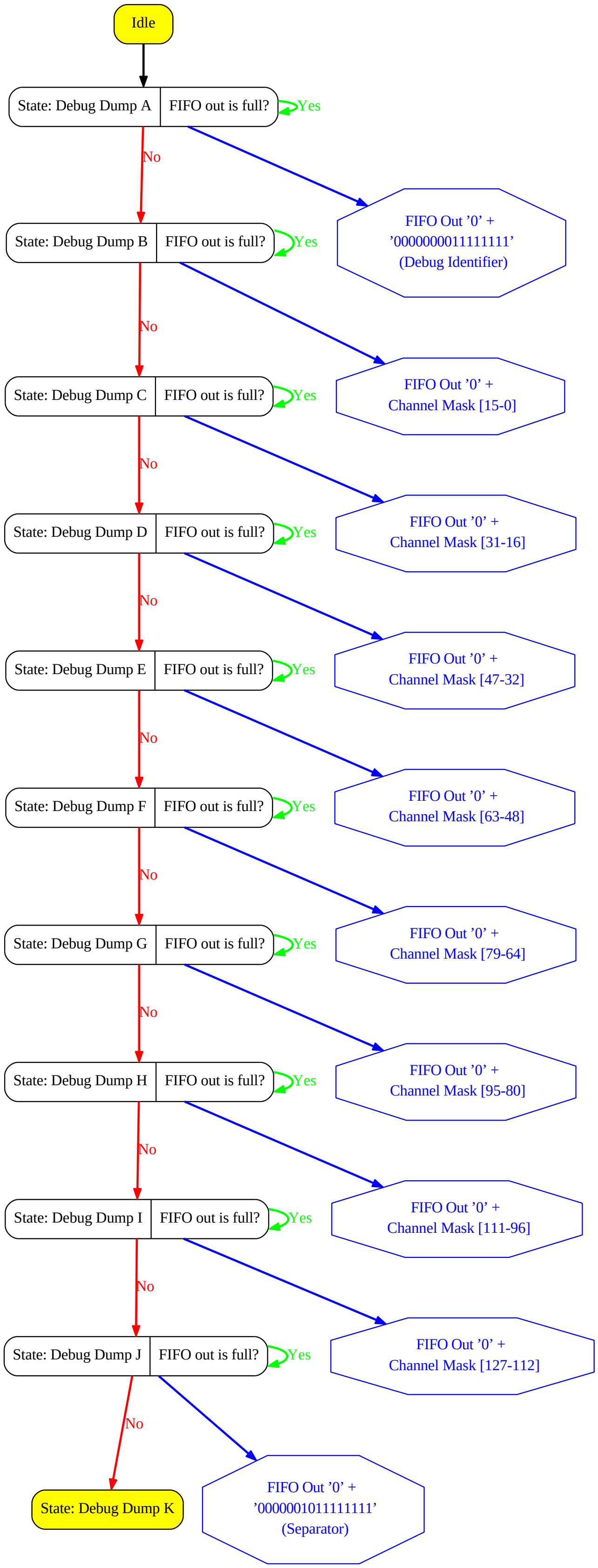

Figure 2.3: Debugging part of the 'Data Packet Refurbish' module, which is enabled by debug constant ID 13. (1 of 3)

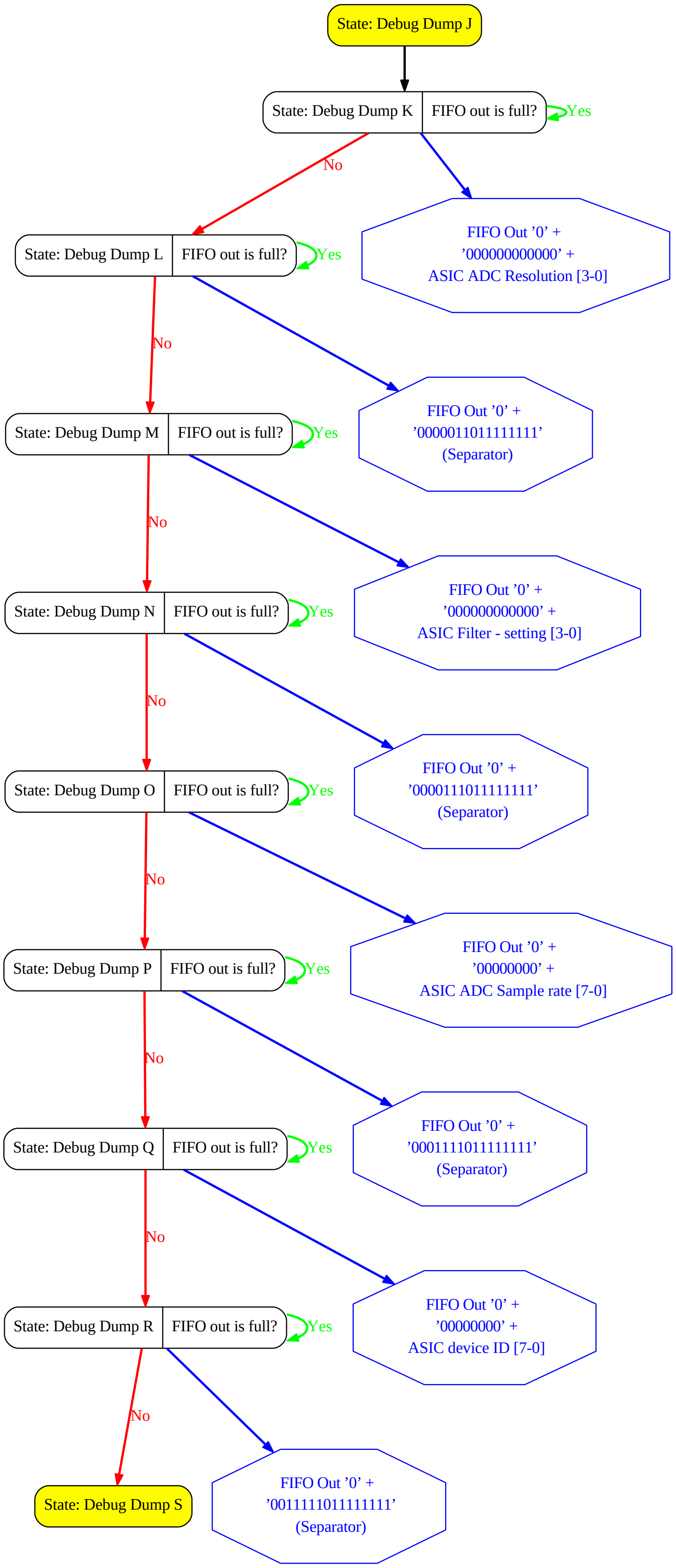

Figure 2.4: Debugging part of the 'Data Packet Refurbish' module, which is enabled by debug constant ID 13. (2 of 3)

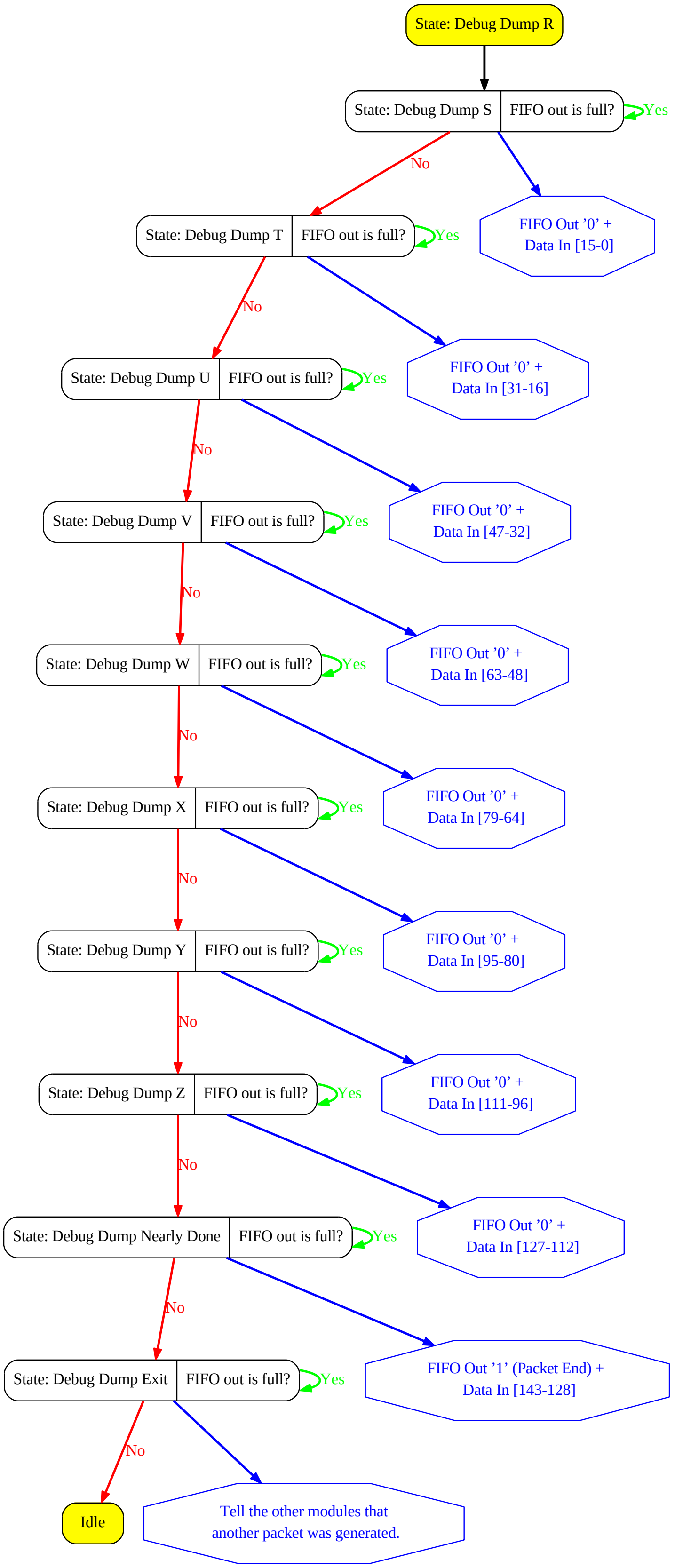

Figure 2.5: Debugging part of the 'Data Packet Refurbish' module, which is enabled by debug constant ID 13. (3 of 3)

Command 2. - Request version and status information

ID 0x0101 request an acknowledgement package containing information about the firmware version. No other parameters are transmitted to the FPGA for this command. The firmware version value, we get back from the FPGA, consists out of two components. One component describes the version of the interface (higher byte) and the second component identifies the version of the mechanics behind the interface (lower byte). This allows the software on the external PC to check via the interface version if it is compatible with the running firmware and the other number allows to keep track which processing logic version is at work.

Input: 0x0101

Command 3. - Open RF connection

ID 0x0102 starts the creation of a RF link between the Zarlink base station module and a Zarlink implant module. The Zarlink needs an IMD transceiver device ID for this procedure. The device ID consists out of 3 bytes. Default is 0xAA, 0xAA and 0xAA, which is also hard-coded in the ASIC. This is followed by a byte that will be ignored by the firmware but is required to fill up the usual 16 bit string in which the FGPA handles its data. Normally, the base station uses the RF link opening protocol for the Zarlink base station module but using the debug constant 10 (and changing the Zarlink module) makes it possible to use the Zarlink implant connection procedure.

In the case of the normal base station mode, the FPGA firmware will send an acknowledgement network package reporting whether the connection attempt was a success or not. In the case of the Zarlink implant connection mode, the firmware delivers an acknowledgement network package after the preparations for the connection have been done and does not waits how the connection to be established.

The figures from 2.6 to 2.13 visualize how the connection within the firmware is created. For a successful connection many individual configuration steps are necessary. In these steps are embedded within a module derived from the template shown in figure 2.18, 2.19 and 2.20. The orange-marked states are representing a structure to handle a connection time out which is shown in figure 2.14.

Input: 0x0102 & IMD transceiver ID0 (uint8) & IMD transceiver ID1 (uint8) & IMD transceiver ID2 (uint8) & 0x00 (uint8)

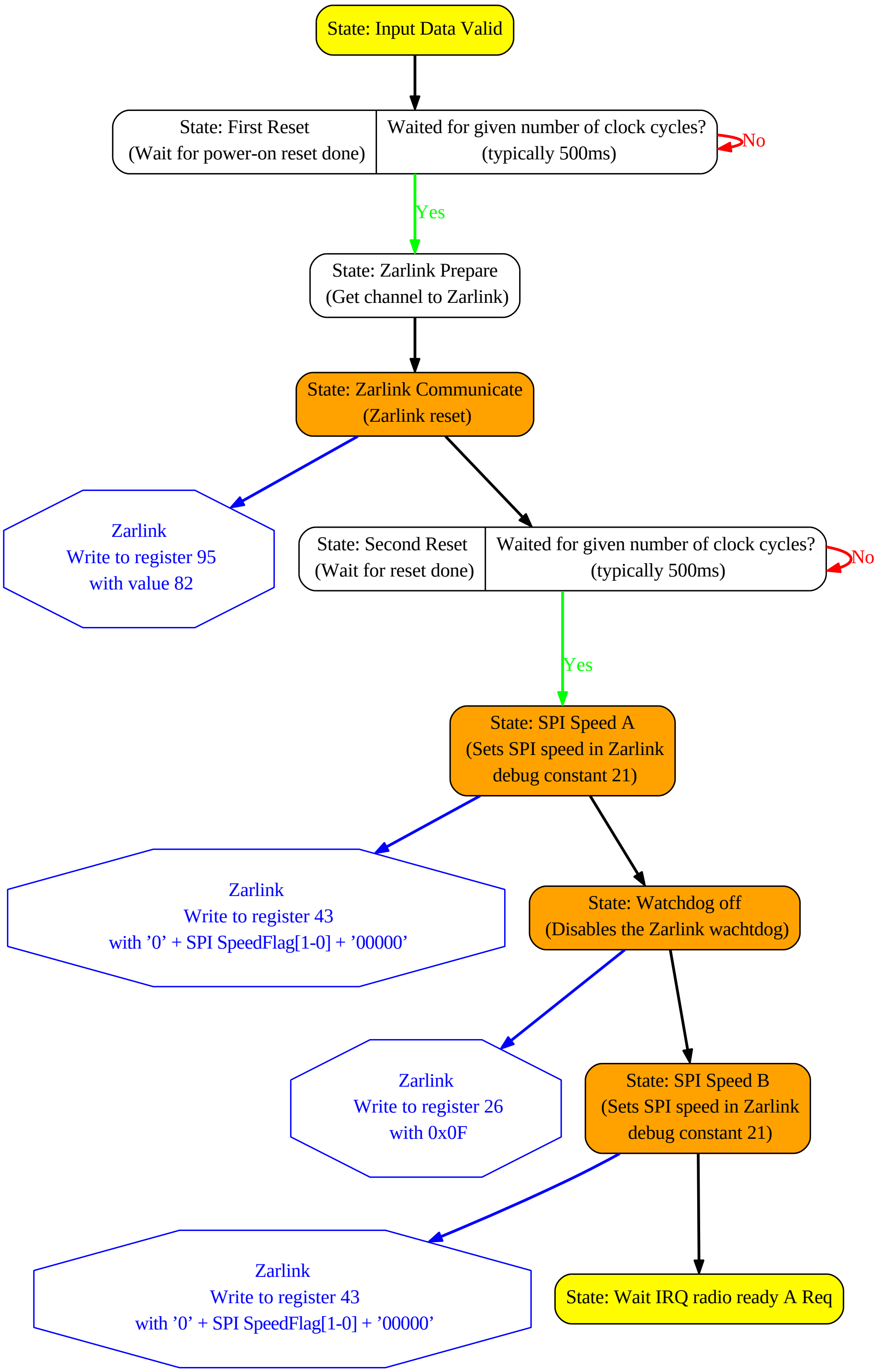

Figure 2.6: Additional part of the 'Zarlink Connection Command' module required for establishing a RF link. (1 of 8)

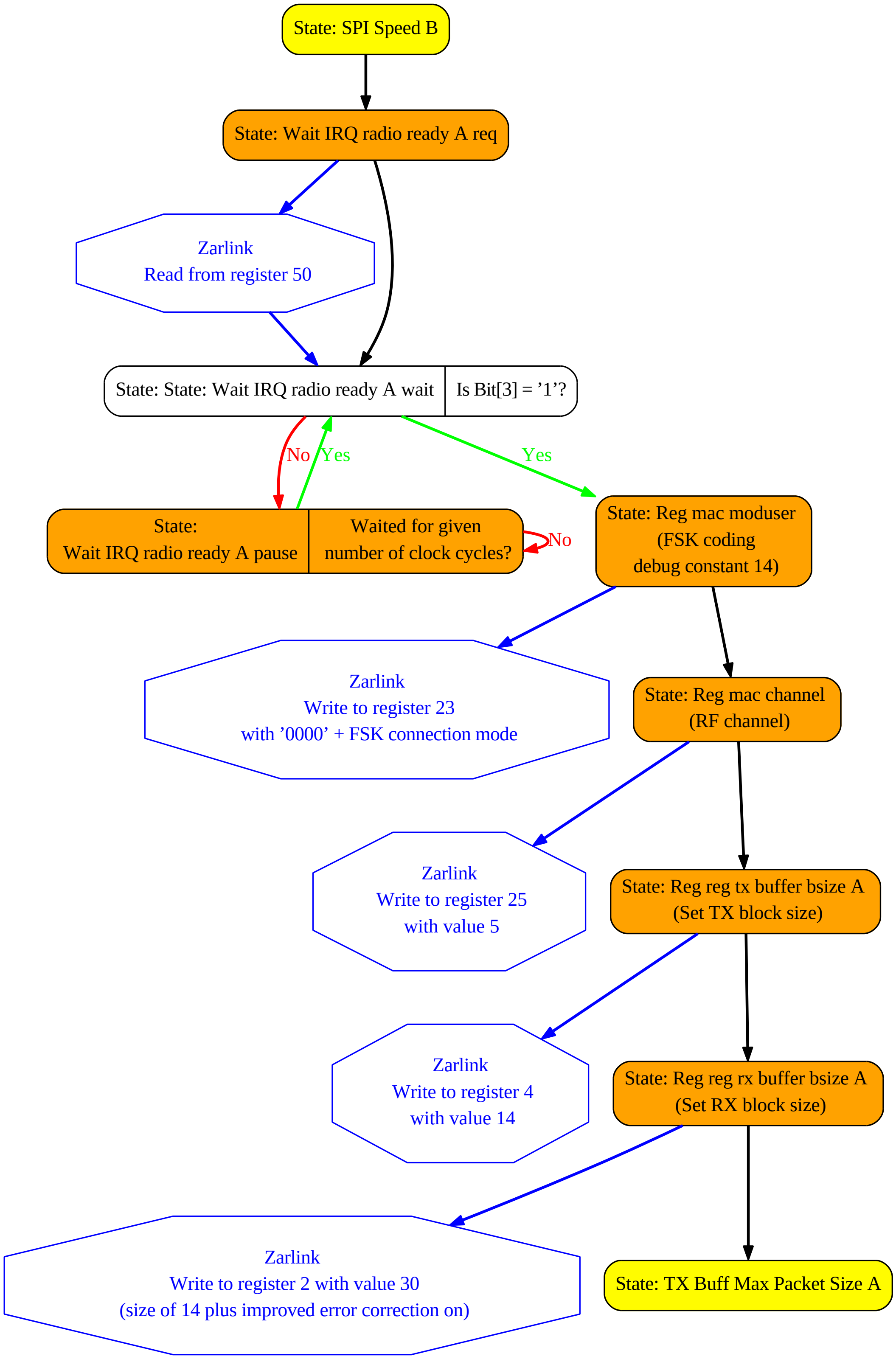

Figure 2.7: Additional part of the ’Zarlink Connection Command’ module required for establishing a RF link. (2 of 8)

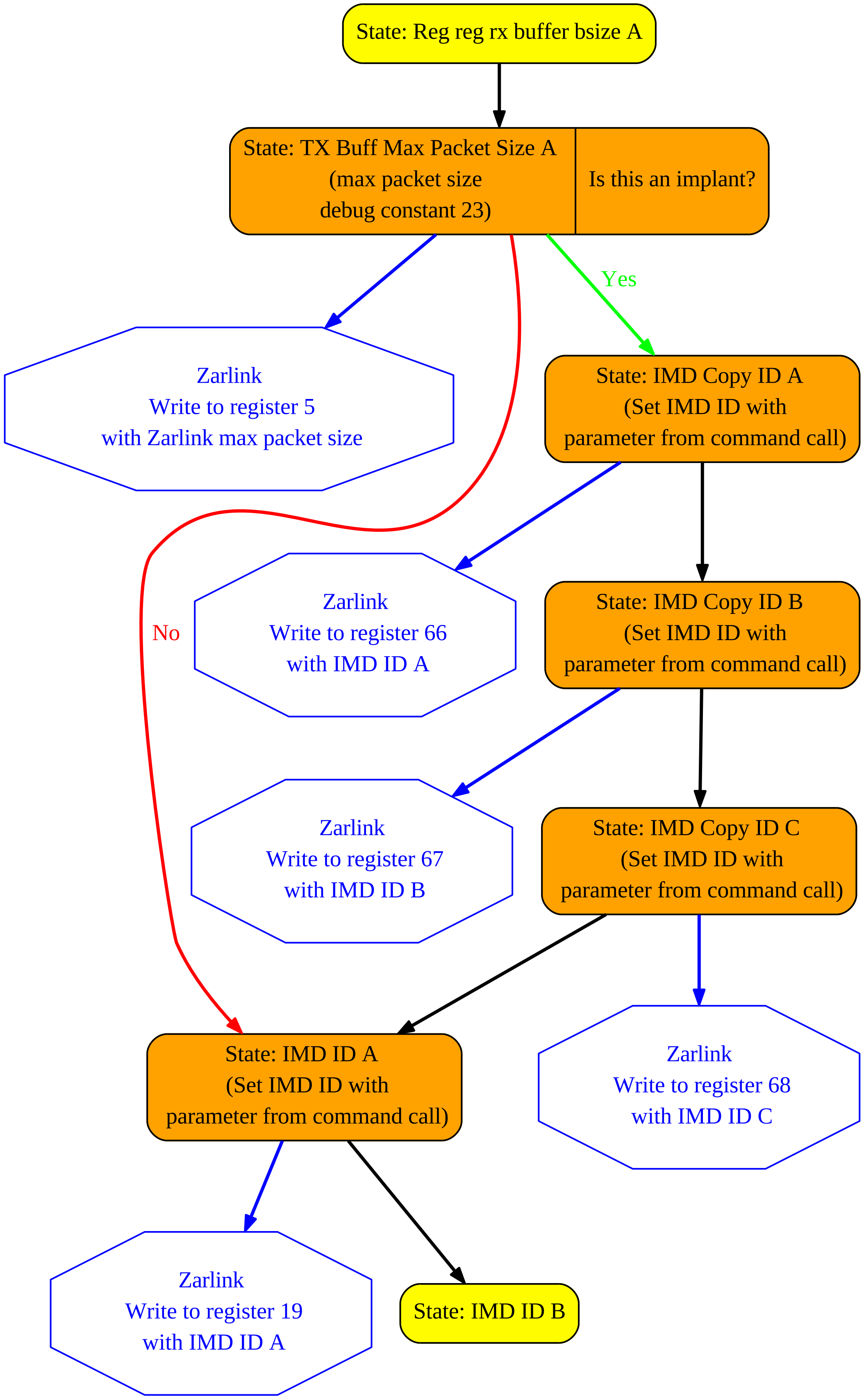

Figure 2.8: Additional part of the 'Zarlink Connection Command' module required for establishing a RF link. (3 of 8)

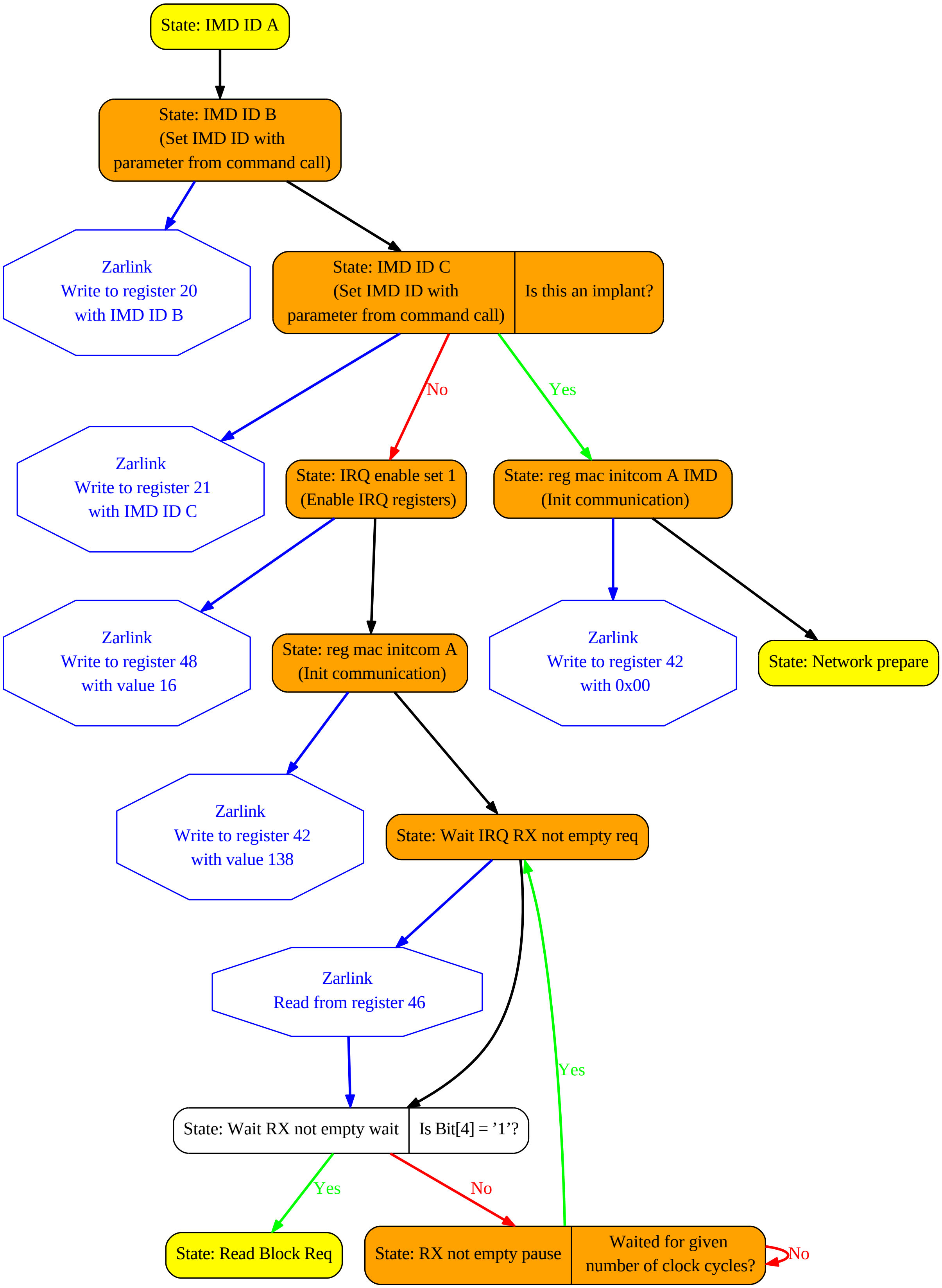

Figure 2.9: Additional part of the 'Zarlink Connection Command' module required for establishing a RF link. (4 of 8)

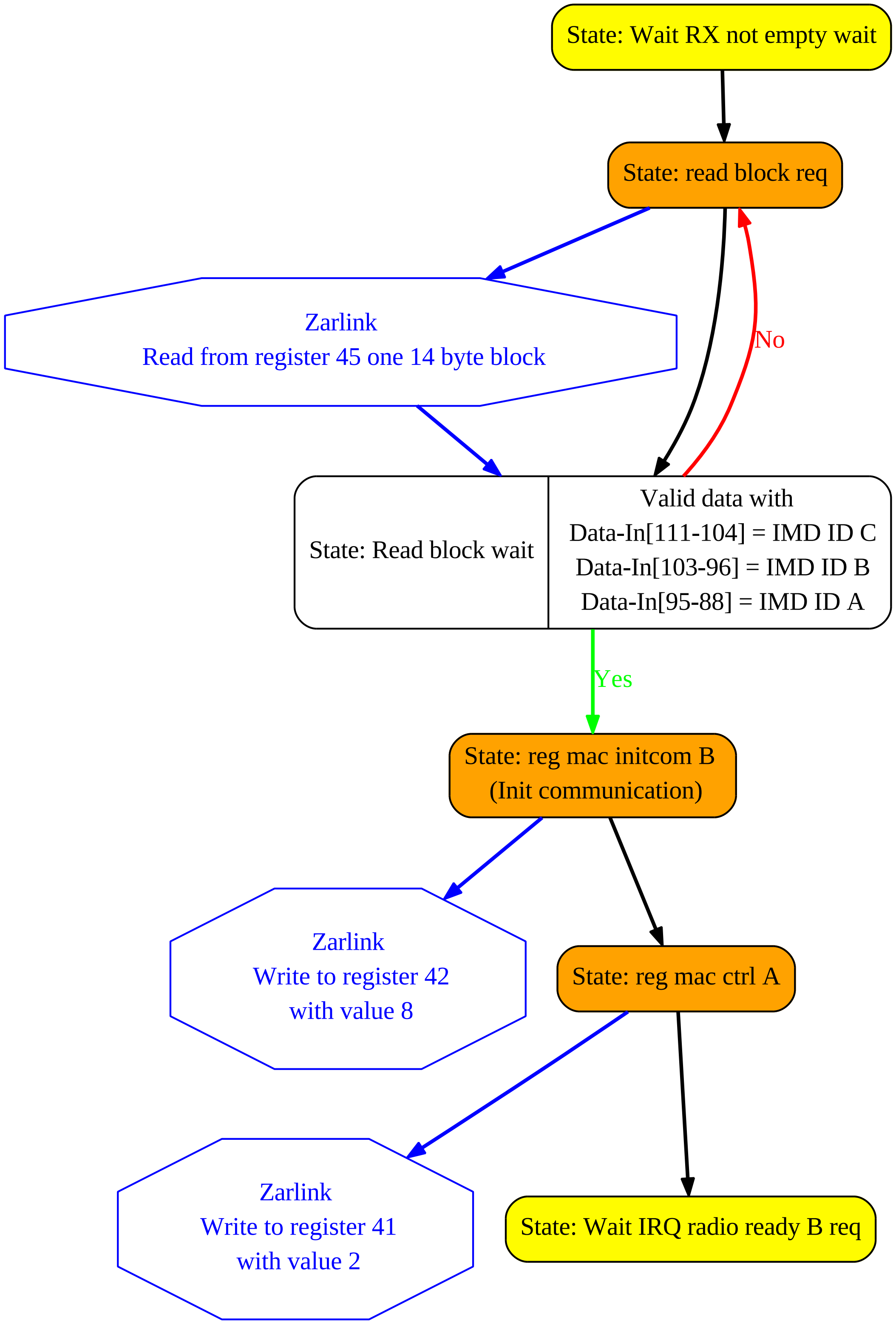

Figure 2.10: Additional part of the 'Zarlink Connection Command' module required for establishing a RF link. (5 of 8)

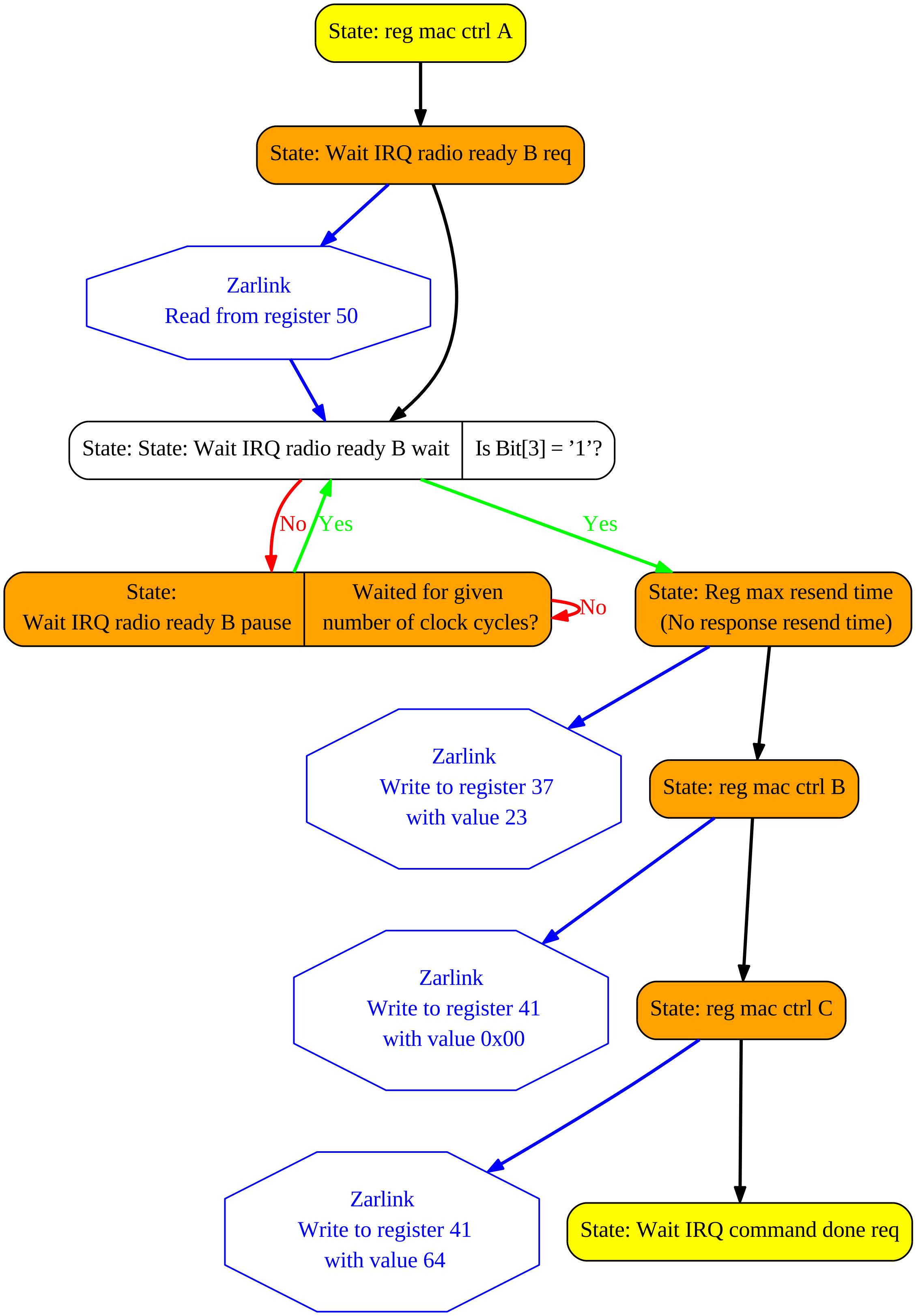

Figure 2.11: Additional part of the 'Zarlink Connection Command' module required for establishing a RF link. (6 of 8)

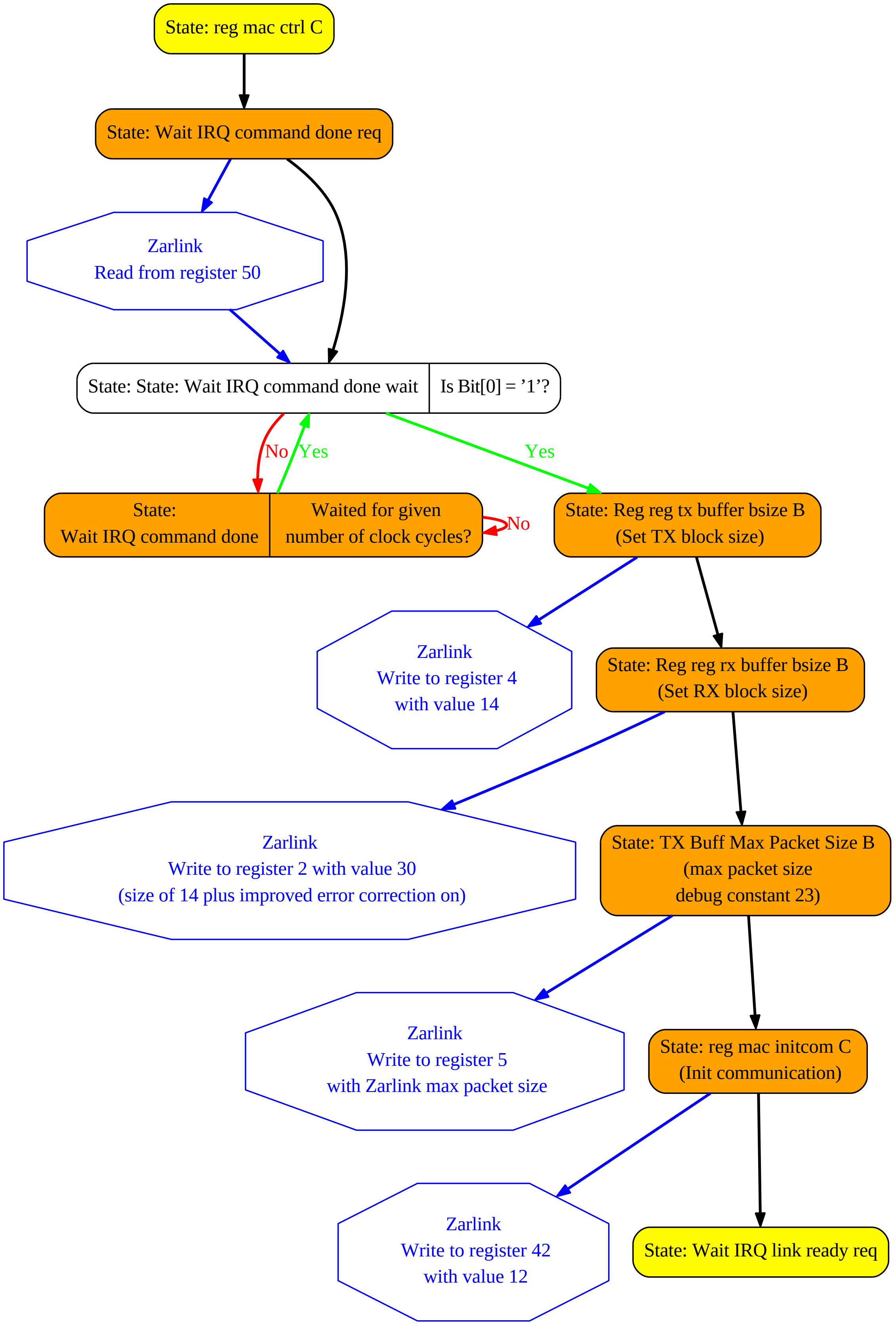

Figure 2.12: Additional part of the 'Zarlink Connection Command' module required for establishing a RF link. (7 of 8)

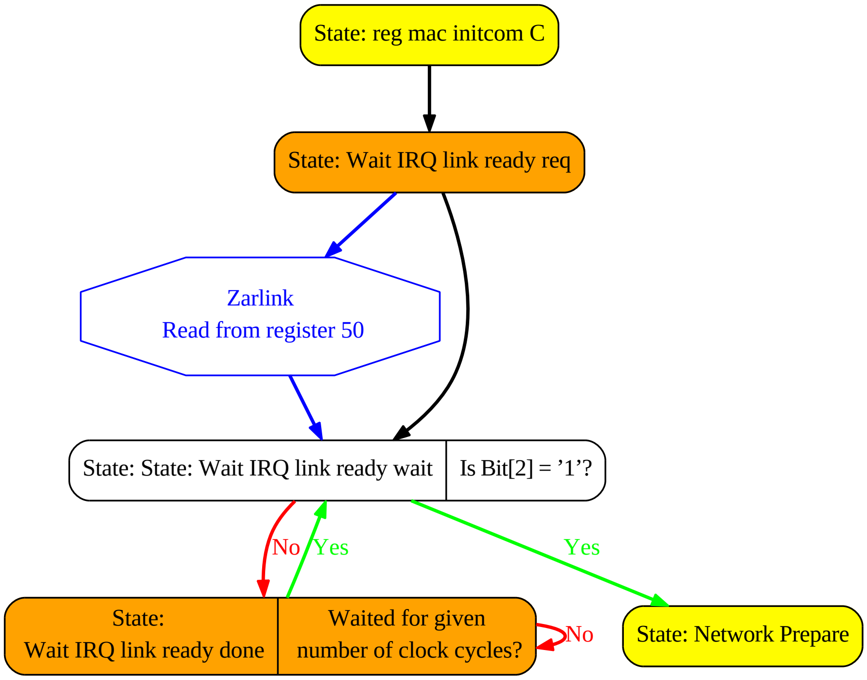

Figure 2.13: Additional part of the 'Zarlink Connection Command' module required for establishing a RF link. (8 of 8)

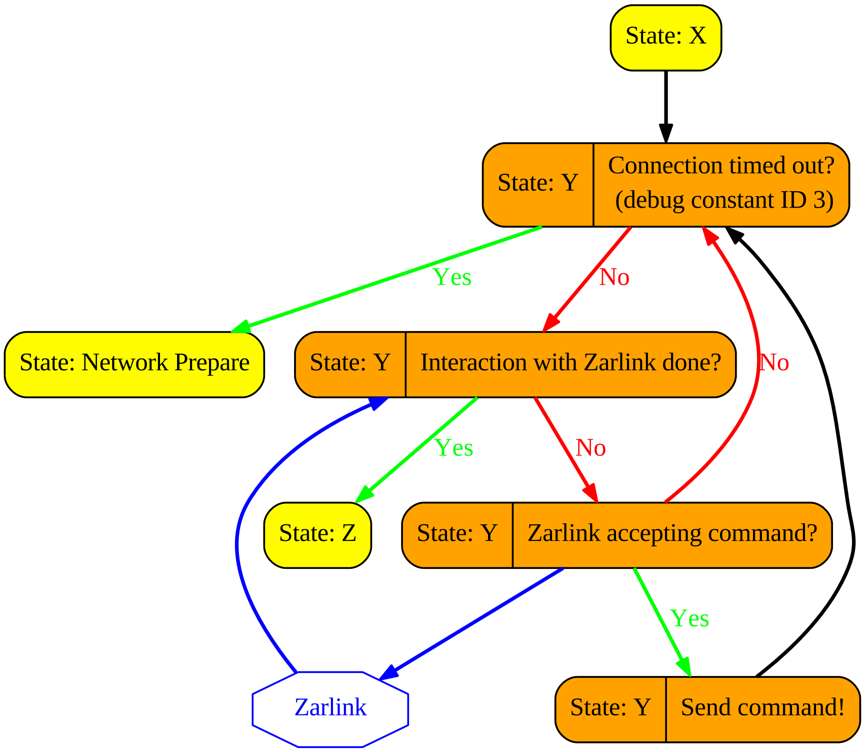

Figure 2.14: For simplifying the 8 prior figures, complex states with time out mechanics were replaced by simplified orange marked states.

Command 4. - Close RF connection

ID 0x0103 closes the RF link between two Zarlink modules. This command package carries no additional information. Furthermore, closing the RF link is not mandatory and thus optional.

Figure 2.15 shows the steps necessary for closing the connection. These states need to be integrated into the template shown in figure 2.18, 2.19 and 2.20 for creating the required module.

Input: 0x0103

Command 5. - Set ASIC channel mask

ID 0x0263 ('c') allows to set the channel mask of the ASIC according a 128 bit long (8x unsigned integer) channel mask. The ASIC uses this information for selecting the requested RHA data acquisition channels from all the available RHA channels. Transmitting this command to the ASIC requires an established Zarlink RF link.

Input: 0x0263 & 128 bit channel mask (8x unit16)

Command 6. - Set ASIC analogue digital converter resolution

ID 0x0272 ('r') changes the analogue digital converter resolution. The ASIC collects from the RHAs recording samples with a resolution of 16 bit. The ASIC is able to cut off the most significant bits for reducing the amount of data which then has to be sent through the very limited RF link. When the requested resolution is $n + 1$ then the bits[3-0] of a 16 bit string have to contain the value n (4 bit unsigned integer).

Input: 0x0272 & n from $n + 1$ bit sample resolution (Bit[3-0] of an uint16)

Command 7. - Set ASIC analogue digital converter frequency

ID 0x0273 ('s') can be used to change the recording frequency. The RHAs on the implant record data with 10000 samples per second and channel. The ASIC has a function allowing to reduce the number of samples per second. Valid frequencies are $n/10\mathrm{kHz}$. The FPGA firmware needs n as an unsigned integer with 16 bits.

Input: 0x0273 & n from $n/10\mathrm{kHz}$ sample frequency (uint16)

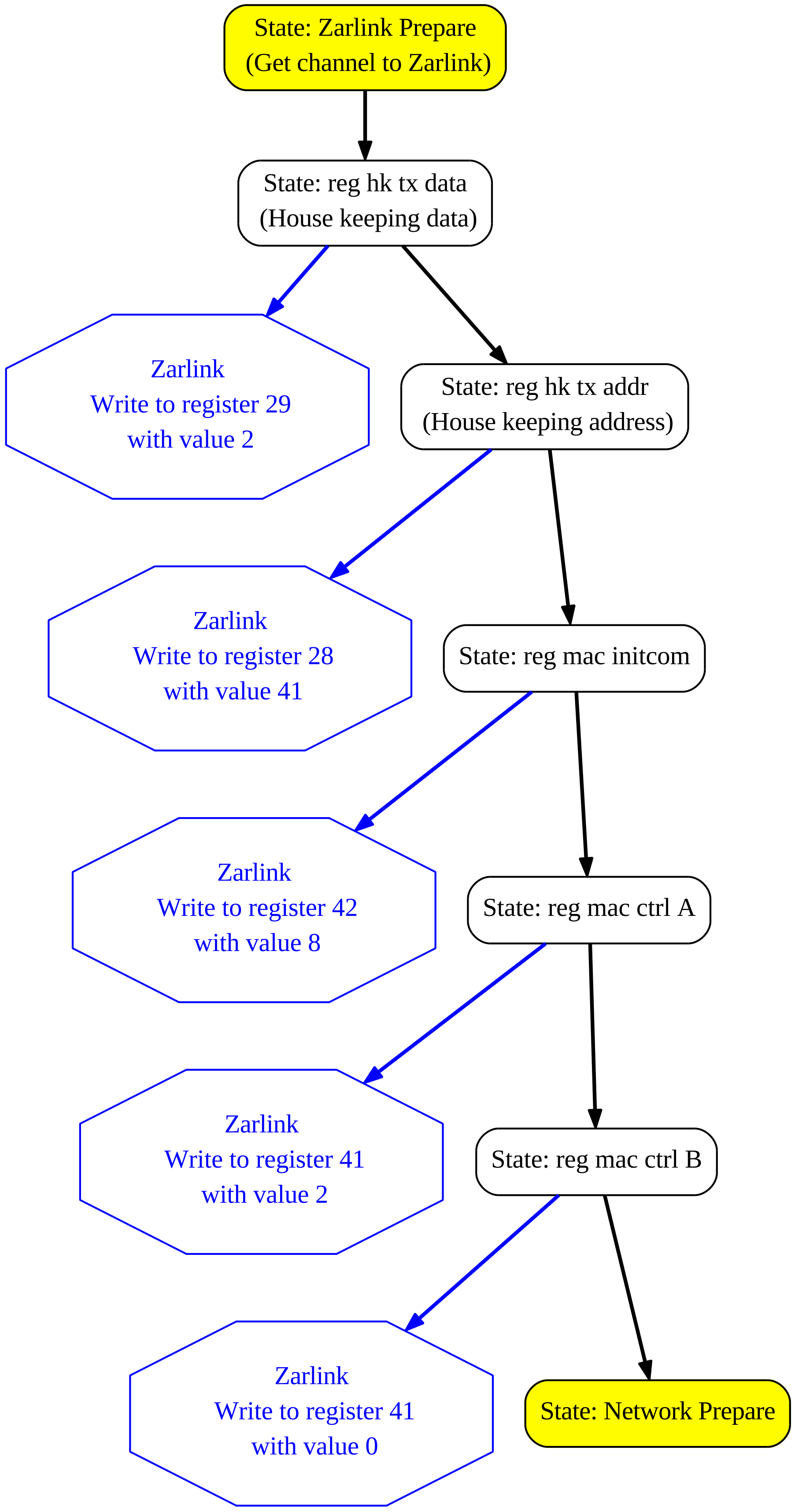

Figure 2.15: Additional part of the 'Zarlink Close RF connection' module.

Command 8. - Set ASIC bandpass filter settings

For development reasons, the ASIC has the capability to control two multiplexers with 4 paths each. These multiplexers can be used to select external components (resistors) for the RHAs. Depending on these resistor values, the low and high cut off frequency of the RHA's analogue bandpass filters are set. ID 0x0266 ('f') is used to configure the setting of the multiplexers. From the 16 bit string send to the firmware, the bits[3-0] are used for the configuration. Two bits for the low cut off frequency and two bits for the high cut off frequency.

Input: 0x0266 & high frequency and low frequency cut off of the analogue RHA bandpass filter (Bit[3-0] of an uint16)

Command 9. - Set base station channel mask

A second source of electro-physiological data for the data acquisition with the base station are classical wire-bond recordings. ID 0x027A ('z') allows to set a 32 bit selection channel mask for the recording process.

Input: 0x027A & 32 bit selection channel mask (2x uint16)

Command 10. - Start ASIC data acquisition

ID 0x0301 starts the data acquisition process on the implant. The FPGA firmware sends all the necessary information to the ASIC via the RF link. The ASIC starts to collect the recorded data from the RHAs, processes and repacks them and send them then via the RF link to the base station. On the base station, the FPGA polls periodically the rx- buffer fill status of the Zarlink base station module and retrieves the incoming data packages from that buffer. Then the firmware analyses the collected ASIC data package and repacks them into network data packages and sends them to the external PC.

Input: 0x0301

Command 11.- Stop ASIC data acquisition

ID 0x0302 stops the data acquisition process on the implant and the polling of the rx- buffer of the Zarlink base station module by the FPGA. Furthermore, the FPGA cleans out the rx- buffer after some waiting period, allowing the implant to send the already stored data packages (within the implant's tx -buffer) to the base station for discarding.

Input: 0x0302

Command 12. - Read Zarlink register from memory page 0

ID 0x0400 can be used to read one selected register from the memory page 0 of the Zarlink IC. The Zarlink has many different registers allowing to control and manage the ZL70102. There are registers in the address range from 0 to 127 available. The requested register address is sent to the firmware as bit[7-0] of a 16 bit unsigned integer.

Input: 0x0400 & register address (Bit[6-0] of an uint16)

Command 13. - Read Zarlink register from memory page 1

ID 0x0401 is similar to command 12. The difference lies in that 0x0401 accesses the second register memory page of the Zarlink instead the first one.

Input: 0x0401 & register address (Bit[6-0] of an uint16)

Command 14. - Write Zarlink register to memory page 0

ID 0x0402 is the counterpart to 0x0400. It allows to write values into selected ZL70102 registers of memory page 0. The firmware needs for such an operation the address (bit[7-0]) and the value (bit[15-8]) concatenated into one 16 bit unsigned long value.

Input: 0x0402 & unit16 (contains register address (Bit[6-0]) and value (bit[15-8]))

Command 15. - Write Zarlink register to memory page 1

ID 0x0403 is the version of 0x0402 for register memory page 1. It can write values into registers on memory page 1 of the ZL70102.

Input: 0x0403 & unit16 (contains register address (Bit[6-0]) and value (bit[15-8]))

Command 16. - Write 14 byte data block to the Zarlink

ID 0x0404 is used to write a 14 byte data block into the Zarlink IC (at register address 0x2D).

Input: 0x0404 & 14 byte data block (14x uint8)

Command 17. - Read 14 byte data block from the Zarlink

ID 0x0405 allows to manually read from the rx- buffer of the Zarlink IC. In normal ASIC data acquisition mode, it is not required to operate the data collection with this command. Thus this command is dedicated for development purposes only.

Input: 0x0405

Command 18. - Set trigger-out channels of the base station

ID 0x0500 sets the trigger-out channels of the base station. These trigger channels are necessary for signalling other external devices that a special event occurred. Depending on the hardware, there are two firmware versions. One version allows to operate 16 trigger output channels. For these channels the pins of the FPGA are used directly. In an other hardware version, an extra trigger-out card is available. Using two multiplexers and 16 dual d-flip flops, this allows to operate 32 trigger-out channels with only 8 FPGA pins.

Input: 0x0500 & 16 bit string or 0x0500 & 32 bit string (depending on the hard- and firmware)

Command 19. - Set amplifier for the data acquisition of the base station

Depending on the hardware type, the classical cable bound data acquisition system of the base station can have amplifiers which can be programmed in their amplification strength. With ID 0x0279 it is possible to addresses each one of these amplifiers and set their amplification factor. In the actual version of the firmware only up to 24 amplifiers are supported. Increasing this number can easily be achieved. Bit[0] selects which one of the two resistors will be set (A = '0' or B = '1').

Input: 0x0279 & which amplifier (Bit[7-0] of an unit16) & amplification (Bit[7-0] of an unit16)

Command 20. - Get selected ASIC channels and their order

ID 0x0502 tells the FPGA firmware to send a list of the selected channels for the implant to the external PC. The list contains the channel number (uint16), the RHA IC number (uint16) and the RHA input channel ID (uint16) of those RHA ICs.

Input: 0x0502

Command 21. - Start data acquisition of the base station analogue digital converters

The base station can be equipped with a classical cable bound electro-physiological recording system. Using ID 0x0303 starts the data acquisition process for this recording system. The FPGA firmware starts then to deliver data packages of the type 0x8003 to the external PC. But this only works if at least one ADC is connected to the base station.

Input: 0x0303

Command 22. - Stop data acquisition of the base station analogue digital converters

ID 0x034 stops the data acquisition process of the base station which was started with 0x0303.

Input: 0x0304

Command 23. - Read trigger-in channels from the base station

ID 0x0503 requests the actual state of the trigger-in channels of the base station. The firmware returns a 32 bit string (2x uint16). Depending of the used hardware, the trigger-in channels are directly the pins of the FPGA or realised through an extra PCB with a multiplexer. In the case of the multiplexer only 8 FPGA pin are used instead of 32.

Input: 0x0503

Check sum - CRC

In a medical application it is very important to ensure that the commands, which a medical device receives and orders it to perform a certain task, was really created and sent by the user. Furthermore, it is also important that the data an implant delivers to the medical personal for medical diagnostics or other external analysis devices are correct. For example could a undetected bit flip in a most significant bit in a configuration value cause the implant to use the wrong sample rate or initiate the wrong task and so on. For addressing this issue, we included a check sum (cyclic redundancy check 'CRC') into the communication between the base station and the external PC.

For future versions of the ASIC, it is planned to introduce also a check sum into the communication between the ASIC and the base station. The communication via

Zarlink RF link has already a build in check sum check. Nevertheless, it is important to insert a check sum as early as possible into the data. This would allow us to detect bit flips during the whole data processing and not only during the RF transmission. On the other hand, a check sum uses bandwidth from the already very limited available resources.

Another important aspect for a future medical implant are security issues. In medical implants that receives or transmits information, it should be mandatory that the channel of information exchange is encrypted. Otherwise a hacker could take over the implant and render it dysfunctional or do harm to the patient (especially if it has simulation features). Securing implants will be very important in the future. In the developed brain implant, we have not included any protection of this kind yet.

Every incoming and outgoing network packet ends with 8 bytes of check sum. The 8 byte of check sum contains four 16 bit unsigned integer counters. The first counter tells us how many '00' bit combinations have occurred in the network package (except the 8 check sum bytes). The second 16 bit word tells us how many '01' bit combinations were found. The third word corresponds to the '10' bit combinations and the last counter represents '11' bit combinations.

The implementation of the check sum in firmware is very easy. All the data that the FPGA enters or leaves, comes in 16 bit words. The check sum calculator only needs to work on one word at the time but has only one clock cycle time to check it. A simple three layer approach can solve the task. In the first layer we use 8 parallel modules that look at the 8 two bit sub-sets. These modules deliver us the information about the type ('00', '01', '10' or '11') of each of the sub-sets. In the second layer, we count the number of found '00', '01', '10' and '11' combinations in this word. In the third layer we accumulate these four numbers over the whole package length. After the end of the package we read out the four numbers, use them and then reset the counters for the next package. This pipeline approach is fast enough to perform the given task in the available time of one clock cycle (on these type of Xilinx Spartan 3 FPGA).

2.1.4 Network commands modules - Firmware

After the definition of the packet structure and the required control sequences, these specifications had to be implemented in the firmware. Very early, it was clear that the number of commands will not be fixed and some commands need to be exchanges during development. Thus it was important to use a design concept, that allowed for adding and removing commands without too much effort.

The concept that was most promising was one command module per network command connected to the Zarlink RF and network resource via a three state bus ('0','1' and the high-impedance state 'Z'). Orchestrating the access to these resources by an arbiter (figure 2.16). Furthermore the idea was to save resources by producing special modules that are used by most of the command modules (like the 'check sum' module and the 'Input Packet Interpreter' module).

All the incoming network packages are collected by the finite state machine from Orange Tree and, and due to a simple modification of their original firmware, were stored into a 4096 word deep and 16 bit wide FIFO, which also bridges the two clock domains (the Ethernet part with 125 MHz and our custom firmware part with 95.833 MHz). All the 16 bit words stored in the FIFO are processed by 'Input Packet Interpreter' module (see figure 2.17).

This module has two main tasks: First it has to check whether the check sum of the package found at the end of the command is okay (and thus checking if the length of the command is also okay) and second, it has to determine whether the analysed command is of a known packet type or an unknown packet type. The payload (beginning after the fixed synchronisation value and the length of the package and ending directly before the CRC information) is transmitted to all the command modules. The 'Input Packet Interpreter' module has two additional outputs that can mark the actual command package as unknown or not valid. Furthermore, the module signals the start and the end of the package.

In the case of an unknown type, a special module reacts to the unknown package. This module reacts to the 'unknown tag' of the 'Input Packet Interpreter' module and produces a network response signalling the external PC that something is wrong. The structure of this 'unknown'- handling module is a simplified version (mainly the Zarlink part was removed) of the template module (see figures 2.18, 2.19 and 2.20) that was used to create most of the command modules.

This network command module template was constructed as follows (see figures 2.18, 2.19 and 2.20): The module looks for valid data from the 'Input Packet Interpreter' module. If this new command package does have a different command ID to its own then the module goes back into its waiting position. In the case that a new package starts and the package has the correct command ID then the module starts the data processing. First it collects all the data from the 'Input Packet Interpreter' module (in the case there is additional data) and waits for the information whether or not

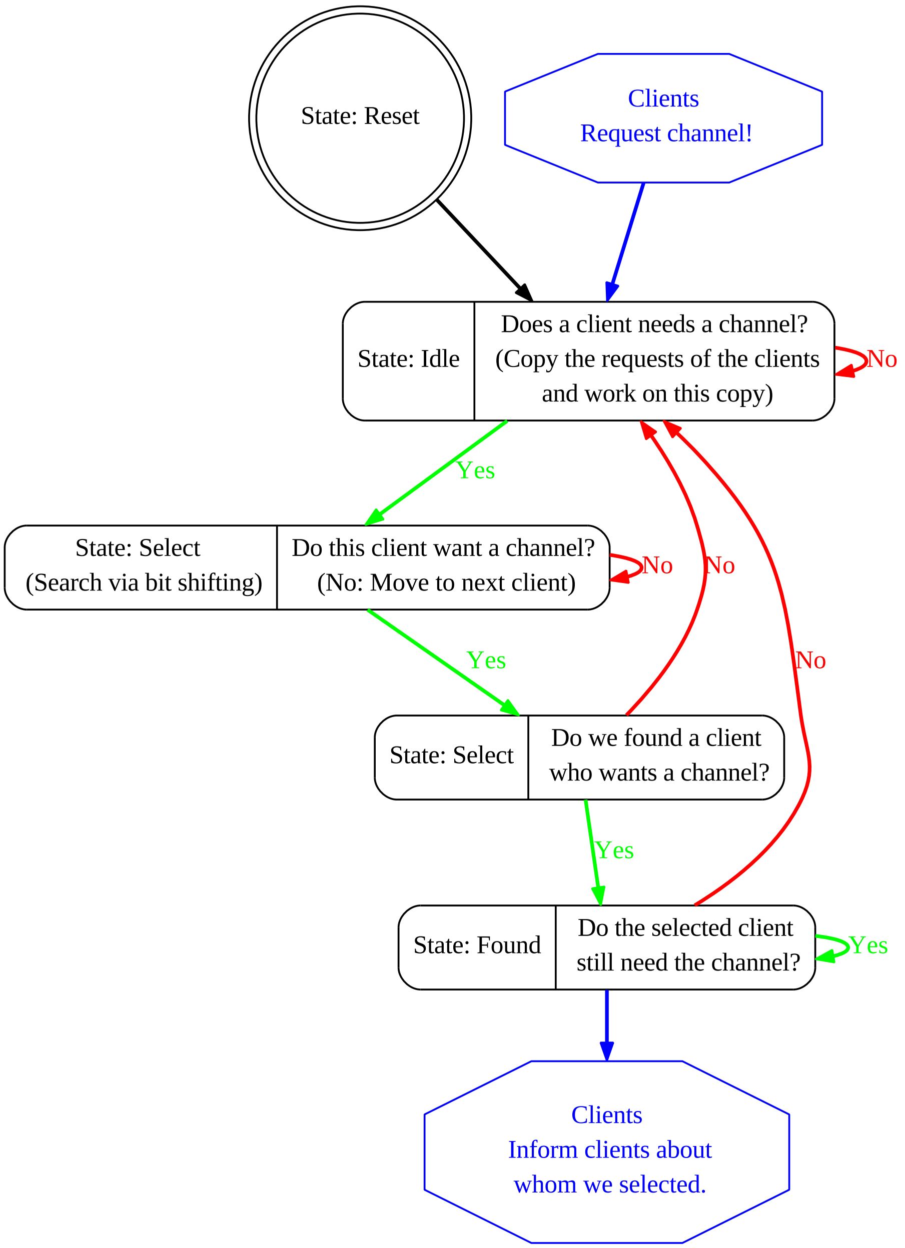

Figure 2.16: Arbiter organising the access of all the modules to the Zarlink and the network resources.

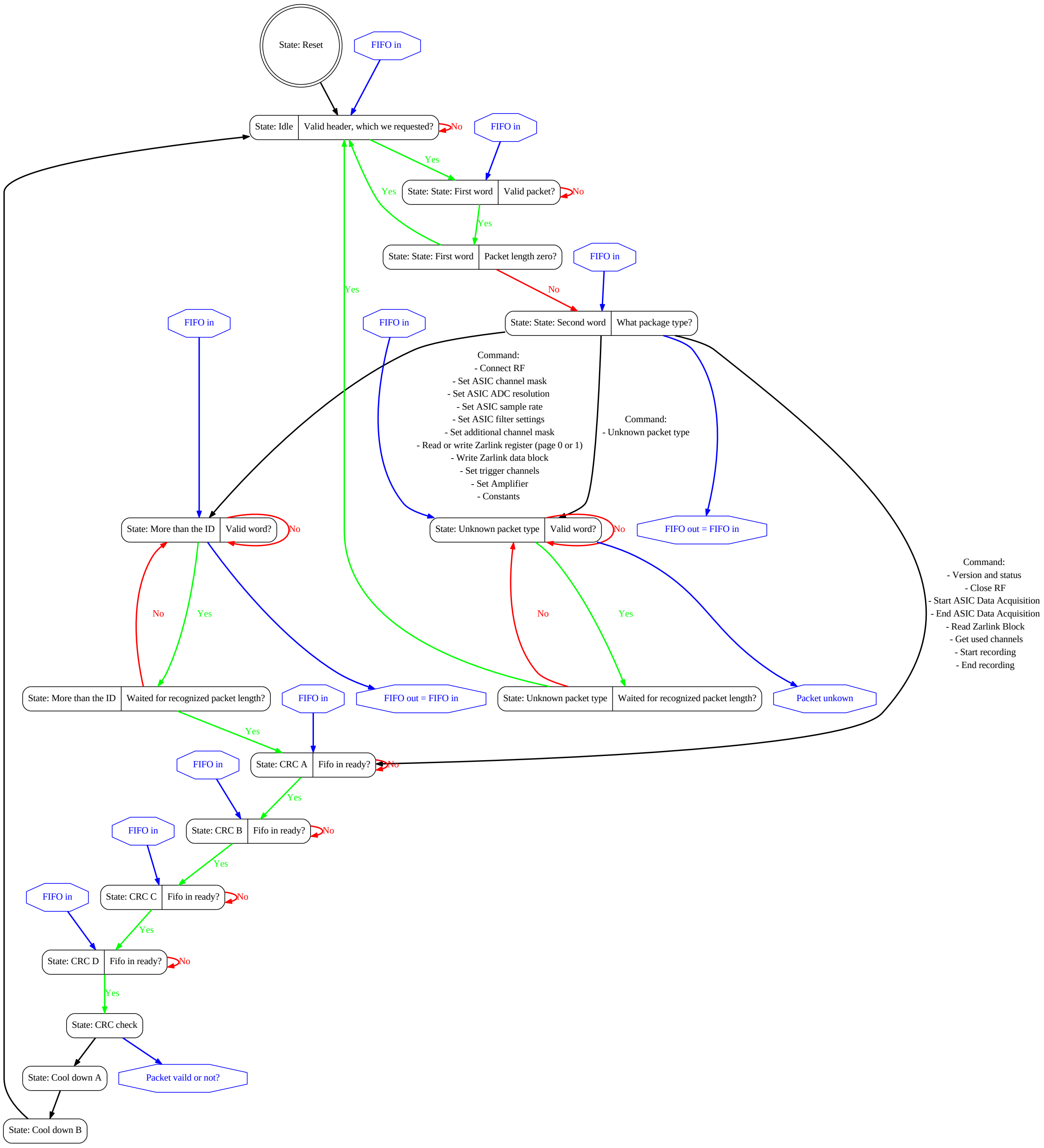

Figure 2.17: 'Input packet interpreter' module checks if the incoming package is of a known type and if the check sum is correct.

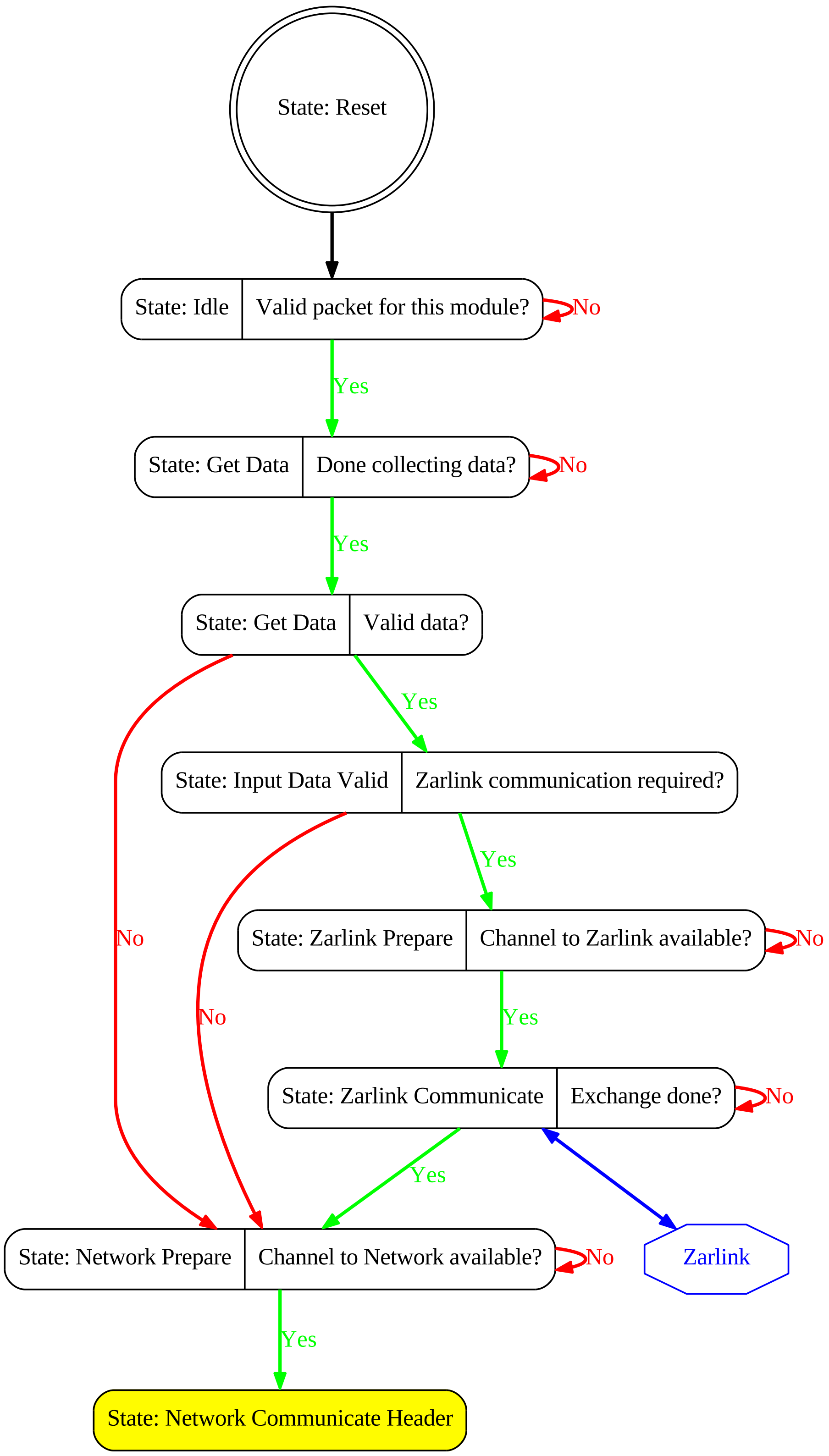

Figure 2.18: Template for the typical FSM of a command module. 1 of 3

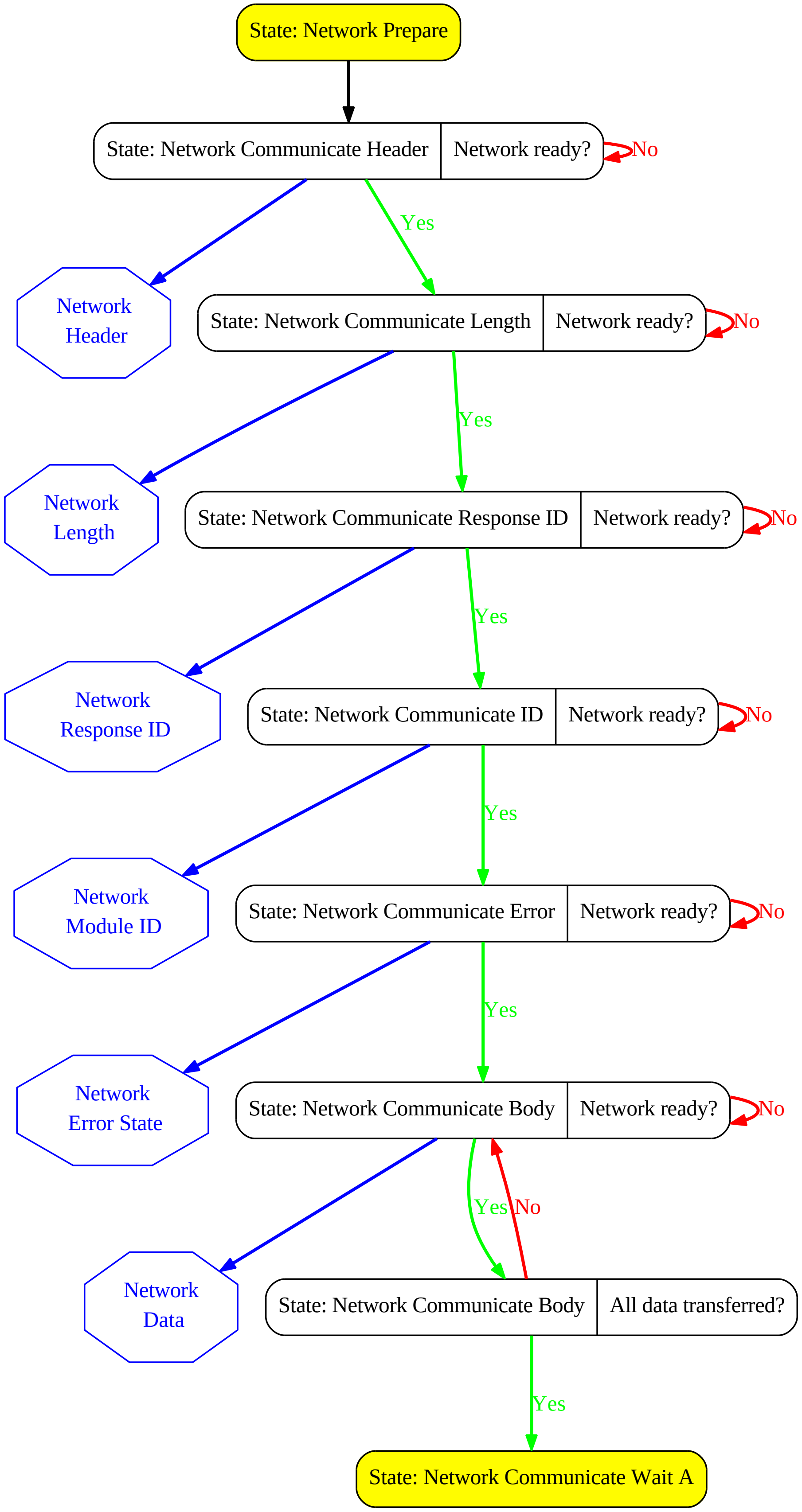

Figure 2.19: Template for the typical FSM of a command module. 2 of 3

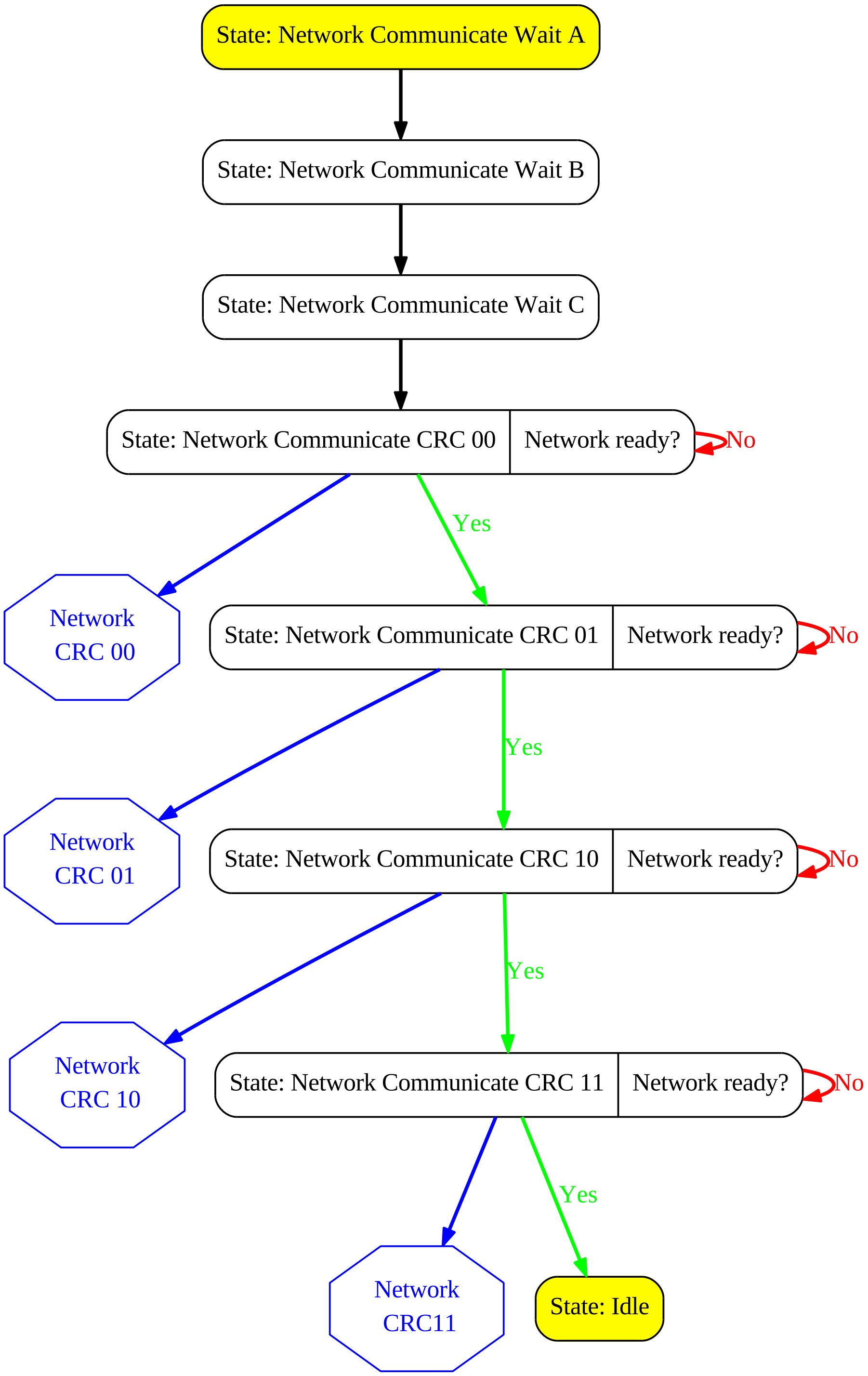

Figure 2.20: Template for the typical FSM of a command module. 3 of 3

this command package is valid. If it is not valid then the module will generate a network package for the external PC, informing it about the problem. With valid data it continues and to sets internal constants (if any were defined in the given module). After that the module can communicate with the Zarlink interface if required. And finally, the module can send data via the network interface back to the external PC. This response will give information about the success of the command and can deliver requested information.

With only a few exceptions, the majority of the command modules are designed using this template. Using VHDL allowed to introduce 'generic' constants in the state machine for defining the command ID, the length of the payload (and if there is any) as well as if the Zarlink interface will be used by this command. During the processing of the incoming data, the command module signals the 'Input Packet Interpreter' module that it is working on the actual command package. This signal stops the 'Input Packet Interpreter' module from starting with the processing of a new command package. Otherwise a second command with the same command ID could have been ignored while the relevant command module is busy.

Since most of the interface for the command modules are identical, it makes it very easy to write test environments for these modules. Furthermore, debugging is relative simplified because major (already verified) parts of the modules are reused without changes.

2.1.5 Zarlink modules

An important feature of the base station and the firmware is the ability to communicate with the implant via a RF link. This link is realized with a Zarlink ZL70102 base station module and a Zarlink ZL70102 implant module. The RF link has a maximal RAW data transfer rate of 800 kbit per second. The manual of the IC tells the reader that this translates in a 515 kbit per second transfer rate. In reality it wasn't possible to achieve these high transfer rates and in many aspects the ZL70102 reacts very sensitive, which makes the application of these ICs rather complicated.

In an application where the implant sends a continuous stream of data from the implant module to the base station module, the data transmission pauses at random points in time and for a random length of time. Thus it is important to have an extra (as large as possible) transmission buffer in the implant as well as in the base station to avoid data loss in such a continuous data transfer mode. This continuous mode is essential for the normal data acquisition operations of the implant.

Several parameters interact with the RF link: First of all, it is necessary to check the ZL70102 buffer-fill-status before collecting data from the receiver-buffer or putting new data into the transmitter-buffer. This status check is done via SPI (Serial Peripheral Interface) communication. Following the manual of the IC, this communication is possible with up to 4MHz. We found that interacting with the IC via SPI causes problems for the RF link. In a worst case scenario it is possible to block the whole RF data transfer with these SPI buffer-status requests. Thus it was necessary to reduce the number of buffer-status requests as much as possible and skip every non-mandatory SPI command. Furthermore, the 'wrong' waveform of the SPI clock and SPI data-in signal can also cause perturbations in the RF link.

After a significant number of tests, debugging and communication with the company Zarlink (now MicroSemi), we were able to find acceptable parameters. The SPI frequency was limited to around 1.5 MHz. With this data transfer rate it is possible to collect all the data and do the necessary buffer checks. For preventing over- and undershoots as best as possible, we set the FPGA pin parameters to 4 mA drive strength and a slow slew. Furthermore, we had to set the FPGA pins for the SPI to PULLDOWN (except the input SDO, which had to be set to PULLUP). Between transferring a block of 14 byte, we introduced a small pause. Finally, we had to deactivate the Zarlink watchdog. Otherwise it was not possible to create a connection between the implant and the base station because the already made configuration were re-setted regularly. It seems that optimizing the training string for the RF link, can reduce the amount and length of these unwanted transmission pauses.

In the figures from 2.6 to 2.13 it is depicted how to create a connection between the base station module and implant. This procedure uses the 'Zarlink Echo' module (see figures 2.22 to 2.26), which uses intensively the 'Zarlink SPI' module (see figure 2.21) for the implementation of the low-level SPI protocol.

'Zarlink SPI' module

The communication between a Zarlink ZL70102 IC and any controller (micro-controller or FPGA) is done via SPI (Serial Peripheral Interface). The controller is always SPI master and the Zarlink is SPI slave. It is possible to read from and write to a register address within the Zarlink. There are different modes for accessing the Zarlink but in the following I will only described what I have used for the firmware. First, we tell the Zarlink if we want to read or write, then give it a seven bit long register address. After that we can, without any pause, write data to the SDI pin or read data from SDO pin (most significant bits are transmitted first). In most cases we read or write one byte. An exception is when we have to deal with the rx- or tx- buffer of the Zarlink. In this special case we must read or write in multiples of 14 byte (this number can be configured using configuration registers but 14 byte is the largest value with no incomplete bytes). Tests showed that reading or writing only 14 byte, followed by a small pause, was less problematic. Thus we can restrain the module in the firmware (see figure 2.21) to transfers with 15 bit or 119 bit plus the read/write bit.

'Zarlink Echo' module

In theory, it would be possible to let all the sub-modules use the 'Zarlink SPI' module directly. In terms of using the resources of the FPGA more efficient and decrease the time needed for debugging, it was beneficial to write an extra module that is placed between the SPI module and other modules requiring the Zarlink. In the figures 2.22 to 2.26 this module is described in more detail. Beside serving the 'Zarlink SPI' module, it takes care of the buffer status in the so called '14 byte block transfer' mode. Furthermore, it implements a way to globally debug the Zarlink connection and export all what is send via the RF link. In this debug mode, a copy of all the incoming and outgoing data is send out via Ethernet (see figures 2.23 to 2.26 for details).

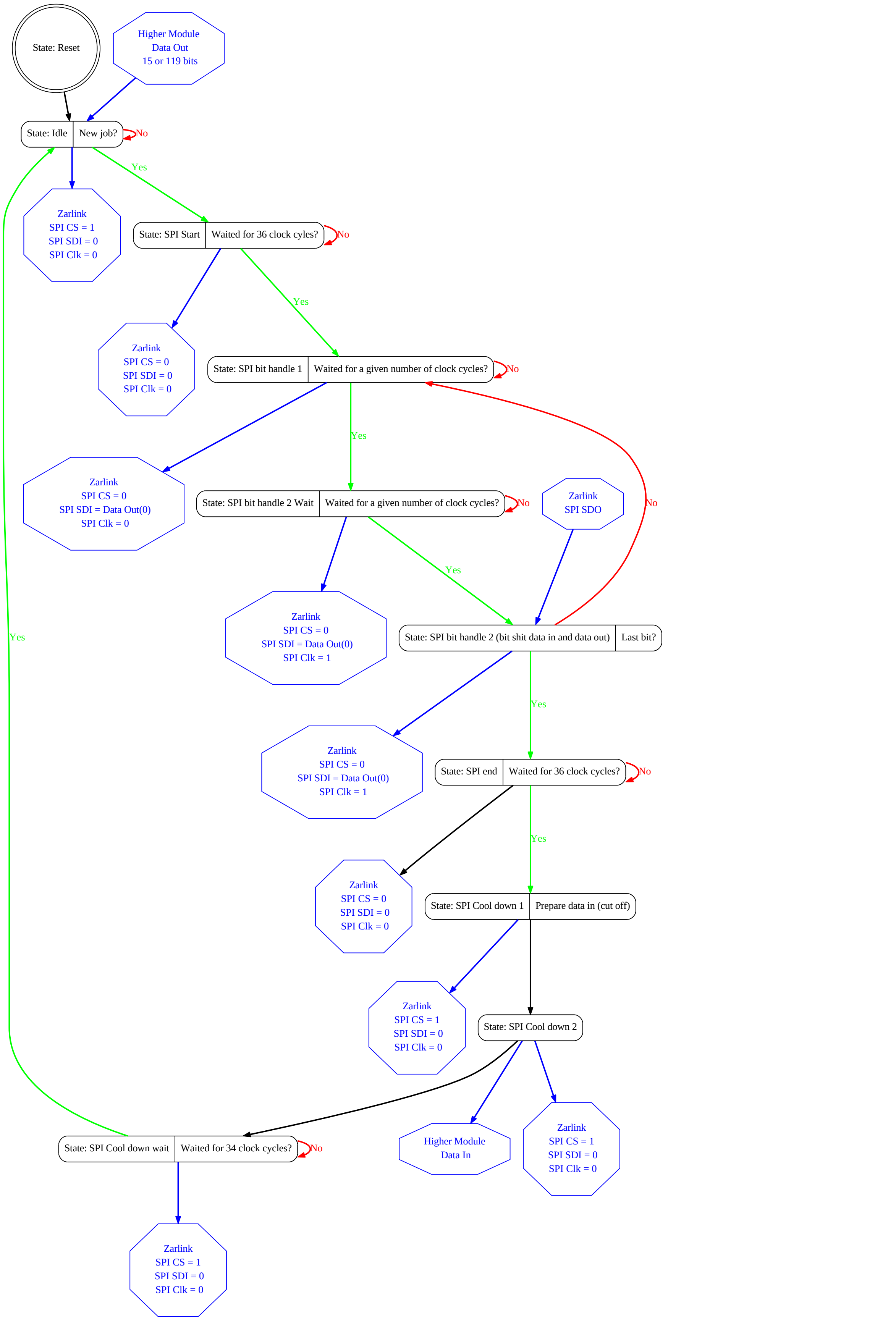

Figure 2.21: For interchanging data between the FPGA and the Zarlink IC the 'Zarlink SPI' module handles the low-level SPI protocol.

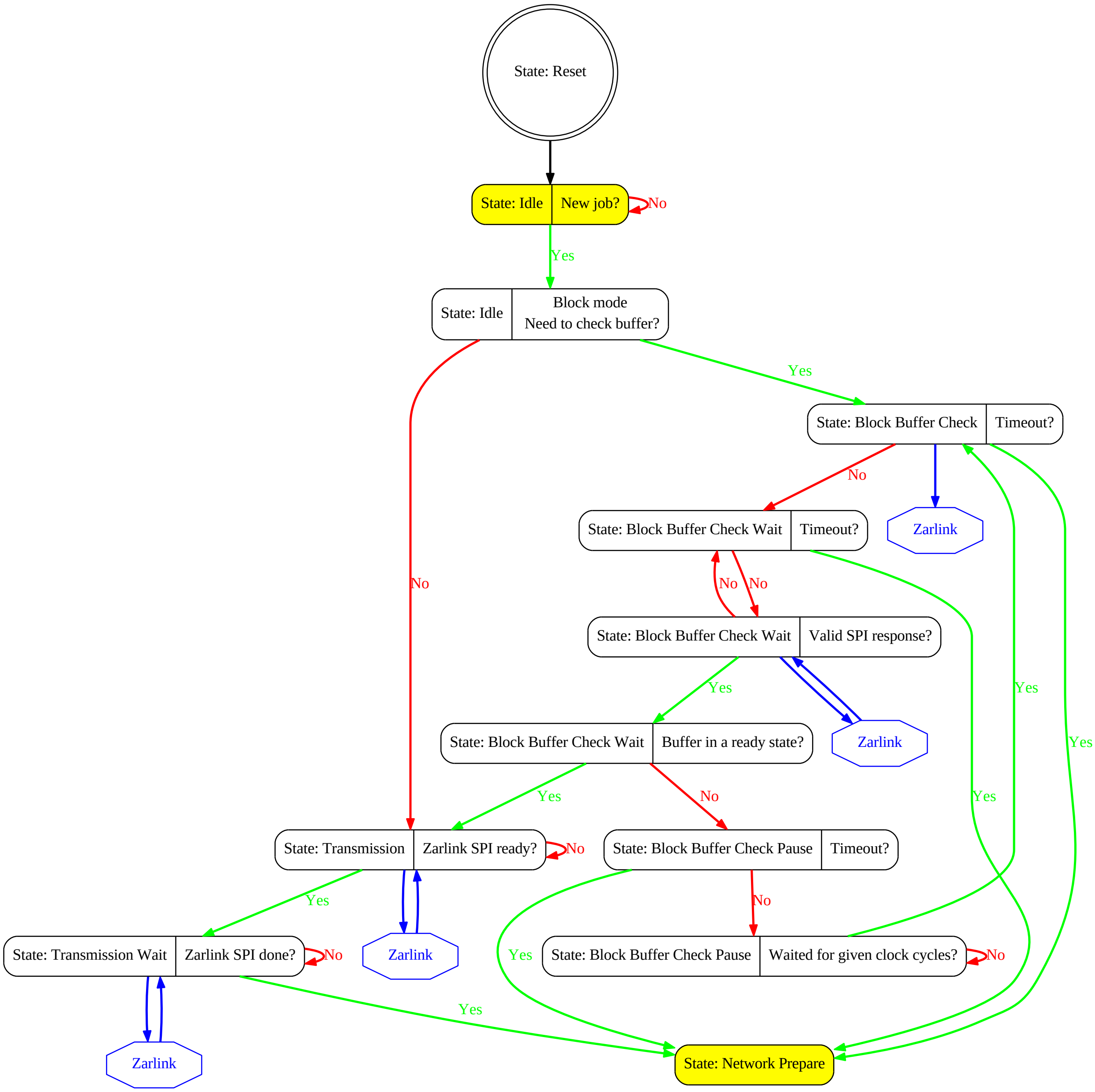

Figure 2.22: 'Zarlink Echo' module is an abstraction layer between SPI layer and the normal data processing modules. 1 of 5

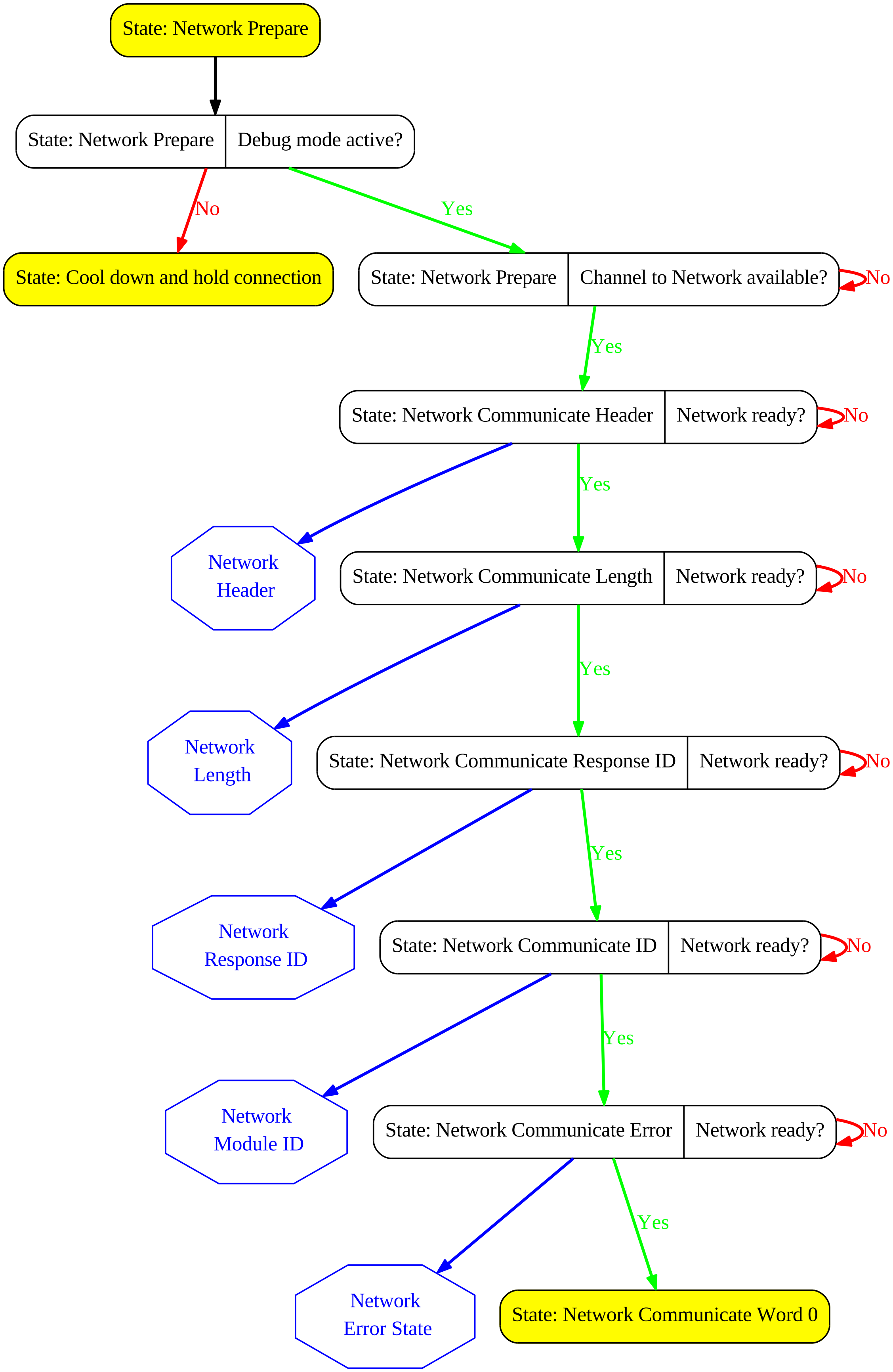

Figure 2.23: 'Zarlink Echo' module is an abstraction layer between SPI layer and the normal data processing modules. 2 of 5

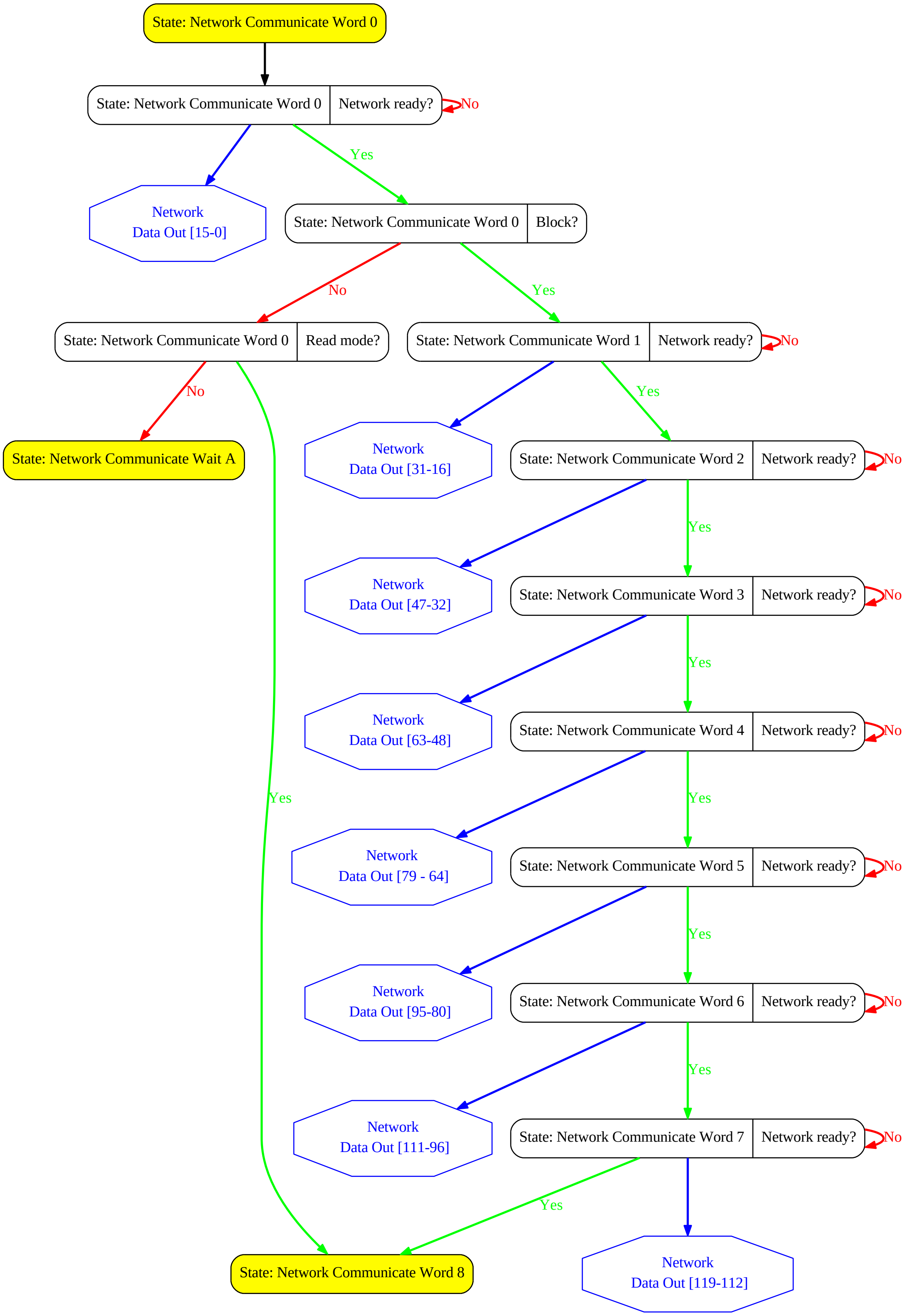

Figure 2.24: 'Zarlink Echo' module is an abstraction layer between SPI layer and the normal data processing modules. 3 of 5

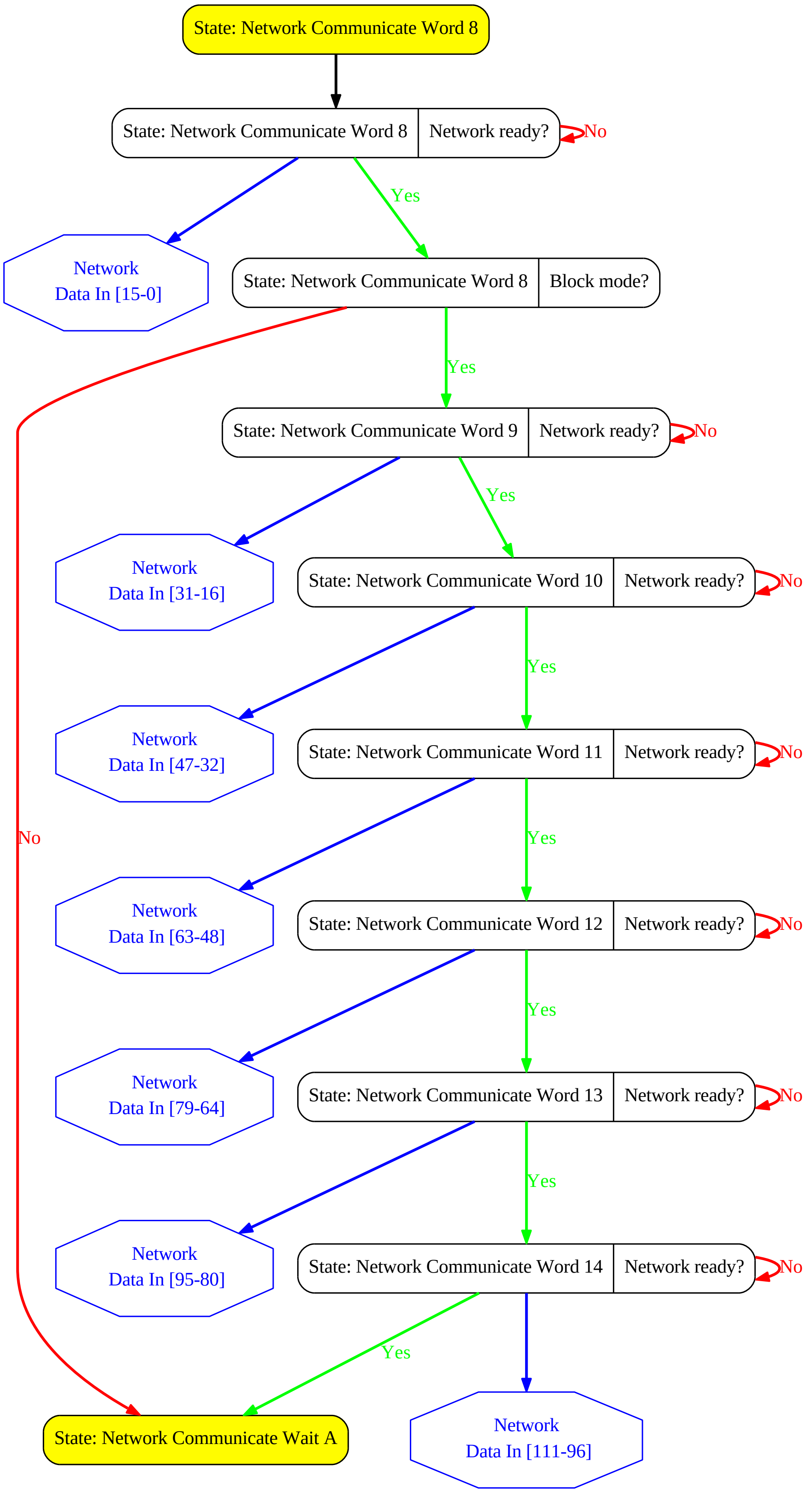

Figure 2.25: 'Zarlink Echo' module is an abstraction layer between SPI layer and the normal data processing modules. 4 of 5

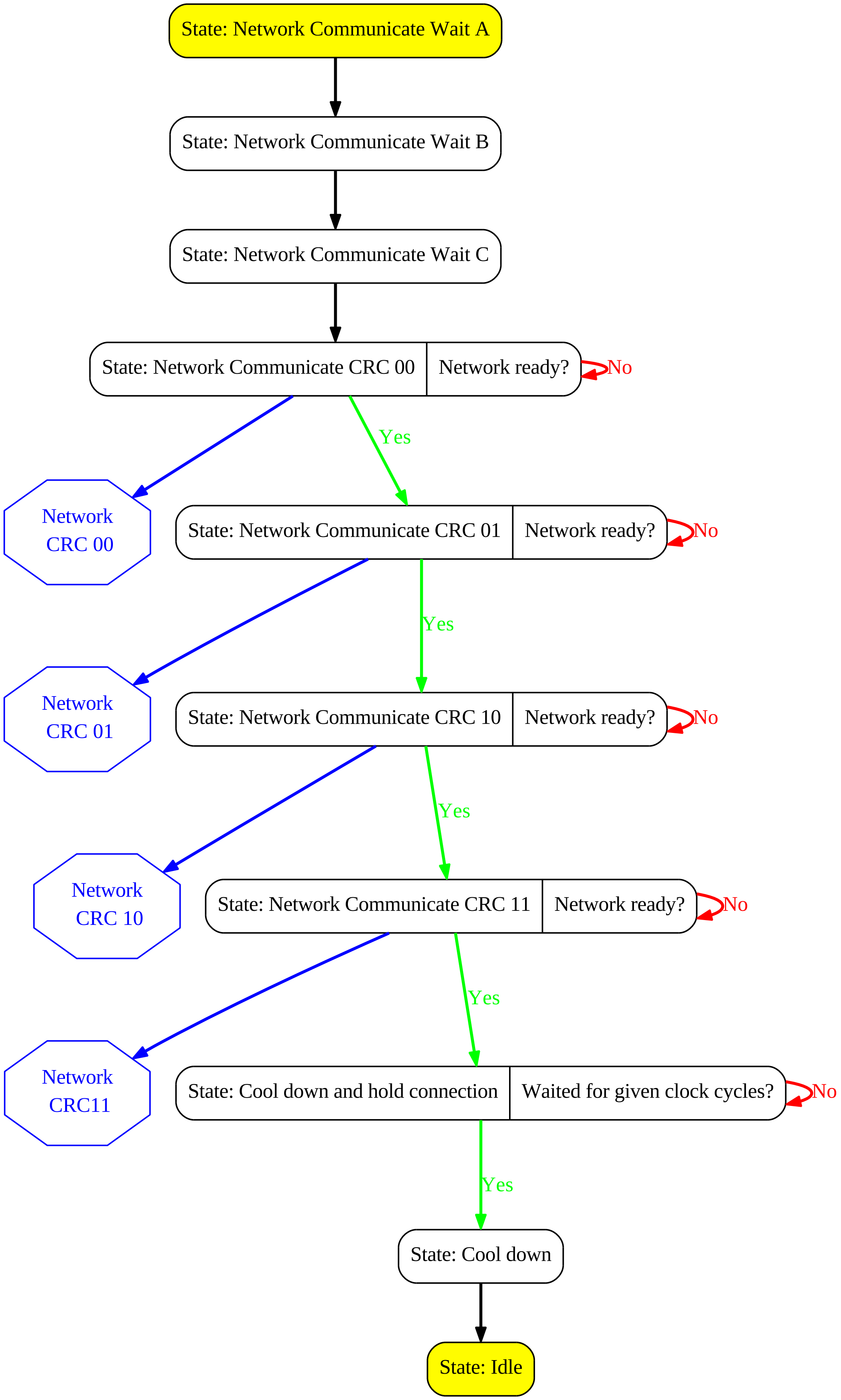

Figure 2.26: 'Zarlink Echo' module is an abstraction layer between SPI layer and the normal data processing modules. 5 of 5

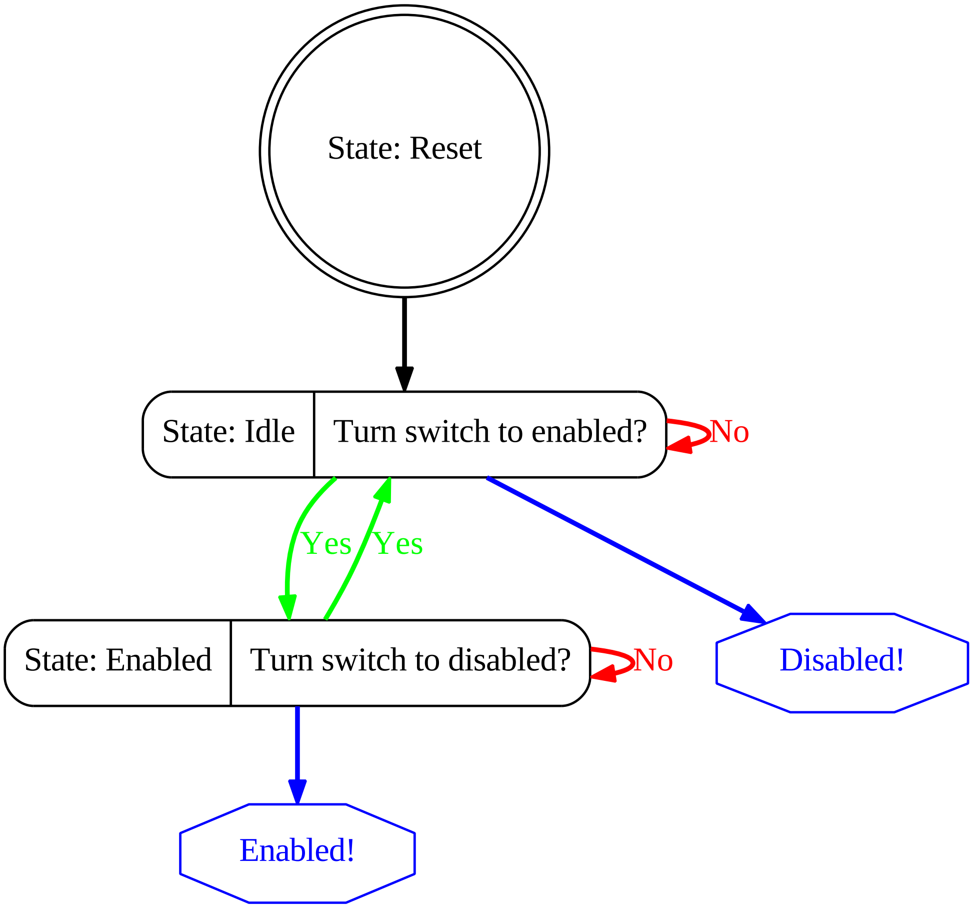

Figure 2.27: Bi-stable switch module for turning the data collection mode on or off via a network command.

2.1.6 Implant data handling modules

After the data acquisition on the implant was started, a continuous stream of data arrives in the rx-buffer of the Zarlink at the base station. Since the Zarlink has only a maximal buffer size of 64 x 14 byte blocks (to be precise: 64 x 113 bit), it is essential to read out the buffer regularly to prevent a buffer overrun and data loss.

After the command for starting data acquisition on the implant was send via RF link, a bi-stable switch is set to its 'on' position (see figure 2.27 ; this switch is set to its disabled position when the data acquisition is stopped again). The status of the switch is observed by the 'Zarlink Data Collector' module (see figure 2.29 and 2.30). When the data polling enabled then the collector module services a clock module (see figure 2.28) for coordinating the time between two read-out attempts.

In theory it would be a good solution to check the buffer-fill-status as often as possible and read out all the data if any is found. In reality, checking the status causes perturbations in the RF link, thus the number of buffer-status read-outs need to be as low as

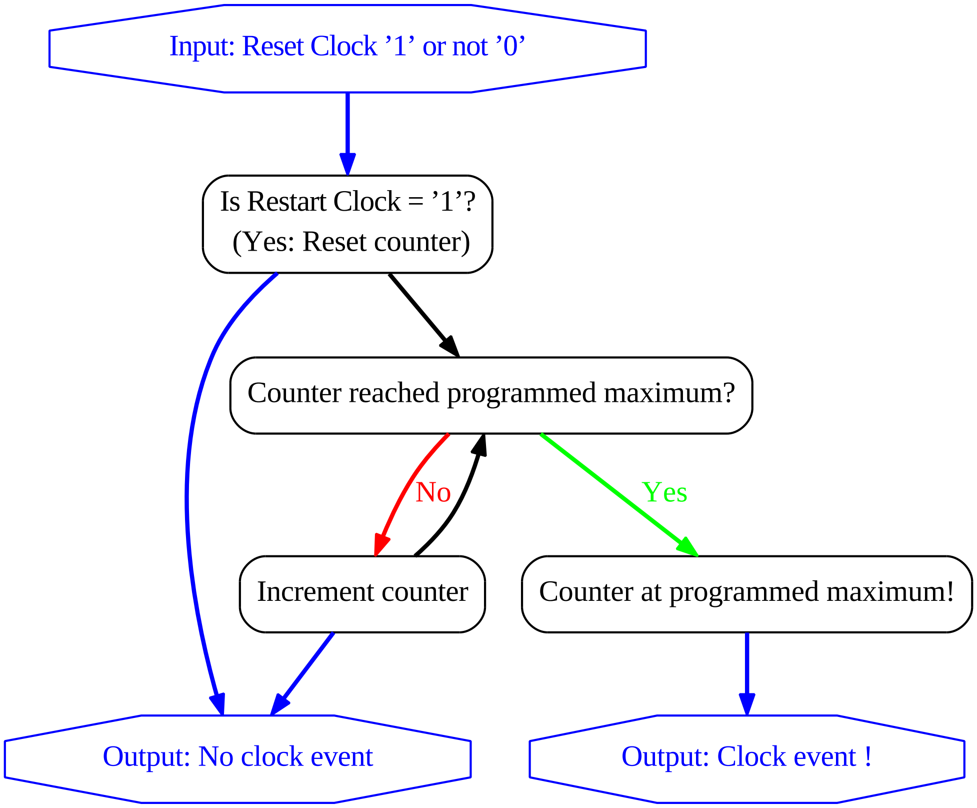

Figure 2.28: A count-down module for determining when the next check of the buffer and its read out has to be done.

possible. An alternative method could have been to use an interrupt pin for signalling that data is available for read-out but the manual of the Zarlink IC states that for a high speed application it is absolutely necessary to check the buffer-fill status-register instead.

The polling clock represents the fact that in an application with a continuous data transfer, the rx-buffer-status is only updated by the Zarlink after several of these 14 byte blocks have been transmitted and not for each individual 14 byte block. With this clock, we prevent any unnecessary buffer-status read-outs in this unfruitful time period.

After the polling-clock signals the collector module that there is the possibility of data within the buffer, the collector module reads out the buffer-fill-status. If it founds a number of blocks in the buffer then it reads them out (see figure 2.29). After that, it checks if there are new packets in the buffer. If not then it restarts the polling clock for a new waiting period. Otherwise it continuous to read the data out of the buffer. In addition, this module allows in debug mode (see figure 2.30) to check the link quality of the RF link.

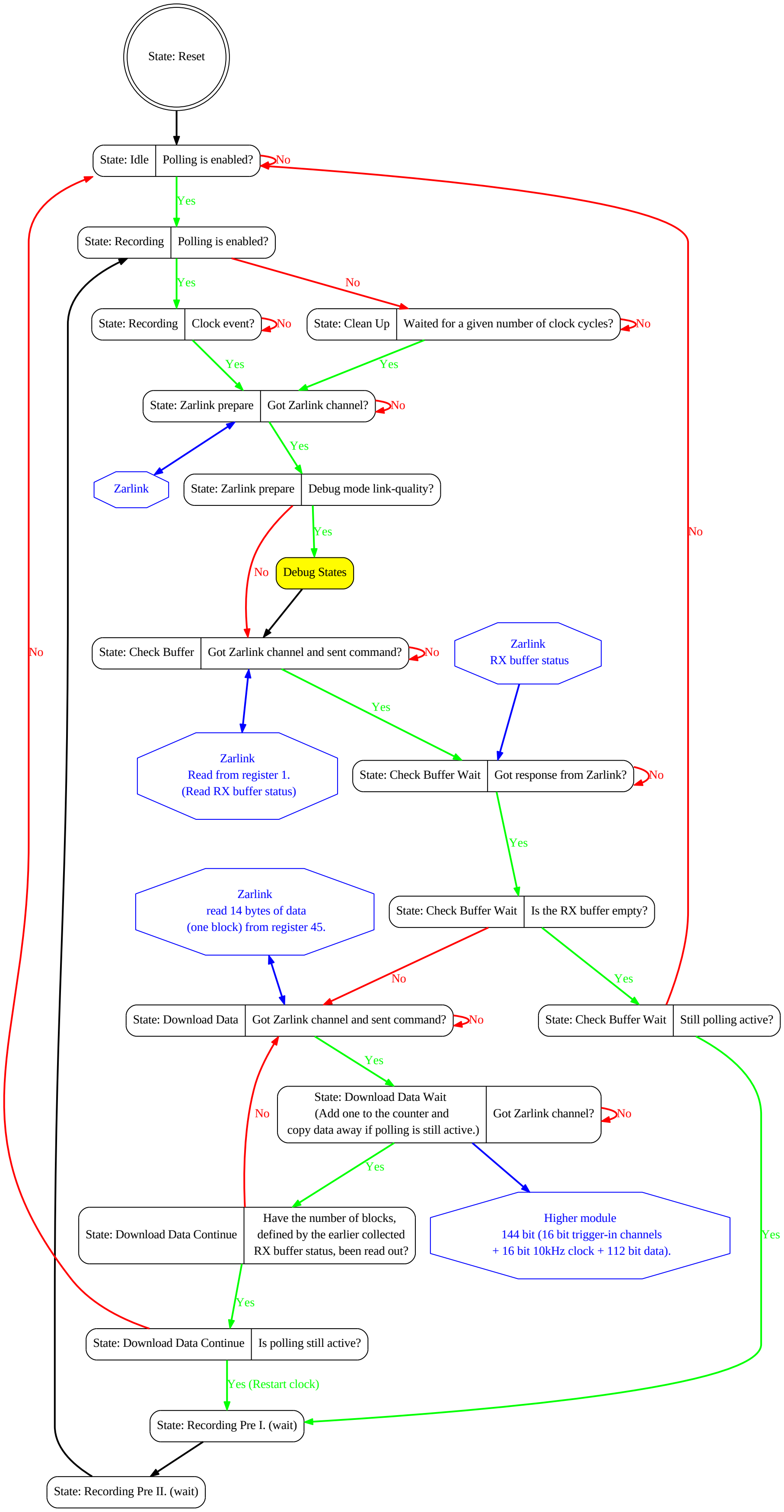

Figure 2.29: 'Zarlink Data Collector' module.

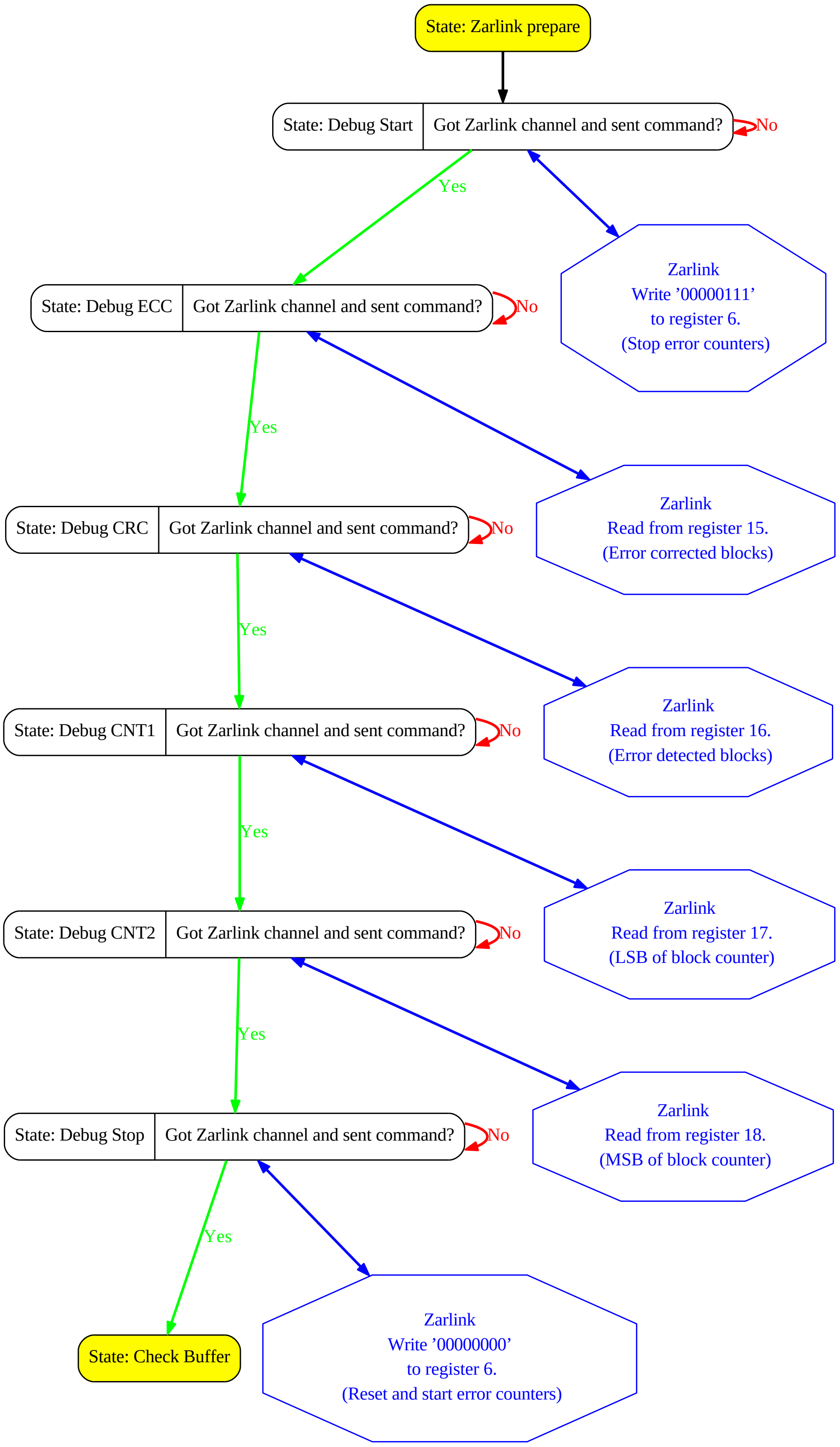

Figure 2.30: ’Zarlink Data Collector’ module. Part for debugging the link quality.

The ASIC data packages

The ASIC for the implant, constructed by the ITEM (Jonas Pistor and Janpeter Hoeffmann) receives information via RF link about: a.) the channel mask, determining which channels are selected to be recorded, b.) the RHA filter setting, c.) the desired sample rate and d.) the resolution in bit for the individual samples. With this information it is possible to start the data acquisition process on the implant. Then the ASIC starts to collect the measurement data from the connected RHAs and then processes them. The result of the information processing is send via RF link to the external base station.

The data from the ASIC is organized in packages. One package consists out of a header and followed by the processed data for all selected channels at one sampling time. There are two types of headers. A long version and a short version. After starting a new data acquisition session, the first header is a long header. This allows us to check if the setting within the ASIC of the implant uses the parameters which we provided earlier. After first packet with the long header, the ASIC sends out only packages with a short header. This design decision was done for saving as much as possible bandwidth for the pay load.

The Zarlink delivers 14 byte blocks (= 112 bit). If the first bit is one then this data block starts a new package. If it is zero then these 14 bytes are a continuation of an ongoing package.

A long header is constructed like follows:
First 14 byte block:
'1' (= Header) & Packet-Type ("0000") & Device-ID (8 bit) & Channel Mask (99 bit of 128 bit)
Second 14 byte block:
'0' & Channel Mask (last 29 bit of 128 bit) & RHA Filter (4 bit) & Timestamp (16 bit) & Sample rate (8 bit) & ADC resolution - 1 (4 bit) & '0' & Pay load data (49 bit)

For a short header the channel mask, the RHA filter settings, the sample rate and the ADC resolution are not transmitted. Thus the short header looks like:
First 14 byte block:
'1' (= Header) & Packet-Type ("0001") & Device-ID (8 bit) & Timestamp (16 bit) & '0' & Pay load data (82 bit)

All the following 14 byte blocks begin with a '0' and contain 111 bit of data. Within the pay load only (ADC resolution) x (number of selected channels) are valid bits.

This packet structure is optimized for the transfer via a strongly band-limited RF link. Exporting this type of data structure directly to the external PC would create a strong computational load on the real time data analysis for the software side. Thus the goal was to convert the incoming Zarlink blocks into outgoing network packages (see section 2.1.3), which are simple to use (e.g. all samples are converted to 16 bit values) even if

extra network bandwidth is required for archiving the simplification.

For 'refurbishing' the packages, I wrote two modules. The 'Data Packet Refurbish' module is dealing with the two headers types (and checking if they are valid) and the overall package structure of the incoming Zarlink blocks (see figure 2.31) as well as preparing the newly created network packages for sending them out via the Ethernet (see figure 2.32 and 2.33). If Zarlink packages from the ASIC are found to be corrupt then an error is send via network (see figure 2.34). It is also possible to activate a debug modus (see figure 2.3 to 2.5) allowing us to look into the details of the 'refurbishing' process from the outside.

The second module, which is a sub-module for the 'Data Packet Refurbish' module, is the 'Stream Decomposer' module (see figure 2.36). Depending on the ADC resolution setting, one sample has a different length within the data package from the ASIC. This module has the task to convert the individual samples into 16 bit samples. Therefore, the single samples have to be cut out of the data stream and filled up with zeros for the most significant bits. The task gets complicated due to the fact that one sample can be distributed over two Zarlink blocks with different bit fragments. Furthermore, the module needs to be able to handle different numbers of channels defined by the actual channel mask (for counting how much channels are active, the 'Active Channel Counting' module recalculates this number every time after the channel mask was changed; see figure 2.35).

The 'Data Packet Refurbish' module prepares the network packages and stores all the necessary data as 16 bit words in a 17 bit wide and 1024 words-deep FIFO. The 17th bit is used as marker where the package ends (='1'). In addition, the refurbish module uses a special counter (see figure 2.37) to signal the next higher module that there is a new fully complete package in the FIFO available.

When the 'Network Data Process Interface' module gets the signal – via the 'Network Packet Counter' module – from the 'Data Packet Refurbish' module that a fully complete data package is ready, then it checks if there is the necessary empty space (1096x 16 bit words) available in the outgoing network FIFO (16 bit width and 4096 words deep). Then it organizes the exclusive access to that FIFO. It copies the complete package into the outgoing network FIFO and adds the 4x 16bit-words of CRC information to it. Finally it informs the 'Network Packet Counter' module that there is now one full data package less in the FIFO between this network interface module and the 'Data Packet Refurbish' module.

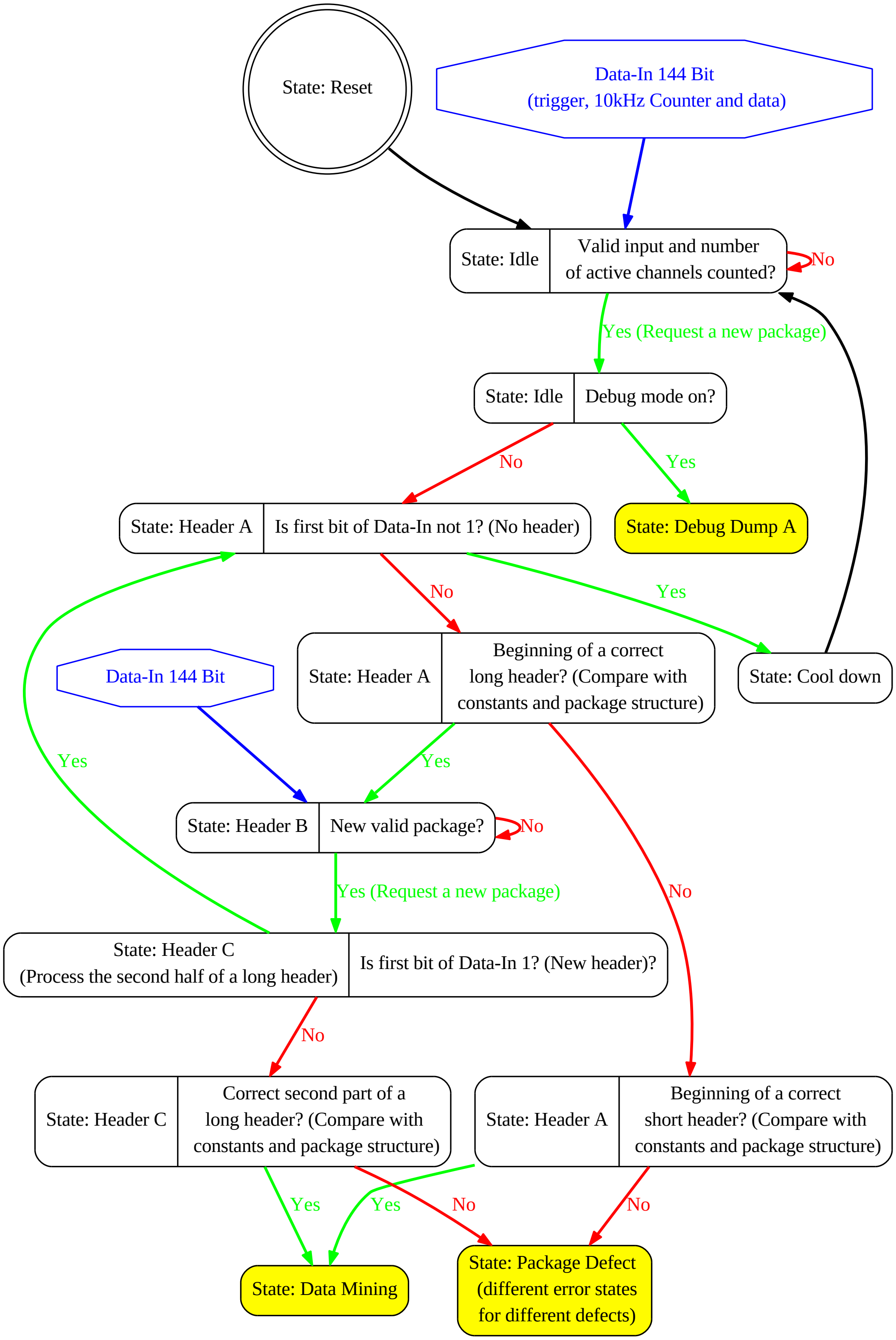

Figure 2.31: 'Data Packet Refurbish' module handling and checking the headers. 1 of 3

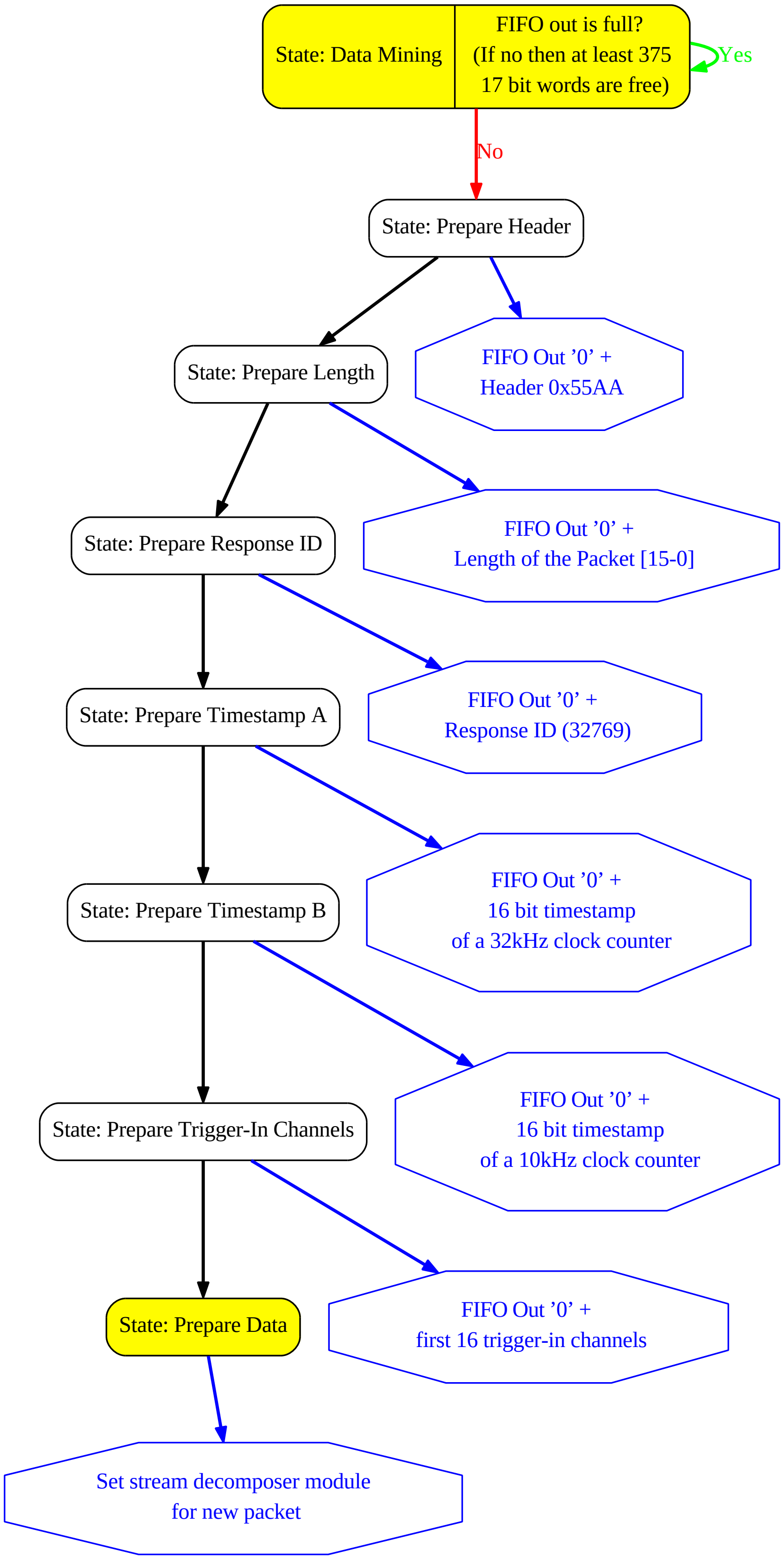

Figure 2.32: 'Data Packet Refurbish' module preparing network packages from the data. 2 of 3

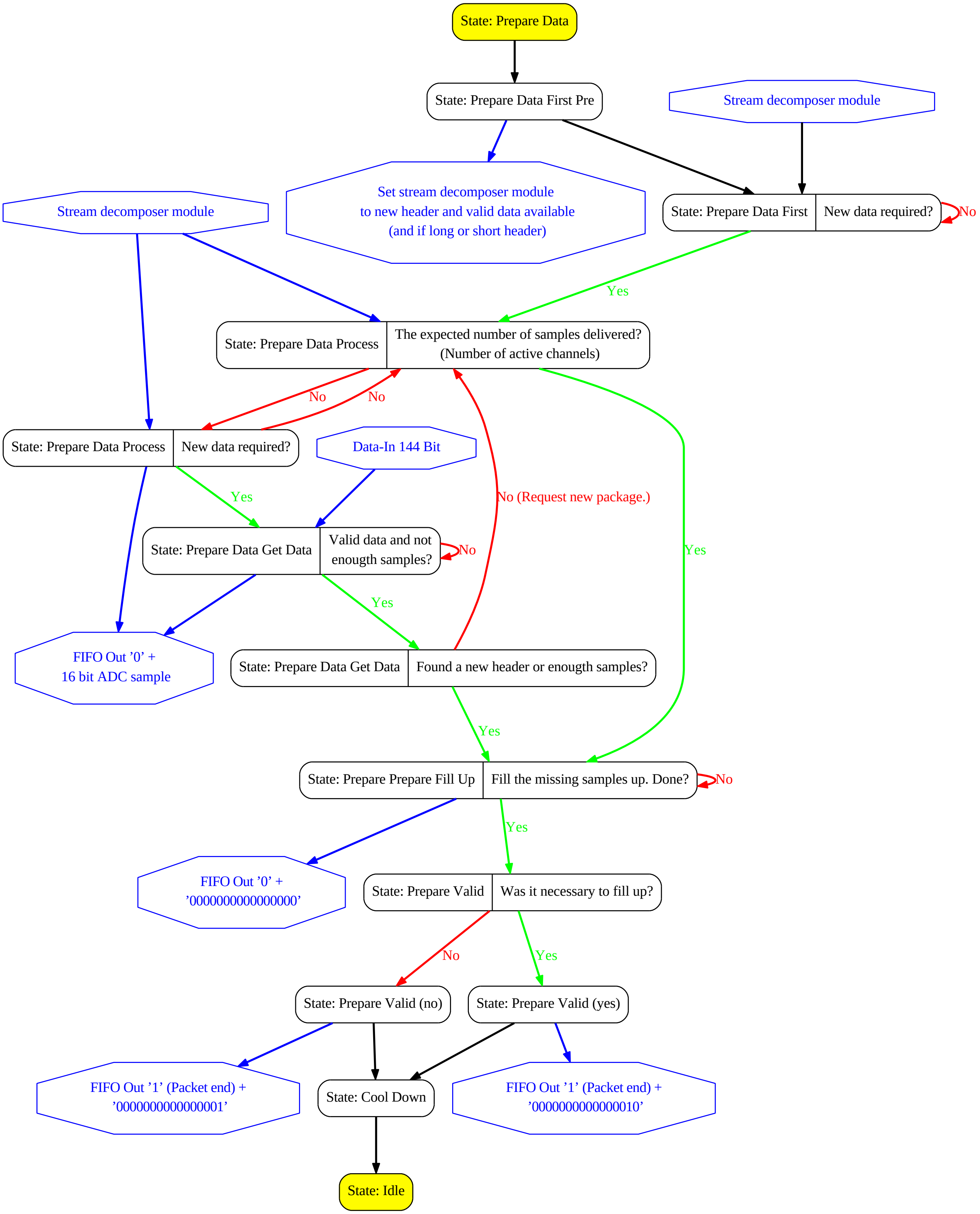

Figure 2.33: 'Data Packet Refurbish' module preparing network packages from the data. 3 of 3

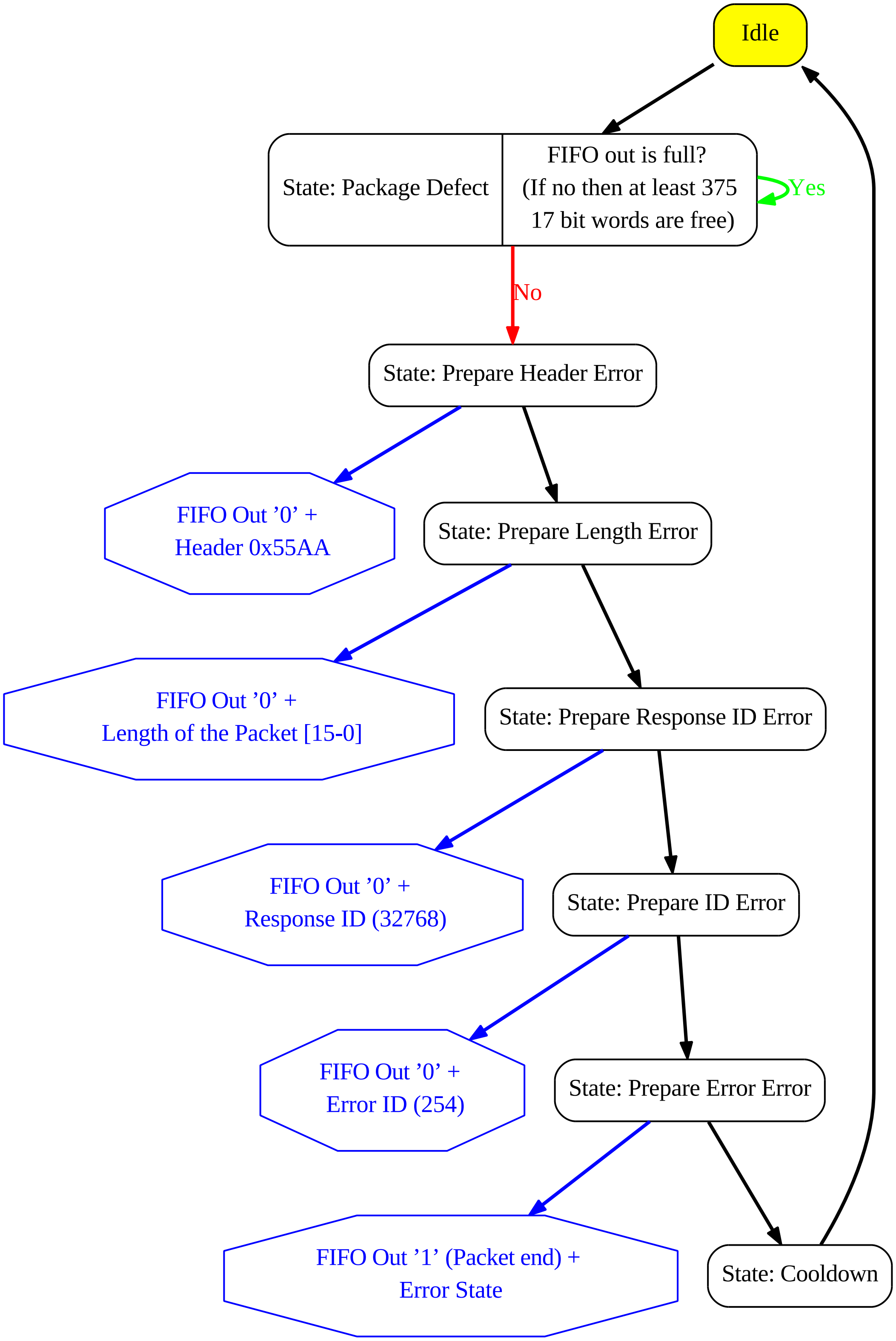

Figure 2.34: 'Data Packet Refurbish' module prepares special network packages if the header is defect. (error handling part)

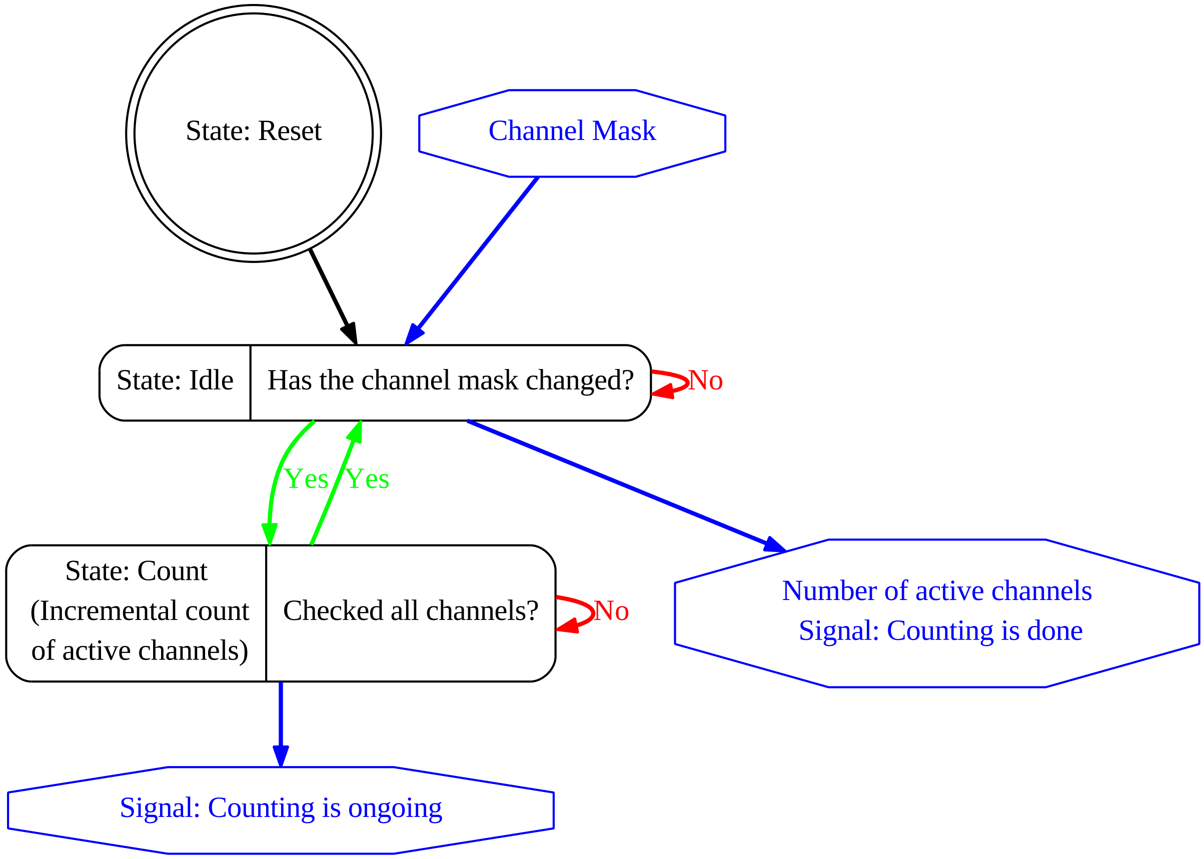

Figure 2.35: 'Channel Mask Counter' module that counts the number of the actual selected channels from the channel mask. This counting process is done automatically every time when the channel mask was changed.

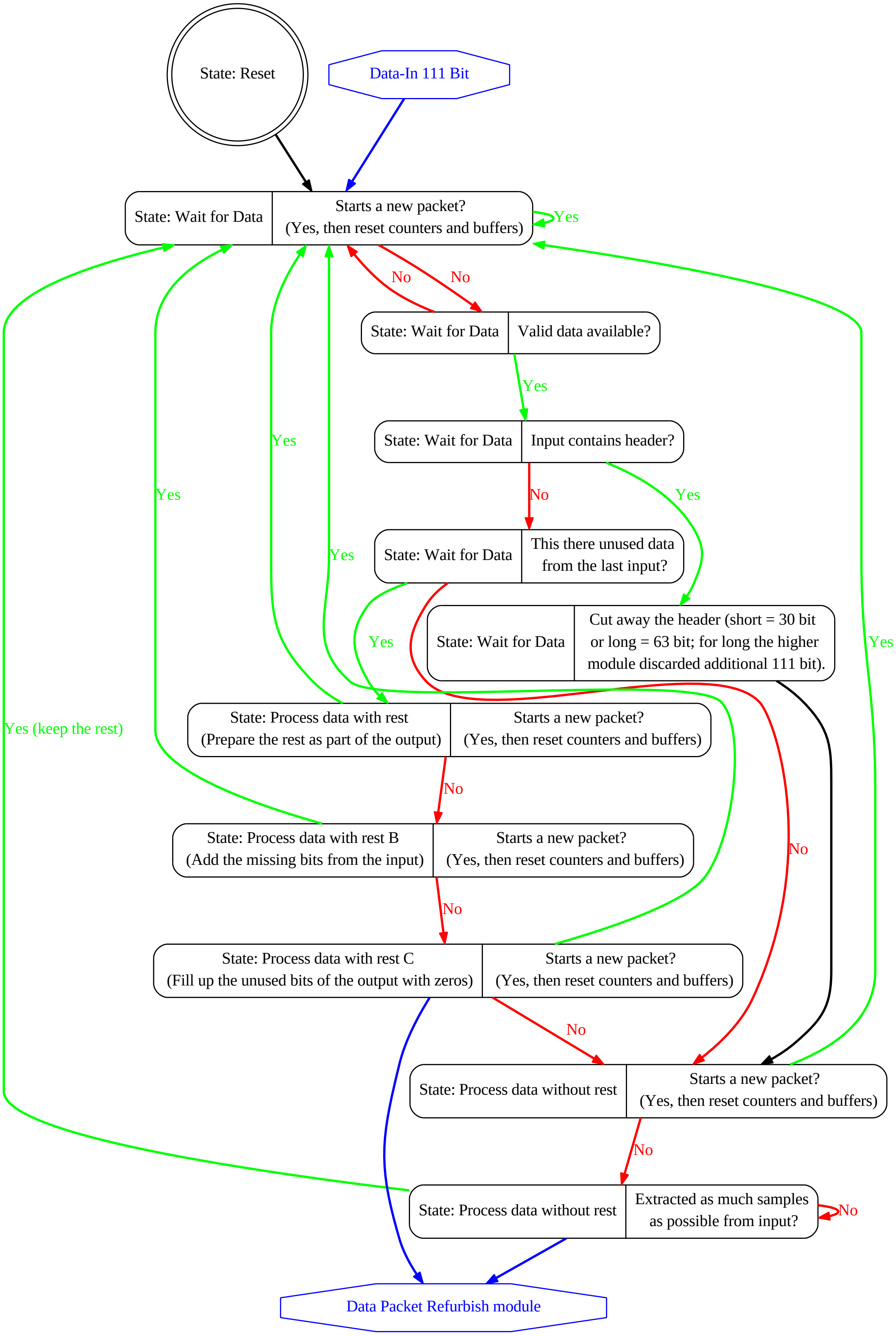

Figure 2.36: 'Stream Decomposer' module converts the samples with different lengths into 16 bit samples.

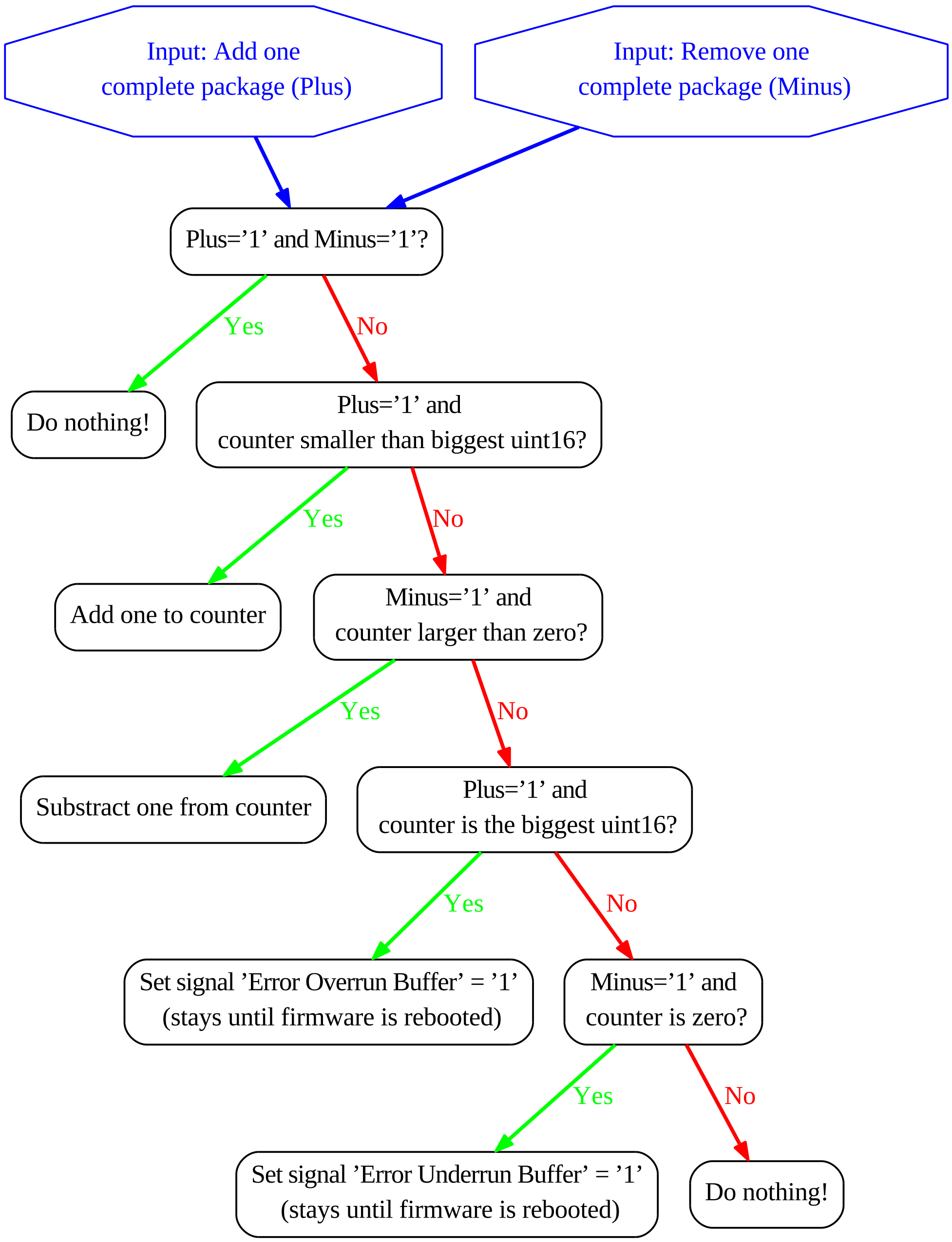

Figure 2.37: 'Network Packet Counter' module knows how many complete packages are in the FIFO.

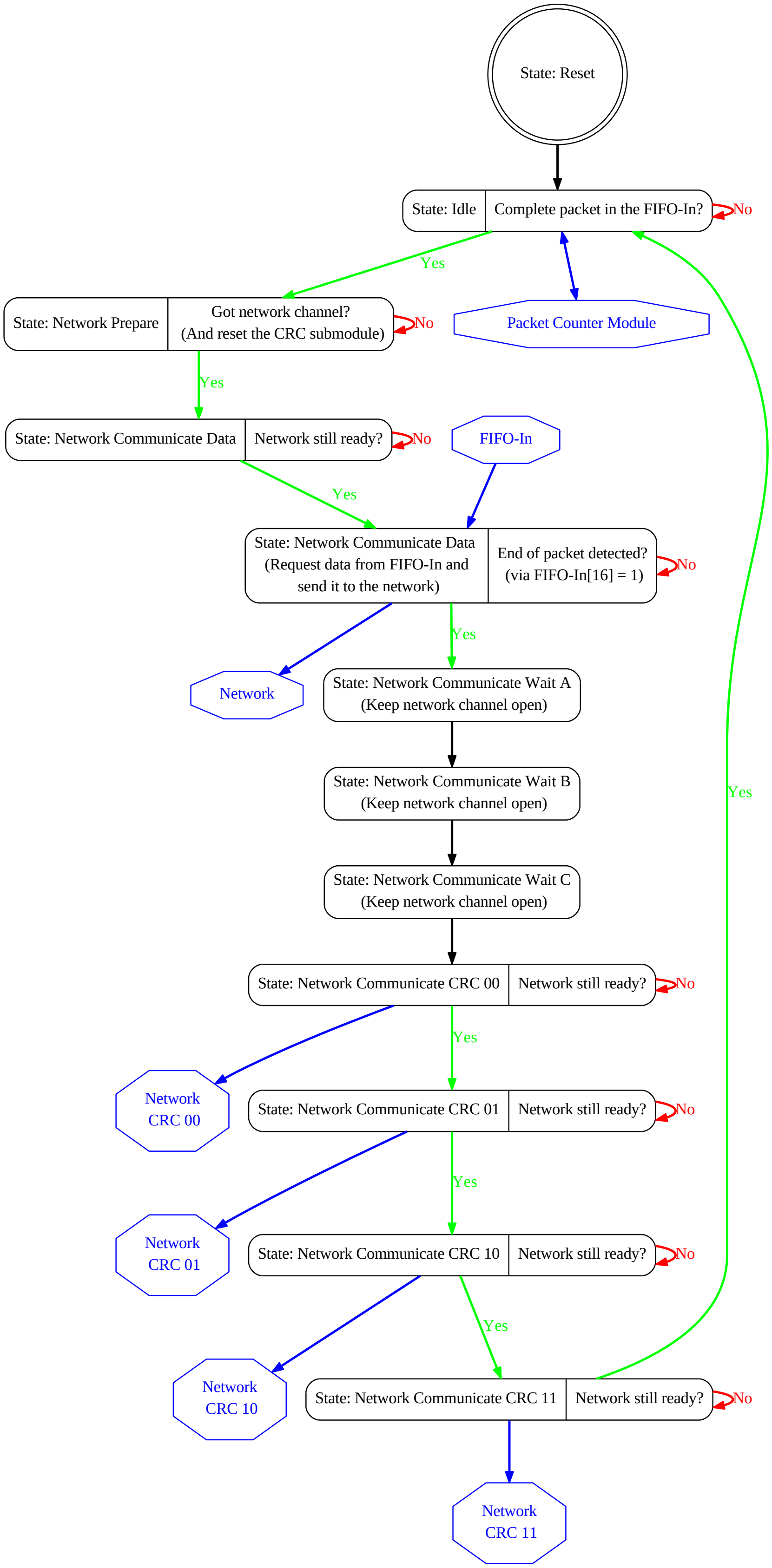

Figure 2.38: 'Network Data Process Interface' module for ASIC and external ADC data is completing the prepared network packages and is transferring them into the outgoing network FIFO.

Test modules

For development purposes regarding the implant's ASIC and Zarlink RF link, I included two test modules ('Test Packet Generator' and 'Test Packet Emitter' module).

The first test module (see figure 2.39) allows to generate 14 byte data blocks of test data like the normal collector module would acquire it over the RF link. This 'Test Packet Generator' module makes it possible to inject such test packages into the data processing chain of the firmware (temporarily replacing the 'Zarlink Data Collector' module). The header and the data structure of the fake packages are exactly like the ASIC would produce them. Instead of using measured data in the pay load, it uses a 8-bit counter, which is updated with every generated test package, concatenated to the ID number of the individual test channel. Then these 16 bit values are cut down to a size that is defined by the selected ADC resolution. This makes it possible to check the data processing on the firmware as well as the software running in the external PC without the need of a real implant.

The second test module (see figure 2.40) also allows to re-route the data packages but for a different purpose. With this module it is possible to transmit the packages via the RF link to another Zarlink IC. The 'Test Packet Emitter' module temporarily replaces in this mode the 'Data Packet Refurbish' module.

The idea behind the combination of these modules is the following: If two base stations are available, then the user can exchange one of the Zarlink base station hardware modules into a Zarlink implant hardware module. The part of the firmware that creates the Zarlink RF connection can be reconfigured by network command packages to use a Zarlink implant module instead of the normal Zarlink hardware. Using this modus, the two base station can connect to each other now. After the connection was established, the 'Test Packet Emitter' module is activated. Followed by an activation of the 'Test Packet Generator' module. Now on the implant simulator, test data is generated by the generator module which is then shipped by the emitter module via the RF link to the base station. The base station collects the test data and processes it. Then the processed data is send to the external PC where the software analysis and displays it for the user. Taken together this modus allows it to test the complete chain and measure its performance (by e.g. changing the parameters of the 'Test Packet Emitter' module). In addition, the simulated implant and the base station are capable of producing very detailed debug information, helping to locate problem in the case they occur.

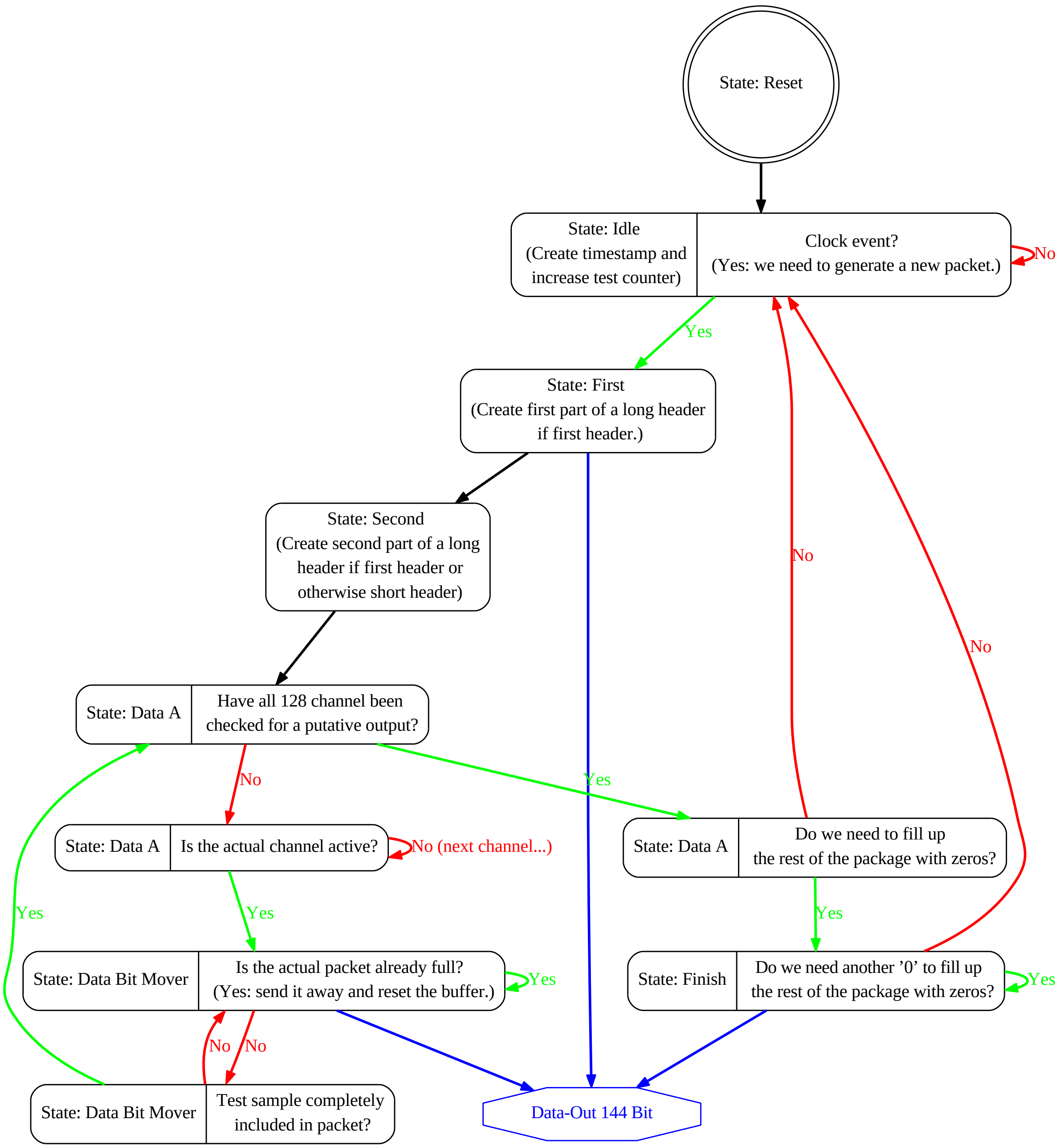

Figure 2.39: 'Test Packet Generator' module produces test packages exactly like the ASIC on the implant is doing it. Instead of measurement data, the module used a simple 8 bit counter and the number of the channel.

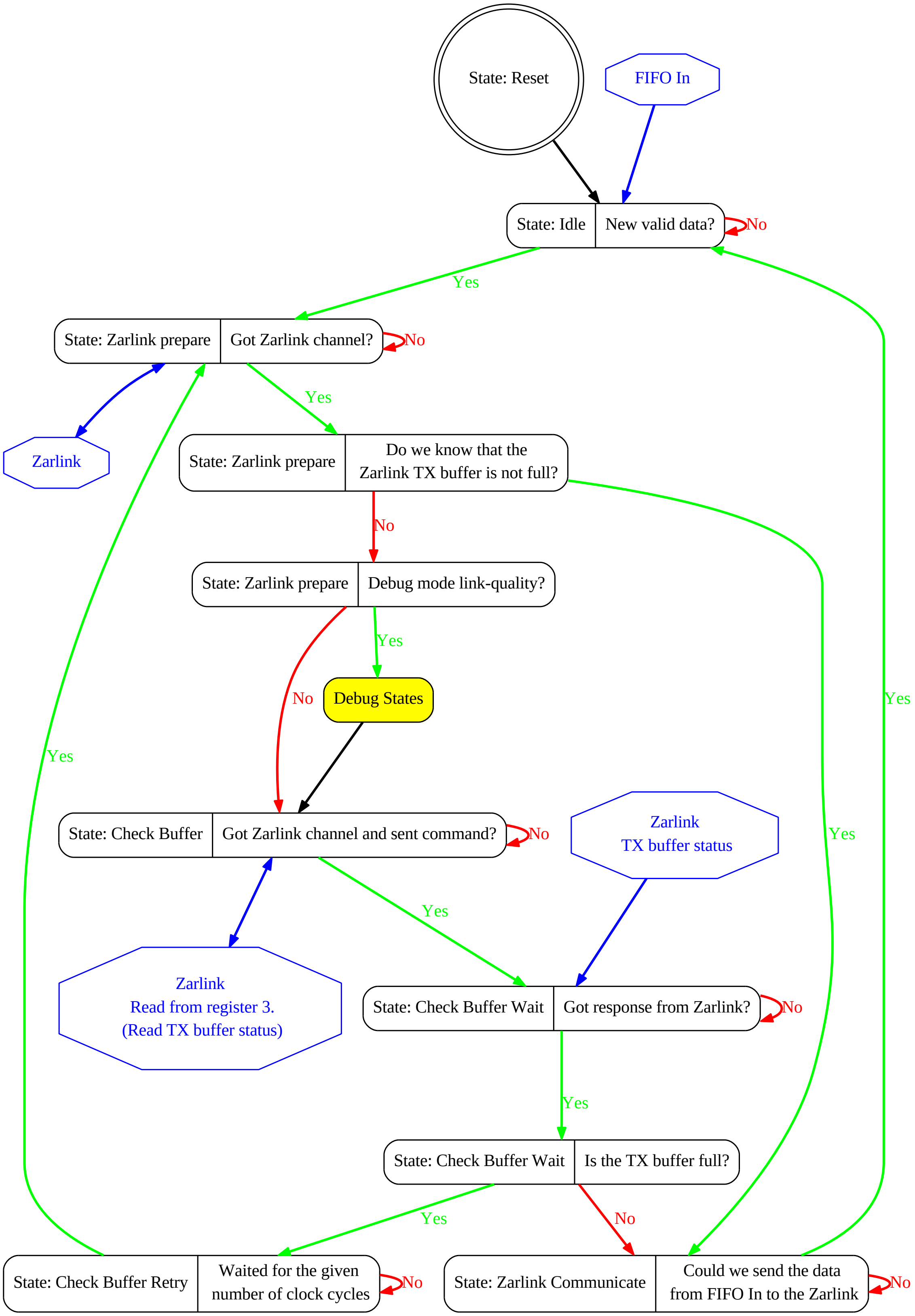

Figure 2.40: 'Test Packet Emitter' module allows to redirect the flow of data within the firmware. It allows to route the data to the Zarlink IC for RF transmission instead of processing and analysing it. The debug part of this module is similar to the one shown in figure 2.30.

2.1.7 Electro-physiological modules

A second way for the base station to collect electro-physiological data is the use of a traditional wire-bound data acquisition system. The analogue signal of the neuronal activity is first amplified and then an analogue digital conversion follows. Depending on the interface between the brain tissue and the electrode, the amplitude of the signal varies. Thus it is helpful to be able to program the amplification factor for the amplifier. A second reason why programming these factors is the application of electrical stimulation. When a electrical stimulation pulse is injected into the brain tissue in the proximity of the measuring electrode, this can result in a saturation of the amplifiers which can prevent measuring for some time. For decreasing the time for which the recording channel can not be use, it is beneficial to reduce the amplification factor of the recording system beforehand.

Taken together the firmware has to manage two aspects of this recording system: a.) control the amplification factor of the individual recording channels and b.) service the analogue to digital converters, collect the resulting digital data, process them and send them to the external PC via Ethernet.

Amplifier

There are many ways to design a programmable amplification for analogue signals. One way is the use of so called digital VGA ICs (variable gain amplifiers ICs). In the beginning of the project, I tried to use such a high-performance IC (AD8370 from Analog Devices). It was very simple to program this IC via SPI. Nevertheless, tests showed that the selected IC created non-uniform amplification factors (and these ICs are kind of expensive). If a sinusoidal signal was sent through the VGA, the amplified version of the sinusoidal signal was steepened compared to the original signal. Since this was not acceptable, in the next version a 'simple' op amp was used. In the feedback loop of the circuit, we introduced programmable resistors (AD5162 8-bit programmable resistor from Analog Devices) for controlling the amplification. These resistors are also programmed via SPI.

Each of these digital resistor ICs (or more precise: digital potentiometer ICs) house two resistors, which can be individually controlled. Depending on the hardware design, it would be possible to handle two analogue channels with one IC. However, in all the designs for this project we used always one IC per analogue channels because of signal integrity concerns and, depending on the version of the hardware design, because there were two programmable op amps per analogue channel necessary.

One important design goal was to change the amplification for each channel individually and independently. With a number of several hundreds of channels (and thus the same amount of digital resistors), the question was how we can address them individually. For selecting which of the available AD5162 is targeted for programming, the 'NOT CS'-

pin ('not chip select'-pins) of the IC has individually to be served by the base station FPGA. The other SPI pins of the AD5162 (like SDI and SClk) can be controlled by common FPGA pins (as long as the driving capacities of the FPGA and speed of the maximal SPI interface of the AD5162 are meet).

In the last version of the analogue-in connector board, I tested two ways of programming the AD5162 ICs. The first version was direct control of all the 'NOT CS'-pins by the FPGA (as well as the corresponding data and clock pins). This direct access was important because this access mode was required for testing purposes. But for a large number of channels this would result in the use of large FPGAs for just controlling all (in the region of 1000) 'NOT CS' - pins. I did this for six channels. For these channels, the 'VGA CS MUX' module, which is just a multiplexer which sets all non selected outputs to a logic high (due to its simplicity it is not presented in more detail), routes the 'NOT CS' port from the SPI module signal to the selected AD5162 IC.

The second version of programming the resistors uses 74HCT595D shift register ICs as mediators between the FPGA and the AD5162 ICs. In the last hardware version within the project, all the SPI pins of 18 resistor ICs are controlled by these daisy chained buffered shift register ICs. As results the number of required FPGA pins for controlling the op amps stays constant at three and does not scale with the number of programmable op amps. This saves FPGA pins and I expect that this design reduces the distribution of high-frequency distortions from the FPGA to the rest of the components on the PCB. For the later reason, the data and clock pins are also handled by the shift registers.

The firmware is written such that it can deal with both ways of managing these resistor ICs simultaneously.

The 'Shift Register IC' module (see 2.41) handles the distribution of SPI data to the AD5162 resistor ICs via the daisy chained 74HCT595D 8-bit buffered shift register ICs. For doing so, it is strongly intertwined with the 'AD5162 SPI' module (see figures 2.42 to 2.47). Not only is the SPI module performing the normal SPI data transfer to the six directly connected channels but it also 'exports' its SPI activity via the 'Shift Register IC' module to the 18 other channels. This makes the SPI module more complex in comparison to normal SPI finite state machines.

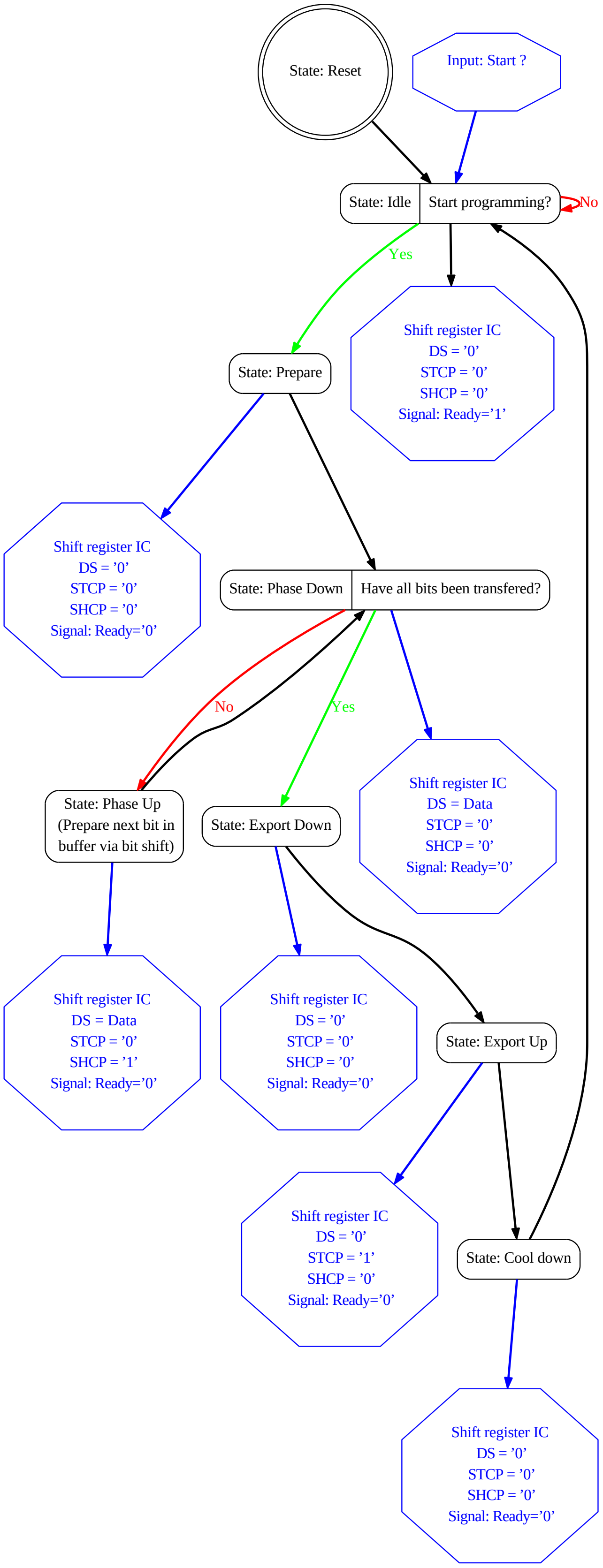

Figure 2.41: 'Shift Register IC' module controls the communication between the SPI module of the firmware and the AD5162 resistor ICs via daisy chained 74HCT595D 8-bit buffered shift register ICs.

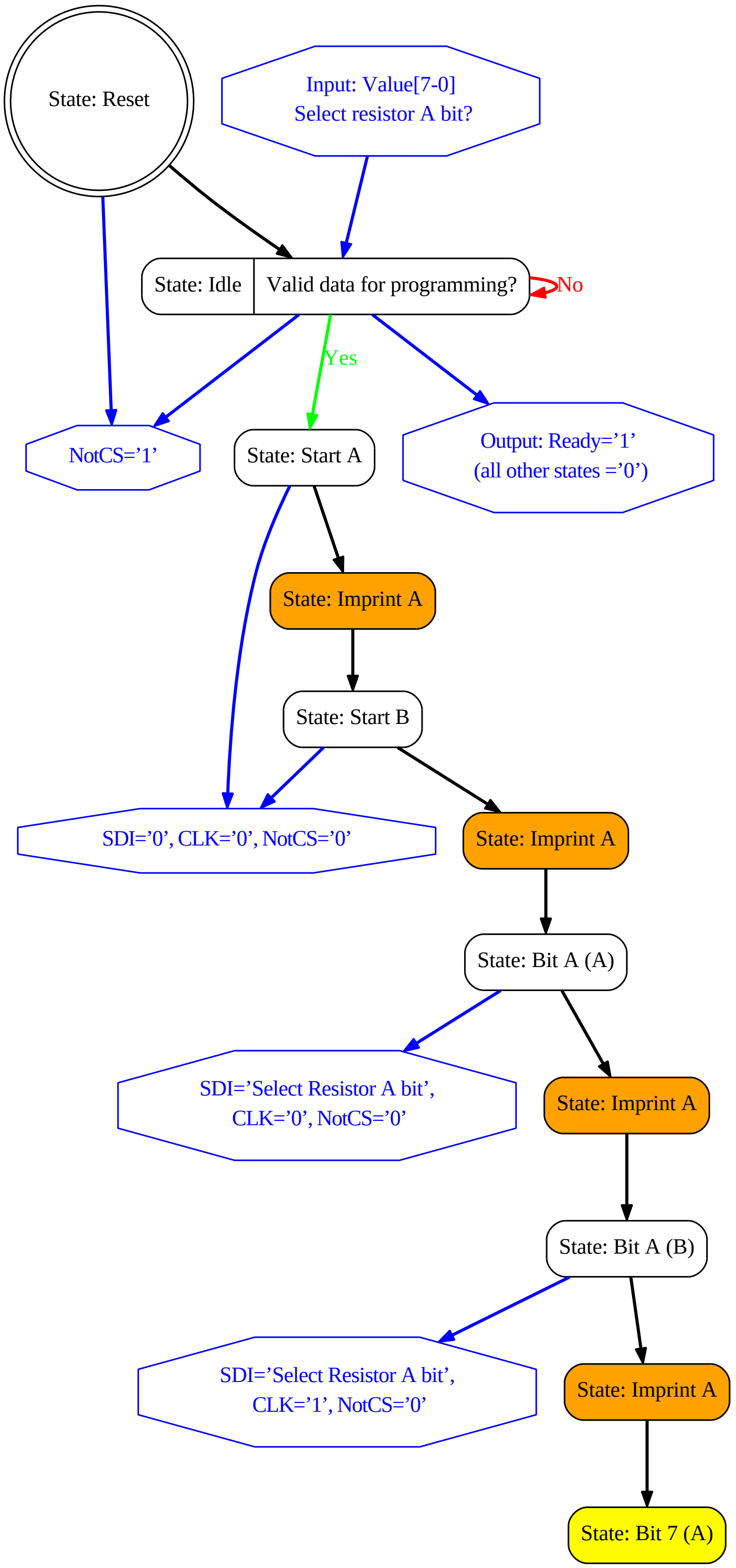

Figure 2.42: 'AD5162 SPI' module for programming the digital resistors via SPI (the orange 'imprint' states are shown in a simplified version. See 2.47 for details on this states.). 1 of 5

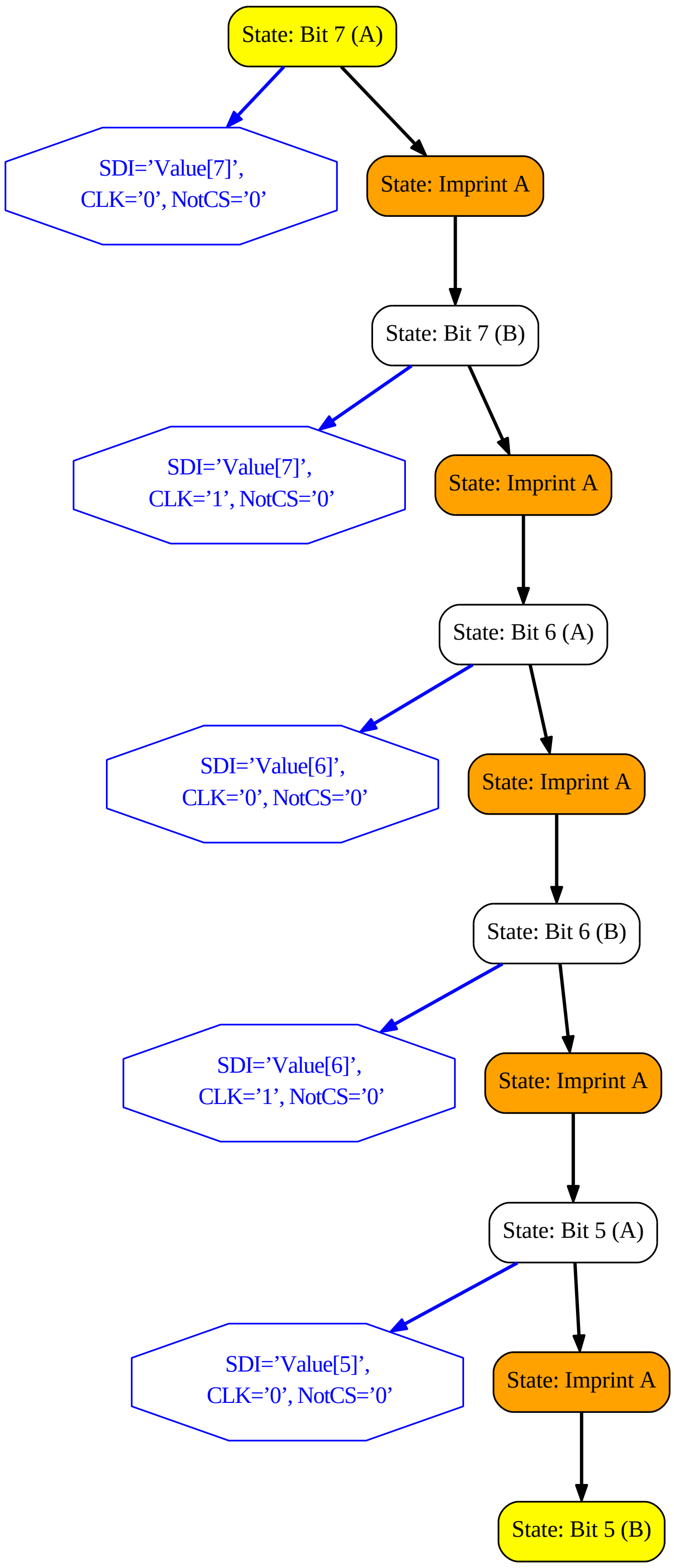

Figure 2.43: 'AD5162 SPI' module for programming the digital resistors via SPI. 2 of 5

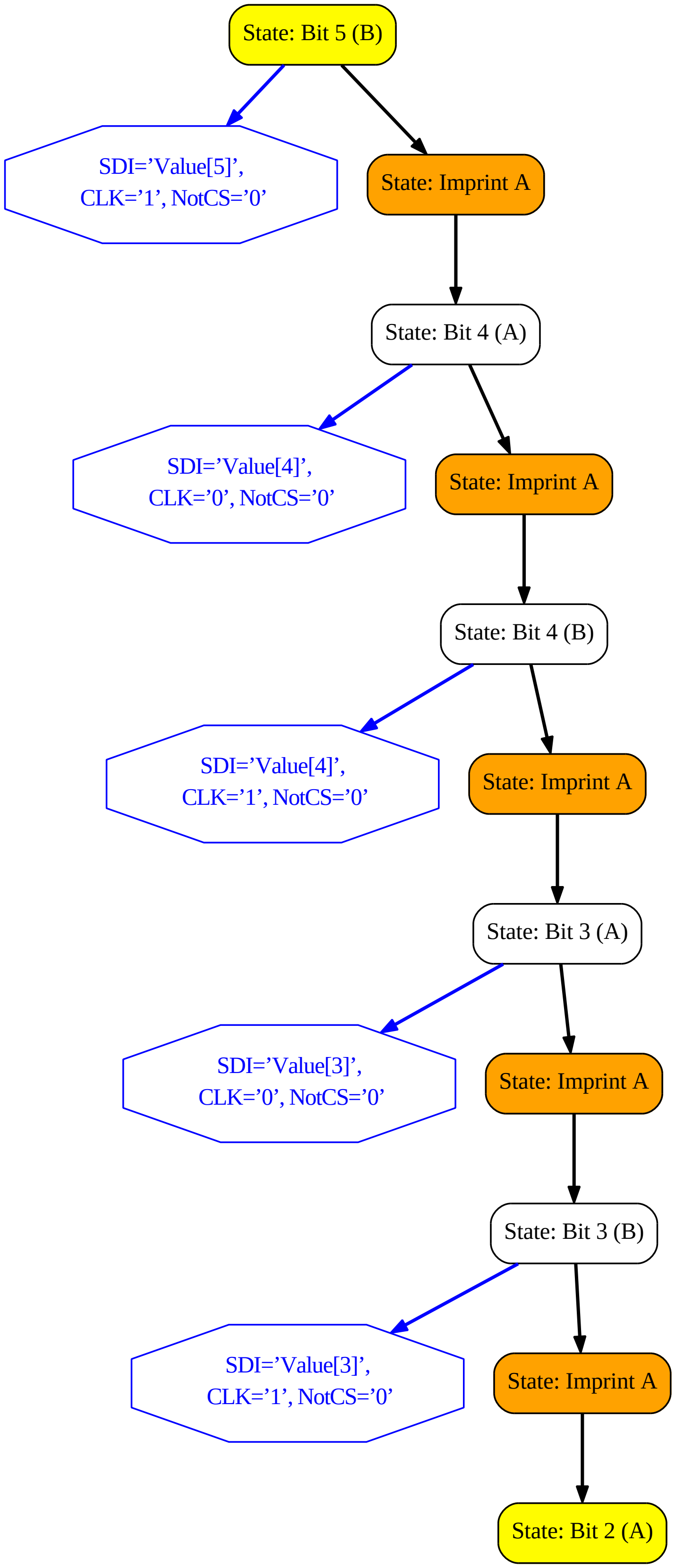

Figure 2.44: 'AD5162 SPI' module for programming the digital resistors via SPI. 3 of 5

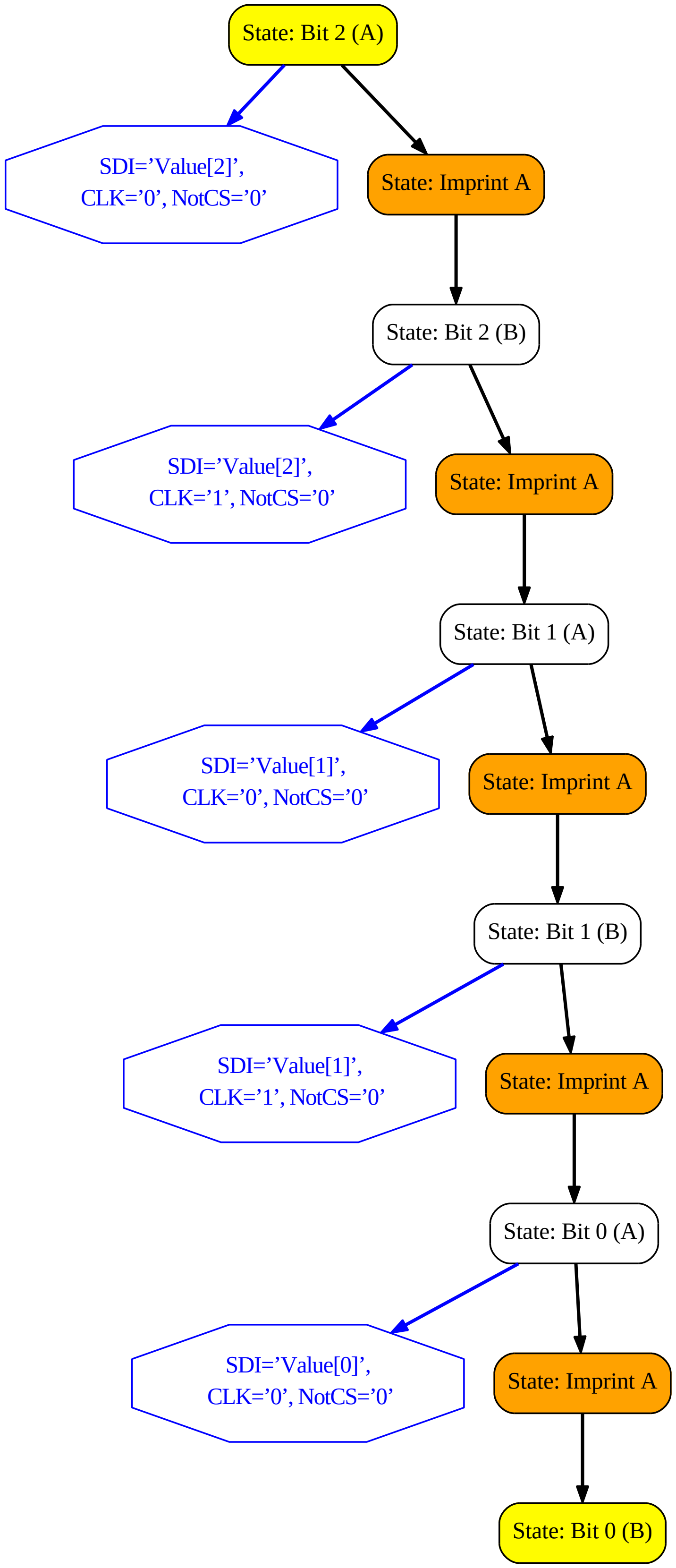

Figure 2.45: 'AD5162 SPI' module for programming the digital resistors via SPI. 4 of 5

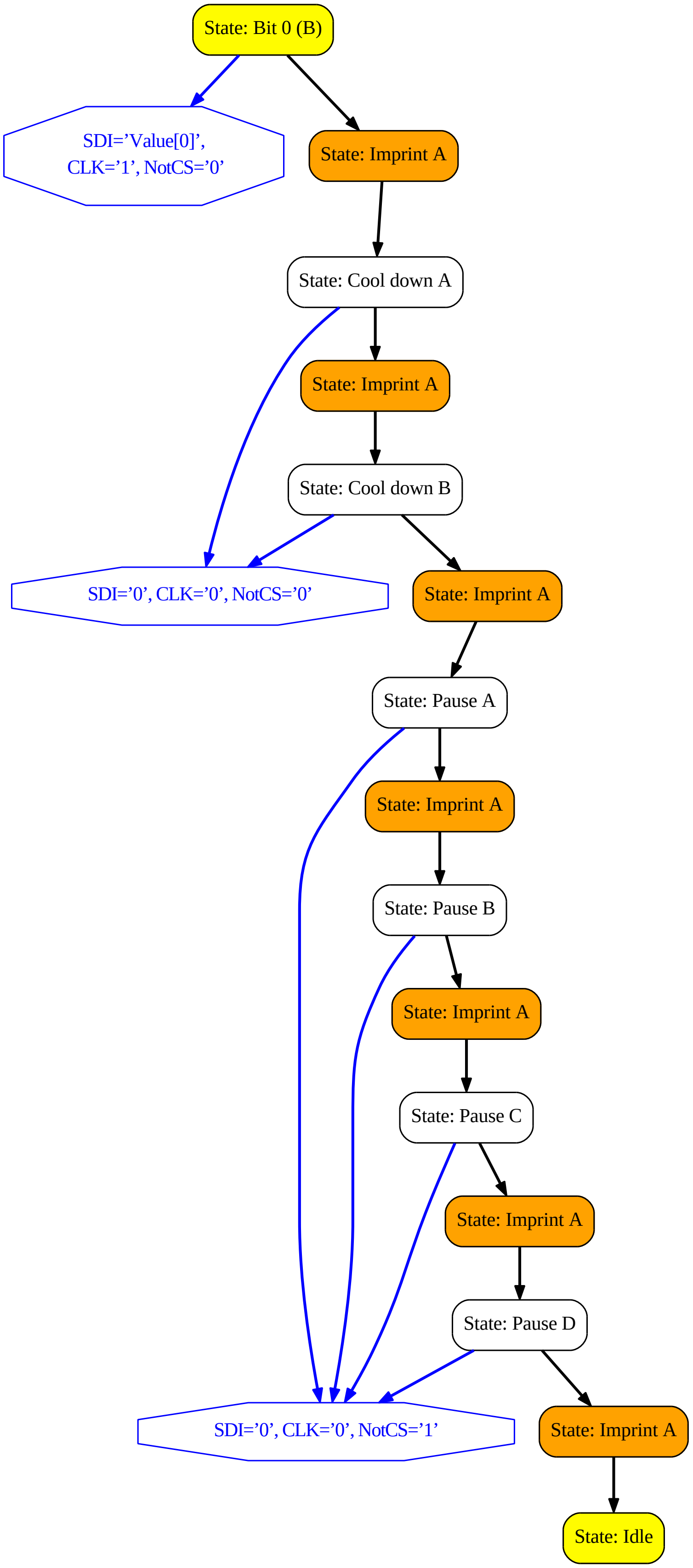

Figure 2.46: 'AD5162 SPI' module for programming the digital resistors via SPI. 5 of 5

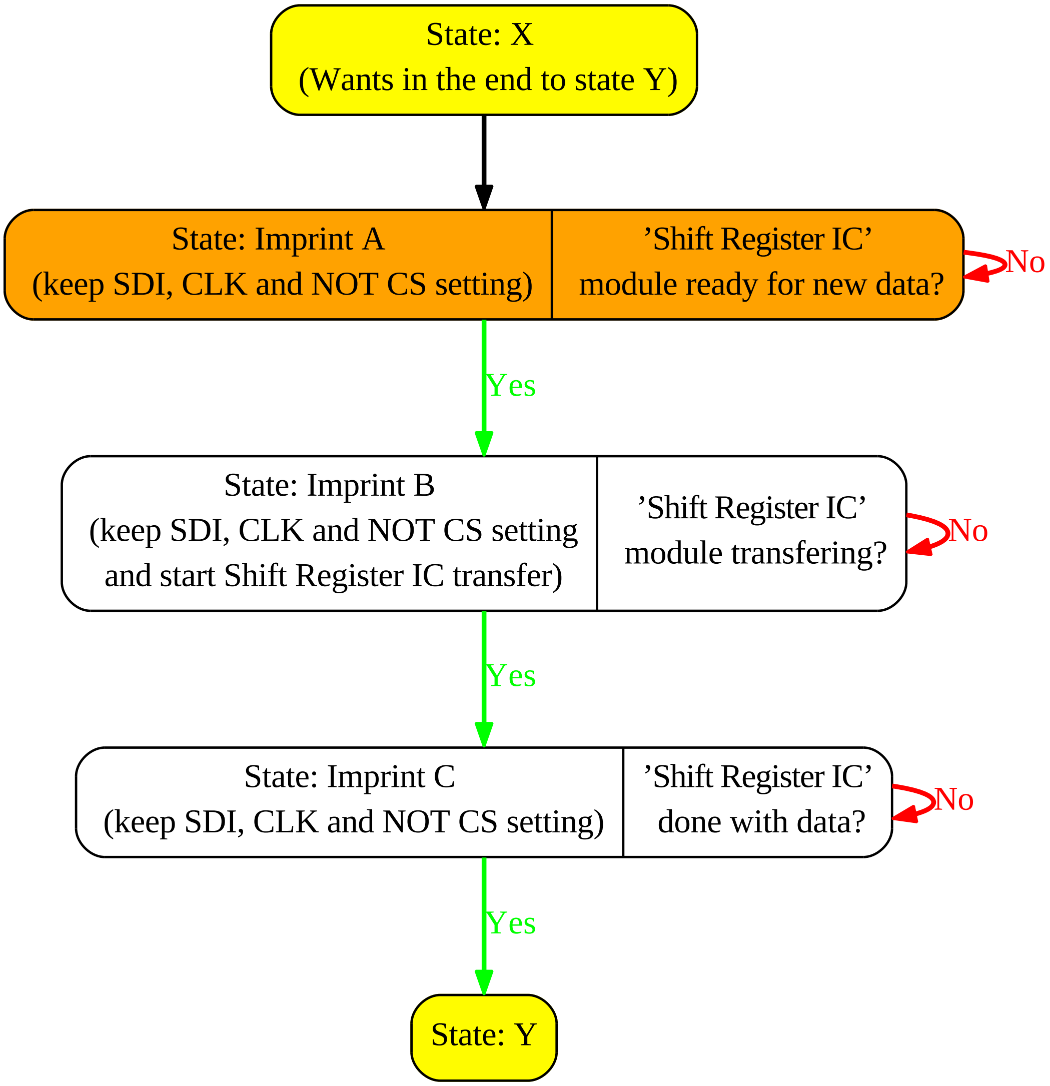

Figure 2.47: ’AD5162 SPI’ module for programming the digital resistors via SPI. The ’imprint’ states of the finite state machine export the actual SPI command to the daisy chained shift registers via the ’Shift Register IC’ module.

Analogue to digital converter

The base station needs to record different types of analogue signals. One type are normal electro-physiological signals. Another type are non-neuronal signals for synchronizing the base station (or the data from the implant) with other devices as well as data about the position and status of the eyes of the test subject (or animal) during an experiment.

All these analogue signals have in common that they need to be digitized with an analogue to digital converter. For the development of the base station, we decided to use a 24-bit ADC (AD7766 from Analog Devices). The idea, behind using an 24-bit ADC, was that this (hopefully) would reduce the requirements for the analogue signal conditioning. Not only did we expect to be able to work with less amplification stages, we also hoped to save bandpass filter stages, due to the integrated digital filter within the AD7766.

Within the firmware the communication between the ADCs and the FPGA is managed by the 'ADC 24Bit Datacollector' module (see figure 2.48). In the last version of the hardware, six channels are bundled. Four of these bundles are available. Within each bundle of channels, six AD7766 are daisy chained. In theory it would be possible to daisy chain more ADCs but each channel needs at least 600k SPI clock cycles per second (24 bits time 25kHz sample rate) plus some clock cycles for handling overhead. In tests (performed by Dieter Gauck, ZKW - Center for cognitive science) it was found that too high SPI clock frequencies can cause perturbations in the digitalization process (random peaks). This creates a trade-off between the number of FPGA pins and the SPI frequency (and the required driving strength). Thus we decided to daisy chain only six ADCs, which allowed me to use as little as 4 mA driving strength and a very low signal slew rate in the QUIETIO mode for the corresponding Xilinx FPGA pins.

The 'ADC 24Bit Datacollector' module implements the SPI protocol for all four channel bundles in parallel and collect four times six 24-bit samples per each sampling incident. These 24-bit vales are converted into 32-bit values and then cut into two 16-bit words, which finally are transmitted to a higher processing module. The firmware assumes that if this type of data acquisition process is used that at least slot one of the ADC connector board is populated. This is important because the SPI module needs information from the 'Not Data Ready' pin about the status of the data conversion process within the ADC IC but the firmware looks (in the actual version) only at the pin from the first slot and assumes that all other three slots behave similar (with minor jitter). In the case of a missing 'Not Data Ready' pin response at slot one, the SPI module will wait for an infinite amount of time.

The 'External ADC Arbiter' module (see figure 2.49) services the 'ADC 24Bit Datacollector' module and collects the data from it. From the incoming stream of samples, the 'External ADC Arbiter' module selects the active channels according to the corresponding channel mask and ignores the other non-relevant samples. The selected

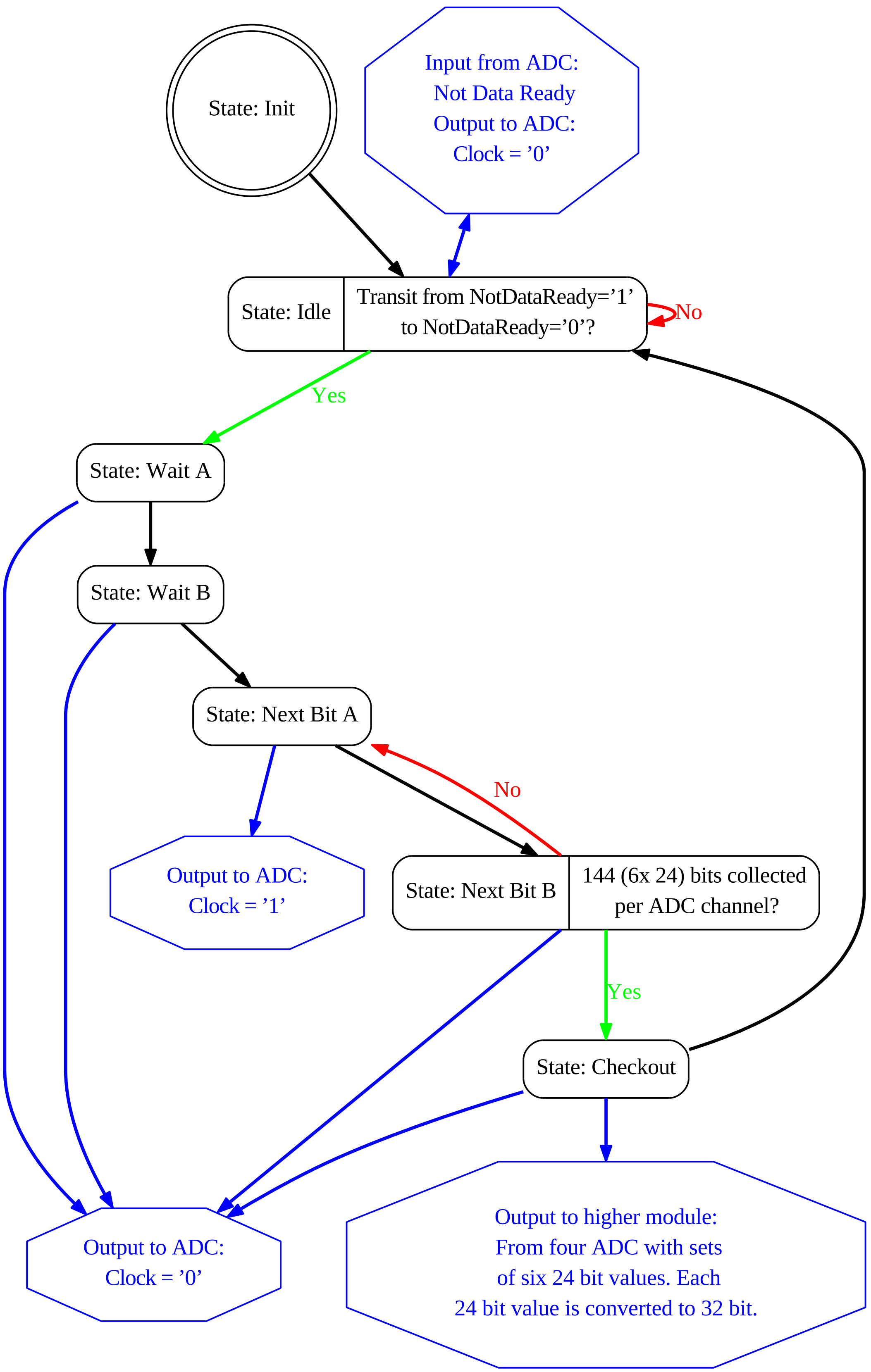

Figure 2.48: ’ADC 24Bit Datacollector’ module handles the communication between the 24 bit ADC (AD7766 from Analog Design) and the FPGA of the base station via SPI.

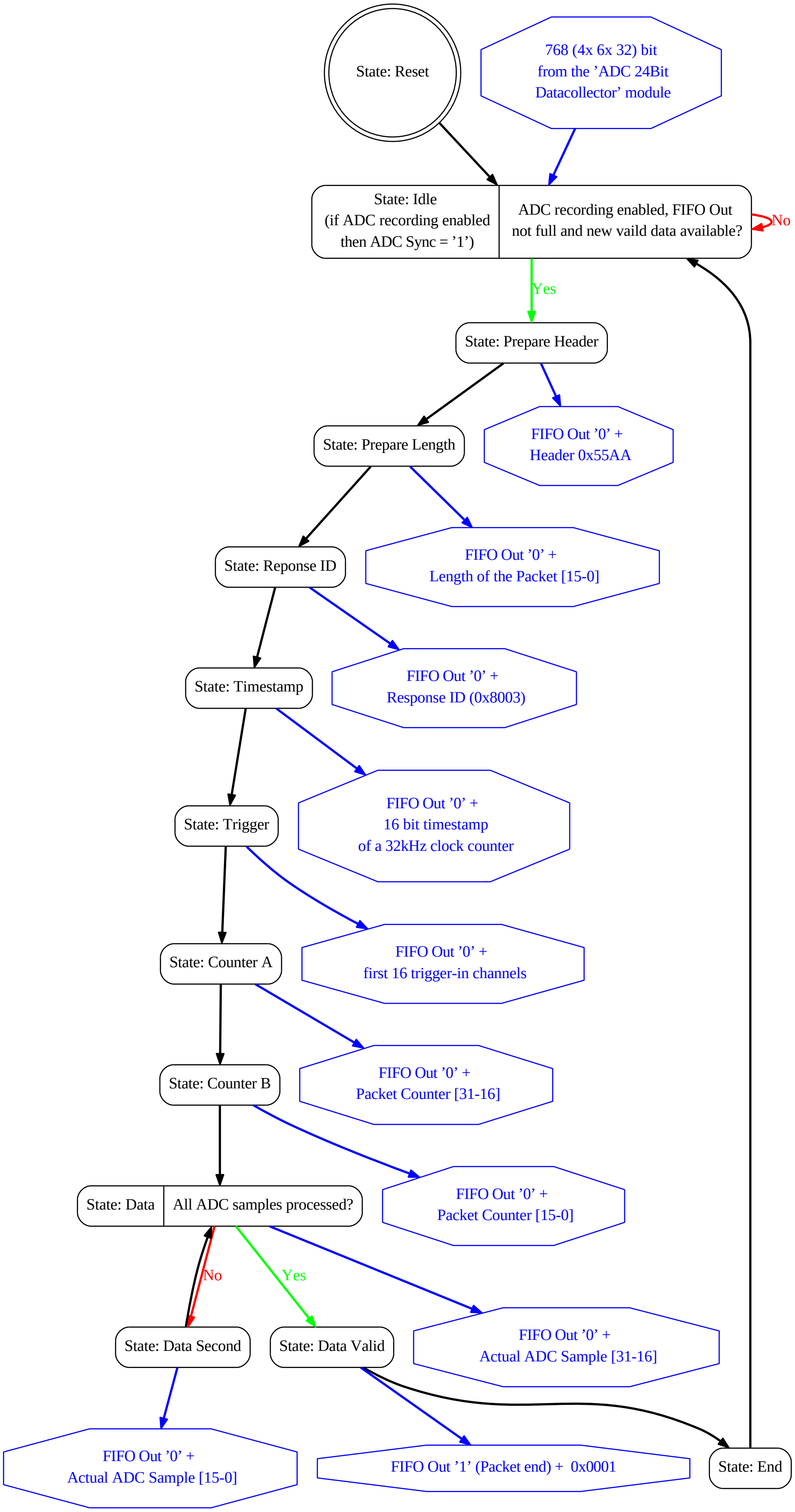

Figure 2.49: 'External ADC Arbiter' module collects the digitalized samples from the ADC and repacks them into preliminary network packages, which are finalized by the 'Network Data Process Interface' module (see figure 2.38).

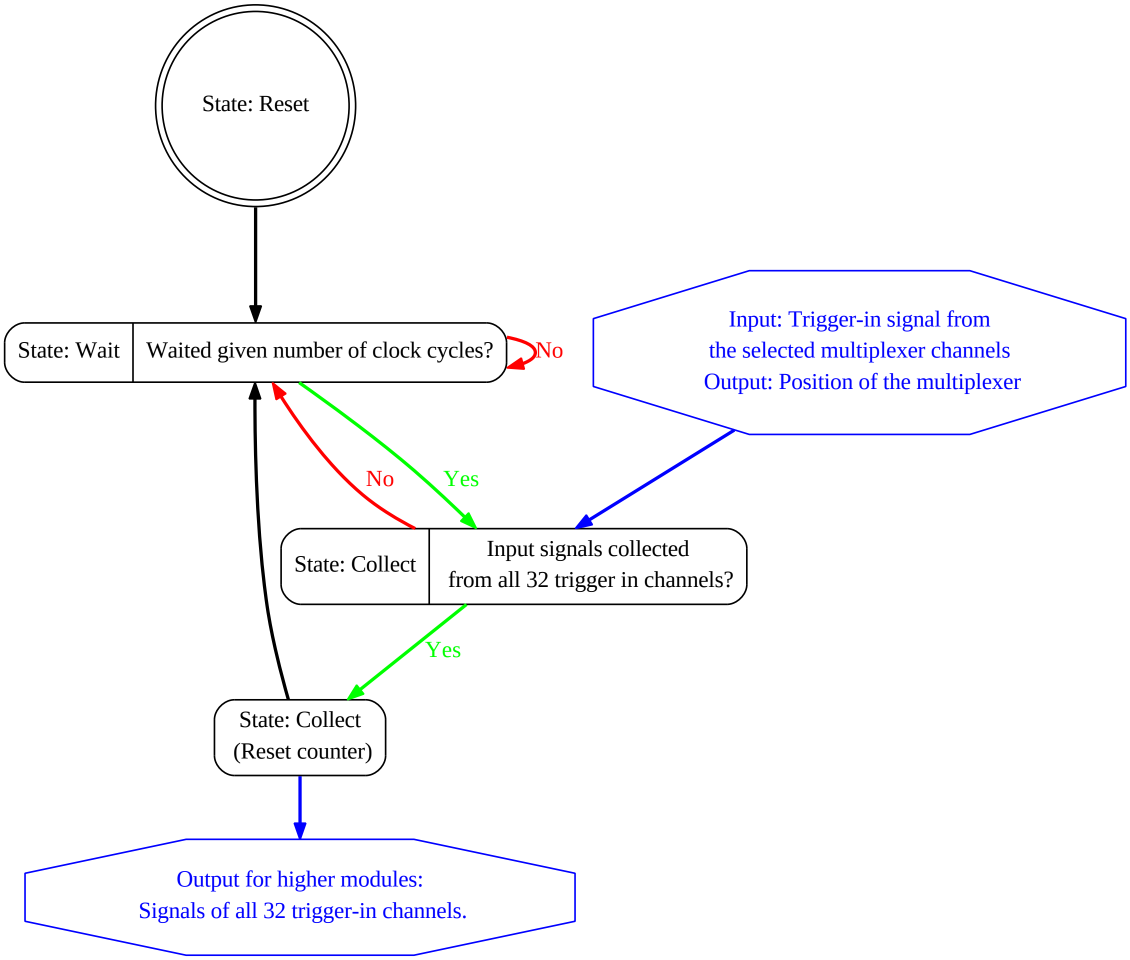

Figure 2.50: 'Trigger-In Multiplexer Control' module checks continuously the status of the trigger-in channels via an ADG732 32-channel analogue multiplexer from Analog Design.

samples are formed into preliminary network packages (without CRC information) and are stored in a FIFO for further processing.

Through a 17-bit wide (the 17th-bit gives information about the end of one set of simultaneously recorded samples) and 1024 words deep FIFO, the 'External ADC Arbiter' module is connected to its 'Network Data Process Interface' module (see figure 2.38) and its 'Network Packet Counter' module (see figure 2.37). Exactly like in the case with the data from the implant's ASIC and RF link, the Network Data Process Interface' module finalizes the network packages and sends them to the network stack.

2.1.8 Trigger channels

Fast binary trigger channels are important for synchronizing other external devices and the base station. We decided that the base station should have 32 ingoing and 32 outgoing channels. It would have been possible to use pins of the FPGA for these tasks but since the actual FPGA has only 80 user IO pins available, I decided to use a multiplexing approach.

For the trigger-in channels I use an ADG732 32-channel analogue multiplexer from Analog Design. The 'Trigger-In Multiplexer Control' module (see 2.50) checks frequently the status of all 32 trigger-in channels. If the input to these channels changes then the internal representation is updated and finally exported to the other modules.

In the case of the trigger-out channels, I use a different (and a bit unusual) approach. In the actual design, the trigger-out channels are buffered with 16 dual D-type flip flops 74AC11074D from Texas Instruments. These keep the desired output fixed as long as they are intended to be constant. If one of these outputs is to be changed then I use two ADG732 32-channel multiplexer for connecting the data and clock pin of the selected d-flip flop with the FPGA. Now the selected D-flip flop can be imprinted with the intended logical state. This design allows to update individual trigger-out channels faster and selectively while all other channels can stay untouched.

However, the actual version of the firmware does only a sequential and complete update of all trigger-out channels. If a selective update is required, this can be realised relatively easy by modifying the responsible 'Trigger-Out Multiplexer Control' module (see 2.51). In the actual version, the module waits until one bit of its inputs changes and then it updates all the channels.

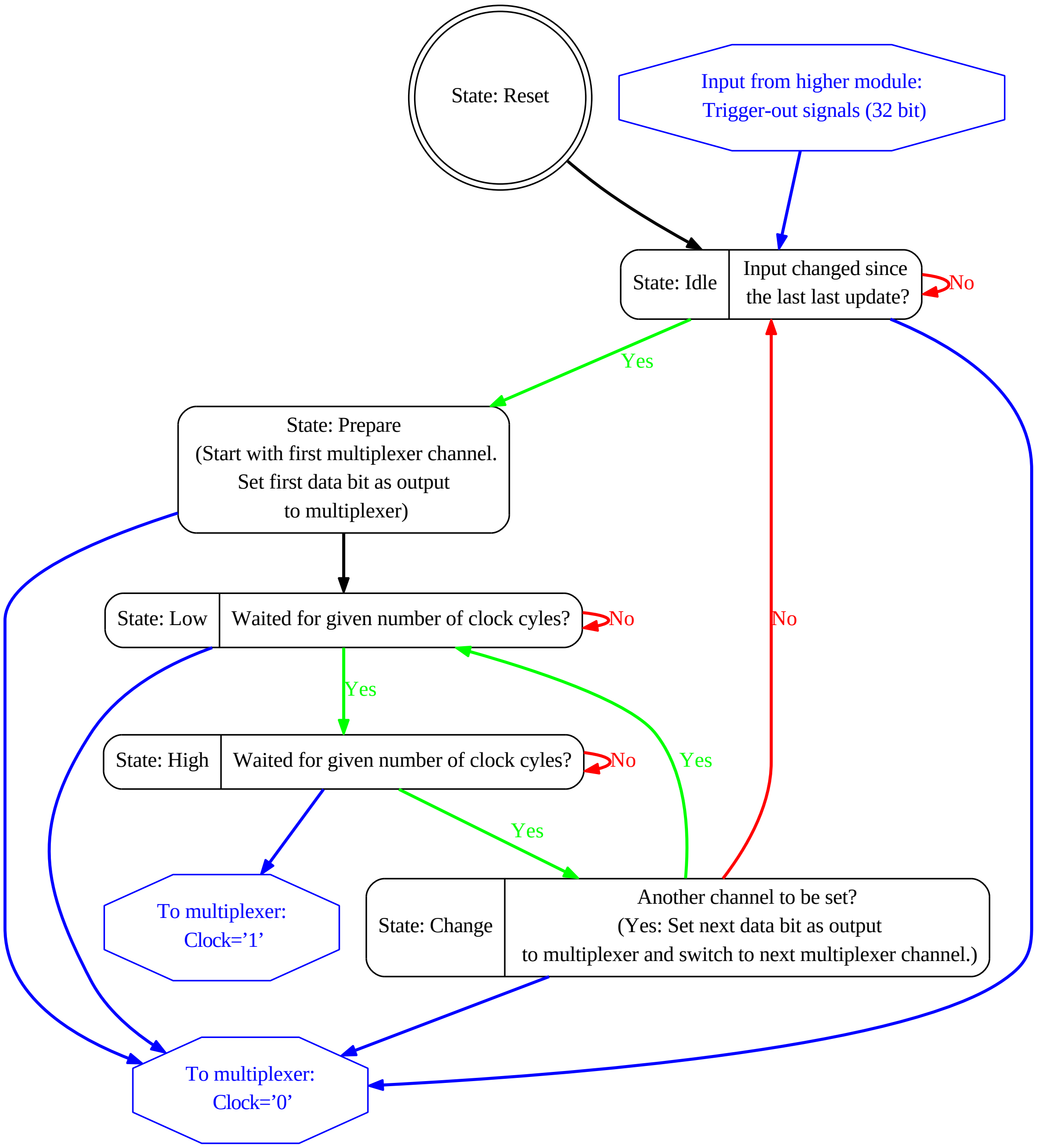

Figure 2.51: 'Trigger-Out Multiplexer Control' module creates and controls 32 trigger-out channels with two ADG732 32-channel analogue multiplexer from Analog Design and 16 dual D-type flip flops 74AC11074D from Texas Instruments.

Chapter 3

Software of the base station

Without the corresponding software, the base station is just pile of electronic components. The concept of the base station is such that an external PC receives the acquired data as well as remotely controls the functions of the base station and the implant given the user's demands. A suitable software running on an external PC needs to communicate with the ZestET1 FPGA via Giga-Ethernet. On the FPGA runs a firmware that defines how the external PC and the ZestET1 have to communicate. It also defines what tasks the FPGA performs and which the PC has to do. I developed, together with Norbert Hauser from Brain Products, the data exchange protocol and the data package structures for the data exchange. It was planned that Brain Products writes a software with a nice user interface. However, it was necessary for me to develop my own set of tools in parallel for testing the hardware and firmware during development. Otherwise each testing cycle would have been too long, due to the necessary communication with the partner. Since it wasn't clear on which operation system I would do the tests, I had to develop a multi-platform software that works under Linux and Microsoft Windows.

In the following I will give a short overview about the software I wrote for this project.

3.1 Programming the FPGA

The FPGA alone has no functionality on its own. When a FPGA is powered up then it sits there and does nothing. For converting it into a high performance data processing system it needs a firmware which tells it how it has to connect its internal components. After turning it's power supply off, the firmware is lost (in most FPGAs) and has to be re-installed on the next powering up process. There are several ways to do that.

One option is to use a so-called JTAG (Joint Test Action Group) cable and special programming software from the FPGA vendor. This even allows to debug and test

the FPGA and its hardware. However, using an extra software during testing disturbs the work-flow. Luckily, the company behind the ZestET1 provides a second way of installing the firmware. As part of the board, a flash memory and CPLD (complex programmable logic device), they program the FPGA during powering up. Orange Tree, manufacturer of the ZestET1, delivers a Windows software for writing the firmware into that flash memory.

For me this wasn't too helpful because I am did my development work under Linux. Thus I asked Orange Tree for information about the programming process because I wanted to write my own tools under Linux for that. Promptly they sent me the complete source code of the Windows programming tool without any questions. With this very helpful information, I started to understand the programming process and converted the Orange Tree software into two multi-platform C++ classes.

The first class handles the Xilinx bit-file. These bit-files are produced by the Xilinx tool chain and contains the information about how the internal components of the FPGA have to be connected. Together with this part, which I call the firmware, the file contains additional information like design name, part name and creation time. My class fragments the file and converts the firmware into a format that the ZestET1 can understand (e.g. reversed byte order for all 32bit data words). The class is also able to show the stored extra information from the bit-file.

The second class is for handling the ZestET1 in the programming process. Within the source code from Orange Tree, I found functions for configuring the FPGA directly (like it is done via a JTAG cable), erasing the Flash memory and write to the Flash memory. For that the CPLD (and it's SPI interface to the FPGA and the Flash memory) had to be controlled with control package sequences via the Ethernet connection.

After three days of programming and testing, I had a working set of multi-platform C++ class, which I can include into my own custom software.

3.2 Monolithic software

Before I defined the data exchange protocol together Norbert Hauser and as result had to rewrite the firmware completely, I wrote a simpler firmware version. The old firmware was a lot less complex and contained only few simple commands for interacting with the Zarlink IC via SPI. To be more precise, the firmware was capable of 14 different commands:
0: Simple read or write operation on one Zarlink registers.
1: Read or write the first byte of a Zarlink data block.
2: Read or write the bytes of a Zarlink data block between the first and the last byte.
3: Read or write the last byte of a Zarlink data block.
4: Write the first 24 bit for setting the clock counter of the auto-poller.
5: Write the second 24 bit for setting the clock counter of the auto-poller.

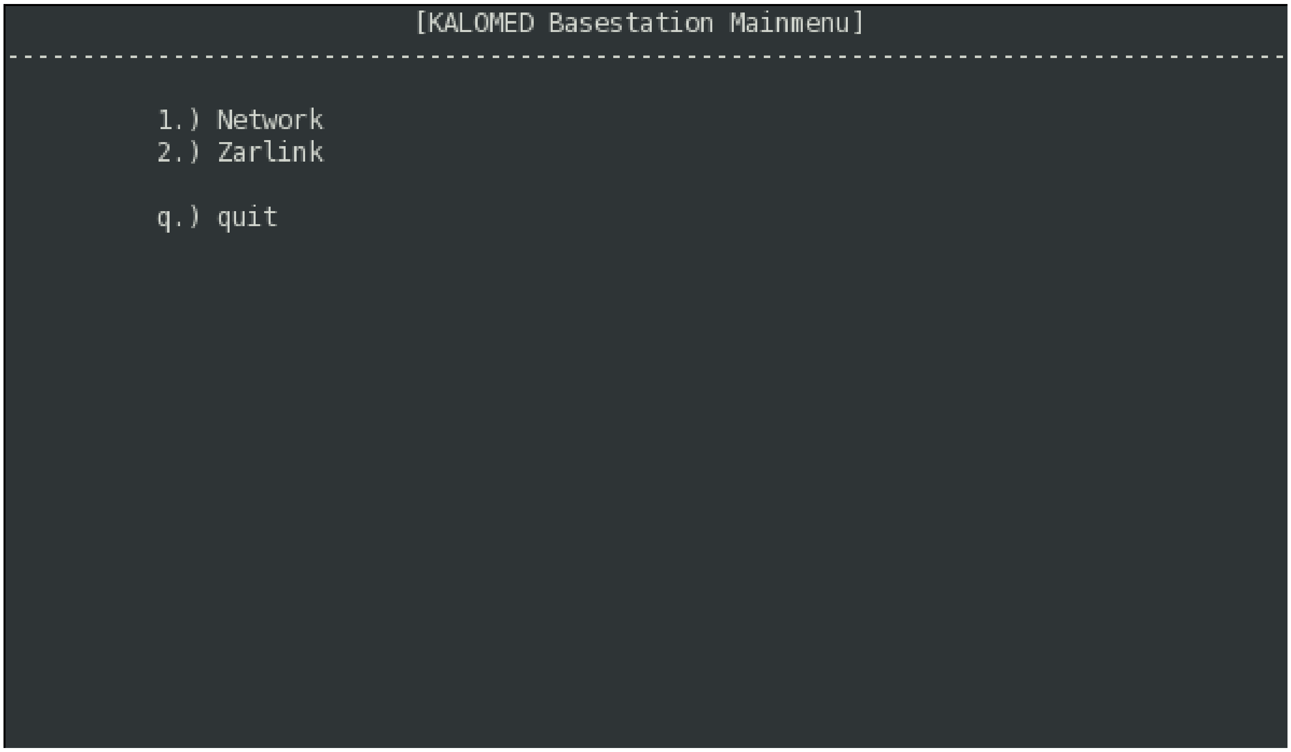

Figure 3.1: Start screen of the software.

6,7,8: The commands are like 1,2,3 but initiated by the auto-poller of the firmware.
9: Auto-poller reads the Zarlink register memory page setting.
10: Auto-poller reads the buffer size from the corresponding Zarlink register.
11: Auto-poller reads the number of filled block in the receive buffer from the corresponding Zarlink register.
12: Sets the speed (1MHz, 2MHz, or 4MHz) of the SPI interface of the FGPA.
13: Auto-poller modifies the Zarlink register memory page setting.

From these 14 commands, the software on the PC is allowed to use 7 commands. The other half is used internally by the firmware. However, the software sees the results of these internal requests.

When a data acquisition process is running then it is essential to download the collected data as fast as possible and regularly from the Zarlink RX (=receiver) buffer. Otherwise the buffer runs full and valuable data is lost. However, this regular data download by an external PC has timing issues. First the software has to check if there is data in the Zarlink RX buffer and then it needs to download it in a byte-by-byte fashion. If e.g. the multi tasking scheduler of the operation system takes too long for re-initiating the software, then data is lost. As a countermeasure, I transferred this data polling into the firmware via an 'auto-poller'. Now it is possible to configure a clock driven module that forces the firmware to check the RX buffer fill status frequently (based on a 48bit value and a 125MHz clock) and downloads all the data from that buffer automatically.

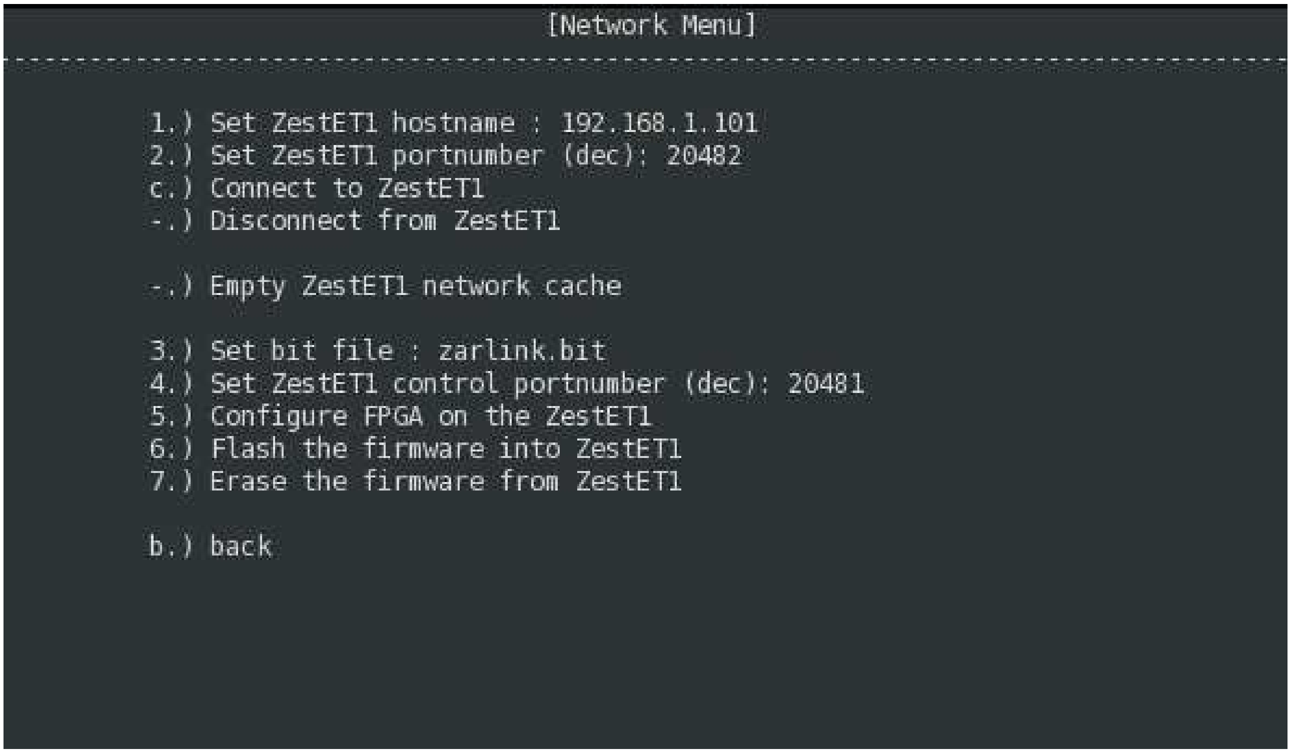

Figure 3.2: Page for configuring the network connection to the ZestET1 as well as configuring and programming the firmware on the ZestET1.

The data is then dumped into the larger ZestET1 network buffer.

When the software gets the data from the network buffer it finds data words marked with the 7 special command IDs. Except the IDs 6, 7, and 8, it ignores all the other ones because the software knows that these commands were not initiated by itself. Within the data stream of 6, 7, and 8 responses, the software finds the beginning of a Zarlink data block, synchronizes to it, decodes the payload from the implant and stores it in a file. Except the help from that auto-poller, the software does everything itself mainly based on the commands 0 to 3.

The software works under Linux (compiled with gcc) and Microsoft Windows (compiled with the Visual Studio). There it runs in a terminal and looks like an old DOS program (for that I wrote operation system specific display libraries). Figure 3.1 shows the start screen of my program. The software is mono-thread and can only perform one operation at a time.

The core of the software is a collection of functions that handle the network connection (e.g. opening, closing, up- and download data, and a command packet builder for the firmware) as well as programming the FPGA (see figure 3.2 for the user interface). Functions like opening and closing a RF connection are done by lengthy software functions based on the simple firmware commands. I also wrote several helper functions

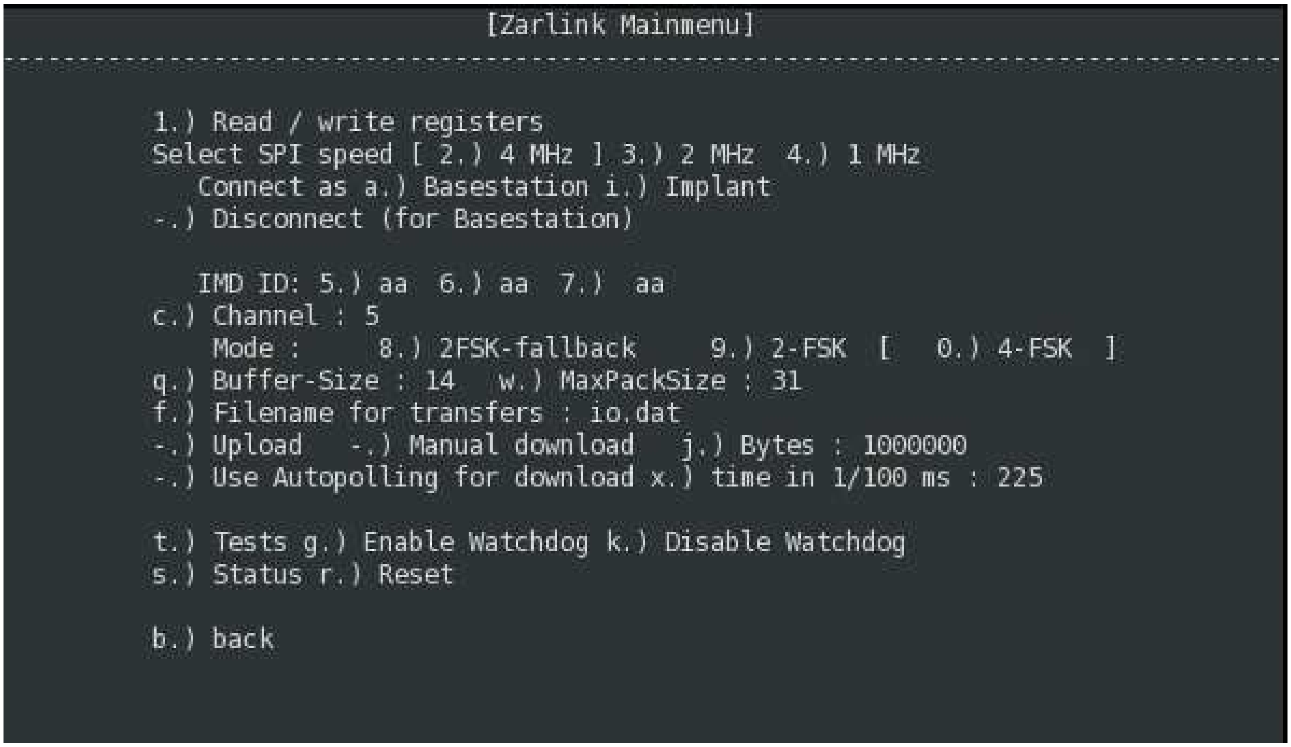

Figure 3.3: Page for configuring and setting up the RF connection and test data transfers.

e.g. for resetting the Zarlink IC, enabling/ disabling the Zarlink watchdog (which removes all the Zarlink setting if no RF connection is established in a given very short time interval), setting the Zarlink's PO3 clock output, setting the Zarlink & firmware SPI speed, and reading & writing Zarlink registers as well as setting the 48bit auto-poller clock. All this functionality is hidden behind a simple user interface (see figure 3.2 and 3.3).

For manipulating the Zarlink registers, I created a database from the Zarlink design manual. Figure 3.4 shows the selection window, which allows to choose by name which register should be manipulated. After selecting the register, the software shows the description text from the manual and handles the read & write properties and number of accessible bits for the individual register (see figure 3.5).

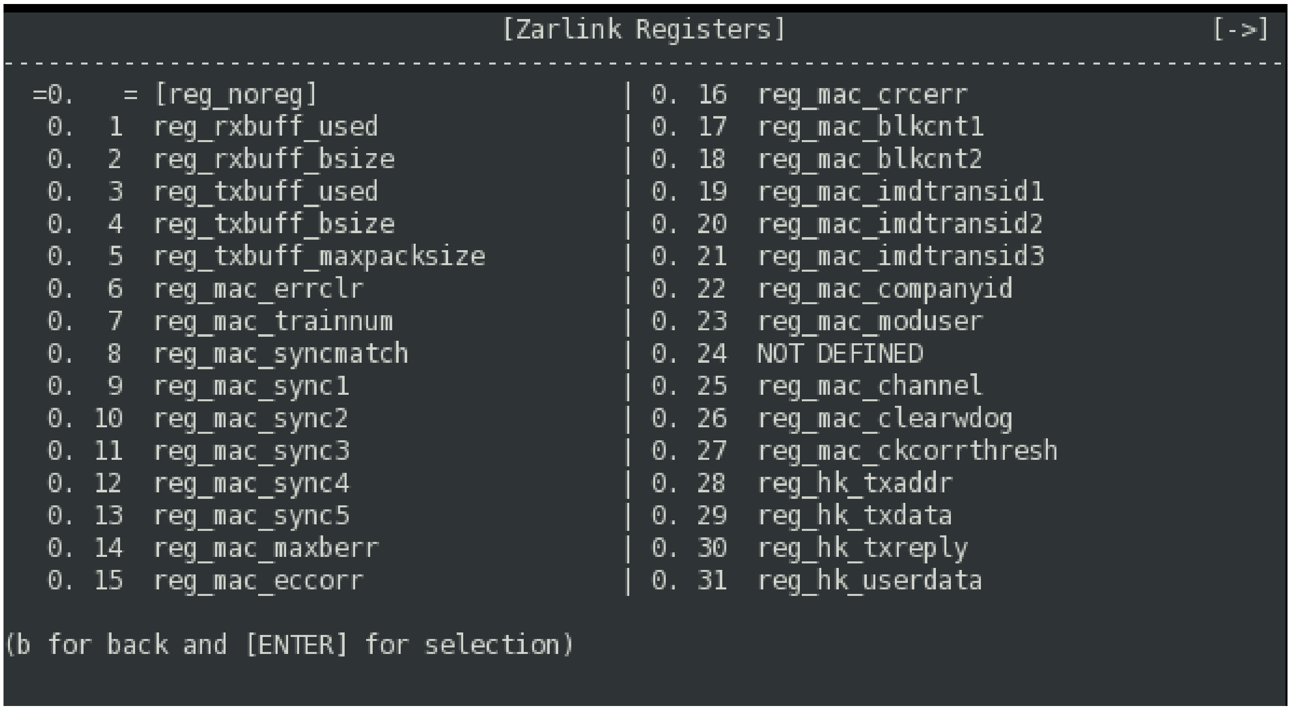

Figure 3.4: Page for selecting Zarlink IC registers.

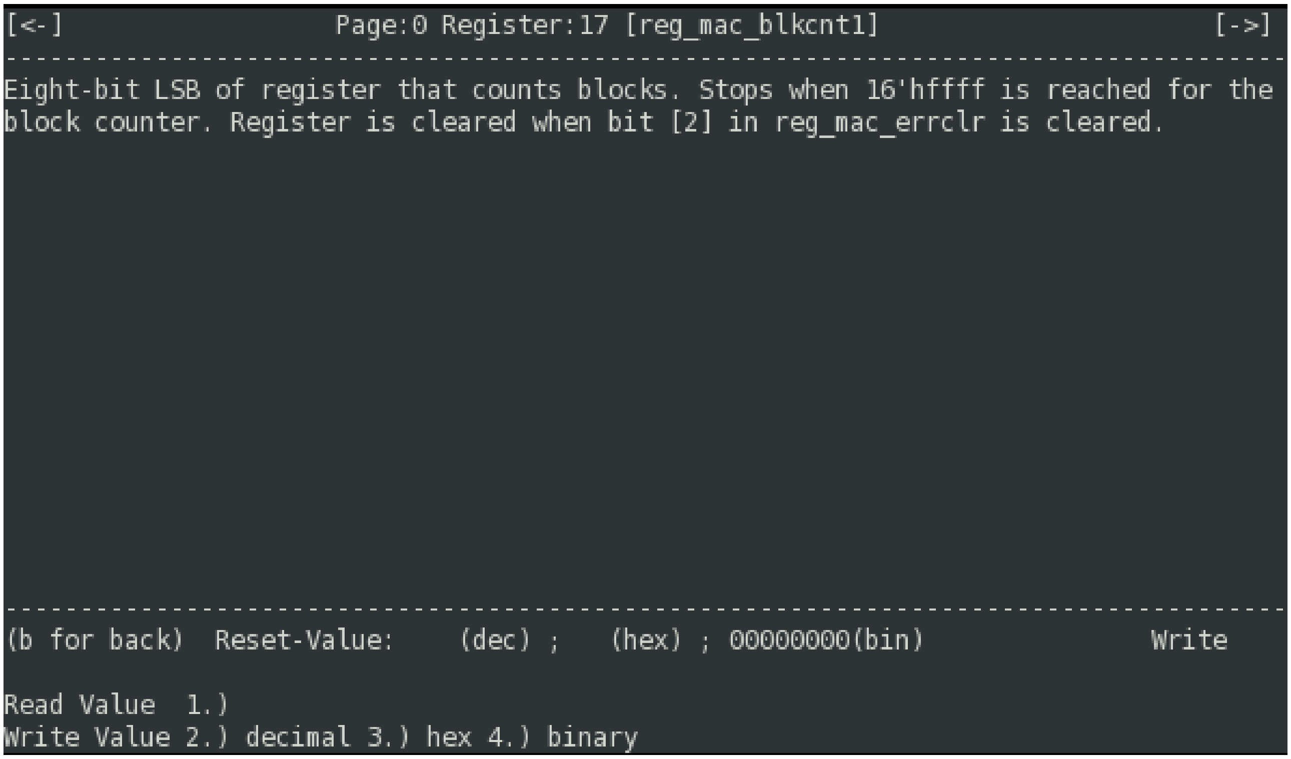

Figure 3.5: Reading and writing the individual registers of the Zarlink IC

3.3 Command line tools

After defining the new more advanced data exchange protocol, I had to rewrite the firmware (which is described in much detail in the previous chapter) completely from scratch. I also had to rewrite the test tools fitting to the new firmware. This time I decided to use simple command line tools instead of a monolithic software. A monolithic software allows to check and control the input of the user much better but command line tools allow to script the tests through a script- or batch-file. This a big time safer if many tests have to be performed.

Form the old software, I could only reuse the network library and the class for programming the FPGA. But since the intelligence and knowledge went into the firmware, the resulting tools are extremely simple. All the tools use the same functions: building the command packages, send the packages via network, reading data from the ZestET1 network buffer which then needs to be analysed.

Analysing the incoming data is also very simple. Depending of the package ID the data is sorted into four files: ASICData.dat(ID 8001, for electro-physiological data recorded by the implant), Zarlink.dat (ID 8002, for debug data from the Zarlink), ADCData.dat (ID 8003, for recorded data from the external ADC channels), and Basestation.dat (ID 8000, for responses to the commands). The four files are simple text files. ADCData.dat and ASICData.dat contain the data for all channels for one sample clock step per line.

3.3.1 List of the tools

In the following I will just give a list of available tools with an extremely short description of how to use them. These tools contain no own intelligence. Everything is done by the firmware. Thus I name the command ID beside the name of the tool. In the former chapter about the firmware, detailed information about the underlying processes can be found.

It is important to understand, that some of these tools write results into the files ASICData.dat, Zarlink.dat, ADCData.dat and Basestation.dat. When a tool is started, it cleans these files. Thus it is necessary to copy these files in advance if the results are required for a later use.

Base station: tool_programming

Configures the ZestET1 FPGA with a given bit-file.

Parameters of the tool:
1. IP e.g. 192.168.1.100
2. Name of the Xilinx bit-file

Base station: tool_debug 0x00FF

Allows to set the debug constants of the firmware.

Parameters of the tool:
1. IP e.g. 192.168.1.100
2. ID of the debug constant
3. New value for debug constant

Base station: tool_trigger 0x0500

Sets the output trigger channels.

Parameters of the tool:
1. IP e.g. 192.168.1.100
2. New value for the output trigger channels (16bit bit mask)

Base station: tool_info 0x0101

Requests information about the firmware version (Basestation.dat will contain the return values).

Parameters of the tool:
1. IP e.g. 192.168.1.100

ASIC: tool_channelinfo 0x0502

Requests information about the ASIC channel setting (Basestation.dat will contain the return values).

Parameters of the tool:
1. IP e.g. 192.168.1.100

ASIC: tool_channelmask_asic 0x0263

Sets the 128 channel of the ASIC.

Parameters of the tool:
1. IP e.g. 192.168.1.100
2. Channel set 01 (8 bit binary mask)
3. Channel set 02 (8 bit binary mask)
4. Channel set 03 (8 bit binary mask)
5. Channel set 04 (8 bit binary mask)
6. Channel set 05 (8 bit binary mask)
7. Channel set 06 (8 bit binary mask)
8. Channel set 07 (8 bit binary mask)
9. Channel set 08 (8 bit binary mask)
10. Channel set 09 (8 bit binary mask)
11. Channel set 10 (8 bit binary mask)
12. Channel set 11 (8 bit binary mask)
13. Channel set 12 (8 bit binary mask)
14. Channel set 13 (8 bit binary mask)
15. Channel set 14 (8 bit binary mask)
16. Channel set 15 (8 bit binary mask)
17. Channel set 16 (8 bit binary mask)

ASIC: tool_start_asic 0x0301

Starts the data acquisition on the implant's ASIC (collected data can be found in ASICData.dat) and collects a given number of samples for each channel.

Parameters of the tool:
1. IP e.g. 192.168.1.100
2. Number of samples for each channel (0 = infinite number of samples)

ASIC: tool_stop_asic 0x0302

Stops the data acquisition on the implant's ASIC.

Parameters of the tool:
1. IP e.g. 192.168.1.100

ASIC: tool_samplerate 0x0273

Sets the implant's sample-rate.

Parameters of the tool:
1. IP e.g. 192.168.1.100
2. New value for the sample-rate

ASIC: tool_resolution 0x0272

Sets the ASIC resolution for one sample in bits.

Parameters of the tool:
1. IP e.g. 192.168.1.100
2. New resolution for the samples. Value n represents n+1 bit resolution with possible values from 0-15.

ASIC: tool_filter 0x0266

Set the ASIC filter parameters.

Parameters of the tool:
1. IP e.g. 192.168.1.100
2. New setting for the filter

Zarlink: tool_init 0x0102

Opens the RF connection (uses 0xAA, 0xAA, 0xAA as IMD ID).

Parameters of the tool:
1. IP e.g. 192.168.1.100

Zarlink: tool_closerf 0x0103

Closes the RF connection.

Parameters of the tool:
1. IP e.g. 192.168.1.100

Zarlink: tool_readblock 0x0405

Reads a 14 byte long Zarlink data block from the Zarlink RX buffer (Basestation.dat will contain the return values).

Parameters of the tool:
1. IP e.g. 192.168.1.100

Zarlink: tool_writeblock 0x0404

Writes a 14 byte long Zarlink data block into the Zarlink TX buffer.

Parameters of the tool:
1. IP e.g. 192.168.1.100
2. Byte 01
3. Byte 02
4. Byte 03
5. Byte 04
6. Byte 05
7. Byte 06
8. Byte 07
9. Byte 08
10. Byte 09
11. Byte 10
12. Byte 11
13. Byte 12
14. Byte 13
15. Byte 14

Zarlink: tool_writereg 0x0402

Sets a Zarlink register on memory page 0.

Parameters of the tool:
1. IP e.g. 192.168.1.100
2. Number of the Zarlink register (0-127)
3. New value for register (0-255)

Zarlink: tool_readreg 0x0400

Reads out a Zarlink register from memory page 0 (Basestation.dat will contain the return values).

Parameters of the tool:
1. IP e.g. 192.168.1.100
2. Number of the Zarlink register (0-127)

Zarlink: tool_writereg_p1 0x0403

Sets a Zarlink register on memory page 1.

Parameters of the tool:
1. IP e.g. 192.168.1.100
2. Number of the Zarlink register (0-127)
3. New value for register (0-255)

Zarlink: tool_readreg_p1 0x0401

Reads out a Zarlink register from memory page 1 (Basestation.dat will contain the return values).

Parameters of the tool:
1. IP e.g. 192.168.1.100
2. Number of the zarlink register (0-127)

ADC: tool_start_adc 0x0303

Starts data acquisition with the external ADC channels (collected data can be found in ADCData.dat) and collects a given number of samples for each channel.

Parameters of the tool:
1. IP e.g. 192.168.1.100
2. Number of samples for each channel (0 = infinite number of samples)

ADC: tool_stop_adc 0x0304

Stops the data acquisition from the external ADC channels.

Parameters of the tool:
1. IP e.g. 192.168.1.100

ADC: tool_channel_adc 0x027A

Selects the channel for the external ADC from which data will be collected.

Parameters of the tool:
1. IP e.g. 192.168.1.100
2. First 16 ADC channels (16 bit binary mask)
3. Second 16 ADC channels (16 bit binary mask)

ADC: tool_setamp 0x279

Sets the amplification value for the programmable external ADC channels.

Parameters of the tool:
1. IP e.g. 192.168.1.100
2. Number of the amplifier e.g. 0
3. New value for the amplification setting 0-255

Matlab: tool_start_adc / tool_start_asic with Matlab

One problem during tests was that we couldn't see the recorded values during the ongoing tests. We had to wait until the data acquisition was done and then we could load the data files into an analysis software and visualize them. This work-flow was not practicable. Thus I modified the original tools responsible for handling the data acquisition process. I wanted to give a real-time like visualization of the incoming data. Thus I added Matlab support to these two tools.

The tools used the so-called Matlab engine extension which starts Matlab as slave. Now the tool can exchange data with its own Matlab session and control it remotely in a way a normal user could do. Hence the collected data was transferred to a ring buffer which I defined in Matlab and plotted the data with the usual Matlab plot tools. The processing of data and plotting them under Matlab can be controlled by several m-files. This makes C++ knowledge for using this tools completely unnecessary.

Parameters of the tool:
1. IP e.g. 192.168.1.100
2. Number of samples for each channel (0 = infinite number of samples)

Chapter 4

The implant

Building an implant is much more complex than designing its base station. Within the Kalomed project sponsored by the BMBF (Bundesministeriums für Bildung und Forschung), we worked on the development of such an brain implant for medical diagnostics and neuro-prosthesis applications. This required specialists from several fields:

1.) Microsystems technology for creating the optimal interface between the implant's electrodes and the brain tissue as well as protecting the implant from the body and the body from the implant. Keeping the system working in such an aggressive environment is far from simple. Alone the long term protection again moisture travelling into the implant and destroying it, is an unsolved problem and subject of intensive worldwide research efforts. Another important topic is the reliable assembly and packaging of the implant as well as the production of flexible PCBs and combining everything in three dimensions for creating the optimally shaped implant.

2.) Custom integrated circuit chip design for optimally using the small available room most efficiently for the necessary electronic components and keeping the energy consumption as low as possible. Due to the small data rate for the wireless data transfer system, it is important that only the relevant data is transmitted. This requires an intelligent control centre which can process the acquired raw data before transmission. It is desirable to build a monolithic custom IC that combines all the necessary function of an implant, e.g. amplifiers, ADCs, RF transceiver, power management, and wireless energy harvesting. However, this is highly cost intensive and due to the different required production processes for us nearly impossible. Thus we based our implant on several commercially available components orchestrated by a self-constructed ASIC (Application-specific integrated circuit).

3.) High frequency transceivers and antennas are required for the wireless RF data exchange. Without an expert in the field of high frequency engineering, it is not possible to build the required antennas, their impedance matching networks, and filter banks. Building an own small and efficient RF transceiver wasn't within the scope of

the project. Hence, we also used a commercial solution from the company Microsemi (formerly Zarlink).

4.) Medical knowledge and European standards & norms for building a system that could be used in a patients in the end.

5.) Testing the implant in a realistic environment, e.g. animal experiments, and using the gained knowledge to improve the implant.

In the following, I will mainly talk about my own contribution for building the Kalomed implant (see figure 4.1) and I will only briefly and partially mention the work of the other contributors. For details see their work and PhD theses:

Microsystem technology (IMSAS Institute for microsensors, -actuators and -systems; Group of Walter Lang): Elena Tolstosheeva, Dmitriy Boll, Thomas Hertzberg, and Darren Gould
Chip design (ITEM Institute of electrodynamics and microelectronics; Group of Steffen Paul): Jonas Pistor and Janpeter Höffmann
HF Design (RF & microwave engineering laboratory; Group of Martin Schneider): Tim Schellenberg
Animal experiments (Brain research institute, department of theoretical neurobiology; Group of Andreas Kreiter): Victor Gordillo Gonzalez

Furthermore, the company Brain Products (www.brainproducts.com) and the department of epileptology at the University Hospital of Bonn (Guido Widman from the group of Christian Elger; www.epileptologie − bonn.de) were also part of the Kalomed project.

First I will discuss the Energy-data-buoy of the implant and then continue with the whole implant itself. For developing the designs, I mainly used the same tools as for the base station. Dieter Gauck managed for me the contact with the involved companies.

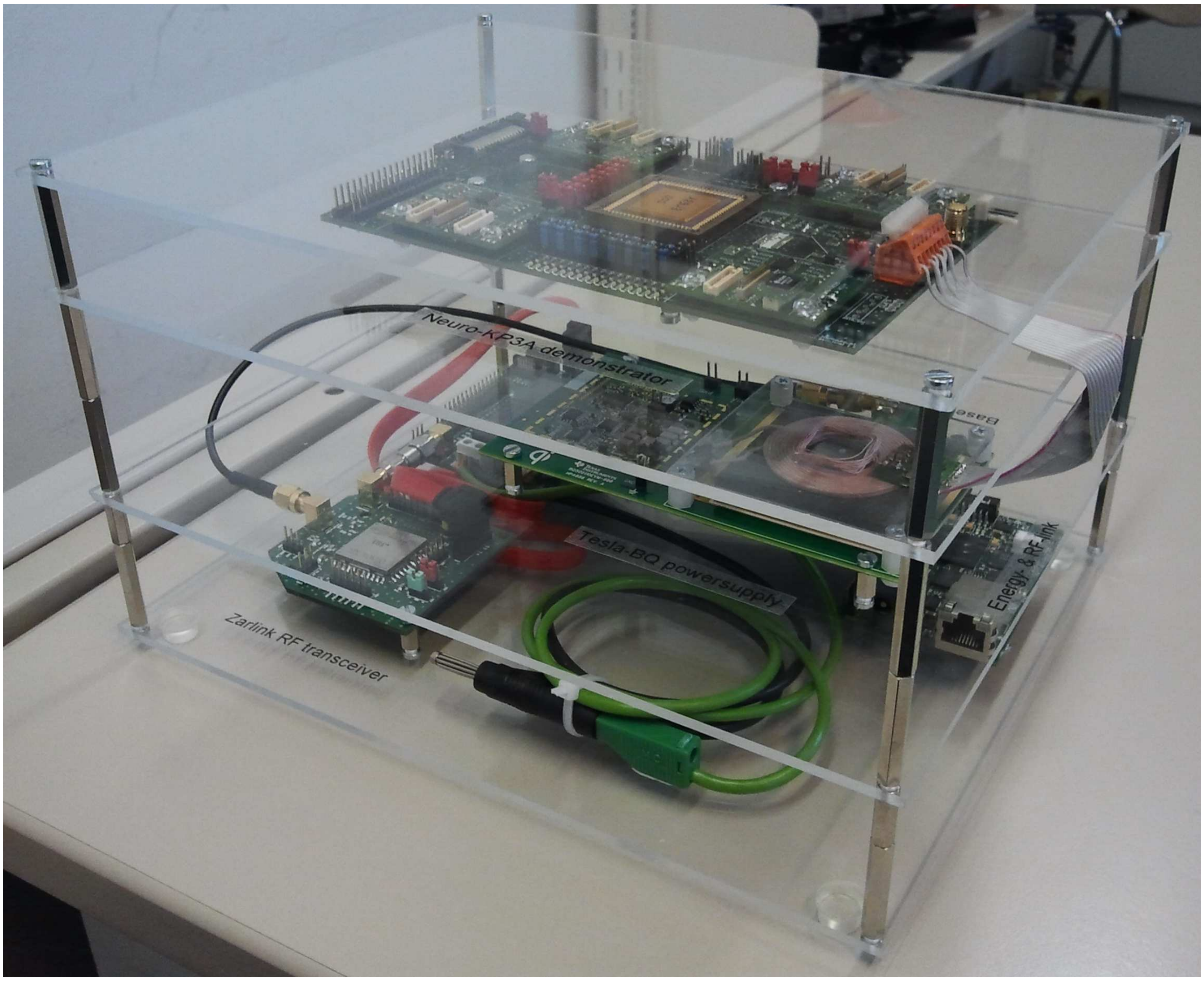

Figure 4.1: Picture of the complete Kalomed implant test system with a minimal version of the base station. Upper layer: Implant test PCB with its ASIC and RHA chips on exchangeable sub-PCBs. Middle layer: The energy-data-buoy is connected via ribbon cable to the implant test PCB. The buoy lies on the Texas Instruments wireless power transmitter. The base station's antenna lies beside the buoy and is connected via a SMA cable to the base station Zarlink transceiver. Lower layer: The base station Zarlink transceiver board is connected via SATA cables to an adapter board. The adapter board is connected via a Samtec cable to the ZestET1 FPGA board. The photo was made by the ITEM (Jonas Pistor and Janpeter Höffmann).

4.1 Energy-data-buoy

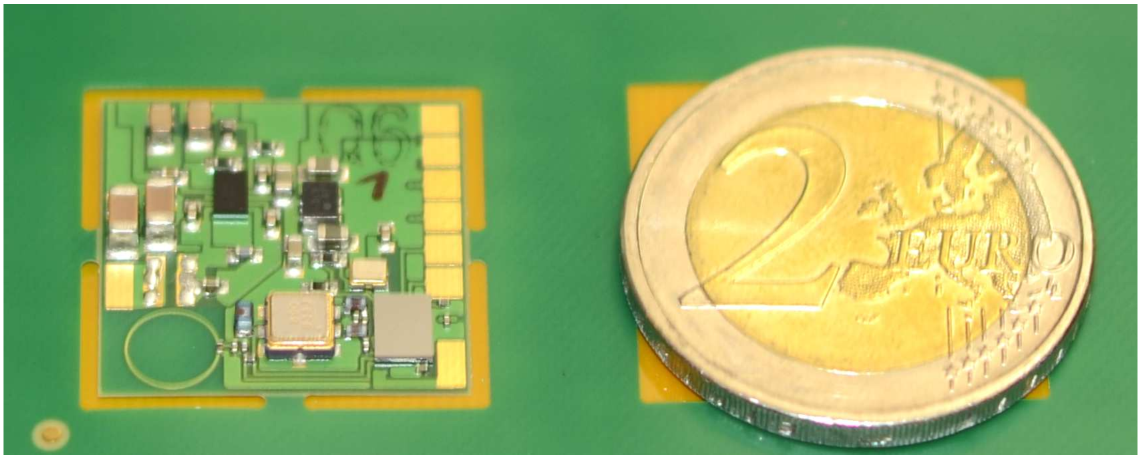

Figure 4.2: Assembled version of the energy-data-buoy. Upper left IC is the TI energy receiver. Right of it, the DC/DC converter. From left to right: Tim Schellenberg's antenna, first part of the matching network, SAW filter, second part of the matching network, and finally the Zarlink ZL70102 IC. Above the Zarlink IC, the 24 MHz clock is located. The energy coil and it's matching capacitor are not installed yet (most left pads above the antenna). These PCBs were assembled by Taube (Berlin).

How to solve the wireless RF data exchange was clear after Tim Schellenberg selected and tested the RF transceiver ZL70102. After that we integrated the Zarlink IC into our system concept and we started to support it on the implant side (ASIC) and base station (FPGA). A big open question was how to get the necessary energy into the implant for operating it and how to regulate the voltages. During the lifetime of the project, we tested several possibility. In the end, I developed an energy-data-buoy (see figure 4.3) which combines the RF implant transceiver and energy harvesting on one small PCB (20mm x 20mm). I constructed the energy-data-buoy such that it can universally be used for all kinds of implants. It provides a 3.3V power rail and a SPI interface for the Zarlink IC with its antenna, Saw filter, and matching networks as well as a 24MHz clock for whatever is connected to it (see figure 4.2).

After I finished the design, the company Andus Electronic ($www.andus.de$) manufactured the PCB (see figure 4.4). In that stage we wanted to test the functionality of the buoy without extra risks. Thus we decided to produce it on hard FR4 substrate with only 0.15mm thickness, nickel & gold contact surfaces, and 0.035mm thick cooper conductors under solder resist. To make the production of the PCB even more complex, it was necessary to fill all VIAs (tube shaped connection between two layers through the substrate of the PCB) and close them with an very even surface of nickel & gold. This was important because one of the ball-grid array ICs required VIAs directly under its balls. The company Taube Electronic ($www.taube-electronic.de$) assembled the board with its very small components and ball-grid array ICs.

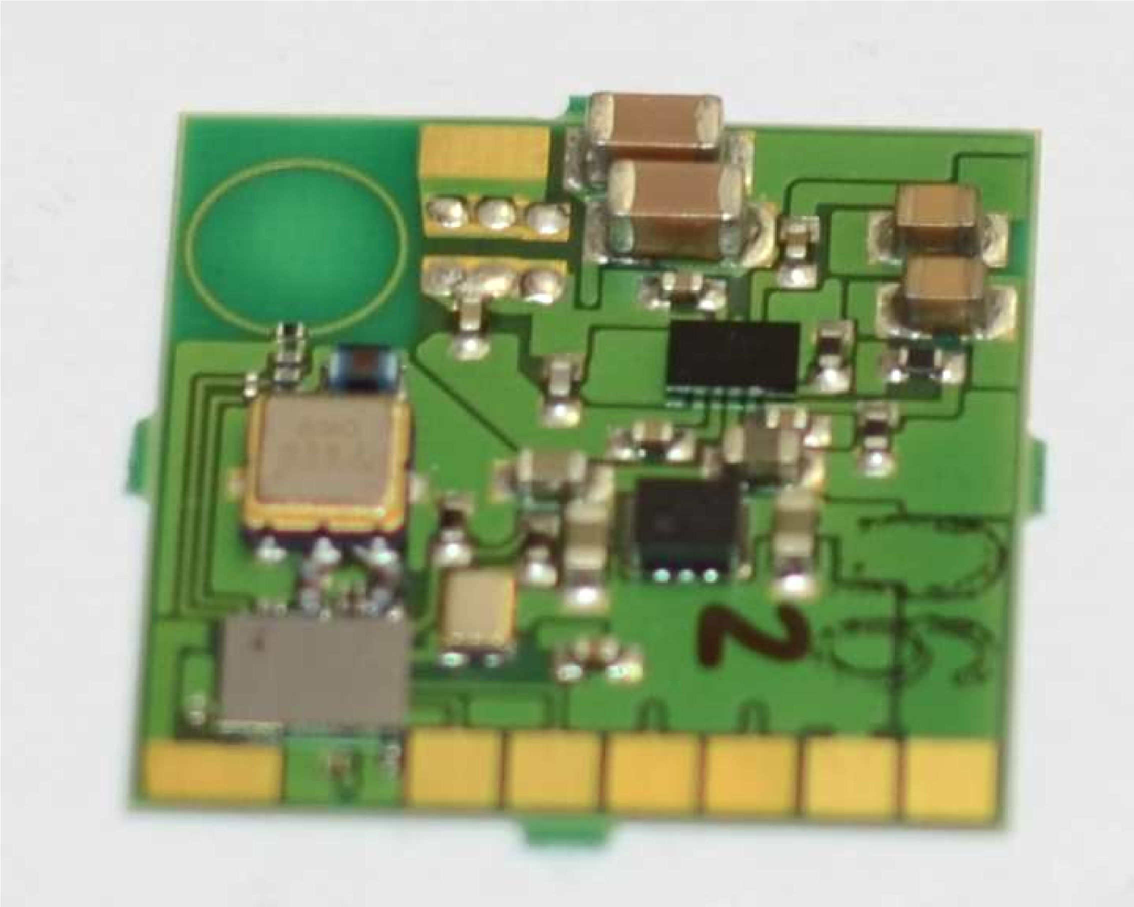

Figure 4.3: Close-up of the energy-data-buoy without connected energy-coil. The lower contacts provide the four SPI connections and a 24MHz clock-line as well as ground and 3.3V supply voltage for the rest of the implant.

In the following, I will discuss the energy harvesting and wireless RF data transfer components in more detail.

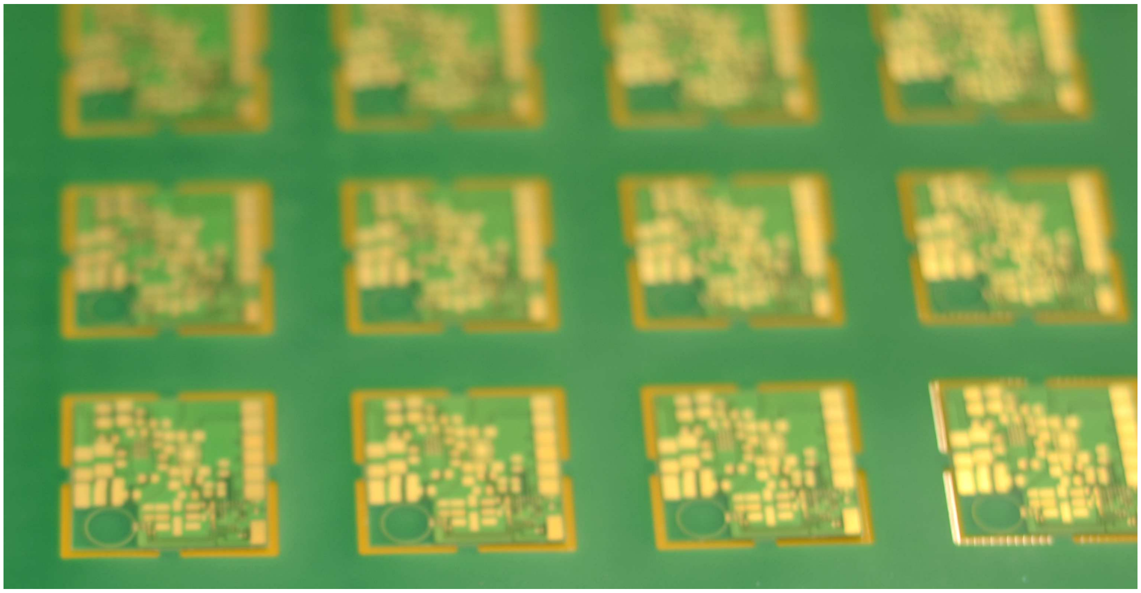

Figure 4.4: PCBs for the Energy-data-buoy on thin but un-flexible FR4 substrate. The PCBs were produced by Andus (Berlin).

4.1.1 Energy

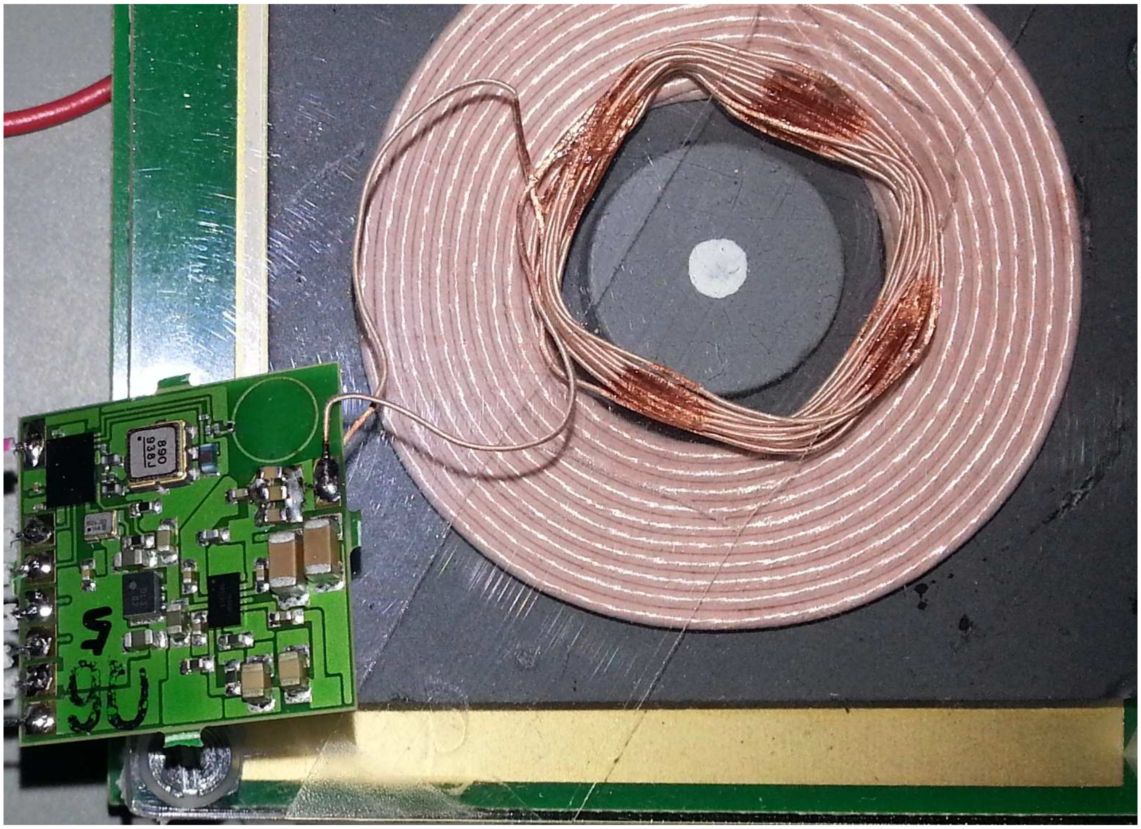

Figure 4.5: Energy-data-buoy and its power-coil on top of the Texas Instruments power transmitter. Photo was made by the IMSAS (Thomas Hertzberg and Elena Tolstosheeva)

During the project Darren Gould and Dmitriy Boll worked on several advanced approaches of delivering wirelessly and robustly energy to the implant. However, it was important to get a temporary solution for the energy transfer for the first runs of the implant test system fast. Such an energy system needs to fulfil several requirements: 1.) It needs to transmit energy in order of magnitude of 100mWatt. 2.) It shouldn't heat up the surrounding tissue, which means that it needs a dynamic link between the transmitter and receiver for adapting the power inflow. 3.) It needs to deliver a stabilized output voltage. 4.) The frequency of the link needs to be far away from the 400MHz of the RF data link. 5.) The IC needs to be available in a chip size package or bare die.

In the Texas Instrument Tesla system I found all these requirements meet. This system was designed according to the QI standard for wirelessly charging mobile phones and MP3 player with up to 5Watt. Both, the transmitter and receivers, are available on evaluation kits for around 100Euros each. This even makes an expensive energy transmitter system unnecessary and first tests are done very easily. The bq51013

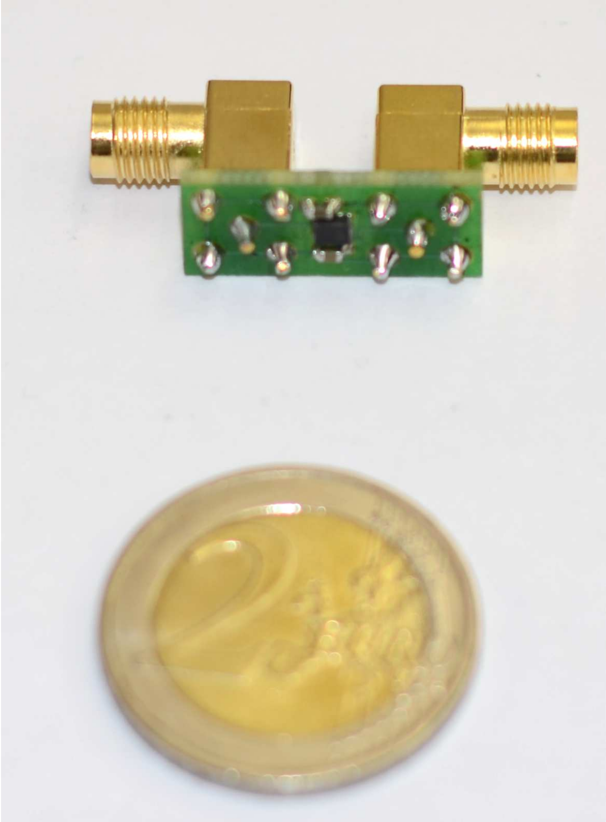

Figure 4.6: For the energy-data-buoy, the very small and efficient DC/DC converter (Torex XCL205) with its two capacitors on its test PCB.

energy receiver IC collects its energy from frequencies between 110kHz - 220kHz. The receiver and transmitter communicate over that field and the transmitter adapts its output frequency to the actual power needs of the receiver. If the receiver needs less power, the transmitter increases the frequency and thus moves it further way from the receiver's resonate frequency with around 100kHz. This allows a controlled energy flow which keeps not required power away from the implant. Also the gap between 220kHz and the 400MHz of the RF data link is conveniently large. The size of the IC in the chip size package is roughly 3mm x 1.9mm x 0.6mm.

We mainly replicated the circuit shown in the manual for the TI bq51013's manual (see there figure 2 'bq5101x Used as a Wireless Power Receiver and Power Supply for System Loads'). We used the values for the components listed in the TI bq51013's evaluation kit manual (bq51013 EVM-725 evaluation module; figure 1. HPA725 EVM Schematic). We changed only the following: 1.) We set the resistor between the Ilim pad and ground to 1.5kΩ for reducing the current limit to 200mA. 2.) On its output we have only two 10µF (10V) capacitors for buffering and then the 5.0V DC output goes directly to the DC/DC converter. 3.) We removed the temperature sensing component by a simple 10kΩ resistors to ground, since the required components would

have been spatially to high and the sensor wouldn't have picked up the temperature at the relevant position anyway. 4.) The pads EN1, EN2 and AD are connected to ground. The pads AD-EN and CHG are left open. 5.) Tim Schellenberg wound a custom quadratic coil for the energy harvesting circuit with the size of 2cm x 2cm with 10 windings (see figure 4.5).

It is important to note that many of the supporting components (mostly capacitors) around the coil and rectification need to withstand voltages in the region of 25V. Thus it is recommended to use 50V rated capacitors for these components. However, capacitors with larger values from this voltage class are build relatively high. This is a problem for implantation because they are higher than the space between brain and scull. This is the reason why in the design for the whole implant, large capacitors are split into many capacitors with smaller values.

A mayor problem with manufacturing a PCB for the bq51013 is that it is necessary to put VIAs inside the pads (it is not possible to route the connections around the obstacles). Normal VIAs are not suitable because they are open constructs (like small tubes). The problem is as follows: On the flip-side of the power IC it's contact pads are located. For making assembly easier, the manufacturer placed already little balls of solder on these pads. Normally during assembly, the PCB and IC is heated, then the balls melt and contact the corresponding pads on the PCB. In the case of an open VIA, the solder from the ball is sucked into that capillary and no solder is left for the contact. Thus it is required to close the VIAs very evenly with metal for a good electrical contact like it is the case for a normal pad and isolate it reliably from other pads with solder resist (otherwise the solder could flow away to other pads). Generating such a high quality filling is complex. The boards needs to be thin and all the VIAs need to have the same diameter.

On the transmitter side we tested the evaluation boards for the old Texas Instruments wireless power transmitter manager bq500110 EVM-688 and the newer bq500210 EVM-689. The ITEM's experiments showed that the newer version created spikes in the recordings which the old version doesn't produces. Thus we decided to use the bq500110 EVM-688 board as power transmitter for all the tests. However, we still noticed a problem even with that power transmitter. In the case when the distance between the implant's coil and the power transmitter coil gets too large (more than several mm), then the power transfer stops. We expect that this is a result of the break-down of the communication between both systems. This communication is necessary for the dynamic frequency adaptation that limits the energy collected by the receiver. Without the information about the actual implant's power requirements, the transmitter shuts itself off for safety reasons. For increasing the maximal distance, it should be possible to improve the coil of the transmitter. An improvement on the implant side is more problematic because of the limited space.

The TI bq51013 delivers a more or less stabilized 5.0V DC output. All the components of the implant need 3.3V supply voltages. Thus we were in the need of a DC/DC converter which was extremely small, needs as few as possible external components,

and is as efficient as possible. For example a normal low-drop out regulator would convert the excess energy into heat. This would be very destructive for the brain tissue. Hence, a DC/DC converter based on switching or injection technology is required. I found in the Torex XCL205 a suitable converter. It is able to convert 5.0V into 3.3V with nearly 90% efficiency, is small (2.5mm x 2.0mm x 1.04mm) and requires only two external capacitors. The XCL205 works at 3MHz and is able to convert up to 600mA, which is much more than required.

For testing the DC/DC converters in advance, I designed a simple test module that can be screwed between a power source and load. PCB-Pool manufactured for me the PCBs and Dmitriy Boll assembled them for me (see figure 4.6).

The final energy-data-buoy was able to deliver several times the energy which was required by the implant. However, the 3.3V power from the energy-data-buoy was polluted by a 24 MHz clock which is also on the buoy. We solved this problem with an additional PI filter, which we then included in the implant design. With the PI filter, the implant test system was successfully powered and measurements were performed completely wirelessly.

4.1.2 Data

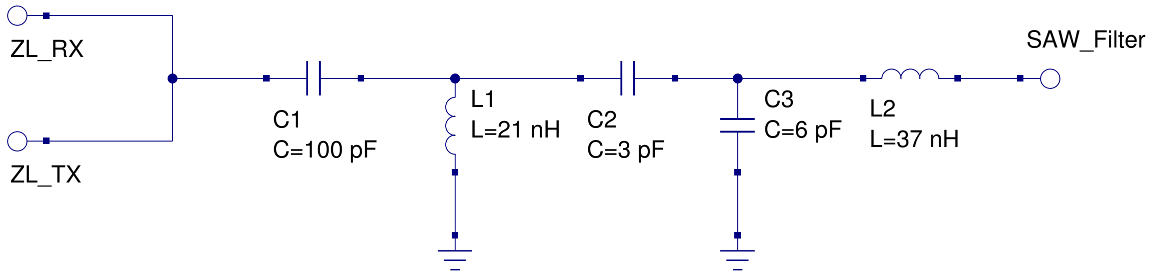

Figure 4.7: The 37nH inductor L2 and the 6pF capacitor C3 match the SAW filter input onto a 50Ω port. The rest of the matching network was taken from Zarlink AIM100 (application implant mezzaine) test module.

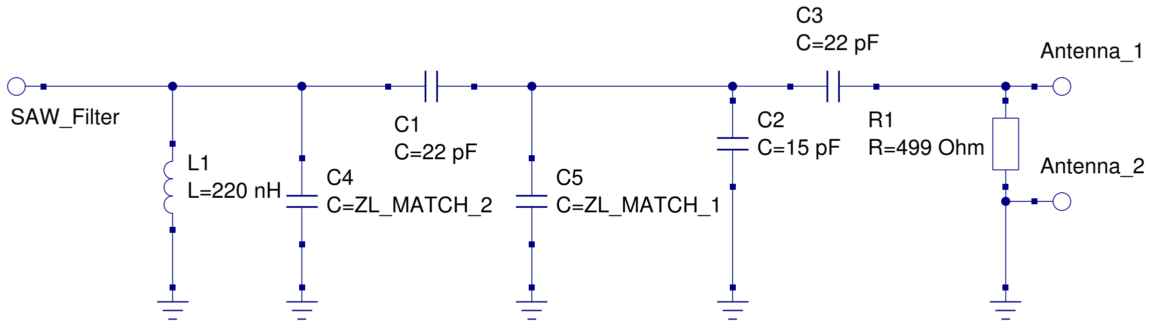

Figure 4.8: The saw filter output and the 220nH inductor create together a 50Ω port and we assume that the antenna has 12.3nH. The antenna matching network was developed by Tim Schellenberg.

Beside the energy harvesting and power management, the energy-data-buoy contains also the necessary equipment for exchanging data with an external base station wirelessly. This radio-frequency link at the MICS band (Medical Implant Communication Service band at around 403 MHz) is created by using Zarlink ZL70102 transceivers on the implant and on the base station.

On the implant we used the chip size package version, which is small and allows an easy assembly process. As external components the ZL70102 needs on the implant side only a few components: 1.) Two 68nF capacitors, where one is placed between VDDD and ground and the other one between VDDA and ground. 2.) 0.1µF capacitor for stabilizing the supply voltage. 3.) A stable 24MHz clock like the Nihon Dempa Kogyo (NDK) NZ2016SA. This 3.3V CMOS clock was discovered by Janpeter Höffmann and is extremely small (2.0mm x 1.6mm x 0.7mm). Since the Zarlink IC needs lower clock signal levels we had to introduce an extra voltage divider (8.2kΩ on the signal and 12kΩ on the ground side). A useful side-effect is that this clock module can also provide a stable clock signal for other components on the implant, which are not directly located

on the buoy. 4.) The antenna matching network with a SAW filter and the antenna itself.

Using the Zarlink ZL70102 within the implant's design is relatively simple and the circuit diagram can nearly completely be taken from the ZL70102 manual. Most of the pins have to be grounded. The pins CLF_REF, PO0, PO1, PO2, PO3, CLF1, IRQ, and XTAL2 stay unconnected. XO_Bypadd, VSUP, WU_EN, VDDIO need to be connected to the 3.3V supply voltage. XTAL1 is connected though the voltage divider to the 24MHz clock. For activating the so-called housekeeping mode on power up, Mode1 pin needs to be connected to the supply voltage and Mode0 pin to ground. This housekeeping mode allows to modify essential settings in the implant's Zarlink IC via the RF link from the base station. The SPI pins (SPI clock, data in, data out and chip select) as well as the 3.3V version of the 24 MHz clock, ground and the 3.3V power rail are routed to pads at the rim of the buoy, allowing to connect all of them to the rest of the implant. And finally the pads RF_RX and RF_TX are connected to the antenna matching network as well as Match1 and Match2.

The most complex part of the RF transceiver installation is the antenna matching network with its filter and the custom made antenna. This work was done by Tim Schellenberg. Tim developed a circular loop antenna (diameter 5mm with a conductor width of 0.2mm), researched the SAW filter and calculated the antenna matching network. One problem was that the SAW filter, which was originally used by Zarlink in their reference design, wasn't available any more. After some research we decided to use the RFM RF3607D (403.5 MHz SAW Filter) which is not as small as the original one but was still acceptable with its 3.8mm x 3.8mm x 1mm. We used the example circuit diagram from the RF3607D's manual and converted both sides into 50Ω ports. The rest of the antenna matching network, between the Zarlink and the SAW filter, was taken from Zarlink AIM100 (application implant mezzaine) test module's reference design (see figure 4.7). On the other side of the filter, the antenna had to be matched to the 50Ω port of the SAW filter. Tim Schellenberg determined the inductance of the antenna with 12nH. Figure 4.9 (which was generated with the open source smith chart tool linsmith http : //sourceforge.net/projects/linsmith/) shows how the matching network in between works when all component values are meet perfectly. However, the reality is typically different. For than reason the Zarlink IC provides two programmable capacitors which can be accessed by the pins Match1 (2.5pF - 15.5pF) and Match2 (2pF - 16pF) while their second side is connected within the IC to ground. The Zarlink IC takes care of automatically finding the optimal setting itself. Together we modified the second part of the antenna matching network such that a large variety of impedances can be matched (see figure 4.8 for the network and figure 4.10 for the corresponding smith charts).

After production, Dmitriy Boll added, besides the missing energy coil and its matching capacitor, a ribbon cable to the pads of the buoy. This allowed us to use the Zarlink transceiver via SPI with ITEM's implant prototype (see figure 4.1). The tests showed that the RF data link also worked fine.

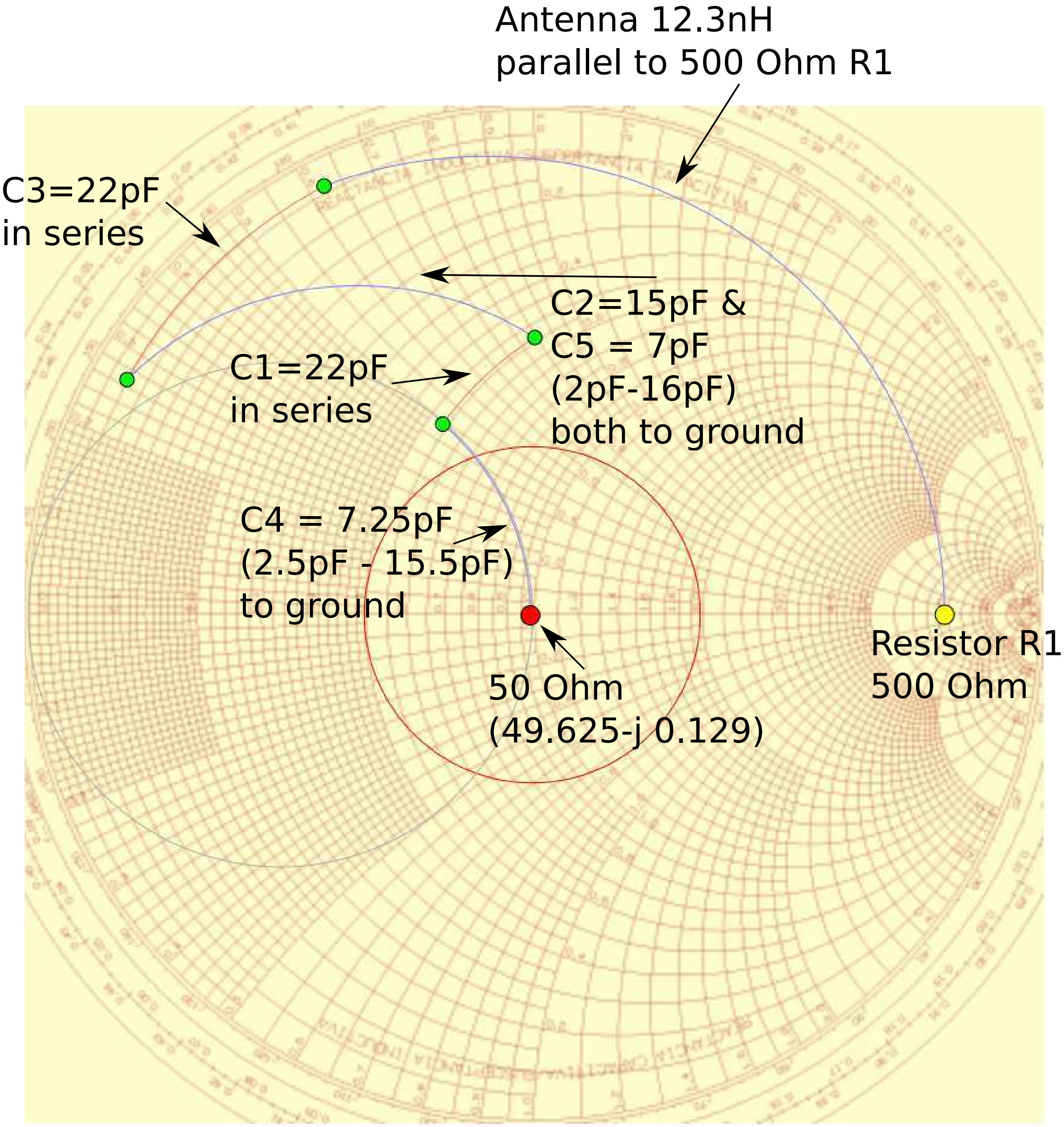

Figure 4.9: Smith chart analysis of the antenna matching network shown in figure 4.8. Figure created with linsmith and modified with Inkscape.

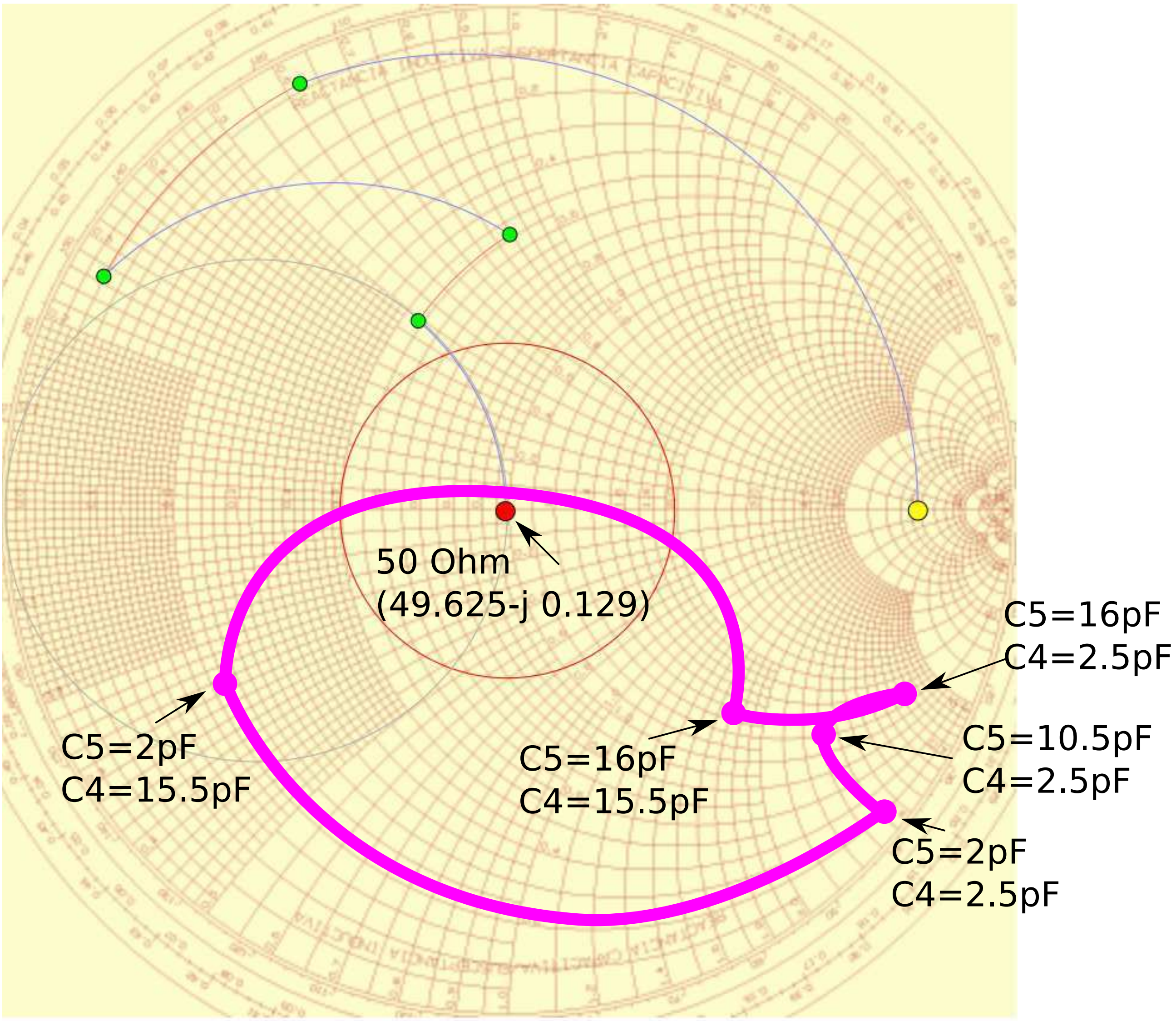

Figure 4.10: Further analysis of the antenna matching network shown in figure 4.8. In comparison to figure 4.9, the programmable capacitors of the Zarlink IC were varied. The pink region shows the matching which is possible with the available capacitor setting.

4.2 The implant's design

The ITEM's ASIC and the RHAs (bio-signal amplifier with ADC from Intan Technologies www.intantech.com) were successfully tested together on the implant test system. After the success with the energy-data-buoy, we wanted to take the next step. The goal was a version of the implant with all features and based on a flexible substrate.

As a first step, I copied the design of the energy-data-buoy and added several Pi filters into the power rails. The test with the original energy-data-buoy showed a pollution of the power rail by the 24MHz clock. We wanted to get rid of these perturbations. Then I added the ITEM ASIC and eight 16 channel RHAs (RHA2116) plus 128 electrodes and one large reference electrode to the design. The functional structure of the whole implant is shown in figure 4.11.

The data acquisition system of the implant is based on the RHA2116, which was discovered by Janpeter Höffmann. This IC is a combination of a 16 channel bio-signal amplifier (optimized for electro-physiological applications), an analogue multiplexer and an (undocumented) analogue-to-digital converter. The ADC uses the SPI protocol for exchanging data with other devices. The aim was to have 128 electrodes on the implant. As result we had to operate 8 of these RHA IC in parallel. The circuit diagram for the supporting components was taken from the RHA's manual.

A custom ASIC was required to collect the acquired samples from all these RHAs, preprocess the data and operate the RF transceiver for sending them to the external base station. This IC was developed by the ITEM (mainly by Jonas Pistor and Janpeter Höffmann). The ASIC contains parts that operate the RHAs via SPI. It also has the means to remove a user defined set of recording channels from the data stream as well as the possibility to reduce the sample rate and number of bits per sample. This all is done by control sequences received via the RF link. The ASIC has the ability to control the power supply of the RHAs, allowing to save energy if not all RHAs are required at a given moment. But the major part of the ASIC is concerned with controlling the Zarlink ZL70102 (opening connections, exchanging data, and analysing incoming control sequences), caching the data and generating data packages which can be understood by the base station.

For reducing the height of the implant, I had to split many of the larger 50V-rated capacitors of the energy harvesting system in many 50V-rated smaller valued capacitors. Especially the 10µF ones were to high. The RHAs band-pass filter were set to 0.15Hz at the lower cut-off frequency (L=1.9MΩ) and to 200Hz at the upper cut-off frequency (H1=403kΩ and H2=634kΩ).

The final goal is to build the implant based on a highly flexible substrate. Elena Tolstosheeva did intensive research on building such foils with electrodes for optimally interfacing brain tissue (supported by animal tests done by Victor Gordillo Gonzalez). However, we decided to use an industrially produced foil as an intermediate step,

hoping to allow us to produce first test systems in a short time. Together with the company Andus we planned the production of a flexible two layer PCB (34mm x 81,5mm x0,05mm) based on DuPont Pyralux AP foil with nickel & gold surface. Again, exactly like with the energy-data-buoy, the VIAs (now uniformly set to 0.1mm) for the BGAs had to be evenly filles & closed and the pads had to be carefully protected by solder resist. However, manufacturing these foils was much more difficult than expected and took much more time than planned. Figure 4.12 and 4.13 show this foil.

Since space is very limited on the implant, it was important to use chip sized packages were ever possible and bare dies where necessary. First we planned to use so-called flip-chip bounding for assembling the bare dies. This method is used to connect the bare die's contacts directly to the PCB's pads by conductive balls as mediator. However, the standard flip-chip bonding processes have requirements for the pad surfaces of the die as well as the PCB. The RHA and the ASIC doesn't meet these requirements. The IMSAS is working on an adapted version but hasn't reached the necessary yield yet. Thus we started to plan the assembly of the implant in cooperation with the company Micro-Hybrid (Berlin, $www.micro-hybrid.de$). We decided to use so-called deep access wire bonding instead. In this special wire bonding process, where the golden bond-wires are installed in a region very close the rim of the die. It doesn't need much more space than flip-chip bonding. On the negative side, it forced me to mirror the corresponding parts of the design.

The implant is still in production. In the end we hope to get a fully working implant on a fold-able substrate, allowing to fold the implant at three positions such that the 128 electrodes are on top and the reference electrode is on the bottom (looking at the scull). In between all the electronic components are enclosed, while the antenna is not covered by any obstacles. When the implant is successfully testes on the bench, then we hope to find an easy way to protect it for short term tests in liquid environments.

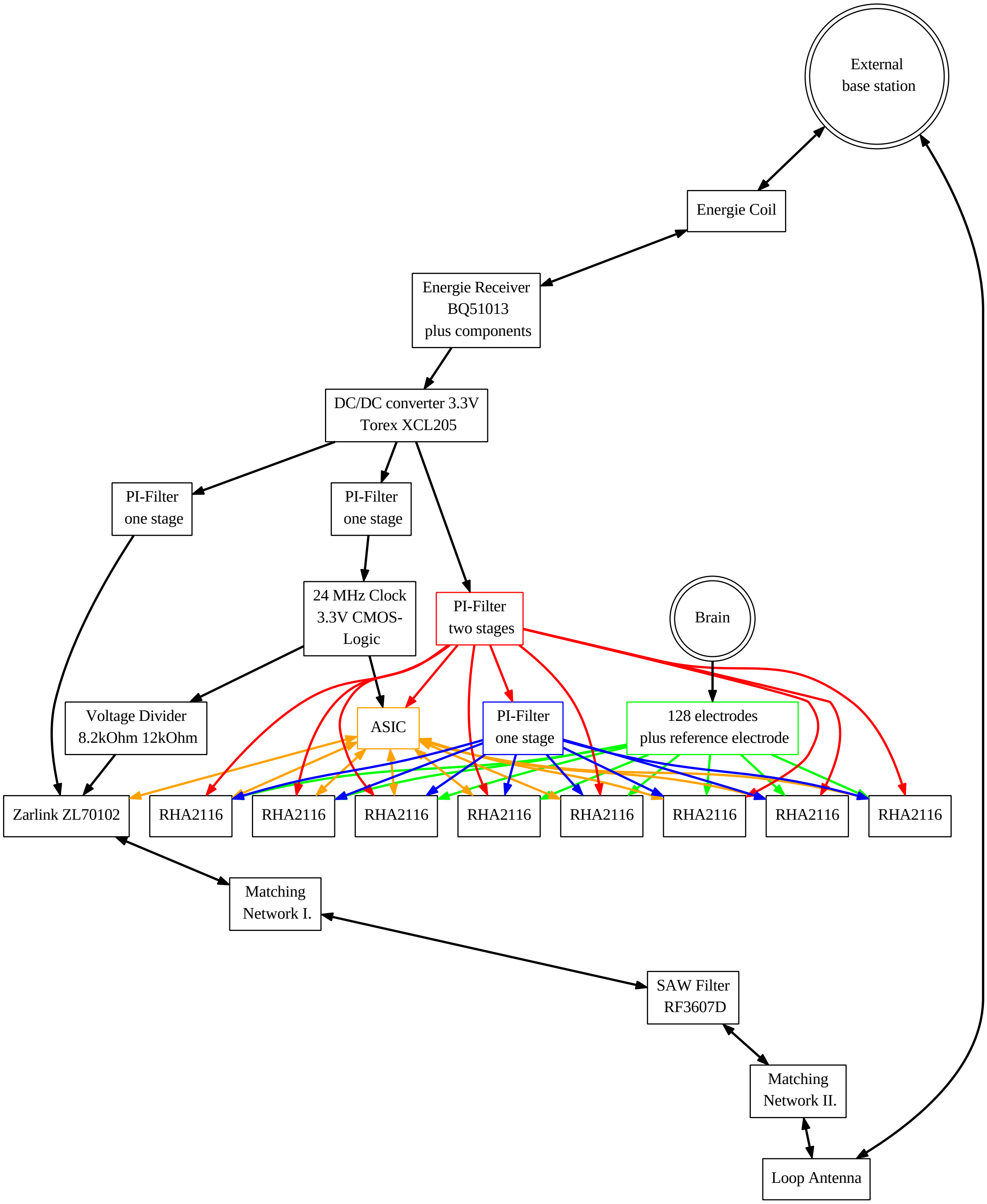

Figure 4.11: Structural overview of the implant. The red arrows represent the digital power rail and the blue arrows the analogue supply voltage to the RHA ICs.

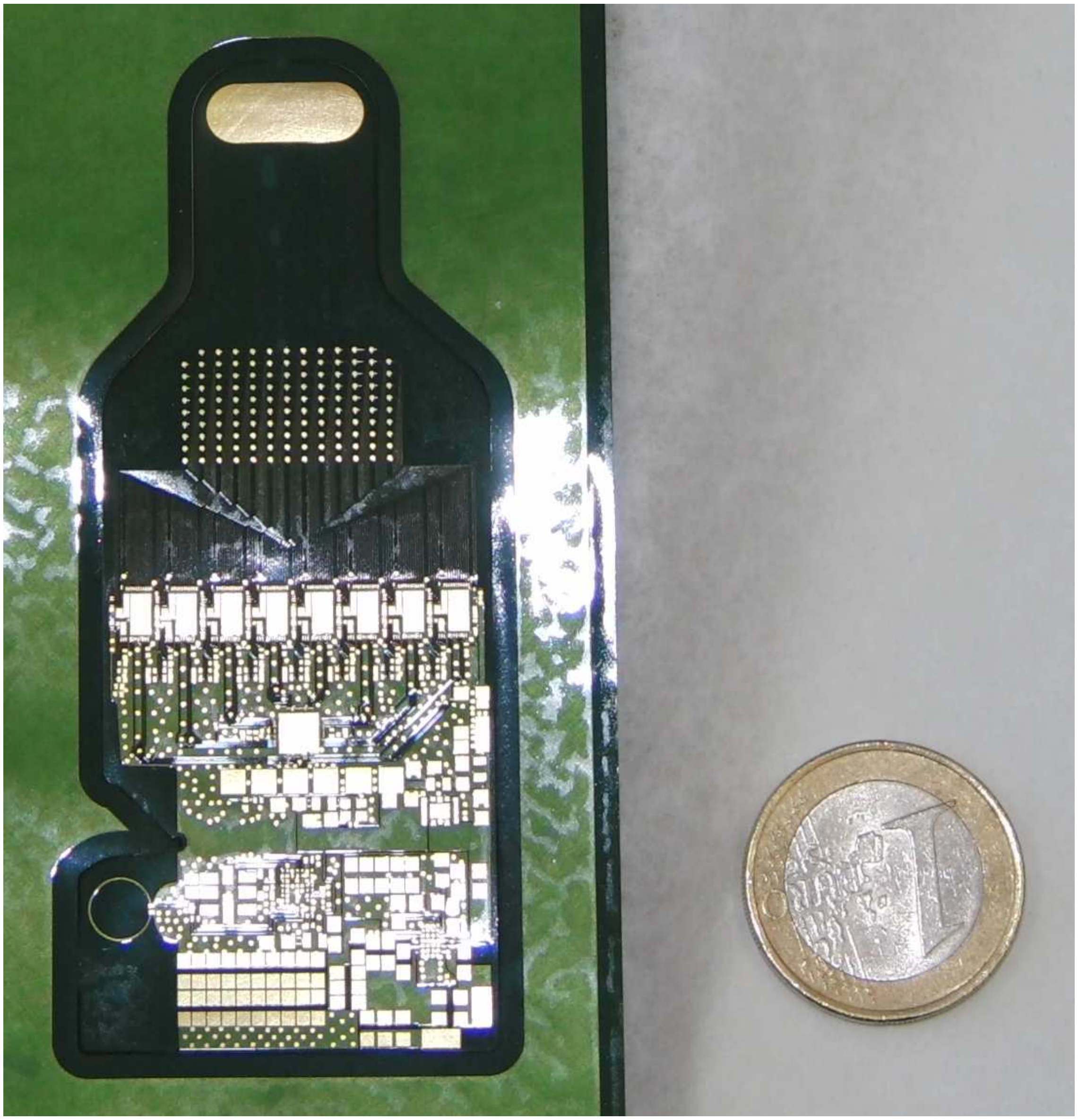

Figure 4.12: Thin flexible foil as PCB for the implant. This PCB was produced by Andus (Berlin).

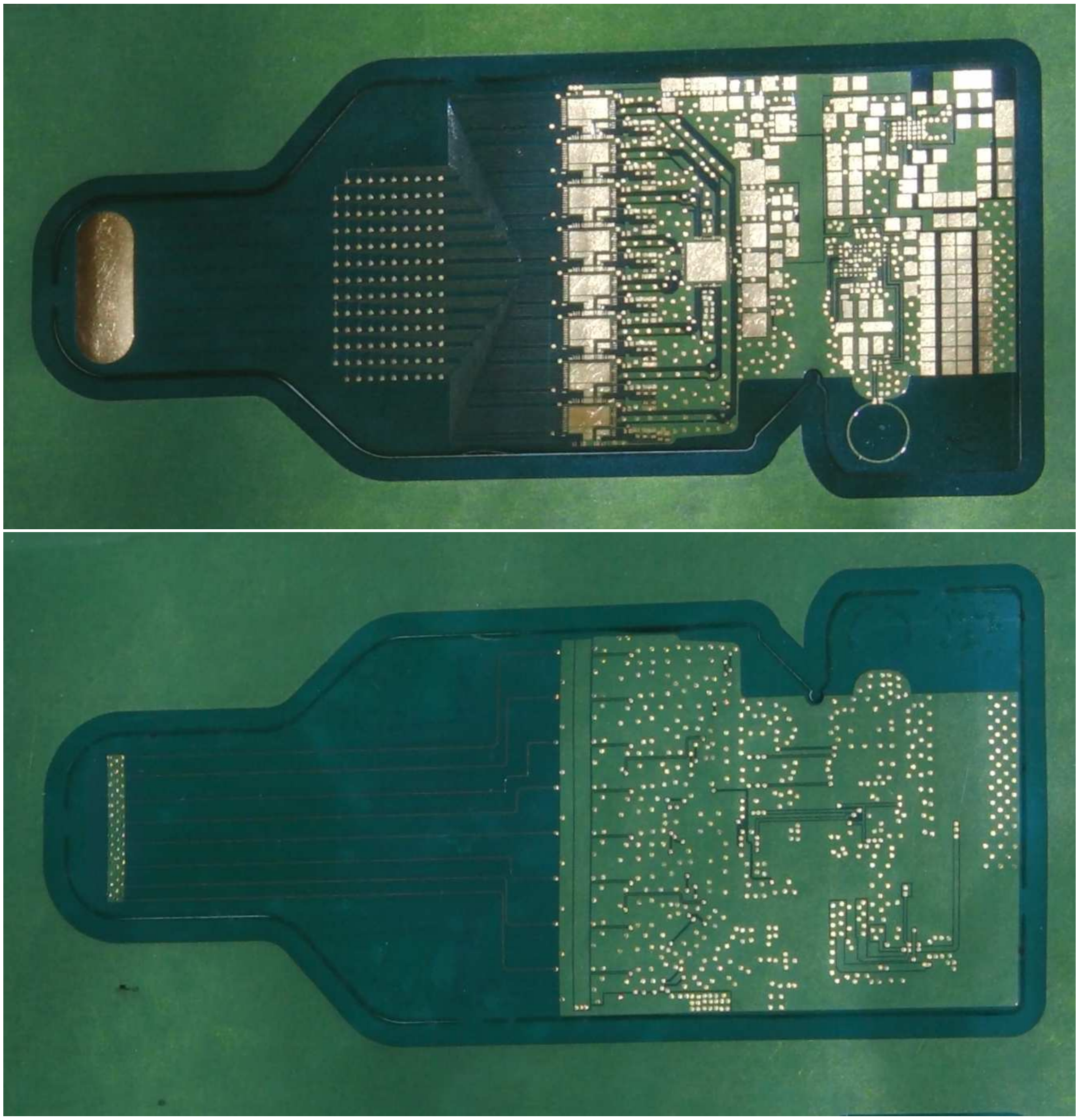

Figure 4.13: Front- and flip-side of the implant's foil.

4.2.1 Pi - filter

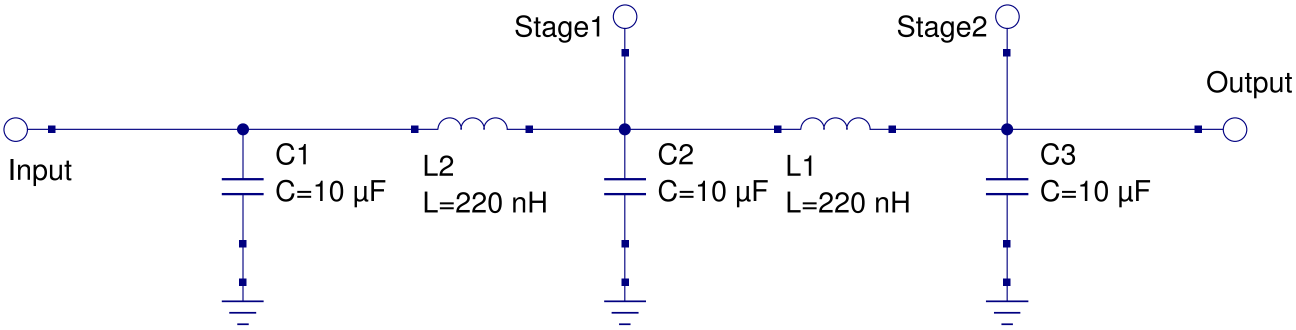

Figure 4.14: Two stages of Pi-filters for removing non DC components from the DC power rail.

During tests with the energy-data-buoy, Jonas Pistor and Janpeter Höffmann found the 3.3V DC power rail be polluted by a high frequency distortions. We expect that this is a residue of the 24MHz clock. Such pollutions are not desirable, especially since this supply voltage is used for powering amplifiers and analogue-to-digital converters. As counter measure, I introduced several one and two stage Pi filters into the design of the implant. The Pi filter, also called capacitor-input filter, (see $http://en.wikipedia.org/wiki/Capacitor-input\_filter$ for details) is a standard counter-measure for this type of problem. One stage of Pi-filter consists out of two capacitors and one inductance. The goal is to use as large as possible capacitors and inductances for the filter. In case of the implant, the maximal values (at a ≈6V-rating) are limited by the available space.

With the largest suitable capacitors and inductances, that I found (see figure 4.14), I simulated the impact of the Pi-filter. The results are shown in the figure 4.15.

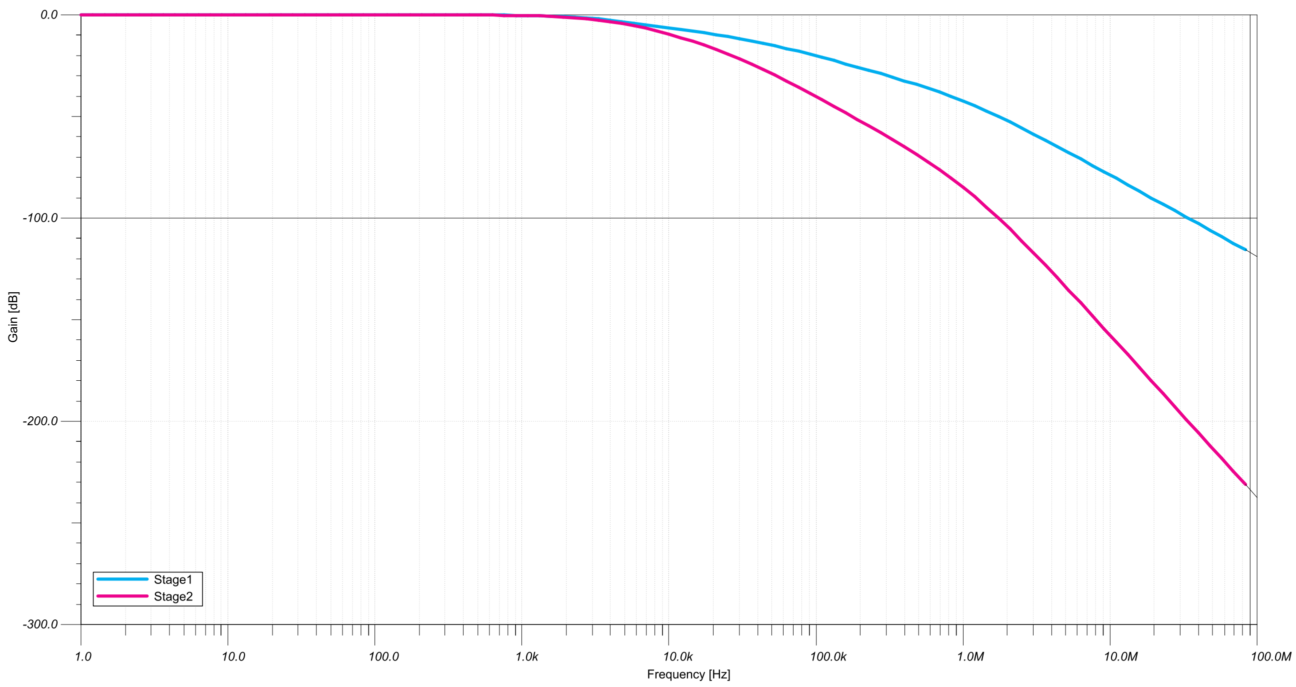

Figure 4.15: Effect of the two PI-filter stages on the DC power rail's unwanted higher frequency components. The filter was simulated with TI Tina 9 and a 1kΩ load.
