# Supplementary material for: Implications for a Wireless, External Device System to Study Electrocorticography
Source: Sensors (Basel). 2017 Apr 4;17(4):761. doi: 10.3390/s17040761 (PMC5421721; doi:10.3390/s17040761)
Supplement: Supplementary file 1 [file sensors-17-00761-s001.zip › Supplement/PCBs/SATA_SMA_RevB.zip!/SATA_SMA_RevB/SATA_SMA_Back.pdf]

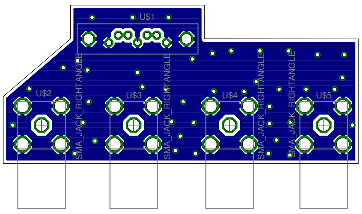
U$1
U$2
SMA_JACK_RIGHTANGLE
U$3
SMA_JACK_RIGHTANGLE
U$4
SMA_JACK_RIGHTANGLE
SMA_JACK_RIGHTANGLE
U$5
