# Supplementary material for: Implications for a Wireless, External Device System to Study Electrocorticography
Source: Sensors (Basel). 2017 Apr 4;17(4):761. doi: 10.3390/s17040761 (PMC5421721; doi:10.3390/s17040761)
Supplement: Supplementary file 1 [file sensors-17-00761-s001.zip › Supplement/PCBs/SATA_SMA_RevB.zip!/SATA_SMA_RevB/SATA_SMA_Front.pdf]

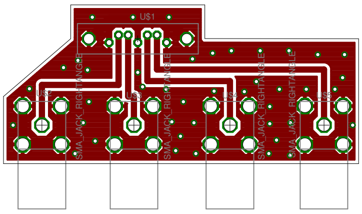

22.08.11 16:07 /home/davrot/eagle/Basestation/SATA_SMA_RevB/SATA_SMA.brd
