# Supplementary material for: Implications for a Wireless, External Device System to Study Electrocorticography
Source: Sensors (Basel). 2017 Apr 4;17(4):761. doi: 10.3390/s17040761 (PMC5421721; doi:10.3390/s17040761)
Supplement: Supplementary file 1 [file sensors-17-00761-s001.zip › Supplement/PCBs/Digital_Out_RevB.zip!/Digital_Out_RevB/Digital_OUT_Front.pdf]

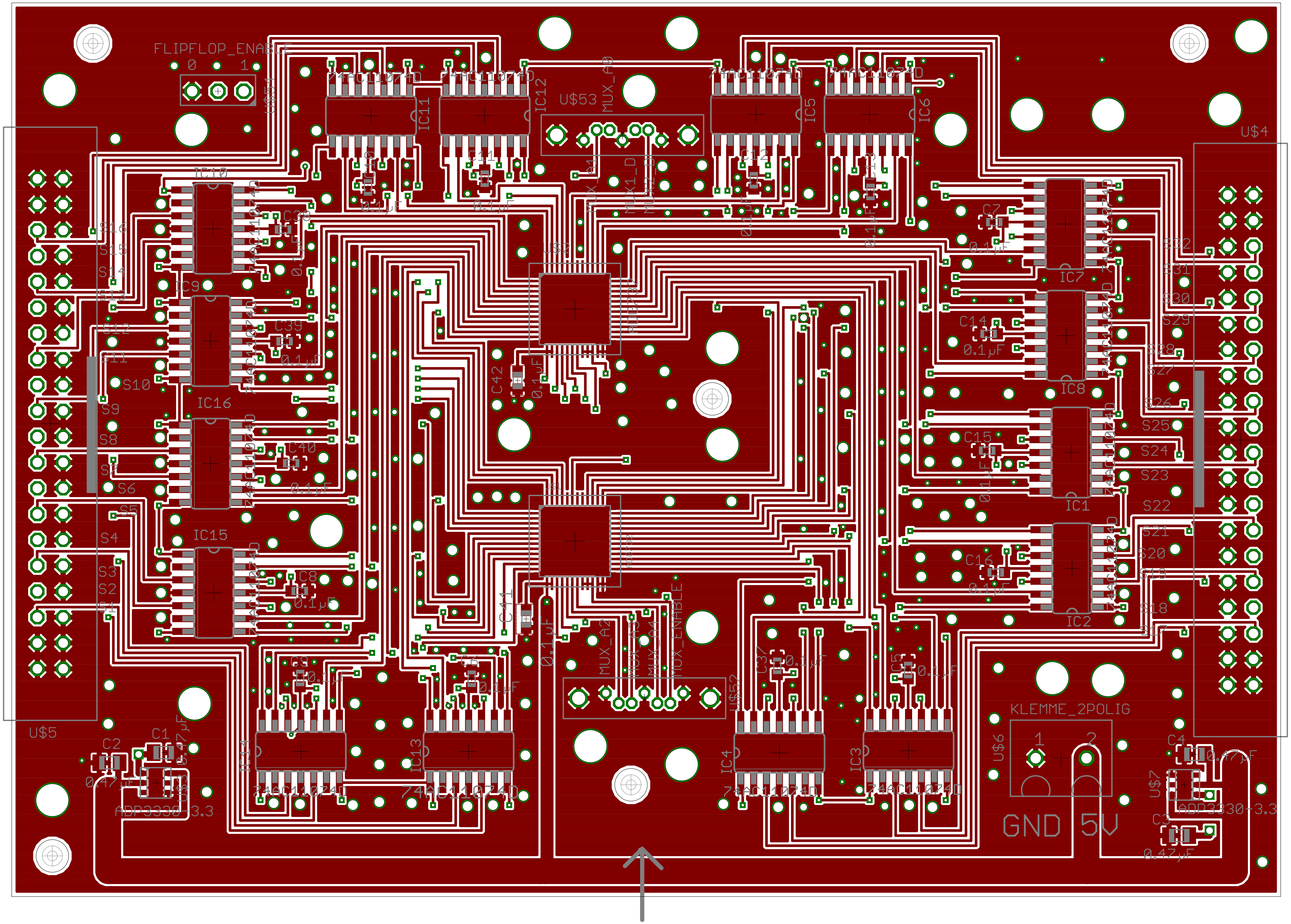

Cut Backside 3.3V Wire here

Otherwise problems will emerge
