# Supplementary material for: Implications for a Wireless, External Device System to Study Electrocorticography
Source: Sensors (Basel). 2017 Apr 4;17(4):761. doi: 10.3390/s17040761 (PMC5421721; doi:10.3390/s17040761)
Supplement: Supplementary file 1 [file sensors-17-00761-s001.zip › Supplement/PCBs/Zarlink_Basisstation.zip!/Zarlink_Basisstation/Zarlink_RevC_Back.pdf]

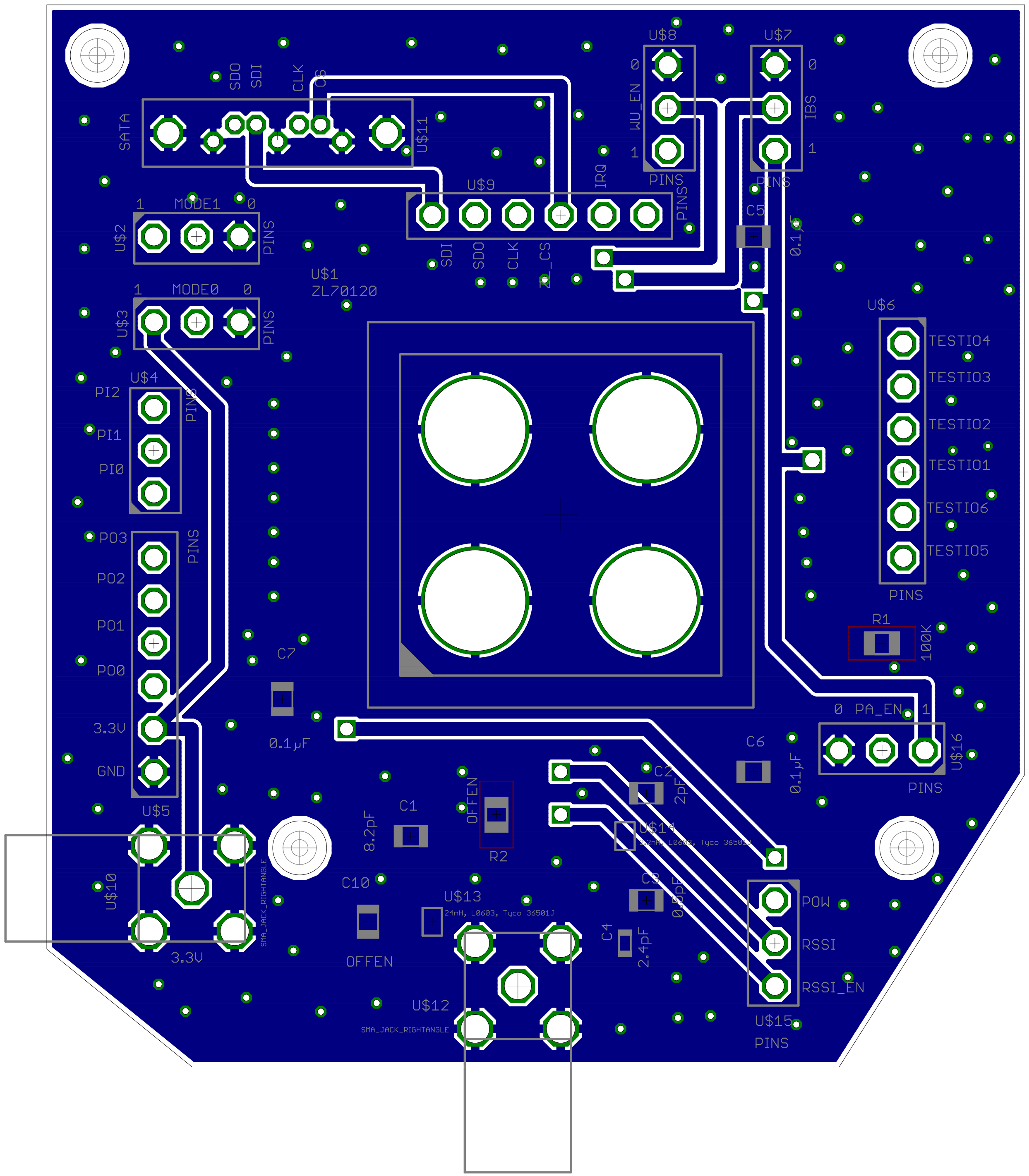

13.08.11 00:08 f=2.70 /home/admin/eagle/ThirdEdition/Zarlink/Zarlink_RevC.brd
