# Supplementary material for: Implications for a Wireless, External Device System to Study Electrocorticography
Source: Sensors (Basel). 2017 Apr 4;17(4):761. doi: 10.3390/s17040761 (PMC5421721; doi:10.3390/s17040761)
Supplement: Supplementary file 1 [file sensors-17-00761-s001.zip › Supplement/PCBs/RfEnergyModule.zip!/RfEnergyModule/Power_Final_Back.pdf]

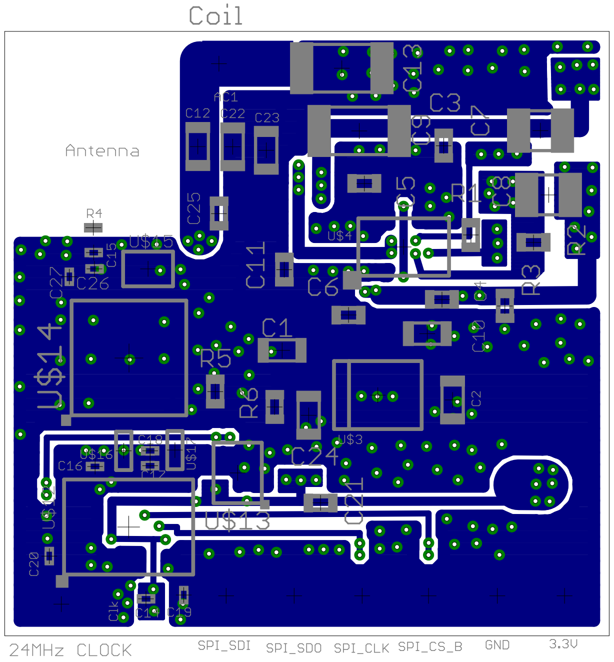
Coil
Antenna
24MHz CLOCK
SPI_SDI
SPI_SDO
SPI_CLK
SPI_CS_B
GND
3.3V
