# Supplementary material for: Implications for a Wireless, External Device System to Study Electrocorticography
Source: Sensors (Basel). 2017 Apr 4;17(4):761. doi: 10.3390/s17040761 (PMC5421721; doi:10.3390/s17040761)
Supplement: Supplementary file 1 [file sensors-17-00761-s001.zip › Supplement/PCBs/RfEnergyModule.zip!/RfEnergyModule/Power_Final_Ciruit.pdf]

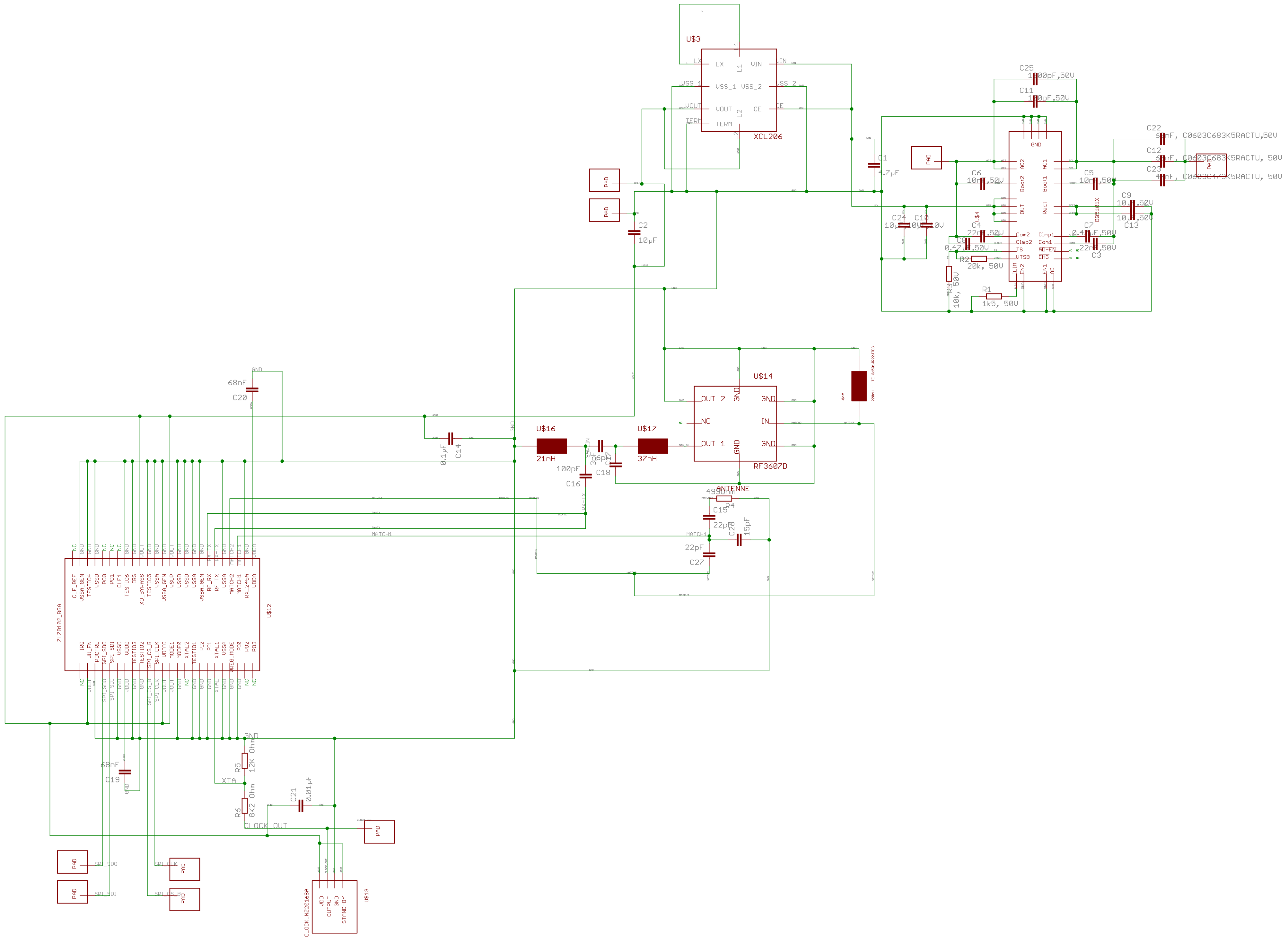

U$3
XCL206
LX
VIN
VSS_1
VSS_2
VOUT
CE
TERM
L1
L2
PAD
C2
10µF
C1
4.7µF
C25
1000pF,50V
C11
100pF,50V
C22
68nF, C0603C683K5RACTU,50V
C12
68nF, C0603C683K5RACTU, 50V
C23
47nF, C0603C473K5RACTU, 50V
C6
10nF,50V
C5
10nF,50V
C9
10µF,50V
C13
C24
C10
C4
22nF,50V
C7
0.47µF,50V
C8
0.47µF,50V
C3
22nF,50V
R2
20k, 50V
R3
10k, 50V
R1
1k5, 50V
U$4
BQ5101X
GND
AC2
AC1
Boot2
Boot1
OUT
Rect
Com2
Com1
Clmp2
Clmp1
TS
AD-EN
VTSB
CHG
ILIM
FN2
FN1
AD
U$14
RF3607D
OUT 2
OUT 1
NC
IN
GND
U$16
21nH
U$17
37nH
C16
100pF
C17
36pF
C18
56pF
C14
0.1µF
C20
68nF
ANTENNE
R4
499Ohm
C15
22pF
C26
15pF
C27
22pF
MATCH1
U$12
ZL70102_BGA
CLF_REF
VSSA_GEN
TEST04
VSSD
PO0
PO1
CLF1
TEST06
IBS
XO_BYPASS
TEST05
VSSA
VSUP
VSSA_GEN
RF_RX
RF_TX
MATCH2
RX_245A
VDDA
IRQ
WU_EN
PDCTRL
SPI_SDO
SPI_SDI
VDDD
TEST03
TEST02
SPI_CS_B
SPI_CLK
VDDIO
MODE1
MODE0
XTAL2
TEST01
PI2
PI1
XTAL1
VREG_MODE
PI0
PO2
PO3
NC
C19
68nF
R5
12K Ohm
R6
8K2 Ohm
XTAL
C21
0.01µF
CLOCK_OUT
SPI_SDO
SPI_SDI
SPI_CLK
SPI_CS_B
U$13
CLOCK_NZ2016SA
VDD
OUTPUT
STAND-BY
U$15
