# Supplementary material for: Implications for a Wireless, External Device System to Study Electrocorticography
Source: Sensors (Basel). 2017 Apr 4;17(4):761. doi: 10.3390/s17040761 (PMC5421721; doi:10.3390/s17040761)
Supplement: Supplementary file 1 [file sensors-17-00761-s001.zip › Supplement/PCBs/RfEnergyModule.zip!/RfEnergyModule/Power_Final_Front.pdf]

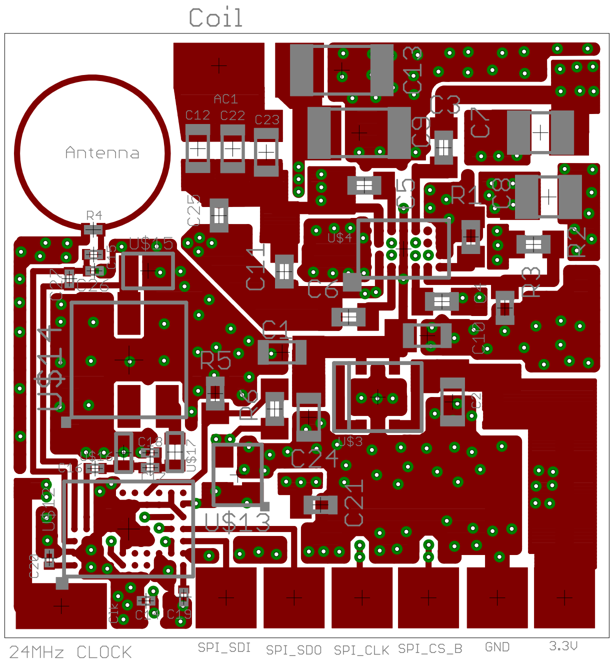
Coil
Antenna
AC1
C12
C22
C23
C13
C3
C7
R1
R2
R3
R5
C11
C6
C1
C24
C21
U$14
U$13
24MHz CLOCK
SPI_SDI
SPI_SDO
SPI_CLK
SPI_CS_B
GND
3.3V
