# Supplementary material for: Implications for a Wireless, External Device System to Study Electrocorticography
Source: Sensors (Basel). 2017 Apr 4;17(4):761. doi: 10.3390/s17040761 (PMC5421721; doi:10.3390/s17040761)
Supplement: Supplementary file 1 [file sensors-17-00761-s001.zip › Supplement/PCBs/BaseBoard_RevBLarge.zip!/BaseBoard_RevBLarge/BaseBoard_Front.pdf]

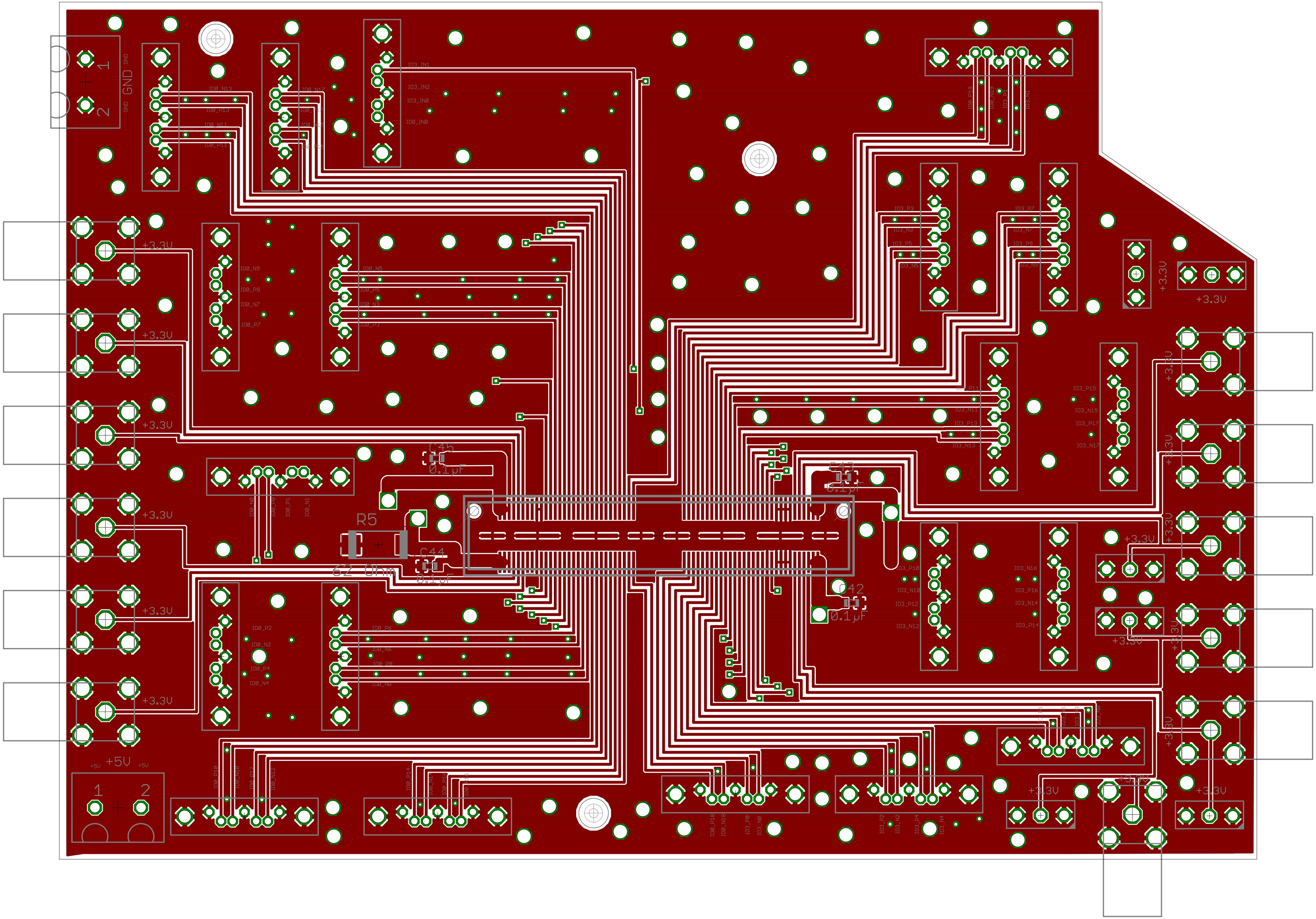

GND
+3.3V
R5
62 Ohm
C45
0.1µF
C44
0.1µF
C43
0.1µF
C42
0.1µF
+5V
