# Supplementary material for: Implications for a Wireless, External Device System to Study Electrocorticography
Source: Sensors (Basel). 2017 Apr 4;17(4):761. doi: 10.3390/s17040761 (PMC5421721; doi:10.3390/s17040761)

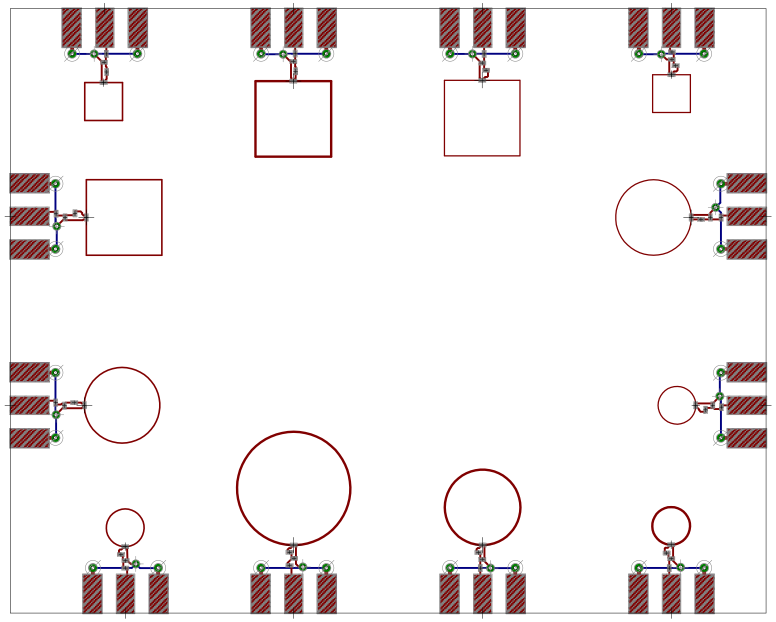

Supplement: Supplementary file 1 [file sensors-17-00761-s001.zip › Supplement/PCBs/Antenna.zip!/Antenna/ExtCoil4.pdf]
