# Supplementary material for: Implications for a Wireless, External Device System to Study Electrocorticography
Source: Sensors (Basel). 2017 Apr 4;17(4):761. doi: 10.3390/s17040761 (PMC5421721; doi:10.3390/s17040761)
Supplement: Supplementary file 1 [file sensors-17-00761-s001.zip › Supplement/PCBs/Eagle_IGLOO_02092014.zip!/IGLOO/PCB-POOL-IGLOO.pdf]

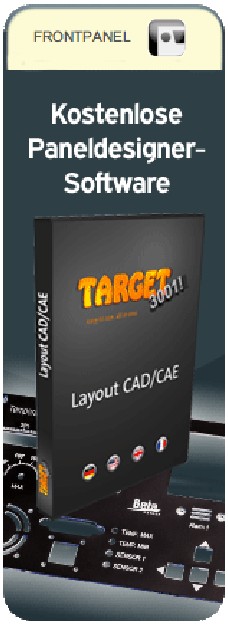

Sie befinden sich hier: **Bestellung**

LEITERPLATTENKONFIGURATION

Ihre Leiterplatte

Gewünschte Anzahl: 3

	Netto EUR	Brutto EUR
Preis pro Stück	40.64	48.36
**Warenwert gesamt**	121.92	145.08

... ab in den Warenkorb

... zum Preisvergleich

Sie können auch gern unsere ergonomische Benutzeroberfläche zur Leiterplattenkonfiguration benutzen. Bitte beachten Sie, dass dazu die Aktivierung von Javascript in Ihrem Browser notwendig ist.

Ein kleiner Tipp: Anstatt nach dem Bearbeiten einer Position auf "Speichern" zu klicken, können Sie auch auf "Bearbeiten" der nächsten Position klicken, da werden Ihre Daten ebenfalls gespeichert.

**1. Lagenzahl**	2	Bearbeiten
**2. Liefermenge**	3	Bearbeiten
**3. Abmessungen**	61mm x 110mm	Bearbeiten
**3a. Nutzen [?]**	Mindestfläche optimal ausnutzen	Bearbeiten
**4. Basismaterial [?]**	FR4, 35µmCu, 1,6mm	Bearbeiten
**5. Stopplack [?]**	ja	Bearbeiten
**6. Bestückungsdruck [?]**	nein	Bearbeiten
**7. Oberfläche [?]**	chem. Nickel/Gold (ENIG) für superflache Pads	Bearbeiten
**8. Layoutvorgaben**	Min. Leiterbahnstärke / -abstand: >= 0.125mm (5mil)<br>Min. Bohrenddurchmesser: >= 0.3mm (12mil)	Bearbeiten
**9. Überlieferung [?]**	nein	Bearbeiten
**10. Magic-PCB [?]**	nein	Bearbeiten
**11. E-Test [?]**	ja	Bearbeiten
**12. Lieferzeit [?]**	8 Arbeitstag(e)	Bearbeiten
**13. Dateiformat [?]**	EAGLE	Bearbeiten
**14. Projektname**	IGLOO	Bearbeiten
**15. Free Stencil [?]**	ja	Bearbeiten
**16. Bestückung [?]**	nein	Bearbeiten
**17. FITS-or-NOT-Nummer [?]**	Ist noch nicht gewählt!	Bearbeiten
**18. Serienproduktion?**		Bearbeiten

Ihr Kundenlogin:

E-Mail-Adresse

Passwort

Login

Passwort vergessen?

Jetzt DIREKT aus EAGLE (ab Version 5.1001) Leiterplattenpreise und Bestückung sicherer und schneller kalkulieren Download hier:

Installationsanleitung
Manual als PDF

Automatische Installation (Windows):
PCB-POOL® BUTTON

Manuelle Installation (alle unterstützten OS):
PCB-POOL® BUTTON

Beta LAYOUT GmbH | Im Aartal 14 | 65326 Aarbergen | Deutschland
Telefon: +49 (0)6120 90701.0 | Telefax: +49 (0)6120 90701.4
E-Mail: info@pcb-pool.com
kostenlose Hotline: **0800 PCBPOOL**
0800 7227665
