# Supplementary material for: Implications for a Wireless, External Device System to Study Electrocorticography
Source: Sensors (Basel). 2017 Apr 4;17(4):761. doi: 10.3390/s17040761 (PMC5421721; doi:10.3390/s17040761)
Supplement: Supplementary file 1 [file sensors-17-00761-s001.zip › Supplement/PCBs/Eagle_IGLOO_02092014.zip!/IGLOO/IGLOO_UNTEN.pdf]

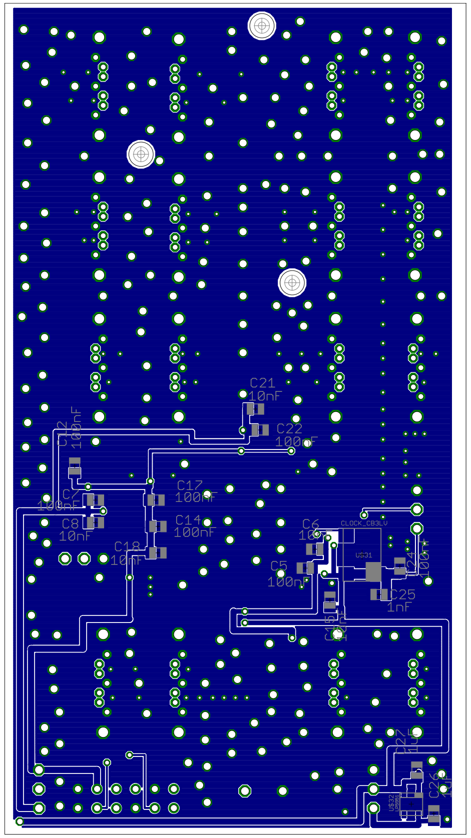
C21
10nF
C22
100nF
C12
100nF
C17
100nF
C14
100nF
C7
100nF
C8
10nF
C18
10nF
C6
C5
100nF
CLOCK_CB3LV
C25
1nF
