# Supplementary material for: Implications for a Wireless, External Device System to Study Electrocorticography
Source: Sensors (Basel). 2017 Apr 4;17(4):761. doi: 10.3390/s17040761 (PMC5421721; doi:10.3390/s17040761)
Supplement: Supplementary file 1 [file sensors-17-00761-s001.zip › Supplement/PCBs/Eagle_IGLOO_02092014.zip!/IGLOO/IGLOO_OBEN.pdf]

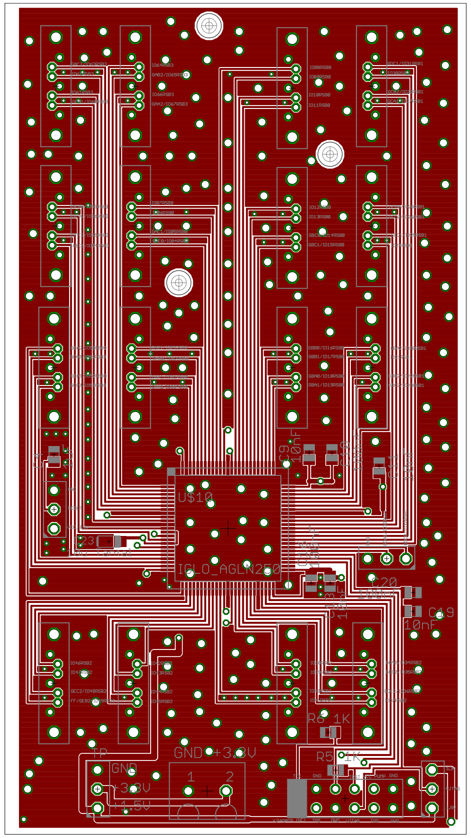

U$10
IGLO_AGLN250
C9
10nF
C19
10nF
R6 1K
R5 1K
GND +3.3V
GND
+3.3V
