# Supplementary material for: Implications for a Wireless, External Device System to Study Electrocorticography
Source: Sensors (Basel). 2017 Apr 4;17(4):761. doi: 10.3390/s17040761 (PMC5421721; doi:10.3390/s17040761)
Supplement: Supplementary file 1 [file sensors-17-00761-s001.zip › Supplement/PCBs/Eagle_RHD2132_02092014.zip!/RHD2132/PCB_TOOL.pdf]

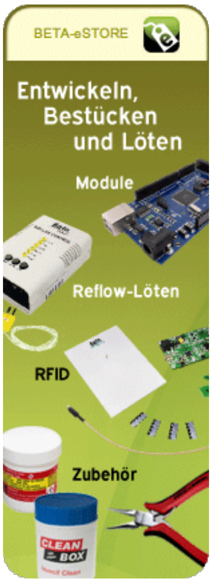

Sie befinden sich hier: **Bestellung | Leiterplatten**

LEITERPLATTENKONFIGURATION

Andere Spezifikationen? **Wechseln zur HTML-Version**

**1. Lagenzahl [?]**	○ 1 ◉ 2 ○ 4 ○ 6
**2. Liefermenge**	4
**3. Abmessungen [?]**	Länge in mm: 38 Breite in mm: 60
**3a. Nutzen**	◉ Mindestfläche optimal ausnutzen<br>○ Nutzen online konfigurieren
**4. Basismaterial [?]**	◉ FR4, 35µmCu, 1,6mm<br>○ FR4, 35µmCu, 1,0mm
**5. Stopplack [?]**	◉ ja ○ nein
**6. Bestückungsdruck [?]**	○ ja, nur BS ○ ja, nur LS<br>○ ja, BS und LS ◉ nein
**7. Oberfläche [?]**	◉ chem. Nickel/Gold (ENIG) für superflache Pads<br>○ HAL (Hot Air Leveling)
**8. Layoutvorgaben [?]**	Min. Leiterbahnstärke / -abstand:<br>◉ >= 0.125mm (5mil)<br>○ >= 0.150mm (6mil)<br>Min. Bohrenddurchmesser:<br>○ >= 0.2mm (8mil)<br>◉ >= 0.3mm (12mil)
**9. Überlieferung [?]**	○ ja, wenn vorhanden ◉ nein<br>○ ja, wenn vorhanden als Magic-PCB® - mit kostenlosem RFID Chip eingebettet (Wichtige Information)
**10. Magic-PCB [?]**	◉ nein ○ ja, ohne Anbindung<br>○ ja, Anbindung elektrisch
**11. E-Test [?]**	◉ ja ○ nein
**12. Lieferzeit in AT [?]**	○ 1 ○ 2 ○ 3 ○ 4<br>○ **5** ○ 7 ◉ **8**
**13. Dateiformat [?]**	EAGLE ▼
**14. Projektname**	RHD2132-2-SATA
**15. Free Stencil [?]**	◉ ja, ich will ○ nein
**16. Bestückung [?]**	○ ja ◉ nein<br>**Anzahl:**<br>4 zu bestückende Einzelleiterplatten<br>SMT-Bauteile pro Einzelleiterplatte [?]<br>THT-Bauteile pro Einzelleiterplatte [?]<br>**Bauteilbestückung**<br>◉ einseitig<br>○ beidseitig<br>**Bauteile [?]**<br>◉ stelle ich bei<br>○ stelle ich teilweise bei, Rest bitte bestellen*<br>○ bitte für mich bestellen *<br>* Die Bestellung erfolgt gemäß Ihrer angegebenen Lieferantenbestellnummern. Es werden 1:1 die aktuellen Listenpreise der Lieferanten berechnet.<br>**Lieferzeit der Bestückung**<br>12 ▼ AT nach Fertigstellung der Leiterplatten und nach Eintreffen der für Sie bestellten oder von Ihnen beigestellten Bauteile<br>**Preis der Bestückung**<br>167.79 EUR zuzüglich Bauteilekosten
**17. FITS-or-NOT-Nummer [?]**	
**18. Serienproduktion?**	Sind Sie zusätzlich an einem Angebot für eine höhere Stückzahl interessiert?<br>☐ ja, bitte

LADEN SIE IHRE DATEI FÜR DARSTELLUNG HOCH

Datei auswählen Keine ausgewählt

Hochladen (.brd)

BS LS

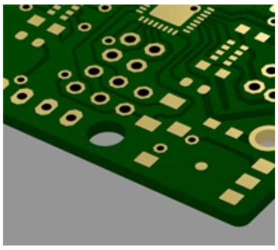

PCB

BS LS

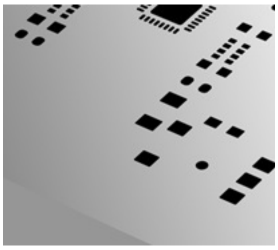

FREE Stencil

Ein kleiner Tipp: Anstatt mit der Maus zwischen den Feldern zu wechseln, können Sie mit dem Tabulator und den Pfeiltasten das Formular schneller ausfüllen.

Ihre Leiterplatte

Gewünschte Anzahl: 4

	Netto EUR	Brutto EUR
Preis pro Nutzen	20.25	24.10
**Warenwert gesamt**	**81.02**	**96.41**

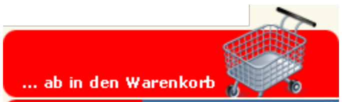

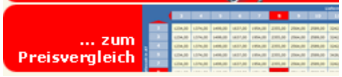

Konfiguration speichern

(c) copyright 2014, Impressum, AGB (nur für Unternehmer), Kundeninformationen, Widerrufsbelehrung, Datenschutz

Beta LAYOUT GmbH | Im Aartal 14 | 65326 Aarbergen | Deutschland
Telefon: +49 (0)6120 90701.0 | Telefax: +49 (0)6120 90701.4
E-Mail: info@pcb-pool.com
kostenlose Hotline: **0800 PCBPOOL**
0800 7227665
