# Supplementary material for: Implications for a Wireless, External Device System to Study Electrocorticography
Source: Sensors (Basel). 2017 Apr 4;17(4):761. doi: 10.3390/s17040761 (PMC5421721; doi:10.3390/s17040761)
Supplement: Supplementary file 1 [file sensors-17-00761-s001.zip › Supplement/PCBs/Eagle_RHD2132_02092014.zip!/RHD2132/RHD2132_Oben.pdf]

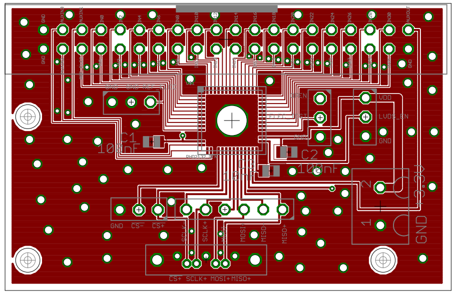
AUXIN1
IN0
IN2
IN4
IN6
IN8
IN10
IN12
IN14
IN16
IN18
IN20
IN22
IN24
IN26
IN28
IN30
AUXOUT
GND
VDD
LVDS_EN
GND
OPEN
C1
100nF
C2
100nF
C3
10nF
RHD2132_QFN
GND
CS-
CS+
SCLK-
SCLK+
MOSI-
MOSI+
MISO-
MISO+
CS+ SCLK+ MOSI+ MISO+
2
1
+3.3V
GND
