# Supplementary material for: Implications for a Wireless, External Device System to Study Electrocorticography
Source: Sensors (Basel). 2017 Apr 4;17(4):761. doi: 10.3390/s17040761 (PMC5421721; doi:10.3390/s17040761)
Supplement: Supplementary file 1 [file sensors-17-00761-s001.zip › Supplement/PCBs/Eagle_RHD2132_02092014.zip!/RHD2132/RHD2132_Unten.pdf]

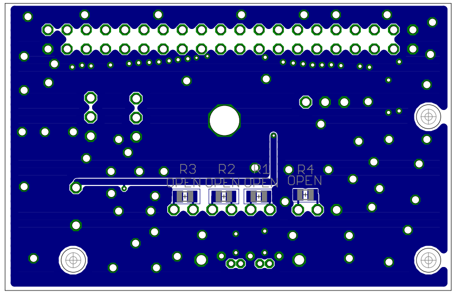
R3
OPEN
R2
OPEN
R1
OPEN
R4
OPEN
