# Supplementary material for: Implications for a Wireless, External Device System to Study Electrocorticography
Source: Sensors (Basel). 2017 Apr 4;17(4):761. doi: 10.3390/s17040761 (PMC5421721; doi:10.3390/s17040761)
Supplement: Supplementary file 1 [file sensors-17-00761-s001.zip › Supplement/RHA/IMPLANT_BASE_RHA_05092014.zip!/IMPLANT_BASE_RHA/ipcore_dir/fifo_generator_ug175.pdf]

LogiCORE IP FIFO Generator v8.1

User Guide

UG175 March 1, 2011

An updated version of UG175 is available in the Xilinx Documentation Center.
