# Supplementary material for: Implications for a Wireless, External Device System to Study Electrocorticography
Source: Sensors (Basel). 2017 Apr 4;17(4):761. doi: 10.3390/s17040761 (PMC5421721; doi:10.3390/s17040761)
Supplement: Supplementary file 1 [file sensors-17-00761-s001.zip › Supplement/RHA/IMPLANT_BASE_RHA_05092014.zip!/IMPLANT_BASE_RHA/Example2_summary.html]

Xilinx Design Summary


|  |  |  |  |
| --- | --- | --- | --- |
| **Example2 Project Status** | | | |
| **Project File:** | Example2.xise | **Parser Errors:** |  |
| **Module Name:** | Example2 | **Implementation State:** | New |
| **Target Device:** | xc3s1400a-5ft256 | - **Errors:** |  |
| **Product Version:** | ISE 14.3 | - **Warnings:** |  |
| **Design Goal:** | Balanced | - **Routing Results:** |  |
| **Design Strategy:** | Xilinx Default (unlocked) | - **Timing Constraints:** |  |
| **Environment:** |  | - **Final Timing Score:** |  |

  

|  |  |  |  |  |  |  |
| --- | --- | --- | --- | --- | --- | --- |
| **Detailed Reports** | | | | | | **[-]** |
| **Report Name** | **Status** | **Generated** | **Errors** | **Warnings** | **Infos** | |
| Synthesis Report |  |  |  |  |  | |
| Translation Report |  |  |  |  |  | |
| Map Report |  |  |  |  |  | |
| Place and Route Report |  |  |  |  |  | |
| Power Report |  |  |  |  |  | |
| Post-PAR Static Timing Report |  |  |  |  |  | |
| Bitgen Report |  |  |  |  |  | |

  

|  |  |  |  |
| --- | --- | --- | --- |
| **Secondary Reports** | | | **[-]** |
| **Report Name** | **Status** | **Generated** | |

  
**Date Generated:** 09/05/2014 - 17:38:29
