# Supplementary material for: Implications for a Wireless, External Device System to Study Electrocorticography
Source: Sensors (Basel). 2017 Apr 4;17(4):761. doi: 10.3390/s17040761 (PMC5421721; doi:10.3390/s17040761)
Supplement: Supplementary file 1 [file sensors-17-00761-s001.zip › Supplement/RHA_FPGA_Firmware/IGLOO_RHA_05092014.zip!/IGLOO_RHA/synthesis/IMPLANT_TOP.htm]

syntmp/IMPLANT\_TOP\_srr.htm log file
