# Supplementary material for: Implications for a Wireless, External Device System to Study Electrocorticography
Source: Sensors (Basel). 2017 Apr 4;17(4):761. doi: 10.3390/s17040761 (PMC5421721; doi:10.3390/s17040761)
Supplement: Supplementary file 1 [file sensors-17-00761-s001.zip › Supplement/RHA_FPGA_Firmware/IGLOO_RHA_05092014.zip!/IGLOO_RHA/synthesis/syntmp/CONTROL_NEXUS_srr.htm]

 ```` ```
#Build: Synplify Pro I-2013.09M-SP1 , Build 034R, Jan 17 2014
#install: C:\Microsemi\Libero_v11.3\Synopsys\synplify_I201309MSP1
#OS: Windows 7 6.1
#Hostname: ITP-PC

#Implementation: synthesis

$ Start of Compile
#Fri Aug 01 16:11:07 2014

Synopsys VHDL Compiler, version comp201309rcp1, Build 078R, built Jan 14 2014
@N: :  | Running in 64-bit mode 
Copyright (C) 1994-2013 Synopsys, Inc. This software and the associated documentation are proprietary to Synopsys, Inc. This software may only be used in accordance with the terms and conditions of a written license agreement with Synopsys, Inc.  All other use or distribution of the software is strictly prohibited.

@N:CD720 : std.vhd(123) | Setting time resolution to ns
@N: : CONTROL_NEXUS.vhd(28) | Top entity is set to CONTROL_NEXUS.

File Dependency file is up to date.  It will not be rewritten.

@W:CD645 : Zarlink_SPI_Module.vhd(2) | Ignoring undefined library unisim
@W:CD642 : Zarlink_SPI_Module.vhd(6) | Ignoring use clause - library unisim not found ...
@W:CD645 : ZARLINK_CONNECT_INIT.vhd(21) | Ignoring undefined library unisim
@W:CD642 : ZARLINK_CONNECT_INIT.vhd(25) | Ignoring use clause - library unisim not found ...
@W:CD645 : ORGANIZER.vhd(21) | Ignoring undefined library unisim
@W:CD642 : ORGANIZER.vhd(25) | Ignoring use clause - library unisim not found ...
@W:CD645 : COMMAND_RECEIVER.vhd(21) | Ignoring undefined library unisim
@W:CD642 : COMMAND_RECEIVER.vhd(25) | Ignoring use clause - library unisim not found ...
@W:CD645 : CONTROL_NEXUS.vhd(21) | Ignoring undefined library unisim
@W:CD642 : CONTROL_NEXUS.vhd(25) | Ignoring use clause - library unisim not found ...
VHDL syntax check successful!
@N:CD630 : CONTROL_NEXUS.vhd(28) | Synthesizing work.control_nexus.behavioral 
@W:CD638 : CONTROL_NEXUS.vhd(116) | Signal fifo_in_readenable_internal is undriven 
@N:CD630 : COMMAND_RECEIVER.vhd(28) | Synthesizing work.command_receiver.behavioral 
@N:CD231 : COMMAND_RECEIVER.vhd(85) | Using onehot encoding for type state_type (sm_idle="100000000000000000000000")
@N:CD364 : COMMAND_RECEIVER.vhd(170) | Removed redundant assignment
@N:CD364 : COMMAND_RECEIVER.vhd(171) | Removed redundant assignment
@N:CD364 : COMMAND_RECEIVER.vhd(172) | Removed redundant assignment
@N:CD364 : COMMAND_RECEIVER.vhd(173) | Removed redundant assignment
@N:CD364 : COMMAND_RECEIVER.vhd(174) | Removed redundant assignment
@N:CD364 : COMMAND_RECEIVER.vhd(175) | Removed redundant assignment
@N:CD364 : COMMAND_RECEIVER.vhd(176) | Removed redundant assignment
@W:CD604 : COMMAND_RECEIVER.vhd(406) | OTHERS clause is not synthesized 
@W:CD604 : COMMAND_RECEIVER.vhd(452) | OTHERS clause is not synthesized 
@W:CD604 : COMMAND_RECEIVER.vhd(496) | OTHERS clause is not synthesized 
@N:CD364 : COMMAND_RECEIVER.vhd(520) | Removed redundant assignment
@N:CD364 : COMMAND_RECEIVER.vhd(521) | Removed redundant assignment
@N:CD364 : COMMAND_RECEIVER.vhd(543) | Removed redundant assignment
@N:CD364 : COMMAND_RECEIVER.vhd(544) | Removed redundant assignment
Post processing for work.command_receiver.behavioral
@N:CD630 : ORGANIZER.vhd(28) | Synthesizing work.organizer.behavioral 
@N:CD231 : ORGANIZER.vhd(88) | Using onehot encoding for type state_type (sm_reset="100000000000000")
@N:CD364 : ORGANIZER.vhd(139) | Removed redundant assignment
Post processing for work.organizer.behavioral
@N:CD630 : ZARLINK_CONNECT_INIT.vhd(27) | Synthesizing work.zarlink_connect_init.behavioral 
@N:CD232 : ZARLINK_CONNECT_INIT.vhd(87) | Using gray code encoding for type state_type
@N:CD364 : ZARLINK_CONNECT_INIT.vhd(242) | Removed redundant assignment
@N:CD364 : ZARLINK_CONNECT_INIT.vhd(251) | Removed redundant assignment
@N:CD364 : ZARLINK_CONNECT_INIT.vhd(253) | Removed redundant assignment
@N:CD364 : ZARLINK_CONNECT_INIT.vhd(254) | Removed redundant assignment
@N:CD364 : ZARLINK_CONNECT_INIT.vhd(255) | Removed redundant assignment
@N:CD364 : ZARLINK_CONNECT_INIT.vhd(256) | Removed redundant assignment
@N:CD364 : ZARLINK_CONNECT_INIT.vhd(258) | Removed redundant assignment
@N:CD364 : ZARLINK_CONNECT_INIT.vhd(259) | Removed redundant assignment
@W:CD604 : ZARLINK_CONNECT_INIT.vhd(1030) | OTHERS clause is not synthesized 
@N:CD364 : ZARLINK_CONNECT_INIT.vhd(1042) | Removed redundant assignment
Post processing for work.zarlink_connect_init.behavioral
@N:CD630 : Zarlink_SPI_Module.vhd(8) | Synthesizing work.zarlink_spi_module.behavioral 
@N:CD232 : Zarlink_SPI_Module.vhd(48) | Using gray code encoding for type state_type
@N:CD364 : Zarlink_SPI_Module.vhd(131) | Removed redundant assignment
@N:CD364 : Zarlink_SPI_Module.vhd(137) | Removed redundant assignment
@N:CD364 : Zarlink_SPI_Module.vhd(138) | Removed redundant assignment
@N:CD364 : Zarlink_SPI_Module.vhd(139) | Removed redundant assignment
@N:CD364 : Zarlink_SPI_Module.vhd(142) | Removed redundant assignment
@W:CD604 : Zarlink_SPI_Module.vhd(396) | OTHERS clause is not synthesized 
Post processing for work.zarlink_spi_module.behavioral
Post processing for work.control_nexus.behavioral
@N:CL201 : Zarlink_SPI_Module.vhd(110) | Trying to extract state machine for register FSM_STATE
Extracted state machine for register FSM_STATE
State machine has 44 reachable states with original encodings of:
   000000
   000001
   000010
   000011
   000100
   000101
   000110
   000111
   001000
   001001
   001010
   001011
   001100
   001101
   001110
   001111
   010000
   010001
   010010
   010011
   010100
   010101
   010110
   010111
   011000
   011001
   011010
   011011
   011100
   011101
   011110
   011111
   110000
   110001
   110010
   110011
   110100
   110101
   110110
   110111
   111100
   111101
   111110
   111111
@N:CL201 : ZARLINK_CONNECT_INIT.vhd(207) | Trying to extract state machine for register FSM_STATE
Extracted state machine for register FSM_STATE
State machine has 36 reachable states with original encodings of:
   000000
   000001
   000010
   000011
   000100
   000101
   000110
   000111
   001000
   001001
   001010
   001011
   001100
   001101
   001110
   001111
   010000
   010001
   010010
   010011
   010100
   010101
   010110
   010111
   011000
   011001
   011010
   011011
   011100
   011101
   011110
   011111
   110000
   110001
   110010
   110011
@N:CL201 : ORGANIZER.vhd(118) | Trying to extract state machine for register SPIMACHINE_STATE
Extracted state machine for register SPIMACHINE_STATE
State machine has 15 reachable states with original encodings of:
   000000000000001
   000000000000010
   000000000000100
   000000000001000
   000000000010000
   000000000100000
   000000001000000
   000000010000000
   000000100000000
   000001000000000
   000010000000000
   000100000000000
   001000000000000
   010000000000000
   100000000000000
@N:CL201 : COMMAND_RECEIVER.vhd(157) | Trying to extract state machine for register SPIMACHINE_STATE
Extracted state machine for register SPIMACHINE_STATE
State machine has 24 reachable states with original encodings of:
   000000000000000000000001
   000000000000000000000010
   000000000000000000000100
   000000000000000000001000
   000000000000000000010000
   000000000000000000100000
   000000000000000001000000
   000000000000000010000000
   000000000000000100000000
   000000000000001000000000
   000000000000010000000000
   000000000000100000000000
   000000000001000000000000
   000000000010000000000000
   000000000100000000000000
   000000001000000000000000
   000000010000000000000000
   000000100000000000000000
   000001000000000000000000
   000010000000000000000000
   000100000000000000000000
   001000000000000000000000
   010000000000000000000000
   100000000000000000000000
@END

At c_vhdl Exit (Real Time elapsed 0h:00m:00s; CPU Time elapsed 0h:00m:00s; Memory used current: 78MB peak: 80MB)

Process took 0h:00m:01s realtime, 0h:00m:01s cputime
# Fri Aug 01 16:11:08 2014

###########################################################]
Pre-mapping Report

Synopsys Microsemi Technology Pre-mapping, Version mapact, Build 1154R, Built Jan 20 2014 10:14:08
Copyright (C) 1994-2013, Synopsys, Inc. This software and the associated documentation are proprietary to Synopsys, Inc. This software may only be used in accordance with the terms and conditions of a written license agreement with Synopsys, Inc.  All other use or distribution of the software is strictly prohibited.
Product Version I-2013.09M-SP1 

Mapper Startup Complete (Real Time elapsed 0h:00m:00s; CPU Time elapsed 0h:00m:00s; Memory used current: 98MB peak: 99MB)

Linked File: CONTROL_NEXUS_scck.rpt
Printing clock  summary report in "C:\Users\ITP\Desktop\IGLOO\IGLOO_RHA\synthesis\CONTROL_NEXUS_scck.rpt" file 
@N:MF248 :  | Running in 64-bit mode. 
@N:MF667 :  | Clock conversion disabled  

Design Input Complete (Real Time elapsed 0h:00m:00s; CPU Time elapsed 0h:00m:00s; Memory used current: 100MB peak: 102MB)


Mapper Initialization Complete (Real Time elapsed 0h:00m:00s; CPU Time elapsed 0h:00m:00s; Memory used current: 100MB peak: 102MB)


Clock Summary
**************

Start     Requested     Requested     Clock     Clock
Clock     Frequency     Period        Type      Group
-----------------------------------------------------
=====================================================

@W:MT532 : zarlink_spi_module.vhd(110) | Found signal identified as System clock which controls 501 sequential elements including Inst_Zarlink_SPI_Module.FSM_STATE[0:43].  Using this clock, which has no specified timing constraint, can adversely impact design performance. 

Finished Pre Mapping Phase.
@N:BN225 :  | Writing default property annotation file C:\Users\ITP\Desktop\IGLOO\IGLOO_RHA\synthesis\CONTROL_NEXUS.sap. 
Pre-mapping successful!

At Mapper Exit (Real Time elapsed 0h:00m:00s; CPU Time elapsed 0h:00m:00s; Memory used current: 47MB peak: 110MB)

Process took 0h:00m:01s realtime, 0h:00m:01s cputime
# Fri Aug 01 16:11:10 2014

###########################################################]
Map & Optimize Report

Synopsys Microsemi Technology Mapper, Version mapact, Build 1154R, Built Jan 20 2014 10:14:08
Copyright (C) 1994-2013, Synopsys, Inc. This software and the associated documentation are proprietary to Synopsys, Inc. This software may only be used in accordance with the terms and conditions of a written license agreement with Synopsys, Inc.  All other use or distribution of the software is strictly prohibited.
Product Version I-2013.09M-SP1 

Mapper Startup Complete (Real Time elapsed 0h:00m:00s; CPU Time elapsed 0h:00m:00s; Memory used current: 99MB peak: 99MB)

@N:MF248 :  | Running in 64-bit mode. 
@N:MF667 :  | Clock conversion disabled  

Design Input Complete (Real Time elapsed 0h:00m:00s; CPU Time elapsed 0h:00m:00s; Memory used current: 99MB peak: 101MB)


Mapper Initialization Complete (Real Time elapsed 0h:00m:00s; CPU Time elapsed 0h:00m:00s; Memory used current: 99MB peak: 101MB)


Starting Optimization and Mapping (Real Time elapsed 0h:00m:00s; CPU Time elapsed 0h:00m:00s; Memory used current: 109MB peak: 109MB)


Available hyper_sources - for debug and ip models
	None Found


Finished RTL optimizations (Real Time elapsed 0h:00m:00s; CPU Time elapsed 0h:00m:00s; Memory used current: 110MB peak: 111MB)

@N: : zarlink_spi_module.vhd(110) | Found counter in view:work.Zarlink_SPI_Module(behavioral) inst Counter[3:0]
Encoding state machine FSM_STATE[0:43] (view:work.Zarlink_SPI_Module(behavioral))
original code -> new code
   000000 -> 000000
   000001 -> 000001
   000010 -> 000011
   000011 -> 000010
   000100 -> 000110
   000101 -> 000111
   000110 -> 000101
   000111 -> 000100
   001000 -> 001100
   001001 -> 001101
   001010 -> 001111
   001011 -> 001110
   001100 -> 001010
   001101 -> 001011
   001110 -> 001001
   001111 -> 001000
   010000 -> 011000
   010001 -> 011001
   010010 -> 011011
   010011 -> 011010
   010100 -> 011110
   010101 -> 011111
   010110 -> 011101
   010111 -> 011100
   011000 -> 010100
   011001 -> 010101
   011010 -> 010111
   011011 -> 010110
   011100 -> 010010
   011101 -> 010011
   011110 -> 010001
   011111 -> 010000
   110000 -> 110000
   110001 -> 110001
   110010 -> 110011
   110011 -> 110010
   110100 -> 110110
   110101 -> 110111
   110110 -> 110101
   110111 -> 110100
   111100 -> 111100
   111101 -> 111101
   111110 -> 111111
   111111 -> 111110
@N: : zarlink_connect_init.vhd(207) | Found counter in view:work.ZARLINK_CONNECT_INIT(behavioral) inst Zarlink_ConnectionTimeoutCounter[31:0]
@N: : zarlink_connect_init.vhd(207) | Found counter in view:work.ZARLINK_CONNECT_INIT(behavioral) inst Zarlink_ResetCounter[23:0]
@N: : zarlink_connect_init.vhd(207) | Found counter in view:work.ZARLINK_CONNECT_INIT(behavioral) inst Zarlink_Retry_Counter[16:0]
Encoding state machine FSM_STATE[0:35] (view:work.ZARLINK_CONNECT_INIT(behavioral))
original code -> new code
   000000 -> 000000
   000001 -> 000001
   000010 -> 000011
   000011 -> 000010
   000100 -> 000110
   000101 -> 000111
   000110 -> 000101
   000111 -> 000100
   001000 -> 001100
   001001 -> 001101
   001010 -> 001111
   001011 -> 001110
   001100 -> 001010
   001101 -> 001011
   001110 -> 001001
   001111 -> 001000
   010000 -> 011000
   010001 -> 011001
   010010 -> 011011
   010011 -> 011010
   010100 -> 011110
   010101 -> 011111
   010110 -> 011101
   010111 -> 011100
   011000 -> 010100
   011001 -> 010101
   011010 -> 010111
   011011 -> 010110
   011100 -> 010010
   011101 -> 010011
   011110 -> 010001
   011111 -> 010000
   110000 -> 110000
   110001 -> 110001
   110010 -> 110011
   110011 -> 110010
@N: : organizer.vhd(118) | Found counter in view:work.ORGANIZER(behavioral) inst WaitForXCyles_Counter[17:0]
@N: : organizer.vhd(118) | Found counter in view:work.ORGANIZER(behavioral) inst CheckOutgoingBuffer_SIZE_COPY[6:0]
Encoding state machine SPIMACHINE_STATE[0:14] (view:work.ORGANIZER(behavioral))
original code -> new code
   000000000000001 -> 000000000000001
   000000000000010 -> 000000000000010
   000000000000100 -> 000000000000100
   000000000001000 -> 000000000001000
   000000000010000 -> 000000000010000
   000000000100000 -> 000000000100000
   000000001000000 -> 000000001000000
   000000010000000 -> 000000010000000
   000000100000000 -> 000000100000000
   000001000000000 -> 000001000000000
   000010000000000 -> 000010000000000
   000100000000000 -> 000100000000000
   001000000000000 -> 001000000000000
   010000000000000 -> 010000000000000
   100000000000000 -> 100000000000000
@N:MO106 : command_receiver.vhd(463) | Found ROM, 'BITMASK_SAMPLE_TOP_INTERNAL_16[14:1]', 16 words by 14 bits 
@N:MO106 : command_receiver.vhd(419) | Found ROM, 'BITMASK_SAMPLE_INTERNAL_17[14:9]', 16 words by 6 bits 
@N:MO106 : command_receiver.vhd(419) | Found ROM, 'BITMASK_SAMPLE_INTERNAL_17[7:1]', 16 words by 7 bits 
Encoding state machine SPIMACHINE_STATE[0:23] (view:work.COMMAND_RECEIVER(behavioral))
original code -> new code
   000000000000000000000001 -> 000000000000000000000001
   000000000000000000000010 -> 000000000000000000000010
   000000000000000000000100 -> 000000000000000000000100
   000000000000000000001000 -> 000000000000000000001000
   000000000000000000010000 -> 000000000000000000010000
   000000000000000000100000 -> 000000000000000000100000
   000000000000000001000000 -> 000000000000000001000000
   000000000000000010000000 -> 000000000000000010000000
   000000000000000100000000 -> 000000000000000100000000
   000000000000001000000000 -> 000000000000001000000000
   000000000000010000000000 -> 000000000000010000000000
   000000000000100000000000 -> 000000000000100000000000
   000000000001000000000000 -> 000000000001000000000000
   000000000010000000000000 -> 000000000010000000000000
   000000000100000000000000 -> 000000000100000000000000
   000000001000000000000000 -> 000000001000000000000000
   000000010000000000000000 -> 000000010000000000000000
   000000100000000000000000 -> 000000100000000000000000
   000001000000000000000000 -> 000001000000000000000000
   000010000000000000000000 -> 000010000000000000000000
   000100000000000000000000 -> 000100000000000000000000
   001000000000000000000000 -> 001000000000000000000000
   010000000000000000000000 -> 010000000000000000000000
   100000000000000000000000 -> 100000000000000000000000
@N:MF238 : command_receiver.vhd(530) | Found 8-bit incrementor, 'un7_counter[7:0]'
@W:BN132 : command_receiver.vhd(460) | Removing sequential instance Inst_COMMAND_RECEIVER.BITMASK_SAMPLE_TOP_INTERNAL[15],  because it is equivalent to instance Inst_COMMAND_RECEIVER.BITMASK_SAMPLE_INTERNAL[0]

Finished factoring (Real Time elapsed 0h:00m:01s; CPU Time elapsed 0h:00m:01s; Memory used current: 124MB peak: 124MB)


Finished gated-clock and generated-clock conversion (Real Time elapsed 0h:00m:01s; CPU Time elapsed 0h:00m:01s; Memory used current: 121MB peak: 124MB)


Finished generic timing optimizations - Pass 1 (Real Time elapsed 0h:00m:01s; CPU Time elapsed 0h:00m:01s; Memory used current: 119MB peak: 124MB)


Starting Early Timing Optimization (Real Time elapsed 0h:00m:01s; CPU Time elapsed 0h:00m:01s; Memory used current: 120MB peak: 124MB)


Finished Early Timing Optimization (Real Time elapsed 0h:00m:05s; CPU Time elapsed 0h:00m:05s; Memory used current: 132MB peak: 132MB)


Finished generic timing optimizations - Pass 2 (Real Time elapsed 0h:00m:05s; CPU Time elapsed 0h:00m:05s; Memory used current: 131MB peak: 132MB)


Finished preparing to map (Real Time elapsed 0h:00m:05s; CPU Time elapsed 0h:00m:05s; Memory used current: 130MB peak: 132MB)


Finished technology mapping (Real Time elapsed 0h:00m:05s; CPU Time elapsed 0h:00m:05s; Memory used current: 146MB peak: 149MB)


High Fanout Net Report
**********************

Driver Instance / Pin Name                                    Fanout, notes                   
----------------------------------------------------------------------------------------------
Inst_ZARLINK_CONNECT_INIT.GetCommand_DATA_INTERNAL[0] / Q     30                              
Inst_ZARLINK_CONNECT_INIT.GetCommand_DATA_INTERNAL[1] / Q     29                              
Inst_ZARLINK_CONNECT_INIT.GetCommand_DATA_INTERNAL[2] / Q     32                              
Inst_ZARLINK_CONNECT_INIT.GetCommand_DATA_INTERNAL[3] / Q     25                              
Inst_ZARLINK_CONNECT_INIT.GetCommand_DATA_INTERNAL[4] / Q     26                              
Inst_ZARLINK_CONNECT_INIT.FSM_STATE[0] / Q                    46                              
Inst_ZARLINK_CONNECT_INIT.FSM_STATE[1] / Q                    41                              
Inst_ZARLINK_CONNECT_INIT.FSM_STATE[2] / Q                    49                              
Inst_ZARLINK_CONNECT_INIT.FSM_STATE[3] / Q                    37                              
Inst_ZARLINK_CONNECT_INIT.FSM_STATE[4] / Q                    36                              
Inst_ZARLINK_CONNECT_INIT.FSM_STATE[5] / Q                    26                              
Inst_Zarlink_SPI_Module.FSM_STATE[0] / Q                      33                              
Inst_Zarlink_SPI_Module.FSM_STATE[3] / Q                      27                              
Inst_Zarlink_SPI_Module.FSM_STATE[4] / Q                      27                              
Inst_Zarlink_SPI_Module.FSM_STATE[5] / Q                      25                              
Reset_pad / Y                                                 434 : 432 asynchronous set/reset
Inst_COMMAND_RECEIVER.N_23_i / Y                              128                             
Inst_COMMAND_RECEIVER.CMD_RECEIVER.un1_fifo_out_valid / Y     71                              
Inst_ZARLINK_CONNECT_INIT.FSM.fsm_state155_i_i_a2_i / Y       27                              
==============================================================================================

@N:FP130 :  | Promoting Net Reset_c on CLKBUF  Reset_pad  
@N:FP130 :  | Promoting Net Clk_c on CLKBUF  Clk_pad  
@N:FP130 :  | Promoting Net Inst_COMMAND_RECEIVER.N_1075 on CLKINT  I_208  
@N:FP130 :  | Promoting Net Clock_4MHz on CLKINT  Inst_COMMAND_RECEIVER.Clk_4MHz_inferred_clock  
@N:FP130 :  | Promoting Net Inst_COMMAND_RECEIVER.CMD_RECEIVER\.un1_fifo_out_valid on CLKINT  I_209  

Finished technology timing optimizations and critical path resynthesis (Real Time elapsed 0h:00m:06s; CPU Time elapsed 0h:00m:06s; Memory used current: 148MB peak: 151MB)

Replicating Combinational Instance Inst_ZARLINK_CONNECT_INIT.FSM.fsm_state155_i_i_a2_i, fanout 27 segments 2
Replicating Sequential Instance Inst_Zarlink_SPI_Module.FSM_STATE[5], fanout 25 segments 2
Replicating Sequential Instance Inst_Zarlink_SPI_Module.FSM_STATE[4], fanout 27 segments 2
Replicating Sequential Instance Inst_Zarlink_SPI_Module.FSM_STATE[3], fanout 27 segments 2
Replicating Sequential Instance Inst_Zarlink_SPI_Module.FSM_STATE[0], fanout 33 segments 2
Replicating Sequential Instance Inst_ZARLINK_CONNECT_INIT.FSM_STATE[5], fanout 26 segments 2
Replicating Sequential Instance Inst_ZARLINK_CONNECT_INIT.FSM_STATE[4], fanout 36 segments 2
Replicating Sequential Instance Inst_ZARLINK_CONNECT_INIT.FSM_STATE[3], fanout 37 segments 2
Replicating Sequential Instance Inst_ZARLINK_CONNECT_INIT.FSM_STATE[2], fanout 49 segments 3
Replicating Sequential Instance Inst_ZARLINK_CONNECT_INIT.FSM_STATE[1], fanout 42 segments 2
Replicating Sequential Instance Inst_ZARLINK_CONNECT_INIT.FSM_STATE[0], fanout 47 segments 2
Replicating Sequential Instance Inst_ZARLINK_CONNECT_INIT.GetCommand_DATA_INTERNAL[4], fanout 26 segments 2
Replicating Sequential Instance Inst_ZARLINK_CONNECT_INIT.GetCommand_DATA_INTERNAL[3], fanout 25 segments 2
Replicating Sequential Instance Inst_ZARLINK_CONNECT_INIT.GetCommand_DATA_INTERNAL[2], fanout 32 segments 2
Replicating Sequential Instance Inst_ZARLINK_CONNECT_INIT.GetCommand_DATA_INTERNAL[1], fanout 29 segments 2
Replicating Sequential Instance Inst_ZARLINK_CONNECT_INIT.GetCommand_DATA_INTERNAL[0], fanout 30 segments 2

Added 0 Buffers
Added 17 Cells via replication
	Added 16 Sequential Cells via replication
	Added 1 Combinational Cells via replication

Finished restoring hierarchy (Real Time elapsed 0h:00m:06s; CPU Time elapsed 0h:00m:06s; Memory used current: 148MB peak: 151MB)


#### START OF CLOCK OPTIMIZATION REPORT #####[

Clock optimization not enabled
1 non-gated/non-generated clock tree(s) driving 394 clock pin(s) of sequential element(s)
1 gated/generated clock tree(s) driving 54 clock pin(s) of sequential element(s)
0 instances converted, 54 sequential instances remain driven by gated/generated clocks

====================================== Non-Gated/Non-Generated Clocks =======================================
Clock Tree ID     Driving Element     Drive Element Type     Fanout     Sample Instance                      
-------------------------------------------------------------------------------------------------------------
ClockId0002        Clk                 port                   394        Inst_COMMAND_RECEIVER.Counter_4MHz[1]
=============================================================================================================
====================================================================================== Gated/Generated Clocks ======================================================================================
Clock Tree ID     Driving Element                    Drive Element Type     Fanout     Sample Instance                      Explanation                                                             
----------------------------------------------------------------------------------------------------------------------------------------------------------------------------------------------------
ClockId0001        Inst_COMMAND_RECEIVER.Clk_4MHz     DFN1C1                 54         Inst_COMMAND_RECEIVER.Counter[7]     No generated or derived clock directive on output of sequential instance
====================================================================================================================================================================================================


##### END OF CLOCK OPTIMIZATION REPORT ######]

Writing Analyst data base C:\Users\ITP\Desktop\IGLOO\IGLOO_RHA\synthesis\CONTROL_NEXUS.srm

Finished Writing Netlist Databases (Real Time elapsed 0h:00m:06s; CPU Time elapsed 0h:00m:06s; Memory used current: 145MB peak: 151MB)

Writing EDIF Netlist and constraint files
I-2013.09M-SP1 

Finished Writing EDIF Netlist and constraint files (Real Time elapsed 0h:00m:06s; CPU Time elapsed 0h:00m:06s; Memory used current: 146MB peak: 151MB)

@W:MT420 :  | Found inferred clock CONTROL_NEXUS|Clk with period 10.00ns. Please declare a user-defined clock on object "p:Clk" 

@W:MT420 :  | Found inferred clock COMMAND_RECEIVER|Clk_4MHz_inferred_clock with period 10.00ns. Please declare a user-defined clock on object "n:Inst_COMMAND_RECEIVER.Clk_4MHz" 


##### START OF TIMING REPORT #####[
# Timing Report written on Fri Aug 01 16:11:17 2014
#


Top view:               CONTROL_NEXUS
Library name:           IGLOO_V2
Operating conditions:   COMWCSTD ( T = 70.0, V = 1.14, P = 3.70, tree_type = balanced_tree )
Requested Frequency:    100.0 MHz
Wire load mode:         top
Wire load model:        igloo
Paths requested:        5
Constraint File(s):    
@N:MT320 :  | Timing report estimates place and route data. Please look at the place and route timing report for final timing. 

@N:MT322 :  | Clock constraints cover only FF-to-FF paths associated with the clock. 


Performance Summary 
*******************


Worst slack in design: -20.980

                                             Requested     Estimated     Requested     Estimated                 Clock        Clock              
Starting Clock                               Frequency     Frequency     Period        Period        Slack       Type         Group              
-------------------------------------------------------------------------------------------------------------------------------------------------
COMMAND_RECEIVER|Clk_4MHz_inferred_clock     100.0 MHz     39.6 MHz      10.000        25.279        -15.279     inferred     Inferred_clkgroup_1
CONTROL_NEXUS|Clk                            100.0 MHz     32.3 MHz      10.000        30.980        -20.980     inferred     Inferred_clkgroup_0
=================================================================================================================================================


Clock Relationships
*******************

Clocks                                                                              |    rise  to  rise     |    fall  to  fall   |    rise  to  fall   |    fall  to  rise 
----------------------------------------------------------------------------------------------------------------------------------------------------------------------------
Starting                                  Ending                                    |  constraint  slack    |  constraint  slack  |  constraint  slack  |  constraint  slack
----------------------------------------------------------------------------------------------------------------------------------------------------------------------------
CONTROL_NEXUS|Clk                         CONTROL_NEXUS|Clk                         |  10.000      -20.980  |  No paths    -      |  No paths    -      |  No paths    -    
CONTROL_NEXUS|Clk                         COMMAND_RECEIVER|Clk_4MHz_inferred_clock  |  Diff grp    -        |  No paths    -      |  No paths    -      |  No paths    -    
COMMAND_RECEIVER|Clk_4MHz_inferred_clock  CONTROL_NEXUS|Clk                         |  Diff grp    -        |  No paths    -      |  No paths    -      |  No paths    -    
COMMAND_RECEIVER|Clk_4MHz_inferred_clock  COMMAND_RECEIVER|Clk_4MHz_inferred_clock  |  10.000      -15.279  |  No paths    -      |  No paths    -      |  No paths    -    
============================================================================================================================================================================
 Note: 'No paths' indicates there are no paths in the design for that pair of clock edges.
       'Diff grp' indicates that paths exist but the starting clock and ending clock are in different clock groups.


Interface Information 
*********************

No IO constraint found


====================================
Detailed Report for Clock: COMMAND_RECEIVER|Clk_4MHz_inferred_clock
====================================


Starting Points with Worst Slack
********************************

                                           Starting                                                                           Arrival            
Instance                                   Reference                                    Type       Pin     Net                Time        Slack  
                                           Clock                                                                                                 
-------------------------------------------------------------------------------------------------------------------------------------------------
Inst_Zarlink_SPI_Module.FSM_STATE[2]       COMMAND_RECEIVER|Clk_4MHz_inferred_clock     DFN1C1     Q       FSM_STATE[2]       1.771       -15.279
Inst_Zarlink_SPI_Module.FSM_STATE[1]       COMMAND_RECEIVER|Clk_4MHz_inferred_clock     DFN1C1     Q       FSM_STATE[1]       1.771       -15.183
Inst_Zarlink_SPI_Module.FSM_STATE[3]       COMMAND_RECEIVER|Clk_4MHz_inferred_clock     DFN1C1     Q       FSM_STATE[3]       1.771       -14.690
Inst_Zarlink_SPI_Module.FSM_STATE[0]       COMMAND_RECEIVER|Clk_4MHz_inferred_clock     DFN1C1     Q       FSM_STATE[0]       1.771       -14.134
Inst_Zarlink_SPI_Module.FSM_STATE[4]       COMMAND_RECEIVER|Clk_4MHz_inferred_clock     DFN1C1     Q       FSM_STATE[4]       1.771       -14.085
Inst_Zarlink_SPI_Module.FSM_STATE[5]       COMMAND_RECEIVER|Clk_4MHz_inferred_clock     DFN1C1     Q       FSM_STATE[5]       1.771       -13.654
Inst_Zarlink_SPI_Module.FSM_STATE_0[5]     COMMAND_RECEIVER|Clk_4MHz_inferred_clock     DFN1C1     Q       FSM_STATE_0[5]     1.771       -12.017
Inst_Zarlink_SPI_Module.FSM_STATE_0[3]     COMMAND_RECEIVER|Clk_4MHz_inferred_clock     DFN1C1     Q       FSM_STATE_0[3]     1.771       -10.138
Inst_Zarlink_SPI_Module.FSM_STATE_0[4]     COMMAND_RECEIVER|Clk_4MHz_inferred_clock     DFN1C1     Q       FSM_STATE_0[4]     1.771       -9.966 
Inst_COMMAND_RECEIVER.Counter[0]           COMMAND_RECEIVER|Clk_4MHz_inferred_clock     DFN1P1     Q       Counter[0]         1.771       -9.937 
=================================================================================================================================================


Ending Points with Worst Slack
******************************

                                                      Starting                                                                                              Required            
Instance                                              Reference                                    Type         Pin     Net                                 Time         Slack  
                                                      Clock                                                                                                                     
--------------------------------------------------------------------------------------------------------------------------------------------------------------------------------
Inst_Zarlink_SPI_Module.SPI_SDI                       COMMAND_RECEIVER|Clk_4MHz_inferred_clock     DFN1C1       D       SPI_SDI_18                          8.622        -15.279
Inst_Zarlink_SPI_Module.FSM_STATE[1]                  COMMAND_RECEIVER|Clk_4MHz_inferred_clock     DFN1C1       D       FSM_STATE_ns[1]                     8.705        -11.742
Inst_Zarlink_SPI_Module.Zarlink_Data_In_Buffer[1]     COMMAND_RECEIVER|Clk_4MHz_inferred_clock     DFN1E1C1     D       N_8                                 8.705        -11.633
Inst_Zarlink_SPI_Module.Zarlink_Data_In_Buffer[2]     COMMAND_RECEIVER|Clk_4MHz_inferred_clock     DFN1E1C1     D       N_10                                8.705        -11.633
Inst_Zarlink_SPI_Module.Zarlink_Data_In_Buffer[3]     COMMAND_RECEIVER|Clk_4MHz_inferred_clock     DFN1E1C1     D       N_12                                8.705        -11.633
Inst_Zarlink_SPI_Module.Zarlink_Data_In_Buffer[4]     COMMAND_RECEIVER|Clk_4MHz_inferred_clock     DFN1E1C1     D       N_14                                8.705        -11.633
Inst_Zarlink_SPI_Module.Zarlink_Data_In_Buffer[5]     COMMAND_RECEIVER|Clk_4MHz_inferred_clock     DFN1E1C1     D       N_16                                8.705        -11.633
Inst_Zarlink_SPI_Module.Zarlink_Data_In_Buffer[6]     COMMAND_RECEIVER|Clk_4MHz_inferred_clock     DFN1E1C1     D       N_18                                8.705        -11.633
Inst_Zarlink_SPI_Module.Zarlink_Data_In_Buffer[7]     COMMAND_RECEIVER|Clk_4MHz_inferred_clock     DFN1E1C1     D       N_20                                8.705        -11.633
Inst_Zarlink_SPI_Module.Zarlink_Data_Out_Copy[0]      COMMAND_RECEIVER|Clk_4MHz_inferred_clock     DFN1E0C1     E       Zarlink_BlockMode_Copy_RNI7SO03     8.956        -11.353
================================================================================================================================================================================


Worst Path Information
View Worst Path in Analyst
***********************


Path information for path number 1: 
      Requested Period:                      10.000
    - Setup time:                            1.378
    + Clock delay at ending point:           0.000 (ideal)
    = Required time:                         8.622

    - Propagation time:                      23.901
    - Clock delay at starting point:         0.000 (ideal)
    = Slack (non-critical) :                 -15.279

    Number of logic level(s):                6
    Starting point:                          Inst_Zarlink_SPI_Module.FSM_STATE[2] / Q
    Ending point:                            Inst_Zarlink_SPI_Module.SPI_SDI / D
    The start point is clocked by            COMMAND_RECEIVER|Clk_4MHz_inferred_clock [rising] on pin CLK
    The end   point is clocked by            COMMAND_RECEIVER|Clk_4MHz_inferred_clock [rising] on pin CLK

Instance / Net                                                Pin      Pin               Arrival     No. of    
Name                                               Type       Name     Dir     Delay     Time        Fan Out(s)
---------------------------------------------------------------------------------------------------------------
Inst_Zarlink_SPI_Module.FSM_STATE[2]               DFN1C1     Q        Out     1.771     1.771       -         
FSM_STATE[2]                                       Net        -        -       5.855     -           23        
Inst_Zarlink_SPI_Module.FSM_STATE_RNIESIE_2[4]     NOR2A      A        In      -         7.626       -         
Inst_Zarlink_SPI_Module.FSM_STATE_RNIESIE_2[4]     NOR2A      Y        Out     1.508     9.134       -         
N_223                                              Net        -        -       2.844     -           4         
Inst_Zarlink_SPI_Module.SPI_SDI_RNO_34             NOR3C      C        In      -         11.978      -         
Inst_Zarlink_SPI_Module.SPI_SDI_RNO_34             NOR3C      Y        Out     1.541     13.519      -         
SPI_SDI_18_iv_0_0_a4_7_1                           Net        -        -       0.773     -           1         
Inst_Zarlink_SPI_Module.SPI_SDI_RNO_24             AO1        C        In      -         14.291      -         
Inst_Zarlink_SPI_Module.SPI_SDI_RNO_24             AO1        Y        Out     1.520     15.811      -         
N_951_tz                                           Net        -        -       0.773     -           1         
Inst_Zarlink_SPI_Module.SPI_SDI_RNO_10             MX2        A        In      -         16.584      -         
Inst_Zarlink_SPI_Module.SPI_SDI_RNO_10             MX2        Y        Out     1.391     17.975      -         
SPI_SDI_18_iv_0_0_8                                Net        -        -       0.773     -           1         
Inst_Zarlink_SPI_Module.SPI_SDI_RNO_2              OR3        C        In      -         18.747      -         
Inst_Zarlink_SPI_Module.SPI_SDI_RNO_2              OR3        Y        Out     1.804     20.551      -         
SPI_SDI_18_iv_0_0_10                               Net        -        -       0.773     -           1         
Inst_Zarlink_SPI_Module.SPI_SDI_RNO                OR3        C        In      -         21.324      -         
Inst_Zarlink_SPI_Module.SPI_SDI_RNO                OR3        Y        Out     1.804     23.128      -         
SPI_SDI_18                                         Net        -        -       0.773     -           1         
Inst_Zarlink_SPI_Module.SPI_SDI                    DFN1C1     D        In      -         23.901      -         
===============================================================================================================
Total path delay (propagation time + setup) of 25.279 is 12.716(50.3%) logic and 12.562(49.7%) route.
Path delay compensated for clock skew. Clock skew is added to clock-to-out value, and is subtracted from setup time value


Path information for path number 2: 
      Requested Period:                      10.000
    - Setup time:                            1.295
    + Clock delay at ending point:           0.000 (ideal)
    = Required time:                         8.705

    - Propagation time:                      23.888
    - Clock delay at starting point:         0.000 (ideal)
    = Slack (non-critical) :                 -15.183

    Number of logic level(s):                6
    Starting point:                          Inst_Zarlink_SPI_Module.FSM_STATE[1] / Q
    Ending point:                            Inst_Zarlink_SPI_Module.SPI_SDI / D
    The start point is clocked by            COMMAND_RECEIVER|Clk_4MHz_inferred_clock [rising] on pin CLK
    The end   point is clocked by            COMMAND_RECEIVER|Clk_4MHz_inferred_clock [rising] on pin CLK

Instance / Net                                                Pin      Pin               Arrival     No. of    
Name                                               Type       Name     Dir     Delay     Time        Fan Out(s)
---------------------------------------------------------------------------------------------------------------
Inst_Zarlink_SPI_Module.FSM_STATE[1]               DFN1C1     Q        Out     1.771     1.771       -         
FSM_STATE[1]                                       Net        -        -       5.722     -           21        
Inst_Zarlink_SPI_Module.FSM_STATE_RNICQIE_0[3]     OR2        A        In      -         7.492       -         
Inst_Zarlink_SPI_Module.FSM_STATE_RNICQIE_0[3]     OR2        Y        Out     1.219     8.712       -         
N_521                                              Net        -        -       2.844     -           4         
Inst_Zarlink_SPI_Module.FSM_STATE_RNIPL5T[5]       NOR2       B        In      -         11.556      -         
Inst_Zarlink_SPI_Module.FSM_STATE_RNIPL5T[5]       NOR2       Y        Out     1.554     13.109      -         
N_231                                              Net        -        -       0.927     -           2         
Inst_Zarlink_SPI_Module.FSM_STATE_RNIV2F41[2]      NOR2B      A        In      -         14.037      -         
Inst_Zarlink_SPI_Module.FSM_STATE_RNIV2F41[2]      NOR2B      Y        Out     1.174     15.210      -         
FSM_STATE_211_d                                    Net        -        -       1.938     -           3         
Inst_Zarlink_SPI_Module.SPI_SDI_RNO_9              NOR2B      B        In      -         17.148      -         
Inst_Zarlink_SPI_Module.SPI_SDI_RNO_9              NOR2B      Y        Out     1.240     18.388      -         
N_598                                              Net        -        -       0.773     -           1         
Inst_Zarlink_SPI_Module.SPI_SDI_RNO_2              OR3        B        In      -         19.161      -         
Inst_Zarlink_SPI_Module.SPI_SDI_RNO_2              OR3        Y        Out     1.541     20.702      -         
SPI_SDI_18_iv_0_0_10                               Net        -        -       0.773     -           1         
Inst_Zarlink_SPI_Module.SPI_SDI_RNO                OR3        C        In      -         21.474      -         
Inst_Zarlink_SPI_Module.SPI_SDI_RNO                OR3        Y        Out     1.641     23.116      -         
SPI_SDI_18                                         Net        -        -       0.773     -           1         
Inst_Zarlink_SPI_Module.SPI_SDI                    DFN1C1     D        In      -         23.888      -         
===============================================================================================================
Total path delay (propagation time + setup) of 25.183 is 11.434(45.4%) logic and 13.749(54.6%) route.
Path delay compensated for clock skew. Clock skew is added to clock-to-out value, and is subtracted from setup time value


Path information for path number 3: 
      Requested Period:                      10.000
    - Setup time:                            1.295
    + Clock delay at ending point:           0.000 (ideal)
    = Required time:                         8.705

    - Propagation time:                      23.416
    - Clock delay at starting point:         0.000 (ideal)
    = Slack (non-critical) :                 -14.711

    Number of logic level(s):                6
    Starting point:                          Inst_Zarlink_SPI_Module.FSM_STATE[2] / Q
    Ending point:                            Inst_Zarlink_SPI_Module.SPI_SDI / D
    The start point is clocked by            COMMAND_RECEIVER|Clk_4MHz_inferred_clock [rising] on pin CLK
    The end   point is clocked by            COMMAND_RECEIVER|Clk_4MHz_inferred_clock [rising] on pin CLK

Instance / Net                                                Pin      Pin               Arrival     No. of    
Name                                               Type       Name     Dir     Delay     Time        Fan Out(s)
---------------------------------------------------------------------------------------------------------------
Inst_Zarlink_SPI_Module.FSM_STATE[2]               DFN1C1     Q        Out     1.771     1.771       -         
FSM_STATE[2]                                       Net        -        -       5.855     -           23        
Inst_Zarlink_SPI_Module.FSM_STATE_RNIESIE_3[4]     NOR2       A        In      -         7.626       -         
Inst_Zarlink_SPI_Module.FSM_STATE_RNIESIE_3[4]     NOR2       Y        Out     1.219     8.845       -         
N_220                                              Net        -        -       2.844     -           4         
Inst_Zarlink_SPI_Module.SPI_SDI_RNO_36             NOR3C      C        In      -         11.690      -         
Inst_Zarlink_SPI_Module.SPI_SDI_RNO_36             NOR3C      Y        Out     1.599     13.289      -         
SPI_SDI_18_iv_0_0_a4_9_1                           Net        -        -       0.773     -           1         
Inst_Zarlink_SPI_Module.SPI_SDI_RNO_25             AO1        C        In      -         14.062      -         
Inst_Zarlink_SPI_Module.SPI_SDI_RNO_25             AO1        Y        Out     1.574     15.636      -         
N_950_tz                                           Net        -        -       0.773     -           1         
Inst_Zarlink_SPI_Module.SPI_SDI_RNO_10             MX2        B        In      -         16.409      -         
Inst_Zarlink_SPI_Module.SPI_SDI_RNO_10             MX2        Y        Out     1.407     17.816      -         
SPI_SDI_18_iv_0_0_8                                Net        -        -       0.773     -           1         
Inst_Zarlink_SPI_Module.SPI_SDI_RNO_2              OR3        C        In      -         18.589      -         
Inst_Zarlink_SPI_Module.SPI_SDI_RNO_2              OR3        Y        Out     1.641     20.230      -         
SPI_SDI_18_iv_0_0_10                               Net        -        -       0.773     -           1         
Inst_Zarlink_SPI_Module.SPI_SDI_RNO                OR3        C        In      -         21.003      -         
Inst_Zarlink_SPI_Module.SPI_SDI_RNO                OR3        Y        Out     1.641     22.644      -         
SPI_SDI_18                                         Net        -        -       0.773     -           1         
Inst_Zarlink_SPI_Module.SPI_SDI                    DFN1C1     D        In      -         23.416      -         
===============================================================================================================
Total path delay (propagation time + setup) of 24.711 is 12.149(49.2%) logic and 12.562(50.8%) route.
Path delay compensated for clock skew. Clock skew is added to clock-to-out value, and is subtracted from setup time value


Path information for path number 4: 
      Requested Period:                      10.000
    - Setup time:                            1.295
    + Clock delay at ending point:           0.000 (ideal)
    = Required time:                         8.705

    - Propagation time:                      23.396
    - Clock delay at starting point:         0.000 (ideal)
    = Slack (non-critical) :                 -14.690

    Number of logic level(s):                6
    Starting point:                          Inst_Zarlink_SPI_Module.FSM_STATE[3] / Q
    Ending point:                            Inst_Zarlink_SPI_Module.SPI_SDI / D
    The start point is clocked by            COMMAND_RECEIVER|Clk_4MHz_inferred_clock [rising] on pin CLK
    The end   point is clocked by            COMMAND_RECEIVER|Clk_4MHz_inferred_clock [rising] on pin CLK

Instance / Net                                                Pin      Pin               Arrival     No. of    
Name                                               Type       Name     Dir     Delay     Time        Fan Out(s)
---------------------------------------------------------------------------------------------------------------
Inst_Zarlink_SPI_Module.FSM_STATE[3]               DFN1C1     Q        Out     1.771     1.771       -         
FSM_STATE[3]                                       Net        -        -       4.895     -           13        
Inst_Zarlink_SPI_Module.FSM_STATE_RNICQIE_0[3]     OR2        B        In      -         6.665       -         
Inst_Zarlink_SPI_Module.FSM_STATE_RNICQIE_0[3]     OR2        Y        Out     1.554     8.219       -         
N_521                                              Net        -        -       2.844     -           4         
Inst_Zarlink_SPI_Module.FSM_STATE_RNIPL5T[5]       NOR2       B        In      -         11.063      -         
Inst_Zarlink_SPI_Module.FSM_STATE_RNIPL5T[5]       NOR2       Y        Out     1.554     12.617      -         
N_231                                              Net        -        -       0.927     -           2         
Inst_Zarlink_SPI_Module.FSM_STATE_RNIV2F41[2]      NOR2B      A        In      -         13.544      -         
Inst_Zarlink_SPI_Module.FSM_STATE_RNIV2F41[2]      NOR2B      Y        Out     1.174     14.717      -         
FSM_STATE_211_d                                    Net        -        -       1.938     -           3         
Inst_Zarlink_SPI_Module.SPI_SDI_RNO_9              NOR2B      B        In      -         16.655      -         
Inst_Zarlink_SPI_Module.SPI_SDI_RNO_9              NOR2B      Y        Out     1.240     17.895      -         
N_598                                              Net        -        -       0.773     -           1         
Inst_Zarlink_SPI_Module.SPI_SDI_RNO_2              OR3        B        In      -         18.668      -         
Inst_Zarlink_SPI_Module.SPI_SDI_RNO_2              OR3        Y        Out     1.541     20.209      -         
SPI_SDI_18_iv_0_0_10                               Net        -        -       0.773     -           1         
Inst_Zarlink_SPI_Module.SPI_SDI_RNO                OR3        C        In      -         20.982      -         
Inst_Zarlink_SPI_Module.SPI_SDI_RNO                OR3        Y        Out     1.641     22.623      -         
SPI_SDI_18                                         Net        -        -       0.773     -           1         
Inst_Zarlink_SPI_Module.SPI_SDI                    DFN1C1     D        In      -         23.396      -         
===============================================================================================================
Total path delay (propagation time + setup) of 24.690 is 11.769(47.7%) logic and 12.922(52.3%) route.
Path delay compensated for clock skew. Clock skew is added to clock-to-out value, and is subtracted from setup time value


Path information for path number 5: 
      Requested Period:                      10.000
    - Setup time:                            1.378
    + Clock delay at ending point:           0.000 (ideal)
    = Required time:                         8.622

    - Propagation time:                      22.756
    - Clock delay at starting point:         0.000 (ideal)
    = Slack (non-critical) :                 -14.134

    Number of logic level(s):                6
    Starting point:                          Inst_Zarlink_SPI_Module.FSM_STATE[0] / Q
    Ending point:                            Inst_Zarlink_SPI_Module.SPI_SDI / D
    The start point is clocked by            COMMAND_RECEIVER|Clk_4MHz_inferred_clock [rising] on pin CLK
    The end   point is clocked by            COMMAND_RECEIVER|Clk_4MHz_inferred_clock [rising] on pin CLK

Instance / Net                                               Pin      Pin               Arrival     No. of    
Name                                              Type       Name     Dir     Delay     Time        Fan Out(s)
--------------------------------------------------------------------------------------------------------------
Inst_Zarlink_SPI_Module.FSM_STATE[0]              DFN1C1     Q        Out     1.771     1.771       -         
FSM_STATE[0]                                      Net        -        -       5.220     -           16        
Inst_Zarlink_SPI_Module.FSM_STATE_RNIDRIE[5]      OR2B       B        In      -         6.991       -         
Inst_Zarlink_SPI_Module.FSM_STATE_RNIDRIE[5]      OR2B       Y        Out     1.508     8.499       -         
N_58                                              Net        -        -       1.938     -           3         
Inst_Zarlink_SPI_Module.FSM_STATE_RNIPL5T[5]      NOR2       A        In      -         10.437      -         
Inst_Zarlink_SPI_Module.FSM_STATE_RNIPL5T[5]      NOR2       Y        Out     0.873     11.309      -         
N_231                                             Net        -        -       0.927     -           2         
Inst_Zarlink_SPI_Module.FSM_STATE_RNIV2F41[2]     NOR2B      A        In      -         12.236      -         
Inst_Zarlink_SPI_Module.FSM_STATE_RNIV2F41[2]     NOR2B      Y        Out     1.236     13.473      -         
FSM_STATE_211_d                                   Net        -        -       1.938     -           3         
Inst_Zarlink_SPI_Module.SPI_SDI_RNO_9             NOR2B      B        In      -         15.411      -         
Inst_Zarlink_SPI_Module.SPI_SDI_RNO_9             NOR2B      Y        Out     1.508     16.918      -         
N_598                                             Net        -        -       0.773     -           1         
Inst_Zarlink_SPI_Module.SPI_SDI_RNO_2             OR3        B        In      -         17.691      -         
Inst_Zarlink_SPI_Module.SPI_SDI_RNO_2             OR3        Y        Out     1.716     19.407      -         
SPI_SDI_18_iv_0_0_10                              Net        -        -       0.773     -           1         
Inst_Zarlink_SPI_Module.SPI_SDI_RNO               OR3        C        In      -         20.180      -         
Inst_Zarlink_SPI_Module.SPI_SDI_RNO               OR3        Y        Out     1.804     21.984      -         
SPI_SDI_18                                        Net        -        -       0.773     -           1         
Inst_Zarlink_SPI_Module.SPI_SDI                   DFN1C1     D        In      -         22.756      -         
==============================================================================================================
Total path delay (propagation time + setup) of 24.134 is 11.794(48.9%) logic and 12.341(51.1%) route.
Path delay compensated for clock skew. Clock skew is added to clock-to-out value, and is subtracted from setup time value


====================================
Detailed Report for Clock: CONTROL_NEXUS|Clk
====================================


Starting Points with Worst Slack
********************************

                                                                   Starting                                                                            Arrival            
Instance                                                           Reference             Type         Pin     Net                                      Time        Slack  
                                                                   Clock                                                                                                  
--------------------------------------------------------------------------------------------------------------------------------------------------------------------------
Inst_ZARLINK_CONNECT_INIT.FSM_STATE[3]                             CONTROL_NEXUS|Clk     DFN1C1       Q       FSM_STATE[3]                             1.771       -20.980
Inst_ZARLINK_CONNECT_INIT.Zarlink_TimeOut                          CONTROL_NEXUS|Clk     DFN1E1C1     Q       Zarlink_TimeOut                          1.771       -20.691
Inst_ZARLINK_CONNECT_INIT.FSM_STATE[4]                             CONTROL_NEXUS|Clk     DFN1C1       Q       FSM_STATE[4]                             1.771       -20.645
Inst_ZARLINK_CONNECT_INIT.Zarlink_ConnectionTimeoutCounter[7]      CONTROL_NEXUS|Clk     DFN1C1       Q       Zarlink_ConnectionTimeoutCounter[7]      1.395       -20.291
Inst_ZARLINK_CONNECT_INIT.Zarlink_ConnectionTimeoutCounter[5]      CONTROL_NEXUS|Clk     DFN1C1       Q       Zarlink_ConnectionTimeoutCounter[5]      1.771       -20.224
Inst_ZARLINK_CONNECT_INIT.Zarlink_ConnectionTimeoutCounter[8]      CONTROL_NEXUS|Clk     DFN1C1       Q       Zarlink_ConnectionTimeoutCounter[8]      1.771       -19.977
Inst_ZARLINK_CONNECT_INIT.Zarlink_ConnectionTimeoutCounter[11]     CONTROL_NEXUS|Clk     DFN1C1       Q       Zarlink_ConnectionTimeoutCounter[11]     1.395       -19.096
Inst_ZARLINK_CONNECT_INIT.Zarlink_ConnectionTimeoutCounter[12]     CONTROL_NEXUS|Clk     DFN1C1       Q       Zarlink_ConnectionTimeoutCounter[12]     1.395       -18.858
Inst_ZARLINK_CONNECT_INIT.Zarlink_Retry_Counter[6]                 CONTROL_NEXUS|Clk     DFN1C1       Q       Zarlink_Retry_Counter[6]                 1.771       -18.403
Inst_ZARLINK_CONNECT_INIT.Zarlink_Retry_Counter[7]                 CONTROL_NEXUS|Clk     DFN1C1       Q       Zarlink_Retry_Counter[7]                 1.771       -18.131
==========================================================================================================================================================================


Ending Points with Worst Slack
******************************

                                                                   Starting                                                                          Required            
Instance                                                           Reference             Type       Pin     Net                                      Time         Slack  
                                                                   Clock                                                                                                 
-------------------------------------------------------------------------------------------------------------------------------------------------------------------------
Inst_ZARLINK_CONNECT_INIT.Z_Done                                   CONTROL_NEXUS|Clk     DFN1C1     D       Z_Done_RNO                               8.705        -20.980
Inst_ZARLINK_CONNECT_INIT.Zarlink_ConnectionTimeoutCounter[21]     CONTROL_NEXUS|Clk     DFN1C1     D       Zarlink_ConnectionTimeoutCounter_n21     8.622        -20.291
Inst_ZARLINK_CONNECT_INIT.Z_Address[4]                             CONTROL_NEXUS|Clk     DFN1C1     D       Z_Address_50[4]                          8.705        -19.744
Inst_ZARLINK_CONNECT_INIT.ZDOR[0]                                  CONTROL_NEXUS|Clk     DFN1C1     D       ZDOR_33[0]                               8.705        -19.497
Inst_ZARLINK_CONNECT_INIT.Z_Address[0]                             CONTROL_NEXUS|Clk     DFN1C1     D       Z_Address_50[0]                          8.705        -19.497
Inst_ZARLINK_CONNECT_INIT.Z_WE                                     CONTROL_NEXUS|Clk     DFN1C1     D       Z_WE_3                                   8.705        -19.405
Inst_ZARLINK_CONNECT_INIT.ZDOR[1]                                  CONTROL_NEXUS|Clk     DFN1C1     D       ZDOR_33[1]                               8.622        -19.146
Inst_ZARLINK_CONNECT_INIT.ZDOR[2]                                  CONTROL_NEXUS|Clk     DFN1C1     D       ZDOR_33[2]                               8.622        -19.050
Inst_ZARLINK_CONNECT_INIT.Zarlink_ConnectionTimeoutCounter[17]     CONTROL_NEXUS|Clk     DFN1C1     D       Zarlink_ConnectionTimeoutCounter_n17     8.705        -18.800
Inst_ZARLINK_CONNECT_INIT.Zarlink_ConnectionTimeoutCounter[20]     CONTROL_NEXUS|Clk     DFN1C1     D       Zarlink_ConnectionTimeoutCounter_n20     8.622        -18.720
=========================================================================================================================================================================


Worst Path Information
View Worst Path in Analyst
***********************


Path information for path number 1: 
      Requested Period:                      10.000
    - Setup time:                            1.295
    + Clock delay at ending point:           0.000 (ideal)
    = Required time:                         8.705

    - Propagation time:                      29.685
    - Clock delay at starting point:         0.000 (ideal)
    = Slack (critical) :                     -20.980

    Number of logic level(s):                7
    Starting point:                          Inst_ZARLINK_CONNECT_INIT.FSM_STATE[3] / Q
    Ending point:                            Inst_ZARLINK_CONNECT_INIT.Z_Done / D
    The start point is clocked by            CONTROL_NEXUS|Clk [rising] on pin CLK
    The end   point is clocked by            CONTROL_NEXUS|Clk [rising] on pin CLK

Instance / Net                                                   Pin      Pin               Arrival     No. of    
Name                                                  Type       Name     Dir     Delay     Time        Fan Out(s)
------------------------------------------------------------------------------------------------------------------
Inst_ZARLINK_CONNECT_INIT.FSM_STATE[3]                DFN1C1     Q        Out     1.771     1.771       -         
FSM_STATE[3]                                          Net        -        -       5.438     -           18        
Inst_ZARLINK_CONNECT_INIT.FSM_STATE_RNI7IG3_3[3]      NOR2       B        In      -         7.208       -         
Inst_ZARLINK_CONNECT_INIT.FSM_STATE_RNI7IG3_3[3]      NOR2       Y        Out     1.554     8.762       -         
FSM_m1_e_1_1                                          Net        -        -       4.268     -           11        
Inst_ZARLINK_CONNECT_INIT.FSM_STATE_1_RNIV37Q[2]      NOR3B      B        In      -         13.030      -         
Inst_ZARLINK_CONNECT_INIT.FSM_STATE_1_RNIV37Q[2]      NOR3B      Y        Out     1.499     14.529      -         
Z_N_5                                                 Net        -        -       0.773     -           1         
Inst_ZARLINK_CONNECT_INIT.FSM_STATE_0_RNIAOK12[0]     OA1B       A        In      -         15.302      -         
Inst_ZARLINK_CONNECT_INIT.FSM_STATE_0_RNIAOK12[0]     OA1B       Y        Out     2.163     17.465      -         
N_471                                                 Net        -        -       1.938     -           3         
Inst_ZARLINK_CONNECT_INIT.FSM_STATE_0_RNIRQCV2[0]     OR2        B        In      -         19.403      -         
Inst_ZARLINK_CONNECT_INIT.FSM_STATE_0_RNIRQCV2[0]     OR2        Y        Out     1.236     20.639      -         
N_316                                                 Net        -        -       1.938     -           3         
Inst_ZARLINK_CONNECT_INIT.Z_Done_RNO_6                AO1        C        In      -         22.577      -         
Inst_ZARLINK_CONNECT_INIT.Z_Done_RNO_6                AO1        Y        Out     1.574     24.151      -         
Z_Done_279_0_0_3                                      Net        -        -       0.773     -           1         
Inst_ZARLINK_CONNECT_INIT.Z_Done_RNO_2                AO1        C        In      -         24.924      -         
Inst_ZARLINK_CONNECT_INIT.Z_Done_RNO_2                AO1        Y        Out     1.574     26.499      -         
Z_Done_279_0_0_5                                      Net        -        -       0.773     -           1         
Inst_ZARLINK_CONNECT_INIT.Z_Done_RNO                  OR3        C        In      -         27.271      -         
Inst_ZARLINK_CONNECT_INIT.Z_Done_RNO                  OR3        Y        Out     1.641     28.912      -         
Z_Done_RNO                                            Net        -        -       0.773     -           1         
Inst_ZARLINK_CONNECT_INIT.Z_Done                      DFN1C1     D        In      -         29.685      -         
==================================================================================================================
Total path delay (propagation time + setup) of 30.980 is 14.308(46.2%) logic and 16.672(53.8%) route.
Path delay compensated for clock skew. Clock skew is added to clock-to-out value, and is subtracted from setup time value


Path information for path number 2: 
      Requested Period:                      10.000
    - Setup time:                            1.295
    + Clock delay at ending point:           0.000 (ideal)
    = Required time:                         8.705

    - Propagation time:                      29.397
    - Clock delay at starting point:         0.000 (ideal)
    = Slack (non-critical) :                 -20.691

    Number of logic level(s):                7
    Starting point:                          Inst_ZARLINK_CONNECT_INIT.Zarlink_TimeOut / Q
    Ending point:                            Inst_ZARLINK_CONNECT_INIT.Z_Done / D
    The start point is clocked by            CONTROL_NEXUS|Clk [rising] on pin CLK
    The end   point is clocked by            CONTROL_NEXUS|Clk [rising] on pin CLK

Instance / Net                                                     Pin      Pin               Arrival     No. of    
Name                                                  Type         Name     Dir     Delay     Time        Fan Out(s)
--------------------------------------------------------------------------------------------------------------------
Inst_ZARLINK_CONNECT_INIT.Zarlink_TimeOut             DFN1E1C1     Q        Out     1.771     1.771       -         
Zarlink_TimeOut                                       Net          -        -       5.926     -           24        
Inst_ZARLINK_CONNECT_INIT.Zarlink_TimeOut_RNI83NM     OR2          B        In      -         7.697       -         
Inst_ZARLINK_CONNECT_INIT.Zarlink_TimeOut_RNI83NM     OR2          Y        Out     1.554     9.251       -         
N_294                                                 Net          -        -       4.790     -           12        
Inst_ZARLINK_CONNECT_INIT.FSM_STATE_RNIFL7Q_2[3]      NOR2         B        In      -         14.041      -         
Inst_ZARLINK_CONNECT_INIT.FSM_STATE_RNIFL7Q_2[3]      NOR2         Y        Out     1.554     15.594      -         
N_638                                                 Net          -        -       1.938     -           3         
Inst_ZARLINK_CONNECT_INIT.FSM_STATE_RNIH2OT[2]        NOR2B        A        In      -         17.532      -         
Inst_ZARLINK_CONNECT_INIT.FSM_STATE_RNIH2OT[2]        NOR2B        Y        Out     1.174     18.706      -         
N_592                                                 Net          -        -       0.773     -           1         
Inst_ZARLINK_CONNECT_INIT.FSM_STATE_0_RNIRQCV2[0]     OR2          A        In      -         19.478      -         
Inst_ZARLINK_CONNECT_INIT.FSM_STATE_0_RNIRQCV2[0]     OR2          Y        Out     0.873     20.351      -         
N_316                                                 Net          -        -       1.938     -           3         
Inst_ZARLINK_CONNECT_INIT.Z_Done_RNO_6                AO1          C        In      -         22.289      -         
Inst_ZARLINK_CONNECT_INIT.Z_Done_RNO_6                AO1          Y        Out     1.574     23.863      -         
Z_Done_279_0_0_3                                      Net          -        -       0.773     -           1         
Inst_ZARLINK_CONNECT_INIT.Z_Done_RNO_2                AO1          C        In      -         24.636      -         
Inst_ZARLINK_CONNECT_INIT.Z_Done_RNO_2                AO1          Y        Out     1.574     26.210      -         
Z_Done_279_0_0_5                                      Net          -        -       0.773     -           1         
Inst_ZARLINK_CONNECT_INIT.Z_Done_RNO                  OR3          C        In      -         26.983      -         
Inst_ZARLINK_CONNECT_INIT.Z_Done_RNO                  OR3          Y        Out     1.641     28.624      -         
Z_Done_RNO                                            Net          -        -       0.773     -           1         
Inst_ZARLINK_CONNECT_INIT.Z_Done                      DFN1C1       D        In      -         29.397      -         
====================================================================================================================
Total path delay (propagation time + setup) of 30.691 is 13.009(42.4%) logic and 17.683(57.6%) route.
Path delay compensated for clock skew. Clock skew is added to clock-to-out value, and is subtracted from setup time value


Path information for path number 3: 
      Requested Period:                      10.000
    - Setup time:                            1.295
    + Clock delay at ending point:           0.000 (ideal)
    = Required time:                         8.705

    - Propagation time:                      29.351
    - Clock delay at starting point:         0.000 (ideal)
    = Slack (non-critical) :                 -20.645

    Number of logic level(s):                7
    Starting point:                          Inst_ZARLINK_CONNECT_INIT.FSM_STATE[4] / Q
    Ending point:                            Inst_ZARLINK_CONNECT_INIT.Z_Done / D
    The start point is clocked by            CONTROL_NEXUS|Clk [rising] on pin CLK
    The end   point is clocked by            CONTROL_NEXUS|Clk [rising] on pin CLK

Instance / Net                                                   Pin      Pin               Arrival     No. of    
Name                                                  Type       Name     Dir     Delay     Time        Fan Out(s)
------------------------------------------------------------------------------------------------------------------
Inst_ZARLINK_CONNECT_INIT.FSM_STATE[4]                DFN1C1     Q        Out     1.771     1.771       -         
FSM_STATE[4]                                          Net        -        -       5.438     -           18        
Inst_ZARLINK_CONNECT_INIT.FSM_STATE_RNI7IG3_3[3]      NOR2       A        In      -         7.208       -         
Inst_ZARLINK_CONNECT_INIT.FSM_STATE_RNI7IG3_3[3]      NOR2       Y        Out     1.219     8.428       -         
FSM_m1_e_1_1                                          Net        -        -       4.268     -           11        
Inst_ZARLINK_CONNECT_INIT.FSM_STATE_1_RNIV37Q[2]      NOR3B      B        In      -         12.696      -         
Inst_ZARLINK_CONNECT_INIT.FSM_STATE_1_RNIV37Q[2]      NOR3B      Y        Out     1.499     14.195      -         
Z_N_5                                                 Net        -        -       0.773     -           1         
Inst_ZARLINK_CONNECT_INIT.FSM_STATE_0_RNIAOK12[0]     OA1B       A        In      -         14.968      -         
Inst_ZARLINK_CONNECT_INIT.FSM_STATE_0_RNIAOK12[0]     OA1B       Y        Out     2.163     17.131      -         
N_471                                                 Net        -        -       1.938     -           3         
Inst_ZARLINK_CONNECT_INIT.FSM_STATE_0_RNIRQCV2[0]     OR2        B        In      -         19.069      -         
Inst_ZARLINK_CONNECT_INIT.FSM_STATE_0_RNIRQCV2[0]     OR2        Y        Out     1.236     20.305      -         
N_316                                                 Net        -        -       1.938     -           3         
Inst_ZARLINK_CONNECT_INIT.Z_Done_RNO_6                AO1        C        In      -         22.243      -         
Inst_ZARLINK_CONNECT_INIT.Z_Done_RNO_6                AO1        Y        Out     1.574     23.817      -         
Z_Done_279_0_0_3                                      Net        -        -       0.773     -           1         
Inst_ZARLINK_CONNECT_INIT.Z_Done_RNO_2                AO1        C        In      -         24.590      -         
Inst_ZARLINK_CONNECT_INIT.Z_Done_RNO_2                AO1        Y        Out     1.574     26.164      -         
Z_Done_279_0_0_5                                      Net        -        -       0.773     -           1         
Inst_ZARLINK_CONNECT_INIT.Z_Done_RNO                  OR3        C        In      -         26.937      -         
Inst_ZARLINK_CONNECT_INIT.Z_Done_RNO                  OR3        Y        Out     1.641     28.578      -         
Z_Done_RNO                                            Net        -        -       0.773     -           1         
Inst_ZARLINK_CONNECT_INIT.Z_Done                      DFN1C1     D        In      -         29.351      -         
==================================================================================================================
Total path delay (propagation time + setup) of 30.645 is 13.974(45.6%) logic and 16.672(54.4%) route.
Path delay compensated for clock skew. Clock skew is added to clock-to-out value, and is subtracted from setup time value


Path information for path number 4: 
      Requested Period:                      10.000
    - Setup time:                            1.378
    + Clock delay at ending point:           0.000 (ideal)
    = Required time:                         8.622

    - Propagation time:                      28.913
    - Clock delay at starting point:         0.000 (ideal)
    = Slack (non-critical) :                 -20.291

    Number of logic level(s):                8
    Starting point:                          Inst_ZARLINK_CONNECT_INIT.Zarlink_ConnectionTimeoutCounter[7] / Q
    Ending point:                            Inst_ZARLINK_CONNECT_INIT.Zarlink_ConnectionTimeoutCounter[21] / D
    The start point is clocked by            CONTROL_NEXUS|Clk [rising] on pin CLK
    The end   point is clocked by            CONTROL_NEXUS|Clk [rising] on pin CLK

Instance / Net                                                                         Pin      Pin               Arrival     No. of    
Name                                                                        Type       Name     Dir     Delay     Time        Fan Out(s)
----------------------------------------------------------------------------------------------------------------------------------------
Inst_ZARLINK_CONNECT_INIT.Zarlink_ConnectionTimeoutCounter[7]               DFN1C1     Q        Out     1.395     1.395       -         
Zarlink_ConnectionTimeoutCounter[7]                                         Net        -        -       3.938     -           8         
Inst_ZARLINK_CONNECT_INIT.Zarlink_ConnectionTimeoutCounter_RNITI1G[11]      NOR3C      C        In      -         5.333       -         
Inst_ZARLINK_CONNECT_INIT.Zarlink_ConnectionTimeoutCounter_RNITI1G[11]      NOR3C      Y        Out     1.599     6.933       -         
Zarlink_m6_0_a2_7_4                                                         Net        -        -       0.773     -           1         
Inst_ZARLINK_CONNECT_INIT.Zarlink_ConnectionTimeoutCounter_RNI26M21[9]      NOR3C      C        In      -         7.705       -         
Inst_ZARLINK_CONNECT_INIT.Zarlink_ConnectionTimeoutCounter_RNI26M21[9]      NOR3C      Y        Out     1.599     9.305       -         
Zarlink_m6_0_a2_7_6                                                         Net        -        -       0.773     -           1         
Inst_ZARLINK_CONNECT_INIT.Zarlink_ConnectionTimeoutCounter_RNIGG022[10]     NOR2B      A        In      -         10.077      -         
Inst_ZARLINK_CONNECT_INIT.Zarlink_ConnectionTimeoutCounter_RNIGG022[10]     NOR2B      Y        Out     1.174     11.251      -         
Zarlink_m6_0_a2_7                                                           Net        -        -       3.074     -           5         
Inst_ZARLINK_CONNECT_INIT.Zarlink_ConnectionTimeoutCounter_RNI2U873[10]     NOR2B      A        In      -         14.325      -         
Inst_ZARLINK_CONNECT_INIT.Zarlink_ConnectionTimeoutCounter_RNI2U873[10]     NOR2B      Y        Out     1.174     15.498      -         
Zarlink_N_13_mux                                                            Net        -        -       2.844     -           4         
Inst_ZARLINK_CONNECT_INIT.Zarlink_ConnectionTimeoutCounter_RNINO3O3[13]     NOR3C      C        In      -         18.342      -         
Inst_ZARLINK_CONNECT_INIT.Zarlink_ConnectionTimeoutCounter_RNINO3O3[13]     NOR3C      Y        Out     1.599     19.942      -         
Zarlink_N_5_mux_0_0                                                         Net        -        -       1.938     -           3         
Inst_ZARLINK_CONNECT_INIT.Zarlink_ConnectionTimeoutCounter_RNI0HRU3[19]     OR3C       B        In      -         21.880      -         
Inst_ZARLINK_CONNECT_INIT.Zarlink_ConnectionTimeoutCounter_RNI0HRU3[19]     OR3C       Y        Out     1.499     23.379      -         
N_58                                                                        Net        -        -       0.927     -           2         
Inst_ZARLINK_CONNECT_INIT.Zarlink_ConnectionTimeoutCounter_RNO_0[21]        OR2A       B        In      -         24.306      -         
Inst_ZARLINK_CONNECT_INIT.Zarlink_ConnectionTimeoutCounter_RNO_0[21]        OR2A       Y        Out     1.554     25.860      -         
N_59                                                                        Net        -        -       0.773     -           1         
Inst_ZARLINK_CONNECT_INIT.Zarlink_ConnectionTimeoutCounter_RNO[21]          XA1C       B        In      -         26.632      -         
Inst_ZARLINK_CONNECT_INIT.Zarlink_ConnectionTimeoutCounter_RNO[21]          XA1C       Y        Out     1.508     28.140      -         
Zarlink_ConnectionTimeoutCounter_n21                                        Net        -        -       0.773     -           1         
Inst_ZARLINK_CONNECT_INIT.Zarlink_ConnectionTimeoutCounter[21]              DFN1C1     D        In      -         28.913      -         
========================================================================================================================================
Total path delay (propagation time + setup) of 30.291 is 14.479(47.8%) logic and 15.812(52.2%) route.
Path delay compensated for clock skew. Clock skew is added to clock-to-out value, and is subtracted from setup time value


Path information for path number 5: 
      Requested Period:                      10.000
    - Setup time:                            1.378
    + Clock delay at ending point:           0.000 (ideal)
    = Required time:                         8.622

    - Propagation time:                      28.846
    - Clock delay at starting point:         0.000 (ideal)
    = Slack (non-critical) :                 -20.224

    Number of logic level(s):                8
    Starting point:                          Inst_ZARLINK_CONNECT_INIT.Zarlink_ConnectionTimeoutCounter[5] / Q
    Ending point:                            Inst_ZARLINK_CONNECT_INIT.Zarlink_ConnectionTimeoutCounter[21] / D
    The start point is clocked by            CONTROL_NEXUS|Clk [rising] on pin CLK
    The end   point is clocked by            CONTROL_NEXUS|Clk [rising] on pin CLK

Instance / Net                                                                         Pin      Pin               Arrival     No. of    
Name                                                                        Type       Name     Dir     Delay     Time        Fan Out(s)
----------------------------------------------------------------------------------------------------------------------------------------
Inst_ZARLINK_CONNECT_INIT.Zarlink_ConnectionTimeoutCounter[5]               DFN1C1     Q        Out     1.771     1.771       -         
Zarlink_ConnectionTimeoutCounter[5]                                         Net        -        -       3.938     -           8         
Inst_ZARLINK_CONNECT_INIT.Zarlink_ConnectionTimeoutCounter_RNI3HKI[8]       NOR2B      A        In      -         5.709       -         
Inst_ZARLINK_CONNECT_INIT.Zarlink_ConnectionTimeoutCounter_RNI3HKI[8]       NOR2B      Y        Out     1.236     6.945       -         
Zarlink_m6_0_a2_7_2                                                         Net        -        -       0.773     -           1         
Inst_ZARLINK_CONNECT_INIT.Zarlink_ConnectionTimeoutCounter_RNIEAAV[10]      NOR3C      C        In      -         7.718       -         
Inst_ZARLINK_CONNECT_INIT.Zarlink_ConnectionTimeoutCounter_RNIEAAV[10]      NOR3C      Y        Out     1.541     9.259       -         
Zarlink_m6_0_a2_7_5                                                         Net        -        -       0.773     -           1         
Inst_ZARLINK_CONNECT_INIT.Zarlink_ConnectionTimeoutCounter_RNIGG022[10]     NOR2B      B        In      -         10.031      -         
Inst_ZARLINK_CONNECT_INIT.Zarlink_ConnectionTimeoutCounter_RNIGG022[10]     NOR2B      Y        Out     1.508     11.539      -         
Zarlink_m6_0_a2_7                                                           Net        -        -       3.074     -           5         
Inst_ZARLINK_CONNECT_INIT.Zarlink_ConnectionTimeoutCounter_RNI2U873[10]     NOR2B      A        In      -         14.613      -         
Inst_ZARLINK_CONNECT_INIT.Zarlink_ConnectionTimeoutCounter_RNI2U873[10]     NOR2B      Y        Out     1.236     15.849      -         
Zarlink_N_13_mux                                                            Net        -        -       2.844     -           4         
Inst_ZARLINK_CONNECT_INIT.Zarlink_ConnectionTimeoutCounter_RNINO3O3[13]     NOR3C      C        In      -         18.693      -         
Inst_ZARLINK_CONNECT_INIT.Zarlink_ConnectionTimeoutCounter_RNINO3O3[13]     NOR3C      Y        Out     1.541     20.234      -         
Zarlink_N_5_mux_0_0                                                         Net        -        -       1.938     -           3         
Inst_ZARLINK_CONNECT_INIT.Zarlink_ConnectionTimeoutCounter_RNI0HRU3[19]     OR3C       B        In      -         22.172      -         
Inst_ZARLINK_CONNECT_INIT.Zarlink_ConnectionTimeoutCounter_RNI0HRU3[19]     OR3C       Y        Out     1.458     23.629      -         
N_58                                                                        Net        -        -       0.927     -           2         
Inst_ZARLINK_CONNECT_INIT.Zarlink_ConnectionTimeoutCounter_RNO_0[21]        OR2A       B        In      -         24.557      -         
Inst_ZARLINK_CONNECT_INIT.Zarlink_ConnectionTimeoutCounter_RNO_0[21]        OR2A       Y        Out     1.236     25.793      -         
N_59                                                                        Net        -        -       0.773     -           1         
Inst_ZARLINK_CONNECT_INIT.Zarlink_ConnectionTimeoutCounter_RNO[21]          XA1C       B        In      -         26.565      -         
Inst_ZARLINK_CONNECT_INIT.Zarlink_ConnectionTimeoutCounter_RNO[21]          XA1C       Y        Out     1.508     28.073      -         
Zarlink_ConnectionTimeoutCounter_n21                                        Net        -        -       0.773     -           1         
Inst_ZARLINK_CONNECT_INIT.Zarlink_ConnectionTimeoutCounter[21]              DFN1C1     D        In      -         28.846      -         
========================================================================================================================================
Total path delay (propagation time + setup) of 30.224 is 14.412(47.7%) logic and 15.812(52.3%) route.
Path delay compensated for clock skew. Clock skew is added to clock-to-out value, and is subtracted from setup time value


##### END OF TIMING REPORT #####]

--------------------------------------------------------------------------------
Target Part: AGL250V2_VQFP100_STD
Report for cell CONTROL_NEXUS.behavioral
  Core Cell usage:
              cell count     area count*area
              AND2     9      1.0        9.0
              AND3     6      1.0        6.0
               AO1    57      1.0       57.0
              AO1A    32      1.0       32.0
              AO1B     4      1.0        4.0
              AO1C     8      1.0        8.0
              AO1D     8      1.0        8.0
              AOI1    17      1.0       17.0
             AOI1B     2      1.0        2.0
               AX1     1      1.0        1.0
              AX1A     4      1.0        4.0
              AX1C     5      1.0        5.0
              AX1E     2      1.0        2.0
            CLKINT     3      0.0        0.0
               GND     5      0.0        0.0
               INV     3      1.0        3.0
               MX2   132      1.0      132.0
              MX2A     2      1.0        2.0
              MX2B     1      1.0        1.0
              NOR2    54      1.0       54.0
             NOR2A   129      1.0      129.0
             NOR2B   156      1.0      156.0
              NOR3    26      1.0       26.0
             NOR3A    65      1.0       65.0
             NOR3B    90      1.0       90.0
             NOR3C    97      1.0       97.0
               OA1    15      1.0       15.0
              OA1A    13      1.0       13.0
              OA1B    11      1.0       11.0
              OA1C    25      1.0       25.0
              OAI1     5      1.0        5.0
               OR2    63      1.0       63.0
              OR2A    42      1.0       42.0
              OR2B    35      1.0       35.0
               OR3    70      1.0       70.0
              OR3A    15      1.0       15.0
              OR3B    17      1.0       17.0
              OR3C    15      1.0       15.0
               VCC     5      0.0        0.0
               XA1    20      1.0       20.0
              XA1A    21      1.0       21.0
              XA1B     3      1.0        3.0
              XA1C     8      1.0        8.0
             XNOR2     6      1.0        6.0
               XO1     5      1.0        5.0
              XOR2    26      1.0       26.0


            DFN1C1   178      1.0      178.0
          DFN1E0C1    12      1.0       12.0
          DFN1E1C1   213      1.0      213.0
          DFN1E1P1     6      1.0        6.0
            DFN1P1    39      1.0       39.0
                   -----          ----------
             TOTAL  1786              1773.0


  IO Cell usage:
              cell count
            CLKBUF     2
             INBUF    10
            OUTBUF   175
                   -----
             TOTAL   187


Core Cells         : 1773 of 6144 (29%)
IO Cells           : 187

  RAM/ROM Usage Summary
Block Rams : 0 of 8 (0%)

Mapper successful!

At Mapper Exit (Real Time elapsed 0h:00m:07s; CPU Time elapsed 0h:00m:06s; Memory used current: 53MB peak: 151MB)

Process took 0h:00m:07s realtime, 0h:00m:06s cputime
# Fri Aug 01 16:11:17 2014

###########################################################]
``` ````
