# Supplementary material for: Implications for a Wireless, External Device System to Study Electrocorticography
Source: Sensors (Basel). 2017 Apr 4;17(4):761. doi: 10.3390/s17040761 (PMC5421721; doi:10.3390/s17040761)
Supplement: Supplementary file 1 [file sensors-17-00761-s001.zip › Supplement/RHA_FPGA_Firmware/IGLOO_RHA_05092014.zip!/IGLOO_RHA/synthesis/syntmp/DATA_ACQUISITION_BLOCK_srr.htm]

 ```` ```
#Build: Synplify Pro I-2013.09M-SP1 , Build 034R, Jan 17 2014
#install: C:\Microsemi\Libero_v11.3\Synopsys\synplify_I201309MSP1
#OS: Windows 7 6.1
#Hostname: ITP-PC

#Implementation: synthesis

$ Start of Compile
#Fri Aug 01 16:09:12 2014

Synopsys VHDL Compiler, version comp201309rcp1, Build 078R, built Jan 14 2014
@N: :  | Running in 64-bit mode 
Copyright (C) 1994-2013 Synopsys, Inc. This software and the associated documentation are proprietary to Synopsys, Inc. This software may only be used in accordance with the terms and conditions of a written license agreement with Synopsys, Inc.  All other use or distribution of the software is strictly prohibited.

@N:CD720 : std.vhd(123) | Setting time resolution to ns
@N: : DATA_ACQUISITION_BLOCK.vhd(27) | Top entity is set to DATA_ACQUISITION_BLOCK.
@W:CD645 : RHA_DATA_COLLECTOR.vhd(21) | Ignoring undefined library unisim
@W:CD642 : RHA_DATA_COLLECTOR.vhd(25) | Ignoring use clause - library unisim not found ...
@W:CD645 : RHA_DATA_CHECKER.vhd(21) | Ignoring undefined library unisim
@W:CD642 : RHA_DATA_CHECKER.vhd(25) | Ignoring use clause - library unisim not found ...
@W:CD645 : RHA_TO_ZL_CONVERTER.vhd(21) | Ignoring undefined library unisim
@W:CD642 : RHA_TO_ZL_CONVERTER.vhd(25) | Ignoring use clause - library unisim not found ...
@W:CD645 : RHA_TESTVALUE_GENERATOR.vhd(21) | Ignoring undefined library unisim
@W:CD642 : RHA_TESTVALUE_GENERATOR.vhd(25) | Ignoring use clause - library unisim not found ...
@W:CD645 : RHA_TEST_MODULATOR.vhd(21) | Ignoring undefined library unisim
@W:CD642 : RHA_TEST_MODULATOR.vhd(25) | Ignoring use clause - library unisim not found ...
@W:CD645 : RHA_ERROR_WATCHDOG.vhd(21) | Ignoring undefined library unisim
@W:CD642 : RHA_ERROR_WATCHDOG.vhd(25) | Ignoring use clause - library unisim not found ...
@W:CD645 : RHA_ARRAY.vhd(21) | Ignoring undefined library unisim
@W:CD642 : RHA_ARRAY.vhd(25) | Ignoring use clause - library unisim not found ...
@W:CD645 : RHA_TESTMODULE.vhd(21) | Ignoring undefined library unisim
@W:CD642 : RHA_TESTMODULE.vhd(25) | Ignoring use clause - library unisim not found ...
@W:CD645 : DATA_ACQUISITION_BLOCK.vhd(21) | Ignoring undefined library unisim
@W:CD642 : DATA_ACQUISITION_BLOCK.vhd(25) | Ignoring use clause - library unisim not found ...
VHDL syntax check successful!
@N:CD630 : DATA_ACQUISITION_BLOCK.vhd(27) | Synthesizing work.data_acquisition_block.behavioral 
@N:CD630 : RHA_ERROR_WATCHDOG.vhd(27) | Synthesizing work.rha_error_watchdog.behavioral 
@N:CD364 : RHA_ERROR_WATCHDOG.vhd(95) | Removed redundant assignment
@N:CD364 : RHA_ERROR_WATCHDOG.vhd(96) | Removed redundant assignment
@N:CD364 : RHA_ERROR_WATCHDOG.vhd(97) | Removed redundant assignment
@N:CD364 : RHA_ERROR_WATCHDOG.vhd(98) | Removed redundant assignment
@N:CD364 : RHA_ERROR_WATCHDOG.vhd(99) | Removed redundant assignment
@N:CD364 : RHA_ERROR_WATCHDOG.vhd(100) | Removed redundant assignment
@N:CD364 : RHA_ERROR_WATCHDOG.vhd(101) | Removed redundant assignment
Post processing for work.rha_error_watchdog.behavioral
@N:CD630 : RHA_TESTMODULE.vhd(28) | Synthesizing work.rha_testmodule.behavioral 
@N:CD630 : RHA_TEST_MODULATOR.vhd(27) | Synthesizing work.rha_test_modulator.behavioral 
@N:CD232 : RHA_TEST_MODULATOR.vhd(43) | Using gray code encoding for type state_type
@N:CD364 : RHA_TEST_MODULATOR.vhd(87) | Removed redundant assignment
@N:CD364 : RHA_TEST_MODULATOR.vhd(88) | Removed redundant assignment
@W:CD604 : RHA_TEST_MODULATOR.vhd(146) | OTHERS clause is not synthesized 
@W:CD604 : RHA_TEST_MODULATOR.vhd(220) | OTHERS clause is not synthesized 
Post processing for work.rha_test_modulator.behavioral
@A:CL282 : RHA_TEST_MODULATOR.vhd(154) | Feedback mux created for signal SPIMACHINE_STATE[4:0] -- possible set/reset assignment for signal missing. Specifying a reset value will improve timing and area.
@N:CD630 : RHA_TESTVALUE_GENERATOR.vhd(27) | Synthesizing work.rha_testvalue_generator.behavioral 
@N:CD364 : RHA_TESTVALUE_GENERATOR.vhd(130) | Removed redundant assignment
@N:CD364 : RHA_TESTVALUE_GENERATOR.vhd(138) | Removed redundant assignment
@N:CD364 : RHA_TESTVALUE_GENERATOR.vhd(142) | Removed redundant assignment
@N:CD364 : RHA_TESTVALUE_GENERATOR.vhd(145) | Removed redundant assignment
@N:CD364 : RHA_TESTVALUE_GENERATOR.vhd(146) | Removed redundant assignment
@W:CD604 : RHA_TESTVALUE_GENERATOR.vhd(205) | OTHERS clause is not synthesized 
@W:CD638 : RHA_TESTVALUE_GENERATOR.vhd(60) | Signal xor_address is undriven 
@W:CD638 : RHA_TESTVALUE_GENERATOR.vhd(61) | Signal xor_header is undriven 
@W:CD638 : RHA_TESTVALUE_GENERATOR.vhd(82) | Signal nextvalue_last is undriven 
Post processing for work.rha_testvalue_generator.behavioral
Post processing for work.rha_testmodule.behavioral
@N:CD630 : RHA_TO_ZL_CONVERTER.vhd(28) | Synthesizing work.rha_to_zl_converter.behavioral 
@N:CD232 : RHA_TO_ZL_CONVERTER.vhd(112) | Using gray code encoding for type state_type
@N:CD364 : RHA_TO_ZL_CONVERTER.vhd(261) | Removed redundant assignment
@N:CD364 : RHA_TO_ZL_CONVERTER.vhd(262) | Removed redundant assignment
@N:CD364 : RHA_TO_ZL_CONVERTER.vhd(300) | Removed redundant assignment
@N:CD364 : RHA_TO_ZL_CONVERTER.vhd(328) | Removed redundant assignment
@N:CD364 : RHA_TO_ZL_CONVERTER.vhd(329) | Removed redundant assignment
@N:CD364 : RHA_TO_ZL_CONVERTER.vhd(331) | Removed redundant assignment
@N:CD364 : RHA_TO_ZL_CONVERTER.vhd(332) | Removed redundant assignment
@N:CD364 : RHA_TO_ZL_CONVERTER.vhd(333) | Removed redundant assignment
@N:CD364 : RHA_TO_ZL_CONVERTER.vhd(334) | Removed redundant assignment
@N:CD364 : RHA_TO_ZL_CONVERTER.vhd(336) | Removed redundant assignment
@N:CD364 : RHA_TO_ZL_CONVERTER.vhd(348) | Removed redundant assignment
@N:CD364 : RHA_TO_ZL_CONVERTER.vhd(583) | Removed redundant assignment
@W:CD604 : RHA_TO_ZL_CONVERTER.vhd(615) | OTHERS clause is not synthesized 
Post processing for work.rha_to_zl_converter.behavioral
@A:CL282 : RHA_TO_ZL_CONVERTER.vhd(314) | Feedback mux created for signal SPIMACHINE_STATE[4:0] -- possible set/reset assignment for signal missing. Specifying a reset value will improve timing and area.
@A:CL282 : RHA_TO_ZL_CONVERTER.vhd(295) | Feedback mux created for signal NewBlock_LAST -- possible set/reset assignment for signal missing. Specifying a reset value will improve timing and area.
@N:CD630 : RHA_ARRAY.vhd(32) | Synthesizing work.rha_array.behavioral 
@N:CD364 : RHA_ARRAY.vhd(450) | Removed redundant assignment
@N:CD364 : RHA_ARRAY.vhd(451) | Removed redundant assignment
@N:CD364 : RHA_ARRAY.vhd(452) | Removed redundant assignment
@N:CD364 : RHA_ARRAY.vhd(453) | Removed redundant assignment
@N:CD364 : RHA_ARRAY.vhd(454) | Removed redundant assignment
@N:CD364 : RHA_ARRAY.vhd(459) | Removed redundant assignment
@N:CD364 : RHA_ARRAY.vhd(461) | Removed redundant assignment
@N:CD364 : RHA_ARRAY.vhd(463) | Removed redundant assignment
@N:CD364 : RHA_ARRAY.vhd(464) | Removed redundant assignment
@N:CD364 : RHA_ARRAY.vhd(465) | Removed redundant assignment
@N:CD364 : RHA_ARRAY.vhd(466) | Removed redundant assignment
@N:CD364 : RHA_ARRAY.vhd(467) | Removed redundant assignment
@N:CD364 : RHA_ARRAY.vhd(468) | Removed redundant assignment
@N:CD364 : RHA_ARRAY.vhd(469) | Removed redundant assignment
@N:CD364 : RHA_ARRAY.vhd(470) | Removed redundant assignment
@N:CD364 : RHA_ARRAY.vhd(565) | Removed redundant assignment
@N:CD364 : RHA_ARRAY.vhd(566) | Removed redundant assignment
@W:CD638 : RHA_ARRAY.vhd(155) | Signal channel_number is undriven 
@N:CD630 : RHA_DATA_CHECKER.vhd(36) | Synthesizing work.rha_data_checker.behavioral 
Post processing for work.rha_data_checker.behavioral
@N:CD630 : RHA_DATA_COLLECTOR.vhd(30) | Synthesizing work.rha_data_collector.behavioral 
@N:CD233 : RHA_DATA_COLLECTOR.vhd(57) | Using sequential encoding for type state_type
@N:CD364 : RHA_DATA_COLLECTOR.vhd(93) | Removed redundant assignment
@N:CD364 : RHA_DATA_COLLECTOR.vhd(96) | Removed redundant assignment
@W:CD604 : RHA_DATA_COLLECTOR.vhd(128) | OTHERS clause is not synthesized 
@N:CD364 : RHA_DATA_COLLECTOR.vhd(149) | Removed redundant assignment
Post processing for work.rha_data_collector.behavioral
@A:CL282 : RHA_DATA_COLLECTOR.vhd(67) | Feedback mux created for signal SPIMACHINE_STATE[0:0] -- possible set/reset assignment for signal missing. Specifying a reset value will improve timing and area.
Post processing for work.rha_array.behavioral
@W:CL190 : RHA_ARRAY.vhd(151) | Optimizing register bit AddressCheckInfo(3) to a constant 0
@W:CL260 : RHA_ARRAY.vhd(151) | Pruning register bit 3 of AddressCheckInfo(79 downto 0)  
Post processing for work.data_acquisition_block.behavioral
@W:CL117 : DATA_ACQUISITION_BLOCK.vhd(154) | Latch generated from process for signal RHA_ADC_clk_TEST; possible missing assignment in an if or case statement.
@W:CL117 : DATA_ACQUISITION_BLOCK.vhd(82) | Latch generated from process for signal RHA_ADC_clk(7 downto 0); possible missing assignment in an if or case statement.
@W:CL189 : RHA_DATA_COLLECTOR.vhd(67) | Register bit SPIMACHINE_STATE(sm_wait) is always 1, optimizing ...
@W:CL190 : RHA_ARRAY.vhd(151) | Optimizing register bit AddressCheckInfo(8) to a constant 0
@W:CL260 : RHA_ARRAY.vhd(151) | Pruning register bit 8 of AddressCheckInfo(79 downto 4)  
@W:CL190 : RHA_ARRAY.vhd(151) | Optimizing register bit AddressCheckInfo(13) to a constant 0
@W:CL260 : RHA_ARRAY.vhd(151) | Pruning register bit 13 of AddressCheckInfo(79 downto 9)  
@W:CL190 : RHA_ARRAY.vhd(151) | Optimizing register bit AddressCheckInfo(18) to a constant 0
@W:CL260 : RHA_ARRAY.vhd(151) | Pruning register bit 18 of AddressCheckInfo(79 downto 14)  
@W:CL190 : RHA_ARRAY.vhd(151) | Optimizing register bit AddressCheckInfo(23) to a constant 0
@W:CL260 : RHA_ARRAY.vhd(151) | Pruning register bit 23 of AddressCheckInfo(79 downto 19)  
@W:CL279 : RHA_ARRAY.vhd(151) | Pruning register bits 2 to 1 of AddressCheckInfo(2 downto 0)  
@W:CL260 : RHA_ARRAY.vhd(151) | Pruning register bit 7 of AddressCheckInfo(7 downto 4)  
@W:CL190 : RHA_ARRAY.vhd(151) | Optimizing register bit AddressCheckInfo(28) to a constant 0
@W:CL260 : RHA_ARRAY.vhd(151) | Pruning register bit 28 of AddressCheckInfo(79 downto 24)  
@N:CL201 : RHA_TO_ZL_CONVERTER.vhd(314) | Trying to extract state machine for register SPIMACHINE_STATE
@W:CL159 : RHA_TO_ZL_CONVERTER.vhd(72) | Input Clk_SLOW is unused
@N:CL201 : RHA_TEST_MODULATOR.vhd(154) | Trying to extract state machine for register SPIMACHINE_STATE
@END

At c_vhdl Exit (Real Time elapsed 0h:00m:00s; CPU Time elapsed 0h:00m:00s; Memory used current: 77MB peak: 82MB)

Process took 0h:00m:01s realtime, 0h:00m:01s cputime
# Fri Aug 01 16:09:13 2014

###########################################################]
Pre-mapping Report

Synopsys Microsemi Technology Pre-mapping, Version mapact, Build 1154R, Built Jan 20 2014 10:14:08
Copyright (C) 1994-2013, Synopsys, Inc. This software and the associated documentation are proprietary to Synopsys, Inc. This software may only be used in accordance with the terms and conditions of a written license agreement with Synopsys, Inc.  All other use or distribution of the software is strictly prohibited.
Product Version I-2013.09M-SP1 

Mapper Startup Complete (Real Time elapsed 0h:00m:00s; CPU Time elapsed 0h:00m:00s; Memory used current: 99MB peak: 99MB)

Linked File: DATA_ACQUISITION_BLOCK_scck.rpt
Printing clock  summary report in "C:\Users\ITP\Desktop\IGLOO\IGLOO_RHA\synthesis\DATA_ACQUISITION_BLOCK_scck.rpt" file 
@N:MF248 :  | Running in 64-bit mode. 
@N:MF667 :  | Clock conversion disabled  

Design Input Complete (Real Time elapsed 0h:00m:00s; CPU Time elapsed 0h:00m:00s; Memory used current: 101MB peak: 103MB)


Mapper Initialization Complete (Real Time elapsed 0h:00m:00s; CPU Time elapsed 0h:00m:00s; Memory used current: 101MB peak: 103MB)

@N:BN362 : rha_test_modulator.vhd(79) | Removing sequential instance NextValue of view:PrimLib.dffr(prim) in hierarchy view:work.RHA_TEST_MODULATOR_RHA_TEST_MOD_1(behavioral) because there are no references to its outputs 
@N:BN362 : rha_test_modulator.vhd(79) | Removing sequential instance NextValue of view:PrimLib.dffr(prim) in hierarchy view:work.RHA_TEST_MODULATOR_RHA_TEST_MOD_2(behavioral) because there are no references to its outputs 
@N:BN362 : rha_test_modulator.vhd(79) | Removing sequential instance NextValue of view:PrimLib.dffr(prim) in hierarchy view:work.RHA_TEST_MODULATOR_RHA_TEST_MOD_3(behavioral) because there are no references to its outputs 
@N:BN362 : rha_test_modulator.vhd(79) | Removing sequential instance NextValue of view:PrimLib.dffr(prim) in hierarchy view:work.RHA_TEST_MODULATOR_RHA_TEST_MOD_4(behavioral) because there are no references to its outputs 
@N:BN362 : rha_test_modulator.vhd(79) | Removing sequential instance NextValue of view:PrimLib.dffr(prim) in hierarchy view:work.RHA_TEST_MODULATOR_RHA_TEST_MOD_5(behavioral) because there are no references to its outputs 
@N:BN362 : rha_test_modulator.vhd(79) | Removing sequential instance NextValue of view:PrimLib.dffr(prim) in hierarchy view:work.RHA_TEST_MODULATOR_RHA_TEST_MOD_6(behavioral) because there are no references to its outputs 
@N:BN362 : rha_test_modulator.vhd(79) | Removing sequential instance NextValue of view:PrimLib.dffr(prim) in hierarchy view:work.RHA_TEST_MODULATOR_RHA_TEST_MOD_7(behavioral) because there are no references to its outputs 
@N:BN362 : rha_test_modulator.vhd(154) | Removing sequential instance ADC_SYNC of view:PrimLib.dffr(prim) in hierarchy view:work.RHA_TEST_MODULATOR_RHA_TEST_MOD_0(behavioral) because there are no references to its outputs 
@N:BN362 : rha_test_modulator.vhd(154) | Removing sequential instance ADC_SYNC of view:PrimLib.dffr(prim) in hierarchy view:work.RHA_TEST_MODULATOR_RHA_TEST_MOD_1(behavioral) because there are no references to its outputs 
@N:BN362 : rha_test_modulator.vhd(154) | Removing sequential instance ADC_SYNC of view:PrimLib.dffr(prim) in hierarchy view:work.RHA_TEST_MODULATOR_RHA_TEST_MOD_2(behavioral) because there are no references to its outputs 
@N:BN362 : rha_test_modulator.vhd(154) | Removing sequential instance ADC_SYNC of view:PrimLib.dffr(prim) in hierarchy view:work.RHA_TEST_MODULATOR_RHA_TEST_MOD_3(behavioral) because there are no references to its outputs 
@N:BN362 : rha_test_modulator.vhd(154) | Removing sequential instance ADC_SYNC of view:PrimLib.dffr(prim) in hierarchy view:work.RHA_TEST_MODULATOR_RHA_TEST_MOD_4(behavioral) because there are no references to its outputs 
@N:BN362 : rha_test_modulator.vhd(154) | Removing sequential instance ADC_SYNC of view:PrimLib.dffr(prim) in hierarchy view:work.RHA_TEST_MODULATOR_RHA_TEST_MOD_5(behavioral) because there are no references to its outputs 
@N:BN362 : rha_test_modulator.vhd(154) | Removing sequential instance ADC_SYNC of view:PrimLib.dffr(prim) in hierarchy view:work.RHA_TEST_MODULATOR_RHA_TEST_MOD_6(behavioral) because there are no references to its outputs 
@N:BN362 : rha_test_modulator.vhd(154) | Removing sequential instance ADC_SYNC of view:PrimLib.dffr(prim) in hierarchy view:work.RHA_TEST_MODULATOR_RHA_TEST_MOD_7(behavioral) because there are no references to its outputs 


Clock Summary
**************

Start     Requested     Requested     Clock     Clock
Clock     Frequency     Period        Type      Group
-----------------------------------------------------
=====================================================

@W:MT532 : rha_data_collector.vhd(67) | Found signal identified as System clock which controls 1121 sequential elements including Inst_RHA_ARRAY.RHA0.Counter[4:0].  Using this clock, which has no specified timing constraint, can adversely impact design performance. 

Finished Pre Mapping Phase.
@N:BN225 :  | Writing default property annotation file C:\Users\ITP\Desktop\IGLOO\IGLOO_RHA\synthesis\DATA_ACQUISITION_BLOCK.sap. 
Pre-mapping successful!

At Mapper Exit (Real Time elapsed 0h:00m:00s; CPU Time elapsed 0h:00m:00s; Memory used current: 48MB peak: 111MB)

Process took 0h:00m:01s realtime, 0h:00m:01s cputime
# Fri Aug 01 16:09:15 2014

###########################################################]
Map & Optimize Report

Synopsys Microsemi Technology Mapper, Version mapact, Build 1154R, Built Jan 20 2014 10:14:08
Copyright (C) 1994-2013, Synopsys, Inc. This software and the associated documentation are proprietary to Synopsys, Inc. This software may only be used in accordance with the terms and conditions of a written license agreement with Synopsys, Inc.  All other use or distribution of the software is strictly prohibited.
Product Version I-2013.09M-SP1 

Mapper Startup Complete (Real Time elapsed 0h:00m:00s; CPU Time elapsed 0h:00m:00s; Memory used current: 99MB peak: 99MB)

@N:MF248 :  | Running in 64-bit mode. 
@N:MF667 :  | Clock conversion disabled  

Design Input Complete (Real Time elapsed 0h:00m:00s; CPU Time elapsed 0h:00m:00s; Memory used current: 99MB peak: 101MB)


Mapper Initialization Complete (Real Time elapsed 0h:00m:00s; CPU Time elapsed 0h:00m:00s; Memory used current: 99MB peak: 101MB)


Starting Optimization and Mapping (Real Time elapsed 0h:00m:00s; CPU Time elapsed 0h:00m:00s; Memory used current: 109MB peak: 110MB)


Available hyper_sources - for debug and ip models
	None Found


Finished RTL optimizations (Real Time elapsed 0h:00m:00s; CPU Time elapsed 0h:00m:00s; Memory used current: 110MB peak: 112MB)

@W:MO160 : rha_array.vhd(151) | Register bit AddressCheckInfo[33] is always 0, optimizing ...
@W:MO160 : rha_array.vhd(151) | Register bit AddressCheckInfo[38] is always 0, optimizing ...
@N:MF238 : rha_data_collector.vhd(122) | Found 5-bit incrementor, 'un7_counter_1[4:0]'
@N: : rha_to_zl_converter.vhd(295) | Found counter in view:work.RHA_TO_ZL_CONVERTER(behavioral) inst Timestamp[15:0]
@N: : rha_testvalue_generator.vhd(125) | Found counter in view:work.RHA_TESTVALUE_GENERATOR(behavioral) inst Sample_Counter[8:0]
@N: : rha_testvalue_generator.vhd(125) | Found counter in view:work.RHA_TESTVALUE_GENERATOR(behavioral) inst Channel_Counter[3:0]
@N:MO106 : rha_testvalue_generator.vhd(155) | Found ROM, 'DATAOUT_ADDRESS[3]', 16 words by 1 bits 
@N:MO106 : rha_test_modulator.vhd(160) | Found ROM, 'SYNC_PROCESS\.SPIMACHINE_STATE_26[4:0]', 26 words by 5 bits 
@W:MO160 : rha_test_modulator.vhd(79) | Register bit TESTVALUE_INTERNAL[23] is always 0, optimizing ...
@W:MO160 : rha_test_modulator.vhd(79) | Register bit TESTVALUE_INTERNAL[22] is always 0, optimizing ...
@W:MO160 : rha_test_modulator.vhd(79) | Register bit TESTVALUE_INTERNAL[21] is always 0, optimizing ...
@W:MO160 : rha_test_modulator.vhd(79) | Register bit TESTVALUE_INTERNAL[2] is always 0, optimizing ...
@N:MO106 : rha_test_modulator.vhd(160) | Found ROM, 'SYNC_PROCESS\.SPIMACHINE_STATE_26[4:0]', 26 words by 5 bits 
@W:MO160 : rha_test_modulator.vhd(79) | Register bit TESTVALUE_INTERNAL[23] is always 0, optimizing ...
@W:MO160 : rha_test_modulator.vhd(79) | Register bit TESTVALUE_INTERNAL[22] is always 0, optimizing ...
@W:MO160 : rha_test_modulator.vhd(79) | Register bit TESTVALUE_INTERNAL[2] is always 0, optimizing ...
@N:MO106 : rha_test_modulator.vhd(160) | Found ROM, 'SYNC_PROCESS\.SPIMACHINE_STATE_26[4:0]', 26 words by 5 bits 
@W:MO160 : rha_test_modulator.vhd(79) | Register bit TESTVALUE_INTERNAL[23] is always 0, optimizing ...
@W:MO160 : rha_test_modulator.vhd(79) | Register bit TESTVALUE_INTERNAL[21] is always 0, optimizing ...
@W:MO160 : rha_test_modulator.vhd(79) | Register bit TESTVALUE_INTERNAL[2] is always 0, optimizing ...
@N:MO106 : rha_test_modulator.vhd(160) | Found ROM, 'SYNC_PROCESS\.SPIMACHINE_STATE_26[4:0]', 26 words by 5 bits 
@W:MO160 : rha_test_modulator.vhd(79) | Register bit TESTVALUE_INTERNAL[23] is always 0, optimizing ...
@W:MO160 : rha_test_modulator.vhd(79) | Register bit TESTVALUE_INTERNAL[2] is always 0, optimizing ...
@N:MO106 : rha_test_modulator.vhd(160) | Found ROM, 'SYNC_PROCESS\.SPIMACHINE_STATE_26[4:0]', 26 words by 5 bits 
@W:MO160 : rha_test_modulator.vhd(79) | Register bit TESTVALUE_INTERNAL[22] is always 0, optimizing ...
@W:MO160 : rha_test_modulator.vhd(79) | Register bit TESTVALUE_INTERNAL[21] is always 0, optimizing ...
@W:MO160 : rha_test_modulator.vhd(79) | Register bit TESTVALUE_INTERNAL[2] is always 0, optimizing ...
@N:MO106 : rha_test_modulator.vhd(160) | Found ROM, 'SYNC_PROCESS\.SPIMACHINE_STATE_26[4:0]', 26 words by 5 bits 
@W:MO160 : rha_test_modulator.vhd(79) | Register bit TESTVALUE_INTERNAL[22] is always 0, optimizing ...
@W:MO160 : rha_test_modulator.vhd(79) | Register bit TESTVALUE_INTERNAL[2] is always 0, optimizing ...
@N:MO106 : rha_test_modulator.vhd(160) | Found ROM, 'SYNC_PROCESS\.SPIMACHINE_STATE_26[4:0]', 26 words by 5 bits 
@W:MO160 : rha_test_modulator.vhd(79) | Register bit TESTVALUE_INTERNAL[21] is always 0, optimizing ...
@W:MO160 : rha_test_modulator.vhd(79) | Register bit TESTVALUE_INTERNAL[2] is always 0, optimizing ...
@N:MO106 : rha_test_modulator.vhd(160) | Found ROM, 'SYNC_PROCESS\.SPIMACHINE_STATE_26[4:0]', 26 words by 5 bits 
@W:MO160 : rha_test_modulator.vhd(79) | Register bit TESTVALUE_INTERNAL[2] is always 0, optimizing ...
Auto Dissolve of MyRHA_TESTVALUE_GENERATOR (inst of view:work.RHA_TESTVALUE_GENERATOR(behavioral))

Finished factoring (Real Time elapsed 0h:00m:01s; CPU Time elapsed 0h:00m:01s; Memory used current: 125MB peak: 125MB)

@W:BN132 : data_acquisition_block.vhd(82) | Removing sequential instance RHA_ADC_clk[7],  because it is equivalent to instance RHA_ADC_clk[6]
@W:BN132 : data_acquisition_block.vhd(82) | Removing sequential instance RHA_ADC_clk[6],  because it is equivalent to instance RHA_ADC_clk[5]
@W:BN132 : data_acquisition_block.vhd(82) | Removing sequential instance RHA_ADC_clk[5],  because it is equivalent to instance RHA_ADC_clk[4]
@W:BN132 : data_acquisition_block.vhd(82) | Removing sequential instance RHA_ADC_clk[4],  because it is equivalent to instance RHA_ADC_clk[3]
@W:BN132 : data_acquisition_block.vhd(82) | Removing sequential instance RHA_ADC_clk[3],  because it is equivalent to instance RHA_ADC_clk[2]
@W:BN132 : data_acquisition_block.vhd(82) | Removing sequential instance RHA_ADC_clk[2],  because it is equivalent to instance RHA_ADC_clk[1]
@W:BN132 : data_acquisition_block.vhd(82) | Removing sequential instance RHA_ADC_clk[1],  because it is equivalent to instance RHA_ADC_clk[0]

Finished gated-clock and generated-clock conversion (Real Time elapsed 0h:00m:01s; CPU Time elapsed 0h:00m:01s; Memory used current: 123MB peak: 126MB)


Finished generic timing optimizations - Pass 1 (Real Time elapsed 0h:00m:02s; CPU Time elapsed 0h:00m:01s; Memory used current: 123MB peak: 126MB)


Starting Early Timing Optimization (Real Time elapsed 0h:00m:02s; CPU Time elapsed 0h:00m:01s; Memory used current: 124MB peak: 126MB)


Finished Early Timing Optimization (Real Time elapsed 0h:00m:02s; CPU Time elapsed 0h:00m:02s; Memory used current: 124MB peak: 126MB)


Finished generic timing optimizations - Pass 2 (Real Time elapsed 0h:00m:02s; CPU Time elapsed 0h:00m:02s; Memory used current: 123MB peak: 126MB)


Finished preparing to map (Real Time elapsed 0h:00m:02s; CPU Time elapsed 0h:00m:02s; Memory used current: 123MB peak: 126MB)


Finished technology mapping (Real Time elapsed 0h:00m:02s; CPU Time elapsed 0h:00m:02s; Memory used current: 122MB peak: 126MB)


High Fanout Net Report
**********************

Driver Instance / Pin Name                                            Fanout, notes                     
--------------------------------------------------------------------------------------------------------
Inst_RHA_ARRAY.DATAOUT_VALID_OUT / Q                                  28                                
Inst_RHA_TO_ZL_CONVERTER.SPIMACHINE_STATE[0] / Q                      102                               
Inst_RHA_TO_ZL_CONVERTER.SPIMACHINE_STATE[1] / Q                      27                                
Inst_RHA_TO_ZL_CONVERTER.SPIMACHINE_STATE[2] / Q                      61                                
Inst_RHA_TO_ZL_CONVERTER.SPIMACHINE_STATE[3] / Q                      31                                
Inst_RHA_TO_ZL_CONVERTER.SPIMACHINE_STATE[4] / Q                      68                                
Reset_pad / Y                                                         1090 : 1044 asynchronous set/reset
Inst_RHA_TO_ZL_CONVERTER.un1_NewBlock_Off_1_sqmuxa / Y                48                                
Inst_RHA_ARRAY.COPY_VALID_DATA.un52_gated_valid / Y                   198                               
Inst_RHA_ARRAY.COPY_VALID_DATA.un48_gated_valid / Y                   401                               
Inst_RHA_ARRAY.COPY_VALID_DATA.un1_readrequest / Y                    209                               
Inst_RHA_TESTMODULE.RHA_TEST_MOD_7.ADC_OUT_PROCESS.adc_out127 / Y     25                                
========================================================================================================

@N:FP130 :  | Promoting Net Reset_c on CLKBUF  Reset_pad  
@N:FP130 :  | Promoting Net Clk_c on CLKBUF  Clk_pad  
@N:FP130 :  | Promoting Net Clk_Slow_c on CLKBUF  Clk_Slow_pad  
@N:FP130 :  | Promoting Net Inst_RHA_ARRAY.COPY_VALID_DATA\.un48_gated_valid on CLKINT  I_49  
@N:FP130 :  | Promoting Net RHA_ADC_clk_TEST on CLKINT  RHA_ADC_clk_TEST_inferred_clock  
@N:FP130 :  | Promoting Net Inst_RHA_ARRAY.COPY_VALID_DATA\.un1_readrequest on CLKINT  I_50  

Finished technology timing optimizations and critical path resynthesis (Real Time elapsed 0h:00m:03s; CPU Time elapsed 0h:00m:02s; Memory used current: 122MB peak: 126MB)

Replicating Combinational Instance Inst_RHA_TESTMODULE.RHA_TEST_MOD_7.ADC_OUT_PROCESS.adc_out127, fanout 25 segments 2
Replicating Combinational Instance Inst_RHA_ARRAY.COPY_VALID_DATA.un52_gated_valid, fanout 198 segments 9
Replicating Combinational Instance Inst_RHA_TO_ZL_CONVERTER.un1_NewBlock_Off_1_sqmuxa, fanout 48 segments 2
Replicating Sequential Instance Inst_RHA_TO_ZL_CONVERTER.SPIMACHINE_STATE[4], fanout 68 segments 3
Replicating Sequential Instance Inst_RHA_TO_ZL_CONVERTER.SPIMACHINE_STATE[3], fanout 31 segments 2
Replicating Sequential Instance Inst_RHA_TO_ZL_CONVERTER.SPIMACHINE_STATE[2], fanout 61 segments 3
Replicating Sequential Instance Inst_RHA_TO_ZL_CONVERTER.SPIMACHINE_STATE[1], fanout 27 segments 2
Replicating Sequential Instance Inst_RHA_TO_ZL_CONVERTER.SPIMACHINE_STATE[0], fanout 102 segments 5
Replicating Sequential Instance Inst_RHA_ARRAY.DATAOUT_VALID_OUT, fanout 28 segments 2

Added 0 Buffers
Added 21 Cells via replication
	Added 11 Sequential Cells via replication
	Added 10 Combinational Cells via replication

Finished restoring hierarchy (Real Time elapsed 0h:00m:03s; CPU Time elapsed 0h:00m:02s; Memory used current: 123MB peak: 126MB)


#### START OF CLOCK OPTIMIZATION REPORT #####[

Clock optimization not enabled
3 non-gated/non-generated clock tree(s) driving 844 clock pin(s) of sequential element(s)
1 gated/generated clock tree(s) driving 259 clock pin(s) of sequential element(s)
0 instances converted, 259 sequential instances remain driven by gated/generated clocks

========================================== Non-Gated/Non-Generated Clocks ===========================================
Clock Tree ID     Driving Element     Drive Element Type     Fanout     Sample Instance                              
---------------------------------------------------------------------------------------------------------------------
ClockId0002        Clk                 port                   570        RHA_ERROR_WATCHDOG.ERROR_ADDRESS_OUT_INTERNAL
ClockId0003        Clk_Slow            port                   272        Inst_RHA_ARRAY.RHA7.Counter[4]               
ClockId0004        TestMode            port                   2          RHA_ADC_clk[0]                               
=====================================================================================================================
========================================================================================== Gated/Generated Clocks ==========================================================================================
Clock Tree ID     Driving Element      Drive Element Type     Fanout     Sample Instance                                            Explanation                                                             
------------------------------------------------------------------------------------------------------------------------------------------------------------------------------------------------------------
ClockId0001        RHA_ADC_clk_TEST     DLN1                   259        Inst_RHA_TESTMODULE.RHA_TEST_MOD_7.SPIMACHINE_STATE[4]     No generated or derived clock directive on output of sequential instance
============================================================================================================================================================================================================


##### END OF CLOCK OPTIMIZATION REPORT ######]

Writing Analyst data base C:\Users\ITP\Desktop\IGLOO\IGLOO_RHA\synthesis\DATA_ACQUISITION_BLOCK.srm

Finished Writing Netlist Databases (Real Time elapsed 0h:00m:03s; CPU Time elapsed 0h:00m:03s; Memory used current: 121MB peak: 126MB)

Writing EDIF Netlist and constraint files
I-2013.09M-SP1 

Finished Writing EDIF Netlist and constraint files (Real Time elapsed 0h:00m:04s; CPU Time elapsed 0h:00m:03s; Memory used current: 122MB peak: 126MB)

@W:MT420 :  | Found inferred clock DATA_ACQUISITION_BLOCK|TestMode with period 10.00ns. Please declare a user-defined clock on object "p:TestMode" 

@W:MT420 :  | Found inferred clock DATA_ACQUISITION_BLOCK|Clk_Slow with period 10.00ns. Please declare a user-defined clock on object "p:Clk_Slow" 

@W:MT420 :  | Found inferred clock DATA_ACQUISITION_BLOCK|Clk with period 10.00ns. Please declare a user-defined clock on object "p:Clk" 

@W:MT420 :  | Found inferred clock DATA_ACQUISITION_BLOCK|RHA_ADC_clk_TEST_inferred_clock with period 10.00ns. Please declare a user-defined clock on object "n:RHA_ADC_clk_TEST" 


##### START OF TIMING REPORT #####[
# Timing Report written on Fri Aug 01 16:09:20 2014
#


Top view:               DATA_ACQUISITION_BLOCK
Library name:           IGLOO_V2
Operating conditions:   COMWCSTD ( T = 70.0, V = 1.14, P = 3.70, tree_type = balanced_tree )
Requested Frequency:    100.0 MHz
Wire load mode:         top
Wire load model:        igloo
Paths requested:        5
Constraint File(s):    
@N:MT320 :  | Timing report estimates place and route data. Please look at the place and route timing report for final timing. 

@N:MT322 :  | Clock constraints cover only FF-to-FF paths associated with the clock. 


Performance Summary 
*******************


Worst slack in design: -31.462

                                                           Requested     Estimated     Requested     Estimated                 Clock        Clock              
Starting Clock                                             Frequency     Frequency     Period        Period        Slack       Type         Group              
---------------------------------------------------------------------------------------------------------------------------------------------------------------
DATA_ACQUISITION_BLOCK|Clk                                 100.0 MHz     24.1 MHz      10.000        41.462        -31.462     inferred     Inferred_clkgroup_1
DATA_ACQUISITION_BLOCK|Clk_Slow                            100.0 MHz     32.3 MHz      10.000        30.980        -10.490     inferred     Inferred_clkgroup_3
DATA_ACQUISITION_BLOCK|RHA_ADC_clk_TEST_inferred_clock     100.0 MHz     20.2 MHz      10.000        49.565        -19.782     inferred     Inferred_clkgroup_2
DATA_ACQUISITION_BLOCK|TestMode                            100.0 MHz     NA            10.000        NA            NA          inferred     Inferred_clkgroup_0
===============================================================================================================================================================


Clock Relationships
*******************

Clocks                                                                                                          |    rise  to  rise     |    fall  to  fall     |    rise  to  fall     |    fall  to  rise   
--------------------------------------------------------------------------------------------------------------------------------------------------------------------------------------------------------------
Starting                                                Ending                                                  |  constraint  slack    |  constraint  slack    |  constraint  slack    |  constraint  slack  
--------------------------------------------------------------------------------------------------------------------------------------------------------------------------------------------------------------
DATA_ACQUISITION_BLOCK|Clk                              DATA_ACQUISITION_BLOCK|Clk                              |  10.000      -31.462  |  No paths    -        |  No paths    -        |  No paths    -      
DATA_ACQUISITION_BLOCK|RHA_ADC_clk_TEST_inferred_clock  DATA_ACQUISITION_BLOCK|RHA_ADC_clk_TEST_inferred_clock  |  10.000      -14.544  |  10.000      -12.544  |  5.000       -3.849   |  5.000       -19.782
DATA_ACQUISITION_BLOCK|RHA_ADC_clk_TEST_inferred_clock  DATA_ACQUISITION_BLOCK|Clk_Slow                         |  No paths    -        |  No paths    -        |  Diff grp    -        |  Diff grp    -      
DATA_ACQUISITION_BLOCK|Clk_Slow                         DATA_ACQUISITION_BLOCK|Clk                              |  No paths    -        |  No paths    -        |  No paths    -        |  Diff grp    -      
DATA_ACQUISITION_BLOCK|Clk_Slow                         DATA_ACQUISITION_BLOCK|Clk_Slow                         |  10.000      -5.193   |  10.000      5.790    |  5.000       -10.490  |  No paths    -      
==============================================================================================================================================================================================================
 Note: 'No paths' indicates there are no paths in the design for that pair of clock edges.
       'Diff grp' indicates that paths exist but the starting clock and ending clock are in different clock groups.


Interface Information 
*********************

No IO constraint found


====================================
Detailed Report for Clock: DATA_ACQUISITION_BLOCK|Clk
====================================


Starting Points with Worst Slack
********************************

                                                       Starting                                                                        Arrival            
Instance                                               Reference                      Type       Pin     Net                           Time        Slack  
                                                       Clock                                                                                              
----------------------------------------------------------------------------------------------------------------------------------------------------------
Inst_RHA_TO_ZL_CONVERTER.SPIMACHINE_STATE[4]           DATA_ACQUISITION_BLOCK|Clk     DFN1E0     Q       SPIMACHINE_STATE[4]           1.771       -31.462
Inst_RHA_TO_ZL_CONVERTER.SPIMACHINE_STATE_1[2]         DATA_ACQUISITION_BLOCK|Clk     DFN1E0     Q       SPIMACHINE_STATE_1[2]         1.771       -30.451
Inst_RHA_TO_ZL_CONVERTER.SPIMACHINE_STATE[0]           DATA_ACQUISITION_BLOCK|Clk     DFN1E0     Q       SPIMACHINE_STATE[0]           1.395       -30.155
Inst_RHA_TO_ZL_CONVERTER.SPIMACHINE_STATE_0[1]         DATA_ACQUISITION_BLOCK|Clk     DFN1E0     Q       SPIMACHINE_STATE_0[1]         1.771       -29.817
Inst_RHA_TO_ZL_CONVERTER.SPIMACHINE_STATE_0[3]         DATA_ACQUISITION_BLOCK|Clk     DFN1E0     Q       SPIMACHINE_STATE_0[3]         1.395       -28.981
Inst_RHA_TO_ZL_CONVERTER.COUNTER_BITS_OF_A_BYTE[0]     DATA_ACQUISITION_BLOCK|Clk     DFN1C1     Q       COUNTER_BITS_OF_A_BYTE[0]     1.771       -28.814
Inst_RHA_TO_ZL_CONVERTER.COUNTER_BITS_OF_A_BYTE[2]     DATA_ACQUISITION_BLOCK|Clk     DFN1C1     Q       COUNTER_BITS_OF_A_BYTE[2]     1.771       -28.706
Inst_RHA_TO_ZL_CONVERTER.COUNTER_BITS_OF_A_BYTE[1]     DATA_ACQUISITION_BLOCK|Clk     DFN1C1     Q       COUNTER_BITS_OF_A_BYTE[1]     1.771       -28.371
Inst_RHA_TO_ZL_CONVERTER.COUNTER_BITS_OF_A_BYTE[3]     DATA_ACQUISITION_BLOCK|Clk     DFN1C1     Q       COUNTER_BITS_OF_A_BYTE[3]     1.771       -27.812
Inst_RHA_TO_ZL_CONVERTER.COUNTER_BITS_PROCESSED[4]     DATA_ACQUISITION_BLOCK|Clk     DFN1C1     Q       COUNTER_BITS_PROCESSED[4]     1.771       -26.847
==========================================================================================================================================================


Ending Points with Worst Slack
******************************

                                                       Starting                                                                             Required            
Instance                                               Reference                      Type         Pin     Net                              Time         Slack  
                                                       Clock                                                                                                    
----------------------------------------------------------------------------------------------------------------------------------------------------------------
Inst_RHA_TO_ZL_CONVERTER.COUNTER_BITS_PROCESSED[6]     DATA_ACQUISITION_BLOCK|Clk     DFN1C1       D       COUNTER_BITS_PROCESSED_9[6]      8.705        -31.462
Inst_RHA_TO_ZL_CONVERTER.COUNTER_BITS_PROCESSED[5]     DATA_ACQUISITION_BLOCK|Clk     DFN1C1       D       COUNTER_BITS_PROCESSED_9[5]      8.705        -31.228
Inst_RHA_TO_ZL_CONVERTER.COUNTER_BITS_PROCESSED[4]     DATA_ACQUISITION_BLOCK|Clk     DFN1C1       D       COUNTER_BITS_PROCESSED_9[4]      8.705        -29.453
Inst_RHA_TO_ZL_CONVERTER.COUNTER_BITS_PROCESSED[3]     DATA_ACQUISITION_BLOCK|Clk     DFN1C1       D       COUNTER_BITS_PROCESSED_9[3]      8.705        -28.288
Inst_RHA_TO_ZL_CONVERTER.COUNTER_BITS_OF_A_BYTE[3]     DATA_ACQUISITION_BLOCK|Clk     DFN1C1       D       COUNTER_BITS_OF_A_BYTE_10[3]     8.705        -27.152
Inst_RHA_TO_ZL_CONVERTER.COUNTER_BITS_PROCESSED[2]     DATA_ACQUISITION_BLOCK|Clk     DFN1C1       D       COUNTER_BITS_PROCESSED_9[2]      8.705        -26.279
Inst_RHA_TO_ZL_CONVERTER.COUNTER_BITS_OF_A_BYTE[2]     DATA_ACQUISITION_BLOCK|Clk     DFN1C1       D       COUNTER_BITS_OF_A_BYTE_10[2]     8.705        -25.377
Inst_RHA_TO_ZL_CONVERTER.FIFO_OUT_DATA_INTERNAL[2]     DATA_ACQUISITION_BLOCK|Clk     DFN1E0C1     D       FIFO_OUT_DATA_INTERNAL_33[2]     8.789        -23.995
Inst_RHA_TO_ZL_CONVERTER.FIFO_OUT_DATA_INTERNAL[3]     DATA_ACQUISITION_BLOCK|Clk     DFN1E0C1     D       FIFO_OUT_DATA_INTERNAL_33[3]     8.789        -23.995
Inst_RHA_TO_ZL_CONVERTER.FIFO_OUT_DATA_INTERNAL[4]     DATA_ACQUISITION_BLOCK|Clk     DFN1E0C1     D       FIFO_OUT_DATA_INTERNAL_33[4]     8.789        -23.995
================================================================================================================================================================


Worst Path Information
View Worst Path in Analyst
***********************


Path information for path number 1: 
      Requested Period:                      10.000
    - Setup time:                            1.295
    + Clock delay at ending point:           0.000 (ideal)
    = Required time:                         8.705

    - Propagation time:                      40.167
    - Clock delay at starting point:         0.000 (ideal)
    = Slack (critical) :                     -31.462

    Number of logic level(s):                11
    Starting point:                          Inst_RHA_TO_ZL_CONVERTER.SPIMACHINE_STATE[4] / Q
    Ending point:                            Inst_RHA_TO_ZL_CONVERTER.COUNTER_BITS_PROCESSED[6] / D
    The start point is clocked by            DATA_ACQUISITION_BLOCK|Clk [rising] on pin CLK
    The end   point is clocked by            DATA_ACQUISITION_BLOCK|Clk [rising] on pin CLK

Instance / Net                                                            Pin      Pin               Arrival     No. of    
Name                                                           Type       Name     Dir     Delay     Time        Fan Out(s)
---------------------------------------------------------------------------------------------------------------------------
Inst_RHA_TO_ZL_CONVERTER.SPIMACHINE_STATE[4]                   DFN1E0     Q        Out     1.771     1.771       -         
SPIMACHINE_STATE[4]                                            Net        -        -       5.788     -           22        
Inst_RHA_TO_ZL_CONVERTER.SPIMACHINE_STATE_RNIUQOD_0[0]         NOR2A      A        In      -         7.559       -         
Inst_RHA_TO_ZL_CONVERTER.SPIMACHINE_STATE_RNIUQOD_0[0]         NOR2A      Y        Out     1.508     9.067       -         
un1_spimachine_state72_4_a3_2                                  Net        -        -       1.938     -           3         
Inst_RHA_TO_ZL_CONVERTER.SPIMACHINE_STATE_0_RNIPL4I1_0[1]      NOR2B      A        In      -         11.005      -         
Inst_RHA_TO_ZL_CONVERTER.SPIMACHINE_STATE_0_RNIPL4I1_0[1]      NOR2B      Y        Out     1.236     12.241      -         
spimachine_state76                                             Net        -        -       4.009     -           9         
Inst_RHA_TO_ZL_CONVERTER.SPIMACHINE_STATE_0_RNIIB943[1]        NOR2       B        In      -         16.250      -         
Inst_RHA_TO_ZL_CONVERTER.SPIMACHINE_STATE_0_RNIIB943[1]        NOR2       Y        Out     1.554     17.804      -         
un1_spimachine_state72_0_0_0                                   Net        -        -       0.773     -           1         
Inst_RHA_TO_ZL_CONVERTER.SPIMACHINE_STATE_RNI4EVAC[0]          NOR3A      A        In      -         18.576      -         
Inst_RHA_TO_ZL_CONVERTER.SPIMACHINE_STATE_RNI4EVAC[0]          NOR3A      Y        Out     1.595     20.172      -         
un1_spimachine_state72_0_2                                     Net        -        -       0.773     -           1         
Inst_RHA_TO_ZL_CONVERTER.BITMASK_SAMPLE_COPY_RNI2MGBG[0]       OR2A       A        In      -         20.944      -         
Inst_RHA_TO_ZL_CONVERTER.BITMASK_SAMPLE_COPY_RNI2MGBG[0]       OR2A       Y        Out     1.290     22.235      -         
BITMASK_SAMPLE_COPY_RNI2MGBG[0]                                Net        -        -       1.938     -           3         
Inst_RHA_TO_ZL_CONVERTER.un1_COUNTER_BITS_PROCESSED_1.I_1      AND2       B        In      -         24.172      -         
Inst_RHA_TO_ZL_CONVERTER.un1_COUNTER_BITS_PROCESSED_1.I_1      AND2       Y        Out     1.508     25.680      -         
DWACT_ADD_CI_0_TMP[0]                                          Net        -        -       0.927     -           2         
Inst_RHA_TO_ZL_CONVERTER.un1_COUNTER_BITS_PROCESSED_1.I_32     NOR2B      A        In      -         26.607      -         
Inst_RHA_TO_ZL_CONVERTER.un1_COUNTER_BITS_PROCESSED_1.I_32     NOR2B      Y        Out     1.236     27.843      -         
DWACT_ADD_CI_0_g_array_1[0]                                    Net        -        -       1.938     -           3         
Inst_RHA_TO_ZL_CONVERTER.un1_COUNTER_BITS_PROCESSED_1.I_38     NOR2B      A        In      -         29.781      -         
Inst_RHA_TO_ZL_CONVERTER.un1_COUNTER_BITS_PROCESSED_1.I_38     NOR2B      Y        Out     1.236     31.017      -         
DWACT_ADD_CI_0_g_array_2[0]                                    Net        -        -       1.938     -           3         
Inst_RHA_TO_ZL_CONVERTER.un1_COUNTER_BITS_PROCESSED_1.I_35     NOR2B      A        In      -         32.955      -         
Inst_RHA_TO_ZL_CONVERTER.un1_COUNTER_BITS_PROCESSED_1.I_35     NOR2B      Y        Out     1.236     34.191      -         
DWACT_ADD_CI_0_g_array_11[0]                                   Net        -        -       0.773     -           1         
Inst_RHA_TO_ZL_CONVERTER.un1_COUNTER_BITS_PROCESSED_1.I_28     XOR2       B        In      -         34.964      -         
Inst_RHA_TO_ZL_CONVERTER.un1_COUNTER_BITS_PROCESSED_1.I_28     XOR2       Y        Out     2.251     37.215      -         
I_28                                                           Net        -        -       0.773     -           1         
Inst_RHA_TO_ZL_CONVERTER.COUNTER_BITS_PROCESSED_RNO[6]         MX2        B        In      -         37.987      -         
Inst_RHA_TO_ZL_CONVERTER.COUNTER_BITS_PROCESSED_RNO[6]         MX2        Y        Out     1.407     39.395      -         
COUNTER_BITS_PROCESSED_9[6]                                    Net        -        -       0.773     -           1         
Inst_RHA_TO_ZL_CONVERTER.COUNTER_BITS_PROCESSED[6]             DFN1C1     D        In      -         40.167      -         
===========================================================================================================================
Total path delay (propagation time + setup) of 41.462 is 19.123(46.1%) logic and 22.339(53.9%) route.
Path delay compensated for clock skew. Clock skew is added to clock-to-out value, and is subtracted from setup time value


Path information for path number 2: 
      Requested Period:                      10.000
    - Setup time:                            1.295
    + Clock delay at ending point:           0.000 (ideal)
    = Required time:                         8.705

    - Propagation time:                      39.934
    - Clock delay at starting point:         0.000 (ideal)
    = Slack (non-critical) :                 -31.228

    Number of logic level(s):                11
    Starting point:                          Inst_RHA_TO_ZL_CONVERTER.SPIMACHINE_STATE[4] / Q
    Ending point:                            Inst_RHA_TO_ZL_CONVERTER.COUNTER_BITS_PROCESSED[5] / D
    The start point is clocked by            DATA_ACQUISITION_BLOCK|Clk [rising] on pin CLK
    The end   point is clocked by            DATA_ACQUISITION_BLOCK|Clk [rising] on pin CLK

Instance / Net                                                            Pin      Pin               Arrival     No. of    
Name                                                           Type       Name     Dir     Delay     Time        Fan Out(s)
---------------------------------------------------------------------------------------------------------------------------
Inst_RHA_TO_ZL_CONVERTER.SPIMACHINE_STATE[4]                   DFN1E0     Q        Out     1.771     1.771       -         
SPIMACHINE_STATE[4]                                            Net        -        -       5.788     -           22        
Inst_RHA_TO_ZL_CONVERTER.SPIMACHINE_STATE_RNIUQOD_0[0]         NOR2A      A        In      -         7.559       -         
Inst_RHA_TO_ZL_CONVERTER.SPIMACHINE_STATE_RNIUQOD_0[0]         NOR2A      Y        Out     1.508     9.067       -         
un1_spimachine_state72_4_a3_2                                  Net        -        -       1.938     -           3         
Inst_RHA_TO_ZL_CONVERTER.SPIMACHINE_STATE_0_RNIPL4I1_0[1]      NOR2B      A        In      -         11.005      -         
Inst_RHA_TO_ZL_CONVERTER.SPIMACHINE_STATE_0_RNIPL4I1_0[1]      NOR2B      Y        Out     1.236     12.241      -         
spimachine_state76                                             Net        -        -       4.009     -           9         
Inst_RHA_TO_ZL_CONVERTER.SPIMACHINE_STATE_0_RNIIB943[1]        NOR2       B        In      -         16.250      -         
Inst_RHA_TO_ZL_CONVERTER.SPIMACHINE_STATE_0_RNIIB943[1]        NOR2       Y        Out     1.554     17.804      -         
un1_spimachine_state72_0_0_0                                   Net        -        -       0.773     -           1         
Inst_RHA_TO_ZL_CONVERTER.SPIMACHINE_STATE_RNI4EVAC[0]          NOR3A      A        In      -         18.576      -         
Inst_RHA_TO_ZL_CONVERTER.SPIMACHINE_STATE_RNI4EVAC[0]          NOR3A      Y        Out     1.595     20.172      -         
un1_spimachine_state72_0_2                                     Net        -        -       0.773     -           1         
Inst_RHA_TO_ZL_CONVERTER.BITMASK_SAMPLE_COPY_RNI2MGBG[0]       OR2A       A        In      -         20.944      -         
Inst_RHA_TO_ZL_CONVERTER.BITMASK_SAMPLE_COPY_RNI2MGBG[0]       OR2A       Y        Out     1.290     22.235      -         
BITMASK_SAMPLE_COPY_RNI2MGBG[0]                                Net        -        -       1.938     -           3         
Inst_RHA_TO_ZL_CONVERTER.un1_COUNTER_BITS_PROCESSED_1.I_1      AND2       B        In      -         24.172      -         
Inst_RHA_TO_ZL_CONVERTER.un1_COUNTER_BITS_PROCESSED_1.I_1      AND2       Y        Out     1.508     25.680      -         
DWACT_ADD_CI_0_TMP[0]                                          Net        -        -       0.927     -           2         
Inst_RHA_TO_ZL_CONVERTER.un1_COUNTER_BITS_PROCESSED_1.I_32     NOR2B      A        In      -         26.607      -         
Inst_RHA_TO_ZL_CONVERTER.un1_COUNTER_BITS_PROCESSED_1.I_32     NOR2B      Y        Out     1.236     27.843      -         
DWACT_ADD_CI_0_g_array_1[0]                                    Net        -        -       1.938     -           3         
Inst_RHA_TO_ZL_CONVERTER.un1_COUNTER_BITS_PROCESSED_1.I_38     NOR2B      A        In      -         29.781      -         
Inst_RHA_TO_ZL_CONVERTER.un1_COUNTER_BITS_PROCESSED_1.I_38     NOR2B      Y        Out     1.236     31.017      -         
DWACT_ADD_CI_0_g_array_2[0]                                    Net        -        -       1.938     -           3         
Inst_RHA_TO_ZL_CONVERTER.un1_COUNTER_BITS_PROCESSED_1.I_36     NOR2B      A        In      -         32.955      -         
Inst_RHA_TO_ZL_CONVERTER.un1_COUNTER_BITS_PROCESSED_1.I_36     NOR2B      Y        Out     1.236     34.191      -         
DWACT_ADD_CI_0_g_array_12_1[0]                                 Net        -        -       0.773     -           1         
Inst_RHA_TO_ZL_CONVERTER.un1_COUNTER_BITS_PROCESSED_1.I_29     XOR2       B        In      -         34.964      -         
Inst_RHA_TO_ZL_CONVERTER.un1_COUNTER_BITS_PROCESSED_1.I_29     XOR2       Y        Out     2.251     37.215      -         
I_29                                                           Net        -        -       0.773     -           1         
Inst_RHA_TO_ZL_CONVERTER.COUNTER_BITS_PROCESSED_RNO[5]         NOR2B      A        In      -         37.987      -         
Inst_RHA_TO_ZL_CONVERTER.COUNTER_BITS_PROCESSED_RNO[5]         NOR2B      Y        Out     1.174     39.161      -         
COUNTER_BITS_PROCESSED_9[5]                                    Net        -        -       0.773     -           1         
Inst_RHA_TO_ZL_CONVERTER.COUNTER_BITS_PROCESSED[5]             DFN1C1     D        In      -         39.934      -         
===========================================================================================================================
Total path delay (propagation time + setup) of 41.228 is 18.889(45.8%) logic and 22.339(54.2%) route.
Path delay compensated for clock skew. Clock skew is added to clock-to-out value, and is subtracted from setup time value


Path information for path number 3: 
      Requested Period:                      10.000
    - Setup time:                            1.295
    + Clock delay at ending point:           0.000 (ideal)
    = Required time:                         8.705

    - Propagation time:                      39.516
    - Clock delay at starting point:         0.000 (ideal)
    = Slack (non-critical) :                 -30.810

    Number of logic level(s):                11
    Starting point:                          Inst_RHA_TO_ZL_CONVERTER.SPIMACHINE_STATE[4] / Q
    Ending point:                            Inst_RHA_TO_ZL_CONVERTER.COUNTER_BITS_PROCESSED[6] / D
    The start point is clocked by            DATA_ACQUISITION_BLOCK|Clk [rising] on pin CLK
    The end   point is clocked by            DATA_ACQUISITION_BLOCK|Clk [rising] on pin CLK

Instance / Net                                                            Pin      Pin               Arrival     No. of    
Name                                                           Type       Name     Dir     Delay     Time        Fan Out(s)
---------------------------------------------------------------------------------------------------------------------------
Inst_RHA_TO_ZL_CONVERTER.SPIMACHINE_STATE[4]                   DFN1E0     Q        Out     1.771     1.771       -         
SPIMACHINE_STATE[4]                                            Net        -        -       5.788     -           22        
Inst_RHA_TO_ZL_CONVERTER.SPIMACHINE_STATE_RNIUQOD_0[0]         NOR2A      A        In      -         7.559       -         
Inst_RHA_TO_ZL_CONVERTER.SPIMACHINE_STATE_RNIUQOD_0[0]         NOR2A      Y        Out     1.508     9.067       -         
un1_spimachine_state72_4_a3_2                                  Net        -        -       1.938     -           3         
Inst_RHA_TO_ZL_CONVERTER.SPIMACHINE_STATE_0_RNIPL4I1[1]        NOR2B      B        In      -         11.005      -         
Inst_RHA_TO_ZL_CONVERTER.SPIMACHINE_STATE_0_RNIPL4I1[1]        NOR2B      Y        Out     1.508     12.512      -         
spimachine_state72                                             Net        -        -       3.420     -           6         
Inst_RHA_TO_ZL_CONVERTER.SPIMACHINE_STATE_0_RNIIB943[1]        NOR2       A        In      -         15.932      -         
Inst_RHA_TO_ZL_CONVERTER.SPIMACHINE_STATE_0_RNIIB943[1]        NOR2       Y        Out     1.219     17.152      -         
un1_spimachine_state72_0_0_0                                   Net        -        -       0.773     -           1         
Inst_RHA_TO_ZL_CONVERTER.SPIMACHINE_STATE_RNI4EVAC[0]          NOR3A      A        In      -         17.924      -         
Inst_RHA_TO_ZL_CONVERTER.SPIMACHINE_STATE_RNI4EVAC[0]          NOR3A      Y        Out     1.595     19.520      -         
un1_spimachine_state72_0_2                                     Net        -        -       0.773     -           1         
Inst_RHA_TO_ZL_CONVERTER.BITMASK_SAMPLE_COPY_RNI2MGBG[0]       OR2A       A        In      -         20.292      -         
Inst_RHA_TO_ZL_CONVERTER.BITMASK_SAMPLE_COPY_RNI2MGBG[0]       OR2A       Y        Out     1.290     21.583      -         
BITMASK_SAMPLE_COPY_RNI2MGBG[0]                                Net        -        -       1.938     -           3         
Inst_RHA_TO_ZL_CONVERTER.un1_COUNTER_BITS_PROCESSED_1.I_1      AND2       B        In      -         23.521      -         
Inst_RHA_TO_ZL_CONVERTER.un1_COUNTER_BITS_PROCESSED_1.I_1      AND2       Y        Out     1.508     25.028      -         
DWACT_ADD_CI_0_TMP[0]                                          Net        -        -       0.927     -           2         
Inst_RHA_TO_ZL_CONVERTER.un1_COUNTER_BITS_PROCESSED_1.I_32     NOR2B      A        In      -         25.955      -         
Inst_RHA_TO_ZL_CONVERTER.un1_COUNTER_BITS_PROCESSED_1.I_32     NOR2B      Y        Out     1.236     27.192      -         
DWACT_ADD_CI_0_g_array_1[0]                                    Net        -        -       1.938     -           3         
Inst_RHA_TO_ZL_CONVERTER.un1_COUNTER_BITS_PROCESSED_1.I_38     NOR2B      A        In      -         29.129      -         
Inst_RHA_TO_ZL_CONVERTER.un1_COUNTER_BITS_PROCESSED_1.I_38     NOR2B      Y        Out     1.236     30.366      -         
DWACT_ADD_CI_0_g_array_2[0]                                    Net        -        -       1.938     -           3         
Inst_RHA_TO_ZL_CONVERTER.un1_COUNTER_BITS_PROCESSED_1.I_35     NOR2B      A        In      -         32.303      -         
Inst_RHA_TO_ZL_CONVERTER.un1_COUNTER_BITS_PROCESSED_1.I_35     NOR2B      Y        Out     1.236     33.540      -         
DWACT_ADD_CI_0_g_array_11[0]                                   Net        -        -       0.773     -           1         
Inst_RHA_TO_ZL_CONVERTER.un1_COUNTER_BITS_PROCESSED_1.I_28     XOR2       B        In      -         34.312      -         
Inst_RHA_TO_ZL_CONVERTER.un1_COUNTER_BITS_PROCESSED_1.I_28     XOR2       Y        Out     2.251     36.563      -         
I_28                                                           Net        -        -       0.773     -           1         
Inst_RHA_TO_ZL_CONVERTER.COUNTER_BITS_PROCESSED_RNO[6]         MX2        B        In      -         37.336      -         
Inst_RHA_TO_ZL_CONVERTER.COUNTER_BITS_PROCESSED_RNO[6]         MX2        Y        Out     1.407     38.743      -         
COUNTER_BITS_PROCESSED_9[6]                                    Net        -        -       0.773     -           1         
Inst_RHA_TO_ZL_CONVERTER.COUNTER_BITS_PROCESSED[6]             DFN1C1     D        In      -         39.516      -         
===========================================================================================================================
Total path delay (propagation time + setup) of 40.810 is 19.060(46.7%) logic and 21.750(53.3%) route.
Path delay compensated for clock skew. Clock skew is added to clock-to-out value, and is subtracted from setup time value


Path information for path number 4: 
      Requested Period:                      10.000
    - Setup time:                            1.295
    + Clock delay at ending point:           0.000 (ideal)
    = Required time:                         8.705

    - Propagation time:                      39.282
    - Clock delay at starting point:         0.000 (ideal)
    = Slack (non-critical) :                 -30.577

    Number of logic level(s):                11
    Starting point:                          Inst_RHA_TO_ZL_CONVERTER.SPIMACHINE_STATE[4] / Q
    Ending point:                            Inst_RHA_TO_ZL_CONVERTER.COUNTER_BITS_PROCESSED[5] / D
    The start point is clocked by            DATA_ACQUISITION_BLOCK|Clk [rising] on pin CLK
    The end   point is clocked by            DATA_ACQUISITION_BLOCK|Clk [rising] on pin CLK

Instance / Net                                                            Pin      Pin               Arrival     No. of    
Name                                                           Type       Name     Dir     Delay     Time        Fan Out(s)
---------------------------------------------------------------------------------------------------------------------------
Inst_RHA_TO_ZL_CONVERTER.SPIMACHINE_STATE[4]                   DFN1E0     Q        Out     1.771     1.771       -         
SPIMACHINE_STATE[4]                                            Net        -        -       5.788     -           22        
Inst_RHA_TO_ZL_CONVERTER.SPIMACHINE_STATE_RNIUQOD_0[0]         NOR2A      A        In      -         7.559       -         
Inst_RHA_TO_ZL_CONVERTER.SPIMACHINE_STATE_RNIUQOD_0[0]         NOR2A      Y        Out     1.508     9.067       -         
un1_spimachine_state72_4_a3_2                                  Net        -        -       1.938     -           3         
Inst_RHA_TO_ZL_CONVERTER.SPIMACHINE_STATE_0_RNIPL4I1[1]        NOR2B      B        In      -         11.005      -         
Inst_RHA_TO_ZL_CONVERTER.SPIMACHINE_STATE_0_RNIPL4I1[1]        NOR2B      Y        Out     1.508     12.512      -         
spimachine_state72                                             Net        -        -       3.420     -           6         
Inst_RHA_TO_ZL_CONVERTER.SPIMACHINE_STATE_0_RNIIB943[1]        NOR2       A        In      -         15.932      -         
Inst_RHA_TO_ZL_CONVERTER.SPIMACHINE_STATE_0_RNIIB943[1]        NOR2       Y        Out     1.219     17.152      -         
un1_spimachine_state72_0_0_0                                   Net        -        -       0.773     -           1         
Inst_RHA_TO_ZL_CONVERTER.SPIMACHINE_STATE_RNI4EVAC[0]          NOR3A      A        In      -         17.924      -         
Inst_RHA_TO_ZL_CONVERTER.SPIMACHINE_STATE_RNI4EVAC[0]          NOR3A      Y        Out     1.595     19.520      -         
un1_spimachine_state72_0_2                                     Net        -        -       0.773     -           1         
Inst_RHA_TO_ZL_CONVERTER.BITMASK_SAMPLE_COPY_RNI2MGBG[0]       OR2A       A        In      -         20.292      -         
Inst_RHA_TO_ZL_CONVERTER.BITMASK_SAMPLE_COPY_RNI2MGBG[0]       OR2A       Y        Out     1.290     21.583      -         
BITMASK_SAMPLE_COPY_RNI2MGBG[0]                                Net        -        -       1.938     -           3         
Inst_RHA_TO_ZL_CONVERTER.un1_COUNTER_BITS_PROCESSED_1.I_1      AND2       B        In      -         23.521      -         
Inst_RHA_TO_ZL_CONVERTER.un1_COUNTER_BITS_PROCESSED_1.I_1      AND2       Y        Out     1.508     25.028      -         
DWACT_ADD_CI_0_TMP[0]                                          Net        -        -       0.927     -           2         
Inst_RHA_TO_ZL_CONVERTER.un1_COUNTER_BITS_PROCESSED_1.I_32     NOR2B      A        In      -         25.955      -         
Inst_RHA_TO_ZL_CONVERTER.un1_COUNTER_BITS_PROCESSED_1.I_32     NOR2B      Y        Out     1.236     27.192      -         
DWACT_ADD_CI_0_g_array_1[0]                                    Net        -        -       1.938     -           3         
Inst_RHA_TO_ZL_CONVERTER.un1_COUNTER_BITS_PROCESSED_1.I_38     NOR2B      A        In      -         29.129      -         
Inst_RHA_TO_ZL_CONVERTER.un1_COUNTER_BITS_PROCESSED_1.I_38     NOR2B      Y        Out     1.236     30.366      -         
DWACT_ADD_CI_0_g_array_2[0]                                    Net        -        -       1.938     -           3         
Inst_RHA_TO_ZL_CONVERTER.un1_COUNTER_BITS_PROCESSED_1.I_36     NOR2B      A        In      -         32.303      -         
Inst_RHA_TO_ZL_CONVERTER.un1_COUNTER_BITS_PROCESSED_1.I_36     NOR2B      Y        Out     1.236     33.540      -         
DWACT_ADD_CI_0_g_array_12_1[0]                                 Net        -        -       0.773     -           1         
Inst_RHA_TO_ZL_CONVERTER.un1_COUNTER_BITS_PROCESSED_1.I_29     XOR2       B        In      -         34.312      -         
Inst_RHA_TO_ZL_CONVERTER.un1_COUNTER_BITS_PROCESSED_1.I_29     XOR2       Y        Out     2.251     36.563      -         
I_29                                                           Net        -        -       0.773     -           1         
Inst_RHA_TO_ZL_CONVERTER.COUNTER_BITS_PROCESSED_RNO[5]         NOR2B      A        In      -         37.336      -         
Inst_RHA_TO_ZL_CONVERTER.COUNTER_BITS_PROCESSED_RNO[5]         NOR2B      Y        Out     1.174     38.509      -         
COUNTER_BITS_PROCESSED_9[5]                                    Net        -        -       0.773     -           1         
Inst_RHA_TO_ZL_CONVERTER.COUNTER_BITS_PROCESSED[5]             DFN1C1     D        In      -         39.282      -         
===========================================================================================================================
Total path delay (propagation time + setup) of 40.577 is 18.826(46.4%) logic and 21.750(53.6%) route.
Path delay compensated for clock skew. Clock skew is added to clock-to-out value, and is subtracted from setup time value


Path information for path number 5: 
      Requested Period:                      10.000
    - Setup time:                            1.295
    + Clock delay at ending point:           0.000 (ideal)
    = Required time:                         8.705

    - Propagation time:                      39.157
    - Clock delay at starting point:         0.000 (ideal)
    = Slack (non-critical) :                 -30.451

    Number of logic level(s):                11
    Starting point:                          Inst_RHA_TO_ZL_CONVERTER.SPIMACHINE_STATE_1[2] / Q
    Ending point:                            Inst_RHA_TO_ZL_CONVERTER.COUNTER_BITS_PROCESSED[6] / D
    The start point is clocked by            DATA_ACQUISITION_BLOCK|Clk [rising] on pin CLK
    The end   point is clocked by            DATA_ACQUISITION_BLOCK|Clk [rising] on pin CLK

Instance / Net                                                            Pin      Pin               Arrival     No. of    
Name                                                           Type       Name     Dir     Delay     Time        Fan Out(s)
---------------------------------------------------------------------------------------------------------------------------
Inst_RHA_TO_ZL_CONVERTER.SPIMACHINE_STATE_1[2]                 DFN1E0     Q        Out     1.771     1.771       -         
SPIMACHINE_STATE_1[2]                                          Net        -        -       5.722     -           21        
Inst_RHA_TO_ZL_CONVERTER.SPIMACHINE_STATE_0_RNIRQB41[1]        NOR3B      B        In      -         7.492       -         
Inst_RHA_TO_ZL_CONVERTER.SPIMACHINE_STATE_0_RNIRQB41[1]        NOR3B      Y        Out     1.458     8.950       -         
un1_spimachine_state72_4_a3_1                                  Net        -        -       0.773     -           1         
Inst_RHA_TO_ZL_CONVERTER.SPIMACHINE_STATE_0_RNIPL4I1_0[1]      NOR2B      B        In      -         9.722       -         
Inst_RHA_TO_ZL_CONVERTER.SPIMACHINE_STATE_0_RNIPL4I1_0[1]      NOR2B      Y        Out     1.508     11.230      -         
spimachine_state76                                             Net        -        -       4.009     -           9         
Inst_RHA_TO_ZL_CONVERTER.SPIMACHINE_STATE_0_RNIIB943[1]        NOR2       B        In      -         15.239      -         
Inst_RHA_TO_ZL_CONVERTER.SPIMACHINE_STATE_0_RNIIB943[1]        NOR2       Y        Out     1.554     16.793      -         
un1_spimachine_state72_0_0_0                                   Net        -        -       0.773     -           1         
Inst_RHA_TO_ZL_CONVERTER.SPIMACHINE_STATE_RNI4EVAC[0]          NOR3A      A        In      -         17.566      -         
Inst_RHA_TO_ZL_CONVERTER.SPIMACHINE_STATE_RNI4EVAC[0]          NOR3A      Y        Out     1.595     19.161      -         
un1_spimachine_state72_0_2                                     Net        -        -       0.773     -           1         
Inst_RHA_TO_ZL_CONVERTER.BITMASK_SAMPLE_COPY_RNI2MGBG[0]       OR2A       A        In      -         19.933      -         
Inst_RHA_TO_ZL_CONVERTER.BITMASK_SAMPLE_COPY_RNI2MGBG[0]       OR2A       Y        Out     1.290     21.224      -         
BITMASK_SAMPLE_COPY_RNI2MGBG[0]                                Net        -        -       1.938     -           3         
Inst_RHA_TO_ZL_CONVERTER.un1_COUNTER_BITS_PROCESSED_1.I_1      AND2       B        In      -         23.162      -         
Inst_RHA_TO_ZL_CONVERTER.un1_COUNTER_BITS_PROCESSED_1.I_1      AND2       Y        Out     1.508     24.669      -         
DWACT_ADD_CI_0_TMP[0]                                          Net        -        -       0.927     -           2         
Inst_RHA_TO_ZL_CONVERTER.un1_COUNTER_BITS_PROCESSED_1.I_32     NOR2B      A        In      -         25.596      -         
Inst_RHA_TO_ZL_CONVERTER.un1_COUNTER_BITS_PROCESSED_1.I_32     NOR2B      Y        Out     1.236     26.833      -         
DWACT_ADD_CI_0_g_array_1[0]                                    Net        -        -       1.938     -           3         
Inst_RHA_TO_ZL_CONVERTER.un1_COUNTER_BITS_PROCESSED_1.I_38     NOR2B      A        In      -         28.770      -         
Inst_RHA_TO_ZL_CONVERTER.un1_COUNTER_BITS_PROCESSED_1.I_38     NOR2B      Y        Out     1.236     30.007      -         
DWACT_ADD_CI_0_g_array_2[0]                                    Net        -        -       1.938     -           3         
Inst_RHA_TO_ZL_CONVERTER.un1_COUNTER_BITS_PROCESSED_1.I_35     NOR2B      A        In      -         31.944      -         
Inst_RHA_TO_ZL_CONVERTER.un1_COUNTER_BITS_PROCESSED_1.I_35     NOR2B      Y        Out     1.236     33.181      -         
DWACT_ADD_CI_0_g_array_11[0]                                   Net        -        -       0.773     -           1         
Inst_RHA_TO_ZL_CONVERTER.un1_COUNTER_BITS_PROCESSED_1.I_28     XOR2       B        In      -         33.953      -         
Inst_RHA_TO_ZL_CONVERTER.un1_COUNTER_BITS_PROCESSED_1.I_28     XOR2       Y        Out     2.251     36.204      -         
I_28                                                           Net        -        -       0.773     -           1         
Inst_RHA_TO_ZL_CONVERTER.COUNTER_BITS_PROCESSED_RNO[6]         MX2        B        In      -         36.977      -         
Inst_RHA_TO_ZL_CONVERTER.COUNTER_BITS_PROCESSED_RNO[6]         MX2        Y        Out     1.407     38.384      -         
COUNTER_BITS_PROCESSED_9[6]                                    Net        -        -       0.773     -           1         
Inst_RHA_TO_ZL_CONVERTER.COUNTER_BITS_PROCESSED[6]             DFN1C1     D        In      -         39.157      -         
===========================================================================================================================
Total path delay (propagation time + setup) of 40.451 is 19.344(47.8%) logic and 21.107(52.2%) route.
Path delay compensated for clock skew. Clock skew is added to clock-to-out value, and is subtracted from setup time value


====================================
Detailed Report for Clock: DATA_ACQUISITION_BLOCK|Clk_Slow
====================================


Starting Points with Worst Slack
********************************

                                   Starting                                                              Arrival            
Instance                           Reference                           Type       Pin     Net            Time        Slack  
                                   Clock                                                                                    
----------------------------------------------------------------------------------------------------------------------------
Inst_RHA_ARRAY.RHA4.Counter[1]     DATA_ACQUISITION_BLOCK|Clk_Slow     DFN1C1     Q       Counter[1]     1.771       -10.490
Inst_RHA_ARRAY.RHA7.Counter[1]     DATA_ACQUISITION_BLOCK|Clk_Slow     DFN1C1     Q       Counter[1]     1.771       -10.490
Inst_RHA_ARRAY.RHA3.Counter[1]     DATA_ACQUISITION_BLOCK|Clk_Slow     DFN1C1     Q       Counter[1]     1.771       -10.490
Inst_RHA_ARRAY.RHA0.Counter[1]     DATA_ACQUISITION_BLOCK|Clk_Slow     DFN1C1     Q       Counter[1]     1.771       -10.490
Inst_RHA_ARRAY.RHA6.Counter[1]     DATA_ACQUISITION_BLOCK|Clk_Slow     DFN1C1     Q       Counter[1]     1.771       -10.490
Inst_RHA_ARRAY.RHA2.Counter[1]     DATA_ACQUISITION_BLOCK|Clk_Slow     DFN1C1     Q       Counter[1]     1.771       -10.490
Inst_RHA_ARRAY.RHA5.Counter[1]     DATA_ACQUISITION_BLOCK|Clk_Slow     DFN1C1     Q       Counter[1]     1.771       -10.490
Inst_RHA_ARRAY.RHA1.Counter[1]     DATA_ACQUISITION_BLOCK|Clk_Slow     DFN1C1     Q       Counter[1]     1.771       -10.490
Inst_RHA_ARRAY.RHA0.Counter[0]     DATA_ACQUISITION_BLOCK|Clk_Slow     DFN1C1     Q       Counter[0]     1.771       -10.390
Inst_RHA_ARRAY.RHA4.Counter[0]     DATA_ACQUISITION_BLOCK|Clk_Slow     DFN1C1     Q       Counter[0]     1.771       -10.390
============================================================================================================================


Ending Points with Worst Slack
******************************

                                      Starting                                                                     Required            
Instance                              Reference                           Type       Pin     Net                   Time         Slack  
                                      Clock                                                                                            
---------------------------------------------------------------------------------------------------------------------------------------
Inst_RHA_ARRAY.RHA3.DATAOUT_FIRST     DATA_ACQUISITION_BLOCK|Clk_Slow     DFN0C1     D       DATAOUT_FIRST_3_0     3.288        -10.490
Inst_RHA_ARRAY.RHA7.DATAOUT_FIRST     DATA_ACQUISITION_BLOCK|Clk_Slow     DFN0C1     D       DATAOUT_FIRST_3       3.288        -10.490
Inst_RHA_ARRAY.RHA2.DATAOUT_FIRST     DATA_ACQUISITION_BLOCK|Clk_Slow     DFN0C1     D       DATAOUT_FIRST_3       3.288        -10.490
Inst_RHA_ARRAY.RHA4.DATAOUT_FIRST     DATA_ACQUISITION_BLOCK|Clk_Slow     DFN0C1     D       DATAOUT_FIRST_3       3.288        -10.490
Inst_RHA_ARRAY.RHA6.DATAOUT_FIRST     DATA_ACQUISITION_BLOCK|Clk_Slow     DFN0C1     D       DATAOUT_FIRST_3       3.288        -10.490
Inst_RHA_ARRAY.RHA1.DATAOUT_FIRST     DATA_ACQUISITION_BLOCK|Clk_Slow     DFN0C1     D       DATAOUT_FIRST_3       3.288        -10.490
Inst_RHA_ARRAY.RHA5.DATAOUT_FIRST     DATA_ACQUISITION_BLOCK|Clk_Slow     DFN0C1     D       DATAOUT_FIRST_3       3.288        -10.490
Inst_RHA_ARRAY.RHA0.DATAOUT_FIRST     DATA_ACQUISITION_BLOCK|Clk_Slow     DFN0C1     D       DATAOUT_FIRST_3       3.288        -10.490
Inst_RHA_ARRAY.RHA3.DATAOUT_VALID     DATA_ACQUISITION_BLOCK|Clk_Slow     DFN0C1     D       un3_counter           3.288        -8.544 
Inst_RHA_ARRAY.RHA2.DATAOUT_VALID     DATA_ACQUISITION_BLOCK|Clk_Slow     DFN0C1     D       un3_counter           3.288        -8.544 
=======================================================================================================================================


Worst Path Information
View Worst Path in Analyst
***********************


Path information for path number 1: 
      Requested Period:                      5.000
    - Setup time:                            1.712
    + Clock delay at ending point:           0.000 (ideal)
    = Required time:                         3.288

    - Propagation time:                      13.778
    - Clock delay at starting point:         0.000 (ideal)
    = Slack (non-critical) :                 -10.490

    Number of logic level(s):                3
    Starting point:                          Inst_RHA_ARRAY.RHA4.Counter[1] / Q
    Ending point:                            Inst_RHA_ARRAY.RHA4.DATAOUT_FIRST / D
    The start point is clocked by            DATA_ACQUISITION_BLOCK|Clk_Slow [rising] on pin CLK
    The end   point is clocked by            DATA_ACQUISITION_BLOCK|Clk_Slow [falling] on pin CLK

Instance / Net                                        Pin      Pin               Arrival     No. of    
Name                                       Type       Name     Dir     Delay     Time        Fan Out(s)
-------------------------------------------------------------------------------------------------------
Inst_RHA_ARRAY.RHA4.Counter[1]             DFN1C1     Q        Out     1.771     1.771       -         
Counter[1]                                 Net        -        -       3.074     -           5         
Inst_RHA_ARRAY.RHA4.Counter_RNILRJ[2]      NOR2       B        In      -         4.845       -         
Inst_RHA_ARRAY.RHA4.Counter_RNILRJ[2]      NOR2       Y        Out     1.554     6.398       -         
un3_counter_1                              Net        -        -       0.773     -           1         
Inst_RHA_ARRAY.RHA4.Counter_RNINNH1[2]     NOR2B      B        In      -         7.171       -         
Inst_RHA_ARRAY.RHA4.Counter_RNINNH1[2]     NOR2B      Y        Out     1.240     8.411       -         
un3_counter                                Net        -        -       3.420     -           6         
Inst_RHA_ARRAY.RHA4.DATAOUT_FIRST_RNO      NOR2B      A        In      -         11.831      -         
Inst_RHA_ARRAY.RHA4.DATAOUT_FIRST_RNO      NOR2B      Y        Out     1.174     13.005      -         
DATAOUT_FIRST_3                            Net        -        -       0.773     -           1         
Inst_RHA_ARRAY.RHA4.DATAOUT_FIRST          DFN0C1     D        In      -         13.778      -         
=======================================================================================================
Total path delay (propagation time + setup) of 15.490 is 7.450(48.1%) logic and 8.039(51.9%) route.
Path delay compensated for clock skew. Clock skew is added to clock-to-out value, and is subtracted from setup time value


Path information for path number 2: 
      Requested Period:                      5.000
    - Setup time:                            1.712
    + Clock delay at ending point:           0.000 (ideal)
    = Required time:                         3.288

    - Propagation time:                      13.778
    - Clock delay at starting point:         0.000 (ideal)
    = Slack (non-critical) :                 -10.490

    Number of logic level(s):                3
    Starting point:                          Inst_RHA_ARRAY.RHA7.Counter[1] / Q
    Ending point:                            Inst_RHA_ARRAY.RHA7.DATAOUT_FIRST / D
    The start point is clocked by            DATA_ACQUISITION_BLOCK|Clk_Slow [rising] on pin CLK
    The end   point is clocked by            DATA_ACQUISITION_BLOCK|Clk_Slow [falling] on pin CLK

Instance / Net                                        Pin      Pin               Arrival     No. of    
Name                                       Type       Name     Dir     Delay     Time        Fan Out(s)
-------------------------------------------------------------------------------------------------------
Inst_RHA_ARRAY.RHA7.Counter[1]             DFN1C1     Q        Out     1.771     1.771       -         
Counter[1]                                 Net        -        -       3.074     -           5         
Inst_RHA_ARRAY.RHA7.Counter_RNIRTV8[2]     NOR2       B        In      -         4.845       -         
Inst_RHA_ARRAY.RHA7.Counter_RNIRTV8[2]     NOR2       Y        Out     1.554     6.398       -         
un3_counter_1                              Net        -        -       0.773     -           1         
Inst_RHA_ARRAY.RHA7.Counter_RNI6TFM[2]     NOR2B      B        In      -         7.171       -         
Inst_RHA_ARRAY.RHA7.Counter_RNI6TFM[2]     NOR2B      Y        Out     1.240     8.411       -         
un3_counter                                Net        -        -       3.420     -           6         
Inst_RHA_ARRAY.RHA7.DATAOUT_FIRST_RNO      NOR2B      A        In      -         11.831      -         
Inst_RHA_ARRAY.RHA7.DATAOUT_FIRST_RNO      NOR2B      Y        Out     1.174     13.005      -         
DATAOUT_FIRST_3                            Net        -        -       0.773     -           1         
Inst_RHA_ARRAY.RHA7.DATAOUT_FIRST          DFN0C1     D        In      -         13.778      -         
=======================================================================================================
Total path delay (propagation time + setup) of 15.490 is 7.450(48.1%) logic and 8.039(51.9%) route.
Path delay compensated for clock skew. Clock skew is added to clock-to-out value, and is subtracted from setup time value


Path information for path number 3: 
      Requested Period:                      5.000
    - Setup time:                            1.712
    + Clock delay at ending point:           0.000 (ideal)
    = Required time:                         3.288

    - Propagation time:                      13.778
    - Clock delay at starting point:         0.000 (ideal)
    = Slack (non-critical) :                 -10.490

    Number of logic level(s):                3
    Starting point:                          Inst_RHA_ARRAY.RHA3.Counter[1] / Q
    Ending point:                            Inst_RHA_ARRAY.RHA3.DATAOUT_FIRST / D
    The start point is clocked by            DATA_ACQUISITION_BLOCK|Clk_Slow [rising] on pin CLK
    The end   point is clocked by            DATA_ACQUISITION_BLOCK|Clk_Slow [falling] on pin CLK

Instance / Net                                         Pin      Pin               Arrival     No. of    
Name                                        Type       Name     Dir     Delay     Time        Fan Out(s)
--------------------------------------------------------------------------------------------------------
Inst_RHA_ARRAY.RHA3.Counter[1]              DFN1C1     Q        Out     1.771     1.771       -         
Counter[1]                                  Net        -        -       3.074     -           5         
Inst_RHA_ARRAY.RHA3.Counter_RNIJ55J[2]      NOR2       B        In      -         4.845       -         
Inst_RHA_ARRAY.RHA3.Counter_RNIJ55J[2]      NOR2       Y        Out     1.554     6.398       -         
un3_counter_1                               Net        -        -       0.773     -           1         
Inst_RHA_ARRAY.RHA3.Counter_RNII0TF1[2]     NOR2B      B        In      -         7.171       -         
Inst_RHA_ARRAY.RHA3.Counter_RNII0TF1[2]     NOR2B      Y        Out     1.240     8.411       -         
un3_counter                                 Net        -        -       3.420     -           6         
Inst_RHA_ARRAY.RHA3.DATAOUT_FIRST_RNO       NOR2B      A        In      -         11.831      -         
Inst_RHA_ARRAY.RHA3.DATAOUT_FIRST_RNO       NOR2B      Y        Out     1.174     13.005      -         
DATAOUT_FIRST_3_0                           Net        -        -       0.773     -           1         
Inst_RHA_ARRAY.RHA3.DATAOUT_FIRST           DFN0C1     D        In      -         13.778      -         
========================================================================================================
Total path delay (propagation time + setup) of 15.490 is 7.450(48.1%) logic and 8.039(51.9%) route.
Path delay compensated for clock skew. Clock skew is added to clock-to-out value, and is subtracted from setup time value


Path information for path number 4: 
      Requested Period:                      5.000
    - Setup time:                            1.712
    + Clock delay at ending point:           0.000 (ideal)
    = Required time:                         3.288

    - Propagation time:                      13.778
    - Clock delay at starting point:         0.000 (ideal)
    = Slack (non-critical) :                 -10.490

    Number of logic level(s):                3
    Starting point:                          Inst_RHA_ARRAY.RHA0.Counter[1] / Q
    Ending point:                            Inst_RHA_ARRAY.RHA0.DATAOUT_FIRST / D
    The start point is clocked by            DATA_ACQUISITION_BLOCK|Clk_Slow [rising] on pin CLK
    The end   point is clocked by            DATA_ACQUISITION_BLOCK|Clk_Slow [falling] on pin CLK

Instance / Net                                        Pin      Pin               Arrival     No. of    
Name                                       Type       Name     Dir     Delay     Time        Fan Out(s)
-------------------------------------------------------------------------------------------------------
Inst_RHA_ARRAY.RHA0.Counter[1]             DFN1C1     Q        Out     1.771     1.771       -         
Counter[1]                                 Net        -        -       3.074     -           5         
Inst_RHA_ARRAY.RHA0.Counter_RNID3PA[2]     NOR2       B        In      -         4.845       -         
Inst_RHA_ARRAY.RHA0.Counter_RNID3PA[2]     NOR2       Y        Out     1.554     6.398       -         
un3_counter_1                              Net        -        -       0.773     -           1         
Inst_RHA_ARRAY.RHA0.Counter_RNI3RUQ[2]     NOR2B      B        In      -         7.171       -         
Inst_RHA_ARRAY.RHA0.Counter_RNI3RUQ[2]     NOR2B      Y        Out     1.240     8.411       -         
un3_counter                                Net        -        -       3.420     -           6         
Inst_RHA_ARRAY.RHA0.DATAOUT_FIRST_RNO      NOR2B      A        In      -         11.831      -         
Inst_RHA_ARRAY.RHA0.DATAOUT_FIRST_RNO      NOR2B      Y        Out     1.174     13.005      -         
DATAOUT_FIRST_3                            Net        -        -       0.773     -           1         
Inst_RHA_ARRAY.RHA0.DATAOUT_FIRST          DFN0C1     D        In      -         13.778      -         
=======================================================================================================
Total path delay (propagation time + setup) of 15.490 is 7.450(48.1%) logic and 8.039(51.9%) route.
Path delay compensated for clock skew. Clock skew is added to clock-to-out value, and is subtracted from setup time value


Path information for path number 5: 
      Requested Period:                      5.000
    - Setup time:                            1.712
    + Clock delay at ending point:           0.000 (ideal)
    = Required time:                         3.288

    - Propagation time:                      13.778
    - Clock delay at starting point:         0.000 (ideal)
    = Slack (non-critical) :                 -10.490

    Number of logic level(s):                3
    Starting point:                          Inst_RHA_ARRAY.RHA6.Counter[1] / Q
    Ending point:                            Inst_RHA_ARRAY.RHA6.DATAOUT_FIRST / D
    The start point is clocked by            DATA_ACQUISITION_BLOCK|Clk_Slow [rising] on pin CLK
    The end   point is clocked by            DATA_ACQUISITION_BLOCK|Clk_Slow [falling] on pin CLK

Instance / Net                                         Pin      Pin               Arrival     No. of    
Name                                        Type       Name     Dir     Delay     Time        Fan Out(s)
--------------------------------------------------------------------------------------------------------
Inst_RHA_ARRAY.RHA6.Counter[1]              DFN1C1     Q        Out     1.771     1.771       -         
Counter[1]                                  Net        -        -       3.074     -           5         
Inst_RHA_ARRAY.RHA6.Counter_RNIP7HR[2]      NOR2       B        In      -         4.845       -         
Inst_RHA_ARRAY.RHA6.Counter_RNIP7HR[2]      NOR2       Y        Out     1.554     6.398       -         
un3_counter_1                               Net        -        -       0.773     -           1         
Inst_RHA_ARRAY.RHA6.Counter_RNI16R42[2]     NOR2B      B        In      -         7.171       -         
Inst_RHA_ARRAY.RHA6.Counter_RNI16R42[2]     NOR2B      Y        Out     1.240     8.411       -         
un3_counter                                 Net        -        -       3.420     -           6         
Inst_RHA_ARRAY.RHA6.DATAOUT_FIRST_RNO       NOR2B      A        In      -         11.831      -         
Inst_RHA_ARRAY.RHA6.DATAOUT_FIRST_RNO       NOR2B      Y        Out     1.174     13.005      -         
DATAOUT_FIRST_3                             Net        -        -       0.773     -           1         
Inst_RHA_ARRAY.RHA6.DATAOUT_FIRST           DFN0C1     D        In      -         13.778      -         
========================================================================================================
Total path delay (propagation time + setup) of 15.490 is 7.450(48.1%) logic and 8.039(51.9%) route.
Path delay compensated for clock skew. Clock skew is added to clock-to-out value, and is subtracted from setup time value


====================================
Detailed Report for Clock: DATA_ACQUISITION_BLOCK|RHA_ADC_clk_TEST_inferred_clock
====================================


Starting Points with Worst Slack
********************************

                                                           Starting                                                                                              Arrival            
Instance                                                   Reference                                                  Type       Pin     Net                     Time        Slack  
                                                           Clock                                                                                                                    
------------------------------------------------------------------------------------------------------------------------------------------------------------------------------------
Inst_RHA_TESTMODULE.RHA_TEST_MOD_1.SPIMACHINE_STATE[2]     DATA_ACQUISITION_BLOCK|RHA_ADC_clk_TEST_inferred_clock     DFN0E0     Q       SPIMACHINE_STATE[2]     1.570       -19.782
Inst_RHA_TESTMODULE.RHA_TEST_MOD_1.SPIMACHINE_STATE[1]     DATA_ACQUISITION_BLOCK|RHA_ADC_clk_TEST_inferred_clock     DFN0E0     Q       SPIMACHINE_STATE[1]     1.570       -19.707
Inst_RHA_TESTMODULE.RHA_TEST_MOD_3.SPIMACHINE_STATE[0]     DATA_ACQUISITION_BLOCK|RHA_ADC_clk_TEST_inferred_clock     DFN0E0     Q       SPIMACHINE_STATE[0]     1.570       -17.423
Inst_RHA_TESTMODULE.RHA_TEST_MOD_4.SPIMACHINE_STATE[2]     DATA_ACQUISITION_BLOCK|RHA_ADC_clk_TEST_inferred_clock     DFN0E0     Q       SPIMACHINE_STATE[2]     1.570       -17.364
Inst_RHA_TESTMODULE.RHA_TEST_MOD_4.SPIMACHINE_STATE[1]     DATA_ACQUISITION_BLOCK|RHA_ADC_clk_TEST_inferred_clock     DFN0E0     Q       SPIMACHINE_STATE[1]     1.570       -17.289
Inst_RHA_TESTMODULE.RHA_TEST_MOD_3.SPIMACHINE_STATE[3]     DATA_ACQUISITION_BLOCK|RHA_ADC_clk_TEST_inferred_clock     DFN0E0     Q       SPIMACHINE_STATE[3]     1.265       -17.222
Inst_RHA_TESTMODULE.RHA_TEST_MOD_0.SPIMACHINE_STATE[2]     DATA_ACQUISITION_BLOCK|RHA_ADC_clk_TEST_inferred_clock     DFN0E0     Q       SPIMACHINE_STATE[2]     1.570       -17.130
Inst_RHA_TESTMODULE.RHA_TEST_MOD_0.SPIMACHINE_STATE[4]     DATA_ACQUISITION_BLOCK|RHA_ADC_clk_TEST_inferred_clock     DFN0E0     Q       SPIMACHINE_STATE[4]     1.570       -17.084
Inst_RHA_TESTMODULE.RHA_TEST_MOD_5.SPIMACHINE_STATE[0]     DATA_ACQUISITION_BLOCK|RHA_ADC_clk_TEST_inferred_clock     DFN0E0     Q       SPIMACHINE_STATE[0]     1.570       -17.076
Inst_RHA_TESTMODULE.RHA_TEST_MOD_4.SPIMACHINE_STATE[4]     DATA_ACQUISITION_BLOCK|RHA_ADC_clk_TEST_inferred_clock     DFN0E0     Q       SPIMACHINE_STATE[4]     1.570       -17.043
====================================================================================================================================================================================


Ending Points with Worst Slack
******************************

                                                              Starting                                                                                                        Required            
Instance                                                      Reference                                                  Type         Pin     Net                             Time         Slack  
                                                              Clock                                                                                                                               
--------------------------------------------------------------------------------------------------------------------------------------------------------------------------------------------------
Inst_RHA_TESTMODULE.RHA_TEST_MOD_1.TESTVALUE_INTERNAL[0]      DATA_ACQUISITION_BLOCK|RHA_ADC_clk_TEST_inferred_clock     DFN1C1       D       TESTVALUE_INTERNAL_RNO_0[0]     3.622        -19.782
Inst_RHA_TESTMODULE.RHA_TEST_MOD_1.TESTVALUE_INTERNAL[1]      DATA_ACQUISITION_BLOCK|RHA_ADC_clk_TEST_inferred_clock     DFN1C1       D       TESTVALUE_INTERNAL_RNO_0[1]     3.622        -19.782
Inst_RHA_TESTMODULE.RHA_TEST_MOD_1.TESTVALUE_INTERNAL[21]     DATA_ACQUISITION_BLOCK|RHA_ADC_clk_TEST_inferred_clock     DFN1C1       D       TESTVALUE_INTERNAL_RNO[21]      3.622        -19.782
Inst_RHA_TESTMODULE.RHA_TEST_MOD_1.LastChannel_Internal       DATA_ACQUISITION_BLOCK|RHA_ADC_clk_TEST_inferred_clock     DFN1E1C1     E       adc_out127                      3.538        -17.540
Inst_RHA_TESTMODULE.RHA_TEST_MOD_1.TESTVALUE_INTERNAL[3]      DATA_ACQUISITION_BLOCK|RHA_ADC_clk_TEST_inferred_clock     DFN1E1C1     E       adc_out127                      3.538        -17.540
Inst_RHA_TESTMODULE.RHA_TEST_MOD_1.TESTVALUE_INTERNAL[4]      DATA_ACQUISITION_BLOCK|RHA_ADC_clk_TEST_inferred_clock     DFN1E1C1     E       adc_out127                      3.538        -17.540
Inst_RHA_TESTMODULE.RHA_TEST_MOD_1.TESTVALUE_INTERNAL[5]      DATA_ACQUISITION_BLOCK|RHA_ADC_clk_TEST_inferred_clock     DFN1E1C1     E       adc_out127                      3.538        -17.540
Inst_RHA_TESTMODULE.RHA_TEST_MOD_1.TESTVALUE_INTERNAL[6]      DATA_ACQUISITION_BLOCK|RHA_ADC_clk_TEST_inferred_clock     DFN1E1C1     E       adc_out127                      3.538        -17.540
Inst_RHA_TESTMODULE.RHA_TEST_MOD_1.TESTVALUE_INTERNAL[7]      DATA_ACQUISITION_BLOCK|RHA_ADC_clk_TEST_inferred_clock     DFN1E1C1     E       adc_out127                      3.538        -17.540
Inst_RHA_TESTMODULE.RHA_TEST_MOD_1.TESTVALUE_INTERNAL[8]      DATA_ACQUISITION_BLOCK|RHA_ADC_clk_TEST_inferred_clock     DFN1E1C1     E       adc_out127                      3.538        -17.540
==================================================================================================================================================================================================


Worst Path Information
View Worst Path in Analyst
***********************


Path information for path number 1: 
      Requested Period:                      5.000
    - Setup time:                            1.378
    + Clock delay at ending point:           0.000 (ideal)
    = Required time:                         3.622

    - Propagation time:                      23.404
    - Clock delay at starting point:         0.000 (ideal)
    = Slack (non-critical) :                 -19.782

    Number of logic level(s):                4
    Starting point:                          Inst_RHA_TESTMODULE.RHA_TEST_MOD_1.SPIMACHINE_STATE[2] / Q
    Ending point:                            Inst_RHA_TESTMODULE.RHA_TEST_MOD_1.TESTVALUE_INTERNAL[0] / D
    The start point is clocked by            DATA_ACQUISITION_BLOCK|RHA_ADC_clk_TEST_inferred_clock [falling] on pin CLK
    The end   point is clocked by            DATA_ACQUISITION_BLOCK|RHA_ADC_clk_TEST_inferred_clock [rising] on pin CLK

Instance / Net                                                                 Pin      Pin               Arrival     No. of    
Name                                                                Type       Name     Dir     Delay     Time        Fan Out(s)
--------------------------------------------------------------------------------------------------------------------------------
Inst_RHA_TESTMODULE.RHA_TEST_MOD_1.SPIMACHINE_STATE[2]              DFN0E0     Q        Out     1.570     1.570       -         
SPIMACHINE_STATE[2]                                                 Net        -        -       4.105     -           10        
Inst_RHA_TESTMODULE.RHA_TEST_MOD_1.SPIMACHINE_STATE_RNIPFUM[1]      OR2A       A        In      -         5.676       -         
Inst_RHA_TESTMODULE.RHA_TEST_MOD_1.SPIMACHINE_STATE_RNIPFUM[1]      OR2A       Y        Out     1.119     6.795       -         
N_42                                                                Net        -        -       3.420     -           6         
Inst_RHA_TESTMODULE.RHA_TEST_MOD_1.SPIMACHINE_STATE_RNI8QD21[4]     NOR2A      B        In      -         10.215      -         
Inst_RHA_TESTMODULE.RHA_TEST_MOD_1.SPIMACHINE_STATE_RNI8QD21[4]     NOR2A      Y        Out     0.927     11.142      -         
N_112                                                               Net        -        -       2.844     -           4         
Inst_RHA_TESTMODULE.RHA_TEST_MOD_1.SPIMACHINE_STATE_RNI1ACP1[3]     NOR2B      A        In      -         13.986      -         
Inst_RHA_TESTMODULE.RHA_TEST_MOD_1.SPIMACHINE_STATE_RNI1ACP1[3]     NOR2B      Y        Out     1.236     15.223      -         
adc_out127                                                          Net        -        -       5.855     -           23        
Inst_RHA_TESTMODULE.RHA_TEST_MOD_1.TESTVALUE_INTERNAL_RNO[0]        OR2        B        In      -         21.078      -         
Inst_RHA_TESTMODULE.RHA_TEST_MOD_1.TESTVALUE_INTERNAL_RNO[0]        OR2        Y        Out     1.554     22.632      -         
TESTVALUE_INTERNAL_RNO_0[0]                                         Net        -        -       0.773     -           1         
Inst_RHA_TESTMODULE.RHA_TEST_MOD_1.TESTVALUE_INTERNAL[0]            DFN1C1     D        In      -         23.404      -         
================================================================================================================================
Total path delay (propagation time + setup) of 24.782 is 7.785(31.4%) logic and 16.998(68.6%) route.
Path delay compensated for clock skew. Clock skew is added to clock-to-out value, and is subtracted from setup time value


Path information for path number 2: 
      Requested Period:                      5.000
    - Setup time:                            1.378
    + Clock delay at ending point:           0.000 (ideal)
    = Required time:                         3.622

    - Propagation time:                      23.404
    - Clock delay at starting point:         0.000 (ideal)
    = Slack (non-critical) :                 -19.782

    Number of logic level(s):                4
    Starting point:                          Inst_RHA_TESTMODULE.RHA_TEST_MOD_1.SPIMACHINE_STATE[2] / Q
    Ending point:                            Inst_RHA_TESTMODULE.RHA_TEST_MOD_1.TESTVALUE_INTERNAL[21] / D
    The start point is clocked by            DATA_ACQUISITION_BLOCK|RHA_ADC_clk_TEST_inferred_clock [falling] on pin CLK
    The end   point is clocked by            DATA_ACQUISITION_BLOCK|RHA_ADC_clk_TEST_inferred_clock [rising] on pin CLK

Instance / Net                                                                 Pin      Pin               Arrival     No. of    
Name                                                                Type       Name     Dir     Delay     Time        Fan Out(s)
--------------------------------------------------------------------------------------------------------------------------------
Inst_RHA_TESTMODULE.RHA_TEST_MOD_1.SPIMACHINE_STATE[2]              DFN0E0     Q        Out     1.570     1.570       -         
SPIMACHINE_STATE[2]                                                 Net        -        -       4.105     -           10        
Inst_RHA_TESTMODULE.RHA_TEST_MOD_1.SPIMACHINE_STATE_RNIPFUM[1]      OR2A       A        In      -         5.676       -         
Inst_RHA_TESTMODULE.RHA_TEST_MOD_1.SPIMACHINE_STATE_RNIPFUM[1]      OR2A       Y        Out     1.119     6.795       -         
N_42                                                                Net        -        -       3.420     -           6         
Inst_RHA_TESTMODULE.RHA_TEST_MOD_1.SPIMACHINE_STATE_RNI8QD21[4]     NOR2A      B        In      -         10.215      -         
Inst_RHA_TESTMODULE.RHA_TEST_MOD_1.SPIMACHINE_STATE_RNI8QD21[4]     NOR2A      Y        Out     0.927     11.142      -         
N_112                                                               Net        -        -       2.844     -           4         
Inst_RHA_TESTMODULE.RHA_TEST_MOD_1.SPIMACHINE_STATE_RNI1ACP1[3]     NOR2B      A        In      -         13.986      -         
Inst_RHA_TESTMODULE.RHA_TEST_MOD_1.SPIMACHINE_STATE_RNI1ACP1[3]     NOR2B      Y        Out     1.236     15.223      -         
adc_out127                                                          Net        -        -       5.855     -           23        
Inst_RHA_TESTMODULE.RHA_TEST_MOD_1.TESTVALUE_INTERNAL_RNO[21]       OR2        B        In      -         21.078      -         
Inst_RHA_TESTMODULE.RHA_TEST_MOD_1.TESTVALUE_INTERNAL_RNO[21]       OR2        Y        Out     1.554     22.632      -         
TESTVALUE_INTERNAL_RNO[21]                                          Net        -        -       0.773     -           1         
Inst_RHA_TESTMODULE.RHA_TEST_MOD_1.TESTVALUE_INTERNAL[21]           DFN1C1     D        In      -         23.404      -         
================================================================================================================================
Total path delay (propagation time + setup) of 24.782 is 7.785(31.4%) logic and 16.998(68.6%) route.
Path delay compensated for clock skew. Clock skew is added to clock-to-out value, and is subtracted from setup time value


Path information for path number 3: 
      Requested Period:                      5.000
    - Setup time:                            1.378
    + Clock delay at ending point:           0.000 (ideal)
    = Required time:                         3.622

    - Propagation time:                      23.404
    - Clock delay at starting point:         0.000 (ideal)
    = Slack (non-critical) :                 -19.782

    Number of logic level(s):                4
    Starting point:                          Inst_RHA_TESTMODULE.RHA_TEST_MOD_1.SPIMACHINE_STATE[2] / Q
    Ending point:                            Inst_RHA_TESTMODULE.RHA_TEST_MOD_1.TESTVALUE_INTERNAL[1] / D
    The start point is clocked by            DATA_ACQUISITION_BLOCK|RHA_ADC_clk_TEST_inferred_clock [falling] on pin CLK
    The end   point is clocked by            DATA_ACQUISITION_BLOCK|RHA_ADC_clk_TEST_inferred_clock [rising] on pin CLK

Instance / Net                                                                 Pin      Pin               Arrival     No. of    
Name                                                                Type       Name     Dir     Delay     Time        Fan Out(s)
--------------------------------------------------------------------------------------------------------------------------------
Inst_RHA_TESTMODULE.RHA_TEST_MOD_1.SPIMACHINE_STATE[2]              DFN0E0     Q        Out     1.570     1.570       -         
SPIMACHINE_STATE[2]                                                 Net        -        -       4.105     -           10        
Inst_RHA_TESTMODULE.RHA_TEST_MOD_1.SPIMACHINE_STATE_RNIPFUM[1]      OR2A       A        In      -         5.676       -         
Inst_RHA_TESTMODULE.RHA_TEST_MOD_1.SPIMACHINE_STATE_RNIPFUM[1]      OR2A       Y        Out     1.119     6.795       -         
N_42                                                                Net        -        -       3.420     -           6         
Inst_RHA_TESTMODULE.RHA_TEST_MOD_1.SPIMACHINE_STATE_RNI8QD21[4]     NOR2A      B        In      -         10.215      -         
Inst_RHA_TESTMODULE.RHA_TEST_MOD_1.SPIMACHINE_STATE_RNI8QD21[4]     NOR2A      Y        Out     0.927     11.142      -         
N_112                                                               Net        -        -       2.844     -           4         
Inst_RHA_TESTMODULE.RHA_TEST_MOD_1.SPIMACHINE_STATE_RNI1ACP1[3]     NOR2B      A        In      -         13.986      -         
Inst_RHA_TESTMODULE.RHA_TEST_MOD_1.SPIMACHINE_STATE_RNI1ACP1[3]     NOR2B      Y        Out     1.236     15.223      -         
adc_out127                                                          Net        -        -       5.855     -           23        
Inst_RHA_TESTMODULE.RHA_TEST_MOD_1.TESTVALUE_INTERNAL_RNO[1]        OR2        B        In      -         21.078      -         
Inst_RHA_TESTMODULE.RHA_TEST_MOD_1.TESTVALUE_INTERNAL_RNO[1]        OR2        Y        Out     1.554     22.632      -         
TESTVALUE_INTERNAL_RNO_0[1]                                         Net        -        -       0.773     -           1         
Inst_RHA_TESTMODULE.RHA_TEST_MOD_1.TESTVALUE_INTERNAL[1]            DFN1C1     D        In      -         23.404      -         
================================================================================================================================
Total path delay (propagation time + setup) of 24.782 is 7.785(31.4%) logic and 16.998(68.6%) route.
Path delay compensated for clock skew. Clock skew is added to clock-to-out value, and is subtracted from setup time value


Path information for path number 4: 
      Requested Period:                      5.000
    - Setup time:                            1.295
    + Clock delay at ending point:           0.000 (ideal)
    = Required time:                         3.705

    - Propagation time:                      23.413
    - Clock delay at starting point:         0.000 (ideal)
    = Slack (non-critical) :                 -19.707

    Number of logic level(s):                4
    Starting point:                          Inst_RHA_TESTMODULE.RHA_TEST_MOD_1.SPIMACHINE_STATE[1] / Q
    Ending point:                            Inst_RHA_TESTMODULE.RHA_TEST_MOD_1.TESTVALUE_INTERNAL[0] / D
    The start point is clocked by            DATA_ACQUISITION_BLOCK|RHA_ADC_clk_TEST_inferred_clock [falling] on pin CLK
    The end   point is clocked by            DATA_ACQUISITION_BLOCK|RHA_ADC_clk_TEST_inferred_clock [rising] on pin CLK

Instance / Net                                                                 Pin      Pin               Arrival     No. of    
Name                                                                Type       Name     Dir     Delay     Time        Fan Out(s)
--------------------------------------------------------------------------------------------------------------------------------
Inst_RHA_TESTMODULE.RHA_TEST_MOD_1.SPIMACHINE_STATE[1]              DFN0E0     Q        Out     1.570     1.570       -         
SPIMACHINE_STATE[1]                                                 Net        -        -       4.009     -           9         
Inst_RHA_TESTMODULE.RHA_TEST_MOD_1.SPIMACHINE_STATE_RNIPFUM[1]      OR2A       B        In      -         5.580       -         
Inst_RHA_TESTMODULE.RHA_TEST_MOD_1.SPIMACHINE_STATE_RNIPFUM[1]      OR2A       Y        Out     1.554     7.133       -         
N_42                                                                Net        -        -       3.420     -           6         
Inst_RHA_TESTMODULE.RHA_TEST_MOD_1.SPIMACHINE_STATE_RNI8QD21[4]     NOR2A      B        In      -         10.554      -         
Inst_RHA_TESTMODULE.RHA_TEST_MOD_1.SPIMACHINE_STATE_RNI8QD21[4]     NOR2A      Y        Out     0.977     11.531      -         
N_112                                                               Net        -        -       2.844     -           4         
Inst_RHA_TESTMODULE.RHA_TEST_MOD_1.SPIMACHINE_STATE_RNI1ACP1[3]     NOR2B      A        In      -         14.375      -         
Inst_RHA_TESTMODULE.RHA_TEST_MOD_1.SPIMACHINE_STATE_RNI1ACP1[3]     NOR2B      Y        Out     1.174     15.548      -         
adc_out127                                                          Net        -        -       5.855     -           23        
Inst_RHA_TESTMODULE.RHA_TEST_MOD_1.TESTVALUE_INTERNAL_RNO[0]        OR2        B        In      -         21.404      -         
Inst_RHA_TESTMODULE.RHA_TEST_MOD_1.TESTVALUE_INTERNAL_RNO[0]        OR2        Y        Out     1.236     22.640      -         
TESTVALUE_INTERNAL_RNO_0[0]                                         Net        -        -       0.773     -           1         
Inst_RHA_TESTMODULE.RHA_TEST_MOD_1.TESTVALUE_INTERNAL[0]            DFN1C1     D        In      -         23.413      -         
================================================================================================================================
Total path delay (propagation time + setup) of 24.707 is 7.805(31.6%) logic and 16.902(68.4%) route.
Path delay compensated for clock skew. Clock skew is added to clock-to-out value, and is subtracted from setup time value


Path information for path number 5: 
      Requested Period:                      5.000
    - Setup time:                            1.295
    + Clock delay at ending point:           0.000 (ideal)
    = Required time:                         3.705

    - Propagation time:                      23.413
    - Clock delay at starting point:         0.000 (ideal)
    = Slack (non-critical) :                 -19.707

    Number of logic level(s):                4
    Starting point:                          Inst_RHA_TESTMODULE.RHA_TEST_MOD_1.SPIMACHINE_STATE[1] / Q
    Ending point:                            Inst_RHA_TESTMODULE.RHA_TEST_MOD_1.TESTVALUE_INTERNAL[21] / D
    The start point is clocked by            DATA_ACQUISITION_BLOCK|RHA_ADC_clk_TEST_inferred_clock [falling] on pin CLK
    The end   point is clocked by            DATA_ACQUISITION_BLOCK|RHA_ADC_clk_TEST_inferred_clock [rising] on pin CLK

Instance / Net                                                                 Pin      Pin               Arrival     No. of    
Name                                                                Type       Name     Dir     Delay     Time        Fan Out(s)
--------------------------------------------------------------------------------------------------------------------------------
Inst_RHA_TESTMODULE.RHA_TEST_MOD_1.SPIMACHINE_STATE[1]              DFN0E0     Q        Out     1.570     1.570       -         
SPIMACHINE_STATE[1]                                                 Net        -        -       4.009     -           9         
Inst_RHA_TESTMODULE.RHA_TEST_MOD_1.SPIMACHINE_STATE_RNIPFUM[1]      OR2A       B        In      -         5.580       -         
Inst_RHA_TESTMODULE.RHA_TEST_MOD_1.SPIMACHINE_STATE_RNIPFUM[1]      OR2A       Y        Out     1.554     7.133       -         
N_42                                                                Net        -        -       3.420     -           6         
Inst_RHA_TESTMODULE.RHA_TEST_MOD_1.SPIMACHINE_STATE_RNI8QD21[4]     NOR2A      B        In      -         10.554      -         
Inst_RHA_TESTMODULE.RHA_TEST_MOD_1.SPIMACHINE_STATE_RNI8QD21[4]     NOR2A      Y        Out     0.977     11.531      -         
N_112                                                               Net        -        -       2.844     -           4         
Inst_RHA_TESTMODULE.RHA_TEST_MOD_1.SPIMACHINE_STATE_RNI1ACP1[3]     NOR2B      A        In      -         14.375      -         
Inst_RHA_TESTMODULE.RHA_TEST_MOD_1.SPIMACHINE_STATE_RNI1ACP1[3]     NOR2B      Y        Out     1.174     15.548      -         
adc_out127                                                          Net        -        -       5.855     -           23        
Inst_RHA_TESTMODULE.RHA_TEST_MOD_1.TESTVALUE_INTERNAL_RNO[21]       OR2        B        In      -         21.404      -         
Inst_RHA_TESTMODULE.RHA_TEST_MOD_1.TESTVALUE_INTERNAL_RNO[21]       OR2        Y        Out     1.236     22.640      -         
TESTVALUE_INTERNAL_RNO[21]                                          Net        -        -       0.773     -           1         
Inst_RHA_TESTMODULE.RHA_TEST_MOD_1.TESTVALUE_INTERNAL[21]           DFN1C1     D        In      -         23.413      -         
================================================================================================================================
Total path delay (propagation time + setup) of 24.707 is 7.805(31.6%) logic and 16.902(68.4%) route.
Path delay compensated for clock skew. Clock skew is added to clock-to-out value, and is subtracted from setup time value


##### END OF TIMING REPORT #####]

--------------------------------------------------------------------------------
Target Part: AGL250V2_VQFP100_STD
Report for cell DATA_ACQUISITION_BLOCK.behavioral
  Core Cell usage:
              cell count     area count*area
              AND2     4      1.0        4.0
              AND3    16      1.0       16.0
               AO1    35      1.0       35.0
              AO15     1      1.0        1.0
              AO16     1      1.0        1.0
              AO17     1      1.0        1.0
              AO1A     9      1.0        9.0
              AO1B    15      1.0       15.0
              AO1D     3      1.0        3.0
              AOI1     4      1.0        4.0
             AOI1B     7      1.0        7.0
              AX1C    12      1.0       12.0
            CLKINT     3      0.0        0.0
               GND    23      0.0        0.0
               INV     3      1.0        3.0
               MX2   689      1.0      689.0
              MX2A    11      1.0       11.0
              MX2B     2      1.0        2.0
              MX2C     2      1.0        2.0
              NOR2    78      1.0       78.0
             NOR2A   109      1.0      109.0
             NOR2B   192      1.0      192.0
              NOR3    20      1.0       20.0
             NOR3A    42      1.0       42.0
             NOR3B    46      1.0       46.0
             NOR3C    48      1.0       48.0
               OA1     4      1.0        4.0
              OA1A     1      1.0        1.0
              OA1B     3      1.0        3.0
              OA1C    16      1.0       16.0
              OAI1     7      1.0        7.0
               OR2    86      1.0       86.0
              OR2A    44      1.0       44.0
               OR3    29      1.0       29.0
              OR3A     5      1.0        5.0
              OR3B     2      1.0        2.0
              OR3C     1      1.0        1.0
               VCC    23      0.0        0.0
               XA1     1      1.0        1.0
              XA1A     2      1.0        2.0
              XA1C     4      1.0        4.0
               XO1     1      1.0        1.0
              XOR2    82      1.0       82.0
              XOR3     9      1.0        9.0


            DFN0C1   224      1.0      224.0
            DFN0E0    40      1.0       40.0
            DFN1C1   136      1.0      136.0
            DFN1E0    16      1.0       16.0
          DFN1E0C1     8      1.0        8.0
          DFN1E1C1   660      1.0      660.0
            DFN1P1    17      1.0       17.0
              DLN0     1      1.0        1.0
              DLN1     1      1.0        1.0
                   -----          ----------
             TOTAL  2799              2750.0


  IO Cell usage:
              cell count
            CLKBUF     3
             INBUF   186
            OUTBUF    24
                   -----
             TOTAL   213


Core Cells         : 2750 of 6144 (45%)
IO Cells           : 213

  RAM/ROM Usage Summary
Block Rams : 0 of 8 (0%)

Mapper successful!

At Mapper Exit (Real Time elapsed 0h:00m:04s; CPU Time elapsed 0h:00m:03s; Memory used current: 52MB peak: 126MB)

Process took 0h:00m:04s realtime, 0h:00m:03s cputime
# Fri Aug 01 16:09:20 2014

###########################################################]
``` ````
