# Supplementary material for: Implications for a Wireless, External Device System to Study Electrocorticography
Source: Sensors (Basel). 2017 Apr 4;17(4):761. doi: 10.3390/s17040761 (PMC5421721; doi:10.3390/s17040761)
Supplement: Supplementary file 1 [file sensors-17-00761-s001.zip › Supplement/RHA_FPGA_Firmware/IGLOO_RHA_05092014.zip!/IGLOO_RHA/synthesis/syntmp/DATA_ACQUISITION_BLOCK_toc.htm]

 


- **DATA\_ACQUISITION\_BLOCK\_syn (synthesis)**
  - **Synthesis -** 
    - Compiler Report
    - Pre-mapping Report
      - Clock Summary
    - Mapper Report
      - Clock Conversion
      - Timing Report
        - Performance Summary
        - Clock Relationships
        - Interface Information
        - Detailed Report for Clocks
          - Clock: DATA\_ACQUISITION\_BLOCK|Clk
            - Starting Points with Worst Slack
            - Ending Points with Worst Slack
            - Worst Path Information
          - Clock: DATA\_ACQUISITION\_BLOCK|Clk\_Slow
            - Starting Points with Worst Slack
            - Ending Points with Worst Slack
            - Worst Path Information
          - Clock: DATA\_ACQUISITION\_BLOCK|RHA\_ADC\_clk\_TEST\_inferred\_clock
            - Starting Points with Worst Slack
            - Ending Points with Worst Slack
            - Worst Path Information
      - Resource Utilization
