# Supplementary material for: Implications for a Wireless, External Device System to Study Electrocorticography
Source: Sensors (Basel). 2017 Apr 4;17(4):761. doi: 10.3390/s17040761 (PMC5421721; doi:10.3390/s17040761)
Supplement: Supplementary file 1 [file sensors-17-00761-s001.zip › Supplement/RHA_FPGA_Firmware/IGLOO_RHA_05092014.zip!/IGLOO_RHA/synthesis/syntmp/FIFO_srr.htm]

 ```` ```
#Build: Synplify Pro I-2013.09M-SP1 , Build 034R, Jan 17 2014
#install: C:\Microsemi\Libero_v11.3\Synopsys\synplify_I201309MSP1
#OS: Windows 7 6.1
#Hostname: ITP-PC

#Implementation: synthesis

$ Start of Compile
#Tue Aug 05 17:25:00 2014

Synopsys VHDL Compiler, version comp201309rcp1, Build 078R, built Jan 14 2014
@N: :  | Running in 64-bit mode 
Copyright (C) 1994-2013 Synopsys, Inc. This software and the associated documentation are proprietary to Synopsys, Inc. This software may only be used in accordance with the terms and conditions of a written license agreement with Synopsys, Inc.  All other use or distribution of the software is strictly prohibited.

@N:CD720 : std.vhd(123) | Setting time resolution to ns
@N: : FIFO.vhd(8) | Top entity is set to FIFO.
VHDL syntax check successful!
@N:CD630 : FIFO.vhd(8) | Synthesizing work.fifo.def_arch 
@N:CD630 : igloo.vhd(2722) | Synthesizing igloo.vcc.syn_black_box 
Post processing for igloo.vcc.syn_black_box
@N:CD630 : igloo.vhd(1787) | Synthesizing igloo.gnd.syn_black_box 
Post processing for igloo.gnd.syn_black_box
@N:CD630 : igloo.vhd(2198) | Synthesizing igloo.or2.syn_black_box 
Post processing for igloo.or2.syn_black_box
@N:CD630 : igloo.vhd(1934) | Synthesizing igloo.inv.syn_black_box 
Post processing for igloo.inv.syn_black_box
@N:CD630 : igloo.vhd(3039) | Synthesizing igloo.fifo4k18.syn_black_box 
Post processing for igloo.fifo4k18.syn_black_box
@N:CD630 : igloo.vhd(13) | Synthesizing igloo.and2.syn_black_box 
Post processing for igloo.and2.syn_black_box
@N:CD630 : igloo.vhd(2032) | Synthesizing igloo.nand2.syn_black_box 
Post processing for igloo.nand2.syn_black_box
@N:CD630 : igloo.vhd(2040) | Synthesizing igloo.nand2a.syn_black_box 
Post processing for igloo.nand2a.syn_black_box
Post processing for work.fifo.def_arch
@END

At c_vhdl Exit (Real Time elapsed 0h:00m:00s; CPU Time elapsed 0h:00m:00s; Memory used current: 70MB peak: 71MB)

Process took 0h:00m:01s realtime, 0h:00m:01s cputime
# Tue Aug 05 17:25:01 2014

###########################################################]
Pre-mapping Report

Synopsys Microsemi Technology Pre-mapping, Version mapact, Build 1154R, Built Jan 20 2014 10:14:08
Copyright (C) 1994-2013, Synopsys, Inc. This software and the associated documentation are proprietary to Synopsys, Inc. This software may only be used in accordance with the terms and conditions of a written license agreement with Synopsys, Inc.  All other use or distribution of the software is strictly prohibited.
Product Version I-2013.09M-SP1 

Mapper Startup Complete (Real Time elapsed 0h:00m:00s; CPU Time elapsed 0h:00m:00s; Memory used current: 98MB peak: 99MB)

Linked File: FIFO_scck.rpt
Printing clock  summary report in "C:\Users\ITP\Desktop\IGLOO\IGLOO_RHA\synthesis\FIFO_scck.rpt" file 
@N:MF248 :  | Running in 64-bit mode. 
@N:MF667 :  | Clock conversion disabled  

Design Input Complete (Real Time elapsed 0h:00m:00s; CPU Time elapsed 0h:00m:00s; Memory used current: 99MB peak: 101MB)


Mapper Initialization Complete (Real Time elapsed 0h:00m:00s; CPU Time elapsed 0h:00m:00s; Memory used current: 99MB peak: 101MB)


Clock Summary
**************

Start     Requested     Requested     Clock     Clock
Clock     Frequency     Period        Type      Group
-----------------------------------------------------
=====================================================

@W:MT532 : fifo.vhd(443) | Found signal identified as System clock which controls 0 sequential elements including \\FIFOBLOCK\[1\]\\.  Using this clock, which has no specified timing constraint, can adversely impact design performance. 

Finished Pre Mapping Phase.
@N:BN225 :  | Writing default property annotation file C:\Users\ITP\Desktop\IGLOO\IGLOO_RHA\synthesis\FIFO.sap. 
Pre-mapping successful!

At Mapper Exit (Real Time elapsed 0h:00m:01s; CPU Time elapsed 0h:00m:00s; Memory used current: 44MB peak: 109MB)

Process took 0h:00m:01s realtime, 0h:00m:01s cputime
# Tue Aug 05 17:25:04 2014

###########################################################]
Map & Optimize Report

Synopsys Microsemi Technology Mapper, Version mapact, Build 1154R, Built Jan 20 2014 10:14:08
Copyright (C) 1994-2013, Synopsys, Inc. This software and the associated documentation are proprietary to Synopsys, Inc. This software may only be used in accordance with the terms and conditions of a written license agreement with Synopsys, Inc.  All other use or distribution of the software is strictly prohibited.
Product Version I-2013.09M-SP1 

Mapper Startup Complete (Real Time elapsed 0h:00m:00s; CPU Time elapsed 0h:00m:00s; Memory used current: 98MB peak: 99MB)

@N:MF248 :  | Running in 64-bit mode. 
@N:MF667 :  | Clock conversion disabled  

Design Input Complete (Real Time elapsed 0h:00m:00s; CPU Time elapsed 0h:00m:00s; Memory used current: 99MB peak: 100MB)


Mapper Initialization Complete (Real Time elapsed 0h:00m:00s; CPU Time elapsed 0h:00m:00s; Memory used current: 99MB peak: 100MB)


Starting Optimization and Mapping (Real Time elapsed 0h:00m:00s; CPU Time elapsed 0h:00m:00s; Memory used current: 107MB peak: 109MB)


Available hyper_sources - for debug and ip models
	None Found


Finished RTL optimizations (Real Time elapsed 0h:00m:00s; CPU Time elapsed 0h:00m:00s; Memory used current: 107MB peak: 109MB)


Finished factoring (Real Time elapsed 0h:00m:00s; CPU Time elapsed 0h:00m:00s; Memory used current: 107MB peak: 109MB)


Finished gated-clock and generated-clock conversion (Real Time elapsed 0h:00m:00s; CPU Time elapsed 0h:00m:00s; Memory used current: 107MB peak: 109MB)


Finished generic timing optimizations - Pass 1 (Real Time elapsed 0h:00m:00s; CPU Time elapsed 0h:00m:00s; Memory used current: 107MB peak: 109MB)


Starting Early Timing Optimization (Real Time elapsed 0h:00m:00s; CPU Time elapsed 0h:00m:00s; Memory used current: 108MB peak: 109MB)


Finished Early Timing Optimization (Real Time elapsed 0h:00m:00s; CPU Time elapsed 0h:00m:00s; Memory used current: 108MB peak: 109MB)


Finished generic timing optimizations - Pass 2 (Real Time elapsed 0h:00m:00s; CPU Time elapsed 0h:00m:00s; Memory used current: 107MB peak: 109MB)


Finished preparing to map (Real Time elapsed 0h:00m:00s; CPU Time elapsed 0h:00m:00s; Memory used current: 108MB peak: 109MB)


Finished technology mapping (Real Time elapsed 0h:00m:00s; CPU Time elapsed 0h:00m:00s; Memory used current: 107MB peak: 109MB)

@N:FP130 :  | Promoting Net WR_CLK_c on CLKBUF  WR_CLK_pad  
@N:FP130 :  | Promoting Net RD_CLK_c on CLKBUF  RD_CLK_pad  

Finished technology timing optimizations and critical path resynthesis (Real Time elapsed 0h:00m:00s; CPU Time elapsed 0h:00m:00s; Memory used current: 107MB peak: 109MB)


Added 0 Buffers
Added 0 Cells via replication
	Added 0 Sequential Cells via replication
	Added 0 Combinational Cells via replication

Finished restoring hierarchy (Real Time elapsed 0h:00m:00s; CPU Time elapsed 0h:00m:00s; Memory used current: 107MB peak: 109MB)


#### START OF CLOCK OPTIMIZATION REPORT #####[

Clock optimization not enabled
2 non-gated/non-generated clock tree(s) driving 16 clock pin(s) of sequential element(s)
0 gated/generated clock tree(s) driving 0 clock pin(s) of sequential element(s)
0 instances converted, 0 sequential instances remain driven by gated/generated clocks

=========================== Non-Gated/Non-Generated Clocks ============================
Clock Tree ID     Driving Element     Drive Element Type     Fanout     Sample Instance
---------------------------------------------------------------------------------------
ClockId0001        RD_CLK              port                   8          \FIFOBLOCK[0]\ 
ClockId0002        WR_CLK              port                   8          \FIFOBLOCK[0]\ 
=======================================================================================


##### END OF CLOCK OPTIMIZATION REPORT ######]

Writing Analyst data base C:\Users\ITP\Desktop\IGLOO\IGLOO_RHA\synthesis\FIFO.srm

Finished Writing Netlist Databases (Real Time elapsed 0h:00m:00s; CPU Time elapsed 0h:00m:00s; Memory used current: 107MB peak: 109MB)

Writing EDIF Netlist and constraint files
I-2013.09M-SP1 

Finished Writing EDIF Netlist and constraint files (Real Time elapsed 0h:00m:00s; CPU Time elapsed 0h:00m:00s; Memory used current: 108MB peak: 109MB)

@W:MT420 :  | Found inferred clock FIFO|WR_CLK with period 10.00ns. Please declare a user-defined clock on object "p:WR_CLK" 

@W:MT420 :  | Found inferred clock FIFO|RD_CLK with period 10.00ns. Please declare a user-defined clock on object "p:RD_CLK" 


##### START OF TIMING REPORT #####[
# Timing Report written on Tue Aug 05 17:25:05 2014
#


Top view:               FIFO
Library name:           IGLOO_V2
Operating conditions:   COMWCSTD ( T = 70.0, V = 1.14, P = 3.70, tree_type = balanced_tree )
Requested Frequency:    100.0 MHz
Wire load mode:         top
Wire load model:        igloo
Paths requested:        5
Constraint File(s):    
@N:MT320 :  | Timing report estimates place and route data. Please look at the place and route timing report for final timing. 

@N:MT322 :  | Clock constraints cover only FF-to-FF paths associated with the clock. 


Performance Summary 
*******************


Worst slack in design: -14.218

                   Requested     Estimated     Requested     Estimated                 Clock        Clock              
Starting Clock     Frequency     Frequency     Period        Period        Slack       Type         Group              
-----------------------------------------------------------------------------------------------------------------------
FIFO|RD_CLK        100.0 MHz     41.3 MHz      10.000        24.218        -14.218     inferred     Inferred_clkgroup_1
FIFO|WR_CLK        100.0 MHz     44.8 MHz      10.000        22.302        -12.302     inferred     Inferred_clkgroup_0
=======================================================================================================================


Clock Relationships
*******************

Clocks                    |    rise  to  rise     |    fall  to  fall   |    rise  to  fall   |    fall  to  rise 
------------------------------------------------------------------------------------------------------------------
Starting     Ending       |  constraint  slack    |  constraint  slack  |  constraint  slack  |  constraint  slack
------------------------------------------------------------------------------------------------------------------
FIFO|WR_CLK  FIFO|WR_CLK  |  10.000      -12.302  |  No paths    -      |  No paths    -      |  No paths    -    
FIFO|RD_CLK  FIFO|RD_CLK  |  10.000      -14.218  |  No paths    -      |  No paths    -      |  No paths    -    
==================================================================================================================
 Note: 'No paths' indicates there are no paths in the design for that pair of clock edges.
       'Diff grp' indicates that paths exist but the starting clock and ending clock are in different clock groups.


Interface Information 
*********************

No IO constraint found


====================================
Detailed Report for Clock: FIFO|RD_CLK
====================================


Starting Points with Worst Slack
********************************

                       Starting                                                     Arrival            
Instance               Reference       Type         Pin       Net                   Time        Slack  
                       Clock                                                                           
-------------------------------------------------------------------------------------------------------
\\FIFOBLOCK\[7\]\\     FIFO|RD_CLK     FIFO4K18     EMPTY     \\EMPTYX_I\[7\]\\     5.697       -14.218
\\FIFOBLOCK\[3\]\\     FIFO|RD_CLK     FIFO4K18     EMPTY     \\EMPTYX_I\[3\]\\     5.697       -13.855
\\FIFOBLOCK\[5\]\\     FIFO|RD_CLK     FIFO4K18     EMPTY     \\EMPTYX_I\[5\]\\     5.697       -13.855
\\FIFOBLOCK\[6\]\\     FIFO|RD_CLK     FIFO4K18     EMPTY     \\EMPTYX_I\[6\]\\     5.697       -13.855
\\FIFOBLOCK\[1\]\\     FIFO|RD_CLK     FIFO4K18     EMPTY     \\EMPTYX_I\[1\]\\     5.697       -13.492
\\FIFOBLOCK\[2\]\\     FIFO|RD_CLK     FIFO4K18     EMPTY     \\EMPTYX_I\[2\]\\     5.697       -13.492
\\FIFOBLOCK\[4\]\\     FIFO|RD_CLK     FIFO4K18     EMPTY     \\EMPTYX_I\[4\]\\     5.697       -13.492
\\FIFOBLOCK\[0\]\\     FIFO|RD_CLK     FIFO4K18     EMPTY     \\EMPTYX_I\[0\]\\     5.697       -13.128
=======================================================================================================


Ending Points with Worst Slack
******************************

                       Starting                                               Required            
Instance               Reference       Type         Pin     Net               Time         Slack  
                       Clock                                                                      
--------------------------------------------------------------------------------------------------
\\FIFOBLOCK\[0\]\\     FIFO|RD_CLK     FIFO4K18     REN     READ_ENABLE_I     5.824        -14.218
\\FIFOBLOCK\[1\]\\     FIFO|RD_CLK     FIFO4K18     REN     READ_ENABLE_I     5.824        -14.218
\\FIFOBLOCK\[2\]\\     FIFO|RD_CLK     FIFO4K18     REN     READ_ENABLE_I     5.824        -14.218
\\FIFOBLOCK\[3\]\\     FIFO|RD_CLK     FIFO4K18     REN     READ_ENABLE_I     5.824        -14.218
\\FIFOBLOCK\[4\]\\     FIFO|RD_CLK     FIFO4K18     REN     READ_ENABLE_I     5.824        -14.218
\\FIFOBLOCK\[5\]\\     FIFO|RD_CLK     FIFO4K18     REN     READ_ENABLE_I     5.824        -14.218
\\FIFOBLOCK\[6\]\\     FIFO|RD_CLK     FIFO4K18     REN     READ_ENABLE_I     5.824        -14.218
\\FIFOBLOCK\[7\]\\     FIFO|RD_CLK     FIFO4K18     REN     READ_ENABLE_I     5.824        -14.218
==================================================================================================


Worst Path Information
View Worst Path in Analyst
***********************


Path information for path number 1: 
      Requested Period:                      10.000
    - Setup time:                            4.176
    + Clock delay at ending point:           0.000 (ideal)
    = Required time:                         5.824

    - Propagation time:                      20.042
    - Clock delay at starting point:         0.000 (ideal)
    = Slack (critical) :                     -14.218

    Number of logic level(s):                5
    Starting point:                          \\FIFOBLOCK\[7\]\\ / EMPTY
    Ending point:                            \\FIFOBLOCK\[0\]\\ / REN
    The start point is clocked by            FIFO|RD_CLK [rising] on pin RCLK
    The end   point is clocked by            FIFO|RD_CLK [rising] on pin RCLK

Instance / Net                      Pin       Pin               Arrival     No. of    
Name                   Type         Name      Dir     Delay     Time        Fan Out(s)
--------------------------------------------------------------------------------------
\\FIFOBLOCK\[7\]\\     FIFO4K18     EMPTY     Out     5.697     5.697       -         
\\EMPTYX_I\[7\]\\      Net          -         -       0.773     -           1         
OR2_EMPTY_RNO_4        OR2          B         In      -         6.469       -         
OR2_EMPTY_RNO_4        OR2          Y         Out     1.236     7.705       -         
OR2_11_Y               Net          -         -       0.773     -           1         
OR2_EMPTY_RNO_0        OR2          B         In      -         8.478       -         
OR2_EMPTY_RNO_0        OR2          Y         Out     1.236     9.714       -         
OR2_19_Y               Net          -         -       0.773     -           1         
OR2_EMPTY              OR2          B         In      -         10.487      -         
OR2_EMPTY              OR2          Y         Out     1.236     11.723      -         
EMPTY_c                Net          -         -       0.927     -           2         
READ_ESTOP_GATE        NAND2        A         In      -         12.650      -         
READ_ESTOP_GATE        NAND2        Y         Out     1.174     13.824      -         
READ_ESTOP_ENABLE      Net          -         -       0.773     -           1         
READ_AND               AND2         B         In      -         14.596      -         
READ_AND               AND2         Y         Out     1.508     16.104      -         
READ_ENABLE_I          Net          -         -       3.938     -           8         
\\FIFOBLOCK\[0\]\\     FIFO4K18     REN       In      -         20.042      -         
======================================================================================
Total path delay (propagation time + setup) of 24.218 is 16.263(67.1%) logic and 7.956(32.9%) route.
Path delay compensated for clock skew. Clock skew is added to clock-to-out value, and is subtracted from setup time value


Path information for path number 2: 
      Requested Period:                      10.000
    - Setup time:                            4.176
    + Clock delay at ending point:           0.000 (ideal)
    = Required time:                         5.824

    - Propagation time:                      20.042
    - Clock delay at starting point:         0.000 (ideal)
    = Slack (critical) :                     -14.218

    Number of logic level(s):                5
    Starting point:                          \\FIFOBLOCK\[7\]\\ / EMPTY
    Ending point:                            \\FIFOBLOCK\[4\]\\ / REN
    The start point is clocked by            FIFO|RD_CLK [rising] on pin RCLK
    The end   point is clocked by            FIFO|RD_CLK [rising] on pin RCLK

Instance / Net                      Pin       Pin               Arrival     No. of    
Name                   Type         Name      Dir     Delay     Time        Fan Out(s)
--------------------------------------------------------------------------------------
\\FIFOBLOCK\[7\]\\     FIFO4K18     EMPTY     Out     5.697     5.697       -         
\\EMPTYX_I\[7\]\\      Net          -         -       0.773     -           1         
OR2_EMPTY_RNO_4        OR2          B         In      -         6.469       -         
OR2_EMPTY_RNO_4        OR2          Y         Out     1.236     7.705       -         
OR2_11_Y               Net          -         -       0.773     -           1         
OR2_EMPTY_RNO_0        OR2          B         In      -         8.478       -         
OR2_EMPTY_RNO_0        OR2          Y         Out     1.236     9.714       -         
OR2_19_Y               Net          -         -       0.773     -           1         
OR2_EMPTY              OR2          B         In      -         10.487      -         
OR2_EMPTY              OR2          Y         Out     1.236     11.723      -         
EMPTY_c                Net          -         -       0.927     -           2         
READ_ESTOP_GATE        NAND2        A         In      -         12.650      -         
READ_ESTOP_GATE        NAND2        Y         Out     1.174     13.824      -         
READ_ESTOP_ENABLE      Net          -         -       0.773     -           1         
READ_AND               AND2         B         In      -         14.596      -         
READ_AND               AND2         Y         Out     1.508     16.104      -         
READ_ENABLE_I          Net          -         -       3.938     -           8         
\\FIFOBLOCK\[4\]\\     FIFO4K18     REN       In      -         20.042      -         
======================================================================================
Total path delay (propagation time + setup) of 24.218 is 16.263(67.1%) logic and 7.956(32.9%) route.
Path delay compensated for clock skew. Clock skew is added to clock-to-out value, and is subtracted from setup time value


Path information for path number 3: 
      Requested Period:                      10.000
    - Setup time:                            4.176
    + Clock delay at ending point:           0.000 (ideal)
    = Required time:                         5.824

    - Propagation time:                      20.042
    - Clock delay at starting point:         0.000 (ideal)
    = Slack (critical) :                     -14.218

    Number of logic level(s):                5
    Starting point:                          \\FIFOBLOCK\[7\]\\ / EMPTY
    Ending point:                            \\FIFOBLOCK\[5\]\\ / REN
    The start point is clocked by            FIFO|RD_CLK [rising] on pin RCLK
    The end   point is clocked by            FIFO|RD_CLK [rising] on pin RCLK

Instance / Net                      Pin       Pin               Arrival     No. of    
Name                   Type         Name      Dir     Delay     Time        Fan Out(s)
--------------------------------------------------------------------------------------
\\FIFOBLOCK\[7\]\\     FIFO4K18     EMPTY     Out     5.697     5.697       -         
\\EMPTYX_I\[7\]\\      Net          -         -       0.773     -           1         
OR2_EMPTY_RNO_4        OR2          B         In      -         6.469       -         
OR2_EMPTY_RNO_4        OR2          Y         Out     1.236     7.705       -         
OR2_11_Y               Net          -         -       0.773     -           1         
OR2_EMPTY_RNO_0        OR2          B         In      -         8.478       -         
OR2_EMPTY_RNO_0        OR2          Y         Out     1.236     9.714       -         
OR2_19_Y               Net          -         -       0.773     -           1         
OR2_EMPTY              OR2          B         In      -         10.487      -         
OR2_EMPTY              OR2          Y         Out     1.236     11.723      -         
EMPTY_c                Net          -         -       0.927     -           2         
READ_ESTOP_GATE        NAND2        A         In      -         12.650      -         
READ_ESTOP_GATE        NAND2        Y         Out     1.174     13.824      -         
READ_ESTOP_ENABLE      Net          -         -       0.773     -           1         
READ_AND               AND2         B         In      -         14.596      -         
READ_AND               AND2         Y         Out     1.508     16.104      -         
READ_ENABLE_I          Net          -         -       3.938     -           8         
\\FIFOBLOCK\[5\]\\     FIFO4K18     REN       In      -         20.042      -         
======================================================================================
Total path delay (propagation time + setup) of 24.218 is 16.263(67.1%) logic and 7.956(32.9%) route.
Path delay compensated for clock skew. Clock skew is added to clock-to-out value, and is subtracted from setup time value


Path information for path number 4: 
      Requested Period:                      10.000
    - Setup time:                            4.176
    + Clock delay at ending point:           0.000 (ideal)
    = Required time:                         5.824

    - Propagation time:                      20.042
    - Clock delay at starting point:         0.000 (ideal)
    = Slack (critical) :                     -14.218

    Number of logic level(s):                5
    Starting point:                          \\FIFOBLOCK\[7\]\\ / EMPTY
    Ending point:                            \\FIFOBLOCK\[2\]\\ / REN
    The start point is clocked by            FIFO|RD_CLK [rising] on pin RCLK
    The end   point is clocked by            FIFO|RD_CLK [rising] on pin RCLK

Instance / Net                      Pin       Pin               Arrival     No. of    
Name                   Type         Name      Dir     Delay     Time        Fan Out(s)
--------------------------------------------------------------------------------------
\\FIFOBLOCK\[7\]\\     FIFO4K18     EMPTY     Out     5.697     5.697       -         
\\EMPTYX_I\[7\]\\      Net          -         -       0.773     -           1         
OR2_EMPTY_RNO_4        OR2          B         In      -         6.469       -         
OR2_EMPTY_RNO_4        OR2          Y         Out     1.236     7.705       -         
OR2_11_Y               Net          -         -       0.773     -           1         
OR2_EMPTY_RNO_0        OR2          B         In      -         8.478       -         
OR2_EMPTY_RNO_0        OR2          Y         Out     1.236     9.714       -         
OR2_19_Y               Net          -         -       0.773     -           1         
OR2_EMPTY              OR2          B         In      -         10.487      -         
OR2_EMPTY              OR2          Y         Out     1.236     11.723      -         
EMPTY_c                Net          -         -       0.927     -           2         
READ_ESTOP_GATE        NAND2        A         In      -         12.650      -         
READ_ESTOP_GATE        NAND2        Y         Out     1.174     13.824      -         
READ_ESTOP_ENABLE      Net          -         -       0.773     -           1         
READ_AND               AND2         B         In      -         14.596      -         
READ_AND               AND2         Y         Out     1.508     16.104      -         
READ_ENABLE_I          Net          -         -       3.938     -           8         
\\FIFOBLOCK\[2\]\\     FIFO4K18     REN       In      -         20.042      -         
======================================================================================
Total path delay (propagation time + setup) of 24.218 is 16.263(67.1%) logic and 7.956(32.9%) route.
Path delay compensated for clock skew. Clock skew is added to clock-to-out value, and is subtracted from setup time value


Path information for path number 5: 
      Requested Period:                      10.000
    - Setup time:                            4.176
    + Clock delay at ending point:           0.000 (ideal)
    = Required time:                         5.824

    - Propagation time:                      20.042
    - Clock delay at starting point:         0.000 (ideal)
    = Slack (critical) :                     -14.218

    Number of logic level(s):                5
    Starting point:                          \\FIFOBLOCK\[7\]\\ / EMPTY
    Ending point:                            \\FIFOBLOCK\[7\]\\ / REN
    The start point is clocked by            FIFO|RD_CLK [rising] on pin RCLK
    The end   point is clocked by            FIFO|RD_CLK [rising] on pin RCLK

Instance / Net                      Pin       Pin               Arrival     No. of    
Name                   Type         Name      Dir     Delay     Time        Fan Out(s)
--------------------------------------------------------------------------------------
\\FIFOBLOCK\[7\]\\     FIFO4K18     EMPTY     Out     5.697     5.697       -         
\\EMPTYX_I\[7\]\\      Net          -         -       0.773     -           1         
OR2_EMPTY_RNO_4        OR2          B         In      -         6.469       -         
OR2_EMPTY_RNO_4        OR2          Y         Out     1.236     7.705       -         
OR2_11_Y               Net          -         -       0.773     -           1         
OR2_EMPTY_RNO_0        OR2          B         In      -         8.478       -         
OR2_EMPTY_RNO_0        OR2          Y         Out     1.236     9.714       -         
OR2_19_Y               Net          -         -       0.773     -           1         
OR2_EMPTY              OR2          B         In      -         10.487      -         
OR2_EMPTY              OR2          Y         Out     1.236     11.723      -         
EMPTY_c                Net          -         -       0.927     -           2         
READ_ESTOP_GATE        NAND2        A         In      -         12.650      -         
READ_ESTOP_GATE        NAND2        Y         Out     1.174     13.824      -         
READ_ESTOP_ENABLE      Net          -         -       0.773     -           1         
READ_AND               AND2         B         In      -         14.596      -         
READ_AND               AND2         Y         Out     1.508     16.104      -         
READ_ENABLE_I          Net          -         -       3.938     -           8         
\\FIFOBLOCK\[7\]\\     FIFO4K18     REN       In      -         20.042      -         
======================================================================================
Total path delay (propagation time + setup) of 24.218 is 16.263(67.1%) logic and 7.956(32.9%) route.
Path delay compensated for clock skew. Clock skew is added to clock-to-out value, and is subtracted from setup time value


====================================
Detailed Report for Clock: FIFO|WR_CLK
====================================


Starting Points with Worst Slack
********************************

                       Starting                                                   Arrival            
Instance               Reference       Type         Pin      Net                  Time        Slack  
                       Clock                                                                         
-----------------------------------------------------------------------------------------------------
\\FIFOBLOCK\[7\]\\     FIFO|WR_CLK     FIFO4K18     FULL     \\FULLX_I\[7\]\\     4.360       -12.302
\\FIFOBLOCK\[3\]\\     FIFO|WR_CLK     FIFO4K18     FULL     \\FULLX_I\[3\]\\     4.360       -11.967
\\FIFOBLOCK\[5\]\\     FIFO|WR_CLK     FIFO4K18     FULL     \\FULLX_I\[5\]\\     4.360       -11.967
\\FIFOBLOCK\[6\]\\     FIFO|WR_CLK     FIFO4K18     FULL     \\FULLX_I\[6\]\\     4.360       -11.967
\\FIFOBLOCK\[1\]\\     FIFO|WR_CLK     FIFO4K18     FULL     \\FULLX_I\[1\]\\     4.360       -11.633
\\FIFOBLOCK\[2\]\\     FIFO|WR_CLK     FIFO4K18     FULL     \\FULLX_I\[2\]\\     4.360       -11.633
\\FIFOBLOCK\[4\]\\     FIFO|WR_CLK     FIFO4K18     FULL     \\FULLX_I\[4\]\\     4.360       -11.633
\\FIFOBLOCK\[0\]\\     FIFO|WR_CLK     FIFO4K18     FULL     \\FULLX_I\[0\]\\     4.360       -11.299
=====================================================================================================


Ending Points with Worst Slack
******************************

                       Starting                                                Required            
Instance               Reference       Type         Pin     Net                Time         Slack  
                       Clock                                                                       
---------------------------------------------------------------------------------------------------
\\FIFOBLOCK\[0\]\\     FIFO|WR_CLK     FIFO4K18     WEN     WRITE_ENABLE_I     7.202        -12.302
\\FIFOBLOCK\[1\]\\     FIFO|WR_CLK     FIFO4K18     WEN     WRITE_ENABLE_I     7.202        -12.302
\\FIFOBLOCK\[2\]\\     FIFO|WR_CLK     FIFO4K18     WEN     WRITE_ENABLE_I     7.202        -12.302
\\FIFOBLOCK\[3\]\\     FIFO|WR_CLK     FIFO4K18     WEN     WRITE_ENABLE_I     7.202        -12.302
\\FIFOBLOCK\[4\]\\     FIFO|WR_CLK     FIFO4K18     WEN     WRITE_ENABLE_I     7.202        -12.302
\\FIFOBLOCK\[5\]\\     FIFO|WR_CLK     FIFO4K18     WEN     WRITE_ENABLE_I     7.202        -12.302
\\FIFOBLOCK\[6\]\\     FIFO|WR_CLK     FIFO4K18     WEN     WRITE_ENABLE_I     7.202        -12.302
\\FIFOBLOCK\[7\]\\     FIFO|WR_CLK     FIFO4K18     WEN     WRITE_ENABLE_I     7.202        -12.302
===================================================================================================


Worst Path Information
View Worst Path in Analyst
***********************


Path information for path number 1: 
      Requested Period:                      10.000
    - Setup time:                            2.798
    + Clock delay at ending point:           0.000 (ideal)
    = Required time:                         7.202

    - Propagation time:                      19.503
    - Clock delay at starting point:         0.000 (ideal)
    = Slack (non-critical) :                 -12.301

    Number of logic level(s):                5
    Starting point:                          \\FIFOBLOCK\[7\]\\ / FULL
    Ending point:                            \\FIFOBLOCK\[0\]\\ / WEN
    The start point is clocked by            FIFO|WR_CLK [rising] on pin WCLK
    The end   point is clocked by            FIFO|WR_CLK [rising] on pin WCLK

Instance / Net                      Pin      Pin               Arrival     No. of    
Name                   Type         Name     Dir     Delay     Time        Fan Out(s)
-------------------------------------------------------------------------------------
\\FIFOBLOCK\[7\]\\     FIFO4K18     FULL     Out     4.360     4.360       -         
\\FULLX_I\[7\]\\       Net          -        -       0.773     -           1         
OR2_7                  OR2          B        In      -         5.133       -         
OR2_7                  OR2          Y        Out     1.554     6.686       -         
OR2_7_Y                Net          -        -       0.773     -           1         
OR2_5                  OR2          B        In      -         7.459       -         
OR2_5                  OR2          Y        Out     1.554     9.012       -         
OR2_5_Y                Net          -        -       0.773     -           1         
OR2_FULL               OR2          B        In      -         9.785       -         
OR2_FULL               OR2          Y        Out     1.554     11.339      -         
FULL_c                 Net          -        -       0.927     -           2         
WRITE_FSTOP_GATE       NAND2        A        In      -         12.266      -         
WRITE_FSTOP_GATE       NAND2        Y        Out     1.236     13.502      -         
WRITE_FSTOP_ENABLE     Net          -        -       0.773     -           1         
WRITE_AND              NAND2A       B        In      -         14.275      -         
WRITE_AND              NAND2A       Y        Out     1.290     15.565      -         
WRITE_ENABLE_I         Net          -        -       3.938     -           8         
\\FIFOBLOCK\[0\]\\     FIFO4K18     WEN      In      -         19.503      -         
=====================================================================================
Total path delay (propagation time + setup) of 22.301 is 14.346(64.3%) logic and 7.956(35.7%) route.
Path delay compensated for clock skew. Clock skew is added to clock-to-out value, and is subtracted from setup time value


Path information for path number 2: 
      Requested Period:                      10.000
    - Setup time:                            2.798
    + Clock delay at ending point:           0.000 (ideal)
    = Required time:                         7.202

    - Propagation time:                      19.503
    - Clock delay at starting point:         0.000 (ideal)
    = Slack (non-critical) :                 -12.301

    Number of logic level(s):                5
    Starting point:                          \\FIFOBLOCK\[7\]\\ / FULL
    Ending point:                            \\FIFOBLOCK\[4\]\\ / WEN
    The start point is clocked by            FIFO|WR_CLK [rising] on pin WCLK
    The end   point is clocked by            FIFO|WR_CLK [rising] on pin WCLK

Instance / Net                      Pin      Pin               Arrival     No. of    
Name                   Type         Name     Dir     Delay     Time        Fan Out(s)
-------------------------------------------------------------------------------------
\\FIFOBLOCK\[7\]\\     FIFO4K18     FULL     Out     4.360     4.360       -         
\\FULLX_I\[7\]\\       Net          -        -       0.773     -           1         
OR2_7                  OR2          B        In      -         5.133       -         
OR2_7                  OR2          Y        Out     1.554     6.686       -         
OR2_7_Y                Net          -        -       0.773     -           1         
OR2_5                  OR2          B        In      -         7.459       -         
OR2_5                  OR2          Y        Out     1.554     9.012       -         
OR2_5_Y                Net          -        -       0.773     -           1         
OR2_FULL               OR2          B        In      -         9.785       -         
OR2_FULL               OR2          Y        Out     1.554     11.339      -         
FULL_c                 Net          -        -       0.927     -           2         
WRITE_FSTOP_GATE       NAND2        A        In      -         12.266      -         
WRITE_FSTOP_GATE       NAND2        Y        Out     1.236     13.502      -         
WRITE_FSTOP_ENABLE     Net          -        -       0.773     -           1         
WRITE_AND              NAND2A       B        In      -         14.275      -         
WRITE_AND              NAND2A       Y        Out     1.290     15.565      -         
WRITE_ENABLE_I         Net          -        -       3.938     -           8         
\\FIFOBLOCK\[4\]\\     FIFO4K18     WEN      In      -         19.503      -         
=====================================================================================
Total path delay (propagation time + setup) of 22.301 is 14.346(64.3%) logic and 7.956(35.7%) route.
Path delay compensated for clock skew. Clock skew is added to clock-to-out value, and is subtracted from setup time value


Path information for path number 3: 
      Requested Period:                      10.000
    - Setup time:                            2.798
    + Clock delay at ending point:           0.000 (ideal)
    = Required time:                         7.202

    - Propagation time:                      19.503
    - Clock delay at starting point:         0.000 (ideal)
    = Slack (non-critical) :                 -12.301

    Number of logic level(s):                5
    Starting point:                          \\FIFOBLOCK\[7\]\\ / FULL
    Ending point:                            \\FIFOBLOCK\[5\]\\ / WEN
    The start point is clocked by            FIFO|WR_CLK [rising] on pin WCLK
    The end   point is clocked by            FIFO|WR_CLK [rising] on pin WCLK

Instance / Net                      Pin      Pin               Arrival     No. of    
Name                   Type         Name     Dir     Delay     Time        Fan Out(s)
-------------------------------------------------------------------------------------
\\FIFOBLOCK\[7\]\\     FIFO4K18     FULL     Out     4.360     4.360       -         
\\FULLX_I\[7\]\\       Net          -        -       0.773     -           1         
OR2_7                  OR2          B        In      -         5.133       -         
OR2_7                  OR2          Y        Out     1.554     6.686       -         
OR2_7_Y                Net          -        -       0.773     -           1         
OR2_5                  OR2          B        In      -         7.459       -         
OR2_5                  OR2          Y        Out     1.554     9.012       -         
OR2_5_Y                Net          -        -       0.773     -           1         
OR2_FULL               OR2          B        In      -         9.785       -         
OR2_FULL               OR2          Y        Out     1.554     11.339      -         
FULL_c                 Net          -        -       0.927     -           2         
WRITE_FSTOP_GATE       NAND2        A        In      -         12.266      -         
WRITE_FSTOP_GATE       NAND2        Y        Out     1.236     13.502      -         
WRITE_FSTOP_ENABLE     Net          -        -       0.773     -           1         
WRITE_AND              NAND2A       B        In      -         14.275      -         
WRITE_AND              NAND2A       Y        Out     1.290     15.565      -         
WRITE_ENABLE_I         Net          -        -       3.938     -           8         
\\FIFOBLOCK\[5\]\\     FIFO4K18     WEN      In      -         19.503      -         
=====================================================================================
Total path delay (propagation time + setup) of 22.301 is 14.346(64.3%) logic and 7.956(35.7%) route.
Path delay compensated for clock skew. Clock skew is added to clock-to-out value, and is subtracted from setup time value


Path information for path number 4: 
      Requested Period:                      10.000
    - Setup time:                            2.798
    + Clock delay at ending point:           0.000 (ideal)
    = Required time:                         7.202

    - Propagation time:                      19.503
    - Clock delay at starting point:         0.000 (ideal)
    = Slack (non-critical) :                 -12.301

    Number of logic level(s):                5
    Starting point:                          \\FIFOBLOCK\[7\]\\ / FULL
    Ending point:                            \\FIFOBLOCK\[2\]\\ / WEN
    The start point is clocked by            FIFO|WR_CLK [rising] on pin WCLK
    The end   point is clocked by            FIFO|WR_CLK [rising] on pin WCLK

Instance / Net                      Pin      Pin               Arrival     No. of    
Name                   Type         Name     Dir     Delay     Time        Fan Out(s)
-------------------------------------------------------------------------------------
\\FIFOBLOCK\[7\]\\     FIFO4K18     FULL     Out     4.360     4.360       -         
\\FULLX_I\[7\]\\       Net          -        -       0.773     -           1         
OR2_7                  OR2          B        In      -         5.133       -         
OR2_7                  OR2          Y        Out     1.554     6.686       -         
OR2_7_Y                Net          -        -       0.773     -           1         
OR2_5                  OR2          B        In      -         7.459       -         
OR2_5                  OR2          Y        Out     1.554     9.012       -         
OR2_5_Y                Net          -        -       0.773     -           1         
OR2_FULL               OR2          B        In      -         9.785       -         
OR2_FULL               OR2          Y        Out     1.554     11.339      -         
FULL_c                 Net          -        -       0.927     -           2         
WRITE_FSTOP_GATE       NAND2        A        In      -         12.266      -         
WRITE_FSTOP_GATE       NAND2        Y        Out     1.236     13.502      -         
WRITE_FSTOP_ENABLE     Net          -        -       0.773     -           1         
WRITE_AND              NAND2A       B        In      -         14.275      -         
WRITE_AND              NAND2A       Y        Out     1.290     15.565      -         
WRITE_ENABLE_I         Net          -        -       3.938     -           8         
\\FIFOBLOCK\[2\]\\     FIFO4K18     WEN      In      -         19.503      -         
=====================================================================================
Total path delay (propagation time + setup) of 22.301 is 14.346(64.3%) logic and 7.956(35.7%) route.
Path delay compensated for clock skew. Clock skew is added to clock-to-out value, and is subtracted from setup time value


Path information for path number 5: 
      Requested Period:                      10.000
    - Setup time:                            2.798
    + Clock delay at ending point:           0.000 (ideal)
    = Required time:                         7.202

    - Propagation time:                      19.503
    - Clock delay at starting point:         0.000 (ideal)
    = Slack (non-critical) :                 -12.301

    Number of logic level(s):                5
    Starting point:                          \\FIFOBLOCK\[7\]\\ / FULL
    Ending point:                            \\FIFOBLOCK\[7\]\\ / WEN
    The start point is clocked by            FIFO|WR_CLK [rising] on pin WCLK
    The end   point is clocked by            FIFO|WR_CLK [rising] on pin WCLK

Instance / Net                      Pin      Pin               Arrival     No. of    
Name                   Type         Name     Dir     Delay     Time        Fan Out(s)
-------------------------------------------------------------------------------------
\\FIFOBLOCK\[7\]\\     FIFO4K18     FULL     Out     4.360     4.360       -         
\\FULLX_I\[7\]\\       Net          -        -       0.773     -           1         
OR2_7                  OR2          B        In      -         5.133       -         
OR2_7                  OR2          Y        Out     1.554     6.686       -         
OR2_7_Y                Net          -        -       0.773     -           1         
OR2_5                  OR2          B        In      -         7.459       -         
OR2_5                  OR2          Y        Out     1.554     9.012       -         
OR2_5_Y                Net          -        -       0.773     -           1         
OR2_FULL               OR2          B        In      -         9.785       -         
OR2_FULL               OR2          Y        Out     1.554     11.339      -         
FULL_c                 Net          -        -       0.927     -           2         
WRITE_FSTOP_GATE       NAND2        A        In      -         12.266      -         
WRITE_FSTOP_GATE       NAND2        Y        Out     1.236     13.502      -         
WRITE_FSTOP_ENABLE     Net          -        -       0.773     -           1         
WRITE_AND              NAND2A       B        In      -         14.275      -         
WRITE_AND              NAND2A       Y        Out     1.290     15.565      -         
WRITE_ENABLE_I         Net          -        -       3.938     -           8         
\\FIFOBLOCK\[7\]\\     FIFO4K18     WEN      In      -         19.503      -         
=====================================================================================
Total path delay (propagation time + setup) of 22.301 is 14.346(64.3%) logic and 7.956(35.7%) route.
Path delay compensated for clock skew. Clock skew is added to clock-to-out value, and is subtracted from setup time value


##### END OF TIMING REPORT #####]

--------------------------------------------------------------------------------
Target Part: AGL250V2_VQFP100_STD
Report for cell FIFO.def_arch
  Core Cell usage:
              cell count     area count*area
              AND2     1      1.0        1.0
               GND     1      0.0        0.0
               INV     2      1.0        2.0
             NAND2     2      1.0        2.0
            NAND2A     1      1.0        1.0
               OR2    28      1.0       28.0
               VCC     1      0.0        0.0


          FIFO4K18     8      0.0        0.0
                   -----          ----------
             TOTAL    44                34.0


  IO Cell usage:
              cell count
            CLKBUF     2
             INBUF    11
            OUTBUF    12
                   -----
             TOTAL    25


Core Cells         : 34 of 6144 (1%)
IO Cells           : 25

  RAM/ROM Usage Summary
Block Rams : 8 of 8 (100%)

Mapper successful!

At Mapper Exit (Real Time elapsed 0h:00m:00s; CPU Time elapsed 0h:00m:00s; Memory used current: 45MB peak: 109MB)

Process took 0h:00m:01s realtime, 0h:00m:01s cputime
# Tue Aug 05 17:25:05 2014

###########################################################]
``` ````
