# Supplementary material for: Implications for a Wireless, External Device System to Study Electrocorticography
Source: Sensors (Basel). 2017 Apr 4;17(4):761. doi: 10.3390/s17040761 (PMC5421721; doi:10.3390/s17040761)
Supplement: Supplementary file 1 [file sensors-17-00761-s001.zip › Supplement/RHA_FPGA_Firmware/IGLOO_RHA_05092014.zip!/IGLOO_RHA/synthesis/syntmp/IGLOO_TOP_srr.htm]

 ```` ```
#Build: Synplify Pro I-2013.09M-SP1 , Build 034R, Jan 17 2014
#install: C:\Microsemi\Libero_v11.3\Synopsys\synplify_I201309MSP1
#OS: Windows 7 6.1
#Hostname: ITP-PC

#Implementation: synthesis

$ Start of Compile
#Fri Sep 05 17:35:13 2014

Synopsys VHDL Compiler, version comp201309rcp1, Build 078R, built Jan 14 2014
@N: :  | Running in 64-bit mode 
Copyright (C) 1994-2013 Synopsys, Inc. This software and the associated documentation are proprietary to Synopsys, Inc. This software may only be used in accordance with the terms and conditions of a written license agreement with Synopsys, Inc.  All other use or distribution of the software is strictly prohibited.

@N:CD720 : std.vhd(123) | Setting time resolution to ns
@N: : IGLOO_TOP.vhd(23) | Top entity is set to IGLOO_TOP.
File C:\Microsemi\Libero_v11.3\Synopsys\synplify_I201309MSP1\lib\proasic\igloo.vhd changed - recompiling
File C:\Users\ITP\Desktop\IGLOO\IGLOO_RHA\hdl\Zarlink_SPI_Module.vhd changed - recompiling
File C:\Users\ITP\Desktop\IGLOO\IGLOO_RHA\hdl\ZARLINK_CONNECT_INIT.vhd changed - recompiling
File C:\Users\ITP\Desktop\IGLOO\IGLOO_RHA\hdl\ORGANIZER.vhd changed - recompiling
File C:\Users\ITP\Desktop\IGLOO\IGLOO_RHA\hdl\COMMAND_RECEIVER.vhd changed - recompiling
File C:\Users\ITP\Desktop\IGLOO\IGLOO_RHA\hdl\RHA_TO_ZL_CONVERTER.vhd changed - recompiling
File C:\Users\ITP\Desktop\IGLOO\IGLOO_RHA\hdl\RHA_ERROR_WATCHDOG.vhd changed - recompiling
File C:\Users\ITP\Desktop\IGLOO\IGLOO_RHA\hdl\RHA_TEST_MODULATOR.vhd changed - recompiling
File C:\Users\ITP\Desktop\IGLOO\IGLOO_RHA\hdl\CONTROL_NEXUS.vhd changed - recompiling
File C:\Users\ITP\Desktop\IGLOO\IGLOO_RHA\hdl\RHA_ARRAY.vhd changed - recompiling
File C:\Users\ITP\Desktop\IGLOO\IGLOO_RHA\hdl\RHA_TESTMODULE.vhd changed - recompiling
File C:\Users\ITP\Desktop\IGLOO\IGLOO_RHA\hdl\DATA_ACQUISITION_BLOCK.vhd changed - recompiling
File C:\Users\ITP\Desktop\IGLOO\IGLOO_RHA\hdl\IMPLANT_TOP.vhd changed - recompiling
File C:\Users\ITP\Desktop\IGLOO\IGLOO_RHA\hdl\IGLOO_TOP.vhd changed - recompiling
VHDL syntax check successful!
@N:CD630 : IGLOO_TOP.vhd(23) | Synthesizing work.igloo_top.igloo_top_arch 
@N:CD630 : IMPLANT_TOP.vhd(26) | Synthesizing work.implant_top.behavioral 
@N:CD364 : IMPLANT_TOP.vhd(206) | Removed redundant assignment
@N:CD364 : IMPLANT_TOP.vhd(297) | Removed redundant assignment
@N:CD630 : DATA_ACQUISITION_BLOCK.vhd(25) | Synthesizing work.data_acquisition_block.behavioral 
@W:CD638 : DATA_ACQUISITION_BLOCK.vhd(78) | Signal rha_adc_clk_test is undriven 
@N:CD630 : RHA_ERROR_WATCHDOG.vhd(25) | Synthesizing work.rha_error_watchdog.behavioral 
@N:CD364 : RHA_ERROR_WATCHDOG.vhd(93) | Removed redundant assignment
@N:CD364 : RHA_ERROR_WATCHDOG.vhd(94) | Removed redundant assignment
@N:CD364 : RHA_ERROR_WATCHDOG.vhd(95) | Removed redundant assignment
@N:CD364 : RHA_ERROR_WATCHDOG.vhd(96) | Removed redundant assignment
@N:CD364 : RHA_ERROR_WATCHDOG.vhd(97) | Removed redundant assignment
@N:CD364 : RHA_ERROR_WATCHDOG.vhd(98) | Removed redundant assignment
@N:CD364 : RHA_ERROR_WATCHDOG.vhd(99) | Removed redundant assignment
Post processing for work.rha_error_watchdog.behavioral
@N:CD630 : RHA_TESTMODULE.vhd(26) | Synthesizing work.rha_testmodule.behavioral 
@N:CD630 : RHA_TEST_MODULATOR.vhd(25) | Synthesizing work.rha_test_modulator.behavioral 
@N:CD232 : RHA_TEST_MODULATOR.vhd(39) | Using gray code encoding for type state_type
@N:CD364 : RHA_TEST_MODULATOR.vhd(134) | Removed redundant assignment
@N:CD364 : RHA_TEST_MODULATOR.vhd(135) | Removed redundant assignment
@W:CD604 : RHA_TEST_MODULATOR.vhd(308) | OTHERS clause is not synthesized 
@N:CD364 : RHA_TEST_MODULATOR.vhd(346) | Removed redundant assignment
@N:CD364 : RHA_TEST_MODULATOR.vhd(354) | Removed redundant assignment
@N:CD364 : RHA_TEST_MODULATOR.vhd(358) | Removed redundant assignment
@N:CD364 : RHA_TEST_MODULATOR.vhd(361) | Removed redundant assignment
@N:CD364 : RHA_TEST_MODULATOR.vhd(362) | Removed redundant assignment
@W:CD604 : RHA_TEST_MODULATOR.vhd(420) | OTHERS clause is not synthesized 
Post processing for work.rha_test_modulator.behavioral
@W:CL111 : RHA_TEST_MODULATOR.vhd(337) | All reachable assignments to TESTVALUE(2) assign '0'; register removed by optimization
@W:CL190 : RHA_TEST_MODULATOR.vhd(123) | Optimizing register bit TESTVALUE_INTERNAL(2) to a constant 0
@W:CL260 : RHA_TEST_MODULATOR.vhd(123) | Pruning register bit 2 of TESTVALUE_INTERNAL(24 downto 0)  
Post processing for work.rha_testmodule.behavioral
@N:CD630 : RHA_TO_ZL_CONVERTER.vhd(26) | Synthesizing work.rha_to_zl_converter.behavioral 
@N:CD232 : RHA_TO_ZL_CONVERTER.vhd(111) | Using gray code encoding for type state_type
@N:CD364 : RHA_TO_ZL_CONVERTER.vhd(261) | Removed redundant assignment
@N:CD364 : RHA_TO_ZL_CONVERTER.vhd(262) | Removed redundant assignment
@N:CD364 : RHA_TO_ZL_CONVERTER.vhd(295) | Removed redundant assignment
@N:CD364 : RHA_TO_ZL_CONVERTER.vhd(324) | Removed redundant assignment
@N:CD364 : RHA_TO_ZL_CONVERTER.vhd(325) | Removed redundant assignment
@N:CD364 : RHA_TO_ZL_CONVERTER.vhd(327) | Removed redundant assignment
@N:CD364 : RHA_TO_ZL_CONVERTER.vhd(328) | Removed redundant assignment
@N:CD364 : RHA_TO_ZL_CONVERTER.vhd(329) | Removed redundant assignment
@N:CD364 : RHA_TO_ZL_CONVERTER.vhd(330) | Removed redundant assignment
@N:CD364 : RHA_TO_ZL_CONVERTER.vhd(332) | Removed redundant assignment
@N:CD364 : RHA_TO_ZL_CONVERTER.vhd(344) | Removed redundant assignment
@N:CD364 : RHA_TO_ZL_CONVERTER.vhd(580) | Removed redundant assignment
@W:CD604 : RHA_TO_ZL_CONVERTER.vhd(615) | OTHERS clause is not synthesized 
Post processing for work.rha_to_zl_converter.behavioral
@W:CL169 : RHA_TO_ZL_CONVERTER.vhd(104) | Pruning register ReadRequest_INTERNAL_LAST4_2  
@W:CL169 : RHA_TO_ZL_CONVERTER.vhd(103) | Pruning register ReadRequest_INTERNAL_LAST3_2  
@W:CL169 : RHA_TO_ZL_CONVERTER.vhd(102) | Pruning register ReadRequest_INTERNAL_LAST2_2  
@A:CL282 : RHA_TO_ZL_CONVERTER.vhd(290) | Feedback mux created for signal NewBlock_LAST -- possible set/reset assignment for signal missing. Specifying a reset value will improve timing and area.
@N:CD630 : RHA_ARRAY.vhd(30) | Synthesizing work.rha_array.behavioral 
@N:CD364 : RHA_ARRAY.vhd(456) | Removed redundant assignment
@N:CD364 : RHA_ARRAY.vhd(457) | Removed redundant assignment
@N:CD364 : RHA_ARRAY.vhd(458) | Removed redundant assignment
@N:CD364 : RHA_ARRAY.vhd(459) | Removed redundant assignment
@N:CD364 : RHA_ARRAY.vhd(460) | Removed redundant assignment
@N:CD364 : RHA_ARRAY.vhd(465) | Removed redundant assignment
@N:CD364 : RHA_ARRAY.vhd(467) | Removed redundant assignment
@N:CD364 : RHA_ARRAY.vhd(469) | Removed redundant assignment
@N:CD364 : RHA_ARRAY.vhd(470) | Removed redundant assignment
@N:CD364 : RHA_ARRAY.vhd(471) | Removed redundant assignment
@N:CD364 : RHA_ARRAY.vhd(472) | Removed redundant assignment
@N:CD364 : RHA_ARRAY.vhd(473) | Removed redundant assignment
@N:CD364 : RHA_ARRAY.vhd(474) | Removed redundant assignment
@N:CD364 : RHA_ARRAY.vhd(475) | Removed redundant assignment
@N:CD364 : RHA_ARRAY.vhd(476) | Removed redundant assignment
@N:CD364 : RHA_ARRAY.vhd(597) | Removed redundant assignment
@N:CD364 : RHA_ARRAY.vhd(598) | Removed redundant assignment
@W:CD638 : RHA_ARRAY.vhd(153) | Signal channel_number is undriven 
@N:CD630 : RHA_DATA_CHECKER.vhd(34) | Synthesizing work.rha_data_checker.behavioral 
Post processing for work.rha_data_checker.behavioral
@N:CD630 : RHA_DATA_COLLECTOR.vhd(28) | Synthesizing work.rha_data_collector.behavioral 
@N:CD233 : RHA_DATA_COLLECTOR.vhd(54) | Using sequential encoding for type state_type
@N:CD364 : RHA_DATA_COLLECTOR.vhd(118) | Removed redundant assignment
@N:CD364 : RHA_DATA_COLLECTOR.vhd(120) | Removed redundant assignment
@N:CD364 : RHA_DATA_COLLECTOR.vhd(128) | Removed redundant assignment
@N:CD364 : RHA_DATA_COLLECTOR.vhd(131) | Removed redundant assignment
@W:CD604 : RHA_DATA_COLLECTOR.vhd(187) | OTHERS clause is not synthesized 
Post processing for work.rha_data_collector.behavioral
@W:CL265 : RHA_DATA_COLLECTOR.vhd(99) | Pruning bit 24 of DATAOUT_DATA_INTERNAL_5(24 downto 0) -- not in use ... 
Post processing for work.rha_array.behavioral
@W:CL190 : RHA_ARRAY.vhd(149) | Optimizing register bit AddressCheckInfo(3) to a constant 0
@W:CL260 : RHA_ARRAY.vhd(149) | Pruning register bit 3 of AddressCheckInfo(79 downto 0)  
Post processing for work.data_acquisition_block.behavioral
@N:CD630 : CONTROL_NEXUS.vhd(28) | Synthesizing work.control_nexus.behavioral 
@W:CD638 : CONTROL_NEXUS.vhd(109) | Signal zarlink_fifo_wait is undriven 
@W:CD638 : CONTROL_NEXUS.vhd(114) | Signal fifo_in_readenable_internal is undriven 
@N:CD630 : ORGANIZER.vhd(26) | Synthesizing work.organizer.behavioral 
@N:CD231 : ORGANIZER.vhd(85) | Using onehot encoding for type state_type (sm_reset="100000000000000")
@N:CD364 : ORGANIZER.vhd(138) | Removed redundant assignment
Post processing for work.organizer.behavioral
@N:CD630 : ZARLINK_CONNECT_INIT.vhd(25) | Synthesizing work.zarlink_connect_init.behavioral 
@N:CD232 : ZARLINK_CONNECT_INIT.vhd(81) | Using gray code encoding for type state_type
@N:CD364 : ZARLINK_CONNECT_INIT.vhd(231) | Removed redundant assignment
@N:CD364 : ZARLINK_CONNECT_INIT.vhd(240) | Removed redundant assignment
@N:CD364 : ZARLINK_CONNECT_INIT.vhd(242) | Removed redundant assignment
@N:CD364 : ZARLINK_CONNECT_INIT.vhd(243) | Removed redundant assignment
@N:CD364 : ZARLINK_CONNECT_INIT.vhd(244) | Removed redundant assignment
@N:CD364 : ZARLINK_CONNECT_INIT.vhd(245) | Removed redundant assignment
@N:CD364 : ZARLINK_CONNECT_INIT.vhd(247) | Removed redundant assignment
@N:CD364 : ZARLINK_CONNECT_INIT.vhd(248) | Removed redundant assignment
@W:CD604 : ZARLINK_CONNECT_INIT.vhd(1019) | OTHERS clause is not synthesized 
@N:CD364 : ZARLINK_CONNECT_INIT.vhd(1031) | Removed redundant assignment
Post processing for work.zarlink_connect_init.behavioral
@N:CD630 : Zarlink_SPI_Module.vhd(7) | Synthesizing work.zarlink_spi_module.behavioral 
@N:CD232 : Zarlink_SPI_Module.vhd(47) | Using gray code encoding for type state_type
@N:CD364 : Zarlink_SPI_Module.vhd(130) | Removed redundant assignment
@N:CD364 : Zarlink_SPI_Module.vhd(136) | Removed redundant assignment
@N:CD364 : Zarlink_SPI_Module.vhd(137) | Removed redundant assignment
@N:CD364 : Zarlink_SPI_Module.vhd(138) | Removed redundant assignment
@N:CD364 : Zarlink_SPI_Module.vhd(141) | Removed redundant assignment
@W:CD604 : Zarlink_SPI_Module.vhd(393) | OTHERS clause is not synthesized 
Post processing for work.zarlink_spi_module.behavioral
@N:CD630 : COMMAND_RECEIVER.vhd(26) | Synthesizing work.command_receiver.behavioral 
@N:CD231 : COMMAND_RECEIVER.vhd(85) | Using onehot encoding for type state_type (sm_idle="100000000000000000000000")
@N:CD364 : COMMAND_RECEIVER.vhd(170) | Removed redundant assignment
@N:CD364 : COMMAND_RECEIVER.vhd(171) | Removed redundant assignment
@N:CD364 : COMMAND_RECEIVER.vhd(172) | Removed redundant assignment
@N:CD364 : COMMAND_RECEIVER.vhd(173) | Removed redundant assignment
@N:CD364 : COMMAND_RECEIVER.vhd(174) | Removed redundant assignment
@N:CD364 : COMMAND_RECEIVER.vhd(175) | Removed redundant assignment
@N:CD364 : COMMAND_RECEIVER.vhd(176) | Removed redundant assignment
@W:CD604 : COMMAND_RECEIVER.vhd(406) | OTHERS clause is not synthesized 
@W:CD604 : COMMAND_RECEIVER.vhd(452) | OTHERS clause is not synthesized 
@W:CD604 : COMMAND_RECEIVER.vhd(496) | OTHERS clause is not synthesized 
@N:CD364 : COMMAND_RECEIVER.vhd(520) | Removed redundant assignment
@N:CD364 : COMMAND_RECEIVER.vhd(521) | Removed redundant assignment
@N:CD364 : COMMAND_RECEIVER.vhd(554) | Removed redundant assignment
@N:CD364 : COMMAND_RECEIVER.vhd(555) | Removed redundant assignment
Post processing for work.command_receiver.behavioral
Post processing for work.control_nexus.behavioral
@W:CL240 : CONTROL_NEXUS.vhd(66) | DEBUG_STRING_ORGANIZER is not assigned a value (floating) -- simulation mismatch possible. 
@N:CD630 : FIFO.vhd(8) | Synthesizing work.fifo.def_arch 
@N:CD630 : igloo.vhd(2722) | Synthesizing igloo.vcc.syn_black_box 
Post processing for igloo.vcc.syn_black_box
@N:CD630 : igloo.vhd(1787) | Synthesizing igloo.gnd.syn_black_box 
Post processing for igloo.gnd.syn_black_box
@N:CD630 : igloo.vhd(2198) | Synthesizing igloo.or2.syn_black_box 
Post processing for igloo.or2.syn_black_box
@N:CD630 : igloo.vhd(1934) | Synthesizing igloo.inv.syn_black_box 
Post processing for igloo.inv.syn_black_box
@N:CD630 : igloo.vhd(3039) | Synthesizing igloo.fifo4k18.syn_black_box 
Post processing for igloo.fifo4k18.syn_black_box
@N:CD630 : igloo.vhd(13) | Synthesizing igloo.and2.syn_black_box 
Post processing for igloo.and2.syn_black_box
@N:CD630 : igloo.vhd(2032) | Synthesizing igloo.nand2.syn_black_box 
Post processing for igloo.nand2.syn_black_box
@N:CD630 : igloo.vhd(2040) | Synthesizing igloo.nand2a.syn_black_box 
Post processing for igloo.nand2a.syn_black_box
Post processing for work.fifo.def_arch
Post processing for work.implant_top.behavioral
@W:CL169 : IMPLANT_TOP.vhd(126) | Pruning register Counter_XXX_4(6 downto 0)  
@W:CL169 : IMPLANT_TOP.vhd(125) | Pruning register FIFO_EMPTY_2  
@W:CL169 : IMPLANT_TOP.vhd(124) | Pruning register FIFO_FULL_2  
@A:CL282 : IMPLANT_TOP.vhd(250) | Feedback mux created for signal FIFO_IN_READENABLE_LAST -- possible set/reset assignment for signal missing. Specifying a reset value will improve timing and area.
Post processing for work.igloo_top.igloo_top_arch
@N:CL201 : COMMAND_RECEIVER.vhd(157) | Trying to extract state machine for register SPIMACHINE_STATE
Extracted state machine for register SPIMACHINE_STATE
State machine has 24 reachable states with original encodings of:
   000000000000000000000001
   000000000000000000000010
   000000000000000000000100
   000000000000000000001000
   000000000000000000010000
   000000000000000000100000
   000000000000000001000000
   000000000000000010000000
   000000000000000100000000
   000000000000001000000000
   000000000000010000000000
   000000000000100000000000
   000000000001000000000000
   000000000010000000000000
   000000000100000000000000
   000000001000000000000000
   000000010000000000000000
   000000100000000000000000
   000001000000000000000000
   000010000000000000000000
   000100000000000000000000
   001000000000000000000000
   010000000000000000000000
   100000000000000000000000
@N:CL201 : Zarlink_SPI_Module.vhd(109) | Trying to extract state machine for register FSM_STATE
Extracted state machine for register FSM_STATE
State machine has 44 reachable states with original encodings of:
   000000
   000001
   000010
   000011
   000100
   000101
   000110
   000111
   001000
   001001
   001010
   001011
   001100
   001101
   001110
   001111
   010000
   010001
   010010
   010011
   010100
   010101
   010110
   010111
   011000
   011001
   011010
   011011
   011100
   011101
   011110
   011111
   110000
   110001
   110010
   110011
   110100
   110101
   110110
   110111
   111100
   111101
   111110
   111111
@N:CL201 : ZARLINK_CONNECT_INIT.vhd(196) | Trying to extract state machine for register FSM_STATE
Extracted state machine for register FSM_STATE
State machine has 36 reachable states with original encodings of:
   000000
   000001
   000010
   000011
   000100
   000101
   000110
   000111
   001000
   001001
   001010
   001011
   001100
   001101
   001110
   001111
   010000
   010001
   010010
   010011
   010100
   010101
   010110
   010111
   011000
   011001
   011010
   011011
   011100
   011101
   011110
   011111
   110000
   110001
   110010
   110011
@N:CL201 : ORGANIZER.vhd(117) | Trying to extract state machine for register SPIMACHINE_STATE
Extracted state machine for register SPIMACHINE_STATE
State machine has 15 reachable states with original encodings of:
   000000000000001
   000000000000010
   000000000000100
   000000000001000
   000000000010000
   000000000100000
   000000001000000
   000000010000000
   000000100000000
   000001000000000
   000010000000000
   000100000000000
   001000000000000
   010000000000000
   100000000000000
@N:CL201 : RHA_DATA_COLLECTOR.vhd(78) | Trying to extract state machine for register SPIMACHINE_STATE
Extracted state machine for register SPIMACHINE_STATE
State machine has 4 reachable states with original encodings of:
   00
   01
   10
   11
@W:CL190 : RHA_ARRAY.vhd(149) | Optimizing register bit AddressCheckInfo(8) to a constant 0
@W:CL260 : RHA_ARRAY.vhd(149) | Pruning register bit 8 of AddressCheckInfo(79 downto 4)  
@W:CL190 : RHA_ARRAY.vhd(149) | Optimizing register bit AddressCheckInfo(13) to a constant 0
@W:CL260 : RHA_ARRAY.vhd(149) | Pruning register bit 13 of AddressCheckInfo(79 downto 9)  
@W:CL190 : RHA_ARRAY.vhd(149) | Optimizing register bit AddressCheckInfo(18) to a constant 0
@W:CL260 : RHA_ARRAY.vhd(149) | Pruning register bit 18 of AddressCheckInfo(79 downto 14)  
@W:CL190 : RHA_ARRAY.vhd(149) | Optimizing register bit AddressCheckInfo(23) to a constant 0
@W:CL260 : RHA_ARRAY.vhd(149) | Pruning register bit 23 of AddressCheckInfo(79 downto 19)  
@W:CL279 : RHA_ARRAY.vhd(149) | Pruning register bits 2 to 1 of AddressCheckInfo(2 downto 0)  
@W:CL260 : RHA_ARRAY.vhd(149) | Pruning register bit 7 of AddressCheckInfo(7 downto 4)  
@W:CL190 : RHA_ARRAY.vhd(149) | Optimizing register bit AddressCheckInfo(28) to a constant 0
@W:CL260 : RHA_ARRAY.vhd(149) | Pruning register bit 28 of AddressCheckInfo(79 downto 24)  
@N:CL201 : RHA_TO_ZL_CONVERTER.vhd(309) | Trying to extract state machine for register SPIMACHINE_STATE
Extracted state machine for register SPIMACHINE_STATE
State machine has 32 reachable states with original encodings of:
   00000
   00001
   00010
   00011
   00100
   00101
   00110
   00111
   01000
   01001
   01010
   01011
   01100
   01101
   01110
   01111
   10000
   10001
   10010
   10011
   10100
   10101
   10110
   10111
   11000
   11001
   11010
   11011
   11100
   11101
   11110
   11111
@W:CL159 : RHA_TO_ZL_CONVERTER.vhd(70) | Input Clk_SLOW is unused
@W:CL279 : RHA_TEST_MODULATOR.vhd(337) | Pruning register bits 20 to 17 of TESTVALUE(24 downto 1)  
@W:CL260 : RHA_TEST_MODULATOR.vhd(337) | Pruning register bit 2 of TESTVALUE(24 downto 1)  
@N:CL201 : RHA_TEST_MODULATOR.vhd(123) | Trying to extract state machine for register SPIMACHINE_STATE
Extracted state machine for register SPIMACHINE_STATE
State machine has 51 reachable states with original encodings of:
   000000
   000001
   000010
   000011
   000100
   000101
   000110
   000111
   001000
   001001
   001010
   001011
   001100
   001101
   001110
   001111
   010000
   010001
   010010
   010011
   010100
   010101
   010110
   010111
   011000
   011001
   011010
   011011
   011100
   011101
   011110
   011111
   101000
   101001
   101011
   110000
   110001
   110010
   110011
   110100
   110101
   110110
   110111
   111000
   111001
   111010
   111011
   111100
   111101
   111110
   111111
@W:CL279 : RHA_TEST_MODULATOR.vhd(123) | Pruning register bits 20 to 17 of TESTVALUE_INTERNAL(24 downto 3)  
@W:CL260 : RHA_TEST_MODULATOR.vhd(123) | Pruning register bit 1 of TESTVALUE_INTERNAL(1 downto 0)  
@W:CL190 : IMPLANT_TOP.vhd(268) | Optimizing register bit DEBUG_COUNTER(3) to a constant 0
@W:CL260 : IMPLANT_TOP.vhd(268) | Pruning register bit 3 of DEBUG_COUNTER(3 downto 0)  
@END

At c_vhdl Exit (Real Time elapsed 0h:00m:01s; CPU Time elapsed 0h:00m:01s; Memory used current: 85MB peak: 91MB)

Process took 0h:00m:01s realtime, 0h:00m:01s cputime
# Fri Sep 05 17:35:14 2014

###########################################################]
Pre-mapping Report

Synopsys Microsemi Technology Pre-mapping, Version mapact, Build 1154R, Built Jan 20 2014 10:14:08
Copyright (C) 1994-2013, Synopsys, Inc. This software and the associated documentation are proprietary to Synopsys, Inc. This software may only be used in accordance with the terms and conditions of a written license agreement with Synopsys, Inc.  All other use or distribution of the software is strictly prohibited.
Product Version I-2013.09M-SP1 

Mapper Startup Complete (Real Time elapsed 0h:00m:00s; CPU Time elapsed 0h:00m:00s; Memory used current: 98MB peak: 99MB)

Reading constraint file: C:\Users\ITP\Desktop\IGLOO\IGLOO_RHA\constraint\IGLOO.sdc
Linked File: IGLOO_TOP_scck.rpt
Printing clock  summary report in "C:\Users\ITP\Desktop\IGLOO\IGLOO_RHA\synthesis\IGLOO_TOP_scck.rpt" file 
@W:BN544 : IGLOO.sdc(3) | create_generated_clock with both -multiply_by and -divide_by not supported for this target technology
@W:BN544 : IGLOO.sdc(5) | create_generated_clock with both -multiply_by and -divide_by not supported for this target technology
@N:MF248 :  | Running in 64-bit mode. 
@N:MF667 :  | Clock conversion disabled  

Design Input Complete (Real Time elapsed 0h:00m:00s; CPU Time elapsed 0h:00m:00s; Memory used current: 102MB peak: 104MB)


Mapper Initialization Complete (Real Time elapsed 0h:00m:00s; CPU Time elapsed 0h:00m:00s; Memory used current: 102MB peak: 104MB)

@N:BN362 : implant_top.vhd(268) | Removing sequential instance DEBUG_ZARLINK_VALID_INTERNAL of view:PrimLib.dffr(prim) in hierarchy view:work.IMPLANT_TOP(behavioral) because there are no references to its outputs 
@N:BN362 : rha_test_modulator.vhd(123) | Removing sequential instance ADC_SYNC_INTERNAL of view:PrimLib.dffr(prim) in hierarchy view:work.RHA_TEST_MODULATOR_RHA_TEST_MOD_0(behavioral) because there are no references to its outputs 
@N:BN362 : rha_test_modulator.vhd(123) | Removing sequential instance ADC_SYNC_INTERNAL of view:PrimLib.dffr(prim) in hierarchy view:work.RHA_TEST_MODULATOR_RHA_TEST_MOD_1(behavioral) because there are no references to its outputs 
@N:BN362 : rha_test_modulator.vhd(123) | Removing sequential instance ADC_SYNC_INTERNAL of view:PrimLib.dffr(prim) in hierarchy view:work.RHA_TEST_MODULATOR_RHA_TEST_MOD_2(behavioral) because there are no references to its outputs 
@N:BN362 : rha_test_modulator.vhd(123) | Removing sequential instance ADC_SYNC_INTERNAL of view:PrimLib.dffr(prim) in hierarchy view:work.RHA_TEST_MODULATOR_RHA_TEST_MOD_3(behavioral) because there are no references to its outputs 
@N:BN362 : rha_test_modulator.vhd(123) | Removing sequential instance ADC_SYNC_INTERNAL of view:PrimLib.dffr(prim) in hierarchy view:work.RHA_TEST_MODULATOR_RHA_TEST_MOD_4(behavioral) because there are no references to its outputs 
@N:BN362 : rha_test_modulator.vhd(123) | Removing sequential instance ADC_SYNC_INTERNAL of view:PrimLib.dffr(prim) in hierarchy view:work.RHA_TEST_MODULATOR_RHA_TEST_MOD_5(behavioral) because there are no references to its outputs 
@N:BN362 : rha_test_modulator.vhd(123) | Removing sequential instance ADC_SYNC_INTERNAL of view:PrimLib.dffr(prim) in hierarchy view:work.RHA_TEST_MODULATOR_RHA_TEST_MOD_6(behavioral) because there are no references to its outputs 
@N:BN362 : rha_test_modulator.vhd(123) | Removing sequential instance ADC_SYNC_INTERNAL of view:PrimLib.dffr(prim) in hierarchy view:work.RHA_TEST_MODULATOR_RHA_TEST_MOD_7(behavioral) because there are no references to its outputs 
@N:BN115 : data_acquisition_block.vhd(164) | Removing instance RHA_ERROR_WATCHDOG of view:work.RHA_ERROR_WATCHDOG(behavioral) because there are no references to its outputs 
@N:BN362 : rha_error_watchdog.vhd(81) | Removing sequential instance ERROR_RHA_ASYNC_FIRST_OUT_INTERNAL of view:PrimLib.dffre(prim) in hierarchy view:work.RHA_ERROR_WATCHDOG(behavioral) because there are no references to its outputs 
@N:BN362 : rha_error_watchdog.vhd(81) | Removing sequential instance ERROR_RHA_ASYNC_VALID_OUT_INTERNAL of view:PrimLib.dffre(prim) in hierarchy view:work.RHA_ERROR_WATCHDOG(behavioral) because there are no references to its outputs 
@N:BN362 : rha_error_watchdog.vhd(81) | Removing sequential instance ERROR_PARITY_OUT_INTERNAL of view:PrimLib.dffre(prim) in hierarchy view:work.RHA_ERROR_WATCHDOG(behavioral) because there are no references to its outputs 
@N:BN362 : rha_error_watchdog.vhd(81) | Removing sequential instance ERROR_PATTERN_OUT_INTERNAL of view:PrimLib.dffre(prim) in hierarchy view:work.RHA_ERROR_WATCHDOG(behavioral) because there are no references to its outputs 
@N:BN362 : rha_error_watchdog.vhd(81) | Removing sequential instance ERROR_ADDRESS_OUT_INTERNAL of view:PrimLib.dffre(prim) in hierarchy view:work.RHA_ERROR_WATCHDOG(behavioral) because there are no references to its outputs 
@N:BN362 : rha_error_watchdog.vhd(81) | Removing sequential instance ERROR_BUFFEROVERFLOW_OUT_INTERNAL of view:PrimLib.dffre(prim) in hierarchy view:work.RHA_ERROR_WATCHDOG(behavioral) because there are no references to its outputs 
@N:BN362 : rha_error_watchdog.vhd(81) | Removing sequential instance ERROR_ALL_OUT_INTERNAL of view:PrimLib.dffre(prim) in hierarchy view:work.RHA_ERROR_WATCHDOG(behavioral) because there are no references to its outputs 
@N:BN362 : rha_array.vhd(591) | Removing sequential instance ERROR_RHA_ASYNC_FIRST_INTERNAL of view:PrimLib.dffre(prim) in hierarchy view:work.RHA_ARRAY(behavioral) because there are no references to its outputs 
@N:BN362 : rha_array.vhd(591) | Removing sequential instance ERROR_RHA_ASYNC_VALID_INTERNAL of view:PrimLib.dffre(prim) in hierarchy view:work.RHA_ARRAY(behavioral) because there are no references to its outputs 
@N:BN362 : rha_array.vhd(149) | Removing sequential instance ERROR_PARITY of view:PrimLib.dffr(prim) in hierarchy view:work.RHA_ARRAY(behavioral) because there are no references to its outputs 
@N:BN362 : rha_array.vhd(149) | Removing sequential instance ERROR_PATTERN of view:PrimLib.dffr(prim) in hierarchy view:work.RHA_ARRAY(behavioral) because there are no references to its outputs 
@N:BN362 : rha_array.vhd(149) | Removing sequential instance ERROR_ADDRESS of view:PrimLib.dffr(prim) in hierarchy view:work.RHA_ARRAY(behavioral) because there are no references to its outputs 
@N:BN362 : rha_array.vhd(149) | Removing sequential instance BufferOverflow_INTERNAL of view:PrimLib.dffre(prim) in hierarchy view:work.RHA_ARRAY(behavioral) because there are no references to its outputs 
@N:BN115 : rha_array.vhd(406) | Removing instance DataChecker of view:work.RHA_DATA_CHECKER(behavioral) because there are no references to its outputs 
@N:BN362 : rha_array.vhd(149) | Removing sequential instance AddressCheckInfo[79:29] of view:PrimLib.dffre(prim) in hierarchy view:work.RHA_ARRAY(behavioral) because there are no references to its outputs 
@N:BN362 : rha_array.vhd(421) | Removing sequential instance DATAOUT_PARITY_INTERNAL[7:0] of view:PrimLib.dffre(prim) in hierarchy view:work.RHA_ARRAY(behavioral) because there are no references to its outputs 
@N:BN362 : rha_array.vhd(421) | Removing sequential instance DATAOUT_PATTERN_INTERNAL[23:0] of view:PrimLib.dffre(prim) in hierarchy view:work.RHA_ARRAY(behavioral) because there are no references to its outputs 
@N:BN362 : rha_array.vhd(421) | Removing sequential instance DATAOUT_ADDRESS_INTERNAL[39:0] of view:PrimLib.dffre(prim) in hierarchy view:work.RHA_ARRAY(behavioral) because there are no references to its outputs 
@N:BN362 : rha_array.vhd(149) | Removing sequential instance AddressCheckInfo[27:24] of view:PrimLib.dffre(prim) in hierarchy view:work.RHA_ARRAY(behavioral) because there are no references to its outputs 
@N:BN362 : rha_array.vhd(149) | Removing sequential instance AddressCheckInfo[22:19] of view:PrimLib.dffre(prim) in hierarchy view:work.RHA_ARRAY(behavioral) because there are no references to its outputs 
@N:BN362 : rha_array.vhd(149) | Removing sequential instance AddressCheckInfo[17:14] of view:PrimLib.dffre(prim) in hierarchy view:work.RHA_ARRAY(behavioral) because there are no references to its outputs 
@N:BN362 : rha_array.vhd(149) | Removing sequential instance AddressCheckInfo[12:9] of view:PrimLib.dffre(prim) in hierarchy view:work.RHA_ARRAY(behavioral) because there are no references to its outputs 
@N:BN362 : rha_array.vhd(149) | Removing sequential instance AddressCheckInfo[6:4] of view:PrimLib.dffre(prim) in hierarchy view:work.RHA_ARRAY(behavioral) because there are no references to its outputs 
@N:BN362 : rha_array.vhd(149) | Removing sequential instance AddressCheckInfo[0] of view:PrimLib.dffre(prim) in hierarchy view:work.RHA_ARRAY(behavioral) because there are no references to its outputs 


Clock Summary
**************

Start             Requested     Requested     Clock        Clock           
Clock             Frequency     Period        Type         Group           
---------------------------------------------------------------------------
IGLOO_TOP|Clk     24.0 MHz      41.666        declared     default_clkgroup
===========================================================================

@W:MT532 : fifo.vhd(443) | Found signal identified as System clock which controls 2584 sequential elements including MYImplant.MyFIFO.\\FIFOBLOCK\[1\]\\.  Using this clock, which has no specified timing constraint, can adversely impact design performance. 

Finished Pre Mapping Phase.
@N:BN225 :  | Writing default property annotation file C:\Users\ITP\Desktop\IGLOO\IGLOO_RHA\synthesis\IGLOO_TOP.sap. 
Pre-mapping successful!

At Mapper Exit (Real Time elapsed 0h:00m:00s; CPU Time elapsed 0h:00m:00s; Memory used current: 50MB peak: 114MB)

Process took 0h:00m:01s realtime, 0h:00m:01s cputime
# Fri Sep 05 17:35:15 2014

###########################################################]
Map & Optimize Report

Synopsys Microsemi Technology Mapper, Version mapact, Build 1154R, Built Jan 20 2014 10:14:08
Copyright (C) 1994-2013, Synopsys, Inc. This software and the associated documentation are proprietary to Synopsys, Inc. This software may only be used in accordance with the terms and conditions of a written license agreement with Synopsys, Inc.  All other use or distribution of the software is strictly prohibited.
Product Version I-2013.09M-SP1 

Mapper Startup Complete (Real Time elapsed 0h:00m:00s; CPU Time elapsed 0h:00m:00s; Memory used current: 99MB peak: 99MB)

@N:MF248 :  | Running in 64-bit mode. 
@N:MF667 :  | Clock conversion disabled  

Design Input Complete (Real Time elapsed 0h:00m:00s; CPU Time elapsed 0h:00m:00s; Memory used current: 99MB peak: 100MB)


Mapper Initialization Complete (Real Time elapsed 0h:00m:00s; CPU Time elapsed 0h:00m:00s; Memory used current: 99MB peak: 100MB)

@W:BN544 : igloo.sdc(3) | create_generated_clock with both -multiply_by and -divide_by not supported for this target technology
@W:BN544 : igloo.sdc(5) | create_generated_clock with both -multiply_by and -divide_by not supported for this target technology


Starting Optimization and Mapping (Real Time elapsed 0h:00m:00s; CPU Time elapsed 0h:00m:00s; Memory used current: 111MB peak: 111MB)

@N:BN362 : implant_top.vhd(268) | Removing sequential instance DEBUG_COUNTER[2:0] of view:PrimLib.dffr(prim) in hierarchy view:work.IMPLANT_TOP(behavioral) because there are no references to its outputs 
@N:BN362 : implant_top.vhd(268) | Removing sequential instance DEBUG_ZARLINK_DATA_INTERNAL[15:0] of view:PrimLib.dffr(prim) in hierarchy view:work.IMPLANT_TOP(behavioral) because there are no references to its outputs 

Available hyper_sources - for debug and ip models
	None Found

@W:MT462 : command_receiver.vhd(504) | Net MYImplant.Inst_CONTROL_NEXUS.Inst_COMMAND_RECEIVER.Clk_Reduced appears to be an unidentified clock source. Assuming default frequency. 

Finished RTL optimizations (Real Time elapsed 0h:00m:00s; CPU Time elapsed 0h:00m:00s; Memory used current: 113MB peak: 115MB)

@N: : implant_top.vhd(293) | Found counter in view:work.IGLOO_TOP(igloo_top_arch) inst MYImplant.RHA_RESET_COUNTER[4:0]
@N:MO106 : command_receiver.vhd(463) | Found ROM, 'BITMASK_SAMPLE_TOP_INTERNAL_16[14:1]', 16 words by 14 bits 
@N:MO106 : command_receiver.vhd(419) | Found ROM, 'BITMASK_SAMPLE_INTERNAL_17[14:9]', 16 words by 6 bits 
@N:MO106 : command_receiver.vhd(419) | Found ROM, 'BITMASK_SAMPLE_INTERNAL_17[7:1]', 16 words by 7 bits 
Encoding state machine SPIMACHINE_STATE[0:23] (view:work.COMMAND_RECEIVER(behavioral))
original code -> new code
   000000000000000000000001 -> 000000000000000000000001
   000000000000000000000010 -> 000000000000000000000010
   000000000000000000000100 -> 000000000000000000000100
   000000000000000000001000 -> 000000000000000000001000
   000000000000000000010000 -> 000000000000000000010000
   000000000000000000100000 -> 000000000000000000100000
   000000000000000001000000 -> 000000000000000001000000
   000000000000000010000000 -> 000000000000000010000000
   000000000000000100000000 -> 000000000000000100000000
   000000000000001000000000 -> 000000000000001000000000
   000000000000010000000000 -> 000000000000010000000000
   000000000000100000000000 -> 000000000000100000000000
   000000000001000000000000 -> 000000000001000000000000
   000000000010000000000000 -> 000000000010000000000000
   000000000100000000000000 -> 000000000100000000000000
   000000001000000000000000 -> 000000001000000000000000
   000000010000000000000000 -> 000000010000000000000000
   000000100000000000000000 -> 000000100000000000000000
   000001000000000000000000 -> 000001000000000000000000
   000010000000000000000000 -> 000010000000000000000000
   000100000000000000000000 -> 000100000000000000000000
   001000000000000000000000 -> 001000000000000000000000
   010000000000000000000000 -> 010000000000000000000000
   100000000000000000000000 -> 100000000000000000000000
@N:MF238 : command_receiver.vhd(530) | Found 8-bit incrementor, 'un7_counter[7:0]'
@N: : zarlink_spi_module.vhd(109) | Found counter in view:work.Zarlink_SPI_Module(behavioral) inst Counter[3:0]
Encoding state machine FSM_STATE[0:43] (view:work.Zarlink_SPI_Module(behavioral))
original code -> new code
   000000 -> 000000
   000001 -> 000001
   000010 -> 000011
   000011 -> 000010
   000100 -> 000110
   000101 -> 000111
   000110 -> 000101
   000111 -> 000100
   001000 -> 001100
   001001 -> 001101
   001010 -> 001111
   001011 -> 001110
   001100 -> 001010
   001101 -> 001011
   001110 -> 001001
   001111 -> 001000
   010000 -> 011000
   010001 -> 011001
   010010 -> 011011
   010011 -> 011010
   010100 -> 011110
   010101 -> 011111
   010110 -> 011101
   010111 -> 011100
   011000 -> 010100
   011001 -> 010101
   011010 -> 010111
   011011 -> 010110
   011100 -> 010010
   011101 -> 010011
   011110 -> 010001
   011111 -> 010000
   110000 -> 110000
   110001 -> 110001
   110010 -> 110011
   110011 -> 110010
   110100 -> 110110
   110101 -> 110111
   110110 -> 110101
   110111 -> 110100
   111100 -> 111100
   111101 -> 111101
   111110 -> 111111
   111111 -> 111110
@N: : zarlink_connect_init.vhd(196) | Found counter in view:work.ZARLINK_CONNECT_INIT(behavioral) inst Zarlink_ConnectionTimeoutCounter[31:0]
@N: : zarlink_connect_init.vhd(196) | Found counter in view:work.ZARLINK_CONNECT_INIT(behavioral) inst Zarlink_ResetCounter[23:0]
@N: : zarlink_connect_init.vhd(196) | Found counter in view:work.ZARLINK_CONNECT_INIT(behavioral) inst Zarlink_Retry_Counter[16:0]
Encoding state machine FSM_STATE[0:35] (view:work.ZARLINK_CONNECT_INIT(behavioral))
original code -> new code
   000000 -> 000000
   000001 -> 000001
   000010 -> 000011
   000011 -> 000010
   000100 -> 000110
   000101 -> 000111
   000110 -> 000101
   000111 -> 000100
   001000 -> 001100
   001001 -> 001101
   001010 -> 001111
   001011 -> 001110
   001100 -> 001010
   001101 -> 001011
   001110 -> 001001
   001111 -> 001000
   010000 -> 011000
   010001 -> 011001
   010010 -> 011011
   010011 -> 011010
   010100 -> 011110
   010101 -> 011111
   010110 -> 011101
   010111 -> 011100
   011000 -> 010100
   011001 -> 010101
   011010 -> 010111
   011011 -> 010110
   011100 -> 010010
   011101 -> 010011
   011110 -> 010001
   011111 -> 010000
   110000 -> 110000
   110001 -> 110001
   110010 -> 110011
   110011 -> 110010
@N: : organizer.vhd(117) | Found counter in view:work.ORGANIZER(behavioral) inst WaitForXCyles_Counter[17:0]
@N: : organizer.vhd(117) | Found counter in view:work.ORGANIZER(behavioral) inst CheckOutgoingBuffer_SIZE_COPY[6:0]
Encoding state machine SPIMACHINE_STATE[0:14] (view:work.ORGANIZER(behavioral))
original code -> new code
   000000000000001 -> 000000000000001
   000000000000010 -> 000000000000010
   000000000000100 -> 000000000000100
   000000000001000 -> 000000000001000
   000000000010000 -> 000000000010000
   000000000100000 -> 000000000100000
   000000001000000 -> 000000001000000
   000000010000000 -> 000000010000000
   000000100000000 -> 000000100000000
   000001000000000 -> 000001000000000
   000010000000000 -> 000010000000000
   000100000000000 -> 000100000000000
   001000000000000 -> 001000000000000
   010000000000000 -> 010000000000000
   100000000000000 -> 100000000000000
Encoding state machine SPIMACHINE_STATE[0:3] (view:work.RHA_DATA_COLLECTOR(behavioral))
original code -> new code
   00 -> 00
   01 -> 01
   10 -> 10
   11 -> 11
@N:MF238 : rha_data_collector.vhd(181) | Found 5-bit incrementor, 'un10_counter_1[4:0]'
@N:BN362 : rha_data_collector.vhd(99) | Removing sequential instance DATAOUT_DATA_INTERNAL_BUFFER[17] of view:PrimLib.dffr(prim) in hierarchy view:work.RHA_DATA_COLLECTOR(behavioral) because there are no references to its outputs 
@N:BN362 : rha_data_collector.vhd(99) | Removing sequential instance DATAOUT_DATA_INTERNAL_BUFFER[18] of view:PrimLib.dffr(prim) in hierarchy view:work.RHA_DATA_COLLECTOR(behavioral) because there are no references to its outputs 
@N:BN362 : rha_data_collector.vhd(99) | Removing sequential instance DATAOUT_DATA_INTERNAL_BUFFER[19] of view:PrimLib.dffr(prim) in hierarchy view:work.RHA_DATA_COLLECTOR(behavioral) because there are no references to its outputs 
@N:BN362 : rha_data_collector.vhd(99) | Removing sequential instance DATAOUT_DATA_INTERNAL_BUFFER[20] of view:PrimLib.dffr(prim) in hierarchy view:work.RHA_DATA_COLLECTOR(behavioral) because there are no references to its outputs 
@N:BN362 : rha_data_collector.vhd(99) | Removing sequential instance DATAOUT_DATA_INTERNAL_BUFFER[21] of view:PrimLib.dffr(prim) in hierarchy view:work.RHA_DATA_COLLECTOR(behavioral) because there are no references to its outputs 
@N:BN362 : rha_data_collector.vhd(99) | Removing sequential instance DATAOUT_DATA_INTERNAL_BUFFER[22] of view:PrimLib.dffr(prim) in hierarchy view:work.RHA_DATA_COLLECTOR(behavioral) because there are no references to its outputs 
@N:BN362 : rha_data_collector.vhd(99) | Removing sequential instance DATAOUT_DATA_INTERNAL_BUFFER[23] of view:PrimLib.dffr(prim) in hierarchy view:work.RHA_DATA_COLLECTOR(behavioral) because there are no references to its outputs 
@N:BN362 : rha_data_collector.vhd(99) | Removing sequential instance DATAOUT_DATA_INTERNAL_BUFFER[24] of view:PrimLib.dffr(prim) in hierarchy view:work.RHA_DATA_COLLECTOR(behavioral) because there are no references to its outputs 
@N:BN362 : rha_data_collector.vhd(99) | Removing sequential instance DATAOUT_DATA_INTERNAL_BUFFER[0] of view:PrimLib.dffr(prim) in hierarchy view:work.RHA_DATA_COLLECTOR(behavioral) because there are no references to its outputs 
@N:BN362 : rha_data_collector.vhd(99) | Removing sequential instance DATAOUT_DATA_INTERNAL_BUFFER_PRE[17] of view:PrimLib.dffr(prim) in hierarchy view:work.RHA_DATA_COLLECTOR(behavioral) because there are no references to its outputs 
@N:BN362 : rha_data_collector.vhd(99) | Removing sequential instance DATAOUT_DATA_INTERNAL_BUFFER_PRE[18] of view:PrimLib.dffr(prim) in hierarchy view:work.RHA_DATA_COLLECTOR(behavioral) because there are no references to its outputs 
@N:BN362 : rha_data_collector.vhd(99) | Removing sequential instance DATAOUT_DATA_INTERNAL_BUFFER_PRE[19] of view:PrimLib.dffr(prim) in hierarchy view:work.RHA_DATA_COLLECTOR(behavioral) because there are no references to its outputs 
@N:BN362 : rha_data_collector.vhd(99) | Removing sequential instance DATAOUT_DATA_INTERNAL_BUFFER_PRE[20] of view:PrimLib.dffr(prim) in hierarchy view:work.RHA_DATA_COLLECTOR(behavioral) because there are no references to its outputs 
@N:BN362 : rha_data_collector.vhd(99) | Removing sequential instance DATAOUT_DATA_INTERNAL_BUFFER_PRE[21] of view:PrimLib.dffr(prim) in hierarchy view:work.RHA_DATA_COLLECTOR(behavioral) because there are no references to its outputs 
@N:BN362 : rha_data_collector.vhd(99) | Removing sequential instance DATAOUT_DATA_INTERNAL_BUFFER_PRE[22] of view:PrimLib.dffr(prim) in hierarchy view:work.RHA_DATA_COLLECTOR(behavioral) because there are no references to its outputs 
@N:BN362 : rha_data_collector.vhd(99) | Removing sequential instance DATAOUT_DATA_INTERNAL_BUFFER_PRE[23] of view:PrimLib.dffr(prim) in hierarchy view:work.RHA_DATA_COLLECTOR(behavioral) because there are no references to its outputs 
@N:BN362 : rha_data_collector.vhd(99) | Removing sequential instance DATAOUT_DATA_INTERNAL_BUFFER_PRE[24] of view:PrimLib.dffr(prim) in hierarchy view:work.RHA_DATA_COLLECTOR(behavioral) because there are no references to its outputs 
@N:BN362 : rha_data_collector.vhd(99) | Removing sequential instance DATAOUT_DATA_INTERNAL_BUFFER_PRE[0] of view:PrimLib.dffr(prim) in hierarchy view:work.RHA_DATA_COLLECTOR(behavioral) because there are no references to its outputs 
@N:BN362 : rha_data_collector.vhd(99) | Removing sequential instance DATAOUT_DATA_INTERNAL[23] of view:PrimLib.dffr(prim) in hierarchy view:work.RHA_DATA_COLLECTOR(behavioral) because there are no references to its outputs 
@N:BN362 : rha_data_collector.vhd(99) | Removing sequential instance DATAOUT_DATA_INTERNAL[22] of view:PrimLib.dffr(prim) in hierarchy view:work.RHA_DATA_COLLECTOR(behavioral) because there are no references to its outputs 
@N:BN362 : rha_data_collector.vhd(99) | Removing sequential instance DATAOUT_DATA_INTERNAL[21] of view:PrimLib.dffr(prim) in hierarchy view:work.RHA_DATA_COLLECTOR(behavioral) because there are no references to its outputs 
@N:BN362 : rha_data_collector.vhd(99) | Removing sequential instance DATAOUT_DATA_INTERNAL[20] of view:PrimLib.dffr(prim) in hierarchy view:work.RHA_DATA_COLLECTOR(behavioral) because there are no references to its outputs 
@N:BN362 : rha_data_collector.vhd(99) | Removing sequential instance DATAOUT_DATA_INTERNAL[19] of view:PrimLib.dffr(prim) in hierarchy view:work.RHA_DATA_COLLECTOR(behavioral) because there are no references to its outputs 
@N:BN362 : rha_data_collector.vhd(99) | Removing sequential instance DATAOUT_DATA_INTERNAL[18] of view:PrimLib.dffr(prim) in hierarchy view:work.RHA_DATA_COLLECTOR(behavioral) because there are no references to its outputs 
@N:BN362 : rha_data_collector.vhd(99) | Removing sequential instance DATAOUT_DATA_INTERNAL[17] of view:PrimLib.dffr(prim) in hierarchy view:work.RHA_DATA_COLLECTOR(behavioral) because there are no references to its outputs 
@N:BN362 : rha_data_collector.vhd(99) | Removing sequential instance DATAOUT_DATA_INTERNAL[16] of view:PrimLib.dffr(prim) in hierarchy view:work.RHA_DATA_COLLECTOR(behavioral) because there are no references to its outputs 
@N: : rha_to_zl_converter.vhd(290) | Found counter in view:work.RHA_TO_ZL_CONVERTER(behavioral) inst Timestamp[15:0]
Encoding state machine SPIMACHINE_STATE[0:31] (view:work.RHA_TO_ZL_CONVERTER(behavioral))
original code -> new code
   00000 -> 00000
   00001 -> 00001
   00010 -> 00011
   00011 -> 00010
   00100 -> 00110
   00101 -> 00111
   00110 -> 00101
   00111 -> 00100
   01000 -> 01100
   01001 -> 01101
   01010 -> 01111
   01011 -> 01110
   01100 -> 01010
   01101 -> 01011
   01110 -> 01001
   01111 -> 01000
   10000 -> 11000
   10001 -> 11001
   10010 -> 11011
   10011 -> 11010
   10100 -> 11110
   10101 -> 11111
   10110 -> 11101
   10111 -> 11100
   11000 -> 10100
   11001 -> 10101
   11010 -> 10111
   11011 -> 10110
   11100 -> 10010
   11101 -> 10011
   11110 -> 10001
   11111 -> 10000
@N:MF176 :  | Default generator successful  
@N:MF176 :  | Default generator successful  
@N: : rha_test_modulator.vhd(337) | Found counter in view:work.RHA_TEST_MODULATOR_RHA_TEST_MOD_0_RHA_TEST_MOD_0_RHA_TEST_MOD_0(behavioral) inst Sample_Counter[8:0]
@N: : rha_test_modulator.vhd(337) | Found counter in view:work.RHA_TEST_MODULATOR_RHA_TEST_MOD_0_RHA_TEST_MOD_0_RHA_TEST_MOD_0(behavioral) inst Channel_Counter[3:0]
@N:MO106 : rha_test_modulator.vhd(370) | Found ROM, 'DATAOUT_ADDRESS[3]', 16 words by 1 bits 
Encoding state machine SPIMACHINE_STATE[0:50] (view:work.RHA_TEST_MODULATOR_RHA_TEST_MOD_0_RHA_TEST_MOD_0_RHA_TEST_MOD_0(behavioral))
original code -> new code
   000000 -> 000000
   000001 -> 000001
   000010 -> 000011
   000011 -> 000010
   000100 -> 000110
   000101 -> 000111
   000110 -> 000101
   000111 -> 000100
   001000 -> 001100
   001001 -> 001101
   001010 -> 001111
   001011 -> 001110
   001100 -> 001010
   001101 -> 001011
   001110 -> 001001
   001111 -> 001000
   010000 -> 011000
   010001 -> 011001
   010010 -> 011011
   010011 -> 011010
   010100 -> 011110
   010101 -> 011111
   010110 -> 011101
   010111 -> 011100
   011000 -> 010100
   011001 -> 010101
   011010 -> 010111
   011011 -> 010110
   011100 -> 010010
   011101 -> 010011
   011110 -> 010001
   011111 -> 010000
   101000 -> 110000
   101001 -> 110001
   101011 -> 110011
   110000 -> 110010
   110001 -> 110110
   110010 -> 110111
   110011 -> 110101
   110100 -> 110100
   110101 -> 111100
   110110 -> 111101
   110111 -> 111111
   111000 -> 111110
   111001 -> 111010
   111010 -> 111011
   111011 -> 111001
   111100 -> 111000
   111101 -> 101000
   111110 -> 101001
   111111 -> 101011
@W:MO160 : rha_test_modulator.vhd(123) | Register bit TESTVALUE_INTERNAL[23] is always 0, optimizing ...
@W:MO160 : rha_test_modulator.vhd(123) | Register bit TESTVALUE_INTERNAL[22] is always 0, optimizing ...
@W:MO160 : rha_test_modulator.vhd(123) | Register bit TESTVALUE_INTERNAL[21] is always 0, optimizing ...
@N: : rha_test_modulator.vhd(337) | Found counter in view:work.RHA_TEST_MODULATOR_RHA_TEST_MOD_5_RHA_TEST_MOD_1(behavioral) inst Sample_Counter[8:0]
@N: : rha_test_modulator.vhd(337) | Found counter in view:work.RHA_TEST_MODULATOR_RHA_TEST_MOD_5_RHA_TEST_MOD_1(behavioral) inst Channel_Counter[3:0]
@N:MO106 : rha_test_modulator.vhd(370) | Found ROM, 'DATAOUT_ADDRESS[3]', 16 words by 1 bits 
Encoding state machine SPIMACHINE_STATE[0:50] (view:work.RHA_TEST_MODULATOR_RHA_TEST_MOD_5_RHA_TEST_MOD_1(behavioral))
original code -> new code
   000000 -> 000000
   000001 -> 000001
   000010 -> 000011
   000011 -> 000010
   000100 -> 000110
   000101 -> 000111
   000110 -> 000101
   000111 -> 000100
   001000 -> 001100
   001001 -> 001101
   001010 -> 001111
   001011 -> 001110
   001100 -> 001010
   001101 -> 001011
   001110 -> 001001
   001111 -> 001000
   010000 -> 011000
   010001 -> 011001
   010010 -> 011011
   010011 -> 011010
   010100 -> 011110
   010101 -> 011111
   010110 -> 011101
   010111 -> 011100
   011000 -> 010100
   011001 -> 010101
   011010 -> 010111
   011011 -> 010110
   011100 -> 010010
   011101 -> 010011
   011110 -> 010001
   011111 -> 010000
   101000 -> 110000
   101001 -> 110001
   101011 -> 110011
   110000 -> 110010
   110001 -> 110110
   110010 -> 110111
   110011 -> 110101
   110100 -> 110100
   110101 -> 111100
   110110 -> 111101
   110111 -> 111111
   111000 -> 111110
   111001 -> 111010
   111010 -> 111011
   111011 -> 111001
   111100 -> 111000
   111101 -> 101000
   111110 -> 101001
   111111 -> 101011
@W:MO160 : rha_test_modulator.vhd(123) | Register bit TESTVALUE_INTERNAL[23] is always 0, optimizing ...
@W:MO160 : rha_test_modulator.vhd(123) | Register bit TESTVALUE_INTERNAL[22] is always 0, optimizing ...
@N: : rha_test_modulator.vhd(337) | Found counter in view:work.RHA_TEST_MODULATOR_RHA_TEST_MOD_0_RHA_TEST_MOD_2(behavioral) inst Sample_Counter[8:0]
@N: : rha_test_modulator.vhd(337) | Found counter in view:work.RHA_TEST_MODULATOR_RHA_TEST_MOD_0_RHA_TEST_MOD_2(behavioral) inst Channel_Counter[3:0]
@N:MO106 : rha_test_modulator.vhd(370) | Found ROM, 'DATAOUT_ADDRESS[3]', 16 words by 1 bits 
Encoding state machine SPIMACHINE_STATE[0:50] (view:work.RHA_TEST_MODULATOR_RHA_TEST_MOD_0_RHA_TEST_MOD_2(behavioral))
original code -> new code
   000000 -> 000000
   000001 -> 000001
   000010 -> 000011
   000011 -> 000010
   000100 -> 000110
   000101 -> 000111
   000110 -> 000101
   000111 -> 000100
   001000 -> 001100
   001001 -> 001101
   001010 -> 001111
   001011 -> 001110
   001100 -> 001010
   001101 -> 001011
   001110 -> 001001
   001111 -> 001000
   010000 -> 011000
   010001 -> 011001
   010010 -> 011011
   010011 -> 011010
   010100 -> 011110
   010101 -> 011111
   010110 -> 011101
   010111 -> 011100
   011000 -> 010100
   011001 -> 010101
   011010 -> 010111
   011011 -> 010110
   011100 -> 010010
   011101 -> 010011
   011110 -> 010001
   011111 -> 010000
   101000 -> 110000
   101001 -> 110001
   101011 -> 110011
   110000 -> 110010
   110001 -> 110110
   110010 -> 110111
   110011 -> 110101
   110100 -> 110100
   110101 -> 111100
   110110 -> 111101
   110111 -> 111111
   111000 -> 111110
   111001 -> 111010
   111010 -> 111011
   111011 -> 111001
   111100 -> 111000
   111101 -> 101000
   111110 -> 101001
   111111 -> 101011
@W:MO160 : rha_test_modulator.vhd(123) | Register bit TESTVALUE_INTERNAL[23] is always 0, optimizing ...
@W:MO160 : rha_test_modulator.vhd(123) | Register bit TESTVALUE_INTERNAL[21] is always 0, optimizing ...
@N: : rha_test_modulator.vhd(337) | Found counter in view:work.RHA_TEST_MODULATOR_RHA_TEST_MOD_3(behavioral) inst Sample_Counter[8:0]
@N: : rha_test_modulator.vhd(337) | Found counter in view:work.RHA_TEST_MODULATOR_RHA_TEST_MOD_3(behavioral) inst Channel_Counter[3:0]
@N:MO106 : rha_test_modulator.vhd(370) | Found ROM, 'DATAOUT_ADDRESS[3]', 16 words by 1 bits 
Encoding state machine SPIMACHINE_STATE[0:50] (view:work.RHA_TEST_MODULATOR_RHA_TEST_MOD_3(behavioral))
original code -> new code
   000000 -> 000000
   000001 -> 000001
   000010 -> 000011
   000011 -> 000010
   000100 -> 000110
   000101 -> 000111
   000110 -> 000101
   000111 -> 000100
   001000 -> 001100
   001001 -> 001101
   001010 -> 001111
   001011 -> 001110
   001100 -> 001010
   001101 -> 001011
   001110 -> 001001
   001111 -> 001000
   010000 -> 011000
   010001 -> 011001
   010010 -> 011011
   010011 -> 011010
   010100 -> 011110
   010101 -> 011111
   010110 -> 011101
   010111 -> 011100
   011000 -> 010100
   011001 -> 010101
   011010 -> 010111
   011011 -> 010110
   011100 -> 010010
   011101 -> 010011
   011110 -> 010001
   011111 -> 010000
   101000 -> 110000
   101001 -> 110001
   101011 -> 110011
   110000 -> 110010
   110001 -> 110110
   110010 -> 110111
   110011 -> 110101
   110100 -> 110100
   110101 -> 111100
   110110 -> 111101
   110111 -> 111111
   111000 -> 111110
   111001 -> 111010
   111010 -> 111011
   111011 -> 111001
   111100 -> 111000
   111101 -> 101000
   111110 -> 101001
   111111 -> 101011
@W:MO160 : rha_test_modulator.vhd(123) | Register bit TESTVALUE_INTERNAL[23] is always 0, optimizing ...
@N: : rha_test_modulator.vhd(337) | Found counter in view:work.RHA_TEST_MODULATOR_RHA_TEST_MOD_0_RHA_TEST_MOD_0(behavioral) inst Sample_Counter[8:0]
@N: : rha_test_modulator.vhd(337) | Found counter in view:work.RHA_TEST_MODULATOR_RHA_TEST_MOD_0_RHA_TEST_MOD_0(behavioral) inst Channel_Counter[3:0]
@N:MO106 : rha_test_modulator.vhd(370) | Found ROM, 'DATAOUT_ADDRESS[3]', 16 words by 1 bits 
Encoding state machine SPIMACHINE_STATE[0:50] (view:work.RHA_TEST_MODULATOR_RHA_TEST_MOD_0_RHA_TEST_MOD_0(behavioral))
original code -> new code
   000000 -> 000000
   000001 -> 000001
   000010 -> 000011
   000011 -> 000010
   000100 -> 000110
   000101 -> 000111
   000110 -> 000101
   000111 -> 000100
   001000 -> 001100
   001001 -> 001101
   001010 -> 001111
   001011 -> 001110
   001100 -> 001010
   001101 -> 001011
   001110 -> 001001
   001111 -> 001000
   010000 -> 011000
   010001 -> 011001
   010010 -> 011011
   010011 -> 011010
   010100 -> 011110
   010101 -> 011111
   010110 -> 011101
   010111 -> 011100
   011000 -> 010100
   011001 -> 010101
   011010 -> 010111
   011011 -> 010110
   011100 -> 010010
   011101 -> 010011
   011110 -> 010001
   011111 -> 010000
   101000 -> 110000
   101001 -> 110001
   101011 -> 110011
   110000 -> 110010
   110001 -> 110110
   110010 -> 110111
   110011 -> 110101
   110100 -> 110100
   110101 -> 111100
   110110 -> 111101
   110111 -> 111111
   111000 -> 111110
   111001 -> 111010
   111010 -> 111011
   111011 -> 111001
   111100 -> 111000
   111101 -> 101000
   111110 -> 101001
   111111 -> 101011
@W:MO160 : rha_test_modulator.vhd(123) | Register bit TESTVALUE_INTERNAL[22] is always 0, optimizing ...
@W:MO160 : rha_test_modulator.vhd(123) | Register bit TESTVALUE_INTERNAL[21] is always 0, optimizing ...
@N: : rha_test_modulator.vhd(337) | Found counter in view:work.RHA_TEST_MODULATOR_RHA_TEST_MOD_5(behavioral) inst Sample_Counter[8:0]
@N: : rha_test_modulator.vhd(337) | Found counter in view:work.RHA_TEST_MODULATOR_RHA_TEST_MOD_5(behavioral) inst Channel_Counter[3:0]
@N:MO106 : rha_test_modulator.vhd(370) | Found ROM, 'DATAOUT_ADDRESS[3]', 16 words by 1 bits 
Encoding state machine SPIMACHINE_STATE[0:50] (view:work.RHA_TEST_MODULATOR_RHA_TEST_MOD_5(behavioral))
original code -> new code
   000000 -> 000000
   000001 -> 000001
   000010 -> 000011
   000011 -> 000010
   000100 -> 000110
   000101 -> 000111
   000110 -> 000101
   000111 -> 000100
   001000 -> 001100
   001001 -> 001101
   001010 -> 001111
   001011 -> 001110
   001100 -> 001010
   001101 -> 001011
   001110 -> 001001
   001111 -> 001000
   010000 -> 011000
   010001 -> 011001
   010010 -> 011011
   010011 -> 011010
   010100 -> 011110
   010101 -> 011111
   010110 -> 011101
   010111 -> 011100
   011000 -> 010100
   011001 -> 010101
   011010 -> 010111
   011011 -> 010110
   011100 -> 010010
   011101 -> 010011
   011110 -> 010001
   011111 -> 010000
   101000 -> 110000
   101001 -> 110001
   101011 -> 110011
   110000 -> 110010
   110001 -> 110110
   110010 -> 110111
   110011 -> 110101
   110100 -> 110100
   110101 -> 111100
   110110 -> 111101
   110111 -> 111111
   111000 -> 111110
   111001 -> 111010
   111010 -> 111011
   111011 -> 111001
   111100 -> 111000
   111101 -> 101000
   111110 -> 101001
   111111 -> 101011
@W:MO160 : rha_test_modulator.vhd(123) | Register bit TESTVALUE_INTERNAL[22] is always 0, optimizing ...
@N: : rha_test_modulator.vhd(337) | Found counter in view:work.RHA_TEST_MODULATOR_RHA_TEST_MOD_0(behavioral) inst Sample_Counter[8:0]
@N: : rha_test_modulator.vhd(337) | Found counter in view:work.RHA_TEST_MODULATOR_RHA_TEST_MOD_0(behavioral) inst Channel_Counter[3:0]
@N:MO106 : rha_test_modulator.vhd(370) | Found ROM, 'DATAOUT_ADDRESS[3]', 16 words by 1 bits 
Encoding state machine SPIMACHINE_STATE[0:50] (view:work.RHA_TEST_MODULATOR_RHA_TEST_MOD_0(behavioral))
original code -> new code
   000000 -> 000000
   000001 -> 000001
   000010 -> 000011
   000011 -> 000010
   000100 -> 000110
   000101 -> 000111
   000110 -> 000101
   000111 -> 000100
   001000 -> 001100
   001001 -> 001101
   001010 -> 001111
   001011 -> 001110
   001100 -> 001010
   001101 -> 001011
   001110 -> 001001
   001111 -> 001000
   010000 -> 011000
   010001 -> 011001
   010010 -> 011011
   010011 -> 011010
   010100 -> 011110
   010101 -> 011111
   010110 -> 011101
   010111 -> 011100
   011000 -> 010100
   011001 -> 010101
   011010 -> 010111
   011011 -> 010110
   011100 -> 010010
   011101 -> 010011
   011110 -> 010001
   011111 -> 010000
   101000 -> 110000
   101001 -> 110001
   101011 -> 110011
   110000 -> 110010
   110001 -> 110110
   110010 -> 110111
   110011 -> 110101
   110100 -> 110100
   110101 -> 111100
   110110 -> 111101
   110111 -> 111111
   111000 -> 111110
   111001 -> 111010
   111010 -> 111011
   111011 -> 111001
   111100 -> 111000
   111101 -> 101000
   111110 -> 101001
   111111 -> 101011
@W:MO160 : rha_test_modulator.vhd(123) | Register bit TESTVALUE_INTERNAL[21] is always 0, optimizing ...
@N: : rha_test_modulator.vhd(337) | Found counter in view:work.RHA_TEST_MODULATOR(behavioral) inst Sample_Counter[8:0]
@N: : rha_test_modulator.vhd(337) | Found counter in view:work.RHA_TEST_MODULATOR(behavioral) inst Channel_Counter[3:0]
@N:MO106 : rha_test_modulator.vhd(370) | Found ROM, 'DATAOUT_ADDRESS[3]', 16 words by 1 bits 
Encoding state machine SPIMACHINE_STATE[0:50] (view:work.RHA_TEST_MODULATOR(behavioral))
original code -> new code
   000000 -> 000000
   000001 -> 000001
   000010 -> 000011
   000011 -> 000010
   000100 -> 000110
   000101 -> 000111
   000110 -> 000101
   000111 -> 000100
   001000 -> 001100
   001001 -> 001101
   001010 -> 001111
   001011 -> 001110
   001100 -> 001010
   001101 -> 001011
   001110 -> 001001
   001111 -> 001000
   010000 -> 011000
   010001 -> 011001
   010010 -> 011011
   010011 -> 011010
   010100 -> 011110
   010101 -> 011111
   010110 -> 011101
   010111 -> 011100
   011000 -> 010100
   011001 -> 010101
   011010 -> 010111
   011011 -> 010110
   011100 -> 010010
   011101 -> 010011
   011110 -> 010001
   011111 -> 010000
   101000 -> 110000
   101001 -> 110001
   101011 -> 110011
   110000 -> 110010
   110001 -> 110110
   110010 -> 110111
   110011 -> 110101
   110100 -> 110100
   110101 -> 111100
   110110 -> 111101
   110111 -> 111111
   111000 -> 111110
   111001 -> 111010
   111010 -> 111011
   111011 -> 111001
   111100 -> 111000
   111101 -> 101000
   111110 -> 101001
   111111 -> 101011
@W:BN132 : command_receiver.vhd(460) | Removing sequential instance MYImplant.Inst_CONTROL_NEXUS.Inst_COMMAND_RECEIVER.BITMASK_SAMPLE_TOP_INTERNAL[15],  because it is equivalent to instance MYImplant.Inst_CONTROL_NEXUS.Inst_COMMAND_RECEIVER.BITMASK_SAMPLE_INTERNAL[0]
@W:BN132 : rha_test_modulator.vhd(337) | Removing sequential instance MYImplant.Inst_DATA_ACQUISITION_BLOCK.Inst_RHA_TESTMODULE.RHA_TEST_MOD_1.TESTVALUE[21],  because it is equivalent to instance MYImplant.Inst_DATA_ACQUISITION_BLOCK.Inst_RHA_TESTMODULE.RHA_TEST_MOD_1.TESTVALUE[1]
@W:BN132 : rha_test_modulator.vhd(337) | Removing sequential instance MYImplant.Inst_DATA_ACQUISITION_BLOCK.Inst_RHA_TESTMODULE.RHA_TEST_MOD_2.TESTVALUE[22],  because it is equivalent to instance MYImplant.Inst_DATA_ACQUISITION_BLOCK.Inst_RHA_TESTMODULE.RHA_TEST_MOD_2.TESTVALUE[1]
@W:BN132 : rha_test_modulator.vhd(337) | Removing sequential instance MYImplant.Inst_DATA_ACQUISITION_BLOCK.Inst_RHA_TESTMODULE.RHA_TEST_MOD_3.TESTVALUE[22],  because it is equivalent to instance MYImplant.Inst_DATA_ACQUISITION_BLOCK.Inst_RHA_TESTMODULE.RHA_TEST_MOD_3.TESTVALUE[21]
@W:BN132 : rha_test_modulator.vhd(337) | Removing sequential instance MYImplant.Inst_DATA_ACQUISITION_BLOCK.Inst_RHA_TESTMODULE.RHA_TEST_MOD_3.TESTVALUE[21],  because it is equivalent to instance MYImplant.Inst_DATA_ACQUISITION_BLOCK.Inst_RHA_TESTMODULE.RHA_TEST_MOD_3.TESTVALUE[1]
@W:BN132 : rha_test_modulator.vhd(337) | Removing sequential instance MYImplant.Inst_DATA_ACQUISITION_BLOCK.Inst_RHA_TESTMODULE.RHA_TEST_MOD_4.TESTVALUE[23],  because it is equivalent to instance MYImplant.Inst_DATA_ACQUISITION_BLOCK.Inst_RHA_TESTMODULE.RHA_TEST_MOD_4.TESTVALUE[1]
@W:BN132 : rha_test_modulator.vhd(337) | Removing sequential instance MYImplant.Inst_DATA_ACQUISITION_BLOCK.Inst_RHA_TESTMODULE.RHA_TEST_MOD_5.TESTVALUE[23],  because it is equivalent to instance MYImplant.Inst_DATA_ACQUISITION_BLOCK.Inst_RHA_TESTMODULE.RHA_TEST_MOD_5.TESTVALUE[21]
@W:BN132 : rha_test_modulator.vhd(337) | Removing sequential instance MYImplant.Inst_DATA_ACQUISITION_BLOCK.Inst_RHA_TESTMODULE.RHA_TEST_MOD_5.TESTVALUE[21],  because it is equivalent to instance MYImplant.Inst_DATA_ACQUISITION_BLOCK.Inst_RHA_TESTMODULE.RHA_TEST_MOD_5.TESTVALUE[1]
@W:BN132 : rha_test_modulator.vhd(337) | Removing sequential instance MYImplant.Inst_DATA_ACQUISITION_BLOCK.Inst_RHA_TESTMODULE.RHA_TEST_MOD_6.TESTVALUE[23],  because it is equivalent to instance MYImplant.Inst_DATA_ACQUISITION_BLOCK.Inst_RHA_TESTMODULE.RHA_TEST_MOD_6.TESTVALUE[22]
@W:BN132 : rha_test_modulator.vhd(337) | Removing sequential instance MYImplant.Inst_DATA_ACQUISITION_BLOCK.Inst_RHA_TESTMODULE.RHA_TEST_MOD_6.TESTVALUE[22],  because it is equivalent to instance MYImplant.Inst_DATA_ACQUISITION_BLOCK.Inst_RHA_TESTMODULE.RHA_TEST_MOD_6.TESTVALUE[1]
@W:BN132 : rha_test_modulator.vhd(337) | Removing sequential instance MYImplant.Inst_DATA_ACQUISITION_BLOCK.Inst_RHA_TESTMODULE.RHA_TEST_MOD_7.TESTVALUE[23],  because it is equivalent to instance MYImplant.Inst_DATA_ACQUISITION_BLOCK.Inst_RHA_TESTMODULE.RHA_TEST_MOD_7.TESTVALUE[22]
@W:BN132 : rha_test_modulator.vhd(337) | Removing sequential instance MYImplant.Inst_DATA_ACQUISITION_BLOCK.Inst_RHA_TESTMODULE.RHA_TEST_MOD_7.TESTVALUE[22],  because it is equivalent to instance MYImplant.Inst_DATA_ACQUISITION_BLOCK.Inst_RHA_TESTMODULE.RHA_TEST_MOD_7.TESTVALUE[21]
@W:BN132 : rha_test_modulator.vhd(337) | Removing sequential instance MYImplant.Inst_DATA_ACQUISITION_BLOCK.Inst_RHA_TESTMODULE.RHA_TEST_MOD_7.TESTVALUE[21],  because it is equivalent to instance MYImplant.Inst_DATA_ACQUISITION_BLOCK.Inst_RHA_TESTMODULE.RHA_TEST_MOD_7.TESTVALUE[1]
Auto Dissolve of MYImplant.Inst_DATA_ACQUISITION_BLOCK (inst of view:work.DATA_ACQUISITION_BLOCK(behavioral))

Finished factoring (Real Time elapsed 0h:00m:02s; CPU Time elapsed 0h:00m:03s; Memory used current: 146MB peak: 146MB)

@W:BN132 : rha_test_modulator.vhd(337) | Removing sequential instance MYImplant.Inst_DATA_ACQUISITION_BLOCK.Inst_RHA_TESTMODULE.RHA_TEST_MOD_7.TESTVALUE[1],  because it is equivalent to instance MYImplant.Inst_DATA_ACQUISITION_BLOCK.Inst_RHA_TESTMODULE.RHA_TEST_MOD_6.TESTVALUE[1]
@W:BN132 : rha_test_modulator.vhd(337) | Removing sequential instance MYImplant.Inst_DATA_ACQUISITION_BLOCK.Inst_RHA_TESTMODULE.RHA_TEST_MOD_6.TESTVALUE[1],  because it is equivalent to instance MYImplant.Inst_DATA_ACQUISITION_BLOCK.Inst_RHA_TESTMODULE.RHA_TEST_MOD_5.TESTVALUE[1]
@W:BN132 : rha_test_modulator.vhd(337) | Removing sequential instance MYImplant.Inst_DATA_ACQUISITION_BLOCK.Inst_RHA_TESTMODULE.RHA_TEST_MOD_5.TESTVALUE[1],  because it is equivalent to instance MYImplant.Inst_DATA_ACQUISITION_BLOCK.Inst_RHA_TESTMODULE.RHA_TEST_MOD_4.TESTVALUE[1]
@W:BN132 : rha_test_modulator.vhd(337) | Removing sequential instance MYImplant.Inst_DATA_ACQUISITION_BLOCK.Inst_RHA_TESTMODULE.RHA_TEST_MOD_4.TESTVALUE[1],  because it is equivalent to instance MYImplant.Inst_DATA_ACQUISITION_BLOCK.Inst_RHA_TESTMODULE.RHA_TEST_MOD_3.TESTVALUE[1]
@W:BN132 : rha_test_modulator.vhd(337) | Removing sequential instance MYImplant.Inst_DATA_ACQUISITION_BLOCK.Inst_RHA_TESTMODULE.RHA_TEST_MOD_3.TESTVALUE[1],  because it is equivalent to instance MYImplant.Inst_DATA_ACQUISITION_BLOCK.Inst_RHA_TESTMODULE.RHA_TEST_MOD_2.TESTVALUE[1]
@W:BN132 : rha_test_modulator.vhd(337) | Removing sequential instance MYImplant.Inst_DATA_ACQUISITION_BLOCK.Inst_RHA_TESTMODULE.RHA_TEST_MOD_2.TESTVALUE[1],  because it is equivalent to instance MYImplant.Inst_DATA_ACQUISITION_BLOCK.Inst_RHA_TESTMODULE.RHA_TEST_MOD_1.TESTVALUE[1]
@W:BN132 : rha_test_modulator.vhd(337) | Removing sequential instance MYImplant.Inst_DATA_ACQUISITION_BLOCK.Inst_RHA_TESTMODULE.RHA_TEST_MOD_1.TESTVALUE[1],  because it is equivalent to instance MYImplant.Inst_DATA_ACQUISITION_BLOCK.Inst_RHA_TESTMODULE.RHA_TEST_MOD_0.TESTVALUE[1]

Finished gated-clock and generated-clock conversion (Real Time elapsed 0h:00m:02s; CPU Time elapsed 0h:00m:03s; Memory used current: 144MB peak: 148MB)


Finished generic timing optimizations - Pass 1 (Real Time elapsed 0h:00m:04s; CPU Time elapsed 0h:00m:04s; Memory used current: 139MB peak: 160MB)


Starting Early Timing Optimization (Real Time elapsed 0h:00m:04s; CPU Time elapsed 0h:00m:04s; Memory used current: 140MB peak: 160MB)


Finished Early Timing Optimization (Real Time elapsed 0h:00m:05s; CPU Time elapsed 0h:00m:05s; Memory used current: 141MB peak: 160MB)


Finished generic timing optimizations - Pass 2 (Real Time elapsed 0h:00m:05s; CPU Time elapsed 0h:00m:05s; Memory used current: 139MB peak: 160MB)


Finished preparing to map (Real Time elapsed 0h:00m:05s; CPU Time elapsed 0h:00m:06s; Memory used current: 139MB peak: 160MB)


Finished technology mapping (Real Time elapsed 0h:00m:06s; CPU Time elapsed 0h:00m:07s; Memory used current: 163MB peak: 216MB)


High Fanout Net Report
**********************

Driver Instance / Pin Name                                                                                Fanout, notes                     
--------------------------------------------------------------------------------------------------------------------------------------------
MYImplant.ResetRHAandFIFO_DELAYED / Q                                                                     1386 : 1384 asynchronous set/reset
MYImplant.Inst_DATA_ACQUISITION_BLOCK.Inst_RHA_TESTMODULE.RHA_TEST_MOD_3.SPIMACHINE_STATE[4] / Q          25                                
MYImplant.Inst_DATA_ACQUISITION_BLOCK.Inst_RHA_TESTMODULE.RHA_TEST_MOD_2.SPIMACHINE_STATE[4] / Q          25                                
MYImplant.Inst_DATA_ACQUISITION_BLOCK.Inst_RHA_TESTMODULE.RHA_TEST_MOD_1.SPIMACHINE_STATE[0] / Q          26                                
MYImplant.Inst_DATA_ACQUISITION_BLOCK.Inst_RHA_TESTMODULE.RHA_TEST_MOD_1.SPIMACHINE_STATE[1] / Q          28                                
MYImplant.Inst_DATA_ACQUISITION_BLOCK.Inst_RHA_TO_ZL_CONVERTER.SPIMACHINE_STATE[0] / Q                    40                                
MYImplant.Inst_DATA_ACQUISITION_BLOCK.Inst_RHA_ARRAY.COPY_VALID_DATA.un4_readrequest / Y                  154                               
MYImplant.Inst_CONTROL_NEXUS.Inst_ZARLINK_CONNECT_INIT.GetCommand_DATA_INTERNAL[0] / Q                    30                                
MYImplant.Inst_CONTROL_NEXUS.Inst_ZARLINK_CONNECT_INIT.GetCommand_DATA_INTERNAL[1] / Q                    30                                
MYImplant.Inst_CONTROL_NEXUS.Inst_ZARLINK_CONNECT_INIT.GetCommand_DATA_INTERNAL[2] / Q                    29                                
MYImplant.Inst_CONTROL_NEXUS.Inst_ZARLINK_CONNECT_INIT.GetCommand_DATA_INTERNAL[4] / Q                    25                                
MYImplant.Inst_CONTROL_NEXUS.Inst_ZARLINK_CONNECT_INIT.GetCommand_DATA_INTERNAL[6] / Q                    28                                
MYImplant.Inst_CONTROL_NEXUS.Inst_ZARLINK_CONNECT_INIT.FSM_STATE[1] / Q                                   33                                
MYImplant.Inst_CONTROL_NEXUS.Inst_ZARLINK_CONNECT_INIT.FSM_STATE[2] / Q                                   29                                
MYImplant.Inst_CONTROL_NEXUS.Inst_ZARLINK_CONNECT_INIT.FSM_STATE[3] / Q                                   26                                
MYImplant.Inst_CONTROL_NEXUS.Inst_Zarlink_SPI_Module.FSM_STATE[3] / Q                                     27                                
Reset_pad / Y                                                                                             463 : 460 asynchronous set/reset  
MYImplant.Inst_CONTROL_NEXUS.Inst_COMMAND_RECEIVER.CMD_RECEIVER.un1_fifo_out_valid / Y                    94                                
MYImplant.Inst_DATA_ACQUISITION_BLOCK.Inst_RHA_ARRAY.COPY_VALID_DATA.un52_gated_valid / Y                 129                               
MYImplant.Inst_DATA_ACQUISITION_BLOCK.Inst_RHA_ARRAY.COPY_VALID_DATA.un48_gated_valid / Y                 258                               
MYImplant.Inst_CONTROL_NEXUS.Inst_COMMAND_RECEIVER.un1_SPIMACHINE_STATE_i_a2 / Y                          140                               
MYImplant.Inst_DATA_ACQUISITION_BLOCK.Inst_RHA_TO_ZL_CONVERTER.un1_NewBlock_Off_1_sqmuxa_i / Y            48                                
MYImplant.Inst_DATA_ACQUISITION_BLOCK.Inst_RHA_TO_ZL_CONVERTER.SPIMACHINE_STATE_s18_0_a4_0_a2 / Y         34                                
MYImplant.Inst_DATA_ACQUISITION_BLOCK.Inst_RHA_TO_ZL_CONVERTER.SPIMACHINE_STATE_s4_0_a2 / Y               31                                
MYImplant.Inst_DATA_ACQUISITION_BLOCK.Inst_RHA_TO_ZL_CONVERTER.SPIMACHINE_STATE_ns_0_a2_1_0_a2[3] / Y     25                                
MYImplant.Inst_CONTROL_NEXUS.Inst_ZARLINK_CONNECT_INIT.Zarlink_ResetCounter_n0_0_a4_i_o2_0_o3 / Y         26                                
MYImplant.Inst_CONTROL_NEXUS.Inst_ZARLINK_CONNECT_INIT.FSM.fsm_state155_i_i_a2_i / Y                      32                                
============================================================================================================================================

@N:FP130 :  | Promoting Net MYImplant.Clk on CLKINT  MYImplant.Clk_inferred_clock  
@N:FP130 :  | Promoting Net Reset_c on CLKBUF  Reset_pad  
@N:FP130 :  | Promoting Net MYImplant.ResetRHAandFIFO_DELAYED on CLKINT  I_740  
@N:FP130 :  | Promoting Net MYImplant.Clk_Reduced on CLKINT  I_741  
@N:FP130 :  | Promoting Net MYImplant.Inst_DATA_ACQUISITION_BLOCK.Inst_RHA_ARRAY.COPY_VALID_DATA\.un48_gated_valid on CLKINT  I_742  
@N:FP130 :  | Promoting Net MYImplant.Inst_DATA_ACQUISITION_BLOCK.Inst_RHA_ARRAY.COPY_VALID_DATA\.un4_readrequest on CLKINT  I_743  

Finished technology timing optimizations and critical path resynthesis (Real Time elapsed 0h:00m:07s; CPU Time elapsed 0h:00m:08s; Memory used current: 163MB peak: 216MB)

Replicating Combinational Instance MYImplant.Inst_CONTROL_NEXUS.Inst_ZARLINK_CONNECT_INIT.FSM.fsm_state155_i_i_a2_i, fanout 32 segments 2
Replicating Combinational Instance MYImplant.Inst_CONTROL_NEXUS.Inst_ZARLINK_CONNECT_INIT.Zarlink_ResetCounter_n0_0_a4_i_o2_0_o3, fanout 26 segments 2
Replicating Combinational Instance MYImplant.Inst_DATA_ACQUISITION_BLOCK.Inst_RHA_TO_ZL_CONVERTER.SPIMACHINE_STATE_ns_0_a2_1_0_a2[3], fanout 25 segments 2
Replicating Combinational Instance MYImplant.Inst_DATA_ACQUISITION_BLOCK.Inst_RHA_TO_ZL_CONVERTER.SPIMACHINE_STATE_s4_0_a2, fanout 31 segments 2
Replicating Combinational Instance MYImplant.Inst_DATA_ACQUISITION_BLOCK.Inst_RHA_TO_ZL_CONVERTER.SPIMACHINE_STATE_s18_0_a4_0_a2, fanout 34 segments 2
Replicating Combinational Instance MYImplant.Inst_DATA_ACQUISITION_BLOCK.Inst_RHA_TO_ZL_CONVERTER.un1_NewBlock_Off_1_sqmuxa_i, fanout 48 segments 2
Replicating Combinational Instance MYImplant.Inst_CONTROL_NEXUS.Inst_COMMAND_RECEIVER.un1_SPIMACHINE_STATE_i_a2, fanout 140 segments 6
Replicating Combinational Instance MYImplant.Inst_DATA_ACQUISITION_BLOCK.Inst_RHA_ARRAY.COPY_VALID_DATA.un52_gated_valid, fanout 129 segments 6
Replicating Combinational Instance MYImplant.Inst_CONTROL_NEXUS.Inst_COMMAND_RECEIVER.CMD_RECEIVER.un1_fifo_out_valid, fanout 94 segments 4
Replicating Sequential Instance MYImplant.Inst_CONTROL_NEXUS.Inst_Zarlink_SPI_Module.FSM_STATE[3], fanout 27 segments 2
Replicating Sequential Instance MYImplant.Inst_CONTROL_NEXUS.Inst_ZARLINK_CONNECT_INIT.FSM_STATE[3], fanout 26 segments 2
Replicating Sequential Instance MYImplant.Inst_CONTROL_NEXUS.Inst_ZARLINK_CONNECT_INIT.FSM_STATE[2], fanout 29 segments 2
Replicating Sequential Instance MYImplant.Inst_CONTROL_NEXUS.Inst_ZARLINK_CONNECT_INIT.FSM_STATE[1], fanout 34 segments 2
Replicating Sequential Instance MYImplant.Inst_CONTROL_NEXUS.Inst_ZARLINK_CONNECT_INIT.GetCommand_DATA_INTERNAL[6], fanout 28 segments 2
Replicating Sequential Instance MYImplant.Inst_CONTROL_NEXUS.Inst_ZARLINK_CONNECT_INIT.GetCommand_DATA_INTERNAL[4], fanout 25 segments 2
Replicating Sequential Instance MYImplant.Inst_CONTROL_NEXUS.Inst_ZARLINK_CONNECT_INIT.GetCommand_DATA_INTERNAL[2], fanout 29 segments 2
Replicating Sequential Instance MYImplant.Inst_CONTROL_NEXUS.Inst_ZARLINK_CONNECT_INIT.GetCommand_DATA_INTERNAL[1], fanout 30 segments 2
Replicating Sequential Instance MYImplant.Inst_CONTROL_NEXUS.Inst_ZARLINK_CONNECT_INIT.GetCommand_DATA_INTERNAL[0], fanout 30 segments 2
Replicating Sequential Instance MYImplant.Inst_DATA_ACQUISITION_BLOCK.Inst_RHA_TO_ZL_CONVERTER.SPIMACHINE_STATE[0], fanout 41 segments 2
Replicating Sequential Instance MYImplant.Inst_DATA_ACQUISITION_BLOCK.Inst_RHA_TESTMODULE.RHA_TEST_MOD_1.SPIMACHINE_STATE[1], fanout 28 segments 2
Replicating Sequential Instance MYImplant.Inst_DATA_ACQUISITION_BLOCK.Inst_RHA_TESTMODULE.RHA_TEST_MOD_1.SPIMACHINE_STATE[0], fanout 26 segments 2
Replicating Sequential Instance MYImplant.Inst_DATA_ACQUISITION_BLOCK.Inst_RHA_TESTMODULE.RHA_TEST_MOD_2.SPIMACHINE_STATE[4], fanout 25 segments 2
Replicating Sequential Instance MYImplant.Inst_DATA_ACQUISITION_BLOCK.Inst_RHA_TESTMODULE.RHA_TEST_MOD_3.SPIMACHINE_STATE[4], fanout 25 segments 2
Replicating Sequential Instance MYImplant.Inst_CONTROL_NEXUS.Inst_ZARLINK_CONNECT_INIT.FSM_STATE[0], fanout 25 segments 2

Added 0 Buffers
Added 34 Cells via replication
	Added 15 Sequential Cells via replication
	Added 19 Combinational Cells via replication

Finished restoring hierarchy (Real Time elapsed 0h:00m:13s; CPU Time elapsed 0h:00m:13s; Memory used current: 164MB peak: 216MB)


#### START OF CLOCK OPTIMIZATION REPORT #####[

Clock optimization not enabled
1 non-gated/non-generated clock tree(s) driving 4 clock pin(s) of sequential element(s)
3 gated/generated clock tree(s) driving 1873 clock pin(s) of sequential element(s)
0 instances converted, 1873 sequential instances remain driven by gated/generated clocks

=========================== Non-Gated/Non-Generated Clocks ============================
Clock Tree ID     Driving Element     Drive Element Type     Fanout     Sample Instance
---------------------------------------------------------------------------------------
ClockId0004        Clk                 port                   4          MYImplant.Clk  
=======================================================================================
========================================================================================================================================= Gated/Generated Clocks ==========================================================================================================================================
Clock Tree ID     Driving Element                                                                     Drive Element Type     Fanout     Sample Instance                                                                            Explanation                                                             
-----------------------------------------------------------------------------------------------------------------------------------------------------------------------------------------------------------------------------------------------------------------------------------------------------------
ClockId0001        MYImplant.Inst_CONTROL_NEXUS.Inst_COMMAND_RECEIVER.Clk_Reduced_Internal_RNIFABI     AO1                    969        MYImplant.Inst_DATA_ACQUISITION_BLOCK.Inst_RHA_TESTMODULE.RHA_TEST_MOD_7.TESTVALUE[24]     Clock conversion disabled                                               
ClockId0002        MYImplant.Clk                                                                       DFN1C0                 862        MYImplant.ResetRHAandFIFO_DELAYED                                                          No generated or derived clock directive on output of sequential instance
ClockId0003        MYImplant.Inst_CONTROL_NEXUS.Inst_COMMAND_RECEIVER.Clk_4MHz                         DFN1C0                 42         MYImplant.Inst_CONTROL_NEXUS.Inst_Zarlink_SPI_Module.Zarlink_Adress_Copy[7]                No generated or derived clock directive on output of sequential instance
===========================================================================================================================================================================================================================================================================================================


##### END OF CLOCK OPTIMIZATION REPORT ######]

Writing Analyst data base C:\Users\ITP\Desktop\IGLOO\IGLOO_RHA\synthesis\IGLOO_TOP.srm

Finished Writing Netlist Databases (Real Time elapsed 0h:00m:14s; CPU Time elapsed 0h:00m:14s; Memory used current: 160MB peak: 216MB)

Writing EDIF Netlist and constraint files
I-2013.09M-SP1 

Finished Writing EDIF Netlist and constraint files (Real Time elapsed 0h:00m:14s; CPU Time elapsed 0h:00m:15s; Memory used current: 163MB peak: 216MB)

Found clock IGLOO_TOP|Clk with period 41.67ns 
@W:MT420 :  | Found inferred clock IMPLANT_TOP|Clk_inferred_clock with period 10.00ns. Please declare a user-defined clock on object "n:MYImplant.Clk" 

@W:MT420 :  | Found inferred clock COMMAND_RECEIVER|Clk_4MHz_inferred_clock with period 10.00ns. Please declare a user-defined clock on object "n:MYImplant.Inst_CONTROL_NEXUS.Inst_COMMAND_RECEIVER.Clk_4MHz" 


##### START OF TIMING REPORT #####[
# Timing Report written on Fri Sep 05 17:35:30 2014
#


Top view:               IGLOO_TOP
Library name:           IGLOO_V2
Operating conditions:   COMWCSTD ( T = 70.0, V = 1.14, P = 3.70, tree_type = balanced_tree )
Requested Frequency:    24.0 MHz
Wire load mode:         top
Wire load model:        igloo
Paths requested:        5
Constraint File(s):    C:\Users\ITP\Desktop\IGLOO\IGLOO_RHA\constraint\IGLOO.sdc
                       
@N:MT320 :  | Timing report estimates place and route data. Please look at the place and route timing report for final timing. 

@N:MT322 :  | Clock constraints cover only FF-to-FF paths associated with the clock. 


Performance Summary 
*******************


Worst slack in design: -28.531

                                             Requested     Estimated     Requested     Estimated                 Clock        Clock              
Starting Clock                               Frequency     Frequency     Period        Period        Slack       Type         Group              
-------------------------------------------------------------------------------------------------------------------------------------------------
COMMAND_RECEIVER|Clk_4MHz_inferred_clock     100.0 MHz     34.3 MHz      10.000        29.175        -19.175     inferred     Inferred_clkgroup_1
IGLOO_TOP|Clk                                24.0 MHz      81.8 MHz      41.666        12.228        29.438      declared     default_clkgroup   
IMPLANT_TOP|Clk_inferred_clock               100.0 MHz     26.0 MHz      10.000        38.531        -28.531     inferred     Inferred_clkgroup_0
=================================================================================================================================================
@W:MT548 : igloo.sdc(3) | Source for clock MYImplant/Clk:Q not found in netlist
@W:MT548 : igloo.sdc(5) | Source for clock MYImplant/Inst_CONTROL_NEXUS/Inst_COMMAND_RECEIVER/TestMode_INTERAL:Q not found in netlist


Clock Relationships
*******************

Clocks                                                                              |    rise  to  rise     |    fall  to  fall   |    rise  to  fall   |    fall  to  rise 
----------------------------------------------------------------------------------------------------------------------------------------------------------------------------
Starting                                  Ending                                    |  constraint  slack    |  constraint  slack  |  constraint  slack  |  constraint  slack
----------------------------------------------------------------------------------------------------------------------------------------------------------------------------
IGLOO_TOP|Clk                             IGLOO_TOP|Clk                             |  41.666      29.438   |  No paths    -      |  No paths    -      |  No paths    -    
IMPLANT_TOP|Clk_inferred_clock            IMPLANT_TOP|Clk_inferred_clock            |  10.000      -28.531  |  No paths    -      |  No paths    -      |  No paths    -    
IMPLANT_TOP|Clk_inferred_clock            COMMAND_RECEIVER|Clk_4MHz_inferred_clock  |  Diff grp    -        |  No paths    -      |  No paths    -      |  No paths    -    
COMMAND_RECEIVER|Clk_4MHz_inferred_clock  IMPLANT_TOP|Clk_inferred_clock            |  Diff grp    -        |  No paths    -      |  No paths    -      |  No paths    -    
COMMAND_RECEIVER|Clk_4MHz_inferred_clock  COMMAND_RECEIVER|Clk_4MHz_inferred_clock  |  10.000      -19.175  |  No paths    -      |  No paths    -      |  No paths    -    
============================================================================================================================================================================
 Note: 'No paths' indicates there are no paths in the design for that pair of clock edges.
       'Diff grp' indicates that paths exist but the starting clock and ending clock are in different clock groups.


Interface Information 
*********************

No IO constraint found


====================================
Detailed Report for Clock: COMMAND_RECEIVER|Clk_4MHz_inferred_clock
====================================


Starting Points with Worst Slack
********************************

                                                                        Starting                                                                             Arrival            
Instance                                                                Reference                                    Type         Pin     Net                Time        Slack  
                                                                        Clock                                                                                                   
--------------------------------------------------------------------------------------------------------------------------------------------------------------------------------
MYImplant.Inst_CONTROL_NEXUS.Inst_Zarlink_SPI_Module.FSM_STATE[4]       COMMAND_RECEIVER|Clk_4MHz_inferred_clock     DFN1C0       Q       FSM_STATE[4]       1.771       -19.175
MYImplant.Inst_CONTROL_NEXUS.Inst_Zarlink_SPI_Module.FSM_STATE[5]       COMMAND_RECEIVER|Clk_4MHz_inferred_clock     DFN1C0       Q       FSM_STATE[5]       1.771       -18.365
MYImplant.Inst_CONTROL_NEXUS.Inst_Zarlink_SPI_Module.FSM_STATE[2]       COMMAND_RECEIVER|Clk_4MHz_inferred_clock     DFN1C0       Q       FSM_STATE[2]       1.771       -17.538
MYImplant.Inst_CONTROL_NEXUS.Inst_Zarlink_SPI_Module.FSM_STATE[0]       COMMAND_RECEIVER|Clk_4MHz_inferred_clock     DFN1C0       Q       FSM_STATE[0]       1.771       -16.974
MYImplant.Inst_CONTROL_NEXUS.Inst_Zarlink_SPI_Module.FSM_STATE[3]       COMMAND_RECEIVER|Clk_4MHz_inferred_clock     DFN1C0       Q       FSM_STATE[3]       1.771       -16.912
MYImplant.Inst_CONTROL_NEXUS.Inst_Zarlink_SPI_Module.FSM_STATE[1]       COMMAND_RECEIVER|Clk_4MHz_inferred_clock     DFN1C0       Q       FSM_STATE[1]       1.771       -15.045
MYImplant.Inst_CONTROL_NEXUS.Inst_Zarlink_SPI_Module.FSM_STATE_0[3]     COMMAND_RECEIVER|Clk_4MHz_inferred_clock     DFN1C0       Q       FSM_STATE_0[3]     1.771       -8.684 
MYImplant.Inst_CONTROL_NEXUS.Inst_Zarlink_SPI_Module.Counter[0]         COMMAND_RECEIVER|Clk_4MHz_inferred_clock     DFN1E1C0     Q       Counter[0]         1.771       -7.883 
MYImplant.Inst_CONTROL_NEXUS.Inst_Zarlink_SPI_Module.Counter[2]         COMMAND_RECEIVER|Clk_4MHz_inferred_clock     DFN1E1C0     Q       Counter[2]         1.771       -7.202 
MYImplant.Inst_CONTROL_NEXUS.Inst_Zarlink_SPI_Module.Counter[1]         COMMAND_RECEIVER|Clk_4MHz_inferred_clock     DFN1E1C0     Q       Counter[1]         1.771       -6.709 
================================================================================================================================================================================


Ending Points with Worst Slack
******************************

                                                                                  Starting                                                                               Required            
Instance                                                                          Reference                                    Type         Pin     Net                  Time         Slack  
                                                                                  Clock                                                                                                      
---------------------------------------------------------------------------------------------------------------------------------------------------------------------------------------------
MYImplant.Inst_CONTROL_NEXUS.Inst_Zarlink_SPI_Module.SPI_SDI                      COMMAND_RECEIVER|Clk_4MHz_inferred_clock     DFN1C0       D       SPI_SDI_17           8.622        -19.175
MYImplant.Inst_CONTROL_NEXUS.Inst_Zarlink_SPI_Module.FSM_STATE[1]                 COMMAND_RECEIVER|Clk_4MHz_inferred_clock     DFN1C0       D       FSM_STATE_ns[1]      8.622        -14.719
MYImplant.Inst_CONTROL_NEXUS.Inst_Zarlink_SPI_Module.Zarlink_RequireValid_Out     COMMAND_RECEIVER|Clk_4MHz_inferred_clock     DFN1C0       D       N_7                  8.705        -14.231
MYImplant.Inst_CONTROL_NEXUS.Inst_Zarlink_SPI_Module.SPI_ZL_CS                    COMMAND_RECEIVER|Clk_4MHz_inferred_clock     DFN1P0       D       SPI_ZL_CS_2          8.705        -13.930
MYImplant.Inst_CONTROL_NEXUS.Inst_Zarlink_SPI_Module.Zarlink_Wait                 COMMAND_RECEIVER|Clk_4MHz_inferred_clock     DFN1C0       D       Zarlink_Wait_RNO     8.622        -13.730
MYImplant.Inst_CONTROL_NEXUS.Inst_Zarlink_SPI_Module.Zarlink_Data_Out_Copy[0]     COMMAND_RECEIVER|Clk_4MHz_inferred_clock     DFN1E0C0     E       un1_N_5_mux_0        8.956        -13.500
MYImplant.Inst_CONTROL_NEXUS.Inst_Zarlink_SPI_Module.Zarlink_Data_Out_Copy[1]     COMMAND_RECEIVER|Clk_4MHz_inferred_clock     DFN1E0C0     E       un1_N_5_mux_0        8.956        -13.500
MYImplant.Inst_CONTROL_NEXUS.Inst_Zarlink_SPI_Module.Zarlink_Data_Out_Copy[2]     COMMAND_RECEIVER|Clk_4MHz_inferred_clock     DFN1E0C0     E       un1_N_5_mux_0        8.956        -13.500
MYImplant.Inst_CONTROL_NEXUS.Inst_Zarlink_SPI_Module.Zarlink_Data_Out_Copy[3]     COMMAND_RECEIVER|Clk_4MHz_inferred_clock     DFN1E0C0     E       un1_N_5_mux_0        8.956        -13.500
MYImplant.Inst_CONTROL_NEXUS.Inst_Zarlink_SPI_Module.Zarlink_Data_Out_Copy[4]     COMMAND_RECEIVER|Clk_4MHz_inferred_clock     DFN1E0C0     E       un1_N_5_mux_0        8.956        -13.500
=============================================================================================================================================================================================


Worst Path Information
View Worst Path in Analyst
***********************


Path information for path number 1: 
      Requested Period:                      10.000
    - Setup time:                            1.378
    + Clock delay at ending point:           0.000 (ideal)
    = Required time:                         8.622

    - Propagation time:                      27.797
    - Clock delay at starting point:         0.000 (ideal)
    = Slack (non-critical) :                 -19.175

    Number of logic level(s):                6
    Starting point:                          MYImplant.Inst_CONTROL_NEXUS.Inst_Zarlink_SPI_Module.FSM_STATE[4] / Q
    Ending point:                            MYImplant.Inst_CONTROL_NEXUS.Inst_Zarlink_SPI_Module.SPI_SDI / D
    The start point is clocked by            COMMAND_RECEIVER|Clk_4MHz_inferred_clock [rising] on pin CLK
    The end   point is clocked by            COMMAND_RECEIVER|Clk_4MHz_inferred_clock [rising] on pin CLK

Instance / Net                                                                            Pin      Pin               Arrival     No. of    
Name                                                                           Type       Name     Dir     Delay     Time        Fan Out(s)
-------------------------------------------------------------------------------------------------------------------------------------------
MYImplant.Inst_CONTROL_NEXUS.Inst_Zarlink_SPI_Module.FSM_STATE[4]              DFN1C0     Q        Out     1.771     1.771       -         
FSM_STATE[4]                                                                   Net        -        -       5.855     -           23        
MYImplant.Inst_CONTROL_NEXUS.Inst_Zarlink_SPI_Module.FSM_STATE_RNIV92J[4]      NOR2A      A        In      -         7.626       -         
MYImplant.Inst_CONTROL_NEXUS.Inst_Zarlink_SPI_Module.FSM_STATE_RNIV92J[4]      NOR2A      Y        Out     1.508     9.134       -         
N_116                                                                          Net        -        -       4.105     -           10        
MYImplant.Inst_CONTROL_NEXUS.Inst_Zarlink_SPI_Module.FSM_STATE_RNIQF461[3]     OR2A       A        In      -         13.239      -         
MYImplant.Inst_CONTROL_NEXUS.Inst_Zarlink_SPI_Module.FSM_STATE_RNIQF461[3]     OR2A       Y        Out     1.119     14.358      -         
N_52                                                                           Net        -        -       4.009     -           9         
MYImplant.Inst_CONTROL_NEXUS.Inst_Zarlink_SPI_Module.SPI_SDI_RNO_28            NOR2A      B        In      -         18.368      -         
MYImplant.Inst_CONTROL_NEXUS.Inst_Zarlink_SPI_Module.SPI_SDI_RNO_28            NOR2A      Y        Out     0.927     19.295      -         
N_109                                                                          Net        -        -       0.773     -           1         
MYImplant.Inst_CONTROL_NEXUS.Inst_Zarlink_SPI_Module.SPI_SDI_RNO_11            OR3        C        In      -         20.067      -         
MYImplant.Inst_CONTROL_NEXUS.Inst_Zarlink_SPI_Module.SPI_SDI_RNO_11            OR3        Y        Out     1.804     21.871      -         
SPI_SDI_17_iv_0_10                                                             Net        -        -       0.773     -           1         
MYImplant.Inst_CONTROL_NEXUS.Inst_Zarlink_SPI_Module.SPI_SDI_RNO_2             OR3        C        In      -         22.644      -         
MYImplant.Inst_CONTROL_NEXUS.Inst_Zarlink_SPI_Module.SPI_SDI_RNO_2             OR3        Y        Out     1.804     24.448      -         
SPI_SDI_17_iv_0_13                                                             Net        -        -       0.773     -           1         
MYImplant.Inst_CONTROL_NEXUS.Inst_Zarlink_SPI_Module.SPI_SDI_RNO               OR3        C        In      -         25.221      -         
MYImplant.Inst_CONTROL_NEXUS.Inst_Zarlink_SPI_Module.SPI_SDI_RNO               OR3        Y        Out     1.804     27.025      -         
SPI_SDI_17                                                                     Net        -        -       0.773     -           1         
MYImplant.Inst_CONTROL_NEXUS.Inst_Zarlink_SPI_Module.SPI_SDI                   DFN1C0     D        In      -         27.797      -         
===========================================================================================================================================
Total path delay (propagation time + setup) of 29.175 is 12.115(41.5%) logic and 17.060(58.5%) route.
Path delay compensated for clock skew. Clock skew is added to clock-to-out value, and is subtracted from setup time value


Path information for path number 2: 
      Requested Period:                      10.000
    - Setup time:                            1.295
    + Clock delay at ending point:           0.000 (ideal)
    = Required time:                         8.705

    - Propagation time:                      27.071
    - Clock delay at starting point:         0.000 (ideal)
    = Slack (non-critical) :                 -18.365

    Number of logic level(s):                6
    Starting point:                          MYImplant.Inst_CONTROL_NEXUS.Inst_Zarlink_SPI_Module.FSM_STATE[5] / Q
    Ending point:                            MYImplant.Inst_CONTROL_NEXUS.Inst_Zarlink_SPI_Module.SPI_SDI / D
    The start point is clocked by            COMMAND_RECEIVER|Clk_4MHz_inferred_clock [rising] on pin CLK
    The end   point is clocked by            COMMAND_RECEIVER|Clk_4MHz_inferred_clock [rising] on pin CLK

Instance / Net                                                                            Pin      Pin               Arrival     No. of    
Name                                                                           Type       Name     Dir     Delay     Time        Fan Out(s)
-------------------------------------------------------------------------------------------------------------------------------------------
MYImplant.Inst_CONTROL_NEXUS.Inst_Zarlink_SPI_Module.FSM_STATE[5]              DFN1C0     Q        Out     1.771     1.771       -         
FSM_STATE[5]                                                                   Net        -        -       5.926     -           24        
MYImplant.Inst_CONTROL_NEXUS.Inst_Zarlink_SPI_Module.FSM_STATE_RNIV92J[4]      NOR2A      B        In      -         7.697       -         
MYImplant.Inst_CONTROL_NEXUS.Inst_Zarlink_SPI_Module.FSM_STATE_RNIV92J[4]      NOR2A      Y        Out     0.977     8.674       -         
N_116                                                                          Net        -        -       4.105     -           10        
MYImplant.Inst_CONTROL_NEXUS.Inst_Zarlink_SPI_Module.FSM_STATE_RNIQF461[3]     OR2A       A        In      -         12.780      -         
MYImplant.Inst_CONTROL_NEXUS.Inst_Zarlink_SPI_Module.FSM_STATE_RNIQF461[3]     OR2A       Y        Out     1.290     14.070      -         
N_52                                                                           Net        -        -       4.009     -           9         
MYImplant.Inst_CONTROL_NEXUS.Inst_Zarlink_SPI_Module.SPI_SDI_RNO_28            NOR2A      B        In      -         18.079      -         
MYImplant.Inst_CONTROL_NEXUS.Inst_Zarlink_SPI_Module.SPI_SDI_RNO_28            NOR2A      Y        Out     0.977     19.056      -         
N_109                                                                          Net        -        -       0.773     -           1         
MYImplant.Inst_CONTROL_NEXUS.Inst_Zarlink_SPI_Module.SPI_SDI_RNO_11            OR3        C        In      -         19.829      -         
MYImplant.Inst_CONTROL_NEXUS.Inst_Zarlink_SPI_Module.SPI_SDI_RNO_11            OR3        Y        Out     1.641     21.470      -         
SPI_SDI_17_iv_0_10                                                             Net        -        -       0.773     -           1         
MYImplant.Inst_CONTROL_NEXUS.Inst_Zarlink_SPI_Module.SPI_SDI_RNO_2             OR3        C        In      -         22.243      -         
MYImplant.Inst_CONTROL_NEXUS.Inst_Zarlink_SPI_Module.SPI_SDI_RNO_2             OR3        Y        Out     1.641     23.884      -         
SPI_SDI_17_iv_0_13                                                             Net        -        -       0.773     -           1         
MYImplant.Inst_CONTROL_NEXUS.Inst_Zarlink_SPI_Module.SPI_SDI_RNO               OR3        C        In      -         24.657      -         
MYImplant.Inst_CONTROL_NEXUS.Inst_Zarlink_SPI_Module.SPI_SDI_RNO               OR3        Y        Out     1.641     26.298      -         
SPI_SDI_17                                                                     Net        -        -       0.773     -           1         
MYImplant.Inst_CONTROL_NEXUS.Inst_Zarlink_SPI_Module.SPI_SDI                   DFN1C0     D        In      -         27.071      -         
===========================================================================================================================================
Total path delay (propagation time + setup) of 28.365 is 11.234(39.6%) logic and 17.131(60.4%) route.
Path delay compensated for clock skew. Clock skew is added to clock-to-out value, and is subtracted from setup time value


Path information for path number 3: 
      Requested Period:                      10.000
    - Setup time:                            1.295
    + Clock delay at ending point:           0.000 (ideal)
    = Required time:                         8.705

    - Propagation time:                      26.244
    - Clock delay at starting point:         0.000 (ideal)
    = Slack (non-critical) :                 -17.538

    Number of logic level(s):                6
    Starting point:                          MYImplant.Inst_CONTROL_NEXUS.Inst_Zarlink_SPI_Module.FSM_STATE[2] / Q
    Ending point:                            MYImplant.Inst_CONTROL_NEXUS.Inst_Zarlink_SPI_Module.SPI_SDI / D
    The start point is clocked by            COMMAND_RECEIVER|Clk_4MHz_inferred_clock [rising] on pin CLK
    The end   point is clocked by            COMMAND_RECEIVER|Clk_4MHz_inferred_clock [rising] on pin CLK

Instance / Net                                                                             Pin      Pin               Arrival     No. of    
Name                                                                            Type       Name     Dir     Delay     Time        Fan Out(s)
--------------------------------------------------------------------------------------------------------------------------------------------
MYImplant.Inst_CONTROL_NEXUS.Inst_Zarlink_SPI_Module.FSM_STATE[2]               DFN1C0     Q        Out     1.771     1.771       -         
FSM_STATE[2]                                                                    Net        -        -       5.855     -           23        
MYImplant.Inst_CONTROL_NEXUS.Inst_Zarlink_SPI_Module.FSM_STATE_RNIR52J_0[3]     OR2        A        In      -         7.626       -         
MYImplant.Inst_CONTROL_NEXUS.Inst_Zarlink_SPI_Module.FSM_STATE_RNIR52J_0[3]     OR2        Y        Out     1.219     8.845       -         
N_48                                                                            Net        -        -       2.844     -           4         
MYImplant.Inst_CONTROL_NEXUS.Inst_Zarlink_SPI_Module.FSM_STATE_RNIQF461[3]      OR2A       B        In      -         11.690      -         
MYImplant.Inst_CONTROL_NEXUS.Inst_Zarlink_SPI_Module.FSM_STATE_RNIQF461[3]      OR2A       Y        Out     1.554     13.243      -         
N_52                                                                            Net        -        -       4.009     -           9         
MYImplant.Inst_CONTROL_NEXUS.Inst_Zarlink_SPI_Module.SPI_SDI_RNO_28             NOR2A      B        In      -         17.252      -         
MYImplant.Inst_CONTROL_NEXUS.Inst_Zarlink_SPI_Module.SPI_SDI_RNO_28             NOR2A      Y        Out     0.977     18.230      -         
N_109                                                                           Net        -        -       0.773     -           1         
MYImplant.Inst_CONTROL_NEXUS.Inst_Zarlink_SPI_Module.SPI_SDI_RNO_11             OR3        C        In      -         19.002      -         
MYImplant.Inst_CONTROL_NEXUS.Inst_Zarlink_SPI_Module.SPI_SDI_RNO_11             OR3        Y        Out     1.641     20.644      -         
SPI_SDI_17_iv_0_10                                                              Net        -        -       0.773     -           1         
MYImplant.Inst_CONTROL_NEXUS.Inst_Zarlink_SPI_Module.SPI_SDI_RNO_2              OR3        C        In      -         21.416      -         
MYImplant.Inst_CONTROL_NEXUS.Inst_Zarlink_SPI_Module.SPI_SDI_RNO_2              OR3        Y        Out     1.641     23.057      -         
SPI_SDI_17_iv_0_13                                                              Net        -        -       0.773     -           1         
MYImplant.Inst_CONTROL_NEXUS.Inst_Zarlink_SPI_Module.SPI_SDI_RNO                OR3        C        In      -         23.830      -         
MYImplant.Inst_CONTROL_NEXUS.Inst_Zarlink_SPI_Module.SPI_SDI_RNO                OR3        Y        Out     1.641     25.471      -         
SPI_SDI_17                                                                      Net        -        -       0.773     -           1         
MYImplant.Inst_CONTROL_NEXUS.Inst_Zarlink_SPI_Module.SPI_SDI                    DFN1C0     D        In      -         26.244      -         
============================================================================================================================================
Total path delay (propagation time + setup) of 27.538 is 11.739(42.6%) logic and 15.799(57.4%) route.
Path delay compensated for clock skew. Clock skew is added to clock-to-out value, and is subtracted from setup time value


Path information for path number 4: 
      Requested Period:                      10.000
    - Setup time:                            1.378
    + Clock delay at ending point:           0.000 (ideal)
    = Required time:                         8.622

    - Propagation time:                      25.596
    - Clock delay at starting point:         0.000 (ideal)
    = Slack (non-critical) :                 -16.974

    Number of logic level(s):                6
    Starting point:                          MYImplant.Inst_CONTROL_NEXUS.Inst_Zarlink_SPI_Module.FSM_STATE[0] / Q
    Ending point:                            MYImplant.Inst_CONTROL_NEXUS.Inst_Zarlink_SPI_Module.SPI_SDI / D
    The start point is clocked by            COMMAND_RECEIVER|Clk_4MHz_inferred_clock [rising] on pin CLK
    The end   point is clocked by            COMMAND_RECEIVER|Clk_4MHz_inferred_clock [rising] on pin CLK

Instance / Net                                                                           Pin      Pin               Arrival     No. of    
Name                                                                          Type       Name     Dir     Delay     Time        Fan Out(s)
------------------------------------------------------------------------------------------------------------------------------------------
MYImplant.Inst_CONTROL_NEXUS.Inst_Zarlink_SPI_Module.FSM_STATE[0]             DFN1C0     Q        Out     1.771     1.771       -         
FSM_STATE[0]                                                                  Net        -        -       5.855     -           23        
MYImplant.Inst_CONTROL_NEXUS.Inst_Zarlink_SPI_Module.FSM_STATE_RNIN12J[1]     XOR2       B        In      -         7.626       -         
MYImplant.Inst_CONTROL_NEXUS.Inst_Zarlink_SPI_Module.FSM_STATE_RNIN12J[1]     XOR2       Y        Out     2.251     9.877       -         
N_43_i                                                                        Net        -        -       4.009     -           9         
MYImplant.Inst_CONTROL_NEXUS.Inst_Zarlink_SPI_Module.SPI_SDI_RNO_35           NOR2A      B        In      -         13.886      -         
MYImplant.Inst_CONTROL_NEXUS.Inst_Zarlink_SPI_Module.SPI_SDI_RNO_35           NOR2A      Y        Out     0.927     14.813      -         
SPI_SDI_17_iv_0_a19_12_0                                                      Net        -        -       0.773     -           1         
MYImplant.Inst_CONTROL_NEXUS.Inst_Zarlink_SPI_Module.SPI_SDI_RNO_28           NOR2A      A        In      -         15.586      -         
MYImplant.Inst_CONTROL_NEXUS.Inst_Zarlink_SPI_Module.SPI_SDI_RNO_28           NOR2A      Y        Out     1.508     17.094      -         
N_109                                                                         Net        -        -       0.773     -           1         
MYImplant.Inst_CONTROL_NEXUS.Inst_Zarlink_SPI_Module.SPI_SDI_RNO_11           OR3        C        In      -         17.866      -         
MYImplant.Inst_CONTROL_NEXUS.Inst_Zarlink_SPI_Module.SPI_SDI_RNO_11           OR3        Y        Out     1.804     19.670      -         
SPI_SDI_17_iv_0_10                                                            Net        -        -       0.773     -           1         
MYImplant.Inst_CONTROL_NEXUS.Inst_Zarlink_SPI_Module.SPI_SDI_RNO_2            OR3        C        In      -         20.443      -         
MYImplant.Inst_CONTROL_NEXUS.Inst_Zarlink_SPI_Module.SPI_SDI_RNO_2            OR3        Y        Out     1.804     22.247      -         
SPI_SDI_17_iv_0_13                                                            Net        -        -       0.773     -           1         
MYImplant.Inst_CONTROL_NEXUS.Inst_Zarlink_SPI_Module.SPI_SDI_RNO              OR3        C        In      -         23.020      -         
MYImplant.Inst_CONTROL_NEXUS.Inst_Zarlink_SPI_Module.SPI_SDI_RNO              OR3        Y        Out     1.804     24.824      -         
SPI_SDI_17                                                                    Net        -        -       0.773     -           1         
MYImplant.Inst_CONTROL_NEXUS.Inst_Zarlink_SPI_Module.SPI_SDI                  DFN1C0     D        In      -         25.596      -         
==========================================================================================================================================
Total path delay (propagation time + setup) of 26.974 is 13.247(49.1%) logic and 13.728(50.9%) route.
Path delay compensated for clock skew. Clock skew is added to clock-to-out value, and is subtracted from setup time value


Path information for path number 5: 
      Requested Period:                      10.000
    - Setup time:                            1.295
    + Clock delay at ending point:           0.000 (ideal)
    = Required time:                         8.705

    - Propagation time:                      25.618
    - Clock delay at starting point:         0.000 (ideal)
    = Slack (non-critical) :                 -16.912

    Number of logic level(s):                6
    Starting point:                          MYImplant.Inst_CONTROL_NEXUS.Inst_Zarlink_SPI_Module.FSM_STATE[3] / Q
    Ending point:                            MYImplant.Inst_CONTROL_NEXUS.Inst_Zarlink_SPI_Module.SPI_SDI / D
    The start point is clocked by            COMMAND_RECEIVER|Clk_4MHz_inferred_clock [rising] on pin CLK
    The end   point is clocked by            COMMAND_RECEIVER|Clk_4MHz_inferred_clock [rising] on pin CLK

Instance / Net                                                                             Pin      Pin               Arrival     No. of    
Name                                                                            Type       Name     Dir     Delay     Time        Fan Out(s)
--------------------------------------------------------------------------------------------------------------------------------------------
MYImplant.Inst_CONTROL_NEXUS.Inst_Zarlink_SPI_Module.FSM_STATE[3]               DFN1C0     Q        Out     1.771     1.771       -         
FSM_STATE[3]                                                                    Net        -        -       4.895     -           13        
MYImplant.Inst_CONTROL_NEXUS.Inst_Zarlink_SPI_Module.FSM_STATE_RNIR52J_0[3]     OR2        B        In      -         6.665       -         
MYImplant.Inst_CONTROL_NEXUS.Inst_Zarlink_SPI_Module.FSM_STATE_RNIR52J_0[3]     OR2        Y        Out     1.554     8.219       -         
N_48                                                                            Net        -        -       2.844     -           4         
MYImplant.Inst_CONTROL_NEXUS.Inst_Zarlink_SPI_Module.FSM_STATE_RNIQF461[3]      OR2A       B        In      -         11.063      -         
MYImplant.Inst_CONTROL_NEXUS.Inst_Zarlink_SPI_Module.FSM_STATE_RNIQF461[3]      OR2A       Y        Out     1.554     12.617      -         
N_52                                                                            Net        -        -       4.009     -           9         
MYImplant.Inst_CONTROL_NEXUS.Inst_Zarlink_SPI_Module.SPI_SDI_RNO_28             NOR2A      B        In      -         16.626      -         
MYImplant.Inst_CONTROL_NEXUS.Inst_Zarlink_SPI_Module.SPI_SDI_RNO_28             NOR2A      Y        Out     0.977     17.603      -         
N_109                                                                           Net        -        -       0.773     -           1         
MYImplant.Inst_CONTROL_NEXUS.Inst_Zarlink_SPI_Module.SPI_SDI_RNO_11             OR3        C        In      -         18.376      -         
MYImplant.Inst_CONTROL_NEXUS.Inst_Zarlink_SPI_Module.SPI_SDI_RNO_11             OR3        Y        Out     1.641     20.017      -         
SPI_SDI_17_iv_0_10                                                              Net        -        -       0.773     -           1         
MYImplant.Inst_CONTROL_NEXUS.Inst_Zarlink_SPI_Module.SPI_SDI_RNO_2              OR3        C        In      -         20.790      -         
MYImplant.Inst_CONTROL_NEXUS.Inst_Zarlink_SPI_Module.SPI_SDI_RNO_2              OR3        Y        Out     1.641     22.431      -         
SPI_SDI_17_iv_0_13                                                              Net        -        -       0.773     -           1         
MYImplant.Inst_CONTROL_NEXUS.Inst_Zarlink_SPI_Module.SPI_SDI_RNO                OR3        C        In      -         23.204      -         
MYImplant.Inst_CONTROL_NEXUS.Inst_Zarlink_SPI_Module.SPI_SDI_RNO                OR3        Y        Out     1.641     24.845      -         
SPI_SDI_17                                                                      Net        -        -       0.773     -           1         
MYImplant.Inst_CONTROL_NEXUS.Inst_Zarlink_SPI_Module.SPI_SDI                    DFN1C0     D        In      -         25.618      -         
============================================================================================================================================
Total path delay (propagation time + setup) of 26.912 is 12.074(44.9%) logic and 14.839(55.1%) route.
Path delay compensated for clock skew. Clock skew is added to clock-to-out value, and is subtracted from setup time value


====================================
Detailed Report for Clock: IGLOO_TOP|Clk
====================================


Starting Points with Worst Slack
********************************

                                                                       Starting                                                 Arrival           
Instance                                                               Reference         Type       Pin     Net                 Time        Slack 
                                                                       Clock                                                                      
--------------------------------------------------------------------------------------------------------------------------------------------------
MYImplant.Inst_CONTROL_NEXUS.Inst_COMMAND_RECEIVER.Clk_4MHz            IGLOO_TOP|Clk     DFN1C0     Q       Clk_4MHz_i          1.771       29.438
MYImplant.Inst_CONTROL_NEXUS.Inst_COMMAND_RECEIVER.Counter_4MHz[1]     IGLOO_TOP|Clk     DFN1C0     Q       Counter_4MHz[1]     1.771       34.650
MYImplant.Clk                                                          IGLOO_TOP|Clk     DFN1C0     Q       Clk_i               1.771       35.681
MYImplant.Inst_CONTROL_NEXUS.Inst_COMMAND_RECEIVER.Counter_4MHz[0]     IGLOO_TOP|Clk     DFN1C0     Q       Counter_4MHz[0]     1.771       35.727
==================================================================================================================================================


Ending Points with Worst Slack
******************************

                                                                       Starting                                                 Required           
Instance                                                               Reference         Type       Pin     Net                 Time         Slack 
                                                                       Clock                                                                       
---------------------------------------------------------------------------------------------------------------------------------------------------
MYImplant.Inst_CONTROL_NEXUS.Inst_COMMAND_RECEIVER.Clk_4MHz            IGLOO_TOP|Clk     DFN1C0     D       Clk_4MHz_RNO        40.371       29.438
MYImplant.Inst_CONTROL_NEXUS.Inst_COMMAND_RECEIVER.Counter_4MHz[0]     IGLOO_TOP|Clk     DFN1C0     D       SUM1                40.371       34.650
MYImplant.Clk                                                          IGLOO_TOP|Clk     DFN1C0     D       Clk_i_i             40.371       35.681
MYImplant.Inst_CONTROL_NEXUS.Inst_COMMAND_RECEIVER.Counter_4MHz[1]     IGLOO_TOP|Clk     DFN1C0     D       Counter_4MHz[0]     40.288       37.590
===================================================================================================================================================


Worst Path Information
View Worst Path in Analyst
***********************


Path information for path number 1: 
      Requested Period:                      41.666
    - Setup time:                            1.295
    + Clock delay at ending point:           0.000 (ideal)
    = Required time:                         40.371

    - Propagation time:                      10.934
    - Clock delay at starting point:         0.000 (ideal)
    = Slack (non-critical) :                 29.438

    Number of logic level(s):                1
    Starting point:                          MYImplant.Inst_CONTROL_NEXUS.Inst_COMMAND_RECEIVER.Clk_4MHz / Q
    Ending point:                            MYImplant.Inst_CONTROL_NEXUS.Inst_COMMAND_RECEIVER.Clk_4MHz / D
    The start point is clocked by            IGLOO_TOP|Clk [rising] on pin CLK
    The end   point is clocked by            IGLOO_TOP|Clk [rising] on pin CLK

Instance / Net                                                                 Pin      Pin               Arrival     No. of    
Name                                                                Type       Name     Dir     Delay     Time        Fan Out(s)
--------------------------------------------------------------------------------------------------------------------------------
MYImplant.Inst_CONTROL_NEXUS.Inst_COMMAND_RECEIVER.Clk_4MHz         DFN1C0     Q        Out     1.771     1.771       -         
Clk_4MHz_i                                                          Net        -        -       7.217     -           43        
MYImplant.Inst_CONTROL_NEXUS.Inst_COMMAND_RECEIVER.Clk_4MHz_RNO     XOR2       A        In      -         8.987       -         
MYImplant.Inst_CONTROL_NEXUS.Inst_COMMAND_RECEIVER.Clk_4MHz_RNO     XOR2       Y        Out     1.174     10.161      -         
Clk_4MHz_RNO                                                        Net        -        -       0.773     -           1         
MYImplant.Inst_CONTROL_NEXUS.Inst_COMMAND_RECEIVER.Clk_4MHz         DFN1C0     D        In      -         10.934      -         
================================================================================================================================
Total path delay (propagation time + setup) of 12.228 is 4.239(34.7%) logic and 7.989(65.3%) route.
Path delay compensated for clock skew. Clock skew is added to clock-to-out value, and is subtracted from setup time value


====================================
Detailed Report for Clock: IMPLANT_TOP|Clk_inferred_clock
====================================


Starting Points with Worst Slack
********************************

                                                                                               Starting                                                                                      Arrival            
Instance                                                                                       Reference                          Type       Pin     Net                                     Time        Slack  
                                                                                               Clock                                                                                                            
----------------------------------------------------------------------------------------------------------------------------------------------------------------------------------------------------------------
MYImplant.Inst_DATA_ACQUISITION_BLOCK.Inst_RHA_TO_ZL_CONVERTER.SPIMACHINE_STATE[2]             IMPLANT_TOP|Clk_inferred_clock     DFN1C1     Q       SPIMACHINE_STATE[2]                     1.771       -28.531
MYImplant.Inst_DATA_ACQUISITION_BLOCK.Inst_RHA_TO_ZL_CONVERTER.SPIMACHINE_STATE[4]             IMPLANT_TOP|Clk_inferred_clock     DFN1C1     Q       SPIMACHINE_STATE[4]                     1.771       -27.992
MYImplant.Inst_CONTROL_NEXUS.Inst_ZARLINK_CONNECT_INIT.Zarlink_ConnectionTimeoutCounter[1]     IMPLANT_TOP|Clk_inferred_clock     DFN1C0     Q       Zarlink_ConnectionTimeoutCounter[1]     1.395       -27.813
MYImplant.Inst_CONTROL_NEXUS.Inst_ZARLINK_CONNECT_INIT.Zarlink_ConnectionTimeoutCounter[0]     IMPLANT_TOP|Clk_inferred_clock     DFN1C0     Q       Zarlink_ConnectionTimeoutCounter[0]     1.395       -27.804
MYImplant.Inst_DATA_ACQUISITION_BLOCK.Inst_RHA_TO_ZL_CONVERTER.COUNTER_BITS_PROCESSED[4]       IMPLANT_TOP|Clk_inferred_clock     DFN1C1     Q       COUNTER_BITS_PROCESSED[4]               1.771       -27.566
MYImplant.Inst_DATA_ACQUISITION_BLOCK.Inst_RHA_TO_ZL_CONVERTER.SPIMACHINE_STATE[0]             IMPLANT_TOP|Clk_inferred_clock     DFN1C1     Q       SPIMACHINE_STATE[0]                     1.771       -27.557
MYImplant.Inst_DATA_ACQUISITION_BLOCK.Inst_RHA_TO_ZL_CONVERTER.COUNTER_BITS_PROCESSED[5]       IMPLANT_TOP|Clk_inferred_clock     DFN1C1     Q       COUNTER_BITS_PROCESSED[5]               1.771       -27.449
MYImplant.Inst_DATA_ACQUISITION_BLOCK.Inst_RHA_TO_ZL_CONVERTER.SPIMACHINE_STATE[1]             IMPLANT_TOP|Clk_inferred_clock     DFN1C1     Q       SPIMACHINE_STATE[1]                     1.395       -27.140
MYImplant.Inst_DATA_ACQUISITION_BLOCK.Inst_RHA_TO_ZL_CONVERTER.COUNTER_BITS_OF_A_BYTE[1]       IMPLANT_TOP|Clk_inferred_clock     DFN1C1     Q       COUNTER_BITS_OF_A_BYTE[1]               1.771       -27.127
MYImplant.Inst_CONTROL_NEXUS.Inst_ZARLINK_CONNECT_INIT.Zarlink_ConnectionTimeoutCounter[9]     IMPLANT_TOP|Clk_inferred_clock     DFN1C0     Q       Zarlink_ConnectionTimeoutCounter[9]     1.395       -27.065
================================================================================================================================================================================================================


Ending Points with Worst Slack
******************************

                                                                                                Starting                                                                                         Required            
Instance                                                                                        Reference                          Type         Pin     Net                                      Time         Slack  
                                                                                                Clock                                                                                                                
---------------------------------------------------------------------------------------------------------------------------------------------------------------------------------------------------------------------
MYImplant.Inst_DATA_ACQUISITION_BLOCK.Inst_RHA_TO_ZL_CONVERTER.FIFO_OUT_DATA_INTERNAL[0]        IMPLANT_TOP|Clk_inferred_clock     DFN1E0C1     E       un1_SPIMACHINE_STATE_7                   8.956        -28.531
MYImplant.Inst_DATA_ACQUISITION_BLOCK.Inst_RHA_TO_ZL_CONVERTER.FIFO_OUT_DATA_INTERNAL[1]        IMPLANT_TOP|Clk_inferred_clock     DFN1E0C1     E       un1_SPIMACHINE_STATE_7                   8.956        -28.531
MYImplant.Inst_DATA_ACQUISITION_BLOCK.Inst_RHA_TO_ZL_CONVERTER.FIFO_OUT_DATA_INTERNAL[2]        IMPLANT_TOP|Clk_inferred_clock     DFN1E0C1     E       un1_SPIMACHINE_STATE_7                   8.956        -28.531
MYImplant.Inst_DATA_ACQUISITION_BLOCK.Inst_RHA_TO_ZL_CONVERTER.FIFO_OUT_DATA_INTERNAL[3]        IMPLANT_TOP|Clk_inferred_clock     DFN1E0C1     E       un1_SPIMACHINE_STATE_7                   8.956        -28.531
MYImplant.Inst_DATA_ACQUISITION_BLOCK.Inst_RHA_TO_ZL_CONVERTER.FIFO_OUT_DATA_INTERNAL[4]        IMPLANT_TOP|Clk_inferred_clock     DFN1E0C1     E       un1_SPIMACHINE_STATE_7                   8.956        -28.531
MYImplant.Inst_DATA_ACQUISITION_BLOCK.Inst_RHA_TO_ZL_CONVERTER.FIFO_OUT_DATA_INTERNAL[5]        IMPLANT_TOP|Clk_inferred_clock     DFN1E0C1     E       un1_SPIMACHINE_STATE_7                   8.956        -28.531
MYImplant.Inst_DATA_ACQUISITION_BLOCK.Inst_RHA_TO_ZL_CONVERTER.FIFO_OUT_DATA_INTERNAL[6]        IMPLANT_TOP|Clk_inferred_clock     DFN1E0C1     E       un1_SPIMACHINE_STATE_7                   8.956        -28.531
MYImplant.Inst_DATA_ACQUISITION_BLOCK.Inst_RHA_TO_ZL_CONVERTER.FIFO_OUT_DATA_INTERNAL[7]        IMPLANT_TOP|Clk_inferred_clock     DFN1E0C1     E       un1_SPIMACHINE_STATE_7                   8.956        -28.531
MYImplant.Inst_DATA_ACQUISITION_BLOCK.Inst_RHA_TO_ZL_CONVERTER.COUNTER_BITS_PROCESSED[6]        IMPLANT_TOP|Clk_inferred_clock     DFN1C1       D       COUNTER_BITS_PROCESSED_10[6]             8.622        -28.071
MYImplant.Inst_CONTROL_NEXUS.Inst_ZARLINK_CONNECT_INIT.Zarlink_ConnectionTimeoutCounter[21]     IMPLANT_TOP|Clk_inferred_clock     DFN1C0       D       Zarlink_ConnectionTimeoutCounter_n21     8.705        -27.813
=====================================================================================================================================================================================================================


Worst Path Information
View Worst Path in Analyst
***********************


Path information for path number 1: 
      Requested Period:                      10.000
    - Setup time:                            1.044
    + Clock delay at ending point:           0.000 (ideal)
    = Required time:                         8.956

    - Propagation time:                      37.487
    - Clock delay at starting point:         0.000 (ideal)
    = Slack (critical) :                     -28.531

    Number of logic level(s):                5
    Starting point:                          MYImplant.Inst_DATA_ACQUISITION_BLOCK.Inst_RHA_TO_ZL_CONVERTER.SPIMACHINE_STATE[2] / Q
    Ending point:                            MYImplant.Inst_DATA_ACQUISITION_BLOCK.Inst_RHA_TO_ZL_CONVERTER.FIFO_OUT_DATA_INTERNAL[0] / E
    The start point is clocked by            IMPLANT_TOP|Clk_inferred_clock [rising] on pin CLK
    The end   point is clocked by            IMPLANT_TOP|Clk_inferred_clock [rising] on pin CLK

Instance / Net                                                                                                Pin      Pin               Arrival     No. of    
Name                                                                                             Type         Name     Dir     Delay     Time        Fan Out(s)
---------------------------------------------------------------------------------------------------------------------------------------------------------------
MYImplant.Inst_DATA_ACQUISITION_BLOCK.Inst_RHA_TO_ZL_CONVERTER.SPIMACHINE_STATE[2]               DFN1C1       Q        Out     1.771     1.771       -         
SPIMACHINE_STATE[2]                                                                              Net          -        -       5.438     -           18        
MYImplant.Inst_DATA_ACQUISITION_BLOCK.Inst_RHA_TO_ZL_CONVERTER.SPIMACHINE_STATE_RNI05BH_1[2]     OR2A         B        In      -         7.208       -         
MYImplant.Inst_DATA_ACQUISITION_BLOCK.Inst_RHA_TO_ZL_CONVERTER.SPIMACHINE_STATE_RNI05BH_1[2]     OR2A         Y        Out     1.554     8.762       -         
N_628                                                                                            Net          -        -       5.546     -           19        
MYImplant.Inst_DATA_ACQUISITION_BLOCK.Inst_RHA_TO_ZL_CONVERTER.SPIMACHINE_STATE_RNIGN0Q[3]       OR2A         B        In      -         14.308      -         
MYImplant.Inst_DATA_ACQUISITION_BLOCK.Inst_RHA_TO_ZL_CONVERTER.SPIMACHINE_STATE_RNIGN0Q[3]       OR2A         Y        Out     1.554     15.862      -         
N_632                                                                                            Net          -        -       5.112     -           15        
MYImplant.Inst_DATA_ACQUISITION_BLOCK.Inst_RHA_TO_ZL_CONVERTER.SPIMACHINE_STATE_RNIU7M21[1]      OR2A         B        In      -         20.974      -         
MYImplant.Inst_DATA_ACQUISITION_BLOCK.Inst_RHA_TO_ZL_CONVERTER.SPIMACHINE_STATE_RNIU7M21[1]      OR2A         Y        Out     1.554     22.527      -         
N_653                                                                                            Net          -        -       3.074     -           5         
MYImplant.Inst_DATA_ACQUISITION_BLOCK.Inst_RHA_TO_ZL_CONVERTER.SPIMACHINE_STATE_RNIBNBB1[0]      NOR2A        B        In      -         25.601      -         
MYImplant.Inst_DATA_ACQUISITION_BLOCK.Inst_RHA_TO_ZL_CONVERTER.SPIMACHINE_STATE_RNIBNBB1[0]      NOR2A        Y        Out     0.977     26.578      -         
SPIMACHINE_STATE_d[13]                                                                           Net          -        -       5.329     -           17        
MYImplant.Inst_DATA_ACQUISITION_BLOCK.Inst_RHA_TO_ZL_CONVERTER.SPIMACHINE_STATE_RNI3L588[0]      OR3          C        In      -         31.907      -         
MYImplant.Inst_DATA_ACQUISITION_BLOCK.Inst_RHA_TO_ZL_CONVERTER.SPIMACHINE_STATE_RNI3L588[0]      OR3          Y        Out     1.641     33.548      -         
un1_SPIMACHINE_STATE_7                                                                           Net          -        -       3.938     -           8         
MYImplant.Inst_DATA_ACQUISITION_BLOCK.Inst_RHA_TO_ZL_CONVERTER.FIFO_OUT_DATA_INTERNAL[0]         DFN1E0C1     E        In      -         37.487      -         
===============================================================================================================================================================
Total path delay (propagation time + setup) of 38.531 is 10.094(26.2%) logic and 28.437(73.8%) route.
Path delay compensated for clock skew. Clock skew is added to clock-to-out value, and is subtracted from setup time value


Path information for path number 2: 
      Requested Period:                      10.000
    - Setup time:                            1.044
    + Clock delay at ending point:           0.000 (ideal)
    = Required time:                         8.956

    - Propagation time:                      37.487
    - Clock delay at starting point:         0.000 (ideal)
    = Slack (critical) :                     -28.531

    Number of logic level(s):                5
    Starting point:                          MYImplant.Inst_DATA_ACQUISITION_BLOCK.Inst_RHA_TO_ZL_CONVERTER.SPIMACHINE_STATE[2] / Q
    Ending point:                            MYImplant.Inst_DATA_ACQUISITION_BLOCK.Inst_RHA_TO_ZL_CONVERTER.FIFO_OUT_DATA_INTERNAL[7] / E
    The start point is clocked by            IMPLANT_TOP|Clk_inferred_clock [rising] on pin CLK
    The end   point is clocked by            IMPLANT_TOP|Clk_inferred_clock [rising] on pin CLK

Instance / Net                                                                                                Pin      Pin               Arrival     No. of    
Name                                                                                             Type         Name     Dir     Delay     Time        Fan Out(s)
---------------------------------------------------------------------------------------------------------------------------------------------------------------
MYImplant.Inst_DATA_ACQUISITION_BLOCK.Inst_RHA_TO_ZL_CONVERTER.SPIMACHINE_STATE[2]               DFN1C1       Q        Out     1.771     1.771       -         
SPIMACHINE_STATE[2]                                                                              Net          -        -       5.438     -           18        
MYImplant.Inst_DATA_ACQUISITION_BLOCK.Inst_RHA_TO_ZL_CONVERTER.SPIMACHINE_STATE_RNI05BH_1[2]     OR2A         B        In      -         7.208       -         
MYImplant.Inst_DATA_ACQUISITION_BLOCK.Inst_RHA_TO_ZL_CONVERTER.SPIMACHINE_STATE_RNI05BH_1[2]     OR2A         Y        Out     1.554     8.762       -         
N_628                                                                                            Net          -        -       5.546     -           19        
MYImplant.Inst_DATA_ACQUISITION_BLOCK.Inst_RHA_TO_ZL_CONVERTER.SPIMACHINE_STATE_RNIGN0Q[3]       OR2A         B        In      -         14.308      -         
MYImplant.Inst_DATA_ACQUISITION_BLOCK.Inst_RHA_TO_ZL_CONVERTER.SPIMACHINE_STATE_RNIGN0Q[3]       OR2A         Y        Out     1.554     15.862      -         
N_632                                                                                            Net          -        -       5.112     -           15        
MYImplant.Inst_DATA_ACQUISITION_BLOCK.Inst_RHA_TO_ZL_CONVERTER.SPIMACHINE_STATE_RNIU7M21[1]      OR2A         B        In      -         20.974      -         
MYImplant.Inst_DATA_ACQUISITION_BLOCK.Inst_RHA_TO_ZL_CONVERTER.SPIMACHINE_STATE_RNIU7M21[1]      OR2A         Y        Out     1.554     22.527      -         
N_653                                                                                            Net          -        -       3.074     -           5         
MYImplant.Inst_DATA_ACQUISITION_BLOCK.Inst_RHA_TO_ZL_CONVERTER.SPIMACHINE_STATE_RNIBNBB1[0]      NOR2A        B        In      -         25.601      -         
MYImplant.Inst_DATA_ACQUISITION_BLOCK.Inst_RHA_TO_ZL_CONVERTER.SPIMACHINE_STATE_RNIBNBB1[0]      NOR2A        Y        Out     0.977     26.578      -         
SPIMACHINE_STATE_d[13]                                                                           Net          -        -       5.329     -           17        
MYImplant.Inst_DATA_ACQUISITION_BLOCK.Inst_RHA_TO_ZL_CONVERTER.SPIMACHINE_STATE_RNI3L588[0]      OR3          C        In      -         31.907      -         
MYImplant.Inst_DATA_ACQUISITION_BLOCK.Inst_RHA_TO_ZL_CONVERTER.SPIMACHINE_STATE_RNI3L588[0]      OR3          Y        Out     1.641     33.548      -         
un1_SPIMACHINE_STATE_7                                                                           Net          -        -       3.938     -           8         
MYImplant.Inst_DATA_ACQUISITION_BLOCK.Inst_RHA_TO_ZL_CONVERTER.FIFO_OUT_DATA_INTERNAL[7]         DFN1E0C1     E        In      -         37.487      -         
===============================================================================================================================================================
Total path delay (propagation time + setup) of 38.531 is 10.094(26.2%) logic and 28.437(73.8%) route.
Path delay compensated for clock skew. Clock skew is added to clock-to-out value, and is subtracted from setup time value


Path information for path number 3: 
      Requested Period:                      10.000
    - Setup time:                            1.044
    + Clock delay at ending point:           0.000 (ideal)
    = Required time:                         8.956

    - Propagation time:                      37.487
    - Clock delay at starting point:         0.000 (ideal)
    = Slack (critical) :                     -28.531

    Number of logic level(s):                5
    Starting point:                          MYImplant.Inst_DATA_ACQUISITION_BLOCK.Inst_RHA_TO_ZL_CONVERTER.SPIMACHINE_STATE[2] / Q
    Ending point:                            MYImplant.Inst_DATA_ACQUISITION_BLOCK.Inst_RHA_TO_ZL_CONVERTER.FIFO_OUT_DATA_INTERNAL[6] / E
    The start point is clocked by            IMPLANT_TOP|Clk_inferred_clock [rising] on pin CLK
    The end   point is clocked by            IMPLANT_TOP|Clk_inferred_clock [rising] on pin CLK

Instance / Net                                                                                                Pin      Pin               Arrival     No. of    
Name                                                                                             Type         Name     Dir     Delay     Time        Fan Out(s)
---------------------------------------------------------------------------------------------------------------------------------------------------------------
MYImplant.Inst_DATA_ACQUISITION_BLOCK.Inst_RHA_TO_ZL_CONVERTER.SPIMACHINE_STATE[2]               DFN1C1       Q        Out     1.771     1.771       -         
SPIMACHINE_STATE[2]                                                                              Net          -        -       5.438     -           18        
MYImplant.Inst_DATA_ACQUISITION_BLOCK.Inst_RHA_TO_ZL_CONVERTER.SPIMACHINE_STATE_RNI05BH_1[2]     OR2A         B        In      -         7.208       -         
MYImplant.Inst_DATA_ACQUISITION_BLOCK.Inst_RHA_TO_ZL_CONVERTER.SPIMACHINE_STATE_RNI05BH_1[2]     OR2A         Y        Out     1.554     8.762       -         
N_628                                                                                            Net          -        -       5.546     -           19        
MYImplant.Inst_DATA_ACQUISITION_BLOCK.Inst_RHA_TO_ZL_CONVERTER.SPIMACHINE_STATE_RNIGN0Q[3]       OR2A         B        In      -         14.308      -         
MYImplant.Inst_DATA_ACQUISITION_BLOCK.Inst_RHA_TO_ZL_CONVERTER.SPIMACHINE_STATE_RNIGN0Q[3]       OR2A         Y        Out     1.554     15.862      -         
N_632                                                                                            Net          -        -       5.112     -           15        
MYImplant.Inst_DATA_ACQUISITION_BLOCK.Inst_RHA_TO_ZL_CONVERTER.SPIMACHINE_STATE_RNIU7M21[1]      OR2A         B        In      -         20.974      -         
MYImplant.Inst_DATA_ACQUISITION_BLOCK.Inst_RHA_TO_ZL_CONVERTER.SPIMACHINE_STATE_RNIU7M21[1]      OR2A         Y        Out     1.554     22.527      -         
N_653                                                                                            Net          -        -       3.074     -           5         
MYImplant.Inst_DATA_ACQUISITION_BLOCK.Inst_RHA_TO_ZL_CONVERTER.SPIMACHINE_STATE_RNIBNBB1[0]      NOR2A        B        In      -         25.601      -         
MYImplant.Inst_DATA_ACQUISITION_BLOCK.Inst_RHA_TO_ZL_CONVERTER.SPIMACHINE_STATE_RNIBNBB1[0]      NOR2A        Y        Out     0.977     26.578      -         
SPIMACHINE_STATE_d[13]                                                                           Net          -        -       5.329     -           17        
MYImplant.Inst_DATA_ACQUISITION_BLOCK.Inst_RHA_TO_ZL_CONVERTER.SPIMACHINE_STATE_RNI3L588[0]      OR3          C        In      -         31.907      -         
MYImplant.Inst_DATA_ACQUISITION_BLOCK.Inst_RHA_TO_ZL_CONVERTER.SPIMACHINE_STATE_RNI3L588[0]      OR3          Y        Out     1.641     33.548      -         
un1_SPIMACHINE_STATE_7                                                                           Net          -        -       3.938     -           8         
MYImplant.Inst_DATA_ACQUISITION_BLOCK.Inst_RHA_TO_ZL_CONVERTER.FIFO_OUT_DATA_INTERNAL[6]         DFN1E0C1     E        In      -         37.487      -         
===============================================================================================================================================================
Total path delay (propagation time + setup) of 38.531 is 10.094(26.2%) logic and 28.437(73.8%) route.
Path delay compensated for clock skew. Clock skew is added to clock-to-out value, and is subtracted from setup time value


Path information for path number 4: 
      Requested Period:                      10.000
    - Setup time:                            1.044
    + Clock delay at ending point:           0.000 (ideal)
    = Required time:                         8.956

    - Propagation time:                      37.487
    - Clock delay at starting point:         0.000 (ideal)
    = Slack (critical) :                     -28.531

    Number of logic level(s):                5
    Starting point:                          MYImplant.Inst_DATA_ACQUISITION_BLOCK.Inst_RHA_TO_ZL_CONVERTER.SPIMACHINE_STATE[2] / Q
    Ending point:                            MYImplant.Inst_DATA_ACQUISITION_BLOCK.Inst_RHA_TO_ZL_CONVERTER.FIFO_OUT_DATA_INTERNAL[5] / E
    The start point is clocked by            IMPLANT_TOP|Clk_inferred_clock [rising] on pin CLK
    The end   point is clocked by            IMPLANT_TOP|Clk_inferred_clock [rising] on pin CLK

Instance / Net                                                                                                Pin      Pin               Arrival     No. of    
Name                                                                                             Type         Name     Dir     Delay     Time        Fan Out(s)
---------------------------------------------------------------------------------------------------------------------------------------------------------------
MYImplant.Inst_DATA_ACQUISITION_BLOCK.Inst_RHA_TO_ZL_CONVERTER.SPIMACHINE_STATE[2]               DFN1C1       Q        Out     1.771     1.771       -         
SPIMACHINE_STATE[2]                                                                              Net          -        -       5.438     -           18        
MYImplant.Inst_DATA_ACQUISITION_BLOCK.Inst_RHA_TO_ZL_CONVERTER.SPIMACHINE_STATE_RNI05BH_1[2]     OR2A         B        In      -         7.208       -         
MYImplant.Inst_DATA_ACQUISITION_BLOCK.Inst_RHA_TO_ZL_CONVERTER.SPIMACHINE_STATE_RNI05BH_1[2]     OR2A         Y        Out     1.554     8.762       -         
N_628                                                                                            Net          -        -       5.546     -           19        
MYImplant.Inst_DATA_ACQUISITION_BLOCK.Inst_RHA_TO_ZL_CONVERTER.SPIMACHINE_STATE_RNIGN0Q[3]       OR2A         B        In      -         14.308      -         
MYImplant.Inst_DATA_ACQUISITION_BLOCK.Inst_RHA_TO_ZL_CONVERTER.SPIMACHINE_STATE_RNIGN0Q[3]       OR2A         Y        Out     1.554     15.862      -         
N_632                                                                                            Net          -        -       5.112     -           15        
MYImplant.Inst_DATA_ACQUISITION_BLOCK.Inst_RHA_TO_ZL_CONVERTER.SPIMACHINE_STATE_RNIU7M21[1]      OR2A         B        In      -         20.974      -         
MYImplant.Inst_DATA_ACQUISITION_BLOCK.Inst_RHA_TO_ZL_CONVERTER.SPIMACHINE_STATE_RNIU7M21[1]      OR2A         Y        Out     1.554     22.527      -         
N_653                                                                                            Net          -        -       3.074     -           5         
MYImplant.Inst_DATA_ACQUISITION_BLOCK.Inst_RHA_TO_ZL_CONVERTER.SPIMACHINE_STATE_RNIBNBB1[0]      NOR2A        B        In      -         25.601      -         
MYImplant.Inst_DATA_ACQUISITION_BLOCK.Inst_RHA_TO_ZL_CONVERTER.SPIMACHINE_STATE_RNIBNBB1[0]      NOR2A        Y        Out     0.977     26.578      -         
SPIMACHINE_STATE_d[13]                                                                           Net          -        -       5.329     -           17        
MYImplant.Inst_DATA_ACQUISITION_BLOCK.Inst_RHA_TO_ZL_CONVERTER.SPIMACHINE_STATE_RNI3L588[0]      OR3          C        In      -         31.907      -         
MYImplant.Inst_DATA_ACQUISITION_BLOCK.Inst_RHA_TO_ZL_CONVERTER.SPIMACHINE_STATE_RNI3L588[0]      OR3          Y        Out     1.641     33.548      -         
un1_SPIMACHINE_STATE_7                                                                           Net          -        -       3.938     -           8         
MYImplant.Inst_DATA_ACQUISITION_BLOCK.Inst_RHA_TO_ZL_CONVERTER.FIFO_OUT_DATA_INTERNAL[5]         DFN1E0C1     E        In      -         37.487      -         
===============================================================================================================================================================
Total path delay (propagation time + setup) of 38.531 is 10.094(26.2%) logic and 28.437(73.8%) route.
Path delay compensated for clock skew. Clock skew is added to clock-to-out value, and is subtracted from setup time value


Path information for path number 5: 
      Requested Period:                      10.000
    - Setup time:                            1.044
    + Clock delay at ending point:           0.000 (ideal)
    = Required time:                         8.956

    - Propagation time:                      37.487
    - Clock delay at starting point:         0.000 (ideal)
    = Slack (critical) :                     -28.531

    Number of logic level(s):                5
    Starting point:                          MYImplant.Inst_DATA_ACQUISITION_BLOCK.Inst_RHA_TO_ZL_CONVERTER.SPIMACHINE_STATE[2] / Q
    Ending point:                            MYImplant.Inst_DATA_ACQUISITION_BLOCK.Inst_RHA_TO_ZL_CONVERTER.FIFO_OUT_DATA_INTERNAL[4] / E
    The start point is clocked by            IMPLANT_TOP|Clk_inferred_clock [rising] on pin CLK
    The end   point is clocked by            IMPLANT_TOP|Clk_inferred_clock [rising] on pin CLK

Instance / Net                                                                                                Pin      Pin               Arrival     No. of    
Name                                                                                             Type         Name     Dir     Delay     Time        Fan Out(s)
---------------------------------------------------------------------------------------------------------------------------------------------------------------
MYImplant.Inst_DATA_ACQUISITION_BLOCK.Inst_RHA_TO_ZL_CONVERTER.SPIMACHINE_STATE[2]               DFN1C1       Q        Out     1.771     1.771       -         
SPIMACHINE_STATE[2]                                                                              Net          -        -       5.438     -           18        
MYImplant.Inst_DATA_ACQUISITION_BLOCK.Inst_RHA_TO_ZL_CONVERTER.SPIMACHINE_STATE_RNI05BH_1[2]     OR2A         B        In      -         7.208       -         
MYImplant.Inst_DATA_ACQUISITION_BLOCK.Inst_RHA_TO_ZL_CONVERTER.SPIMACHINE_STATE_RNI05BH_1[2]     OR2A         Y        Out     1.554     8.762       -         
N_628                                                                                            Net          -        -       5.546     -           19        
MYImplant.Inst_DATA_ACQUISITION_BLOCK.Inst_RHA_TO_ZL_CONVERTER.SPIMACHINE_STATE_RNIGN0Q[3]       OR2A         B        In      -         14.308      -         
MYImplant.Inst_DATA_ACQUISITION_BLOCK.Inst_RHA_TO_ZL_CONVERTER.SPIMACHINE_STATE_RNIGN0Q[3]       OR2A         Y        Out     1.554     15.862      -         
N_632                                                                                            Net          -        -       5.112     -           15        
MYImplant.Inst_DATA_ACQUISITION_BLOCK.Inst_RHA_TO_ZL_CONVERTER.SPIMACHINE_STATE_RNIU7M21[1]      OR2A         B        In      -         20.974      -         
MYImplant.Inst_DATA_ACQUISITION_BLOCK.Inst_RHA_TO_ZL_CONVERTER.SPIMACHINE_STATE_RNIU7M21[1]      OR2A         Y        Out     1.554     22.527      -         
N_653                                                                                            Net          -        -       3.074     -           5         
MYImplant.Inst_DATA_ACQUISITION_BLOCK.Inst_RHA_TO_ZL_CONVERTER.SPIMACHINE_STATE_RNIBNBB1[0]      NOR2A        B        In      -         25.601      -         
MYImplant.Inst_DATA_ACQUISITION_BLOCK.Inst_RHA_TO_ZL_CONVERTER.SPIMACHINE_STATE_RNIBNBB1[0]      NOR2A        Y        Out     0.977     26.578      -         
SPIMACHINE_STATE_d[13]                                                                           Net          -        -       5.329     -           17        
MYImplant.Inst_DATA_ACQUISITION_BLOCK.Inst_RHA_TO_ZL_CONVERTER.SPIMACHINE_STATE_RNI3L588[0]      OR3          C        In      -         31.907      -         
MYImplant.Inst_DATA_ACQUISITION_BLOCK.Inst_RHA_TO_ZL_CONVERTER.SPIMACHINE_STATE_RNI3L588[0]      OR3          Y        Out     1.641     33.548      -         
un1_SPIMACHINE_STATE_7                                                                           Net          -        -       3.938     -           8         
MYImplant.Inst_DATA_ACQUISITION_BLOCK.Inst_RHA_TO_ZL_CONVERTER.FIFO_OUT_DATA_INTERNAL[4]         DFN1E0C1     E        In      -         37.487      -         
===============================================================================================================================================================
Total path delay (propagation time + setup) of 38.531 is 10.094(26.2%) logic and 28.437(73.8%) route.
Path delay compensated for clock skew. Clock skew is added to clock-to-out value, and is subtracted from setup time value


##### END OF TIMING REPORT #####]

--------------------------------------------------------------------------------
Target Part: AGLN250V2_VQFP100_STD
Report for cell IGLOO_TOP.igloo_top_arch
  Core Cell usage:
              cell count     area count*area
              AND2     8      1.0        8.0
              AND3    22      1.0       22.0
               AO1   249      1.0      249.0
              AO1A   109      1.0      109.0
              AO1B    21      1.0       21.0
              AO1C    16      1.0       16.0
              AO1D     8      1.0        8.0
              AOI1    16      1.0       16.0
             AOI1B    14      1.0       14.0
               AX1     3      1.0        3.0
              AX1A     2      1.0        2.0
              AX1B     3      1.0        3.0
              AX1C    46      1.0       46.0
              AX1D     1      1.0        1.0
              AX1E     3      1.0        3.0
              AXO6     1      1.0        1.0
             AXOI2     1      1.0        1.0
            CLKINT     5      0.0        0.0
               GND    28      0.0        0.0
               INV    25      1.0       25.0
               MX2   454      1.0      454.0
              MX2A    11      1.0       11.0
              MX2B     2      1.0        2.0
              MX2C    11      1.0       11.0
             NAND2     2      1.0        2.0
            NAND2A     1      1.0        1.0
              NOR2   190      1.0      190.0
             NOR2A   574      1.0      574.0
             NOR2B   589      1.0      589.0
              NOR3    43      1.0       43.0
             NOR3A   138      1.0      138.0
             NOR3B   222      1.0      222.0
             NOR3C   275      1.0      275.0
               OA1    55      1.0       55.0
              OA1A    18      1.0       18.0
              OA1B    18      1.0       18.0
              OA1C    19      1.0       19.0
              OAI1     2      1.0        2.0
               OR2   166      1.0      166.0
              OR2A   108      1.0      108.0
              OR2B    37      1.0       37.0
               OR3   304      1.0      304.0
              OR3A    20      1.0       20.0
              OR3B    12      1.0       12.0
              OR3C     5      1.0        5.0
               VCC    28      0.0        0.0
               XA1     8      1.0        8.0
              XA1A    30      1.0       30.0
              XA1B     9      1.0        9.0
              XA1C    26      1.0       26.0
              XAI1     3      1.0        3.0
             XAI1A     2      1.0        2.0
             XNOR2    10      1.0       10.0
             XNOR3     4      1.0        4.0
               XO1     5      1.0        5.0
              XO1A     2      1.0        2.0
              XOR2   161      1.0      161.0
              XOR3    28      1.0       28.0


            DFI1C0     1      1.0        1.0
            DFN1C0   184      1.0      184.0
            DFN1C1   470      1.0      470.0
            DFN1E0     1      1.0        1.0
          DFN1E0C0     8      1.0        8.0
          DFN1E0C1    96      1.0       96.0
            DFN1E1     1      1.0        1.0
          DFN1E1C0   218      1.0      218.0
          DFN1E1C1   798      1.0      798.0
          DFN1E1P0     6      1.0        6.0
            DFN1P0    53      1.0       53.0
            DFN1P1    25      1.0       25.0
          FIFO4K18     8      0.0        0.0
                   -----          ----------
             TOTAL  6042              5973.0


  IO Cell usage:
              cell count
            CLKBUF     1
             INBUF    18
            OUTBUF    19
                   -----
             TOTAL    38


Core Cells         : 5973 of 6144 (97%)
IO Cells           : 38

  RAM/ROM Usage Summary
Block Rams : 8 of 8 (100%)

Mapper successful!

At Mapper Exit (Real Time elapsed 0h:00m:15s; CPU Time elapsed 0h:00m:15s; Memory used current: 60MB peak: 216MB)

Process took 0h:00m:15s realtime, 0h:00m:15s cputime
# Fri Sep 05 17:35:31 2014

###########################################################]
``` ````
