# Supplementary material for: Implications for a Wireless, External Device System to Study Electrocorticography
Source: Sensors (Basel). 2017 Apr 4;17(4):761. doi: 10.3390/s17040761 (PMC5421721; doi:10.3390/s17040761)
Supplement: Supplementary file 1 [file sensors-17-00761-s001.zip › Supplement/RHA_FPGA_Firmware/IGLOO_RHA_05092014.zip!/IGLOO_RHA/synthesis/syntmp/IMPLANT_TOP_srr.htm]

 ```` ```
#Build: Synplify Pro I-2013.09M-SP1 , Build 034R, Jan 17 2014
#install: C:\Microsemi\Libero_v11.3\Synopsys\synplify_I201309MSP1
#OS: Windows 7 6.1
#Hostname: ITP-PC

#Implementation: synthesis

$ Start of Compile
#Fri Aug 01 16:16:59 2014

Synopsys VHDL Compiler, version comp201309rcp1, Build 078R, built Jan 14 2014
@N: :  | Running in 64-bit mode 
Copyright (C) 1994-2013 Synopsys, Inc. This software and the associated documentation are proprietary to Synopsys, Inc. This software may only be used in accordance with the terms and conditions of a written license agreement with Synopsys, Inc.  All other use or distribution of the software is strictly prohibited.

@N:CD720 : std.vhd(123) | Setting time resolution to ns
@N: : IMPLANT_TOP.vhd(27) | Top entity is set to IMPLANT_TOP.
File C:\Users\ITP\Desktop\IGLOO\IGLOO_RHA\hdl\RHA_DATA_COLLECTOR.vhd changed - recompiling
File C:\Users\ITP\Desktop\IGLOO\IGLOO_RHA\hdl\RHA_DATA_CHECKER.vhd changed - recompiling
File C:\Users\ITP\Desktop\IGLOO\IGLOO_RHA\hdl\RHA_TO_ZL_CONVERTER.vhd changed - recompiling
File C:\Users\ITP\Desktop\IGLOO\IGLOO_RHA\hdl\RHA_TESTVALUE_GENERATOR.vhd changed - recompiling
File C:\Users\ITP\Desktop\IGLOO\IGLOO_RHA\hdl\RHA_TEST_MODULATOR.vhd changed - recompiling
File C:\Users\ITP\Desktop\IGLOO\IGLOO_RHA\hdl\RHA_ERROR_WATCHDOG.vhd changed - recompiling
File C:\Users\ITP\Desktop\IGLOO\IGLOO_RHA\smartgen\FIFO\FIFO.vhd changed - recompiling
File C:\Microsemi\Libero_v11.3\Synopsys\synplify_I201309MSP1\lib\proasic\igloo.vhd changed - recompiling
File C:\Users\ITP\Desktop\IGLOO\IGLOO_RHA\hdl\CONTROL_NEXUS.vhd changed - recompiling
File C:\Users\ITP\Desktop\IGLOO\IGLOO_RHA\hdl\RHA_ARRAY.vhd changed - recompiling
File C:\Users\ITP\Desktop\IGLOO\IGLOO_RHA\hdl\RHA_TESTMODULE.vhd changed - recompiling
File C:\Users\ITP\Desktop\IGLOO\IGLOO_RHA\hdl\DATA_ACQUISITION_BLOCK.vhd changed - recompiling
File C:\Users\ITP\Desktop\IGLOO\IGLOO_RHA\hdl\IMPLANT_TOP.vhd changed - recompiling
@W:CD645 : Zarlink_SPI_Module.vhd(2) | Ignoring undefined library unisim
@W:CD642 : Zarlink_SPI_Module.vhd(6) | Ignoring use clause - library unisim not found ...
@W:CD645 : ZARLINK_CONNECT_INIT.vhd(21) | Ignoring undefined library unisim
@W:CD642 : ZARLINK_CONNECT_INIT.vhd(25) | Ignoring use clause - library unisim not found ...
@W:CD645 : ORGANIZER.vhd(21) | Ignoring undefined library unisim
@W:CD642 : ORGANIZER.vhd(25) | Ignoring use clause - library unisim not found ...
@W:CD645 : COMMAND_RECEIVER.vhd(21) | Ignoring undefined library unisim
@W:CD642 : COMMAND_RECEIVER.vhd(25) | Ignoring use clause - library unisim not found ...
@W:CD645 : RHA_DATA_COLLECTOR.vhd(21) | Ignoring undefined library unisim
@W:CD642 : RHA_DATA_COLLECTOR.vhd(25) | Ignoring use clause - library unisim not found ...
@W:CD645 : RHA_DATA_CHECKER.vhd(21) | Ignoring undefined library unisim
@W:CD642 : RHA_DATA_CHECKER.vhd(25) | Ignoring use clause - library unisim not found ...
@W:CD645 : RHA_TO_ZL_CONVERTER.vhd(21) | Ignoring undefined library unisim
@W:CD642 : RHA_TO_ZL_CONVERTER.vhd(25) | Ignoring use clause - library unisim not found ...
@W:CD645 : RHA_TESTVALUE_GENERATOR.vhd(21) | Ignoring undefined library unisim
@W:CD642 : RHA_TESTVALUE_GENERATOR.vhd(25) | Ignoring use clause - library unisim not found ...
@W:CD645 : RHA_TEST_MODULATOR.vhd(21) | Ignoring undefined library unisim
@W:CD642 : RHA_TEST_MODULATOR.vhd(25) | Ignoring use clause - library unisim not found ...
@W:CD645 : RHA_ERROR_WATCHDOG.vhd(21) | Ignoring undefined library unisim
@W:CD642 : RHA_ERROR_WATCHDOG.vhd(25) | Ignoring use clause - library unisim not found ...
@W:CD645 : CONTROL_NEXUS.vhd(21) | Ignoring undefined library unisim
@W:CD642 : CONTROL_NEXUS.vhd(25) | Ignoring use clause - library unisim not found ...
@W:CD645 : RHA_ARRAY.vhd(21) | Ignoring undefined library unisim
@W:CD642 : RHA_ARRAY.vhd(25) | Ignoring use clause - library unisim not found ...
@W:CD645 : RHA_TESTMODULE.vhd(21) | Ignoring undefined library unisim
@W:CD642 : RHA_TESTMODULE.vhd(25) | Ignoring use clause - library unisim not found ...
@W:CD645 : DATA_ACQUISITION_BLOCK.vhd(21) | Ignoring undefined library unisim
@W:CD642 : DATA_ACQUISITION_BLOCK.vhd(25) | Ignoring use clause - library unisim not found ...
@W:CD645 : IMPLANT_TOP.vhd(21) | Ignoring undefined library unisim
@W:CD642 : IMPLANT_TOP.vhd(25) | Ignoring use clause - library unisim not found ...
VHDL syntax check successful!
@N:CD630 : IMPLANT_TOP.vhd(27) | Synthesizing work.implant_top.behavioral 
@N:CD364 : IMPLANT_TOP.vhd(235) | Removed redundant assignment
@N:CD630 : DATA_ACQUISITION_BLOCK.vhd(27) | Synthesizing work.data_acquisition_block.behavioral 
@N:CD630 : RHA_ERROR_WATCHDOG.vhd(27) | Synthesizing work.rha_error_watchdog.behavioral 
@N:CD364 : RHA_ERROR_WATCHDOG.vhd(95) | Removed redundant assignment
@N:CD364 : RHA_ERROR_WATCHDOG.vhd(96) | Removed redundant assignment
@N:CD364 : RHA_ERROR_WATCHDOG.vhd(97) | Removed redundant assignment
@N:CD364 : RHA_ERROR_WATCHDOG.vhd(98) | Removed redundant assignment
@N:CD364 : RHA_ERROR_WATCHDOG.vhd(99) | Removed redundant assignment
@N:CD364 : RHA_ERROR_WATCHDOG.vhd(100) | Removed redundant assignment
@N:CD364 : RHA_ERROR_WATCHDOG.vhd(101) | Removed redundant assignment
Post processing for work.rha_error_watchdog.behavioral
@N:CD630 : RHA_TESTMODULE.vhd(28) | Synthesizing work.rha_testmodule.behavioral 
@N:CD630 : RHA_TEST_MODULATOR.vhd(27) | Synthesizing work.rha_test_modulator.behavioral 
@N:CD232 : RHA_TEST_MODULATOR.vhd(43) | Using gray code encoding for type state_type
@N:CD364 : RHA_TEST_MODULATOR.vhd(87) | Removed redundant assignment
@N:CD364 : RHA_TEST_MODULATOR.vhd(88) | Removed redundant assignment
@W:CD604 : RHA_TEST_MODULATOR.vhd(146) | OTHERS clause is not synthesized 
@W:CD604 : RHA_TEST_MODULATOR.vhd(220) | OTHERS clause is not synthesized 
Post processing for work.rha_test_modulator.behavioral
@A:CL282 : RHA_TEST_MODULATOR.vhd(154) | Feedback mux created for signal SPIMACHINE_STATE[4:0] -- possible set/reset assignment for signal missing. Specifying a reset value will improve timing and area.
@N:CD630 : RHA_TESTVALUE_GENERATOR.vhd(27) | Synthesizing work.rha_testvalue_generator.behavioral 
@N:CD364 : RHA_TESTVALUE_GENERATOR.vhd(130) | Removed redundant assignment
@N:CD364 : RHA_TESTVALUE_GENERATOR.vhd(138) | Removed redundant assignment
@N:CD364 : RHA_TESTVALUE_GENERATOR.vhd(142) | Removed redundant assignment
@N:CD364 : RHA_TESTVALUE_GENERATOR.vhd(145) | Removed redundant assignment
@N:CD364 : RHA_TESTVALUE_GENERATOR.vhd(146) | Removed redundant assignment
@W:CD604 : RHA_TESTVALUE_GENERATOR.vhd(205) | OTHERS clause is not synthesized 
@W:CD638 : RHA_TESTVALUE_GENERATOR.vhd(60) | Signal xor_address is undriven 
@W:CD638 : RHA_TESTVALUE_GENERATOR.vhd(61) | Signal xor_header is undriven 
@W:CD638 : RHA_TESTVALUE_GENERATOR.vhd(82) | Signal nextvalue_last is undriven 
Post processing for work.rha_testvalue_generator.behavioral
Post processing for work.rha_testmodule.behavioral
@N:CD630 : RHA_TO_ZL_CONVERTER.vhd(28) | Synthesizing work.rha_to_zl_converter.behavioral 
@N:CD232 : RHA_TO_ZL_CONVERTER.vhd(112) | Using gray code encoding for type state_type
@N:CD364 : RHA_TO_ZL_CONVERTER.vhd(261) | Removed redundant assignment
@N:CD364 : RHA_TO_ZL_CONVERTER.vhd(262) | Removed redundant assignment
@N:CD364 : RHA_TO_ZL_CONVERTER.vhd(300) | Removed redundant assignment
@N:CD364 : RHA_TO_ZL_CONVERTER.vhd(328) | Removed redundant assignment
@N:CD364 : RHA_TO_ZL_CONVERTER.vhd(329) | Removed redundant assignment
@N:CD364 : RHA_TO_ZL_CONVERTER.vhd(331) | Removed redundant assignment
@N:CD364 : RHA_TO_ZL_CONVERTER.vhd(332) | Removed redundant assignment
@N:CD364 : RHA_TO_ZL_CONVERTER.vhd(333) | Removed redundant assignment
@N:CD364 : RHA_TO_ZL_CONVERTER.vhd(334) | Removed redundant assignment
@N:CD364 : RHA_TO_ZL_CONVERTER.vhd(336) | Removed redundant assignment
@N:CD364 : RHA_TO_ZL_CONVERTER.vhd(348) | Removed redundant assignment
@N:CD364 : RHA_TO_ZL_CONVERTER.vhd(583) | Removed redundant assignment
@W:CD604 : RHA_TO_ZL_CONVERTER.vhd(615) | OTHERS clause is not synthesized 
Post processing for work.rha_to_zl_converter.behavioral
@A:CL282 : RHA_TO_ZL_CONVERTER.vhd(314) | Feedback mux created for signal SPIMACHINE_STATE[4:0] -- possible set/reset assignment for signal missing. Specifying a reset value will improve timing and area.
@A:CL282 : RHA_TO_ZL_CONVERTER.vhd(295) | Feedback mux created for signal NewBlock_LAST -- possible set/reset assignment for signal missing. Specifying a reset value will improve timing and area.
@N:CD630 : RHA_ARRAY.vhd(32) | Synthesizing work.rha_array.behavioral 
@N:CD364 : RHA_ARRAY.vhd(450) | Removed redundant assignment
@N:CD364 : RHA_ARRAY.vhd(451) | Removed redundant assignment
@N:CD364 : RHA_ARRAY.vhd(452) | Removed redundant assignment
@N:CD364 : RHA_ARRAY.vhd(453) | Removed redundant assignment
@N:CD364 : RHA_ARRAY.vhd(454) | Removed redundant assignment
@N:CD364 : RHA_ARRAY.vhd(459) | Removed redundant assignment
@N:CD364 : RHA_ARRAY.vhd(461) | Removed redundant assignment
@N:CD364 : RHA_ARRAY.vhd(463) | Removed redundant assignment
@N:CD364 : RHA_ARRAY.vhd(464) | Removed redundant assignment
@N:CD364 : RHA_ARRAY.vhd(465) | Removed redundant assignment
@N:CD364 : RHA_ARRAY.vhd(466) | Removed redundant assignment
@N:CD364 : RHA_ARRAY.vhd(467) | Removed redundant assignment
@N:CD364 : RHA_ARRAY.vhd(468) | Removed redundant assignment
@N:CD364 : RHA_ARRAY.vhd(469) | Removed redundant assignment
@N:CD364 : RHA_ARRAY.vhd(470) | Removed redundant assignment
@N:CD364 : RHA_ARRAY.vhd(565) | Removed redundant assignment
@N:CD364 : RHA_ARRAY.vhd(566) | Removed redundant assignment
@W:CD638 : RHA_ARRAY.vhd(155) | Signal channel_number is undriven 
@N:CD630 : RHA_DATA_CHECKER.vhd(36) | Synthesizing work.rha_data_checker.behavioral 
Post processing for work.rha_data_checker.behavioral
@N:CD630 : RHA_DATA_COLLECTOR.vhd(30) | Synthesizing work.rha_data_collector.behavioral 
@N:CD233 : RHA_DATA_COLLECTOR.vhd(57) | Using sequential encoding for type state_type
@N:CD364 : RHA_DATA_COLLECTOR.vhd(93) | Removed redundant assignment
@N:CD364 : RHA_DATA_COLLECTOR.vhd(96) | Removed redundant assignment
@W:CD604 : RHA_DATA_COLLECTOR.vhd(128) | OTHERS clause is not synthesized 
@N:CD364 : RHA_DATA_COLLECTOR.vhd(149) | Removed redundant assignment
Post processing for work.rha_data_collector.behavioral
@A:CL282 : RHA_DATA_COLLECTOR.vhd(67) | Feedback mux created for signal SPIMACHINE_STATE[0:0] -- possible set/reset assignment for signal missing. Specifying a reset value will improve timing and area.
Post processing for work.rha_array.behavioral
@W:CL190 : RHA_ARRAY.vhd(151) | Optimizing register bit AddressCheckInfo(3) to a constant 0
@W:CL260 : RHA_ARRAY.vhd(151) | Pruning register bit 3 of AddressCheckInfo(79 downto 0)  
Post processing for work.data_acquisition_block.behavioral
@W:CL117 : DATA_ACQUISITION_BLOCK.vhd(154) | Latch generated from process for signal RHA_ADC_clk_TEST; possible missing assignment in an if or case statement.
@W:CL117 : DATA_ACQUISITION_BLOCK.vhd(82) | Latch generated from process for signal RHA_ADC_clk(7 downto 0); possible missing assignment in an if or case statement.
@N:CD630 : CONTROL_NEXUS.vhd(28) | Synthesizing work.control_nexus.behavioral 
@W:CD638 : CONTROL_NEXUS.vhd(116) | Signal fifo_in_readenable_internal is undriven 
@N:CD630 : COMMAND_RECEIVER.vhd(28) | Synthesizing work.command_receiver.behavioral 
@N:CD231 : COMMAND_RECEIVER.vhd(85) | Using onehot encoding for type state_type (sm_idle="100000000000000000000000")
@N:CD364 : COMMAND_RECEIVER.vhd(170) | Removed redundant assignment
@N:CD364 : COMMAND_RECEIVER.vhd(171) | Removed redundant assignment
@N:CD364 : COMMAND_RECEIVER.vhd(172) | Removed redundant assignment
@N:CD364 : COMMAND_RECEIVER.vhd(173) | Removed redundant assignment
@N:CD364 : COMMAND_RECEIVER.vhd(174) | Removed redundant assignment
@N:CD364 : COMMAND_RECEIVER.vhd(175) | Removed redundant assignment
@N:CD364 : COMMAND_RECEIVER.vhd(176) | Removed redundant assignment
@W:CD604 : COMMAND_RECEIVER.vhd(406) | OTHERS clause is not synthesized 
@W:CD604 : COMMAND_RECEIVER.vhd(452) | OTHERS clause is not synthesized 
@W:CD604 : COMMAND_RECEIVER.vhd(496) | OTHERS clause is not synthesized 
@N:CD364 : COMMAND_RECEIVER.vhd(520) | Removed redundant assignment
@N:CD364 : COMMAND_RECEIVER.vhd(521) | Removed redundant assignment
@N:CD364 : COMMAND_RECEIVER.vhd(543) | Removed redundant assignment
@N:CD364 : COMMAND_RECEIVER.vhd(544) | Removed redundant assignment
Post processing for work.command_receiver.behavioral
@N:CD630 : ORGANIZER.vhd(28) | Synthesizing work.organizer.behavioral 
@N:CD231 : ORGANIZER.vhd(88) | Using onehot encoding for type state_type (sm_reset="100000000000000")
@N:CD364 : ORGANIZER.vhd(139) | Removed redundant assignment
Post processing for work.organizer.behavioral
@N:CD630 : ZARLINK_CONNECT_INIT.vhd(27) | Synthesizing work.zarlink_connect_init.behavioral 
@N:CD232 : ZARLINK_CONNECT_INIT.vhd(87) | Using gray code encoding for type state_type
@N:CD364 : ZARLINK_CONNECT_INIT.vhd(242) | Removed redundant assignment
@N:CD364 : ZARLINK_CONNECT_INIT.vhd(251) | Removed redundant assignment
@N:CD364 : ZARLINK_CONNECT_INIT.vhd(253) | Removed redundant assignment
@N:CD364 : ZARLINK_CONNECT_INIT.vhd(254) | Removed redundant assignment
@N:CD364 : ZARLINK_CONNECT_INIT.vhd(255) | Removed redundant assignment
@N:CD364 : ZARLINK_CONNECT_INIT.vhd(256) | Removed redundant assignment
@N:CD364 : ZARLINK_CONNECT_INIT.vhd(258) | Removed redundant assignment
@N:CD364 : ZARLINK_CONNECT_INIT.vhd(259) | Removed redundant assignment
@W:CD604 : ZARLINK_CONNECT_INIT.vhd(1030) | OTHERS clause is not synthesized 
@N:CD364 : ZARLINK_CONNECT_INIT.vhd(1042) | Removed redundant assignment
Post processing for work.zarlink_connect_init.behavioral
@N:CD630 : Zarlink_SPI_Module.vhd(8) | Synthesizing work.zarlink_spi_module.behavioral 
@N:CD232 : Zarlink_SPI_Module.vhd(48) | Using gray code encoding for type state_type
@N:CD364 : Zarlink_SPI_Module.vhd(131) | Removed redundant assignment
@N:CD364 : Zarlink_SPI_Module.vhd(137) | Removed redundant assignment
@N:CD364 : Zarlink_SPI_Module.vhd(138) | Removed redundant assignment
@N:CD364 : Zarlink_SPI_Module.vhd(139) | Removed redundant assignment
@N:CD364 : Zarlink_SPI_Module.vhd(142) | Removed redundant assignment
@W:CD604 : Zarlink_SPI_Module.vhd(396) | OTHERS clause is not synthesized 
Post processing for work.zarlink_spi_module.behavioral
Post processing for work.control_nexus.behavioral
@N:CD630 : FIFO.vhd(8) | Synthesizing work.fifo.def_arch 
@N:CD630 : igloo.vhd(2722) | Synthesizing igloo.vcc.syn_black_box 
Post processing for igloo.vcc.syn_black_box
@N:CD630 : igloo.vhd(1787) | Synthesizing igloo.gnd.syn_black_box 
Post processing for igloo.gnd.syn_black_box
@N:CD630 : igloo.vhd(2198) | Synthesizing igloo.or2.syn_black_box 
Post processing for igloo.or2.syn_black_box
@N:CD630 : igloo.vhd(1934) | Synthesizing igloo.inv.syn_black_box 
Post processing for igloo.inv.syn_black_box
@N:CD630 : igloo.vhd(3039) | Synthesizing igloo.fifo4k18.syn_black_box 
Post processing for igloo.fifo4k18.syn_black_box
@N:CD630 : igloo.vhd(13) | Synthesizing igloo.and2.syn_black_box 
Post processing for igloo.and2.syn_black_box
@N:CD630 : igloo.vhd(2032) | Synthesizing igloo.nand2.syn_black_box 
Post processing for igloo.nand2.syn_black_box
@N:CD630 : igloo.vhd(2040) | Synthesizing igloo.nand2a.syn_black_box 
Post processing for igloo.nand2a.syn_black_box
Post processing for work.fifo.def_arch
Post processing for work.implant_top.behavioral
@A:CL282 : IMPLANT_TOP.vhd(189) | Feedback mux created for signal FIFO_IN_READENABLE_LAST -- possible set/reset assignment for signal missing. Specifying a reset value will improve timing and area.
@N:CL201 : Zarlink_SPI_Module.vhd(110) | Trying to extract state machine for register FSM_STATE
Extracted state machine for register FSM_STATE
State machine has 44 reachable states with original encodings of:
   000000
   000001
   000010
   000011
   000100
   000101
   000110
   000111
   001000
   001001
   001010
   001011
   001100
   001101
   001110
   001111
   010000
   010001
   010010
   010011
   010100
   010101
   010110
   010111
   011000
   011001
   011010
   011011
   011100
   011101
   011110
   011111
   110000
   110001
   110010
   110011
   110100
   110101
   110110
   110111
   111100
   111101
   111110
   111111
@N:CL201 : ZARLINK_CONNECT_INIT.vhd(207) | Trying to extract state machine for register FSM_STATE
Extracted state machine for register FSM_STATE
State machine has 36 reachable states with original encodings of:
   000000
   000001
   000010
   000011
   000100
   000101
   000110
   000111
   001000
   001001
   001010
   001011
   001100
   001101
   001110
   001111
   010000
   010001
   010010
   010011
   010100
   010101
   010110
   010111
   011000
   011001
   011010
   011011
   011100
   011101
   011110
   011111
   110000
   110001
   110010
   110011
@N:CL201 : ORGANIZER.vhd(118) | Trying to extract state machine for register SPIMACHINE_STATE
Extracted state machine for register SPIMACHINE_STATE
State machine has 15 reachable states with original encodings of:
   000000000000001
   000000000000010
   000000000000100
   000000000001000
   000000000010000
   000000000100000
   000000001000000
   000000010000000
   000000100000000
   000001000000000
   000010000000000
   000100000000000
   001000000000000
   010000000000000
   100000000000000
@N:CL201 : COMMAND_RECEIVER.vhd(157) | Trying to extract state machine for register SPIMACHINE_STATE
Extracted state machine for register SPIMACHINE_STATE
State machine has 24 reachable states with original encodings of:
   000000000000000000000001
   000000000000000000000010
   000000000000000000000100
   000000000000000000001000
   000000000000000000010000
   000000000000000000100000
   000000000000000001000000
   000000000000000010000000
   000000000000000100000000
   000000000000001000000000
   000000000000010000000000
   000000000000100000000000
   000000000001000000000000
   000000000010000000000000
   000000000100000000000000
   000000001000000000000000
   000000010000000000000000
   000000100000000000000000
   000001000000000000000000
   000010000000000000000000
   000100000000000000000000
   001000000000000000000000
   010000000000000000000000
   100000000000000000000000
@W:CL189 : RHA_DATA_COLLECTOR.vhd(67) | Register bit SPIMACHINE_STATE(sm_wait) is always 1, optimizing ...
@W:CL190 : RHA_ARRAY.vhd(151) | Optimizing register bit AddressCheckInfo(8) to a constant 0
@W:CL260 : RHA_ARRAY.vhd(151) | Pruning register bit 8 of AddressCheckInfo(79 downto 4)  
@W:CL190 : RHA_ARRAY.vhd(151) | Optimizing register bit AddressCheckInfo(13) to a constant 0
@W:CL260 : RHA_ARRAY.vhd(151) | Pruning register bit 13 of AddressCheckInfo(79 downto 9)  
@W:CL190 : RHA_ARRAY.vhd(151) | Optimizing register bit AddressCheckInfo(18) to a constant 0
@W:CL260 : RHA_ARRAY.vhd(151) | Pruning register bit 18 of AddressCheckInfo(79 downto 14)  
@W:CL190 : RHA_ARRAY.vhd(151) | Optimizing register bit AddressCheckInfo(23) to a constant 0
@W:CL260 : RHA_ARRAY.vhd(151) | Pruning register bit 23 of AddressCheckInfo(79 downto 19)  
@W:CL279 : RHA_ARRAY.vhd(151) | Pruning register bits 2 to 1 of AddressCheckInfo(2 downto 0)  
@W:CL260 : RHA_ARRAY.vhd(151) | Pruning register bit 7 of AddressCheckInfo(7 downto 4)  
@W:CL190 : RHA_ARRAY.vhd(151) | Optimizing register bit AddressCheckInfo(28) to a constant 0
@W:CL260 : RHA_ARRAY.vhd(151) | Pruning register bit 28 of AddressCheckInfo(79 downto 24)  
@N:CL201 : RHA_TO_ZL_CONVERTER.vhd(314) | Trying to extract state machine for register SPIMACHINE_STATE
@W:CL159 : RHA_TO_ZL_CONVERTER.vhd(72) | Input Clk_SLOW is unused
@N:CL201 : RHA_TEST_MODULATOR.vhd(154) | Trying to extract state machine for register SPIMACHINE_STATE
@W:CL190 : IMPLANT_TOP.vhd(206) | Optimizing register bit DEBUG_COUNTER(3) to a constant 0
@W:CL260 : IMPLANT_TOP.vhd(206) | Pruning register bit 3 of DEBUG_COUNTER(3 downto 0)  
@END

At c_vhdl Exit (Real Time elapsed 0h:00m:01s; CPU Time elapsed 0h:00m:01s; Memory used current: 85MB peak: 91MB)

Process took 0h:00m:01s realtime, 0h:00m:01s cputime
# Fri Aug 01 16:17:00 2014

###########################################################]
Pre-mapping Report

Synopsys Microsemi Technology Pre-mapping, Version mapact, Build 1154R, Built Jan 20 2014 10:14:08
Copyright (C) 1994-2013, Synopsys, Inc. This software and the associated documentation are proprietary to Synopsys, Inc. This software may only be used in accordance with the terms and conditions of a written license agreement with Synopsys, Inc.  All other use or distribution of the software is strictly prohibited.
Product Version I-2013.09M-SP1 

Mapper Startup Complete (Real Time elapsed 0h:00m:00s; CPU Time elapsed 0h:00m:00s; Memory used current: 98MB peak: 99MB)

Linked File: IMPLANT_TOP_scck.rpt
Printing clock  summary report in "C:\Users\ITP\Desktop\IGLOO\IGLOO_RHA\synthesis\IMPLANT_TOP_scck.rpt" file 
@N:MF248 :  | Running in 64-bit mode. 
@N:MF667 :  | Clock conversion disabled  

Design Input Complete (Real Time elapsed 0h:00m:00s; CPU Time elapsed 0h:00m:00s; Memory used current: 102MB peak: 104MB)


Mapper Initialization Complete (Real Time elapsed 0h:00m:00s; CPU Time elapsed 0h:00m:00s; Memory used current: 102MB peak: 104MB)

@N:BN362 : rha_test_modulator.vhd(79) | Removing sequential instance NextValue of view:PrimLib.dffr(prim) in hierarchy view:work.RHA_TEST_MODULATOR_RHA_TEST_MOD_1(behavioral) because there are no references to its outputs 
@N:BN362 : rha_test_modulator.vhd(79) | Removing sequential instance NextValue of view:PrimLib.dffr(prim) in hierarchy view:work.RHA_TEST_MODULATOR_RHA_TEST_MOD_2(behavioral) because there are no references to its outputs 
@N:BN362 : rha_test_modulator.vhd(79) | Removing sequential instance NextValue of view:PrimLib.dffr(prim) in hierarchy view:work.RHA_TEST_MODULATOR_RHA_TEST_MOD_3(behavioral) because there are no references to its outputs 
@N:BN362 : rha_test_modulator.vhd(79) | Removing sequential instance NextValue of view:PrimLib.dffr(prim) in hierarchy view:work.RHA_TEST_MODULATOR_RHA_TEST_MOD_4(behavioral) because there are no references to its outputs 
@N:BN362 : rha_test_modulator.vhd(79) | Removing sequential instance NextValue of view:PrimLib.dffr(prim) in hierarchy view:work.RHA_TEST_MODULATOR_RHA_TEST_MOD_5(behavioral) because there are no references to its outputs 
@N:BN362 : rha_test_modulator.vhd(79) | Removing sequential instance NextValue of view:PrimLib.dffr(prim) in hierarchy view:work.RHA_TEST_MODULATOR_RHA_TEST_MOD_6(behavioral) because there are no references to its outputs 
@N:BN362 : rha_test_modulator.vhd(79) | Removing sequential instance NextValue of view:PrimLib.dffr(prim) in hierarchy view:work.RHA_TEST_MODULATOR_RHA_TEST_MOD_7(behavioral) because there are no references to its outputs 
@N:BN362 : rha_test_modulator.vhd(154) | Removing sequential instance ADC_SYNC of view:PrimLib.dffr(prim) in hierarchy view:work.RHA_TEST_MODULATOR_RHA_TEST_MOD_0(behavioral) because there are no references to its outputs 
@N:BN362 : rha_test_modulator.vhd(154) | Removing sequential instance ADC_SYNC of view:PrimLib.dffr(prim) in hierarchy view:work.RHA_TEST_MODULATOR_RHA_TEST_MOD_1(behavioral) because there are no references to its outputs 
@N:BN362 : rha_test_modulator.vhd(154) | Removing sequential instance ADC_SYNC of view:PrimLib.dffr(prim) in hierarchy view:work.RHA_TEST_MODULATOR_RHA_TEST_MOD_2(behavioral) because there are no references to its outputs 
@N:BN362 : rha_test_modulator.vhd(154) | Removing sequential instance ADC_SYNC of view:PrimLib.dffr(prim) in hierarchy view:work.RHA_TEST_MODULATOR_RHA_TEST_MOD_3(behavioral) because there are no references to its outputs 
@N:BN362 : rha_test_modulator.vhd(154) | Removing sequential instance ADC_SYNC of view:PrimLib.dffr(prim) in hierarchy view:work.RHA_TEST_MODULATOR_RHA_TEST_MOD_4(behavioral) because there are no references to its outputs 
@N:BN362 : rha_test_modulator.vhd(154) | Removing sequential instance ADC_SYNC of view:PrimLib.dffr(prim) in hierarchy view:work.RHA_TEST_MODULATOR_RHA_TEST_MOD_5(behavioral) because there are no references to its outputs 
@N:BN362 : rha_test_modulator.vhd(154) | Removing sequential instance ADC_SYNC of view:PrimLib.dffr(prim) in hierarchy view:work.RHA_TEST_MODULATOR_RHA_TEST_MOD_6(behavioral) because there are no references to its outputs 
@N:BN362 : rha_test_modulator.vhd(154) | Removing sequential instance ADC_SYNC of view:PrimLib.dffr(prim) in hierarchy view:work.RHA_TEST_MODULATOR_RHA_TEST_MOD_7(behavioral) because there are no references to its outputs 


Clock Summary
**************

Start     Requested     Requested     Clock     Clock
Clock     Frequency     Period        Type      Group
-----------------------------------------------------
=====================================================

@W:MT532 : fifo.vhd(443) | Found signal identified as System clock which controls 1650 sequential elements including MyFIFO.\\FIFOBLOCK\[1\]\\.  Using this clock, which has no specified timing constraint, can adversely impact design performance. 

Finished Pre Mapping Phase.
@N:BN225 :  | Writing default property annotation file C:\Users\ITP\Desktop\IGLOO\IGLOO_RHA\synthesis\IMPLANT_TOP.sap. 
Pre-mapping successful!

At Mapper Exit (Real Time elapsed 0h:00m:00s; CPU Time elapsed 0h:00m:00s; Memory used current: 50MB peak: 113MB)

Process took 0h:00m:01s realtime, 0h:00m:01s cputime
# Fri Aug 01 16:17:02 2014

###########################################################]
Map & Optimize Report

Synopsys Microsemi Technology Mapper, Version mapact, Build 1154R, Built Jan 20 2014 10:14:08
Copyright (C) 1994-2013, Synopsys, Inc. This software and the associated documentation are proprietary to Synopsys, Inc. This software may only be used in accordance with the terms and conditions of a written license agreement with Synopsys, Inc.  All other use or distribution of the software is strictly prohibited.
Product Version I-2013.09M-SP1 

Mapper Startup Complete (Real Time elapsed 0h:00m:00s; CPU Time elapsed 0h:00m:00s; Memory used current: 99MB peak: 99MB)

@N:MF248 :  | Running in 64-bit mode. 
@N:MF667 :  | Clock conversion disabled  

Design Input Complete (Real Time elapsed 0h:00m:00s; CPU Time elapsed 0h:00m:00s; Memory used current: 99MB peak: 101MB)


Mapper Initialization Complete (Real Time elapsed 0h:00m:00s; CPU Time elapsed 0h:00m:00s; Memory used current: 99MB peak: 101MB)


Starting Optimization and Mapping (Real Time elapsed 0h:00m:00s; CPU Time elapsed 0h:00m:00s; Memory used current: 111MB peak: 111MB)


Available hyper_sources - for debug and ip models
	None Found

@W:MT462 : command_receiver.vhd(504) | Net Inst_CONTROL_NEXUS.Inst_COMMAND_RECEIVER.Clk_Reduced appears to be an unidentified clock source. Assuming default frequency. 

Finished RTL optimizations (Real Time elapsed 0h:00m:00s; CPU Time elapsed 0h:00m:00s; Memory used current: 113MB peak: 115MB)

@N: : implant_top.vhd(231) | Found counter in view:work.IMPLANT_TOP(behavioral) inst RHA_RESET_COUNTER[4:0]
@N: : implant_top.vhd(206) | Found counter in view:work.IMPLANT_TOP(behavioral) inst DEBUG_COUNTER[2:0]
@N: : zarlink_spi_module.vhd(110) | Found counter in view:work.Zarlink_SPI_Module(behavioral) inst Counter[3:0]
Encoding state machine FSM_STATE[0:43] (view:work.Zarlink_SPI_Module(behavioral))
original code -> new code
   000000 -> 000000
   000001 -> 000001
   000010 -> 000011
   000011 -> 000010
   000100 -> 000110
   000101 -> 000111
   000110 -> 000101
   000111 -> 000100
   001000 -> 001100
   001001 -> 001101
   001010 -> 001111
   001011 -> 001110
   001100 -> 001010
   001101 -> 001011
   001110 -> 001001
   001111 -> 001000
   010000 -> 011000
   010001 -> 011001
   010010 -> 011011
   010011 -> 011010
   010100 -> 011110
   010101 -> 011111
   010110 -> 011101
   010111 -> 011100
   011000 -> 010100
   011001 -> 010101
   011010 -> 010111
   011011 -> 010110
   011100 -> 010010
   011101 -> 010011
   011110 -> 010001
   011111 -> 010000
   110000 -> 110000
   110001 -> 110001
   110010 -> 110011
   110011 -> 110010
   110100 -> 110110
   110101 -> 110111
   110110 -> 110101
   110111 -> 110100
   111100 -> 111100
   111101 -> 111101
   111110 -> 111111
   111111 -> 111110
@N: : zarlink_connect_init.vhd(207) | Found counter in view:work.ZARLINK_CONNECT_INIT(behavioral) inst Zarlink_ConnectionTimeoutCounter[31:0]
@N: : zarlink_connect_init.vhd(207) | Found counter in view:work.ZARLINK_CONNECT_INIT(behavioral) inst Zarlink_ResetCounter[23:0]
@N: : zarlink_connect_init.vhd(207) | Found counter in view:work.ZARLINK_CONNECT_INIT(behavioral) inst Zarlink_Retry_Counter[16:0]
Encoding state machine FSM_STATE[0:35] (view:work.ZARLINK_CONNECT_INIT(behavioral))
original code -> new code
   000000 -> 000000
   000001 -> 000001
   000010 -> 000011
   000011 -> 000010
   000100 -> 000110
   000101 -> 000111
   000110 -> 000101
   000111 -> 000100
   001000 -> 001100
   001001 -> 001101
   001010 -> 001111
   001011 -> 001110
   001100 -> 001010
   001101 -> 001011
   001110 -> 001001
   001111 -> 001000
   010000 -> 011000
   010001 -> 011001
   010010 -> 011011
   010011 -> 011010
   010100 -> 011110
   010101 -> 011111
   010110 -> 011101
   010111 -> 011100
   011000 -> 010100
   011001 -> 010101
   011010 -> 010111
   011011 -> 010110
   011100 -> 010010
   011101 -> 010011
   011110 -> 010001
   011111 -> 010000
   110000 -> 110000
   110001 -> 110001
   110010 -> 110011
   110011 -> 110010
@N: : organizer.vhd(118) | Found counter in view:work.ORGANIZER(behavioral) inst WaitForXCyles_Counter[17:0]
@N: : organizer.vhd(118) | Found counter in view:work.ORGANIZER(behavioral) inst CheckOutgoingBuffer_SIZE_COPY[6:0]
Encoding state machine SPIMACHINE_STATE[0:14] (view:work.ORGANIZER(behavioral))
original code -> new code
   000000000000001 -> 000000000000001
   000000000000010 -> 000000000000010
   000000000000100 -> 000000000000100
   000000000001000 -> 000000000001000
   000000000010000 -> 000000000010000
   000000000100000 -> 000000000100000
   000000001000000 -> 000000001000000
   000000010000000 -> 000000010000000
   000000100000000 -> 000000100000000
   000001000000000 -> 000001000000000
   000010000000000 -> 000010000000000
   000100000000000 -> 000100000000000
   001000000000000 -> 001000000000000
   010000000000000 -> 010000000000000
   100000000000000 -> 100000000000000
@N:MO106 : command_receiver.vhd(463) | Found ROM, 'BITMASK_SAMPLE_TOP_INTERNAL_16[14:1]', 16 words by 14 bits 
@N:MO106 : command_receiver.vhd(419) | Found ROM, 'BITMASK_SAMPLE_INTERNAL_17[14:9]', 16 words by 6 bits 
@N:MO106 : command_receiver.vhd(419) | Found ROM, 'BITMASK_SAMPLE_INTERNAL_17[7:1]', 16 words by 7 bits 
Encoding state machine SPIMACHINE_STATE[0:23] (view:work.COMMAND_RECEIVER(behavioral))
original code -> new code
   000000000000000000000001 -> 000000000000000000000001
   000000000000000000000010 -> 000000000000000000000010
   000000000000000000000100 -> 000000000000000000000100
   000000000000000000001000 -> 000000000000000000001000
   000000000000000000010000 -> 000000000000000000010000
   000000000000000000100000 -> 000000000000000000100000
   000000000000000001000000 -> 000000000000000001000000
   000000000000000010000000 -> 000000000000000010000000
   000000000000000100000000 -> 000000000000000100000000
   000000000000001000000000 -> 000000000000001000000000
   000000000000010000000000 -> 000000000000010000000000
   000000000000100000000000 -> 000000000000100000000000
   000000000001000000000000 -> 000000000001000000000000
   000000000010000000000000 -> 000000000010000000000000
   000000000100000000000000 -> 000000000100000000000000
   000000001000000000000000 -> 000000001000000000000000
   000000010000000000000000 -> 000000010000000000000000
   000000100000000000000000 -> 000000100000000000000000
   000001000000000000000000 -> 000001000000000000000000
   000010000000000000000000 -> 000010000000000000000000
   000100000000000000000000 -> 000100000000000000000000
   001000000000000000000000 -> 001000000000000000000000
   010000000000000000000000 -> 010000000000000000000000
   100000000000000000000000 -> 100000000000000000000000
@N:MF238 : command_receiver.vhd(530) | Found 8-bit incrementor, 'un7_counter[7:0]'
@W:MO160 : rha_array.vhd(151) | Register bit AddressCheckInfo[33] is always 0, optimizing ...
@W:MO160 : rha_array.vhd(151) | Register bit AddressCheckInfo[38] is always 0, optimizing ...
@N:MF238 : rha_data_collector.vhd(122) | Found 5-bit incrementor, 'un7_counter_1[4:0]'
@N: : rha_to_zl_converter.vhd(295) | Found counter in view:work.RHA_TO_ZL_CONVERTER(behavioral) inst Timestamp[15:0]
@N: : rha_testvalue_generator.vhd(125) | Found counter in view:work.RHA_TESTVALUE_GENERATOR(behavioral) inst Sample_Counter[8:0]
@N: : rha_testvalue_generator.vhd(125) | Found counter in view:work.RHA_TESTVALUE_GENERATOR(behavioral) inst Channel_Counter[3:0]
@N:MO106 : rha_testvalue_generator.vhd(155) | Found ROM, 'DATAOUT_ADDRESS[3]', 16 words by 1 bits 
@N:MO106 : rha_test_modulator.vhd(160) | Found ROM, 'SYNC_PROCESS\.SPIMACHINE_STATE_26[4:0]', 26 words by 5 bits 
@W:MO160 : rha_test_modulator.vhd(79) | Register bit TESTVALUE_INTERNAL[23] is always 0, optimizing ...
@W:MO160 : rha_test_modulator.vhd(79) | Register bit TESTVALUE_INTERNAL[22] is always 0, optimizing ...
@W:MO160 : rha_test_modulator.vhd(79) | Register bit TESTVALUE_INTERNAL[21] is always 0, optimizing ...
@W:MO160 : rha_test_modulator.vhd(79) | Register bit TESTVALUE_INTERNAL[2] is always 0, optimizing ...
@N:MO106 : rha_test_modulator.vhd(160) | Found ROM, 'SYNC_PROCESS\.SPIMACHINE_STATE_26[4:0]', 26 words by 5 bits 
@W:MO160 : rha_test_modulator.vhd(79) | Register bit TESTVALUE_INTERNAL[23] is always 0, optimizing ...
@W:MO160 : rha_test_modulator.vhd(79) | Register bit TESTVALUE_INTERNAL[22] is always 0, optimizing ...
@W:MO160 : rha_test_modulator.vhd(79) | Register bit TESTVALUE_INTERNAL[2] is always 0, optimizing ...
@N:MO106 : rha_test_modulator.vhd(160) | Found ROM, 'SYNC_PROCESS\.SPIMACHINE_STATE_26[4:0]', 26 words by 5 bits 
@W:MO160 : rha_test_modulator.vhd(79) | Register bit TESTVALUE_INTERNAL[23] is always 0, optimizing ...
@W:MO160 : rha_test_modulator.vhd(79) | Register bit TESTVALUE_INTERNAL[21] is always 0, optimizing ...
@W:MO160 : rha_test_modulator.vhd(79) | Register bit TESTVALUE_INTERNAL[2] is always 0, optimizing ...
@N:MO106 : rha_test_modulator.vhd(160) | Found ROM, 'SYNC_PROCESS\.SPIMACHINE_STATE_26[4:0]', 26 words by 5 bits 
@W:MO160 : rha_test_modulator.vhd(79) | Register bit TESTVALUE_INTERNAL[23] is always 0, optimizing ...
@W:MO160 : rha_test_modulator.vhd(79) | Register bit TESTVALUE_INTERNAL[2] is always 0, optimizing ...
@N:MO106 : rha_test_modulator.vhd(160) | Found ROM, 'SYNC_PROCESS\.SPIMACHINE_STATE_26[4:0]', 26 words by 5 bits 
@W:MO160 : rha_test_modulator.vhd(79) | Register bit TESTVALUE_INTERNAL[22] is always 0, optimizing ...
@W:MO160 : rha_test_modulator.vhd(79) | Register bit TESTVALUE_INTERNAL[21] is always 0, optimizing ...
@W:MO160 : rha_test_modulator.vhd(79) | Register bit TESTVALUE_INTERNAL[2] is always 0, optimizing ...
@N:MO106 : rha_test_modulator.vhd(160) | Found ROM, 'SYNC_PROCESS\.SPIMACHINE_STATE_26[4:0]', 26 words by 5 bits 
@W:MO160 : rha_test_modulator.vhd(79) | Register bit TESTVALUE_INTERNAL[22] is always 0, optimizing ...
@W:MO160 : rha_test_modulator.vhd(79) | Register bit TESTVALUE_INTERNAL[2] is always 0, optimizing ...
@N:MO106 : rha_test_modulator.vhd(160) | Found ROM, 'SYNC_PROCESS\.SPIMACHINE_STATE_26[4:0]', 26 words by 5 bits 
@W:MO160 : rha_test_modulator.vhd(79) | Register bit TESTVALUE_INTERNAL[21] is always 0, optimizing ...
@W:MO160 : rha_test_modulator.vhd(79) | Register bit TESTVALUE_INTERNAL[2] is always 0, optimizing ...
@N:MO106 : rha_test_modulator.vhd(160) | Found ROM, 'SYNC_PROCESS\.SPIMACHINE_STATE_26[4:0]', 26 words by 5 bits 
@W:MO160 : rha_test_modulator.vhd(79) | Register bit TESTVALUE_INTERNAL[2] is always 0, optimizing ...
@W:BN132 : command_receiver.vhd(460) | Removing sequential instance Inst_CONTROL_NEXUS.Inst_COMMAND_RECEIVER.BITMASK_SAMPLE_TOP_INTERNAL[15],  because it is equivalent to instance Inst_CONTROL_NEXUS.Inst_COMMAND_RECEIVER.BITMASK_SAMPLE_INTERNAL[0]
Auto Dissolve of MyRHA_TESTVALUE_GENERATOR (inst of view:work.RHA_TESTVALUE_GENERATOR(behavioral))
Auto Dissolve of Inst_DATA_ACQUISITION_BLOCK (inst of view:work.DATA_ACQUISITION_BLOCK(behavioral))

Finished factoring (Real Time elapsed 0h:00m:02s; CPU Time elapsed 0h:00m:02s; Memory used current: 129MB peak: 137MB)

@W:BN132 : data_acquisition_block.vhd(82) | Removing sequential instance Inst_DATA_ACQUISITION_BLOCK.RHA_ADC_clk[7],  because it is equivalent to instance Inst_DATA_ACQUISITION_BLOCK.RHA_ADC_clk[6]
@W:BN132 : data_acquisition_block.vhd(82) | Removing sequential instance Inst_DATA_ACQUISITION_BLOCK.RHA_ADC_clk[6],  because it is equivalent to instance Inst_DATA_ACQUISITION_BLOCK.RHA_ADC_clk[5]
@W:BN132 : data_acquisition_block.vhd(82) | Removing sequential instance Inst_DATA_ACQUISITION_BLOCK.RHA_ADC_clk[5],  because it is equivalent to instance Inst_DATA_ACQUISITION_BLOCK.RHA_ADC_clk[4]
@W:BN132 : data_acquisition_block.vhd(82) | Removing sequential instance Inst_DATA_ACQUISITION_BLOCK.RHA_ADC_clk[4],  because it is equivalent to instance Inst_DATA_ACQUISITION_BLOCK.RHA_ADC_clk[3]
@W:BN132 : data_acquisition_block.vhd(82) | Removing sequential instance Inst_DATA_ACQUISITION_BLOCK.RHA_ADC_clk[3],  because it is equivalent to instance Inst_DATA_ACQUISITION_BLOCK.RHA_ADC_clk[2]
@W:BN132 : data_acquisition_block.vhd(82) | Removing sequential instance Inst_DATA_ACQUISITION_BLOCK.RHA_ADC_clk[2],  because it is equivalent to instance Inst_DATA_ACQUISITION_BLOCK.RHA_ADC_clk[1]
@W:BN132 : data_acquisition_block.vhd(82) | Removing sequential instance Inst_DATA_ACQUISITION_BLOCK.RHA_ADC_clk[1],  because it is equivalent to instance Inst_DATA_ACQUISITION_BLOCK.RHA_ADC_clk[0]

Finished gated-clock and generated-clock conversion (Real Time elapsed 0h:00m:02s; CPU Time elapsed 0h:00m:02s; Memory used current: 126MB peak: 137MB)


Finished generic timing optimizations - Pass 1 (Real Time elapsed 0h:00m:03s; CPU Time elapsed 0h:00m:03s; Memory used current: 136MB peak: 143MB)


Starting Early Timing Optimization (Real Time elapsed 0h:00m:03s; CPU Time elapsed 0h:00m:03s; Memory used current: 138MB peak: 143MB)


Finished Early Timing Optimization (Real Time elapsed 0h:00m:04s; CPU Time elapsed 0h:00m:04s; Memory used current: 138MB peak: 143MB)


Finished generic timing optimizations - Pass 2 (Real Time elapsed 0h:00m:04s; CPU Time elapsed 0h:00m:04s; Memory used current: 136MB peak: 143MB)


Finished preparing to map (Real Time elapsed 0h:00m:04s; CPU Time elapsed 0h:00m:04s; Memory used current: 135MB peak: 143MB)


Finished technology mapping (Real Time elapsed 0h:00m:05s; CPU Time elapsed 0h:00m:05s; Memory used current: 155MB peak: 158MB)


High Fanout Net Report
**********************

Driver Instance / Pin Name                                                                        Fanout, notes                     
------------------------------------------------------------------------------------------------------------------------------------
Inst_DATA_ACQUISITION_BLOCK.Inst_RHA_ARRAY.DATAOUT_VALID_OUT / Q                                  28                                
Inst_DATA_ACQUISITION_BLOCK.Inst_RHA_TO_ZL_CONVERTER.SPIMACHINE_STATE[0] / Q                      107                               
Inst_DATA_ACQUISITION_BLOCK.Inst_RHA_TO_ZL_CONVERTER.SPIMACHINE_STATE[2] / Q                      71                                
Inst_DATA_ACQUISITION_BLOCK.Inst_RHA_TO_ZL_CONVERTER.SPIMACHINE_STATE[4] / Q                      68                                
Inst_CONTROL_NEXUS.Inst_ZARLINK_CONNECT_INIT.GetCommand_DATA_INTERNAL[0] / Q                      30                                
Inst_CONTROL_NEXUS.Inst_ZARLINK_CONNECT_INIT.GetCommand_DATA_INTERNAL[1] / Q                      33                                
Inst_CONTROL_NEXUS.Inst_ZARLINK_CONNECT_INIT.GetCommand_DATA_INTERNAL[3] / Q                      28                                
Inst_CONTROL_NEXUS.Inst_ZARLINK_CONNECT_INIT.GetCommand_DATA_INTERNAL[4] / Q                      29                                
Inst_CONTROL_NEXUS.Inst_ZARLINK_CONNECT_INIT.FSM_STATE[0] / Q                                     25                                
Inst_CONTROL_NEXUS.Inst_ZARLINK_CONNECT_INIT.FSM_STATE[1] / Q                                     29                                
Inst_CONTROL_NEXUS.Inst_ZARLINK_CONNECT_INIT.FSM_STATE[2] / Q                                     27                                
Inst_CONTROL_NEXUS.Inst_ZARLINK_CONNECT_INIT.FSM_STATE[3] / Q                                     26                                
Inst_CONTROL_NEXUS.Inst_ZARLINK_CONNECT_INIT.FSM_STATE[5] / Q                                     26                                
Inst_CONTROL_NEXUS.Inst_Zarlink_SPI_Module.FSM_STATE[0] / Q                                       25                                
ResetRHAandFIFO_DELAYED / Q                                                                       1098 : 1044 asynchronous set/reset
Reset_pad / Y                                                                                     442 : 439 asynchronous set/reset  
Inst_CONTROL_NEXUS.Inst_COMMAND_RECEIVER.CMD_RECEIVER.un1_fifo_out_valid / Y                      59                                
Inst_CONTROL_NEXUS.Inst_COMMAND_RECEIVER.un1_SPIMACHINE_STATE_i_a2 / Y                            139                               
Inst_DATA_ACQUISITION_BLOCK.Inst_RHA_ARRAY.COPY_VALID_DATA.un48_gated_valid / Y                   399                               
Inst_DATA_ACQUISITION_BLOCK.Inst_RHA_ARRAY.COPY_VALID_DATA.un52_gated_valid / Y                   198                               
Inst_DATA_ACQUISITION_BLOCK.Inst_RHA_TO_ZL_CONVERTER.un1_NewBlock_Off_1_sqmuxa / Y                48                                
Inst_CONTROL_NEXUS.Inst_ZARLINK_CONNECT_INIT.Zarlink_ResetCounter_n0_0_a4_i_o2_0_o3 / Y           26                                
Inst_CONTROL_NEXUS.Inst_ZARLINK_CONNECT_INIT.FSM.fsm_state155_i_i_a2_i / Y                        32                                
Inst_DATA_ACQUISITION_BLOCK.Inst_RHA_TESTMODULE.RHA_TEST_MOD_7.ADC_OUT_PROCESS.adc_out127 / Y     25                                
Inst_DATA_ACQUISITION_BLOCK.Inst_RHA_ARRAY.COPY_m6_0 / Y                                          209                               
====================================================================================================================================

@N:FP130 :  | Promoting Net Clk_c on CLKBUF  Clk_pad  
@N:FP130 :  | Promoting Net Reset_c on CLKBUF  Reset_pad  
@N:FP130 :  | Promoting Net ResetRHAandFIFO_DELAYED_c_c_c_c_c_c_c_c on CLKINT  I_280  
@N:FP130 :  | Promoting Net Inst_DATA_ACQUISITION_BLOCK.Inst_RHA_ARRAY.COPY_VALID_DATA\.un48_gated_valid on CLKINT  I_281  
@N:FP130 :  | Promoting Net Inst_DATA_ACQUISITION_BLOCK.RHA_ADC_clk_TEST on CLKINT  Inst_DATA_ACQUISITION_BLOCK.RHA_ADC_clk_TEST_inferred_clock  
@N:FP130 :  | Promoting Net Clk_Reduced on CLKINT  I_282  

Finished technology timing optimizations and critical path resynthesis (Real Time elapsed 0h:00m:05s; CPU Time elapsed 0h:00m:05s; Memory used current: 157MB peak: 161MB)

Replicating Combinational Instance Inst_DATA_ACQUISITION_BLOCK.Inst_RHA_ARRAY.COPY_m6_0, fanout 209 segments 9
Replicating Combinational Instance Inst_DATA_ACQUISITION_BLOCK.Inst_RHA_TESTMODULE.RHA_TEST_MOD_7.ADC_OUT_PROCESS.adc_out127, fanout 25 segments 2
Replicating Combinational Instance Inst_CONTROL_NEXUS.Inst_ZARLINK_CONNECT_INIT.FSM.fsm_state155_i_i_a2_i, fanout 32 segments 2
Replicating Combinational Instance Inst_CONTROL_NEXUS.Inst_ZARLINK_CONNECT_INIT.Zarlink_ResetCounter_n0_0_a4_i_o2_0_o3, fanout 26 segments 2
Replicating Combinational Instance Inst_DATA_ACQUISITION_BLOCK.Inst_RHA_TO_ZL_CONVERTER.un1_NewBlock_Off_1_sqmuxa, fanout 48 segments 2
Replicating Combinational Instance Inst_DATA_ACQUISITION_BLOCK.Inst_RHA_ARRAY.COPY_VALID_DATA.un52_gated_valid, fanout 198 segments 9
Replicating Combinational Instance Inst_CONTROL_NEXUS.Inst_COMMAND_RECEIVER.un1_SPIMACHINE_STATE_i_a2, fanout 139 segments 6
Replicating Combinational Instance Inst_CONTROL_NEXUS.Inst_COMMAND_RECEIVER.CMD_RECEIVER.un1_fifo_out_valid, fanout 59 segments 3
Replicating Sequential Instance Inst_CONTROL_NEXUS.Inst_Zarlink_SPI_Module.FSM_STATE[0], fanout 25 segments 2
Replicating Sequential Instance Inst_CONTROL_NEXUS.Inst_ZARLINK_CONNECT_INIT.FSM_STATE[5], fanout 26 segments 2
Replicating Sequential Instance Inst_CONTROL_NEXUS.Inst_ZARLINK_CONNECT_INIT.FSM_STATE[3], fanout 26 segments 2
Replicating Sequential Instance Inst_CONTROL_NEXUS.Inst_ZARLINK_CONNECT_INIT.FSM_STATE[2], fanout 27 segments 2
Replicating Sequential Instance Inst_CONTROL_NEXUS.Inst_ZARLINK_CONNECT_INIT.FSM_STATE[1], fanout 30 segments 2
Replicating Sequential Instance Inst_CONTROL_NEXUS.Inst_ZARLINK_CONNECT_INIT.FSM_STATE[0], fanout 27 segments 2
Replicating Sequential Instance Inst_CONTROL_NEXUS.Inst_ZARLINK_CONNECT_INIT.GetCommand_DATA_INTERNAL[4], fanout 29 segments 2
Replicating Sequential Instance Inst_CONTROL_NEXUS.Inst_ZARLINK_CONNECT_INIT.GetCommand_DATA_INTERNAL[3], fanout 28 segments 2
Replicating Sequential Instance Inst_CONTROL_NEXUS.Inst_ZARLINK_CONNECT_INIT.GetCommand_DATA_INTERNAL[1], fanout 33 segments 2
Replicating Sequential Instance Inst_CONTROL_NEXUS.Inst_ZARLINK_CONNECT_INIT.GetCommand_DATA_INTERNAL[0], fanout 30 segments 2
Replicating Sequential Instance Inst_DATA_ACQUISITION_BLOCK.Inst_RHA_TO_ZL_CONVERTER.SPIMACHINE_STATE[4], fanout 68 segments 3
Replicating Sequential Instance Inst_DATA_ACQUISITION_BLOCK.Inst_RHA_TO_ZL_CONVERTER.SPIMACHINE_STATE[2], fanout 71 segments 3
Replicating Sequential Instance Inst_DATA_ACQUISITION_BLOCK.Inst_RHA_TO_ZL_CONVERTER.SPIMACHINE_STATE[0], fanout 107 segments 5
Replicating Sequential Instance Inst_DATA_ACQUISITION_BLOCK.Inst_RHA_ARRAY.DATAOUT_VALID_OUT, fanout 28 segments 2

Added 0 Buffers
Added 46 Cells via replication
	Added 19 Sequential Cells via replication
	Added 27 Combinational Cells via replication

Finished restoring hierarchy (Real Time elapsed 0h:00m:09s; CPU Time elapsed 0h:00m:09s; Memory used current: 158MB peak: 161MB)


#### START OF CLOCK OPTIMIZATION REPORT #####[

Clock optimization not enabled
1 non-gated/non-generated clock tree(s) driving 983 clock pin(s) of sequential element(s)
5 gated/generated clock tree(s) driving 604 clock pin(s) of sequential element(s)
0 instances converted, 604 sequential instances remain driven by gated/generated clocks

============================== Non-Gated/Non-Generated Clocks ===============================
Clock Tree ID     Driving Element     Drive Element Type     Fanout     Sample Instance      
---------------------------------------------------------------------------------------------
ClockId0006        Clk                 port                   983        FIFO_IN_READENABLE_DX
=============================================================================================
=================================================================================================================================== Gated/Generated Clocks ===================================================================================================================================
Clock Tree ID     Driving Element                                                            Drive Element Type     Fanout     Sample Instance                                                                        Explanation                                                             
----------------------------------------------------------------------------------------------------------------------------------------------------------------------------------------------------------------------------------------------------------------------------------------------
ClockId0001        Inst_CONTROL_NEXUS.Inst_COMMAND_RECEIVER.Clk_Reduced_Internal_RNI48LS3     AO1                    272        Inst_DATA_ACQUISITION_BLOCK.Inst_RHA_ARRAY.RHA7.Counter[4]                             Clock conversion disabled                                               
ClockId0002        Inst_DATA_ACQUISITION_BLOCK.RHA_ADC_clk_TEST                               DLN1                   259        Inst_DATA_ACQUISITION_BLOCK.Inst_RHA_TESTMODULE.RHA_TEST_MOD_7.SPIMACHINE_STATE[4]     No generated or derived clock directive on output of sequential instance
ClockId0003        Inst_CONTROL_NEXUS.Inst_COMMAND_RECEIVER.Clk_4MHz                          DFN1C1                 51         Inst_CONTROL_NEXUS.Inst_COMMAND_RECEIVER.Counter[7]                                    No generated or derived clock directive on output of sequential instance
ClockId0004        Inst_CONTROL_NEXUS.Inst_Zarlink_SPI_Module.SPI_CLK                         DFN1C1                 20         DEBUG_ZARLINK_VALID_INTERNAL                                                           No generated or derived clock directive on output of sequential instance
ClockId0005        Inst_CONTROL_NEXUS.Inst_COMMAND_RECEIVER.TestMode_INTERAL                  DFN1E1P1               2          Inst_DATA_ACQUISITION_BLOCK.RHA_ADC_clk[0]                                             No generated or derived clock directive on output of sequential instance
==============================================================================================================================================================================================================================================================================================


##### END OF CLOCK OPTIMIZATION REPORT ######]

Writing Analyst data base C:\Users\ITP\Desktop\IGLOO\IGLOO_RHA\synthesis\IMPLANT_TOP.srm

Finished Writing Netlist Databases (Real Time elapsed 0h:00m:10s; CPU Time elapsed 0h:00m:10s; Memory used current: 154MB peak: 161MB)

Writing EDIF Netlist and constraint files
I-2013.09M-SP1 

Finished Writing EDIF Netlist and constraint files (Real Time elapsed 0h:00m:11s; CPU Time elapsed 0h:00m:10s; Memory used current: 156MB peak: 161MB)

@W:MT420 :  | Found inferred clock IMPLANT_TOP|Clk with period 10.00ns. Please declare a user-defined clock on object "p:Clk" 

@W:MT420 :  | Found inferred clock DATA_ACQUISITION_BLOCK|RHA_ADC_clk_TEST_inferred_clock with period 10.00ns. Please declare a user-defined clock on object "n:Inst_DATA_ACQUISITION_BLOCK.RHA_ADC_clk_TEST" 

@W:MT420 :  | Found inferred clock COMMAND_RECEIVER|Clk_4MHz_inferred_clock with period 10.00ns. Please declare a user-defined clock on object "n:Inst_CONTROL_NEXUS.Inst_COMMAND_RECEIVER.Clk_4MHz" 

@W:MT420 :  | Found inferred clock COMMAND_RECEIVER|TestMode_INTERAL_inferred_clock with period 10.00ns. Please declare a user-defined clock on object "n:Inst_CONTROL_NEXUS.Inst_COMMAND_RECEIVER.TestMode_INTERAL" 

@W:MT420 :  | Found inferred clock Zarlink_SPI_Module|SPI_CLK_inferred_clock with period 10.00ns. Please declare a user-defined clock on object "n:Inst_CONTROL_NEXUS.Inst_Zarlink_SPI_Module.SPI_CLK" 


##### START OF TIMING REPORT #####[
# Timing Report written on Fri Aug 01 16:17:13 2014
#


Top view:               IMPLANT_TOP
Library name:           IGLOO_V2
Operating conditions:   COMWCSTD ( T = 70.0, V = 1.14, P = 3.70, tree_type = balanced_tree )
Requested Frequency:    100.0 MHz
Wire load mode:         top
Wire load model:        igloo
Paths requested:        5
Constraint File(s):    
@N:MT320 :  | Timing report estimates place and route data. Please look at the place and route timing report for final timing. 

@N:MT322 :  | Clock constraints cover only FF-to-FF paths associated with the clock. 


Performance Summary 
*******************


Worst slack in design: -33.091

                                                           Requested     Estimated     Requested     Estimated                 Clock        Clock              
Starting Clock                                             Frequency     Frequency     Period        Period        Slack       Type         Group              
---------------------------------------------------------------------------------------------------------------------------------------------------------------
COMMAND_RECEIVER|Clk_4MHz_inferred_clock                   100.0 MHz     32.3 MHz      10.000        30.980        -17.906     inferred     Inferred_clkgroup_4
COMMAND_RECEIVER|TestMode_INTERAL_inferred_clock           100.0 MHz     NA            10.000        NA            NA          inferred     Inferred_clkgroup_2
DATA_ACQUISITION_BLOCK|RHA_ADC_clk_TEST_inferred_clock     100.0 MHz     20.2 MHz      10.000        49.565        -19.782     inferred     Inferred_clkgroup_3
IMPLANT_TOP|Clk                                            100.0 MHz     23.2 MHz      10.000        43.091        -33.091     inferred     Inferred_clkgroup_0
Zarlink_SPI_Module|SPI_CLK_inferred_clock                  100.0 MHz     111.7 MHz     10.000        8.950         1.050       inferred     Inferred_clkgroup_1
===============================================================================================================================================================


Clock Relationships
*******************

Clocks                                                                                                          |    rise  to  rise     |    fall  to  fall     |    rise  to  fall     |    fall  to  rise   
--------------------------------------------------------------------------------------------------------------------------------------------------------------------------------------------------------------
Starting                                                Ending                                                  |  constraint  slack    |  constraint  slack    |  constraint  slack    |  constraint  slack  
--------------------------------------------------------------------------------------------------------------------------------------------------------------------------------------------------------------
IMPLANT_TOP|Clk                                         IMPLANT_TOP|Clk                                         |  10.000      -33.091  |  No paths    -        |  No paths    -        |  No paths    -      
IMPLANT_TOP|Clk                                         COMMAND_RECEIVER|TestMode_INTERAL_inferred_clock        |  Diff grp    -        |  No paths    -        |  Diff grp    -        |  No paths    -      
IMPLANT_TOP|Clk                                         DATA_ACQUISITION_BLOCK|RHA_ADC_clk_TEST_inferred_clock  |  No paths    -        |  No paths    -        |  Diff grp    -        |  No paths    -      
IMPLANT_TOP|Clk                                         COMMAND_RECEIVER|Clk_4MHz_inferred_clock                |  Diff grp    -        |  No paths    -        |  Diff grp    -        |  No paths    -      
Zarlink_SPI_Module|SPI_CLK_inferred_clock               Zarlink_SPI_Module|SPI_CLK_inferred_clock               |  10.000      1.050    |  No paths    -        |  No paths    -        |  No paths    -      
DATA_ACQUISITION_BLOCK|RHA_ADC_clk_TEST_inferred_clock  DATA_ACQUISITION_BLOCK|RHA_ADC_clk_TEST_inferred_clock  |  10.000      -14.544  |  10.000      -12.544  |  5.000       -3.849   |  5.000       -19.782
DATA_ACQUISITION_BLOCK|RHA_ADC_clk_TEST_inferred_clock  COMMAND_RECEIVER|Clk_4MHz_inferred_clock                |  No paths    -        |  No paths    -        |  Diff grp    -        |  Diff grp    -      
COMMAND_RECEIVER|Clk_4MHz_inferred_clock                IMPLANT_TOP|Clk                                         |  Diff grp    -        |  No paths    -        |  No paths    -        |  Diff grp    -      
COMMAND_RECEIVER|Clk_4MHz_inferred_clock                Zarlink_SPI_Module|SPI_CLK_inferred_clock               |  Diff grp    -        |  No paths    -        |  No paths    -        |  No paths    -      
COMMAND_RECEIVER|Clk_4MHz_inferred_clock                COMMAND_RECEIVER|TestMode_INTERAL_inferred_clock        |  Diff grp    -        |  No paths    -        |  Diff grp    -        |  No paths    -      
COMMAND_RECEIVER|Clk_4MHz_inferred_clock                COMMAND_RECEIVER|Clk_4MHz_inferred_clock                |  10.000      -17.906  |  10.000      5.790    |  5.000       -10.490  |  No paths    -      
==============================================================================================================================================================================================================
 Note: 'No paths' indicates there are no paths in the design for that pair of clock edges.
       'Diff grp' indicates that paths exist but the starting clock and ending clock are in different clock groups.


Interface Information 
*********************

No IO constraint found


====================================
Detailed Report for Clock: COMMAND_RECEIVER|Clk_4MHz_inferred_clock
====================================


Starting Points with Worst Slack
********************************

                                                               Starting                                                                           Arrival            
Instance                                                       Reference                                    Type       Pin     Net                Time        Slack  
                                                               Clock                                                                                                 
---------------------------------------------------------------------------------------------------------------------------------------------------------------------
Inst_CONTROL_NEXUS.Inst_Zarlink_SPI_Module.FSM_STATE[4]        COMMAND_RECEIVER|Clk_4MHz_inferred_clock     DFN1C1     Q       FSM_STATE[4]       1.771       -17.906
Inst_CONTROL_NEXUS.Inst_Zarlink_SPI_Module.FSM_STATE[2]        COMMAND_RECEIVER|Clk_4MHz_inferred_clock     DFN1C1     Q       FSM_STATE[2]       1.395       -16.811
Inst_CONTROL_NEXUS.Inst_Zarlink_SPI_Module.FSM_STATE[5]        COMMAND_RECEIVER|Clk_4MHz_inferred_clock     DFN1C1     Q       FSM_STATE[5]       1.771       -16.594
Inst_CONTROL_NEXUS.Inst_Zarlink_SPI_Module.FSM_STATE[3]        COMMAND_RECEIVER|Clk_4MHz_inferred_clock     DFN1C1     Q       FSM_STATE[3]       1.771       -15.759
Inst_CONTROL_NEXUS.Inst_Zarlink_SPI_Module.FSM_STATE[0]        COMMAND_RECEIVER|Clk_4MHz_inferred_clock     DFN1C1     Q       FSM_STATE[0]       1.771       -15.730
Inst_CONTROL_NEXUS.Inst_Zarlink_SPI_Module.FSM_STATE[1]        COMMAND_RECEIVER|Clk_4MHz_inferred_clock     DFN1C1     Q       FSM_STATE[1]       1.771       -14.394
Inst_CONTROL_NEXUS.Inst_Zarlink_SPI_Module.FSM_STATE_0[0]      COMMAND_RECEIVER|Clk_4MHz_inferred_clock     DFN1C1     Q       FSM_STATE_0[0]     1.771       -11.549
Inst_DATA_ACQUISITION_BLOCK.Inst_RHA_ARRAY.RHA6.Counter[1]     COMMAND_RECEIVER|Clk_4MHz_inferred_clock     DFN1C1     Q       Counter[1]         1.771       -10.490
Inst_DATA_ACQUISITION_BLOCK.Inst_RHA_ARRAY.RHA7.Counter[1]     COMMAND_RECEIVER|Clk_4MHz_inferred_clock     DFN1C1     Q       Counter[1]         1.771       -10.490
Inst_DATA_ACQUISITION_BLOCK.Inst_RHA_ARRAY.RHA5.Counter[1]     COMMAND_RECEIVER|Clk_4MHz_inferred_clock     DFN1C1     Q       Counter[1]         1.771       -10.490
=====================================================================================================================================================================


Ending Points with Worst Slack
******************************

                                                                         Starting                                                                               Required            
Instance                                                                 Reference                                    Type         Pin     Net                  Time         Slack  
                                                                         Clock                                                                                                      
------------------------------------------------------------------------------------------------------------------------------------------------------------------------------------
Inst_CONTROL_NEXUS.Inst_Zarlink_SPI_Module.SPI_SDI                       COMMAND_RECEIVER|Clk_4MHz_inferred_clock     DFN1C1       D       SPI_SDI_18           8.622        -17.906
Inst_CONTROL_NEXUS.Inst_Zarlink_SPI_Module.FSM_STATE[2]                  COMMAND_RECEIVER|Clk_4MHz_inferred_clock     DFN1C1       D       FSM_STATE_ns[2]      8.622        -15.279
Inst_CONTROL_NEXUS.Inst_Zarlink_SPI_Module.FSM_STATE[3]                  COMMAND_RECEIVER|Clk_4MHz_inferred_clock     DFN1C1       D       FSM_STATE_RNO[3]     8.705        -14.686
Inst_CONTROL_NEXUS.Inst_Zarlink_SPI_Module.FSM_STATE[0]                  COMMAND_RECEIVER|Clk_4MHz_inferred_clock     DFN1C1       D       FSM_STATE_ns[0]      8.705        -13.608
Inst_CONTROL_NEXUS.Inst_Zarlink_SPI_Module.FSM_STATE_0[0]                COMMAND_RECEIVER|Clk_4MHz_inferred_clock     DFN1C1       D       FSM_STATE_ns[0]      8.705        -13.608
Inst_CONTROL_NEXUS.Inst_Zarlink_SPI_Module.FSM_STATE[4]                  COMMAND_RECEIVER|Clk_4MHz_inferred_clock     DFN1C1       D       FSM_STATE_ns[4]      8.705        -13.279
Inst_CONTROL_NEXUS.Inst_Zarlink_SPI_Module.Zarlink_Data_In_Buffer[1]     COMMAND_RECEIVER|Clk_4MHz_inferred_clock     DFN1E1C1     D       N_8                  8.705        -12.381
Inst_CONTROL_NEXUS.Inst_Zarlink_SPI_Module.Zarlink_Data_In_Buffer[2]     COMMAND_RECEIVER|Clk_4MHz_inferred_clock     DFN1E1C1     D       N_10                 8.705        -12.381
Inst_CONTROL_NEXUS.Inst_Zarlink_SPI_Module.Zarlink_Data_In_Buffer[3]     COMMAND_RECEIVER|Clk_4MHz_inferred_clock     DFN1E1C1     D       N_12                 8.705        -12.381
Inst_CONTROL_NEXUS.Inst_Zarlink_SPI_Module.Zarlink_Data_In_Buffer[4]     COMMAND_RECEIVER|Clk_4MHz_inferred_clock     DFN1E1C1     D       N_14                 8.705        -12.381
====================================================================================================================================================================================


Worst Path Information
View Worst Path in Analyst
***********************


Path information for path number 1: 
      Requested Period:                      10.000
    - Setup time:                            1.378
    + Clock delay at ending point:           0.000 (ideal)
    = Required time:                         8.622

    - Propagation time:                      26.527
    - Clock delay at starting point:         0.000 (ideal)
    = Slack (non-critical) :                 -17.906

    Number of logic level(s):                7
    Starting point:                          Inst_CONTROL_NEXUS.Inst_Zarlink_SPI_Module.FSM_STATE[4] / Q
    Ending point:                            Inst_CONTROL_NEXUS.Inst_Zarlink_SPI_Module.SPI_SDI / D
    The start point is clocked by            COMMAND_RECEIVER|Clk_4MHz_inferred_clock [rising] on pin CLK
    The end   point is clocked by            COMMAND_RECEIVER|Clk_4MHz_inferred_clock [rising] on pin CLK

Instance / Net                                                                   Pin      Pin               Arrival     No. of    
Name                                                                  Type       Name     Dir     Delay     Time        Fan Out(s)
----------------------------------------------------------------------------------------------------------------------------------
Inst_CONTROL_NEXUS.Inst_Zarlink_SPI_Module.FSM_STATE[4]               DFN1C1     Q        Out     1.771     1.771       -         
FSM_STATE[4]                                                          Net        -        -       5.926     -           24        
Inst_CONTROL_NEXUS.Inst_Zarlink_SPI_Module.FSM_STATE_RNIAKJC_1[2]     NOR2A      A        In      -         7.697       -         
Inst_CONTROL_NEXUS.Inst_Zarlink_SPI_Module.FSM_STATE_RNIAKJC_1[2]     NOR2A      Y        Out     1.508     9.204       -         
N_216                                                                 Net        -        -       3.420     -           6         
Inst_CONTROL_NEXUS.Inst_Zarlink_SPI_Module.SPI_SDI_RNO_35             NOR2B      B        In      -         12.625      -         
Inst_CONTROL_NEXUS.Inst_Zarlink_SPI_Module.SPI_SDI_RNO_35             NOR2B      Y        Out     1.508     14.133      -         
SPI_SDI_18_iv_0_0_a4_1_0                                              Net        -        -       0.773     -           1         
Inst_CONTROL_NEXUS.Inst_Zarlink_SPI_Module.SPI_SDI_RNO_30             NOR3B      B        In      -         14.905      -         
Inst_CONTROL_NEXUS.Inst_Zarlink_SPI_Module.SPI_SDI_RNO_30             NOR3B      Y        Out     1.458     16.363      -         
N_582                                                                 Net        -        -       0.773     -           1         
Inst_CONTROL_NEXUS.Inst_Zarlink_SPI_Module.SPI_SDI_RNO_17             AO1A       C        In      -         17.135      -         
Inst_CONTROL_NEXUS.Inst_Zarlink_SPI_Module.SPI_SDI_RNO_17             AO1A       Y        Out     1.520     18.655      -         
SPI_SDI_18_iv_0_0_4                                                   Net        -        -       0.773     -           1         
Inst_CONTROL_NEXUS.Inst_Zarlink_SPI_Module.SPI_SDI_RNO_5              OR3        C        In      -         19.428      -         
Inst_CONTROL_NEXUS.Inst_Zarlink_SPI_Module.SPI_SDI_RNO_5              OR3        Y        Out     1.804     21.232      -         
SPI_SDI_18_iv_0_0_6                                                   Net        -        -       0.773     -           1         
Inst_CONTROL_NEXUS.Inst_Zarlink_SPI_Module.SPI_SDI_RNO_0              OR3        C        In      -         22.005      -         
Inst_CONTROL_NEXUS.Inst_Zarlink_SPI_Module.SPI_SDI_RNO_0              OR3        Y        Out     1.804     23.809      -         
SPI_SDI_18_iv_0_0_11                                                  Net        -        -       0.773     -           1         
Inst_CONTROL_NEXUS.Inst_Zarlink_SPI_Module.SPI_SDI_RNO                OR3        A        In      -         24.581      -         
Inst_CONTROL_NEXUS.Inst_Zarlink_SPI_Module.SPI_SDI_RNO                OR3        Y        Out     1.174     25.755      -         
SPI_SDI_18                                                            Net        -        -       0.773     -           1         
Inst_CONTROL_NEXUS.Inst_Zarlink_SPI_Module.SPI_SDI                    DFN1C1     D        In      -         26.527      -         
==================================================================================================================================
Total path delay (propagation time + setup) of 27.906 is 13.923(49.9%) logic and 13.982(50.1%) route.
Path delay compensated for clock skew. Clock skew is added to clock-to-out value, and is subtracted from setup time value


Path information for path number 2: 
      Requested Period:                      10.000
    - Setup time:                            1.378
    + Clock delay at ending point:           0.000 (ideal)
    = Required time:                         8.622

    - Propagation time:                      25.433
    - Clock delay at starting point:         0.000 (ideal)
    = Slack (non-critical) :                 -16.811

    Number of logic level(s):                7
    Starting point:                          Inst_CONTROL_NEXUS.Inst_Zarlink_SPI_Module.FSM_STATE[2] / Q
    Ending point:                            Inst_CONTROL_NEXUS.Inst_Zarlink_SPI_Module.SPI_SDI / D
    The start point is clocked by            COMMAND_RECEIVER|Clk_4MHz_inferred_clock [rising] on pin CLK
    The end   point is clocked by            COMMAND_RECEIVER|Clk_4MHz_inferred_clock [rising] on pin CLK

Instance / Net                                                                   Pin      Pin               Arrival     No. of    
Name                                                                  Type       Name     Dir     Delay     Time        Fan Out(s)
----------------------------------------------------------------------------------------------------------------------------------
Inst_CONTROL_NEXUS.Inst_Zarlink_SPI_Module.FSM_STATE[2]               DFN1C1     Q        Out     1.395     1.395       -         
FSM_STATE[2]                                                          Net        -        -       5.788     -           22        
Inst_CONTROL_NEXUS.Inst_Zarlink_SPI_Module.FSM_STATE_RNIAKJC_1[2]     NOR2A      B        In      -         7.183       -         
Inst_CONTROL_NEXUS.Inst_Zarlink_SPI_Module.FSM_STATE_RNIAKJC_1[2]     NOR2A      Y        Out     0.927     8.110       -         
N_216                                                                 Net        -        -       3.420     -           6         
Inst_CONTROL_NEXUS.Inst_Zarlink_SPI_Module.SPI_SDI_RNO_35             NOR2B      B        In      -         11.531      -         
Inst_CONTROL_NEXUS.Inst_Zarlink_SPI_Module.SPI_SDI_RNO_35             NOR2B      Y        Out     1.508     13.038      -         
SPI_SDI_18_iv_0_0_a4_1_0                                              Net        -        -       0.773     -           1         
Inst_CONTROL_NEXUS.Inst_Zarlink_SPI_Module.SPI_SDI_RNO_30             NOR3B      B        In      -         13.811      -         
Inst_CONTROL_NEXUS.Inst_Zarlink_SPI_Module.SPI_SDI_RNO_30             NOR3B      Y        Out     1.458     15.269      -         
N_582                                                                 Net        -        -       0.773     -           1         
Inst_CONTROL_NEXUS.Inst_Zarlink_SPI_Module.SPI_SDI_RNO_17             AO1A       C        In      -         16.041      -         
Inst_CONTROL_NEXUS.Inst_Zarlink_SPI_Module.SPI_SDI_RNO_17             AO1A       Y        Out     1.520     17.561      -         
SPI_SDI_18_iv_0_0_4                                                   Net        -        -       0.773     -           1         
Inst_CONTROL_NEXUS.Inst_Zarlink_SPI_Module.SPI_SDI_RNO_5              OR3        C        In      -         18.334      -         
Inst_CONTROL_NEXUS.Inst_Zarlink_SPI_Module.SPI_SDI_RNO_5              OR3        Y        Out     1.804     20.138      -         
SPI_SDI_18_iv_0_0_6                                                   Net        -        -       0.773     -           1         
Inst_CONTROL_NEXUS.Inst_Zarlink_SPI_Module.SPI_SDI_RNO_0              OR3        C        In      -         20.910      -         
Inst_CONTROL_NEXUS.Inst_Zarlink_SPI_Module.SPI_SDI_RNO_0              OR3        Y        Out     1.804     22.715      -         
SPI_SDI_18_iv_0_0_11                                                  Net        -        -       0.773     -           1         
Inst_CONTROL_NEXUS.Inst_Zarlink_SPI_Module.SPI_SDI_RNO                OR3        A        In      -         23.487      -         
Inst_CONTROL_NEXUS.Inst_Zarlink_SPI_Module.SPI_SDI_RNO                OR3        Y        Out     1.174     24.661      -         
SPI_SDI_18                                                            Net        -        -       0.773     -           1         
Inst_CONTROL_NEXUS.Inst_Zarlink_SPI_Module.SPI_SDI                    DFN1C1     D        In      -         25.433      -         
==================================================================================================================================
Total path delay (propagation time + setup) of 26.811 is 12.967(48.4%) logic and 13.844(51.6%) route.
Path delay compensated for clock skew. Clock skew is added to clock-to-out value, and is subtracted from setup time value


Path information for path number 3: 
      Requested Period:                      10.000
    - Setup time:                            1.378
    + Clock delay at ending point:           0.000 (ideal)
    = Required time:                         8.622

    - Propagation time:                      25.216
    - Clock delay at starting point:         0.000 (ideal)
    = Slack (non-critical) :                 -16.594

    Number of logic level(s):                6
    Starting point:                          Inst_CONTROL_NEXUS.Inst_Zarlink_SPI_Module.FSM_STATE[5] / Q
    Ending point:                            Inst_CONTROL_NEXUS.Inst_Zarlink_SPI_Module.SPI_SDI / D
    The start point is clocked by            COMMAND_RECEIVER|Clk_4MHz_inferred_clock [rising] on pin CLK
    The end   point is clocked by            COMMAND_RECEIVER|Clk_4MHz_inferred_clock [rising] on pin CLK

Instance / Net                                                                 Pin      Pin               Arrival     No. of    
Name                                                                Type       Name     Dir     Delay     Time        Fan Out(s)
--------------------------------------------------------------------------------------------------------------------------------
Inst_CONTROL_NEXUS.Inst_Zarlink_SPI_Module.FSM_STATE[5]             DFN1C1     Q        Out     1.771     1.771       -         
FSM_STATE[5]                                                        Net        -        -       5.926     -           24        
Inst_CONTROL_NEXUS.Inst_Zarlink_SPI_Module.FSM_STATE_RNI9JJC[0]     OR2B       A        In      -         7.697       -         
Inst_CONTROL_NEXUS.Inst_Zarlink_SPI_Module.FSM_STATE_RNI9JJC[0]     OR2B       Y        Out     1.236     8.933       -         
N_58                                                                Net        -        -       3.420     -           6         
Inst_CONTROL_NEXUS.Inst_Zarlink_SPI_Module.FSM_STATE_RNICBTI[1]     OR2A       B        In      -         12.354      -         
Inst_CONTROL_NEXUS.Inst_Zarlink_SPI_Module.FSM_STATE_RNICBTI[1]     OR2A       Y        Out     1.236     13.590      -         
N_525                                                               Net        -        -       2.844     -           4         
Inst_CONTROL_NEXUS.Inst_Zarlink_SPI_Module.SPI_SDI_RNO_13           NOR3A      C        In      -         16.434      -         
Inst_CONTROL_NEXUS.Inst_Zarlink_SPI_Module.SPI_SDI_RNO_13           NOR3A      Y        Out     1.541     17.975      -         
N_596                                                               Net        -        -       0.773     -           1         
Inst_CONTROL_NEXUS.Inst_Zarlink_SPI_Module.SPI_SDI_RNO_3            OR3        C        In      -         18.747      -         
Inst_CONTROL_NEXUS.Inst_Zarlink_SPI_Module.SPI_SDI_RNO_3            OR3        Y        Out     1.804     20.552      -         
SPI_SDI_18_iv_0_0_5                                                 Net        -        -       0.773     -           1         
Inst_CONTROL_NEXUS.Inst_Zarlink_SPI_Module.SPI_SDI_RNO_0            OR3        A        In      -         21.324      -         
Inst_CONTROL_NEXUS.Inst_Zarlink_SPI_Module.SPI_SDI_RNO_0            OR3        Y        Out     1.174     22.498      -         
SPI_SDI_18_iv_0_0_11                                                Net        -        -       0.773     -           1         
Inst_CONTROL_NEXUS.Inst_Zarlink_SPI_Module.SPI_SDI_RNO              OR3        A        In      -         23.270      -         
Inst_CONTROL_NEXUS.Inst_Zarlink_SPI_Module.SPI_SDI_RNO              OR3        Y        Out     1.174     24.444      -         
SPI_SDI_18                                                          Net        -        -       0.773     -           1         
Inst_CONTROL_NEXUS.Inst_Zarlink_SPI_Module.SPI_SDI                  DFN1C1     D        In      -         25.216      -         
================================================================================================================================
Total path delay (propagation time + setup) of 26.594 is 11.313(42.5%) logic and 15.281(57.5%) route.
Path delay compensated for clock skew. Clock skew is added to clock-to-out value, and is subtracted from setup time value


Path information for path number 4: 
      Requested Period:                      10.000
    - Setup time:                            1.378
    + Clock delay at ending point:           0.000 (ideal)
    = Required time:                         8.622

    - Propagation time:                      25.095
    - Clock delay at starting point:         0.000 (ideal)
    = Slack (non-critical) :                 -16.473

    Number of logic level(s):                6
    Starting point:                          Inst_CONTROL_NEXUS.Inst_Zarlink_SPI_Module.FSM_STATE[4] / Q
    Ending point:                            Inst_CONTROL_NEXUS.Inst_Zarlink_SPI_Module.SPI_SDI / D
    The start point is clocked by            COMMAND_RECEIVER|Clk_4MHz_inferred_clock [rising] on pin CLK
    The end   point is clocked by            COMMAND_RECEIVER|Clk_4MHz_inferred_clock [rising] on pin CLK

Instance / Net                                                                   Pin      Pin               Arrival     No. of    
Name                                                                  Type       Name     Dir     Delay     Time        Fan Out(s)
----------------------------------------------------------------------------------------------------------------------------------
Inst_CONTROL_NEXUS.Inst_Zarlink_SPI_Module.FSM_STATE[4]               DFN1C1     Q        Out     1.771     1.771       -         
FSM_STATE[4]                                                          Net        -        -       5.926     -           24        
Inst_CONTROL_NEXUS.Inst_Zarlink_SPI_Module.FSM_STATE_RNIAKJC_0[2]     OR2B       B        In      -         7.697       -         
Inst_CONTROL_NEXUS.Inst_Zarlink_SPI_Module.FSM_STATE_RNIAKJC_0[2]     OR2B       Y        Out     1.508     9.204       -         
N_52                                                                  Net        -        -       4.268     -           11        
Inst_CONTROL_NEXUS.Inst_Zarlink_SPI_Module.SPI_SDI_RNO_29             NOR3A      C        In      -         13.473      -         
Inst_CONTROL_NEXUS.Inst_Zarlink_SPI_Module.SPI_SDI_RNO_29             NOR3A      Y        Out     1.541     15.014      -         
SPI_SDI_18_iv_0_0_a4_15_1                                             Net        -        -       0.773     -           1         
Inst_CONTROL_NEXUS.Inst_Zarlink_SPI_Module.SPI_SDI_RNO_17             AO1A       B        In      -         15.786      -         
Inst_CONTROL_NEXUS.Inst_Zarlink_SPI_Module.SPI_SDI_RNO_17             AO1A       Y        Out     1.437     17.223      -         
SPI_SDI_18_iv_0_0_4                                                   Net        -        -       0.773     -           1         
Inst_CONTROL_NEXUS.Inst_Zarlink_SPI_Module.SPI_SDI_RNO_5              OR3        C        In      -         17.996      -         
Inst_CONTROL_NEXUS.Inst_Zarlink_SPI_Module.SPI_SDI_RNO_5              OR3        Y        Out     1.804     19.800      -         
SPI_SDI_18_iv_0_0_6                                                   Net        -        -       0.773     -           1         
Inst_CONTROL_NEXUS.Inst_Zarlink_SPI_Module.SPI_SDI_RNO_0              OR3        C        In      -         20.572      -         
Inst_CONTROL_NEXUS.Inst_Zarlink_SPI_Module.SPI_SDI_RNO_0              OR3        Y        Out     1.804     22.376      -         
SPI_SDI_18_iv_0_0_11                                                  Net        -        -       0.773     -           1         
Inst_CONTROL_NEXUS.Inst_Zarlink_SPI_Module.SPI_SDI_RNO                OR3        A        In      -         23.149      -         
Inst_CONTROL_NEXUS.Inst_Zarlink_SPI_Module.SPI_SDI_RNO                OR3        Y        Out     1.174     24.322      -         
SPI_SDI_18                                                            Net        -        -       0.773     -           1         
Inst_CONTROL_NEXUS.Inst_Zarlink_SPI_Module.SPI_SDI                    DFN1C1     D        In      -         25.095      -         
==================================================================================================================================
Total path delay (propagation time + setup) of 26.473 is 12.416(46.9%) logic and 14.057(53.1%) route.
Path delay compensated for clock skew. Clock skew is added to clock-to-out value, and is subtracted from setup time value


Path information for path number 5: 
      Requested Period:                      10.000
    - Setup time:                            1.378
    + Clock delay at ending point:           0.000 (ideal)
    = Required time:                         8.622

    - Propagation time:                      25.024
    - Clock delay at starting point:         0.000 (ideal)
    = Slack (non-critical) :                 -16.402

    Number of logic level(s):                5
    Starting point:                          Inst_CONTROL_NEXUS.Inst_Zarlink_SPI_Module.FSM_STATE[4] / Q
    Ending point:                            Inst_CONTROL_NEXUS.Inst_Zarlink_SPI_Module.SPI_SDI / D
    The start point is clocked by            COMMAND_RECEIVER|Clk_4MHz_inferred_clock [rising] on pin CLK
    The end   point is clocked by            COMMAND_RECEIVER|Clk_4MHz_inferred_clock [rising] on pin CLK

Instance / Net                                                                   Pin      Pin               Arrival     No. of    
Name                                                                  Type       Name     Dir     Delay     Time        Fan Out(s)
----------------------------------------------------------------------------------------------------------------------------------
Inst_CONTROL_NEXUS.Inst_Zarlink_SPI_Module.FSM_STATE[4]               DFN1C1     Q        Out     1.771     1.771       -         
FSM_STATE[4]                                                          Net        -        -       5.926     -           24        
Inst_CONTROL_NEXUS.Inst_Zarlink_SPI_Module.FSM_STATE_RNIAKJC_1[2]     NOR2A      A        In      -         7.697       -         
Inst_CONTROL_NEXUS.Inst_Zarlink_SPI_Module.FSM_STATE_RNIAKJC_1[2]     NOR2A      Y        Out     1.508     9.204       -         
N_216                                                                 Net        -        -       3.420     -           6         
Inst_CONTROL_NEXUS.Inst_Zarlink_SPI_Module.FSM_STATE_RNIMA7P_0[2]     NOR2A      A        In      -         12.625      -         
Inst_CONTROL_NEXUS.Inst_Zarlink_SPI_Module.FSM_STATE_RNIMA7P_0[2]     NOR2A      Y        Out     1.508     14.133      -         
N_595_2                                                               Net        -        -       3.938     -           8         
Inst_CONTROL_NEXUS.Inst_Zarlink_SPI_Module.SPI_SDI_RNO_9              NOR3C      A        In      -         18.071      -         
Inst_CONTROL_NEXUS.Inst_Zarlink_SPI_Module.SPI_SDI_RNO_9              NOR3C      Y        Out     1.115     19.186      -         
N_595                                                                 Net        -        -       0.773     -           1         
Inst_CONTROL_NEXUS.Inst_Zarlink_SPI_Module.SPI_SDI_RNO_2              OR3        B        In      -         19.958      -         
Inst_CONTROL_NEXUS.Inst_Zarlink_SPI_Module.SPI_SDI_RNO_2              OR3        Y        Out     1.716     21.675      -         
SPI_SDI_18_iv_0_0_12                                                  Net        -        -       0.773     -           1         
Inst_CONTROL_NEXUS.Inst_Zarlink_SPI_Module.SPI_SDI_RNO                OR3        C        In      -         22.447      -         
Inst_CONTROL_NEXUS.Inst_Zarlink_SPI_Module.SPI_SDI_RNO                OR3        Y        Out     1.804     24.252      -         
SPI_SDI_18                                                            Net        -        -       0.773     -           1         
Inst_CONTROL_NEXUS.Inst_Zarlink_SPI_Module.SPI_SDI                    DFN1C1     D        In      -         25.024      -         
==================================================================================================================================
Total path delay (propagation time + setup) of 26.402 is 10.800(40.9%) logic and 15.603(59.1%) route.
Path delay compensated for clock skew. Clock skew is added to clock-to-out value, and is subtracted from setup time value


====================================
Detailed Report for Clock: DATA_ACQUISITION_BLOCK|RHA_ADC_clk_TEST_inferred_clock
====================================


Starting Points with Worst Slack
********************************

                                                                                       Starting                                                                                              Arrival            
Instance                                                                               Reference                                                  Type       Pin     Net                     Time        Slack  
                                                                                       Clock                                                                                                                    
----------------------------------------------------------------------------------------------------------------------------------------------------------------------------------------------------------------
Inst_DATA_ACQUISITION_BLOCK.Inst_RHA_TESTMODULE.RHA_TEST_MOD_1.SPIMACHINE_STATE[2]     DATA_ACQUISITION_BLOCK|RHA_ADC_clk_TEST_inferred_clock     DFN0E0     Q       SPIMACHINE_STATE[2]     1.570       -19.782
Inst_DATA_ACQUISITION_BLOCK.Inst_RHA_TESTMODULE.RHA_TEST_MOD_1.SPIMACHINE_STATE[1]     DATA_ACQUISITION_BLOCK|RHA_ADC_clk_TEST_inferred_clock     DFN0E0     Q       SPIMACHINE_STATE[1]     1.570       -19.707
Inst_DATA_ACQUISITION_BLOCK.Inst_RHA_TESTMODULE.RHA_TEST_MOD_3.SPIMACHINE_STATE[0]     DATA_ACQUISITION_BLOCK|RHA_ADC_clk_TEST_inferred_clock     DFN0E0     Q       SPIMACHINE_STATE[0]     1.570       -17.423
Inst_DATA_ACQUISITION_BLOCK.Inst_RHA_TESTMODULE.RHA_TEST_MOD_4.SPIMACHINE_STATE[2]     DATA_ACQUISITION_BLOCK|RHA_ADC_clk_TEST_inferred_clock     DFN0E0     Q       SPIMACHINE_STATE[2]     1.570       -17.364
Inst_DATA_ACQUISITION_BLOCK.Inst_RHA_TESTMODULE.RHA_TEST_MOD_4.SPIMACHINE_STATE[1]     DATA_ACQUISITION_BLOCK|RHA_ADC_clk_TEST_inferred_clock     DFN0E0     Q       SPIMACHINE_STATE[1]     1.570       -17.289
Inst_DATA_ACQUISITION_BLOCK.Inst_RHA_TESTMODULE.RHA_TEST_MOD_3.SPIMACHINE_STATE[3]     DATA_ACQUISITION_BLOCK|RHA_ADC_clk_TEST_inferred_clock     DFN0E0     Q       SPIMACHINE_STATE[3]     1.265       -17.222
Inst_DATA_ACQUISITION_BLOCK.Inst_RHA_TESTMODULE.RHA_TEST_MOD_0.SPIMACHINE_STATE[2]     DATA_ACQUISITION_BLOCK|RHA_ADC_clk_TEST_inferred_clock     DFN0E0     Q       SPIMACHINE_STATE[2]     1.570       -17.130
Inst_DATA_ACQUISITION_BLOCK.Inst_RHA_TESTMODULE.RHA_TEST_MOD_0.SPIMACHINE_STATE[4]     DATA_ACQUISITION_BLOCK|RHA_ADC_clk_TEST_inferred_clock     DFN0E0     Q       SPIMACHINE_STATE[4]     1.570       -17.084
Inst_DATA_ACQUISITION_BLOCK.Inst_RHA_TESTMODULE.RHA_TEST_MOD_5.SPIMACHINE_STATE[0]     DATA_ACQUISITION_BLOCK|RHA_ADC_clk_TEST_inferred_clock     DFN0E0     Q       SPIMACHINE_STATE[0]     1.570       -17.076
Inst_DATA_ACQUISITION_BLOCK.Inst_RHA_TESTMODULE.RHA_TEST_MOD_4.SPIMACHINE_STATE[4]     DATA_ACQUISITION_BLOCK|RHA_ADC_clk_TEST_inferred_clock     DFN0E0     Q       SPIMACHINE_STATE[4]     1.570       -17.043
================================================================================================================================================================================================================


Ending Points with Worst Slack
******************************

                                                                                          Starting                                                                                                        Required            
Instance                                                                                  Reference                                                  Type         Pin     Net                             Time         Slack  
                                                                                          Clock                                                                                                                               
------------------------------------------------------------------------------------------------------------------------------------------------------------------------------------------------------------------------------
Inst_DATA_ACQUISITION_BLOCK.Inst_RHA_TESTMODULE.RHA_TEST_MOD_1.TESTVALUE_INTERNAL[0]      DATA_ACQUISITION_BLOCK|RHA_ADC_clk_TEST_inferred_clock     DFN1C1       D       TESTVALUE_INTERNAL_RNO_0[0]     3.622        -19.782
Inst_DATA_ACQUISITION_BLOCK.Inst_RHA_TESTMODULE.RHA_TEST_MOD_1.TESTVALUE_INTERNAL[1]      DATA_ACQUISITION_BLOCK|RHA_ADC_clk_TEST_inferred_clock     DFN1C1       D       TESTVALUE_INTERNAL_RNO_0[1]     3.622        -19.782
Inst_DATA_ACQUISITION_BLOCK.Inst_RHA_TESTMODULE.RHA_TEST_MOD_1.TESTVALUE_INTERNAL[21]     DATA_ACQUISITION_BLOCK|RHA_ADC_clk_TEST_inferred_clock     DFN1C1       D       TESTVALUE_INTERNAL_RNO[21]      3.622        -19.782
Inst_DATA_ACQUISITION_BLOCK.Inst_RHA_TESTMODULE.RHA_TEST_MOD_1.LastChannel_Internal       DATA_ACQUISITION_BLOCK|RHA_ADC_clk_TEST_inferred_clock     DFN1E1C1     E       adc_out127                      3.538        -17.540
Inst_DATA_ACQUISITION_BLOCK.Inst_RHA_TESTMODULE.RHA_TEST_MOD_1.TESTVALUE_INTERNAL[3]      DATA_ACQUISITION_BLOCK|RHA_ADC_clk_TEST_inferred_clock     DFN1E1C1     E       adc_out127                      3.538        -17.540
Inst_DATA_ACQUISITION_BLOCK.Inst_RHA_TESTMODULE.RHA_TEST_MOD_1.TESTVALUE_INTERNAL[4]      DATA_ACQUISITION_BLOCK|RHA_ADC_clk_TEST_inferred_clock     DFN1E1C1     E       adc_out127                      3.538        -17.540
Inst_DATA_ACQUISITION_BLOCK.Inst_RHA_TESTMODULE.RHA_TEST_MOD_1.TESTVALUE_INTERNAL[5]      DATA_ACQUISITION_BLOCK|RHA_ADC_clk_TEST_inferred_clock     DFN1E1C1     E       adc_out127                      3.538        -17.540
Inst_DATA_ACQUISITION_BLOCK.Inst_RHA_TESTMODULE.RHA_TEST_MOD_1.TESTVALUE_INTERNAL[6]      DATA_ACQUISITION_BLOCK|RHA_ADC_clk_TEST_inferred_clock     DFN1E1C1     E       adc_out127                      3.538        -17.540
Inst_DATA_ACQUISITION_BLOCK.Inst_RHA_TESTMODULE.RHA_TEST_MOD_1.TESTVALUE_INTERNAL[7]      DATA_ACQUISITION_BLOCK|RHA_ADC_clk_TEST_inferred_clock     DFN1E1C1     E       adc_out127                      3.538        -17.540
Inst_DATA_ACQUISITION_BLOCK.Inst_RHA_TESTMODULE.RHA_TEST_MOD_1.TESTVALUE_INTERNAL[8]      DATA_ACQUISITION_BLOCK|RHA_ADC_clk_TEST_inferred_clock     DFN1E1C1     E       adc_out127                      3.538        -17.540
==============================================================================================================================================================================================================================


Worst Path Information
View Worst Path in Analyst
***********************


Path information for path number 1: 
      Requested Period:                      5.000
    - Setup time:                            1.378
    + Clock delay at ending point:           0.000 (ideal)
    = Required time:                         3.622

    - Propagation time:                      23.404
    - Clock delay at starting point:         0.000 (ideal)
    = Slack (non-critical) :                 -19.782

    Number of logic level(s):                4
    Starting point:                          Inst_DATA_ACQUISITION_BLOCK.Inst_RHA_TESTMODULE.RHA_TEST_MOD_1.SPIMACHINE_STATE[2] / Q
    Ending point:                            Inst_DATA_ACQUISITION_BLOCK.Inst_RHA_TESTMODULE.RHA_TEST_MOD_1.TESTVALUE_INTERNAL[0] / D
    The start point is clocked by            DATA_ACQUISITION_BLOCK|RHA_ADC_clk_TEST_inferred_clock [falling] on pin CLK
    The end   point is clocked by            DATA_ACQUISITION_BLOCK|RHA_ADC_clk_TEST_inferred_clock [rising] on pin CLK

Instance / Net                                                                                            Pin      Pin               Arrival     No. of    
Name                                                                                           Type       Name     Dir     Delay     Time        Fan Out(s)
-----------------------------------------------------------------------------------------------------------------------------------------------------------
Inst_DATA_ACQUISITION_BLOCK.Inst_RHA_TESTMODULE.RHA_TEST_MOD_1.SPIMACHINE_STATE[2]             DFN0E0     Q        Out     1.570     1.570       -         
SPIMACHINE_STATE[2]                                                                            Net        -        -       4.105     -           10        
Inst_DATA_ACQUISITION_BLOCK.Inst_RHA_TESTMODULE.RHA_TEST_MOD_1.SPIMACHINE_STATE_RNI7VEB[1]     OR2A       A        In      -         5.676       -         
Inst_DATA_ACQUISITION_BLOCK.Inst_RHA_TESTMODULE.RHA_TEST_MOD_1.SPIMACHINE_STATE_RNI7VEB[1]     OR2A       Y        Out     1.119     6.795       -         
N_42                                                                                           Net        -        -       3.420     -           6         
Inst_DATA_ACQUISITION_BLOCK.Inst_RHA_TESTMODULE.RHA_TEST_MOD_1.SPIMACHINE_STATE_RNIDH6H[4]     NOR2A      B        In      -         10.215      -         
Inst_DATA_ACQUISITION_BLOCK.Inst_RHA_TESTMODULE.RHA_TEST_MOD_1.SPIMACHINE_STATE_RNIDH6H[4]     NOR2A      Y        Out     0.927     11.142      -         
N_112                                                                                          Net        -        -       2.844     -           4         
Inst_DATA_ACQUISITION_BLOCK.Inst_RHA_TESTMODULE.RHA_TEST_MOD_1.SPIMACHINE_STATE_RNIKGLS[3]     NOR2B      A        In      -         13.986      -         
Inst_DATA_ACQUISITION_BLOCK.Inst_RHA_TESTMODULE.RHA_TEST_MOD_1.SPIMACHINE_STATE_RNIKGLS[3]     NOR2B      Y        Out     1.236     15.223      -         
adc_out127                                                                                     Net        -        -       5.855     -           23        
Inst_DATA_ACQUISITION_BLOCK.Inst_RHA_TESTMODULE.RHA_TEST_MOD_1.TESTVALUE_INTERNAL_RNO[0]       OR2        B        In      -         21.078      -         
Inst_DATA_ACQUISITION_BLOCK.Inst_RHA_TESTMODULE.RHA_TEST_MOD_1.TESTVALUE_INTERNAL_RNO[0]       OR2        Y        Out     1.554     22.632      -         
TESTVALUE_INTERNAL_RNO_0[0]                                                                    Net        -        -       0.773     -           1         
Inst_DATA_ACQUISITION_BLOCK.Inst_RHA_TESTMODULE.RHA_TEST_MOD_1.TESTVALUE_INTERNAL[0]           DFN1C1     D        In      -         23.404      -         
===========================================================================================================================================================
Total path delay (propagation time + setup) of 24.782 is 7.785(31.4%) logic and 16.998(68.6%) route.
Path delay compensated for clock skew. Clock skew is added to clock-to-out value, and is subtracted from setup time value


Path information for path number 2: 
      Requested Period:                      5.000
    - Setup time:                            1.378
    + Clock delay at ending point:           0.000 (ideal)
    = Required time:                         3.622

    - Propagation time:                      23.404
    - Clock delay at starting point:         0.000 (ideal)
    = Slack (non-critical) :                 -19.782

    Number of logic level(s):                4
    Starting point:                          Inst_DATA_ACQUISITION_BLOCK.Inst_RHA_TESTMODULE.RHA_TEST_MOD_1.SPIMACHINE_STATE[2] / Q
    Ending point:                            Inst_DATA_ACQUISITION_BLOCK.Inst_RHA_TESTMODULE.RHA_TEST_MOD_1.TESTVALUE_INTERNAL[21] / D
    The start point is clocked by            DATA_ACQUISITION_BLOCK|RHA_ADC_clk_TEST_inferred_clock [falling] on pin CLK
    The end   point is clocked by            DATA_ACQUISITION_BLOCK|RHA_ADC_clk_TEST_inferred_clock [rising] on pin CLK

Instance / Net                                                                                            Pin      Pin               Arrival     No. of    
Name                                                                                           Type       Name     Dir     Delay     Time        Fan Out(s)
-----------------------------------------------------------------------------------------------------------------------------------------------------------
Inst_DATA_ACQUISITION_BLOCK.Inst_RHA_TESTMODULE.RHA_TEST_MOD_1.SPIMACHINE_STATE[2]             DFN0E0     Q        Out     1.570     1.570       -         
SPIMACHINE_STATE[2]                                                                            Net        -        -       4.105     -           10        
Inst_DATA_ACQUISITION_BLOCK.Inst_RHA_TESTMODULE.RHA_TEST_MOD_1.SPIMACHINE_STATE_RNI7VEB[1]     OR2A       A        In      -         5.676       -         
Inst_DATA_ACQUISITION_BLOCK.Inst_RHA_TESTMODULE.RHA_TEST_MOD_1.SPIMACHINE_STATE_RNI7VEB[1]     OR2A       Y        Out     1.119     6.795       -         
N_42                                                                                           Net        -        -       3.420     -           6         
Inst_DATA_ACQUISITION_BLOCK.Inst_RHA_TESTMODULE.RHA_TEST_MOD_1.SPIMACHINE_STATE_RNIDH6H[4]     NOR2A      B        In      -         10.215      -         
Inst_DATA_ACQUISITION_BLOCK.Inst_RHA_TESTMODULE.RHA_TEST_MOD_1.SPIMACHINE_STATE_RNIDH6H[4]     NOR2A      Y        Out     0.927     11.142      -         
N_112                                                                                          Net        -        -       2.844     -           4         
Inst_DATA_ACQUISITION_BLOCK.Inst_RHA_TESTMODULE.RHA_TEST_MOD_1.SPIMACHINE_STATE_RNIKGLS[3]     NOR2B      A        In      -         13.986      -         
Inst_DATA_ACQUISITION_BLOCK.Inst_RHA_TESTMODULE.RHA_TEST_MOD_1.SPIMACHINE_STATE_RNIKGLS[3]     NOR2B      Y        Out     1.236     15.223      -         
adc_out127                                                                                     Net        -        -       5.855     -           23        
Inst_DATA_ACQUISITION_BLOCK.Inst_RHA_TESTMODULE.RHA_TEST_MOD_1.TESTVALUE_INTERNAL_RNO[21]      OR2        B        In      -         21.078      -         
Inst_DATA_ACQUISITION_BLOCK.Inst_RHA_TESTMODULE.RHA_TEST_MOD_1.TESTVALUE_INTERNAL_RNO[21]      OR2        Y        Out     1.554     22.632      -         
TESTVALUE_INTERNAL_RNO[21]                                                                     Net        -        -       0.773     -           1         
Inst_DATA_ACQUISITION_BLOCK.Inst_RHA_TESTMODULE.RHA_TEST_MOD_1.TESTVALUE_INTERNAL[21]          DFN1C1     D        In      -         23.404      -         
===========================================================================================================================================================
Total path delay (propagation time + setup) of 24.782 is 7.785(31.4%) logic and 16.998(68.6%) route.
Path delay compensated for clock skew. Clock skew is added to clock-to-out value, and is subtracted from setup time value


Path information for path number 3: 
      Requested Period:                      5.000
    - Setup time:                            1.378
    + Clock delay at ending point:           0.000 (ideal)
    = Required time:                         3.622

    - Propagation time:                      23.404
    - Clock delay at starting point:         0.000 (ideal)
    = Slack (non-critical) :                 -19.782

    Number of logic level(s):                4
    Starting point:                          Inst_DATA_ACQUISITION_BLOCK.Inst_RHA_TESTMODULE.RHA_TEST_MOD_1.SPIMACHINE_STATE[2] / Q
    Ending point:                            Inst_DATA_ACQUISITION_BLOCK.Inst_RHA_TESTMODULE.RHA_TEST_MOD_1.TESTVALUE_INTERNAL[1] / D
    The start point is clocked by            DATA_ACQUISITION_BLOCK|RHA_ADC_clk_TEST_inferred_clock [falling] on pin CLK
    The end   point is clocked by            DATA_ACQUISITION_BLOCK|RHA_ADC_clk_TEST_inferred_clock [rising] on pin CLK

Instance / Net                                                                                            Pin      Pin               Arrival     No. of    
Name                                                                                           Type       Name     Dir     Delay     Time        Fan Out(s)
-----------------------------------------------------------------------------------------------------------------------------------------------------------
Inst_DATA_ACQUISITION_BLOCK.Inst_RHA_TESTMODULE.RHA_TEST_MOD_1.SPIMACHINE_STATE[2]             DFN0E0     Q        Out     1.570     1.570       -         
SPIMACHINE_STATE[2]                                                                            Net        -        -       4.105     -           10        
Inst_DATA_ACQUISITION_BLOCK.Inst_RHA_TESTMODULE.RHA_TEST_MOD_1.SPIMACHINE_STATE_RNI7VEB[1]     OR2A       A        In      -         5.676       -         
Inst_DATA_ACQUISITION_BLOCK.Inst_RHA_TESTMODULE.RHA_TEST_MOD_1.SPIMACHINE_STATE_RNI7VEB[1]     OR2A       Y        Out     1.119     6.795       -         
N_42                                                                                           Net        -        -       3.420     -           6         
Inst_DATA_ACQUISITION_BLOCK.Inst_RHA_TESTMODULE.RHA_TEST_MOD_1.SPIMACHINE_STATE_RNIDH6H[4]     NOR2A      B        In      -         10.215      -         
Inst_DATA_ACQUISITION_BLOCK.Inst_RHA_TESTMODULE.RHA_TEST_MOD_1.SPIMACHINE_STATE_RNIDH6H[4]     NOR2A      Y        Out     0.927     11.142      -         
N_112                                                                                          Net        -        -       2.844     -           4         
Inst_DATA_ACQUISITION_BLOCK.Inst_RHA_TESTMODULE.RHA_TEST_MOD_1.SPIMACHINE_STATE_RNIKGLS[3]     NOR2B      A        In      -         13.986      -         
Inst_DATA_ACQUISITION_BLOCK.Inst_RHA_TESTMODULE.RHA_TEST_MOD_1.SPIMACHINE_STATE_RNIKGLS[3]     NOR2B      Y        Out     1.236     15.223      -         
adc_out127                                                                                     Net        -        -       5.855     -           23        
Inst_DATA_ACQUISITION_BLOCK.Inst_RHA_TESTMODULE.RHA_TEST_MOD_1.TESTVALUE_INTERNAL_RNO[1]       OR2        B        In      -         21.078      -         
Inst_DATA_ACQUISITION_BLOCK.Inst_RHA_TESTMODULE.RHA_TEST_MOD_1.TESTVALUE_INTERNAL_RNO[1]       OR2        Y        Out     1.554     22.632      -         
TESTVALUE_INTERNAL_RNO_0[1]                                                                    Net        -        -       0.773     -           1         
Inst_DATA_ACQUISITION_BLOCK.Inst_RHA_TESTMODULE.RHA_TEST_MOD_1.TESTVALUE_INTERNAL[1]           DFN1C1     D        In      -         23.404      -         
===========================================================================================================================================================
Total path delay (propagation time + setup) of 24.782 is 7.785(31.4%) logic and 16.998(68.6%) route.
Path delay compensated for clock skew. Clock skew is added to clock-to-out value, and is subtracted from setup time value


Path information for path number 4: 
      Requested Period:                      5.000
    - Setup time:                            1.295
    + Clock delay at ending point:           0.000 (ideal)
    = Required time:                         3.705

    - Propagation time:                      23.413
    - Clock delay at starting point:         0.000 (ideal)
    = Slack (non-critical) :                 -19.707

    Number of logic level(s):                4
    Starting point:                          Inst_DATA_ACQUISITION_BLOCK.Inst_RHA_TESTMODULE.RHA_TEST_MOD_1.SPIMACHINE_STATE[1] / Q
    Ending point:                            Inst_DATA_ACQUISITION_BLOCK.Inst_RHA_TESTMODULE.RHA_TEST_MOD_1.TESTVALUE_INTERNAL[0] / D
    The start point is clocked by            DATA_ACQUISITION_BLOCK|RHA_ADC_clk_TEST_inferred_clock [falling] on pin CLK
    The end   point is clocked by            DATA_ACQUISITION_BLOCK|RHA_ADC_clk_TEST_inferred_clock [rising] on pin CLK

Instance / Net                                                                                            Pin      Pin               Arrival     No. of    
Name                                                                                           Type       Name     Dir     Delay     Time        Fan Out(s)
-----------------------------------------------------------------------------------------------------------------------------------------------------------
Inst_DATA_ACQUISITION_BLOCK.Inst_RHA_TESTMODULE.RHA_TEST_MOD_1.SPIMACHINE_STATE[1]             DFN0E0     Q        Out     1.570     1.570       -         
SPIMACHINE_STATE[1]                                                                            Net        -        -       4.009     -           9         
Inst_DATA_ACQUISITION_BLOCK.Inst_RHA_TESTMODULE.RHA_TEST_MOD_1.SPIMACHINE_STATE_RNI7VEB[1]     OR2A       B        In      -         5.580       -         
Inst_DATA_ACQUISITION_BLOCK.Inst_RHA_TESTMODULE.RHA_TEST_MOD_1.SPIMACHINE_STATE_RNI7VEB[1]     OR2A       Y        Out     1.554     7.133       -         
N_42                                                                                           Net        -        -       3.420     -           6         
Inst_DATA_ACQUISITION_BLOCK.Inst_RHA_TESTMODULE.RHA_TEST_MOD_1.SPIMACHINE_STATE_RNIDH6H[4]     NOR2A      B        In      -         10.554      -         
Inst_DATA_ACQUISITION_BLOCK.Inst_RHA_TESTMODULE.RHA_TEST_MOD_1.SPIMACHINE_STATE_RNIDH6H[4]     NOR2A      Y        Out     0.977     11.531      -         
N_112                                                                                          Net        -        -       2.844     -           4         
Inst_DATA_ACQUISITION_BLOCK.Inst_RHA_TESTMODULE.RHA_TEST_MOD_1.SPIMACHINE_STATE_RNIKGLS[3]     NOR2B      A        In      -         14.375      -         
Inst_DATA_ACQUISITION_BLOCK.Inst_RHA_TESTMODULE.RHA_TEST_MOD_1.SPIMACHINE_STATE_RNIKGLS[3]     NOR2B      Y        Out     1.174     15.548      -         
adc_out127                                                                                     Net        -        -       5.855     -           23        
Inst_DATA_ACQUISITION_BLOCK.Inst_RHA_TESTMODULE.RHA_TEST_MOD_1.TESTVALUE_INTERNAL_RNO[0]       OR2        B        In      -         21.404      -         
Inst_DATA_ACQUISITION_BLOCK.Inst_RHA_TESTMODULE.RHA_TEST_MOD_1.TESTVALUE_INTERNAL_RNO[0]       OR2        Y        Out     1.236     22.640      -         
TESTVALUE_INTERNAL_RNO_0[0]                                                                    Net        -        -       0.773     -           1         
Inst_DATA_ACQUISITION_BLOCK.Inst_RHA_TESTMODULE.RHA_TEST_MOD_1.TESTVALUE_INTERNAL[0]           DFN1C1     D        In      -         23.413      -         
===========================================================================================================================================================
Total path delay (propagation time + setup) of 24.707 is 7.805(31.6%) logic and 16.902(68.4%) route.
Path delay compensated for clock skew. Clock skew is added to clock-to-out value, and is subtracted from setup time value


Path information for path number 5: 
      Requested Period:                      5.000
    - Setup time:                            1.295
    + Clock delay at ending point:           0.000 (ideal)
    = Required time:                         3.705

    - Propagation time:                      23.413
    - Clock delay at starting point:         0.000 (ideal)
    = Slack (non-critical) :                 -19.707

    Number of logic level(s):                4
    Starting point:                          Inst_DATA_ACQUISITION_BLOCK.Inst_RHA_TESTMODULE.RHA_TEST_MOD_1.SPIMACHINE_STATE[1] / Q
    Ending point:                            Inst_DATA_ACQUISITION_BLOCK.Inst_RHA_TESTMODULE.RHA_TEST_MOD_1.TESTVALUE_INTERNAL[21] / D
    The start point is clocked by            DATA_ACQUISITION_BLOCK|RHA_ADC_clk_TEST_inferred_clock [falling] on pin CLK
    The end   point is clocked by            DATA_ACQUISITION_BLOCK|RHA_ADC_clk_TEST_inferred_clock [rising] on pin CLK

Instance / Net                                                                                            Pin      Pin               Arrival     No. of    
Name                                                                                           Type       Name     Dir     Delay     Time        Fan Out(s)
-----------------------------------------------------------------------------------------------------------------------------------------------------------
Inst_DATA_ACQUISITION_BLOCK.Inst_RHA_TESTMODULE.RHA_TEST_MOD_1.SPIMACHINE_STATE[1]             DFN0E0     Q        Out     1.570     1.570       -         
SPIMACHINE_STATE[1]                                                                            Net        -        -       4.009     -           9         
Inst_DATA_ACQUISITION_BLOCK.Inst_RHA_TESTMODULE.RHA_TEST_MOD_1.SPIMACHINE_STATE_RNI7VEB[1]     OR2A       B        In      -         5.580       -         
Inst_DATA_ACQUISITION_BLOCK.Inst_RHA_TESTMODULE.RHA_TEST_MOD_1.SPIMACHINE_STATE_RNI7VEB[1]     OR2A       Y        Out     1.554     7.133       -         
N_42                                                                                           Net        -        -       3.420     -           6         
Inst_DATA_ACQUISITION_BLOCK.Inst_RHA_TESTMODULE.RHA_TEST_MOD_1.SPIMACHINE_STATE_RNIDH6H[4]     NOR2A      B        In      -         10.554      -         
Inst_DATA_ACQUISITION_BLOCK.Inst_RHA_TESTMODULE.RHA_TEST_MOD_1.SPIMACHINE_STATE_RNIDH6H[4]     NOR2A      Y        Out     0.977     11.531      -         
N_112                                                                                          Net        -        -       2.844     -           4         
Inst_DATA_ACQUISITION_BLOCK.Inst_RHA_TESTMODULE.RHA_TEST_MOD_1.SPIMACHINE_STATE_RNIKGLS[3]     NOR2B      A        In      -         14.375      -         
Inst_DATA_ACQUISITION_BLOCK.Inst_RHA_TESTMODULE.RHA_TEST_MOD_1.SPIMACHINE_STATE_RNIKGLS[3]     NOR2B      Y        Out     1.174     15.548      -         
adc_out127                                                                                     Net        -        -       5.855     -           23        
Inst_DATA_ACQUISITION_BLOCK.Inst_RHA_TESTMODULE.RHA_TEST_MOD_1.TESTVALUE_INTERNAL_RNO[21]      OR2        B        In      -         21.404      -         
Inst_DATA_ACQUISITION_BLOCK.Inst_RHA_TESTMODULE.RHA_TEST_MOD_1.TESTVALUE_INTERNAL_RNO[21]      OR2        Y        Out     1.236     22.640      -         
TESTVALUE_INTERNAL_RNO[21]                                                                     Net        -        -       0.773     -           1         
Inst_DATA_ACQUISITION_BLOCK.Inst_RHA_TESTMODULE.RHA_TEST_MOD_1.TESTVALUE_INTERNAL[21]          DFN1C1     D        In      -         23.413      -         
===========================================================================================================================================================
Total path delay (propagation time + setup) of 24.707 is 7.805(31.6%) logic and 16.902(68.4%) route.
Path delay compensated for clock skew. Clock skew is added to clock-to-out value, and is subtracted from setup time value


====================================
Detailed Report for Clock: IMPLANT_TOP|Clk
====================================


Starting Points with Worst Slack
********************************

                                                                                     Starting                                                                         Arrival            
Instance                                                                             Reference           Type         Pin     Net                                     Time        Slack  
                                                                                     Clock                                                                                               
-----------------------------------------------------------------------------------------------------------------------------------------------------------------------------------------
Inst_DATA_ACQUISITION_BLOCK.Inst_RHA_TO_ZL_CONVERTER.COUNTER_BITS_OF_A_BYTE[1]       IMPLANT_TOP|Clk     DFN1C1       Q       COUNTER_BITS_OF_A_BYTE[1]               1.771       -33.091
Inst_DATA_ACQUISITION_BLOCK.Inst_RHA_TO_ZL_CONVERTER.SPIMACHINE_STATE[1]             IMPLANT_TOP|Clk     DFN1E0       Q       SPIMACHINE_STATE[1]                     1.771       -33.020
Inst_DATA_ACQUISITION_BLOCK.Inst_RHA_TO_ZL_CONVERTER.SPIMACHINE_STATE[4]             IMPLANT_TOP|Clk     DFN1E0       Q       SPIMACHINE_STATE[4]                     1.771       -32.798
Inst_DATA_ACQUISITION_BLOCK.Inst_RHA_TO_ZL_CONVERTER.COUNTER_BITS_OF_A_BYTE[2]       IMPLANT_TOP|Clk     DFN1C1       Q       COUNTER_BITS_OF_A_BYTE[2]               1.771       -32.757
Inst_DATA_ACQUISITION_BLOCK.Inst_RHA_TO_ZL_CONVERTER.SPIMACHINE_STATE[3]             IMPLANT_TOP|Clk     DFN1E0       Q       SPIMACHINE_STATE[3]                     1.771       -32.431
Inst_DATA_ACQUISITION_BLOCK.Inst_RHA_TO_ZL_CONVERTER.COUNTER_BITS_OF_A_BYTE[0]       IMPLANT_TOP|Clk     DFN1C1       Q       COUNTER_BITS_OF_A_BYTE[0]               1.771       -32.189
Inst_DATA_ACQUISITION_BLOCK.Inst_RHA_TO_ZL_CONVERTER.COUNTER_BITS_OF_A_BYTE[3]       IMPLANT_TOP|Clk     DFN1C1       Q       COUNTER_BITS_OF_A_BYTE[3]               1.771       -31.349
Inst_CONTROL_NEXUS.Inst_ZARLINK_CONNECT_INIT.Zarlink_ConnectionTimeoutCounter[0]     IMPLANT_TOP|Clk     DFN1C1       Q       Zarlink_ConnectionTimeoutCounter[0]     1.395       -30.836
Inst_CONTROL_NEXUS.Inst_COMMAND_RECEIVER.ChannelMask_INTERNAL[48]                    IMPLANT_TOP|Clk     DFN1E1C1     Q       ChannelMask[48]                         1.771       -30.067
Inst_DATA_ACQUISITION_BLOCK.Inst_RHA_TO_ZL_CONVERTER.SPIMACHINE_STATE[2]             IMPLANT_TOP|Clk     DFN1E0       Q       SPIMACHINE_STATE[2]                     1.771       -29.963
=========================================================================================================================================================================================


Ending Points with Worst Slack
******************************

                                                                                      Starting                                                                          Required            
Instance                                                                              Reference           Type         Pin     Net                                      Time         Slack  
                                                                                      Clock                                                                                                 
--------------------------------------------------------------------------------------------------------------------------------------------------------------------------------------------
Inst_DATA_ACQUISITION_BLOCK.Inst_RHA_TO_ZL_CONVERTER.COUNTER_BITS_PROCESSED[6]        IMPLANT_TOP|Clk     DFN1C1       D       COUNTER_BITS_PROCESSED_9[6]              8.705        -33.091
Inst_DATA_ACQUISITION_BLOCK.Inst_RHA_TO_ZL_CONVERTER.COUNTER_BITS_PROCESSED[5]        IMPLANT_TOP|Clk     DFN1C1       D       COUNTER_BITS_PROCESSED_9[5]              8.705        -32.857
Inst_DATA_ACQUISITION_BLOCK.Inst_RHA_TO_ZL_CONVERTER.COUNTER_BITS_PROCESSED[4]        IMPLANT_TOP|Clk     DFN1C1       D       COUNTER_BITS_PROCESSED_9[4]              8.705        -31.082
Inst_CONTROL_NEXUS.Inst_ZARLINK_CONNECT_INIT.Zarlink_ConnectionTimeoutCounter[31]     IMPLANT_TOP|Clk     DFN1C1       D       Zarlink_ConnectionTimeoutCounter_n31     8.705        -30.836
Inst_DATA_ACQUISITION_BLOCK.Inst_RHA_ARRAY.DATAOUT_VALIDSTRING[0]                     IMPLANT_TOP|Clk     DFN1E0C1     D       DATAOUT_VALIDSTRING_5[0]                 8.705        -30.067
Inst_DATA_ACQUISITION_BLOCK.Inst_RHA_ARRAY.DATAOUT_VALIDSTRING[1]                     IMPLANT_TOP|Clk     DFN1E0C1     D       DATAOUT_VALIDSTRING_5[1]                 8.705        -30.067
Inst_DATA_ACQUISITION_BLOCK.Inst_RHA_ARRAY.DATAOUT_VALIDSTRING[2]                     IMPLANT_TOP|Clk     DFN1E0C1     D       DATAOUT_VALIDSTRING_5[2]                 8.705        -30.067
Inst_DATA_ACQUISITION_BLOCK.Inst_RHA_ARRAY.DATAOUT_VALIDSTRING[3]                     IMPLANT_TOP|Clk     DFN1E0C1     D       DATAOUT_VALIDSTRING_5[3]                 8.705        -30.067
Inst_DATA_ACQUISITION_BLOCK.Inst_RHA_ARRAY.DATAOUT_VALIDSTRING[4]                     IMPLANT_TOP|Clk     DFN1E0C1     D       DATAOUT_VALIDSTRING_5[4]                 8.705        -30.067
Inst_DATA_ACQUISITION_BLOCK.Inst_RHA_ARRAY.DATAOUT_VALIDSTRING[5]                     IMPLANT_TOP|Clk     DFN1E0C1     D       DATAOUT_VALIDSTRING_5[5]                 8.705        -30.067
============================================================================================================================================================================================


Worst Path Information
View Worst Path in Analyst
***********************


Path information for path number 1: 
      Requested Period:                      10.000
    - Setup time:                            1.295
    + Clock delay at ending point:           0.000 (ideal)
    = Required time:                         8.705

    - Propagation time:                      41.796
    - Clock delay at starting point:         0.000 (ideal)
    = Slack (critical) :                     -33.091

    Number of logic level(s):                12
    Starting point:                          Inst_DATA_ACQUISITION_BLOCK.Inst_RHA_TO_ZL_CONVERTER.COUNTER_BITS_OF_A_BYTE[1] / Q
    Ending point:                            Inst_DATA_ACQUISITION_BLOCK.Inst_RHA_TO_ZL_CONVERTER.COUNTER_BITS_PROCESSED[6] / D
    The start point is clocked by            IMPLANT_TOP|Clk [rising] on pin CLK
    The end   point is clocked by            IMPLANT_TOP|Clk [rising] on pin CLK

Instance / Net                                                                                         Pin      Pin               Arrival     No. of    
Name                                                                                        Type       Name     Dir     Delay     Time        Fan Out(s)
--------------------------------------------------------------------------------------------------------------------------------------------------------
Inst_DATA_ACQUISITION_BLOCK.Inst_RHA_TO_ZL_CONVERTER.COUNTER_BITS_OF_A_BYTE[1]              DFN1C1     Q        Out     1.771     1.771       -         
COUNTER_BITS_OF_A_BYTE[1]                                                                   Net        -        -       1.938     -           3         
Inst_DATA_ACQUISITION_BLOCK.Inst_RHA_TO_ZL_CONVERTER.COUNTER_BITS_OF_A_BYTE_RNIP0GI[1]      NOR2       B        In      -         3.708       -         
Inst_DATA_ACQUISITION_BLOCK.Inst_RHA_TO_ZL_CONVERTER.COUNTER_BITS_OF_A_BYTE_RNIP0GI[1]      NOR2       Y        Out     1.554     5.262       -         
un6_counter_bits_of_a_byte_2                                                                Net        -        -       1.938     -           3         
Inst_DATA_ACQUISITION_BLOCK.Inst_RHA_TO_ZL_CONVERTER.COUNTER_BITS_OF_A_BYTE_RNII1051[3]     NOR2B      B        In      -         7.200       -         
Inst_DATA_ACQUISITION_BLOCK.Inst_RHA_TO_ZL_CONVERTER.COUNTER_BITS_OF_A_BYTE_RNII1051[3]     NOR2B      Y        Out     1.240     8.440       -         
un6_counter_bits_of_a_byte                                                                  Net        -        -       3.667     -           7         
Inst_DATA_ACQUISITION_BLOCK.Inst_RHA_TO_ZL_CONVERTER.BITS_TO_PROCESS_RNI7FG91[0]            OR2A       B        In      -         12.107      -         
Inst_DATA_ACQUISITION_BLOCK.Inst_RHA_TO_ZL_CONVERTER.BITS_TO_PROCESS_RNI7FG91[0]            OR2A       Y        Out     1.236     13.343      -         
un7_counter_bits_of_a_byte                                                                  Net        -        -       3.074     -           5         
Inst_DATA_ACQUISITION_BLOCK.Inst_RHA_TO_ZL_CONVERTER.BITMASK_SAMPLE_COPY_RNI10C32[0]        OA1A       B        In      -         16.417      -         
Inst_DATA_ACQUISITION_BLOCK.Inst_RHA_TO_ZL_CONVERTER.BITMASK_SAMPLE_COPY_RNI10C32[0]        OA1A       Y        Out     2.168     18.584      -         
COUNTER_BITS_PROCESSED_0_sqmuxa_2                                                           Net        -        -       1.938     -           3         
Inst_DATA_ACQUISITION_BLOCK.Inst_RHA_TO_ZL_CONVERTER.SPIMACHINE_STATE_3_RNIQOHDC[0]         OR3        C        In      -         20.522      -         
Inst_DATA_ACQUISITION_BLOCK.Inst_RHA_TO_ZL_CONVERTER.SPIMACHINE_STATE_3_RNIQOHDC[0]         OR3        Y        Out     1.641     22.164      -         
un1_spimachine_state72_4_1                                                                  Net        -        -       0.773     -           1         
Inst_DATA_ACQUISITION_BLOCK.Inst_RHA_TO_ZL_CONVERTER.SPIMACHINE_STATE_0_RNIVF3AE[0]         NOR2A      B        In      -         22.936      -         
Inst_DATA_ACQUISITION_BLOCK.Inst_RHA_TO_ZL_CONVERTER.SPIMACHINE_STATE_0_RNIVF3AE[0]         NOR2A      Y        Out     0.927     23.863      -         
SPIMACHINE_STATE_0_RNIVF3AE[0]                                                              Net        -        -       1.938     -           3         
Inst_DATA_ACQUISITION_BLOCK.Inst_RHA_TO_ZL_CONVERTER.un1_COUNTER_BITS_PROCESSED_1.I_1       AND2       B        In      -         25.801      -         
Inst_DATA_ACQUISITION_BLOCK.Inst_RHA_TO_ZL_CONVERTER.un1_COUNTER_BITS_PROCESSED_1.I_1       AND2       Y        Out     1.508     27.309      -         
DWACT_ADD_CI_0_TMP[0]                                                                       Net        -        -       0.927     -           2         
Inst_DATA_ACQUISITION_BLOCK.Inst_RHA_TO_ZL_CONVERTER.un1_COUNTER_BITS_PROCESSED_1.I_32      NOR2B      A        In      -         28.236      -         
Inst_DATA_ACQUISITION_BLOCK.Inst_RHA_TO_ZL_CONVERTER.un1_COUNTER_BITS_PROCESSED_1.I_32      NOR2B      Y        Out     1.236     29.472      -         
DWACT_ADD_CI_0_g_array_1[0]                                                                 Net        -        -       1.938     -           3         
Inst_DATA_ACQUISITION_BLOCK.Inst_RHA_TO_ZL_CONVERTER.un1_COUNTER_BITS_PROCESSED_1.I_38      NOR2B      A        In      -         31.410      -         
Inst_DATA_ACQUISITION_BLOCK.Inst_RHA_TO_ZL_CONVERTER.un1_COUNTER_BITS_PROCESSED_1.I_38      NOR2B      Y        Out     1.236     32.646      -         
DWACT_ADD_CI_0_g_array_2[0]                                                                 Net        -        -       1.938     -           3         
Inst_DATA_ACQUISITION_BLOCK.Inst_RHA_TO_ZL_CONVERTER.un1_COUNTER_BITS_PROCESSED_1.I_35      NOR2B      A        In      -         34.584      -         
Inst_DATA_ACQUISITION_BLOCK.Inst_RHA_TO_ZL_CONVERTER.un1_COUNTER_BITS_PROCESSED_1.I_35      NOR2B      Y        Out     1.236     35.820      -         
DWACT_ADD_CI_0_g_array_11[0]                                                                Net        -        -       0.773     -           1         
Inst_DATA_ACQUISITION_BLOCK.Inst_RHA_TO_ZL_CONVERTER.un1_COUNTER_BITS_PROCESSED_1.I_28      XOR2       B        In      -         36.593      -         
Inst_DATA_ACQUISITION_BLOCK.Inst_RHA_TO_ZL_CONVERTER.un1_COUNTER_BITS_PROCESSED_1.I_28      XOR2       Y        Out     2.251     38.844      -         
I_28                                                                                        Net        -        -       0.773     -           1         
Inst_DATA_ACQUISITION_BLOCK.Inst_RHA_TO_ZL_CONVERTER.COUNTER_BITS_PROCESSED_RNO[6]          MX2        B        In      -         39.616      -         
Inst_DATA_ACQUISITION_BLOCK.Inst_RHA_TO_ZL_CONVERTER.COUNTER_BITS_PROCESSED_RNO[6]          MX2        Y        Out     1.407     41.024      -         
COUNTER_BITS_PROCESSED_9[6]                                                                 Net        -        -       0.773     -           1         
Inst_DATA_ACQUISITION_BLOCK.Inst_RHA_TO_ZL_CONVERTER.COUNTER_BITS_PROCESSED[6]              DFN1C1     D        In      -         41.796      -         
========================================================================================================================================================
Total path delay (propagation time + setup) of 43.091 is 20.706(48.1%) logic and 22.385(51.9%) route.
Path delay compensated for clock skew. Clock skew is added to clock-to-out value, and is subtracted from setup time value


Path information for path number 2: 
      Requested Period:                      10.000
    - Setup time:                            1.295
    + Clock delay at ending point:           0.000 (ideal)
    = Required time:                         8.705

    - Propagation time:                      41.725
    - Clock delay at starting point:         0.000 (ideal)
    = Slack (non-critical) :                 -33.020

    Number of logic level(s):                11
    Starting point:                          Inst_DATA_ACQUISITION_BLOCK.Inst_RHA_TO_ZL_CONVERTER.SPIMACHINE_STATE[1] / Q
    Ending point:                            Inst_DATA_ACQUISITION_BLOCK.Inst_RHA_TO_ZL_CONVERTER.COUNTER_BITS_PROCESSED[6] / D
    The start point is clocked by            IMPLANT_TOP|Clk [rising] on pin CLK
    The end   point is clocked by            IMPLANT_TOP|Clk [rising] on pin CLK

Instance / Net                                                                                        Pin      Pin               Arrival     No. of    
Name                                                                                       Type       Name     Dir     Delay     Time        Fan Out(s)
-------------------------------------------------------------------------------------------------------------------------------------------------------
Inst_DATA_ACQUISITION_BLOCK.Inst_RHA_TO_ZL_CONVERTER.SPIMACHINE_STATE[1]                   DFN1E0     Q        Out     1.771     1.771       -         
SPIMACHINE_STATE[1]                                                                        Net        -        -       5.926     -           24        
Inst_DATA_ACQUISITION_BLOCK.Inst_RHA_TO_ZL_CONVERTER.SPIMACHINE_STATE_RNIKFP9[3]           NOR3B      A        In      -         7.697       -         
Inst_DATA_ACQUISITION_BLOCK.Inst_RHA_TO_ZL_CONVERTER.SPIMACHINE_STATE_RNIKFP9[3]           NOR3B      Y        Out     1.541     9.238       -         
spimachine_state78_1                                                                       Net        -        -       3.420     -           6         
Inst_DATA_ACQUISITION_BLOCK.Inst_RHA_TO_ZL_CONVERTER.SPIMACHINE_STATE_0_RNISSMD[0]         NOR3B      B        In      -         12.658      -         
Inst_DATA_ACQUISITION_BLOCK.Inst_RHA_TO_ZL_CONVERTER.SPIMACHINE_STATE_0_RNISSMD[0]         NOR3B      Y        Out     1.458     14.116      -         
spimachine_state78                                                                         Net        -        -       3.667     -           7         
Inst_DATA_ACQUISITION_BLOCK.Inst_RHA_TO_ZL_CONVERTER.BITMASK_SAMPLE_COPY_RNI10C32[0]       OA1A       C        In      -         17.783      -         
Inst_DATA_ACQUISITION_BLOCK.Inst_RHA_TO_ZL_CONVERTER.BITMASK_SAMPLE_COPY_RNI10C32[0]       OA1A       Y        Out     0.973     18.756      -         
COUNTER_BITS_PROCESSED_0_sqmuxa_2                                                          Net        -        -       1.938     -           3         
Inst_DATA_ACQUISITION_BLOCK.Inst_RHA_TO_ZL_CONVERTER.SPIMACHINE_STATE_3_RNIQOHDC[0]        OR3        C        In      -         20.693      -         
Inst_DATA_ACQUISITION_BLOCK.Inst_RHA_TO_ZL_CONVERTER.SPIMACHINE_STATE_3_RNIQOHDC[0]        OR3        Y        Out     1.804     22.497      -         
un1_spimachine_state72_4_1                                                                 Net        -        -       0.773     -           1         
Inst_DATA_ACQUISITION_BLOCK.Inst_RHA_TO_ZL_CONVERTER.SPIMACHINE_STATE_0_RNIVF3AE[0]        NOR2A      B        In      -         23.270      -         
Inst_DATA_ACQUISITION_BLOCK.Inst_RHA_TO_ZL_CONVERTER.SPIMACHINE_STATE_0_RNIVF3AE[0]        NOR2A      Y        Out     0.977     24.247      -         
SPIMACHINE_STATE_0_RNIVF3AE[0]                                                             Net        -        -       1.938     -           3         
Inst_DATA_ACQUISITION_BLOCK.Inst_RHA_TO_ZL_CONVERTER.un1_COUNTER_BITS_PROCESSED_1.I_1      AND2       B        In      -         26.185      -         
Inst_DATA_ACQUISITION_BLOCK.Inst_RHA_TO_ZL_CONVERTER.un1_COUNTER_BITS_PROCESSED_1.I_1      AND2       Y        Out     1.240     27.425      -         
DWACT_ADD_CI_0_TMP[0]                                                                      Net        -        -       0.927     -           2         
Inst_DATA_ACQUISITION_BLOCK.Inst_RHA_TO_ZL_CONVERTER.un1_COUNTER_BITS_PROCESSED_1.I_32     NOR2B      A        In      -         28.353      -         
Inst_DATA_ACQUISITION_BLOCK.Inst_RHA_TO_ZL_CONVERTER.un1_COUNTER_BITS_PROCESSED_1.I_32     NOR2B      Y        Out     1.174     29.526      -         
DWACT_ADD_CI_0_g_array_1[0]                                                                Net        -        -       1.938     -           3         
Inst_DATA_ACQUISITION_BLOCK.Inst_RHA_TO_ZL_CONVERTER.un1_COUNTER_BITS_PROCESSED_1.I_38     NOR2B      A        In      -         31.464      -         
Inst_DATA_ACQUISITION_BLOCK.Inst_RHA_TO_ZL_CONVERTER.un1_COUNTER_BITS_PROCESSED_1.I_38     NOR2B      Y        Out     1.174     32.637      -         
DWACT_ADD_CI_0_g_array_2[0]                                                                Net        -        -       1.938     -           3         
Inst_DATA_ACQUISITION_BLOCK.Inst_RHA_TO_ZL_CONVERTER.un1_COUNTER_BITS_PROCESSED_1.I_35     NOR2B      A        In      -         34.575      -         
Inst_DATA_ACQUISITION_BLOCK.Inst_RHA_TO_ZL_CONVERTER.un1_COUNTER_BITS_PROCESSED_1.I_35     NOR2B      Y        Out     1.174     35.749      -         
DWACT_ADD_CI_0_g_array_11[0]                                                               Net        -        -       0.773     -           1         
Inst_DATA_ACQUISITION_BLOCK.Inst_RHA_TO_ZL_CONVERTER.un1_COUNTER_BITS_PROCESSED_1.I_28     XOR2       B        In      -         36.521      -         
Inst_DATA_ACQUISITION_BLOCK.Inst_RHA_TO_ZL_CONVERTER.un1_COUNTER_BITS_PROCESSED_1.I_28     XOR2       Y        Out     2.251     38.772      -         
I_28                                                                                       Net        -        -       0.773     -           1         
Inst_DATA_ACQUISITION_BLOCK.Inst_RHA_TO_ZL_CONVERTER.COUNTER_BITS_PROCESSED_RNO[6]         MX2        B        In      -         39.545      -         
Inst_DATA_ACQUISITION_BLOCK.Inst_RHA_TO_ZL_CONVERTER.COUNTER_BITS_PROCESSED_RNO[6]         MX2        Y        Out     1.407     40.952      -         
COUNTER_BITS_PROCESSED_9[6]                                                                Net        -        -       0.773     -           1         
Inst_DATA_ACQUISITION_BLOCK.Inst_RHA_TO_ZL_CONVERTER.COUNTER_BITS_PROCESSED[6]             DFN1C1     D        In      -         41.725      -         
=======================================================================================================================================================
Total path delay (propagation time + setup) of 43.020 is 18.237(42.4%) logic and 24.782(57.6%) route.
Path delay compensated for clock skew. Clock skew is added to clock-to-out value, and is subtracted from setup time value


Path information for path number 3: 
      Requested Period:                      10.000
    - Setup time:                            1.295
    + Clock delay at ending point:           0.000 (ideal)
    = Required time:                         8.705

    - Propagation time:                      41.562
    - Clock delay at starting point:         0.000 (ideal)
    = Slack (non-critical) :                 -32.857

    Number of logic level(s):                12
    Starting point:                          Inst_DATA_ACQUISITION_BLOCK.Inst_RHA_TO_ZL_CONVERTER.COUNTER_BITS_OF_A_BYTE[1] / Q
    Ending point:                            Inst_DATA_ACQUISITION_BLOCK.Inst_RHA_TO_ZL_CONVERTER.COUNTER_BITS_PROCESSED[5] / D
    The start point is clocked by            IMPLANT_TOP|Clk [rising] on pin CLK
    The end   point is clocked by            IMPLANT_TOP|Clk [rising] on pin CLK

Instance / Net                                                                                         Pin      Pin               Arrival     No. of    
Name                                                                                        Type       Name     Dir     Delay     Time        Fan Out(s)
--------------------------------------------------------------------------------------------------------------------------------------------------------
Inst_DATA_ACQUISITION_BLOCK.Inst_RHA_TO_ZL_CONVERTER.COUNTER_BITS_OF_A_BYTE[1]              DFN1C1     Q        Out     1.771     1.771       -         
COUNTER_BITS_OF_A_BYTE[1]                                                                   Net        -        -       1.938     -           3         
Inst_DATA_ACQUISITION_BLOCK.Inst_RHA_TO_ZL_CONVERTER.COUNTER_BITS_OF_A_BYTE_RNIP0GI[1]      NOR2       B        In      -         3.708       -         
Inst_DATA_ACQUISITION_BLOCK.Inst_RHA_TO_ZL_CONVERTER.COUNTER_BITS_OF_A_BYTE_RNIP0GI[1]      NOR2       Y        Out     1.554     5.262       -         
un6_counter_bits_of_a_byte_2                                                                Net        -        -       1.938     -           3         
Inst_DATA_ACQUISITION_BLOCK.Inst_RHA_TO_ZL_CONVERTER.COUNTER_BITS_OF_A_BYTE_RNII1051[3]     NOR2B      B        In      -         7.200       -         
Inst_DATA_ACQUISITION_BLOCK.Inst_RHA_TO_ZL_CONVERTER.COUNTER_BITS_OF_A_BYTE_RNII1051[3]     NOR2B      Y        Out     1.240     8.440       -         
un6_counter_bits_of_a_byte                                                                  Net        -        -       3.667     -           7         
Inst_DATA_ACQUISITION_BLOCK.Inst_RHA_TO_ZL_CONVERTER.BITS_TO_PROCESS_RNI7FG91[0]            OR2A       B        In      -         12.107      -         
Inst_DATA_ACQUISITION_BLOCK.Inst_RHA_TO_ZL_CONVERTER.BITS_TO_PROCESS_RNI7FG91[0]            OR2A       Y        Out     1.236     13.343      -         
un7_counter_bits_of_a_byte                                                                  Net        -        -       3.074     -           5         
Inst_DATA_ACQUISITION_BLOCK.Inst_RHA_TO_ZL_CONVERTER.BITMASK_SAMPLE_COPY_RNI10C32[0]        OA1A       B        In      -         16.417      -         
Inst_DATA_ACQUISITION_BLOCK.Inst_RHA_TO_ZL_CONVERTER.BITMASK_SAMPLE_COPY_RNI10C32[0]        OA1A       Y        Out     2.168     18.584      -         
COUNTER_BITS_PROCESSED_0_sqmuxa_2                                                           Net        -        -       1.938     -           3         
Inst_DATA_ACQUISITION_BLOCK.Inst_RHA_TO_ZL_CONVERTER.SPIMACHINE_STATE_3_RNIQOHDC[0]         OR3        C        In      -         20.522      -         
Inst_DATA_ACQUISITION_BLOCK.Inst_RHA_TO_ZL_CONVERTER.SPIMACHINE_STATE_3_RNIQOHDC[0]         OR3        Y        Out     1.641     22.164      -         
un1_spimachine_state72_4_1                                                                  Net        -        -       0.773     -           1         
Inst_DATA_ACQUISITION_BLOCK.Inst_RHA_TO_ZL_CONVERTER.SPIMACHINE_STATE_0_RNIVF3AE[0]         NOR2A      B        In      -         22.936      -         
Inst_DATA_ACQUISITION_BLOCK.Inst_RHA_TO_ZL_CONVERTER.SPIMACHINE_STATE_0_RNIVF3AE[0]         NOR2A      Y        Out     0.927     23.863      -         
SPIMACHINE_STATE_0_RNIVF3AE[0]                                                              Net        -        -       1.938     -           3         
Inst_DATA_ACQUISITION_BLOCK.Inst_RHA_TO_ZL_CONVERTER.un1_COUNTER_BITS_PROCESSED_1.I_1       AND2       B        In      -         25.801      -         
Inst_DATA_ACQUISITION_BLOCK.Inst_RHA_TO_ZL_CONVERTER.un1_COUNTER_BITS_PROCESSED_1.I_1       AND2       Y        Out     1.508     27.309      -         
DWACT_ADD_CI_0_TMP[0]                                                                       Net        -        -       0.927     -           2         
Inst_DATA_ACQUISITION_BLOCK.Inst_RHA_TO_ZL_CONVERTER.un1_COUNTER_BITS_PROCESSED_1.I_32      NOR2B      A        In      -         28.236      -         
Inst_DATA_ACQUISITION_BLOCK.Inst_RHA_TO_ZL_CONVERTER.un1_COUNTER_BITS_PROCESSED_1.I_32      NOR2B      Y        Out     1.236     29.472      -         
DWACT_ADD_CI_0_g_array_1[0]                                                                 Net        -        -       1.938     -           3         
Inst_DATA_ACQUISITION_BLOCK.Inst_RHA_TO_ZL_CONVERTER.un1_COUNTER_BITS_PROCESSED_1.I_38      NOR2B      A        In      -         31.410      -         
Inst_DATA_ACQUISITION_BLOCK.Inst_RHA_TO_ZL_CONVERTER.un1_COUNTER_BITS_PROCESSED_1.I_38      NOR2B      Y        Out     1.236     32.646      -         
DWACT_ADD_CI_0_g_array_2[0]                                                                 Net        -        -       1.938     -           3         
Inst_DATA_ACQUISITION_BLOCK.Inst_RHA_TO_ZL_CONVERTER.un1_COUNTER_BITS_PROCESSED_1.I_36      NOR2B      A        In      -         34.584      -         
Inst_DATA_ACQUISITION_BLOCK.Inst_RHA_TO_ZL_CONVERTER.un1_COUNTER_BITS_PROCESSED_1.I_36      NOR2B      Y        Out     1.236     35.820      -         
DWACT_ADD_CI_0_g_array_12_1[0]                                                              Net        -        -       0.773     -           1         
Inst_DATA_ACQUISITION_BLOCK.Inst_RHA_TO_ZL_CONVERTER.un1_COUNTER_BITS_PROCESSED_1.I_29      XOR2       B        In      -         36.593      -         
Inst_DATA_ACQUISITION_BLOCK.Inst_RHA_TO_ZL_CONVERTER.un1_COUNTER_BITS_PROCESSED_1.I_29      XOR2       Y        Out     2.251     38.844      -         
I_29                                                                                        Net        -        -       0.773     -           1         
Inst_DATA_ACQUISITION_BLOCK.Inst_RHA_TO_ZL_CONVERTER.COUNTER_BITS_PROCESSED_RNO[5]          NOR2B      A        In      -         39.616      -         
Inst_DATA_ACQUISITION_BLOCK.Inst_RHA_TO_ZL_CONVERTER.COUNTER_BITS_PROCESSED_RNO[5]          NOR2B      Y        Out     1.174     40.790      -         
COUNTER_BITS_PROCESSED_9[5]                                                                 Net        -        -       0.773     -           1         
Inst_DATA_ACQUISITION_BLOCK.Inst_RHA_TO_ZL_CONVERTER.COUNTER_BITS_PROCESSED[5]              DFN1C1     D        In      -         41.562      -         
========================================================================================================================================================
Total path delay (propagation time + setup) of 42.857 is 20.472(47.8%) logic and 22.385(52.2%) route.
Path delay compensated for clock skew. Clock skew is added to clock-to-out value, and is subtracted from setup time value


Path information for path number 4: 
      Requested Period:                      10.000
    - Setup time:                            1.295
    + Clock delay at ending point:           0.000 (ideal)
    = Required time:                         8.705

    - Propagation time:                      41.504
    - Clock delay at starting point:         0.000 (ideal)
    = Slack (non-critical) :                 -32.798

    Number of logic level(s):                11
    Starting point:                          Inst_DATA_ACQUISITION_BLOCK.Inst_RHA_TO_ZL_CONVERTER.SPIMACHINE_STATE[4] / Q
    Ending point:                            Inst_DATA_ACQUISITION_BLOCK.Inst_RHA_TO_ZL_CONVERTER.COUNTER_BITS_PROCESSED[6] / D
    The start point is clocked by            IMPLANT_TOP|Clk [rising] on pin CLK
    The end   point is clocked by            IMPLANT_TOP|Clk [rising] on pin CLK

Instance / Net                                                                                        Pin      Pin               Arrival     No. of    
Name                                                                                       Type       Name     Dir     Delay     Time        Fan Out(s)
-------------------------------------------------------------------------------------------------------------------------------------------------------
Inst_DATA_ACQUISITION_BLOCK.Inst_RHA_TO_ZL_CONVERTER.SPIMACHINE_STATE[4]                   DFN1E0     Q        Out     1.771     1.771       -         
SPIMACHINE_STATE[4]                                                                        Net        -        -       5.788     -           22        
Inst_DATA_ACQUISITION_BLOCK.Inst_RHA_TO_ZL_CONVERTER.SPIMACHINE_STATE_RNIKFP9[3]           NOR3B      B        In      -         7.559       -         
Inst_DATA_ACQUISITION_BLOCK.Inst_RHA_TO_ZL_CONVERTER.SPIMACHINE_STATE_RNIKFP9[3]           NOR3B      Y        Out     1.458     9.017       -         
spimachine_state78_1                                                                       Net        -        -       3.420     -           6         
Inst_DATA_ACQUISITION_BLOCK.Inst_RHA_TO_ZL_CONVERTER.SPIMACHINE_STATE_0_RNISSMD[0]         NOR3B      B        In      -         12.437      -         
Inst_DATA_ACQUISITION_BLOCK.Inst_RHA_TO_ZL_CONVERTER.SPIMACHINE_STATE_0_RNISSMD[0]         NOR3B      Y        Out     1.458     13.895      -         
spimachine_state78                                                                         Net        -        -       3.667     -           7         
Inst_DATA_ACQUISITION_BLOCK.Inst_RHA_TO_ZL_CONVERTER.BITMASK_SAMPLE_COPY_RNI10C32[0]       OA1A       C        In      -         17.561      -         
Inst_DATA_ACQUISITION_BLOCK.Inst_RHA_TO_ZL_CONVERTER.BITMASK_SAMPLE_COPY_RNI10C32[0]       OA1A       Y        Out     0.973     18.534      -         
COUNTER_BITS_PROCESSED_0_sqmuxa_2                                                          Net        -        -       1.938     -           3         
Inst_DATA_ACQUISITION_BLOCK.Inst_RHA_TO_ZL_CONVERTER.SPIMACHINE_STATE_3_RNIQOHDC[0]        OR3        C        In      -         20.472      -         
Inst_DATA_ACQUISITION_BLOCK.Inst_RHA_TO_ZL_CONVERTER.SPIMACHINE_STATE_3_RNIQOHDC[0]        OR3        Y        Out     1.804     22.276      -         
un1_spimachine_state72_4_1                                                                 Net        -        -       0.773     -           1         
Inst_DATA_ACQUISITION_BLOCK.Inst_RHA_TO_ZL_CONVERTER.SPIMACHINE_STATE_0_RNIVF3AE[0]        NOR2A      B        In      -         23.049      -         
Inst_DATA_ACQUISITION_BLOCK.Inst_RHA_TO_ZL_CONVERTER.SPIMACHINE_STATE_0_RNIVF3AE[0]        NOR2A      Y        Out     0.977     24.026      -         
SPIMACHINE_STATE_0_RNIVF3AE[0]                                                             Net        -        -       1.938     -           3         
Inst_DATA_ACQUISITION_BLOCK.Inst_RHA_TO_ZL_CONVERTER.un1_COUNTER_BITS_PROCESSED_1.I_1      AND2       B        In      -         25.964      -         
Inst_DATA_ACQUISITION_BLOCK.Inst_RHA_TO_ZL_CONVERTER.un1_COUNTER_BITS_PROCESSED_1.I_1      AND2       Y        Out     1.240     27.204      -         
DWACT_ADD_CI_0_TMP[0]                                                                      Net        -        -       0.927     -           2         
Inst_DATA_ACQUISITION_BLOCK.Inst_RHA_TO_ZL_CONVERTER.un1_COUNTER_BITS_PROCESSED_1.I_32     NOR2B      A        In      -         28.131      -         
Inst_DATA_ACQUISITION_BLOCK.Inst_RHA_TO_ZL_CONVERTER.un1_COUNTER_BITS_PROCESSED_1.I_32     NOR2B      Y        Out     1.174     29.305      -         
DWACT_ADD_CI_0_g_array_1[0]                                                                Net        -        -       1.938     -           3         
Inst_DATA_ACQUISITION_BLOCK.Inst_RHA_TO_ZL_CONVERTER.un1_COUNTER_BITS_PROCESSED_1.I_38     NOR2B      A        In      -         31.243      -         
Inst_DATA_ACQUISITION_BLOCK.Inst_RHA_TO_ZL_CONVERTER.un1_COUNTER_BITS_PROCESSED_1.I_38     NOR2B      Y        Out     1.174     32.416      -         
DWACT_ADD_CI_0_g_array_2[0]                                                                Net        -        -       1.938     -           3         
Inst_DATA_ACQUISITION_BLOCK.Inst_RHA_TO_ZL_CONVERTER.un1_COUNTER_BITS_PROCESSED_1.I_35     NOR2B      A        In      -         34.354      -         
Inst_DATA_ACQUISITION_BLOCK.Inst_RHA_TO_ZL_CONVERTER.un1_COUNTER_BITS_PROCESSED_1.I_35     NOR2B      Y        Out     1.174     35.527      -         
DWACT_ADD_CI_0_g_array_11[0]                                                               Net        -        -       0.773     -           1         
Inst_DATA_ACQUISITION_BLOCK.Inst_RHA_TO_ZL_CONVERTER.un1_COUNTER_BITS_PROCESSED_1.I_28     XOR2       B        In      -         36.300      -         
Inst_DATA_ACQUISITION_BLOCK.Inst_RHA_TO_ZL_CONVERTER.un1_COUNTER_BITS_PROCESSED_1.I_28     XOR2       Y        Out     2.251     38.551      -         
I_28                                                                                       Net        -        -       0.773     -           1         
Inst_DATA_ACQUISITION_BLOCK.Inst_RHA_TO_ZL_CONVERTER.COUNTER_BITS_PROCESSED_RNO[6]         MX2        B        In      -         39.324      -         
Inst_DATA_ACQUISITION_BLOCK.Inst_RHA_TO_ZL_CONVERTER.COUNTER_BITS_PROCESSED_RNO[6]         MX2        Y        Out     1.407     40.731      -         
COUNTER_BITS_PROCESSED_9[6]                                                                Net        -        -       0.773     -           1         
Inst_DATA_ACQUISITION_BLOCK.Inst_RHA_TO_ZL_CONVERTER.COUNTER_BITS_PROCESSED[6]             DFN1C1     D        In      -         41.504      -         
=======================================================================================================================================================
Total path delay (propagation time + setup) of 42.798 is 18.154(42.4%) logic and 24.644(57.6%) route.
Path delay compensated for clock skew. Clock skew is added to clock-to-out value, and is subtracted from setup time value


Path information for path number 5: 
      Requested Period:                      10.000
    - Setup time:                            1.295
    + Clock delay at ending point:           0.000 (ideal)
    = Required time:                         8.705

    - Propagation time:                      41.491
    - Clock delay at starting point:         0.000 (ideal)
    = Slack (non-critical) :                 -32.786

    Number of logic level(s):                11
    Starting point:                          Inst_DATA_ACQUISITION_BLOCK.Inst_RHA_TO_ZL_CONVERTER.SPIMACHINE_STATE[1] / Q
    Ending point:                            Inst_DATA_ACQUISITION_BLOCK.Inst_RHA_TO_ZL_CONVERTER.COUNTER_BITS_PROCESSED[5] / D
    The start point is clocked by            IMPLANT_TOP|Clk [rising] on pin CLK
    The end   point is clocked by            IMPLANT_TOP|Clk [rising] on pin CLK

Instance / Net                                                                                        Pin      Pin               Arrival     No. of    
Name                                                                                       Type       Name     Dir     Delay     Time        Fan Out(s)
-------------------------------------------------------------------------------------------------------------------------------------------------------
Inst_DATA_ACQUISITION_BLOCK.Inst_RHA_TO_ZL_CONVERTER.SPIMACHINE_STATE[1]                   DFN1E0     Q        Out     1.771     1.771       -         
SPIMACHINE_STATE[1]                                                                        Net        -        -       5.926     -           24        
Inst_DATA_ACQUISITION_BLOCK.Inst_RHA_TO_ZL_CONVERTER.SPIMACHINE_STATE_RNIKFP9[3]           NOR3B      A        In      -         7.697       -         
Inst_DATA_ACQUISITION_BLOCK.Inst_RHA_TO_ZL_CONVERTER.SPIMACHINE_STATE_RNIKFP9[3]           NOR3B      Y        Out     1.541     9.238       -         
spimachine_state78_1                                                                       Net        -        -       3.420     -           6         
Inst_DATA_ACQUISITION_BLOCK.Inst_RHA_TO_ZL_CONVERTER.SPIMACHINE_STATE_0_RNISSMD[0]         NOR3B      B        In      -         12.658      -         
Inst_DATA_ACQUISITION_BLOCK.Inst_RHA_TO_ZL_CONVERTER.SPIMACHINE_STATE_0_RNISSMD[0]         NOR3B      Y        Out     1.458     14.116      -         
spimachine_state78                                                                         Net        -        -       3.667     -           7         
Inst_DATA_ACQUISITION_BLOCK.Inst_RHA_TO_ZL_CONVERTER.BITMASK_SAMPLE_COPY_RNI10C32[0]       OA1A       C        In      -         17.783      -         
Inst_DATA_ACQUISITION_BLOCK.Inst_RHA_TO_ZL_CONVERTER.BITMASK_SAMPLE_COPY_RNI10C32[0]       OA1A       Y        Out     0.973     18.756      -         
COUNTER_BITS_PROCESSED_0_sqmuxa_2                                                          Net        -        -       1.938     -           3         
Inst_DATA_ACQUISITION_BLOCK.Inst_RHA_TO_ZL_CONVERTER.SPIMACHINE_STATE_3_RNIQOHDC[0]        OR3        C        In      -         20.693      -         
Inst_DATA_ACQUISITION_BLOCK.Inst_RHA_TO_ZL_CONVERTER.SPIMACHINE_STATE_3_RNIQOHDC[0]        OR3        Y        Out     1.804     22.497      -         
un1_spimachine_state72_4_1                                                                 Net        -        -       0.773     -           1         
Inst_DATA_ACQUISITION_BLOCK.Inst_RHA_TO_ZL_CONVERTER.SPIMACHINE_STATE_0_RNIVF3AE[0]        NOR2A      B        In      -         23.270      -         
Inst_DATA_ACQUISITION_BLOCK.Inst_RHA_TO_ZL_CONVERTER.SPIMACHINE_STATE_0_RNIVF3AE[0]        NOR2A      Y        Out     0.977     24.247      -         
SPIMACHINE_STATE_0_RNIVF3AE[0]                                                             Net        -        -       1.938     -           3         
Inst_DATA_ACQUISITION_BLOCK.Inst_RHA_TO_ZL_CONVERTER.un1_COUNTER_BITS_PROCESSED_1.I_1      AND2       B        In      -         26.185      -         
Inst_DATA_ACQUISITION_BLOCK.Inst_RHA_TO_ZL_CONVERTER.un1_COUNTER_BITS_PROCESSED_1.I_1      AND2       Y        Out     1.240     27.425      -         
DWACT_ADD_CI_0_TMP[0]                                                                      Net        -        -       0.927     -           2         
Inst_DATA_ACQUISITION_BLOCK.Inst_RHA_TO_ZL_CONVERTER.un1_COUNTER_BITS_PROCESSED_1.I_32     NOR2B      A        In      -         28.353      -         
Inst_DATA_ACQUISITION_BLOCK.Inst_RHA_TO_ZL_CONVERTER.un1_COUNTER_BITS_PROCESSED_1.I_32     NOR2B      Y        Out     1.174     29.526      -         
DWACT_ADD_CI_0_g_array_1[0]                                                                Net        -        -       1.938     -           3         
Inst_DATA_ACQUISITION_BLOCK.Inst_RHA_TO_ZL_CONVERTER.un1_COUNTER_BITS_PROCESSED_1.I_38     NOR2B      A        In      -         31.464      -         
Inst_DATA_ACQUISITION_BLOCK.Inst_RHA_TO_ZL_CONVERTER.un1_COUNTER_BITS_PROCESSED_1.I_38     NOR2B      Y        Out     1.174     32.637      -         
DWACT_ADD_CI_0_g_array_2[0]                                                                Net        -        -       1.938     -           3         
Inst_DATA_ACQUISITION_BLOCK.Inst_RHA_TO_ZL_CONVERTER.un1_COUNTER_BITS_PROCESSED_1.I_36     NOR2B      A        In      -         34.575      -         
Inst_DATA_ACQUISITION_BLOCK.Inst_RHA_TO_ZL_CONVERTER.un1_COUNTER_BITS_PROCESSED_1.I_36     NOR2B      Y        Out     1.174     35.749      -         
DWACT_ADD_CI_0_g_array_12_1[0]                                                             Net        -        -       0.773     -           1         
Inst_DATA_ACQUISITION_BLOCK.Inst_RHA_TO_ZL_CONVERTER.un1_COUNTER_BITS_PROCESSED_1.I_29     XOR2       B        In      -         36.521      -         
Inst_DATA_ACQUISITION_BLOCK.Inst_RHA_TO_ZL_CONVERTER.un1_COUNTER_BITS_PROCESSED_1.I_29     XOR2       Y        Out     2.251     38.772      -         
I_29                                                                                       Net        -        -       0.773     -           1         
Inst_DATA_ACQUISITION_BLOCK.Inst_RHA_TO_ZL_CONVERTER.COUNTER_BITS_PROCESSED_RNO[5]         NOR2B      A        In      -         39.545      -         
Inst_DATA_ACQUISITION_BLOCK.Inst_RHA_TO_ZL_CONVERTER.COUNTER_BITS_PROCESSED_RNO[5]         NOR2B      Y        Out     1.174     40.718      -         
COUNTER_BITS_PROCESSED_9[5]                                                                Net        -        -       0.773     -           1         
Inst_DATA_ACQUISITION_BLOCK.Inst_RHA_TO_ZL_CONVERTER.COUNTER_BITS_PROCESSED[5]             DFN1C1     D        In      -         41.491      -         
=======================================================================================================================================================
Total path delay (propagation time + setup) of 42.786 is 18.003(42.1%) logic and 24.782(57.9%) route.
Path delay compensated for clock skew. Clock skew is added to clock-to-out value, and is subtracted from setup time value


====================================
Detailed Report for Clock: Zarlink_SPI_Module|SPI_CLK_inferred_clock
====================================


Starting Points with Worst Slack
********************************

                                   Starting                                                                                              Arrival          
Instance                           Reference                                     Type       Pin     Net                                  Time        Slack
                                   Clock                                                                                                                  
----------------------------------------------------------------------------------------------------------------------------------------------------------
DEBUG_COUNTER[0]                   Zarlink_SPI_Module|SPI_CLK_inferred_clock     DFN1C1     Q       DEBUG_COUNTER[0]                     1.771       1.050
DEBUG_COUNTER[1]                   Zarlink_SPI_Module|SPI_CLK_inferred_clock     DFN1C1     Q       DEBUG_COUNTER[1]                     1.771       1.894
DEBUG_COUNTER[2]                   Zarlink_SPI_Module|SPI_CLK_inferred_clock     DFN1C1     Q       DEBUG_COUNTER[2]                     1.771       3.610
DEBUG_ZARLINK_DATA_INTERNAL[0]     Zarlink_SPI_Module|SPI_CLK_inferred_clock     DFN1C1     Q       DEBUG_ZARLINK_DATA_INTERNAL_c[0]     1.771       5.924
DEBUG_ZARLINK_DATA_INTERNAL[1]     Zarlink_SPI_Module|SPI_CLK_inferred_clock     DFN1C1     Q       DEBUG_ZARLINK_DATA_INTERNAL_c[1]     1.771       5.924
DEBUG_ZARLINK_DATA_INTERNAL[2]     Zarlink_SPI_Module|SPI_CLK_inferred_clock     DFN1C1     Q       DEBUG_ZARLINK_DATA_INTERNAL_c[2]     1.771       5.924
DEBUG_ZARLINK_DATA_INTERNAL[3]     Zarlink_SPI_Module|SPI_CLK_inferred_clock     DFN1C1     Q       DEBUG_ZARLINK_DATA_INTERNAL_c[3]     1.771       5.924
DEBUG_ZARLINK_DATA_INTERNAL[4]     Zarlink_SPI_Module|SPI_CLK_inferred_clock     DFN1C1     Q       DEBUG_ZARLINK_DATA_INTERNAL_c[4]     1.771       5.924
DEBUG_ZARLINK_DATA_INTERNAL[5]     Zarlink_SPI_Module|SPI_CLK_inferred_clock     DFN1C1     Q       DEBUG_ZARLINK_DATA_INTERNAL_c[5]     1.771       5.924
DEBUG_ZARLINK_DATA_INTERNAL[6]     Zarlink_SPI_Module|SPI_CLK_inferred_clock     DFN1C1     Q       DEBUG_ZARLINK_DATA_INTERNAL_c[6]     1.771       5.924
==========================================================================================================================================================


Ending Points with Worst Slack
******************************

                                   Starting                                                                                              Required          
Instance                           Reference                                     Type       Pin     Net                                  Time         Slack
                                   Clock                                                                                                                   
-----------------------------------------------------------------------------------------------------------------------------------------------------------
DEBUG_COUNTER[2]                   Zarlink_SPI_Module|SPI_CLK_inferred_clock     DFN1C1     D       N_8_i                                8.705        1.050
DEBUG_COUNTER[1]                   Zarlink_SPI_Module|SPI_CLK_inferred_clock     DFN1C1     D       N_7_i                                8.705        1.067
DEBUG_COUNTER[0]                   Zarlink_SPI_Module|SPI_CLK_inferred_clock     DFN1C1     D       DEBUG_COUNTER_i[0]                   8.705        2.099
DEBUG_ZARLINK_VALID_INTERNAL       Zarlink_SPI_Module|SPI_CLK_inferred_clock     DFN1C1     D       ZL_DEBUG\.un2_debug_counter          8.622        2.119
DEBUG_ZARLINK_DATA_INTERNAL[1]     Zarlink_SPI_Module|SPI_CLK_inferred_clock     DFN1C1     D       DEBUG_ZARLINK_DATA_INTERNAL_c[0]     8.622        5.924
DEBUG_ZARLINK_DATA_INTERNAL[2]     Zarlink_SPI_Module|SPI_CLK_inferred_clock     DFN1C1     D       DEBUG_ZARLINK_DATA_INTERNAL_c[1]     8.622        5.924
DEBUG_ZARLINK_DATA_INTERNAL[3]     Zarlink_SPI_Module|SPI_CLK_inferred_clock     DFN1C1     D       DEBUG_ZARLINK_DATA_INTERNAL_c[2]     8.622        5.924
DEBUG_ZARLINK_DATA_INTERNAL[4]     Zarlink_SPI_Module|SPI_CLK_inferred_clock     DFN1C1     D       DEBUG_ZARLINK_DATA_INTERNAL_c[3]     8.622        5.924
DEBUG_ZARLINK_DATA_INTERNAL[5]     Zarlink_SPI_Module|SPI_CLK_inferred_clock     DFN1C1     D       DEBUG_ZARLINK_DATA_INTERNAL_c[4]     8.622        5.924
DEBUG_ZARLINK_DATA_INTERNAL[6]     Zarlink_SPI_Module|SPI_CLK_inferred_clock     DFN1C1     D       DEBUG_ZARLINK_DATA_INTERNAL_c[5]     8.622        5.924
===========================================================================================================================================================


Worst Path Information
View Worst Path in Analyst
***********************


Path information for path number 1: 
      Requested Period:                      10.000
    - Setup time:                            1.295
    + Clock delay at ending point:           0.000 (ideal)
    = Required time:                         8.705

    - Propagation time:                      7.655
    - Clock delay at starting point:         0.000 (ideal)
    = Slack (non-critical) :                 1.050

    Number of logic level(s):                1
    Starting point:                          DEBUG_COUNTER[0] / Q
    Ending point:                            DEBUG_COUNTER[2] / D
    The start point is clocked by            Zarlink_SPI_Module|SPI_CLK_inferred_clock [rising] on pin CLK
    The end   point is clocked by            Zarlink_SPI_Module|SPI_CLK_inferred_clock [rising] on pin CLK

Instance / Net                      Pin      Pin               Arrival     No. of    
Name                     Type       Name     Dir     Delay     Time        Fan Out(s)
-------------------------------------------------------------------------------------
DEBUG_COUNTER[0]         DFN1C1     Q        Out     1.771     1.771       -         
DEBUG_COUNTER[0]         Net        -        -       2.844     -           4         
DEBUG_COUNTER_RNO[2]     AX1C       A        In      -         4.615       -         
DEBUG_COUNTER_RNO[2]     AX1C       Y        Out     2.268     6.883       -         
N_8_i                    Net        -        -       0.773     -           1         
DEBUG_COUNTER[2]         DFN1C1     D        In      -         7.655       -         
=====================================================================================
Total path delay (propagation time + setup) of 8.950 is 5.333(59.6%) logic and 3.617(40.4%) route.
Path delay compensated for clock skew. Clock skew is added to clock-to-out value, and is subtracted from setup time value


##### END OF TIMING REPORT #####]

--------------------------------------------------------------------------------
Target Part: AGL250V2_VQFP100_STD
Report for cell IMPLANT_TOP.behavioral
  Core Cell usage:
              cell count     area count*area
              AND2     9      1.0        9.0
              AND3    22      1.0       22.0
               AO1   109      1.0      109.0
              AO15     1      1.0        1.0
              AO17     1      1.0        1.0
              AO1A    83      1.0       83.0
              AO1B    23      1.0       23.0
              AO1C    13      1.0       13.0
              AO1D    20      1.0       20.0
              AOI1    19      1.0       19.0
             AOI1B    13      1.0       13.0
              AX1A     1      1.0        1.0
              AX1C    13      1.0       13.0
              AX1E     1      1.0        1.0
            CLKINT     4      0.0        0.0
               GND    30      0.0        0.0
               INV     8      1.0        8.0
               MX2   769      1.0      769.0
              MX2A    10      1.0       10.0
              MX2B     6      1.0        6.0
              MX2C     2      1.0        2.0
             NAND2     2      1.0        2.0
            NAND2A     1      1.0        1.0
              NOR2   139      1.0      139.0
             NOR2A   255      1.0      255.0
             NOR2B   325      1.0      325.0
              NOR3    39      1.0       39.0
             NOR3A    98      1.0       98.0
             NOR3B   110      1.0      110.0
             NOR3C   107      1.0      107.0
               OA1    28      1.0       28.0
              OA1A    11      1.0       11.0
              OA1B    10      1.0       10.0
              OA1C    34      1.0       34.0
              OAI1    10      1.0       10.0
               OR2   169      1.0      169.0
              OR2A   131      1.0      131.0
              OR2B    28      1.0       28.0
               OR3   104      1.0      104.0
              OR3A    19      1.0       19.0
              OR3B    10      1.0       10.0
              OR3C     5      1.0        5.0
               VCC    30      0.0        0.0
               XA1     7      1.0        7.0
              XA1A    36      1.0       36.0
              XA1B     5      1.0        5.0
              XA1C    31      1.0       31.0
              XAI1     3      1.0        3.0
             XAI1A     2      1.0        2.0
             XNOR2     6      1.0        6.0
               XO1     6      1.0        6.0
              XO1A     2      1.0        2.0
              XOR2   107      1.0      107.0
              XOR3     9      1.0        9.0


            DFN0C1   224      1.0      224.0
            DFN0E0    40      1.0       40.0
            DFN1C1   334      1.0      334.0
            DFN1E0    15      1.0       15.0
          DFN1E0C1   229      1.0      229.0
          DFN1E1C1   653      1.0      653.0
          DFN1E1P1     6      1.0        6.0
            DFN1P1    68      1.0       68.0
              DLN0     1      1.0        1.0
              DLN1     1      1.0        1.0
          FIFO4K18     8      0.0        0.0
                   -----          ----------
             TOTAL  4615              4543.0


  IO Cell usage:
              cell count
            CLKBUF     2
             INBUF    17
            OUTBUF    43
                   -----
             TOTAL    62


Core Cells         : 4543 of 6144 (74%)
IO Cells           : 62

  RAM/ROM Usage Summary
Block Rams : 8 of 8 (100%)

Mapper successful!

At Mapper Exit (Real Time elapsed 0h:00m:11s; CPU Time elapsed 0h:00m:11s; Memory used current: 58MB peak: 161MB)

Process took 0h:00m:11s realtime, 0h:00m:11s cputime
# Fri Aug 01 16:17:13 2014

###########################################################]
``` ````
