# Supplementary material for: Implications for a Wireless, External Device System to Study Electrocorticography
Source: Sensors (Basel). 2017 Apr 4;17(4):761. doi: 10.3390/s17040761 (PMC5421721; doi:10.3390/s17040761)
Supplement: Supplementary file 1 [file sensors-17-00761-s001.zip › Supplement/RHD/IGLOO_RHD_02092014.zip!/IGLOO_RHD/synthesis/syntmp/IGLOO_TOP_srr.htm]

 ```` ```
#Build: Synplify Pro I-2013.09M-SP1 , Build 034R, Jan 17 2014
#install: C:\Microsemi\Libero_v11.3\Synopsys\synplify_I201309MSP1
#OS: Windows 7 6.1
#Hostname: ITP-PC

#Implementation: synthesis

$ Start of Compile
#Tue Sep 02 15:34:21 2014

Synopsys VHDL Compiler, version comp201309rcp1, Build 078R, built Jan 14 2014
@N: :  | Running in 64-bit mode 
Copyright (C) 1994-2013 Synopsys, Inc. This software and the associated documentation are proprietary to Synopsys, Inc. This software may only be used in accordance with the terms and conditions of a written license agreement with Synopsys, Inc.  All other use or distribution of the software is strictly prohibited.

@N:CD720 : std.vhd(123) | Setting time resolution to ns
@N: : IGLOO_TOP.vhd(23) | Top entity is set to IGLOO_TOP.
File C:\Microsemi\Libero_v11.3\Synopsys\synplify_I201309MSP1\lib\proasic\igloo.vhd changed - recompiling
File C:\Users\ITP\Desktop\IGLOO\IGLOO_RHD\hdl\Zarlink_SPI_Module.vhd changed - recompiling
File C:\Users\ITP\Desktop\IGLOO\IGLOO_RHD\hdl\ZARLINK_CONNECT_INIT.vhd changed - recompiling
File C:\Users\ITP\Desktop\IGLOO\IGLOO_RHD\hdl\ORGANIZER.vhd changed - recompiling
File C:\Users\ITP\Desktop\IGLOO\IGLOO_RHD\hdl\COMMAND_RECEIVER.vhd changed - recompiling
File C:\Users\ITP\Desktop\IGLOO\IGLOO_RHD\hdl\RHA_DATA_COLLECTOR.vhd changed - recompiling
File C:\Users\ITP\Desktop\IGLOO\IGLOO_RHD\hdl\RHA_TO_ZL_CONVERTER.vhd changed - recompiling
File C:\Users\ITP\Desktop\IGLOO\IGLOO_RHD\hdl\RHA_TEST_MODULATOR.vhd changed - recompiling
File C:\Users\ITP\Desktop\IGLOO\IGLOO_RHD\hdl\CONTROL_NEXUS.vhd changed - recompiling
File C:\Users\ITP\Desktop\IGLOO\IGLOO_RHD\hdl\RHA_ARRAY.vhd changed - recompiling
File C:\Users\ITP\Desktop\IGLOO\IGLOO_RHD\hdl\RHA_TESTMODULE.vhd changed - recompiling
File C:\Users\ITP\Desktop\IGLOO\IGLOO_RHD\hdl\DATA_ACQUISITION_BLOCK.vhd changed - recompiling
File C:\Users\ITP\Desktop\IGLOO\IGLOO_RHD\hdl\IMPLANT_TOP.vhd changed - recompiling
File C:\Users\ITP\Desktop\IGLOO\IGLOO_RHD\hdl\IGLOO_TOP.vhd changed - recompiling
VHDL syntax check successful!
@N:CD630 : IGLOO_TOP.vhd(23) | Synthesizing work.igloo_top.igloo_top_arch 
@N:CD630 : IMPLANT_TOP.vhd(25) | Synthesizing work.implant_top.behavioral 
@N:CD364 : IMPLANT_TOP.vhd(544) | Removed redundant assignment
@N:CD364 : IMPLANT_TOP.vhd(545) | Removed redundant assignment
@N:CD630 : DATA_ACQUISITION_BLOCK.vhd(25) | Synthesizing work.data_acquisition_block.behavioral 
@N:CD364 : DATA_ACQUISITION_BLOCK.vhd(181) | Removed redundant assignment
@W:CG296 : DATA_ACQUISITION_BLOCK.vhd(187) | Incomplete sensitivity list - assuming completeness
@W:CG290 : DATA_ACQUISITION_BLOCK.vhd(206) | Referenced variable command_bitmask is not in sensitivity list
@W:CG290 : DATA_ACQUISITION_BLOCK.vhd(200) | Referenced variable command_samplerate is not in sensitivity list
@W:CG290 : DATA_ACQUISITION_BLOCK.vhd(204) | Referenced variable command_workmode is not in sensitivity list
@W:CG290 : DATA_ACQUISITION_BLOCK.vhd(202) | Referenced variable command_testmode is not in sensitivity list
@W:CG290 : DATA_ACQUISITION_BLOCK.vhd(196) | Referenced variable command_aux is not in sensitivity list
@W:CG290 : DATA_ACQUISITION_BLOCK.vhd(194) | Referenced variable command_channel is not in sensitivity list
@W:CG290 : DATA_ACQUISITION_BLOCK.vhd(198) | Referenced variable command_rhd_command is not in sensitivity list
@N:CD364 : DATA_ACQUISITION_BLOCK.vhd(229) | Removed redundant assignment
@N:CD364 : DATA_ACQUISITION_BLOCK.vhd(240) | Removed redundant assignment
@N:CD364 : DATA_ACQUISITION_BLOCK.vhd(251) | Removed redundant assignment
@N:CD364 : DATA_ACQUISITION_BLOCK.vhd(252) | Removed redundant assignment
@N:CD364 : DATA_ACQUISITION_BLOCK.vhd(253) | Removed redundant assignment
@N:CD364 : DATA_ACQUISITION_BLOCK.vhd(267) | Removed redundant assignment
@N:CD364 : DATA_ACQUISITION_BLOCK.vhd(277) | Removed redundant assignment
@N:CD364 : DATA_ACQUISITION_BLOCK.vhd(287) | Removed redundant assignment
@N:CD364 : DATA_ACQUISITION_BLOCK.vhd(297) | Removed redundant assignment
@N:CD630 : RHA_TESTMODULE.vhd(25) | Synthesizing work.rhd_testmodule.behavioral 
@N:CD630 : RHA_TEST_MODULATOR.vhd(26) | Synthesizing work.rhd_test_modulator.behavioral 
@N:CD231 : RHA_TEST_MODULATOR.vhd(43) | Using onehot encoding for type state_type (sm_bit00="1000000000000000")
@W:CD604 : RHA_TEST_MODULATOR.vhd(121) | OTHERS clause is not synthesized 
@N:CD364 : RHA_TEST_MODULATOR.vhd(125) | Removed redundant assignment
@N:CD364 : RHA_TEST_MODULATOR.vhd(126) | Removed redundant assignment
@W:CD604 : RHA_TEST_MODULATOR.vhd(213) | OTHERS clause is not synthesized 
@W:CD604 : RHA_TEST_MODULATOR.vhd(256) | OTHERS clause is not synthesized 
@N:CD364 : RHA_TEST_MODULATOR.vhd(273) | Removed redundant assignment
@N:CD364 : RHA_TEST_MODULATOR.vhd(274) | Removed redundant assignment
Post processing for work.rhd_test_modulator.behavioral
@W:CL265 : RHA_TEST_MODULATOR.vhd(75) | Pruning bit 0 of DATA_IN_18(15 downto 0) -- not in use ... 
Post processing for work.rhd_testmodule.behavioral
@N:CD630 : RHA_TO_ZL_CONVERTER.vhd(25) | Synthesizing work.rhd_to_zl_converter.behavioral 
@N:CD231 : RHA_TO_ZL_CONVERTER.vhd(101) | Using onehot encoding for type state_type (sm_idle="100000000000000")
@N:CD364 : RHA_TO_ZL_CONVERTER.vhd(181) | Removed redundant assignment
@N:CD364 : RHA_TO_ZL_CONVERTER.vhd(182) | Removed redundant assignment
@N:CD364 : RHA_TO_ZL_CONVERTER.vhd(223) | Removed redundant assignment
@N:CD364 : RHA_TO_ZL_CONVERTER.vhd(224) | Removed redundant assignment
@N:CD364 : RHA_TO_ZL_CONVERTER.vhd(271) | Removed redundant assignment
@N:CD364 : RHA_TO_ZL_CONVERTER.vhd(273) | Removed redundant assignment
@N:CD364 : RHA_TO_ZL_CONVERTER.vhd(274) | Removed redundant assignment
@N:CD364 : RHA_TO_ZL_CONVERTER.vhd(275) | Removed redundant assignment
@N:CD364 : RHA_TO_ZL_CONVERTER.vhd(276) | Removed redundant assignment
@N:CD364 : RHA_TO_ZL_CONVERTER.vhd(278) | Removed redundant assignment
@N:CD364 : RHA_TO_ZL_CONVERTER.vhd(285) | Removed redundant assignment
@N:CD364 : RHA_TO_ZL_CONVERTER.vhd(286) | Removed redundant assignment
@N:CD364 : RHA_TO_ZL_CONVERTER.vhd(287) | Removed redundant assignment
@N:CD364 : RHA_TO_ZL_CONVERTER.vhd(297) | Removed redundant assignment
@N:CD364 : RHA_TO_ZL_CONVERTER.vhd(305) | Removed redundant assignment
@N:CD364 : RHA_TO_ZL_CONVERTER.vhd(313) | Removed redundant assignment
@N:CD364 : RHA_TO_ZL_CONVERTER.vhd(485) | Removed redundant assignment
@W:CD604 : RHA_TO_ZL_CONVERTER.vhd(531) | OTHERS clause is not synthesized 
Post processing for work.rhd_to_zl_converter.behavioral
@A:CL282 : RHA_TO_ZL_CONVERTER.vhd(246) | Feedback mux created for signal SPIMACHINE_STATE[0:14] -- possible set/reset assignment for signal missing. Specifying a reset value will improve timing and area.
@A:CL282 : RHA_TO_ZL_CONVERTER.vhd(215) | Feedback mux created for signal NewBlock_LAST -- possible set/reset assignment for signal missing. Specifying a reset value will improve timing and area.
@A:CL282 : RHA_TO_ZL_CONVERTER.vhd(89) | Feedback mux created for signal Command_Processed_LAST -- possible set/reset assignment for signal missing. Specifying a reset value will improve timing and area.
@N:CD630 : RHA_ARRAY.vhd(30) | Synthesizing work.rhd_array.behavioral 
@N:CD233 : RHA_ARRAY.vhd(112) | Using sequential encoding for type state_type
@N:CD233 : RHA_ARRAY.vhd(123) | Using sequential encoding for type state_type_sr
@N:CD364 : RHA_ARRAY.vhd(350) | Removed redundant assignment
@N:CD364 : RHA_ARRAY.vhd(351) | Removed redundant assignment
@N:CD364 : RHA_ARRAY.vhd(352) | Removed redundant assignment
@N:CD364 : RHA_ARRAY.vhd(356) | Removed redundant assignment
@N:CD364 : RHA_ARRAY.vhd(357) | Removed redundant assignment
@N:CD364 : RHA_ARRAY.vhd(360) | Removed redundant assignment
@N:CD364 : RHA_ARRAY.vhd(361) | Removed redundant assignment
@N:CD364 : RHA_ARRAY.vhd(362) | Removed redundant assignment
@N:CD364 : RHA_ARRAY.vhd(363) | Removed redundant assignment
@N:CD364 : RHA_ARRAY.vhd(364) | Removed redundant assignment
@N:CD364 : RHA_ARRAY.vhd(365) | Removed redundant assignment
@N:CD364 : RHA_ARRAY.vhd(366) | Removed redundant assignment
@N:CD364 : RHA_ARRAY.vhd(367) | Removed redundant assignment
@N:CD364 : RHA_ARRAY.vhd(368) | Removed redundant assignment
@W:CD604 : RHA_ARRAY.vhd(467) | OTHERS clause is not synthesized 
@N:CD364 : RHA_ARRAY.vhd(494) | Removed redundant assignment
@N:CD364 : RHA_ARRAY.vhd(495) | Removed redundant assignment
@N:CD364 : RHA_ARRAY.vhd(496) | Removed redundant assignment
@W:CD604 : RHA_ARRAY.vhd(519) | OTHERS clause is not synthesized 
@N:CD364 : RHA_ARRAY.vhd(546) | Removed redundant assignment
@N:CD364 : RHA_ARRAY.vhd(547) | Removed redundant assignment
@N:CD364 : RHA_ARRAY.vhd(555) | Removed redundant assignment
@N:CD630 : RHA_DATA_COLLECTOR.vhd(31) | Synthesizing work.rhd_data_collector.behavioral 
@N:CD232 : RHA_DATA_COLLECTOR.vhd(68) | Using gray code encoding for type state_type
@N:CD364 : RHA_DATA_COLLECTOR.vhd(140) | Removed redundant assignment
@N:CD364 : RHA_DATA_COLLECTOR.vhd(141) | Removed redundant assignment
@N:CD364 : RHA_DATA_COLLECTOR.vhd(142) | Removed redundant assignment
@N:CD364 : RHA_DATA_COLLECTOR.vhd(143) | Removed redundant assignment
@N:CD364 : RHA_DATA_COLLECTOR.vhd(144) | Removed redundant assignment
@N:CD364 : RHA_DATA_COLLECTOR.vhd(145) | Removed redundant assignment
@N:CD364 : RHA_DATA_COLLECTOR.vhd(146) | Removed redundant assignment
@N:CD364 : RHA_DATA_COLLECTOR.vhd(147) | Removed redundant assignment
@N:CD364 : RHA_DATA_COLLECTOR.vhd(148) | Removed redundant assignment
@W:CD604 : RHA_DATA_COLLECTOR.vhd(246) | OTHERS clause is not synthesized 
@N:CD364 : RHA_DATA_COLLECTOR.vhd(267) | Removed redundant assignment
Post processing for work.rhd_data_collector.behavioral
@A:CL282 : RHA_DATA_COLLECTOR.vhd(118) | Feedback mux created for signal DATAIN_DATA_Copy[15:0] -- possible set/reset assignment for signal missing. Specifying a reset value will improve timing and area.
Post processing for work.rhd_array.behavioral
Post processing for work.data_acquisition_block.behavioral
@N:CD630 : CONTROL_NEXUS.vhd(26) | Synthesizing work.control_nexus.behavioral 
@W:CD638 : CONTROL_NEXUS.vhd(160) | Signal debug_processongoing_internal is undriven 
@W:CD638 : CONTROL_NEXUS.vhd(161) | Signal debug_processparameter_internal is undriven 
@N:CD630 : COMMAND_RECEIVER.vhd(109) | Synthesizing work.command_receiver.behavioral 
@N:CD232 : COMMAND_RECEIVER.vhd(209) | Using gray code encoding for type state_type
@N:CD364 : COMMAND_RECEIVER.vhd(320) | Removed redundant assignment
@N:CD364 : COMMAND_RECEIVER.vhd(321) | Removed redundant assignment
@N:CD364 : COMMAND_RECEIVER.vhd(322) | Removed redundant assignment
@N:CD364 : COMMAND_RECEIVER.vhd(323) | Removed redundant assignment
@N:CD364 : COMMAND_RECEIVER.vhd(324) | Removed redundant assignment
@N:CD364 : COMMAND_RECEIVER.vhd(325) | Removed redundant assignment
@N:CD364 : COMMAND_RECEIVER.vhd(326) | Removed redundant assignment
@N:CD364 : COMMAND_RECEIVER.vhd(327) | Removed redundant assignment
@N:CD364 : COMMAND_RECEIVER.vhd(330) | Removed redundant assignment
@N:CD364 : COMMAND_RECEIVER.vhd(331) | Removed redundant assignment
@N:CD364 : COMMAND_RECEIVER.vhd(332) | Removed redundant assignment
@N:CD364 : COMMAND_RECEIVER.vhd(333) | Removed redundant assignment
@N:CD364 : COMMAND_RECEIVER.vhd(334) | Removed redundant assignment
@N:CD364 : COMMAND_RECEIVER.vhd(335) | Removed redundant assignment
@N:CD364 : COMMAND_RECEIVER.vhd(336) | Removed redundant assignment
@N:CD364 : COMMAND_RECEIVER.vhd(337) | Removed redundant assignment
@N:CD364 : COMMAND_RECEIVER.vhd(340) | Removed redundant assignment
@N:CD364 : COMMAND_RECEIVER.vhd(343) | Removed redundant assignment
@N:CD364 : COMMAND_RECEIVER.vhd(346) | Removed redundant assignment
@N:CD364 : COMMAND_RECEIVER.vhd(349) | Removed redundant assignment
@N:CD364 : COMMAND_RECEIVER.vhd(352) | Removed redundant assignment
@N:CD364 : COMMAND_RECEIVER.vhd(353) | Removed redundant assignment
@N:CD364 : COMMAND_RECEIVER.vhd(354) | Removed redundant assignment
@N:CD364 : COMMAND_RECEIVER.vhd(355) | Removed redundant assignment
@N:CD364 : COMMAND_RECEIVER.vhd(357) | Removed redundant assignment
@N:CD364 : COMMAND_RECEIVER.vhd(358) | Removed redundant assignment
@W:CD604 : COMMAND_RECEIVER.vhd(688) | OTHERS clause is not synthesized 
Post processing for work.command_receiver.behavioral
@W:CL169 : COMMAND_RECEIVER.vhd(238) | Pruning register TEMP_WHICH_8BIT_2(7 downto 0)  
@W:CL271 : COMMAND_RECEIVER.vhd(200) | Pruning bits 7 to 3 of TEMP_WHICH_VALUE_3(7 downto 0) -- not in use ... 
@N:CD630 : ORGANIZER.vhd(26) | Synthesizing work.organizer.behavioral 
@N:CD231 : ORGANIZER.vhd(85) | Using onehot encoding for type state_type (sm_reset="100000000000000")
@N:CD364 : ORGANIZER.vhd(138) | Removed redundant assignment
Post processing for work.organizer.behavioral
@N:CD630 : ZARLINK_CONNECT_INIT.vhd(25) | Synthesizing work.zarlink_connect_init.behavioral 
@N:CD232 : ZARLINK_CONNECT_INIT.vhd(81) | Using gray code encoding for type state_type
@N:CD364 : ZARLINK_CONNECT_INIT.vhd(231) | Removed redundant assignment
@N:CD364 : ZARLINK_CONNECT_INIT.vhd(240) | Removed redundant assignment
@N:CD364 : ZARLINK_CONNECT_INIT.vhd(242) | Removed redundant assignment
@N:CD364 : ZARLINK_CONNECT_INIT.vhd(243) | Removed redundant assignment
@N:CD364 : ZARLINK_CONNECT_INIT.vhd(244) | Removed redundant assignment
@N:CD364 : ZARLINK_CONNECT_INIT.vhd(245) | Removed redundant assignment
@N:CD364 : ZARLINK_CONNECT_INIT.vhd(247) | Removed redundant assignment
@N:CD364 : ZARLINK_CONNECT_INIT.vhd(248) | Removed redundant assignment
@W:CD604 : ZARLINK_CONNECT_INIT.vhd(1019) | OTHERS clause is not synthesized 
@N:CD364 : ZARLINK_CONNECT_INIT.vhd(1031) | Removed redundant assignment
Post processing for work.zarlink_connect_init.behavioral
@N:CD630 : Zarlink_SPI_Module.vhd(6) | Synthesizing work.zarlink_spi_module.behavioral 
@N:CD232 : Zarlink_SPI_Module.vhd(46) | Using gray code encoding for type state_type
@N:CD364 : Zarlink_SPI_Module.vhd(129) | Removed redundant assignment
@N:CD364 : Zarlink_SPI_Module.vhd(135) | Removed redundant assignment
@N:CD364 : Zarlink_SPI_Module.vhd(136) | Removed redundant assignment
@N:CD364 : Zarlink_SPI_Module.vhd(137) | Removed redundant assignment
@N:CD364 : Zarlink_SPI_Module.vhd(140) | Removed redundant assignment
@W:CD604 : Zarlink_SPI_Module.vhd(394) | OTHERS clause is not synthesized 
Post processing for work.zarlink_spi_module.behavioral
Post processing for work.control_nexus.behavioral
@N:CD630 : FIFO.vhd(8) | Synthesizing work.fifo.def_arch 
@N:CD630 : igloo.vhd(2722) | Synthesizing igloo.vcc.syn_black_box 
Post processing for igloo.vcc.syn_black_box
@N:CD630 : igloo.vhd(1787) | Synthesizing igloo.gnd.syn_black_box 
Post processing for igloo.gnd.syn_black_box
@N:CD630 : igloo.vhd(2198) | Synthesizing igloo.or2.syn_black_box 
Post processing for igloo.or2.syn_black_box
@N:CD630 : igloo.vhd(1934) | Synthesizing igloo.inv.syn_black_box 
Post processing for igloo.inv.syn_black_box
@N:CD630 : igloo.vhd(3039) | Synthesizing igloo.fifo4k18.syn_black_box 
Post processing for igloo.fifo4k18.syn_black_box
@N:CD630 : igloo.vhd(13) | Synthesizing igloo.and2.syn_black_box 
Post processing for igloo.and2.syn_black_box
@N:CD630 : igloo.vhd(2032) | Synthesizing igloo.nand2.syn_black_box 
Post processing for igloo.nand2.syn_black_box
@N:CD630 : igloo.vhd(2040) | Synthesizing igloo.nand2a.syn_black_box 
Post processing for igloo.nand2a.syn_black_box
Post processing for work.fifo.def_arch
Post processing for work.implant_top.behavioral
@W:CL169 : IMPLANT_TOP.vhd(405) | Pruning register DEBUG_COUNTER_4(3 downto 0)  
@W:CL169 : IMPLANT_TOP.vhd(405) | Pruning register DEBUG_ZARLINK_VALID_INTERNAL_2  
@W:CL169 : IMPLANT_TOP.vhd(405) | Pruning register DEBUG_ZARLINK_DATA_INTERNAL_6(15 downto 0)  
@W:CL169 : IMPLANT_TOP.vhd(181) | Pruning register FIFO_EMPTY_2  
@W:CL169 : IMPLANT_TOP.vhd(180) | Pruning register FIFO_FULL_2  
@A:CL282 : IMPLANT_TOP.vhd(388) | Feedback mux created for signal FIFO_IN_READENABLE_LAST -- possible set/reset assignment for signal missing. Specifying a reset value will improve timing and area.
Post processing for work.igloo_top.igloo_top_arch
@N:CL201 : Zarlink_SPI_Module.vhd(108) | Trying to extract state machine for register FSM_STATE
Extracted state machine for register FSM_STATE
State machine has 44 reachable states with original encodings of:
   000000
   000001
   000010
   000011
   000100
   000101
   000110
   000111
   001000
   001001
   001010
   001011
   001100
   001101
   001110
   001111
   010000
   010001
   010010
   010011
   010100
   010101
   010110
   010111
   011000
   011001
   011010
   011011
   011100
   011101
   011110
   011111
   110000
   110001
   110010
   110011
   110100
   110101
   110110
   110111
   111100
   111101
   111110
   111111
@N:CL201 : ZARLINK_CONNECT_INIT.vhd(196) | Trying to extract state machine for register FSM_STATE
Extracted state machine for register FSM_STATE
State machine has 36 reachable states with original encodings of:
   000000
   000001
   000010
   000011
   000100
   000101
   000110
   000111
   001000
   001001
   001010
   001011
   001100
   001101
   001110
   001111
   010000
   010001
   010010
   010011
   010100
   010101
   010110
   010111
   011000
   011001
   011010
   011011
   011100
   011101
   011110
   011111
   110000
   110001
   110010
   110011
@N:CL201 : ORGANIZER.vhd(117) | Trying to extract state machine for register SPIMACHINE_STATE
Extracted state machine for register SPIMACHINE_STATE
State machine has 15 reachable states with original encodings of:
   000000000000001
   000000000000010
   000000000000100
   000000000001000
   000000000010000
   000000000100000
   000000001000000
   000000010000000
   000000100000000
   000001000000000
   000010000000000
   000100000000000
   001000000000000
   010000000000000
   100000000000000
@N:CL201 : COMMAND_RECEIVER.vhd(273) | Trying to extract state machine for register SPIMACHINE_STATE
Extracted state machine for register SPIMACHINE_STATE
State machine has 25 reachable states with original encodings of:
   00000
   00001
   00010
   00011
   00100
   00101
   00110
   00111
   01000
   01001
   01010
   01011
   01100
   01101
   01110
   01111
   10100
   11000
   11001
   11010
   11011
   11100
   11101
   11110
   11111
@N:CL201 : RHA_DATA_COLLECTOR.vhd(118) | Trying to extract state machine for register SPIMACHINE_STATE
Extracted state machine for register SPIMACHINE_STATE
State machine has 25 reachable states with original encodings of:
   00000
   00001
   00010
   00011
   00100
   00101
   00110
   00111
   01000
   01001
   01010
   01011
   01100
   01101
   01110
   01111
   10100
   11000
   11001
   11010
   11011
   11100
   11101
   11110
   11111
@N:CL201 : RHA_TEST_MODULATOR.vhd(169) | Trying to extract state machine for register SPIMACHINE_STATE_O
Extracted state machine for register SPIMACHINE_STATE_O
State machine has 16 reachable states with original encodings of:
   0000000000000001
   0000000000000010
   0000000000000100
   0000000000001000
   0000000000010000
   0000000000100000
   0000000001000000
   0000000010000000
   0000000100000000
   0000001000000000
   0000010000000000
   0000100000000000
   0001000000000000
   0010000000000000
   0100000000000000
   1000000000000000
@N:CL201 : RHA_TEST_MODULATOR.vhd(75) | Trying to extract state machine for register SPIMACHINE_STATE_I
Extracted state machine for register SPIMACHINE_STATE_I
State machine has 16 reachable states with original encodings of:
   0000000000000001
   0000000000000010
   0000000000000100
   0000000000001000
   0000000000010000
   0000000000100000
   0000000001000000
   0000000010000000
   0000000100000000
   0000001000000000
   0000010000000000
   0000100000000000
   0001000000000000
   0010000000000000
   0100000000000000
   1000000000000000
@W:CL159 : RHA_TEST_MODULATOR.vhd(31) | Input RHD_Clk_OUTSIDE is unused
@W:CL246 : RHA_TESTMODULE.vhd(27) | Input port bits 7 to 1 of rhd_mosi_outside(7 downto 0) are unused 
@W:CL246 : RHA_TESTMODULE.vhd(29) | Input port bits 7 to 1 of rhd_clk_outside(7 downto 0) are unused 
@W:CL246 : RHA_TESTMODULE.vhd(30) | Input port bits 7 to 1 of rhd_cs_outside(7 downto 0) are unused 
@END

At c_vhdl Exit (Real Time elapsed 0h:00m:02s; CPU Time elapsed 0h:00m:02s; Memory used current: 85MB peak: 89MB)

Process took 0h:00m:02s realtime, 0h:00m:02s cputime
# Tue Sep 02 15:34:23 2014

###########################################################]
Pre-mapping Report

Synopsys Microsemi Technology Pre-mapping, Version mapact, Build 1154R, Built Jan 20 2014 10:14:08
Copyright (C) 1994-2013, Synopsys, Inc. This software and the associated documentation are proprietary to Synopsys, Inc. This software may only be used in accordance with the terms and conditions of a written license agreement with Synopsys, Inc.  All other use or distribution of the software is strictly prohibited.
Product Version I-2013.09M-SP1 

Mapper Startup Complete (Real Time elapsed 0h:00m:00s; CPU Time elapsed 0h:00m:00s; Memory used current: 98MB peak: 99MB)

Reading constraint file: C:\Users\ITP\Desktop\IGLOO\IGLOO_RHD\constraint\IGLOO.sdc
Linked File: IGLOO_TOP_scck.rpt
Printing clock  summary report in "C:\Users\ITP\Desktop\IGLOO\IGLOO_RHD\synthesis\IGLOO_TOP_scck.rpt" file 
@W:BN544 : IGLOO.sdc(3) | create_generated_clock with both -multiply_by and -divide_by not supported for this target technology
@W:BN544 : IGLOO.sdc(4) | create_generated_clock with both -multiply_by and -divide_by not supported for this target technology
@N:MF248 :  | Running in 64-bit mode. 
@N:MF667 :  | Clock conversion disabled  

Design Input Complete (Real Time elapsed 0h:00m:00s; CPU Time elapsed 0h:00m:00s; Memory used current: 103MB peak: 104MB)


Mapper Initialization Complete (Real Time elapsed 0h:00m:00s; CPU Time elapsed 0h:00m:00s; Memory used current: 103MB peak: 104MB)

@N:BN362 : rha_data_collector.vhd(259) | Removing sequential instance NewBlock_OUT of view:PrimLib.dffr(prim) in hierarchy view:work.RHD_DATA_COLLECTOR_RHD1(behavioral) because there are no references to its outputs 
@N:BN362 : rha_data_collector.vhd(259) | Removing sequential instance Command_OUT of view:PrimLib.dffr(prim) in hierarchy view:work.RHD_DATA_COLLECTOR_RHD1(behavioral) because there are no references to its outputs 
@N:BN362 : rha_data_collector.vhd(118) | Removing sequential instance TEST_INFO_INTERNAL of view:PrimLib.dffr(prim) in hierarchy view:work.RHD_DATA_COLLECTOR_RHD1(behavioral) because there are no references to its outputs 
@N:BN362 : rha_data_collector.vhd(259) | Removing sequential instance NewBlock_OUT of view:PrimLib.dffr(prim) in hierarchy view:work.RHD_DATA_COLLECTOR_RHD2(behavioral) because there are no references to its outputs 
@N:BN362 : rha_data_collector.vhd(259) | Removing sequential instance Command_OUT of view:PrimLib.dffr(prim) in hierarchy view:work.RHD_DATA_COLLECTOR_RHD2(behavioral) because there are no references to its outputs 
@N:BN362 : rha_data_collector.vhd(118) | Removing sequential instance TEST_INFO_INTERNAL of view:PrimLib.dffr(prim) in hierarchy view:work.RHD_DATA_COLLECTOR_RHD2(behavioral) because there are no references to its outputs 
@N:BN362 : rha_data_collector.vhd(259) | Removing sequential instance NewBlock_OUT of view:PrimLib.dffr(prim) in hierarchy view:work.RHD_DATA_COLLECTOR_RHD3(behavioral) because there are no references to its outputs 
@N:BN362 : rha_data_collector.vhd(259) | Removing sequential instance Command_OUT of view:PrimLib.dffr(prim) in hierarchy view:work.RHD_DATA_COLLECTOR_RHD3(behavioral) because there are no references to its outputs 
@N:BN362 : rha_data_collector.vhd(118) | Removing sequential instance TEST_INFO_INTERNAL of view:PrimLib.dffr(prim) in hierarchy view:work.RHD_DATA_COLLECTOR_RHD3(behavioral) because there are no references to its outputs 
@N:BN362 : rha_data_collector.vhd(259) | Removing sequential instance NewBlock_OUT of view:PrimLib.dffr(prim) in hierarchy view:work.RHD_DATA_COLLECTOR_RHD4(behavioral) because there are no references to its outputs 
@N:BN362 : rha_data_collector.vhd(259) | Removing sequential instance Command_OUT of view:PrimLib.dffr(prim) in hierarchy view:work.RHD_DATA_COLLECTOR_RHD4(behavioral) because there are no references to its outputs 
@N:BN362 : rha_data_collector.vhd(118) | Removing sequential instance TEST_INFO_INTERNAL of view:PrimLib.dffr(prim) in hierarchy view:work.RHD_DATA_COLLECTOR_RHD4(behavioral) because there are no references to its outputs 
@N:BN362 : rha_data_collector.vhd(259) | Removing sequential instance NewBlock_OUT of view:PrimLib.dffr(prim) in hierarchy view:work.RHD_DATA_COLLECTOR_RHD5(behavioral) because there are no references to its outputs 
@N:BN362 : rha_data_collector.vhd(259) | Removing sequential instance Command_OUT of view:PrimLib.dffr(prim) in hierarchy view:work.RHD_DATA_COLLECTOR_RHD5(behavioral) because there are no references to its outputs 
@N:BN362 : rha_data_collector.vhd(118) | Removing sequential instance TEST_INFO_INTERNAL of view:PrimLib.dffr(prim) in hierarchy view:work.RHD_DATA_COLLECTOR_RHD5(behavioral) because there are no references to its outputs 
@N:BN362 : rha_data_collector.vhd(259) | Removing sequential instance NewBlock_OUT of view:PrimLib.dffr(prim) in hierarchy view:work.RHD_DATA_COLLECTOR_RHD6(behavioral) because there are no references to its outputs 
@N:BN362 : rha_data_collector.vhd(259) | Removing sequential instance Command_OUT of view:PrimLib.dffr(prim) in hierarchy view:work.RHD_DATA_COLLECTOR_RHD6(behavioral) because there are no references to its outputs 
@N:BN362 : rha_data_collector.vhd(118) | Removing sequential instance TEST_INFO_INTERNAL of view:PrimLib.dffr(prim) in hierarchy view:work.RHD_DATA_COLLECTOR_RHD6(behavioral) because there are no references to its outputs 
@N:BN362 : rha_data_collector.vhd(259) | Removing sequential instance NewBlock_OUT of view:PrimLib.dffr(prim) in hierarchy view:work.RHD_DATA_COLLECTOR_RHD7(behavioral) because there are no references to its outputs 
@N:BN362 : rha_data_collector.vhd(259) | Removing sequential instance Command_OUT of view:PrimLib.dffr(prim) in hierarchy view:work.RHD_DATA_COLLECTOR_RHD7(behavioral) because there are no references to its outputs 
@N:BN362 : rha_data_collector.vhd(118) | Removing sequential instance TEST_INFO_INTERNAL of view:PrimLib.dffr(prim) in hierarchy view:work.RHD_DATA_COLLECTOR_RHD7(behavioral) because there are no references to its outputs 
@N:BN362 : rha_array.vhd(325) | Removing sequential instance DATAIN_USED_EXT of view:PrimLib.dffr(prim) in hierarchy view:work.RHD_ARRAY(behavioral) because there are no references to its outputs 
@N:BN362 : rha_data_collector.vhd(118) | Removing sequential instance NewBlock_Export of view:PrimLib.dffre(prim) in hierarchy view:work.RHD_DATA_COLLECTOR_RHD1(behavioral) because there are no references to its outputs 
@N:BN362 : rha_data_collector.vhd(118) | Removing sequential instance Command_Export of view:PrimLib.dffre(prim) in hierarchy view:work.RHD_DATA_COLLECTOR_RHD1(behavioral) because there are no references to its outputs 
@N:BN362 : rha_data_collector.vhd(118) | Removing sequential instance NewBlock_Export of view:PrimLib.dffre(prim) in hierarchy view:work.RHD_DATA_COLLECTOR_RHD2(behavioral) because there are no references to its outputs 
@N:BN362 : rha_data_collector.vhd(118) | Removing sequential instance Command_Export of view:PrimLib.dffre(prim) in hierarchy view:work.RHD_DATA_COLLECTOR_RHD2(behavioral) because there are no references to its outputs 
@N:BN362 : rha_data_collector.vhd(118) | Removing sequential instance NewBlock_Export of view:PrimLib.dffre(prim) in hierarchy view:work.RHD_DATA_COLLECTOR_RHD3(behavioral) because there are no references to its outputs 
@N:BN362 : rha_data_collector.vhd(118) | Removing sequential instance Command_Export of view:PrimLib.dffre(prim) in hierarchy view:work.RHD_DATA_COLLECTOR_RHD3(behavioral) because there are no references to its outputs 
@N:BN362 : rha_data_collector.vhd(118) | Removing sequential instance NewBlock_Export of view:PrimLib.dffre(prim) in hierarchy view:work.RHD_DATA_COLLECTOR_RHD4(behavioral) because there are no references to its outputs 
@N:BN362 : rha_data_collector.vhd(118) | Removing sequential instance Command_Export of view:PrimLib.dffre(prim) in hierarchy view:work.RHD_DATA_COLLECTOR_RHD4(behavioral) because there are no references to its outputs 
@N:BN362 : rha_data_collector.vhd(118) | Removing sequential instance NewBlock_Export of view:PrimLib.dffre(prim) in hierarchy view:work.RHD_DATA_COLLECTOR_RHD5(behavioral) because there are no references to its outputs 
@N:BN362 : rha_data_collector.vhd(118) | Removing sequential instance Command_Export of view:PrimLib.dffre(prim) in hierarchy view:work.RHD_DATA_COLLECTOR_RHD5(behavioral) because there are no references to its outputs 
@N:BN362 : rha_data_collector.vhd(118) | Removing sequential instance NewBlock_Export of view:PrimLib.dffre(prim) in hierarchy view:work.RHD_DATA_COLLECTOR_RHD6(behavioral) because there are no references to its outputs 
@N:BN362 : rha_data_collector.vhd(118) | Removing sequential instance Command_Export of view:PrimLib.dffre(prim) in hierarchy view:work.RHD_DATA_COLLECTOR_RHD6(behavioral) because there are no references to its outputs 
@N:BN362 : rha_data_collector.vhd(118) | Removing sequential instance NewBlock_Export of view:PrimLib.dffre(prim) in hierarchy view:work.RHD_DATA_COLLECTOR_RHD7(behavioral) because there are no references to its outputs 
@N:BN362 : rha_data_collector.vhd(118) | Removing sequential instance Command_Export of view:PrimLib.dffre(prim) in hierarchy view:work.RHD_DATA_COLLECTOR_RHD7(behavioral) because there are no references to its outputs 
@N:BN362 : rha_data_collector.vhd(118) | Removing sequential instance NewBlock_Hold of view:PrimLib.dffre(prim) in hierarchy view:work.RHD_DATA_COLLECTOR_RHD1(behavioral) because there are no references to its outputs 
@N:BN362 : rha_data_collector.vhd(118) | Removing sequential instance Command_Hold of view:PrimLib.dffre(prim) in hierarchy view:work.RHD_DATA_COLLECTOR_RHD1(behavioral) because there are no references to its outputs 
@N:BN362 : rha_data_collector.vhd(118) | Removing sequential instance NewBlock_Hold of view:PrimLib.dffre(prim) in hierarchy view:work.RHD_DATA_COLLECTOR_RHD2(behavioral) because there are no references to its outputs 
@N:BN362 : rha_data_collector.vhd(118) | Removing sequential instance Command_Hold of view:PrimLib.dffre(prim) in hierarchy view:work.RHD_DATA_COLLECTOR_RHD2(behavioral) because there are no references to its outputs 
@N:BN362 : rha_data_collector.vhd(118) | Removing sequential instance NewBlock_Hold of view:PrimLib.dffre(prim) in hierarchy view:work.RHD_DATA_COLLECTOR_RHD3(behavioral) because there are no references to its outputs 
@N:BN362 : rha_data_collector.vhd(118) | Removing sequential instance Command_Hold of view:PrimLib.dffre(prim) in hierarchy view:work.RHD_DATA_COLLECTOR_RHD3(behavioral) because there are no references to its outputs 
@N:BN362 : rha_data_collector.vhd(118) | Removing sequential instance NewBlock_Hold of view:PrimLib.dffre(prim) in hierarchy view:work.RHD_DATA_COLLECTOR_RHD4(behavioral) because there are no references to its outputs 
@N:BN362 : rha_data_collector.vhd(118) | Removing sequential instance Command_Hold of view:PrimLib.dffre(prim) in hierarchy view:work.RHD_DATA_COLLECTOR_RHD4(behavioral) because there are no references to its outputs 
@N:BN362 : rha_data_collector.vhd(118) | Removing sequential instance NewBlock_Hold of view:PrimLib.dffre(prim) in hierarchy view:work.RHD_DATA_COLLECTOR_RHD5(behavioral) because there are no references to its outputs 
@N:BN362 : rha_data_collector.vhd(118) | Removing sequential instance Command_Hold of view:PrimLib.dffre(prim) in hierarchy view:work.RHD_DATA_COLLECTOR_RHD5(behavioral) because there are no references to its outputs 
@N:BN362 : rha_data_collector.vhd(118) | Removing sequential instance NewBlock_Hold of view:PrimLib.dffre(prim) in hierarchy view:work.RHD_DATA_COLLECTOR_RHD6(behavioral) because there are no references to its outputs 
@N:BN362 : rha_data_collector.vhd(118) | Removing sequential instance Command_Hold of view:PrimLib.dffre(prim) in hierarchy view:work.RHD_DATA_COLLECTOR_RHD6(behavioral) because there are no references to its outputs 
@N:BN362 : rha_data_collector.vhd(118) | Removing sequential instance NewBlock_Hold of view:PrimLib.dffre(prim) in hierarchy view:work.RHD_DATA_COLLECTOR_RHD7(behavioral) because there are no references to its outputs 
@N:BN362 : rha_data_collector.vhd(118) | Removing sequential instance Command_Hold of view:PrimLib.dffre(prim) in hierarchy view:work.RHD_DATA_COLLECTOR_RHD7(behavioral) because there are no references to its outputs 
@N:BN362 : rha_data_collector.vhd(118) | Removing sequential instance NewBlock_Import of view:PrimLib.dffre(prim) in hierarchy view:work.RHD_DATA_COLLECTOR_RHD1(behavioral) because there are no references to its outputs 
@N:BN362 : rha_data_collector.vhd(118) | Removing sequential instance Command_Import of view:PrimLib.dffre(prim) in hierarchy view:work.RHD_DATA_COLLECTOR_RHD1(behavioral) because there are no references to its outputs 
@N:BN362 : rha_data_collector.vhd(118) | Removing sequential instance NewBlock_Import of view:PrimLib.dffre(prim) in hierarchy view:work.RHD_DATA_COLLECTOR_RHD2(behavioral) because there are no references to its outputs 
@N:BN362 : rha_data_collector.vhd(118) | Removing sequential instance Command_Import of view:PrimLib.dffre(prim) in hierarchy view:work.RHD_DATA_COLLECTOR_RHD2(behavioral) because there are no references to its outputs 
@N:BN362 : rha_data_collector.vhd(118) | Removing sequential instance NewBlock_Import of view:PrimLib.dffre(prim) in hierarchy view:work.RHD_DATA_COLLECTOR_RHD3(behavioral) because there are no references to its outputs 
@N:BN362 : rha_data_collector.vhd(118) | Removing sequential instance Command_Import of view:PrimLib.dffre(prim) in hierarchy view:work.RHD_DATA_COLLECTOR_RHD3(behavioral) because there are no references to its outputs 
@N:BN362 : rha_data_collector.vhd(118) | Removing sequential instance NewBlock_Import of view:PrimLib.dffre(prim) in hierarchy view:work.RHD_DATA_COLLECTOR_RHD4(behavioral) because there are no references to its outputs 
@N:BN362 : rha_data_collector.vhd(118) | Removing sequential instance Command_Import of view:PrimLib.dffre(prim) in hierarchy view:work.RHD_DATA_COLLECTOR_RHD4(behavioral) because there are no references to its outputs 
@N:BN362 : rha_data_collector.vhd(118) | Removing sequential instance NewBlock_Import of view:PrimLib.dffre(prim) in hierarchy view:work.RHD_DATA_COLLECTOR_RHD5(behavioral) because there are no references to its outputs 
@N:BN362 : rha_data_collector.vhd(118) | Removing sequential instance Command_Import of view:PrimLib.dffre(prim) in hierarchy view:work.RHD_DATA_COLLECTOR_RHD5(behavioral) because there are no references to its outputs 
@N:BN362 : rha_data_collector.vhd(118) | Removing sequential instance NewBlock_Import of view:PrimLib.dffre(prim) in hierarchy view:work.RHD_DATA_COLLECTOR_RHD6(behavioral) because there are no references to its outputs 
@N:BN362 : rha_data_collector.vhd(118) | Removing sequential instance Command_Import of view:PrimLib.dffre(prim) in hierarchy view:work.RHD_DATA_COLLECTOR_RHD6(behavioral) because there are no references to its outputs 
@N:BN362 : rha_data_collector.vhd(118) | Removing sequential instance NewBlock_Import of view:PrimLib.dffre(prim) in hierarchy view:work.RHD_DATA_COLLECTOR_RHD7(behavioral) because there are no references to its outputs 
@N:BN362 : rha_data_collector.vhd(118) | Removing sequential instance Command_Import of view:PrimLib.dffre(prim) in hierarchy view:work.RHD_DATA_COLLECTOR_RHD7(behavioral) because there are no references to its outputs 


Clock Summary
**************

Start             Requested     Requested     Clock        Clock           
Clock             Frequency     Period        Type         Group           
---------------------------------------------------------------------------
IGLOO_TOP|Clk     24.0 MHz      41.666        declared     default_clkgroup
===========================================================================

@W:MT532 : fifo.vhd(443) | Found signal identified as System clock which controls 2079 sequential elements including MYImplant.MyFIFO.\\FIFOBLOCK\[1\]\\.  Using this clock, which has no specified timing constraint, can adversely impact design performance. 

Finished Pre Mapping Phase.
@N:BN225 :  | Writing default property annotation file C:\Users\ITP\Desktop\IGLOO\IGLOO_RHD\synthesis\IGLOO_TOP.sap. 
Pre-mapping successful!

At Mapper Exit (Real Time elapsed 0h:00m:00s; CPU Time elapsed 0h:00m:00s; Memory used current: 49MB peak: 113MB)

Process took 0h:00m:01s realtime, 0h:00m:01s cputime
# Tue Sep 02 15:34:27 2014

###########################################################]
Map & Optimize Report

Synopsys Microsemi Technology Mapper, Version mapact, Build 1154R, Built Jan 20 2014 10:14:08
Copyright (C) 1994-2013, Synopsys, Inc. This software and the associated documentation are proprietary to Synopsys, Inc. This software may only be used in accordance with the terms and conditions of a written license agreement with Synopsys, Inc.  All other use or distribution of the software is strictly prohibited.
Product Version I-2013.09M-SP1 

Mapper Startup Complete (Real Time elapsed 0h:00m:00s; CPU Time elapsed 0h:00m:00s; Memory used current: 99MB peak: 99MB)

@N:MF248 :  | Running in 64-bit mode. 
@N:MF667 :  | Clock conversion disabled  

Design Input Complete (Real Time elapsed 0h:00m:00s; CPU Time elapsed 0h:00m:00s; Memory used current: 99MB peak: 100MB)


Mapper Initialization Complete (Real Time elapsed 0h:00m:00s; CPU Time elapsed 0h:00m:00s; Memory used current: 99MB peak: 100MB)

@W:BN544 : igloo.sdc(3) | create_generated_clock with both -multiply_by and -divide_by not supported for this target technology
@W:BN544 : igloo.sdc(4) | create_generated_clock with both -multiply_by and -divide_by not supported for this target technology


Starting Optimization and Mapping (Real Time elapsed 0h:00m:00s; CPU Time elapsed 0h:00m:00s; Memory used current: 111MB peak: 112MB)


Available hyper_sources - for debug and ip models
	None Found


Finished RTL optimizations (Real Time elapsed 0h:00m:00s; CPU Time elapsed 0h:00m:00s; Memory used current: 112MB peak: 114MB)

@N: : zarlink_spi_module.vhd(108) | Found counter in view:work.Zarlink_SPI_Module(behavioral) inst Counter[3:0]
Encoding state machine FSM_STATE[0:43] (view:work.Zarlink_SPI_Module(behavioral))
original code -> new code
   000000 -> 000000
   000001 -> 000001
   000010 -> 000011
   000011 -> 000010
   000100 -> 000110
   000101 -> 000111
   000110 -> 000101
   000111 -> 000100
   001000 -> 001100
   001001 -> 001101
   001010 -> 001111
   001011 -> 001110
   001100 -> 001010
   001101 -> 001011
   001110 -> 001001
   001111 -> 001000
   010000 -> 011000
   010001 -> 011001
   010010 -> 011011
   010011 -> 011010
   010100 -> 011110
   010101 -> 011111
   010110 -> 011101
   010111 -> 011100
   011000 -> 010100
   011001 -> 010101
   011010 -> 010111
   011011 -> 010110
   011100 -> 010010
   011101 -> 010011
   011110 -> 010001
   011111 -> 010000
   110000 -> 110000
   110001 -> 110001
   110010 -> 110011
   110011 -> 110010
   110100 -> 110110
   110101 -> 110111
   110110 -> 110101
   110111 -> 110100
   111100 -> 111100
   111101 -> 111101
   111110 -> 111111
   111111 -> 111110
@N: : zarlink_connect_init.vhd(196) | Found counter in view:work.ZARLINK_CONNECT_INIT(behavioral) inst Zarlink_ConnectionTimeoutCounter[31:0]
@N: : zarlink_connect_init.vhd(196) | Found counter in view:work.ZARLINK_CONNECT_INIT(behavioral) inst Zarlink_ResetCounter[23:0]
@N: : zarlink_connect_init.vhd(196) | Found counter in view:work.ZARLINK_CONNECT_INIT(behavioral) inst Zarlink_Retry_Counter[16:0]
Encoding state machine FSM_STATE[0:35] (view:work.ZARLINK_CONNECT_INIT(behavioral))
original code -> new code
   000000 -> 000000
   000001 -> 000001
   000010 -> 000011
   000011 -> 000010
   000100 -> 000110
   000101 -> 000111
   000110 -> 000101
   000111 -> 000100
   001000 -> 001100
   001001 -> 001101
   001010 -> 001111
   001011 -> 001110
   001100 -> 001010
   001101 -> 001011
   001110 -> 001001
   001111 -> 001000
   010000 -> 011000
   010001 -> 011001
   010010 -> 011011
   010011 -> 011010
   010100 -> 011110
   010101 -> 011111
   010110 -> 011101
   010111 -> 011100
   011000 -> 010100
   011001 -> 010101
   011010 -> 010111
   011011 -> 010110
   011100 -> 010010
   011101 -> 010011
   011110 -> 010001
   011111 -> 010000
   110000 -> 110000
   110001 -> 110001
   110010 -> 110011
   110011 -> 110010
@N: : organizer.vhd(117) | Found counter in view:work.ORGANIZER(behavioral) inst WaitForXCyles_Counter[17:0]
@N: : organizer.vhd(117) | Found counter in view:work.ORGANIZER(behavioral) inst CheckOutgoingBuffer_SIZE_COPY[6:0]
Encoding state machine SPIMACHINE_STATE[0:14] (view:work.ORGANIZER(behavioral))
original code -> new code
   000000000000001 -> 000000000000001
   000000000000010 -> 000000000000010
   000000000000100 -> 000000000000100
   000000000001000 -> 000000000001000
   000000000010000 -> 000000000010000
   000000000100000 -> 000000000100000
   000000001000000 -> 000000001000000
   000000010000000 -> 000000010000000
   000000100000000 -> 000000100000000
   000001000000000 -> 000001000000000
   000010000000000 -> 000010000000000
   000100000000000 -> 000100000000000
   001000000000000 -> 001000000000000
   010000000000000 -> 010000000000000
   100000000000000 -> 100000000000000
Encoding state machine SPIMACHINE_STATE[0:24] (view:work.COMMAND_RECEIVER(behavioral))
original code -> new code
   00000 -> 00000
   00001 -> 00001
   00010 -> 00011
   00011 -> 00010
   00100 -> 00110
   00101 -> 00111
   00110 -> 00101
   00111 -> 00100
   01000 -> 01100
   01001 -> 01101
   01010 -> 01111
   01011 -> 01110
   01100 -> 01010
   01101 -> 01011
   01110 -> 01001
   01111 -> 01000
   10100 -> 11000
   11000 -> 11001
   11001 -> 11011
   11010 -> 11010
   11011 -> 11110
   11100 -> 11111
   11101 -> 11101
   11110 -> 11100
   11111 -> 10100
@N: : rha_array.vhd(325) | Found counter in view:work.RHD_ARRAY(behavioral) inst Channel_Counter[5:0]
@N: : rha_array.vhd(482) | Found counter in view:work.RHD_ARRAY(behavioral) inst SampleRate_Counter[31:0]
@N:MF238 : rha_array.vhd(505) | Found 5-bit incrementor, 'un2_samplerate_smallcounter_1[4:0]'
Encoding state machine SPIMACHINE_STATE[0:24] (view:work.RHD_DATA_COLLECTOR_RHD0(behavioral))
original code -> new code
   00000 -> 00000
   00001 -> 00001
   00010 -> 00011
   00011 -> 00010
   00100 -> 00110
   00101 -> 00111
   00110 -> 00101
   00111 -> 00100
   01000 -> 01100
   01001 -> 01101
   01010 -> 01111
   01011 -> 01110
   01100 -> 01010
   01101 -> 01011
   01110 -> 01001
   01111 -> 01000
   10100 -> 11000
   11000 -> 11001
   11001 -> 11011
   11010 -> 11010
   11011 -> 11110
   11100 -> 11111
   11101 -> 11101
   11110 -> 11100
   11111 -> 10100
Encoding state machine SPIMACHINE_STATE[0:24] (view:work.RHD_DATA_COLLECTOR(behavioral))
original code -> new code
   00000 -> 00000
   00001 -> 00001
   00010 -> 00011
   00011 -> 00010
   00100 -> 00110
   00101 -> 00111
   00110 -> 00101
   00111 -> 00100
   01000 -> 01100
   01001 -> 01101
   01010 -> 01111
   01011 -> 01110
   01100 -> 01010
   01101 -> 01011
   01110 -> 01001
   01111 -> 01000
   10100 -> 11000
   11000 -> 11001
   11001 -> 11011
   11010 -> 11010
   11011 -> 11110
   11100 -> 11111
   11101 -> 11101
   11110 -> 11100
   11111 -> 10100
@N: : rha_to_zl_converter.vhd(215) | Found counter in view:work.RHD_TO_ZL_CONVERTER(behavioral) inst CMDRESPONSE_COUNTER[15:0]
@N: : rha_to_zl_converter.vhd(215) | Found counter in view:work.RHD_TO_ZL_CONVERTER(behavioral) inst Timestamp[15:0]
@N:MF176 :  | Default generator successful  
@N:MF176 :  | Default generator successful  
Encoding state machine SPIMACHINE_STATE_O[0:15] (view:work.RHD_TEST_MODULATOR(behavioral))
original code -> new code
   0000000000000001 -> 0000000000000001
   0000000000000010 -> 0000000000000010
   0000000000000100 -> 0000000000000100
   0000000000001000 -> 0000000000001000
   0000000000010000 -> 0000000000010000
   0000000000100000 -> 0000000000100000
   0000000001000000 -> 0000000001000000
   0000000010000000 -> 0000000010000000
   0000000100000000 -> 0000000100000000
   0000001000000000 -> 0000001000000000
   0000010000000000 -> 0000010000000000
   0000100000000000 -> 0000100000000000
   0001000000000000 -> 0001000000000000
   0010000000000000 -> 0010000000000000
   0100000000000000 -> 0100000000000000
   1000000000000000 -> 1000000000000000
Encoding state machine SPIMACHINE_STATE_I[0:15] (view:work.RHD_TEST_MODULATOR(behavioral))
original code -> new code
   0000000000000001 -> 0000000000000001
   0000000000000010 -> 0000000000000010
   0000000000000100 -> 0000000000000100
   0000000000001000 -> 0000000000001000
   0000000000010000 -> 0000000000010000
   0000000000100000 -> 0000000000100000
   0000000001000000 -> 0000000001000000
   0000000010000000 -> 0000000010000000
   0000000100000000 -> 0000000100000000
   0000001000000000 -> 0000001000000000
   0000010000000000 -> 0000010000000000
   0000100000000000 -> 0000100000000000
   0001000000000000 -> 0001000000000000
   0010000000000000 -> 0010000000000000
   0100000000000000 -> 0100000000000000
   1000000000000000 -> 1000000000000000

Finished factoring (Real Time elapsed 0h:00m:03s; CPU Time elapsed 0h:00m:02s; Memory used current: 138MB peak: 139MB)


Finished gated-clock and generated-clock conversion (Real Time elapsed 0h:00m:03s; CPU Time elapsed 0h:00m:02s; Memory used current: 133MB peak: 139MB)


Finished generic timing optimizations - Pass 1 (Real Time elapsed 0h:00m:05s; CPU Time elapsed 0h:00m:04s; Memory used current: 139MB peak: 146MB)


Starting Early Timing Optimization (Real Time elapsed 0h:00m:05s; CPU Time elapsed 0h:00m:04s; Memory used current: 141MB peak: 146MB)


Finished Early Timing Optimization (Real Time elapsed 0h:00m:16s; CPU Time elapsed 0h:00m:14s; Memory used current: 153MB peak: 154MB)


Finished generic timing optimizations - Pass 2 (Real Time elapsed 0h:00m:16s; CPU Time elapsed 0h:00m:15s; Memory used current: 151MB peak: 154MB)


Finished preparing to map (Real Time elapsed 0h:00m:16s; CPU Time elapsed 0h:00m:15s; Memory used current: 149MB peak: 154MB)


Finished technology mapping (Real Time elapsed 0h:00m:17s; CPU Time elapsed 0h:00m:16s; Memory used current: 191MB peak: 195MB)


High Fanout Net Report
**********************

Driver Instance / Pin Name                                                                                            Fanout, notes                     
--------------------------------------------------------------------------------------------------------------------------------------------------------
MYImplant.Inst_CONTROL_NEXUS.Inst_COMMAND_RECEIVER.TestMode_INTERAL / Q                                               56                                
Reset_pad / Y                                                                                                         1727 : 1689 asynchronous set/reset
MYImplant.Inst_DATA_ACQUISITION_BLOCK.Inst_RHD_TESTMODULE.RHD_TEST_MOD_0.SPIMACHINE_STATE_I[15] / Q                   32                                
MYImplant.Inst_DATA_ACQUISITION_BLOCK.Inst_RHD_TO_ZL_CONVERTER.SPIMACHINE_STATE[3] / Q                                54                                
MYImplant.Inst_DATA_ACQUISITION_BLOCK.Inst_RHD_ARRAY.SPIMACHINE_STATE[0] / Q                                          324                               
MYImplant.Inst_DATA_ACQUISITION_BLOCK.Inst_RHD_ARRAY.SPIMACHINE_STATE_SR[0] / Q                                       57                                
MYImplant.Inst_DATA_ACQUISITION_BLOCK.Inst_RHD_ARRAY.SampleRate_Counter_e1_i_a2_0 / Y                                 29                                
MYImplant.Inst_CONTROL_NEXUS.Inst_COMMAND_RECEIVER.SPIMACHINE_STATE[2] / Q                                            36                                
MYImplant.Inst_CONTROL_NEXUS.Inst_COMMAND_RECEIVER.SPIMACHINE_STATE[4] / Q                                            34                                
MYImplant.Inst_CONTROL_NEXUS.Inst_ZARLINK_CONNECT_INIT.FSM_STATE[1] / Q                                               29                                
MYImplant.Inst_CONTROL_NEXUS.Inst_ZARLINK_CONNECT_INIT.FSM_STATE[2] / Q                                               27                                
MYImplant.Inst_CONTROL_NEXUS.Inst_ZARLINK_CONNECT_INIT.FSM_STATE[3] / Q                                               27                                
MYImplant.Inst_CONTROL_NEXUS.Inst_Zarlink_SPI_Module.FSM_STATE[3] / Q                                                 25                                
MYImplant.Inst_CONTROL_NEXUS.Inst_Zarlink_SPI_Module.FSM_STATE[4] / Q                                                 25                                
MYImplant.Inst_CONTROL_NEXUS.Inst_COMMAND_RECEIVER.CMD_RECEIVER.ChannelMask_Temp_13_0_iv_0_i_0_o5[9] / Y              32                                
MYImplant.Inst_CONTROL_NEXUS.Inst_COMMAND_RECEIVER.N_1357_i_i_o2_i_a2_0_a2 / Y                                        33                                
MYImplant.Inst_CONTROL_NEXUS.Inst_COMMAND_RECEIVER.ChannelMask_Changed_2_sqmuxa_0_a2_1_a2_1_a5 / Y                    32                                
MYImplant.Inst_CONTROL_NEXUS.Inst_COMMAND_RECEIVER.CMD_RECEIVER.SampleRate_INTERNAL_13_0_iv_i_0_0_o2[22] / Y          27                                
MYImplant.Inst_CONTROL_NEXUS.Inst_COMMAND_RECEIVER.ChannelMask_Changed_6_sqmuxa_0_a2_0_a2_0_a5 / Y                    32                                
MYImplant.Inst_DATA_ACQUISITION_BLOCK.Inst_RHD_ARRAY.COPY_VALID_DATA.dataout_valid / Y                                154                               
MYImplant.Inst_DATA_ACQUISITION_BLOCK.Inst_RHD_ARRAY.COPY_VALID_DATA.un1_readrequest / Y                              136                               
MYImplant.Inst_CONTROL_NEXUS.Inst_COMMAND_RECEIVER.CMD_RECEIVER.un1_fifo_out_valid / Y                                30                                
MYImplant.Inst_DATA_ACQUISITION_BLOCK.Inst_RHD_TESTMODULE.RHD_TEST_MOD_0.RHD_MISO_OUTSIDE_iv / Y                      49                                
MYImplant.Inst_CONTROL_NEXUS.Inst_COMMAND_RECEIVER.ChannelMask_Changed_0_sqmuxa_0_a2_2_a2_2_a5 / Y                    32                                
MYImplant.Inst_CONTROL_NEXUS.Inst_COMMAND_RECEIVER.ChannelMask_Changed_1_sqmuxa_0_a2_1_a2_1_a5 / Y                    32                                
MYImplant.Inst_CONTROL_NEXUS.Inst_COMMAND_RECEIVER.ChannelMask_Changed_3_sqmuxa_0_a2_1_a2_1_a5 / Y                    32                                
MYImplant.Inst_CONTROL_NEXUS.Inst_COMMAND_RECEIVER.ChannelMask_Changed_4_sqmuxa_0_a2_1_a2_1_a5 / Y                    32                                
MYImplant.Inst_CONTROL_NEXUS.Inst_COMMAND_RECEIVER.ChannelMask_Changed_5_sqmuxa_0_a2_0_a2_0_a5 / Y                    32                                
MYImplant.Inst_DATA_ACQUISITION_BLOCK.Inst_RHD_TESTMODULE.RHD_TEST_MOD_0.CS_RISING_PROCESS.un1_rhd_cs_outside / Y     32                                
MYImplant.Inst_CONTROL_NEXUS.Inst_ZARLINK_CONNECT_INIT.Zarlink_ResetCounter_n0_0_a4_i_o2_0_o3 / Y                     26                                
MYImplant.Inst_CONTROL_NEXUS.Inst_ZARLINK_CONNECT_INIT.FSM.fsm_state155_i_i_a2_i / Y                                  32                                
MYImplant.Inst_DATA_ACQUISITION_BLOCK.Inst_RHD_TO_ZL_CONVERTER.un1_Command_Processed_Off_2_sqmuxa / Y                 49                                
MYImplant.Inst_CONTROL_NEXUS.Inst_COMMAND_RECEIVER.ChannelMask_Changed_7_sqmuxa_0_a2_0_a2_0_a5 / Y                    32                                
MYImplant.Inst_DATA_ACQUISITION_BLOCK.Inst_RHD_ARRAY.Channel_m1_0_a2 / Y                                              286                               
========================================================================================================================================================

@N:FP130 :  | Promoting Net Reset_c on CLKBUF  Reset_pad  
@N:FP130 :  | Promoting Net MYImplant.Clk on CLKINT  MYImplant.Clk_inferred_clock  
@N:FP130 :  | Promoting Net MYImplant.Inst_DATA_ACQUISITION_BLOCK.Inst_RHD_ARRAY.SPIMACHINE_STATE[0] on CLKINT  I_542  
@N:FP130 :  | Promoting Net MYImplant.Inst_DATA_ACQUISITION_BLOCK.Inst_RHD_ARRAY.Channel_N_3_mux on CLKINT  I_543  
@N:FP130 :  | Promoting Net MYImplant.Inst_DATA_ACQUISITION_BLOCK.Inst_RHD_ARRAY.COPY_VALID_DATA\.dataout_valid on CLKINT  I_544  
@N:FP130 :  | Promoting Net MYImplant.Inst_DATA_ACQUISITION_BLOCK.Inst_RHD_ARRAY.COPY_VALID_DATA\.un1_readrequest on CLKINT  I_545  

Finished technology timing optimizations and critical path resynthesis (Real Time elapsed 0h:00m:18s; CPU Time elapsed 0h:00m:16s; Memory used current: 191MB peak: 195MB)

Replicating Combinational Instance MYImplant.Inst_CONTROL_NEXUS.Inst_COMMAND_RECEIVER.ChannelMask_Changed_7_sqmuxa_0_a2_0_a2_0_a5, fanout 32 segments 2
Replicating Combinational Instance MYImplant.Inst_DATA_ACQUISITION_BLOCK.Inst_RHD_TO_ZL_CONVERTER.un1_Command_Processed_Off_2_sqmuxa, fanout 49 segments 3
Replicating Combinational Instance MYImplant.Inst_CONTROL_NEXUS.Inst_ZARLINK_CONNECT_INIT.FSM.fsm_state155_i_i_a2_i, fanout 32 segments 2
Replicating Combinational Instance MYImplant.Inst_CONTROL_NEXUS.Inst_ZARLINK_CONNECT_INIT.Zarlink_ResetCounter_n0_0_a4_i_o2_0_o3, fanout 26 segments 2
Replicating Combinational Instance MYImplant.Inst_DATA_ACQUISITION_BLOCK.Inst_RHD_TESTMODULE.RHD_TEST_MOD_0.CS_RISING_PROCESS.un1_rhd_cs_outside, fanout 32 segments 2
Replicating Combinational Instance MYImplant.Inst_CONTROL_NEXUS.Inst_COMMAND_RECEIVER.ChannelMask_Changed_5_sqmuxa_0_a2_0_a2_0_a5, fanout 32 segments 2
Replicating Combinational Instance MYImplant.Inst_CONTROL_NEXUS.Inst_COMMAND_RECEIVER.ChannelMask_Changed_4_sqmuxa_0_a2_1_a2_1_a5, fanout 32 segments 2
Replicating Combinational Instance MYImplant.Inst_CONTROL_NEXUS.Inst_COMMAND_RECEIVER.ChannelMask_Changed_3_sqmuxa_0_a2_1_a2_1_a5, fanout 32 segments 2
Replicating Combinational Instance MYImplant.Inst_CONTROL_NEXUS.Inst_COMMAND_RECEIVER.ChannelMask_Changed_1_sqmuxa_0_a2_1_a2_1_a5, fanout 32 segments 2
Replicating Combinational Instance MYImplant.Inst_CONTROL_NEXUS.Inst_COMMAND_RECEIVER.ChannelMask_Changed_0_sqmuxa_0_a2_2_a2_2_a5, fanout 32 segments 2
Replicating Combinational Instance MYImplant.Inst_DATA_ACQUISITION_BLOCK.Inst_RHD_TESTMODULE.RHD_TEST_MOD_0.RHD_MISO_OUTSIDE_iv, fanout 49 segments 3
Replicating Combinational Instance MYImplant.Inst_CONTROL_NEXUS.Inst_COMMAND_RECEIVER.CMD_RECEIVER.un1_fifo_out_valid, fanout 30 segments 2
Replicating Combinational Instance MYImplant.Inst_CONTROL_NEXUS.Inst_COMMAND_RECEIVER.ChannelMask_Changed_6_sqmuxa_0_a2_0_a2_0_a5, fanout 32 segments 2
Replicating Combinational Instance MYImplant.Inst_CONTROL_NEXUS.Inst_COMMAND_RECEIVER.CMD_RECEIVER.SampleRate_INTERNAL_13_0_iv_i_0_0_o2[22], fanout 27 segments 2
Replicating Combinational Instance MYImplant.Inst_CONTROL_NEXUS.Inst_COMMAND_RECEIVER.ChannelMask_Changed_2_sqmuxa_0_a2_1_a2_1_a5, fanout 32 segments 2
Replicating Combinational Instance MYImplant.Inst_CONTROL_NEXUS.Inst_COMMAND_RECEIVER.N_1357_i_i_o2_i_a2_0_a2, fanout 33 segments 2
Replicating Combinational Instance MYImplant.Inst_CONTROL_NEXUS.Inst_COMMAND_RECEIVER.CMD_RECEIVER.ChannelMask_Temp_13_0_iv_0_i_0_o5[9], fanout 32 segments 2
Replicating Sequential Instance MYImplant.Inst_CONTROL_NEXUS.Inst_Zarlink_SPI_Module.FSM_STATE[4], fanout 25 segments 2
Replicating Sequential Instance MYImplant.Inst_CONTROL_NEXUS.Inst_Zarlink_SPI_Module.FSM_STATE[3], fanout 25 segments 2
Replicating Sequential Instance MYImplant.Inst_CONTROL_NEXUS.Inst_ZARLINK_CONNECT_INIT.FSM_STATE[3], fanout 27 segments 2
Replicating Sequential Instance MYImplant.Inst_CONTROL_NEXUS.Inst_ZARLINK_CONNECT_INIT.FSM_STATE[2], fanout 27 segments 2
Replicating Sequential Instance MYImplant.Inst_CONTROL_NEXUS.Inst_ZARLINK_CONNECT_INIT.FSM_STATE[1], fanout 30 segments 2
Replicating Sequential Instance MYImplant.Inst_CONTROL_NEXUS.Inst_COMMAND_RECEIVER.SPIMACHINE_STATE[4], fanout 35 segments 2
Replicating Sequential Instance MYImplant.Inst_CONTROL_NEXUS.Inst_COMMAND_RECEIVER.SPIMACHINE_STATE[2], fanout 36 segments 2
Replicating Combinational Instance MYImplant.Inst_DATA_ACQUISITION_BLOCK.Inst_RHD_ARRAY.SampleRate_Counter_e1_i_a2_0, fanout 29 segments 2
Replicating Sequential Instance MYImplant.Inst_DATA_ACQUISITION_BLOCK.Inst_RHD_ARRAY.SPIMACHINE_STATE_SR[0], fanout 58 segments 3
Replicating Sequential Instance MYImplant.Inst_DATA_ACQUISITION_BLOCK.Inst_RHD_TO_ZL_CONVERTER.SPIMACHINE_STATE[3], fanout 54 segments 3
Replicating Sequential Instance MYImplant.Inst_DATA_ACQUISITION_BLOCK.Inst_RHD_TESTMODULE.RHD_TEST_MOD_0.SPIMACHINE_STATE_I[15], fanout 32 segments 2
Replicating Sequential Instance MYImplant.Inst_CONTROL_NEXUS.Inst_COMMAND_RECEIVER.TestMode_INTERAL, fanout 56 segments 3
Replicating Sequential Instance MYImplant.Inst_CONTROL_NEXUS.Inst_ZARLINK_CONNECT_INIT.FSM_STATE[0], fanout 25 segments 2
Replicating Sequential Instance MYImplant.Inst_CONTROL_NEXUS.Inst_COMMAND_RECEIVER.TEMP_WHICH_RHD[1], fanout 25 segments 2
Replicating Sequential Instance MYImplant.Inst_CONTROL_NEXUS.Inst_COMMAND_RECEIVER.TEMP_WHICH_RHD[0], fanout 25 segments 2

Added 0 Buffers
Added 37 Cells via replication
	Added 17 Sequential Cells via replication
	Added 20 Combinational Cells via replication

Finished restoring hierarchy (Real Time elapsed 0h:00m:23s; CPU Time elapsed 0h:00m:21s; Memory used current: 192MB peak: 195MB)


#### START OF CLOCK OPTIMIZATION REPORT #####[

Clock optimization not enabled
1 non-gated/non-generated clock tree(s) driving 4 clock pin(s) of sequential element(s)
2 gated/generated clock tree(s) driving 1864 clock pin(s) of sequential element(s)
0 instances converted, 1864 sequential instances remain driven by gated/generated clocks

================================ Non-Gated/Non-Generated Clocks =================================
Clock Tree ID     Driving Element     Drive Element Type     Fanout     Sample Instance          
-------------------------------------------------------------------------------------------------
ClockId0003        Clk                 port                   4          MYImplant.Counter_4MHz[1]
=================================================================================================
====================================================================================================== Gated/Generated Clocks =======================================================================================================
Clock Tree ID     Driving Element          Drive Element Type     Fanout     Sample Instance                                                                 Explanation                                                             
-------------------------------------------------------------------------------------------------------------------------------------------------------------------------------------------------------------------------------------
ClockId0001        MYImplant.Clk            DFN1C0                 1821       MYImplant.FIFO_AEMPTY                                                           No generated or derived clock directive on output of sequential instance
ClockId0002        MYImplant.Clock_4MHz     DFN1C0                 43         MYImplant.Inst_CONTROL_NEXUS.Inst_Zarlink_SPI_Module.Zarlink_Adress_Copy[7]     No generated or derived clock directive on output of sequential instance
=====================================================================================================================================================================================================================================


##### END OF CLOCK OPTIMIZATION REPORT ######]

Writing Analyst data base C:\Users\ITP\Desktop\IGLOO\IGLOO_RHD\synthesis\IGLOO_TOP.srm

Finished Writing Netlist Databases (Real Time elapsed 0h:00m:23s; CPU Time elapsed 0h:00m:22s; Memory used current: 184MB peak: 195MB)

Writing EDIF Netlist and constraint files
I-2013.09M-SP1 

Finished Writing EDIF Netlist and constraint files (Real Time elapsed 0h:00m:24s; CPU Time elapsed 0h:00m:23s; Memory used current: 186MB peak: 195MB)

Found clock IGLOO_TOP|Clk with period 41.67ns 
@W:MT420 :  | Found inferred clock IMPLANT_TOP|Clk_inferred_clock with period 10.00ns. Please declare a user-defined clock on object "n:MYImplant.Clk" 

@W:MT420 :  | Found inferred clock IMPLANT_TOP|Clock_4MHz_inferred_clock with period 10.00ns. Please declare a user-defined clock on object "n:MYImplant.Clock_4MHz" 


##### START OF TIMING REPORT #####[
# Timing Report written on Tue Sep 02 15:34:51 2014
#


Top view:               IGLOO_TOP
Library name:           IGLOO_V2
Operating conditions:   COMWCSTD ( T = 70.0, V = 1.14, P = 3.70, tree_type = balanced_tree )
Requested Frequency:    24.0 MHz
Wire load mode:         top
Wire load model:        igloo
Paths requested:        5
Constraint File(s):    C:\Users\ITP\Desktop\IGLOO\IGLOO_RHD\constraint\IGLOO.sdc
                       
@N:MT320 :  | Timing report estimates place and route data. Please look at the place and route timing report for final timing. 

@N:MT322 :  | Clock constraints cover only FF-to-FF paths associated with the clock. 


Performance Summary 
*******************


Worst slack in design: -27.896

                                          Requested     Estimated     Requested     Estimated                 Clock        Clock              
Starting Clock                            Frequency     Frequency     Period        Period        Slack       Type         Group              
----------------------------------------------------------------------------------------------------------------------------------------------
IGLOO_TOP|Clk                             24.0 MHz      81.3 MHz      41.666        12.295        29.371      declared     default_clkgroup   
IMPLANT_TOP|Clk_inferred_clock            100.0 MHz     15.2 MHz      10.000        65.793        -27.896     inferred     Inferred_clkgroup_0
IMPLANT_TOP|Clock_4MHz_inferred_clock     100.0 MHz     36.0 MHz      10.000        27.743        -17.743     inferred     Inferred_clkgroup_1
==============================================================================================================================================
@W:MT548 : igloo.sdc(3) | Source for clock MYImplant/Clk:Q not found in netlist
@W:MT548 : igloo.sdc(4) | Source for clock MYImplant/Clock_4MHz:Q not found in netlist


Clock Relationships
*******************

Clocks                                                                        |    rise  to  rise     |    fall  to  fall     |    rise  to  fall     |    fall  to  rise   
----------------------------------------------------------------------------------------------------------------------------------------------------------------------------
Starting                               Ending                                 |  constraint  slack    |  constraint  slack    |  constraint  slack    |  constraint  slack  
----------------------------------------------------------------------------------------------------------------------------------------------------------------------------
IGLOO_TOP|Clk                          IGLOO_TOP|Clk                          |  41.666      29.371   |  No paths    -        |  No paths    -        |  No paths    -      
IMPLANT_TOP|Clk_inferred_clock         IMPLANT_TOP|Clk_inferred_clock         |  10.000      -26.714  |  10.000      -18.750  |  5.000       -13.292  |  5.000       -27.896
IMPLANT_TOP|Clk_inferred_clock         IMPLANT_TOP|Clock_4MHz_inferred_clock  |  Diff grp    -        |  No paths    -        |  No paths    -        |  No paths    -      
IMPLANT_TOP|Clock_4MHz_inferred_clock  IMPLANT_TOP|Clk_inferred_clock         |  Diff grp    -        |  No paths    -        |  No paths    -        |  No paths    -      
IMPLANT_TOP|Clock_4MHz_inferred_clock  IMPLANT_TOP|Clock_4MHz_inferred_clock  |  10.000      -17.743  |  No paths    -        |  No paths    -        |  No paths    -      
============================================================================================================================================================================
 Note: 'No paths' indicates there are no paths in the design for that pair of clock edges.
       'Diff grp' indicates that paths exist but the starting clock and ending clock are in different clock groups.


Interface Information 
*********************

No IO constraint found


====================================
Detailed Report for Clock: IGLOO_TOP|Clk
====================================


Starting Points with Worst Slack
********************************

                              Starting                                                 Arrival           
Instance                      Reference         Type       Pin     Net                 Time        Slack 
                              Clock                                                                      
---------------------------------------------------------------------------------------------------------
MYImplant.Clock_4MHz          IGLOO_TOP|Clk     DFN1C0     Q       Clock_4MHz_i        1.771       29.371
MYImplant.Counter_4MHz[1]     IGLOO_TOP|Clk     DFN1C0     Q       Counter_4MHz[1]     1.771       34.650
MYImplant.Clk                 IGLOO_TOP|Clk     DFN1C0     Q       Clk_i               1.771       35.681
MYImplant.Counter_4MHz[0]     IGLOO_TOP|Clk     DFN1C0     Q       Counter_4MHz[0]     1.771       35.727
=========================================================================================================


Ending Points with Worst Slack
******************************

                              Starting                                                 Required           
Instance                      Reference         Type       Pin     Net                 Time         Slack 
                              Clock                                                                       
----------------------------------------------------------------------------------------------------------
MYImplant.Clock_4MHz          IGLOO_TOP|Clk     DFN1C0     D       Clock_4MHz_RNO      40.371       29.371
MYImplant.Counter_4MHz[0]     IGLOO_TOP|Clk     DFN1C0     D       SUM1                40.371       34.650
MYImplant.Clk                 IGLOO_TOP|Clk     DFN1C0     D       Clk_i_i             40.371       35.681
MYImplant.Counter_4MHz[1]     IGLOO_TOP|Clk     DFN1C0     D       Counter_4MHz[0]     40.288       37.590
==========================================================================================================


Worst Path Information
View Worst Path in Analyst
***********************


Path information for path number 1: 
      Requested Period:                      41.666
    - Setup time:                            1.295
    + Clock delay at ending point:           0.000 (ideal)
    = Required time:                         40.371

    - Propagation time:                      11.000
    - Clock delay at starting point:         0.000 (ideal)
    = Slack (non-critical) :                 29.371

    Number of logic level(s):                1
    Starting point:                          MYImplant.Clock_4MHz / Q
    Ending point:                            MYImplant.Clock_4MHz / D
    The start point is clocked by            IGLOO_TOP|Clk [rising] on pin CLK
    The end   point is clocked by            IGLOO_TOP|Clk [rising] on pin CLK

Instance / Net                          Pin      Pin               Arrival     No. of    
Name                         Type       Name     Dir     Delay     Time        Fan Out(s)
-----------------------------------------------------------------------------------------
MYImplant.Clock_4MHz         DFN1C0     Q        Out     1.771     1.771       -         
Clock_4MHz_i                 Net        -        -       7.284     -           44        
MYImplant.Clock_4MHz_RNO     XOR2       A        In      -         9.054       -         
MYImplant.Clock_4MHz_RNO     XOR2       Y        Out     1.174     10.228      -         
Clock_4MHz_RNO               Net        -        -       0.773     -           1         
MYImplant.Clock_4MHz         DFN1C0     D        In      -         11.000      -         
=========================================================================================
Total path delay (propagation time + setup) of 12.295 is 4.239(34.5%) logic and 8.056(65.5%) route.
Path delay compensated for clock skew. Clock skew is added to clock-to-out value, and is subtracted from setup time value


====================================
Detailed Report for Clock: IMPLANT_TOP|Clk_inferred_clock
====================================


Starting Points with Worst Slack
********************************

                                                                                                    Starting                                                                            Arrival            
Instance                                                                                            Reference                          Type       Pin     Net                           Time        Slack  
                                                                                                    Clock                                                                                                  
-----------------------------------------------------------------------------------------------------------------------------------------------------------------------------------------------------------
MYImplant.Inst_DATA_ACQUISITION_BLOCK.Inst_RHD_TESTMODULE.RHD_TEST_MOD_0.SPIMACHINE_STATE_O[14]     IMPLANT_TOP|Clk_inferred_clock     DFN0C0     Q       SPIMACHINE_STATE_O[14]        1.570       -27.896
MYImplant.Inst_DATA_ACQUISITION_BLOCK.Inst_RHD_TO_ZL_CONVERTER.COUNTER_BITS_OF_A_BYTE[1]            IMPLANT_TOP|Clk_inferred_clock     DFN1C0     Q       COUNTER_BITS_OF_A_BYTE[1]     1.771       -26.714
MYImplant.Inst_DATA_ACQUISITION_BLOCK.Inst_RHD_TO_ZL_CONVERTER.COUNTER_BITS_OF_A_BYTE[0]            IMPLANT_TOP|Clk_inferred_clock     DFN1C0     Q       COUNTER_BITS_OF_A_BYTE[0]     1.771       -26.492
MYImplant.Inst_DATA_ACQUISITION_BLOCK.Inst_RHD_TO_ZL_CONVERTER.COUNTER_BITS_OF_A_BYTE[3]            IMPLANT_TOP|Clk_inferred_clock     DFN1C0     Q       COUNTER_BITS_OF_A_BYTE[3]     1.771       -25.828
MYImplant.Inst_DATA_ACQUISITION_BLOCK.Inst_RHD_TESTMODULE.RHD_TEST_MOD_0.SPIMACHINE_STATE_O[5]      IMPLANT_TOP|Clk_inferred_clock     DFN0C0     Q       SPIMACHINE_STATE_O[5]         1.570       -25.516
MYImplant.Inst_DATA_ACQUISITION_BLOCK.Inst_RHD_TESTMODULE.RHD_TEST_MOD_0.SPIMACHINE_STATE_O[7]      IMPLANT_TOP|Clk_inferred_clock     DFN0C0     Q       SPIMACHINE_STATE_O[7]         1.570       -25.483
MYImplant.Inst_DATA_ACQUISITION_BLOCK.Inst_RHD_TO_ZL_CONVERTER.COUNTER_BITS_OF_A_BYTE[2]            IMPLANT_TOP|Clk_inferred_clock     DFN1C0     Q       COUNTER_BITS_OF_A_BYTE[2]     1.771       -25.473
MYImplant.Inst_DATA_ACQUISITION_BLOCK.Inst_RHD_TESTMODULE.RHD_TEST_MOD_0.SPIMACHINE_STATE_O[2]      IMPLANT_TOP|Clk_inferred_clock     DFN0C0     Q       SPIMACHINE_STATE_O[2]         1.570       -25.232
MYImplant.Inst_DATA_ACQUISITION_BLOCK.Inst_RHD_TESTMODULE.RHD_TEST_MOD_0.SPIMACHINE_STATE_O[8]      IMPLANT_TOP|Clk_inferred_clock     DFN0C0     Q       SPIMACHINE_STATE_O[8]         1.570       -25.232
MYImplant.Inst_DATA_ACQUISITION_BLOCK.Inst_RHD_TESTMODULE.RHD_TEST_MOD_0.SPIMACHINE_STATE_O[6]      IMPLANT_TOP|Clk_inferred_clock     DFN0C0     Q       SPIMACHINE_STATE_O[6]         1.570       -24.973
===========================================================================================================================================================================================================


Ending Points with Worst Slack
******************************

                                                                                    Starting                                                                             Required            
Instance                                                                            Reference                          Type         Pin     Net                          Time         Slack  
                                                                                    Clock                                                                                                    
---------------------------------------------------------------------------------------------------------------------------------------------------------------------------------------------
MYImplant.Inst_DATA_ACQUISITION_BLOCK.Inst_RHD_ARRAY.RHD2.DATAOUT_DATA_Copy[12]     IMPLANT_TOP|Clk_inferred_clock     DFN1E0C0     D       N_29                         3.622        -27.896
MYImplant.Inst_DATA_ACQUISITION_BLOCK.Inst_RHD_ARRAY.RHD5.DATAOUT_DATA_Copy[12]     IMPLANT_TOP|Clk_inferred_clock     DFN1E0C0     D       N_29                         3.622        -27.896
MYImplant.Inst_DATA_ACQUISITION_BLOCK.Inst_RHD_ARRAY.RHD7.DATAOUT_DATA_Copy[12]     IMPLANT_TOP|Clk_inferred_clock     DFN1E0C0     D       N_29                         3.622        -27.896
MYImplant.Inst_DATA_ACQUISITION_BLOCK.Inst_RHD_ARRAY.RHD0.DATAOUT_DATA_Copy[12]     IMPLANT_TOP|Clk_inferred_clock     DFN1E0C0     D       N_61                         3.622        -27.813
MYImplant.Inst_DATA_ACQUISITION_BLOCK.Inst_RHD_ARRAY.RHD2.DATAOUT_DATA_Copy[9]      IMPLANT_TOP|Clk_inferred_clock     DFN1E0C0     D       N_11_i                       3.622        -27.713
MYImplant.Inst_DATA_ACQUISITION_BLOCK.Inst_RHD_ARRAY.RHD6.DATAOUT_DATA_Copy[9]      IMPLANT_TOP|Clk_inferred_clock     DFN1E0C0     D       N_11_i                       3.622        -27.713
MYImplant.Inst_DATA_ACQUISITION_BLOCK.Inst_RHD_ARRAY.RHD5.DATAOUT_DATA_Copy[9]      IMPLANT_TOP|Clk_inferred_clock     DFN1E0C0     D       N_11_i                       3.622        -27.713
MYImplant.Inst_DATA_ACQUISITION_BLOCK.Inst_RHD_ARRAY.RHD0.DATAOUT_DATA_Copy[14]     IMPLANT_TOP|Clk_inferred_clock     DFN1E0C0     D       N_26_i                       3.622        -27.713
MYImplant.Inst_DATA_ACQUISITION_BLOCK.Inst_RHD_ARRAY.RHD7.DATAOUT_DATA_Copy[9]      IMPLANT_TOP|Clk_inferred_clock     DFN1E0C0     D       N_11_i                       3.622        -26.907
MYImplant.Inst_DATA_ACQUISITION_BLOCK.Inst_RHD_ARRAY.RHD1.DATAOUT_DATA_Copy[11]     IMPLANT_TOP|Clk_inferred_clock     DFN1E0C0     D       DATAOUT_DATA_Copy_18[11]     3.622        -26.907
=============================================================================================================================================================================================


Worst Path Information
View Worst Path in Analyst
***********************


Path information for path number 1: 
      Requested Period:                      5.000
    - Setup time:                            1.378
    + Clock delay at ending point:           0.000 (ideal)
    = Required time:                         3.622

    - Propagation time:                      31.518
    - Clock delay at starting point:         0.000 (ideal)
    = Slack (critical) :                     -27.896

    Number of logic level(s):                8
    Starting point:                          MYImplant.Inst_DATA_ACQUISITION_BLOCK.Inst_RHD_TESTMODULE.RHD_TEST_MOD_0.SPIMACHINE_STATE_O[14] / Q
    Ending point:                            MYImplant.Inst_DATA_ACQUISITION_BLOCK.Inst_RHD_ARRAY.RHD5.DATAOUT_DATA_Copy[12] / D
    The start point is clocked by            IMPLANT_TOP|Clk_inferred_clock [falling] on pin CLK
    The end   point is clocked by            IMPLANT_TOP|Clk_inferred_clock [rising] on pin CLK

Instance / Net                                                                                                         Pin      Pin               Arrival     No. of    
Name                                                                                                      Type         Name     Dir     Delay     Time        Fan Out(s)
------------------------------------------------------------------------------------------------------------------------------------------------------------------------
MYImplant.Inst_DATA_ACQUISITION_BLOCK.Inst_RHD_TESTMODULE.RHD_TEST_MOD_0.SPIMACHINE_STATE_O[14]           DFN0C0       Q        Out     1.570     1.570       -         
SPIMACHINE_STATE_O[14]                                                                                    Net          -        -       0.927     -           2         
MYImplant.Inst_DATA_ACQUISITION_BLOCK.Inst_RHD_TESTMODULE.RHD_TEST_MOD_0.DATA_OUT_COPY_RNIEONL[1]         NOR2B        A        In      -         2.498       -         
MYImplant.Inst_DATA_ACQUISITION_BLOCK.Inst_RHD_TESTMODULE.RHD_TEST_MOD_0.DATA_OUT_COPY_RNIEONL[1]         NOR2B        Y        Out     1.236     3.734       -         
DATA_OUT_COPY_m[1]                                                                                        Net          -        -       0.773     -           1         
MYImplant.Inst_DATA_ACQUISITION_BLOCK.Inst_RHD_TESTMODULE.RHD_TEST_MOD_0.DATA_OUT_COPY_RNIL6KB1[8]        AO1          C        In      -         4.506       -         
MYImplant.Inst_DATA_ACQUISITION_BLOCK.Inst_RHD_TESTMODULE.RHD_TEST_MOD_0.DATA_OUT_COPY_RNIL6KB1[8]        AO1          Y        Out     1.520     6.026       -         
RHD_MISO_OUTSIDE_iv_12_1                                                                                  Net          -        -       0.773     -           1         
MYImplant.Inst_DATA_ACQUISITION_BLOCK.Inst_RHD_TESTMODULE.RHD_TEST_MOD_0.DATA_OUT_COPY_RNIA9FJ2[10]       OR3          C        In      -         6.799       -         
MYImplant.Inst_DATA_ACQUISITION_BLOCK.Inst_RHD_TESTMODULE.RHD_TEST_MOD_0.DATA_OUT_COPY_RNIA9FJ2[10]       OR3          Y        Out     1.804     8.603       -         
RHD_MISO_OUTSIDE_iv_12_4                                                                                  Net          -        -       0.773     -           1         
MYImplant.Inst_DATA_ACQUISITION_BLOCK.Inst_RHD_TESTMODULE.RHD_TEST_MOD_0.DATA_OUT_COPY_RNI2R9R4[12]       OR3          C        In      -         9.376       -         
MYImplant.Inst_DATA_ACQUISITION_BLOCK.Inst_RHD_TESTMODULE.RHD_TEST_MOD_0.DATA_OUT_COPY_RNI2R9R4[12]       OR3          Y        Out     1.804     11.180      -         
RHD_MISO_OUTSIDE_iv_12                                                                                    Net          -        -       3.420     -           6         
MYImplant.Inst_DATA_ACQUISITION_BLOCK.Inst_RHD_TESTMODULE.RHD_TEST_MOD_0.DATA_OUT_COPY_RNI46O5A_1[15]     OR3          C        In      -         14.600      -         
MYImplant.Inst_DATA_ACQUISITION_BLOCK.Inst_RHD_TESTMODULE.RHD_TEST_MOD_0.DATA_OUT_COPY_RNI46O5A_1[15]     OR3          Y        Out     1.804     16.404      -         
RHD_MISO_TEST_0[1]                                                                                        Net          -        -       5.329     -           17        
MYImplant.Inst_DATA_ACQUISITION_BLOCK.Inst_RHD_TESTMODULE.RHD_TEST_MOD_0.DATA_OUT_COPY_RNI1NTCA[15]       MX2          B        In      -         21.733      -         
MYImplant.Inst_DATA_ACQUISITION_BLOCK.Inst_RHD_TESTMODULE.RHD_TEST_MOD_0.DATA_OUT_COPY_RNI1NTCA[15]       MX2          Y        Out     1.374     23.107      -         
RHD_MISO_ARRAY[5]                                                                                         Net          -        -       3.420     -           6         
MYImplant.Inst_DATA_ACQUISITION_BLOCK.Inst_RHD_ARRAY.RHD5.DATAOUT_DATA_Copy_RNO_1[12]                     NOR3         C        In      -         26.528      -         
MYImplant.Inst_DATA_ACQUISITION_BLOCK.Inst_RHD_ARRAY.RHD5.DATAOUT_DATA_Copy_RNO_1[12]                     NOR3         Y        Out     1.804     28.332      -         
N_443                                                                                                     Net          -        -       0.773     -           1         
MYImplant.Inst_DATA_ACQUISITION_BLOCK.Inst_RHD_ARRAY.RHD5.DATAOUT_DATA_Copy_RNO[12]                       NOR3         C        In      -         29.104      -         
MYImplant.Inst_DATA_ACQUISITION_BLOCK.Inst_RHD_ARRAY.RHD5.DATAOUT_DATA_Copy_RNO[12]                       NOR3         Y        Out     1.641     30.746      -         
N_29                                                                                                      Net          -        -       0.773     -           1         
MYImplant.Inst_DATA_ACQUISITION_BLOCK.Inst_RHD_ARRAY.RHD5.DATAOUT_DATA_Copy[12]                           DFN1E0C0     D        In      -         31.518      -         
========================================================================================================================================================================
Total path delay (propagation time + setup) of 32.896 is 15.936(48.4%) logic and 16.960(51.6%) route.
Path delay compensated for clock skew. Clock skew is added to clock-to-out value, and is subtracted from setup time value


Path information for path number 2: 
      Requested Period:                      5.000
    - Setup time:                            1.378
    + Clock delay at ending point:           0.000 (ideal)
    = Required time:                         3.622

    - Propagation time:                      31.518
    - Clock delay at starting point:         0.000 (ideal)
    = Slack (critical) :                     -27.896

    Number of logic level(s):                8
    Starting point:                          MYImplant.Inst_DATA_ACQUISITION_BLOCK.Inst_RHD_TESTMODULE.RHD_TEST_MOD_0.SPIMACHINE_STATE_O[14] / Q
    Ending point:                            MYImplant.Inst_DATA_ACQUISITION_BLOCK.Inst_RHD_ARRAY.RHD7.DATAOUT_DATA_Copy[12] / D
    The start point is clocked by            IMPLANT_TOP|Clk_inferred_clock [falling] on pin CLK
    The end   point is clocked by            IMPLANT_TOP|Clk_inferred_clock [rising] on pin CLK

Instance / Net                                                                                                         Pin      Pin               Arrival     No. of    
Name                                                                                                      Type         Name     Dir     Delay     Time        Fan Out(s)
------------------------------------------------------------------------------------------------------------------------------------------------------------------------
MYImplant.Inst_DATA_ACQUISITION_BLOCK.Inst_RHD_TESTMODULE.RHD_TEST_MOD_0.SPIMACHINE_STATE_O[14]           DFN0C0       Q        Out     1.570     1.570       -         
SPIMACHINE_STATE_O[14]                                                                                    Net          -        -       0.927     -           2         
MYImplant.Inst_DATA_ACQUISITION_BLOCK.Inst_RHD_TESTMODULE.RHD_TEST_MOD_0.DATA_OUT_COPY_RNIEONL[1]         NOR2B        A        In      -         2.498       -         
MYImplant.Inst_DATA_ACQUISITION_BLOCK.Inst_RHD_TESTMODULE.RHD_TEST_MOD_0.DATA_OUT_COPY_RNIEONL[1]         NOR2B        Y        Out     1.236     3.734       -         
DATA_OUT_COPY_m[1]                                                                                        Net          -        -       0.773     -           1         
MYImplant.Inst_DATA_ACQUISITION_BLOCK.Inst_RHD_TESTMODULE.RHD_TEST_MOD_0.DATA_OUT_COPY_RNIL6KB1[8]        AO1          C        In      -         4.506       -         
MYImplant.Inst_DATA_ACQUISITION_BLOCK.Inst_RHD_TESTMODULE.RHD_TEST_MOD_0.DATA_OUT_COPY_RNIL6KB1[8]        AO1          Y        Out     1.520     6.026       -         
RHD_MISO_OUTSIDE_iv_12_1                                                                                  Net          -        -       0.773     -           1         
MYImplant.Inst_DATA_ACQUISITION_BLOCK.Inst_RHD_TESTMODULE.RHD_TEST_MOD_0.DATA_OUT_COPY_RNIA9FJ2[10]       OR3          C        In      -         6.799       -         
MYImplant.Inst_DATA_ACQUISITION_BLOCK.Inst_RHD_TESTMODULE.RHD_TEST_MOD_0.DATA_OUT_COPY_RNIA9FJ2[10]       OR3          Y        Out     1.804     8.603       -         
RHD_MISO_OUTSIDE_iv_12_4                                                                                  Net          -        -       0.773     -           1         
MYImplant.Inst_DATA_ACQUISITION_BLOCK.Inst_RHD_TESTMODULE.RHD_TEST_MOD_0.DATA_OUT_COPY_RNI2R9R4[12]       OR3          C        In      -         9.376       -         
MYImplant.Inst_DATA_ACQUISITION_BLOCK.Inst_RHD_TESTMODULE.RHD_TEST_MOD_0.DATA_OUT_COPY_RNI2R9R4[12]       OR3          Y        Out     1.804     11.180      -         
RHD_MISO_OUTSIDE_iv_12                                                                                    Net          -        -       3.420     -           6         
MYImplant.Inst_DATA_ACQUISITION_BLOCK.Inst_RHD_TESTMODULE.RHD_TEST_MOD_0.DATA_OUT_COPY_RNI46O5A_1[15]     OR3          C        In      -         14.600      -         
MYImplant.Inst_DATA_ACQUISITION_BLOCK.Inst_RHD_TESTMODULE.RHD_TEST_MOD_0.DATA_OUT_COPY_RNI46O5A_1[15]     OR3          Y        Out     1.804     16.404      -         
RHD_MISO_TEST_0[1]                                                                                        Net          -        -       5.329     -           17        
MYImplant.Inst_DATA_ACQUISITION_BLOCK.Inst_RHD_TESTMODULE.RHD_TEST_MOD_0.DATA_OUT_COPY_RNI3PTCA[15]       MX2          B        In      -         21.733      -         
MYImplant.Inst_DATA_ACQUISITION_BLOCK.Inst_RHD_TESTMODULE.RHD_TEST_MOD_0.DATA_OUT_COPY_RNI3PTCA[15]       MX2          Y        Out     1.374     23.107      -         
RHD_MISO_ARRAY[7]                                                                                         Net          -        -       3.420     -           6         
MYImplant.Inst_DATA_ACQUISITION_BLOCK.Inst_RHD_ARRAY.RHD7.DATAOUT_DATA_Copy_RNO_1[12]                     NOR3         C        In      -         26.528      -         
MYImplant.Inst_DATA_ACQUISITION_BLOCK.Inst_RHD_ARRAY.RHD7.DATAOUT_DATA_Copy_RNO_1[12]                     NOR3         Y        Out     1.804     28.332      -         
N_443                                                                                                     Net          -        -       0.773     -           1         
MYImplant.Inst_DATA_ACQUISITION_BLOCK.Inst_RHD_ARRAY.RHD7.DATAOUT_DATA_Copy_RNO[12]                       NOR3         C        In      -         29.104      -         
MYImplant.Inst_DATA_ACQUISITION_BLOCK.Inst_RHD_ARRAY.RHD7.DATAOUT_DATA_Copy_RNO[12]                       NOR3         Y        Out     1.641     30.746      -         
N_29                                                                                                      Net          -        -       0.773     -           1         
MYImplant.Inst_DATA_ACQUISITION_BLOCK.Inst_RHD_ARRAY.RHD7.DATAOUT_DATA_Copy[12]                           DFN1E0C0     D        In      -         31.518      -         
========================================================================================================================================================================
Total path delay (propagation time + setup) of 32.896 is 15.936(48.4%) logic and 16.960(51.6%) route.
Path delay compensated for clock skew. Clock skew is added to clock-to-out value, and is subtracted from setup time value


Path information for path number 3: 
      Requested Period:                      5.000
    - Setup time:                            1.378
    + Clock delay at ending point:           0.000 (ideal)
    = Required time:                         3.622

    - Propagation time:                      31.518
    - Clock delay at starting point:         0.000 (ideal)
    = Slack (critical) :                     -27.896

    Number of logic level(s):                8
    Starting point:                          MYImplant.Inst_DATA_ACQUISITION_BLOCK.Inst_RHD_TESTMODULE.RHD_TEST_MOD_0.SPIMACHINE_STATE_O[14] / Q
    Ending point:                            MYImplant.Inst_DATA_ACQUISITION_BLOCK.Inst_RHD_ARRAY.RHD2.DATAOUT_DATA_Copy[12] / D
    The start point is clocked by            IMPLANT_TOP|Clk_inferred_clock [falling] on pin CLK
    The end   point is clocked by            IMPLANT_TOP|Clk_inferred_clock [rising] on pin CLK

Instance / Net                                                                                                         Pin      Pin               Arrival     No. of    
Name                                                                                                      Type         Name     Dir     Delay     Time        Fan Out(s)
------------------------------------------------------------------------------------------------------------------------------------------------------------------------
MYImplant.Inst_DATA_ACQUISITION_BLOCK.Inst_RHD_TESTMODULE.RHD_TEST_MOD_0.SPIMACHINE_STATE_O[14]           DFN0C0       Q        Out     1.570     1.570       -         
SPIMACHINE_STATE_O[14]                                                                                    Net          -        -       0.927     -           2         
MYImplant.Inst_DATA_ACQUISITION_BLOCK.Inst_RHD_TESTMODULE.RHD_TEST_MOD_0.DATA_OUT_COPY_RNIEONL[1]         NOR2B        A        In      -         2.498       -         
MYImplant.Inst_DATA_ACQUISITION_BLOCK.Inst_RHD_TESTMODULE.RHD_TEST_MOD_0.DATA_OUT_COPY_RNIEONL[1]         NOR2B        Y        Out     1.236     3.734       -         
DATA_OUT_COPY_m[1]                                                                                        Net          -        -       0.773     -           1         
MYImplant.Inst_DATA_ACQUISITION_BLOCK.Inst_RHD_TESTMODULE.RHD_TEST_MOD_0.DATA_OUT_COPY_RNIL6KB1[8]        AO1          C        In      -         4.506       -         
MYImplant.Inst_DATA_ACQUISITION_BLOCK.Inst_RHD_TESTMODULE.RHD_TEST_MOD_0.DATA_OUT_COPY_RNIL6KB1[8]        AO1          Y        Out     1.520     6.026       -         
RHD_MISO_OUTSIDE_iv_12_1                                                                                  Net          -        -       0.773     -           1         
MYImplant.Inst_DATA_ACQUISITION_BLOCK.Inst_RHD_TESTMODULE.RHD_TEST_MOD_0.DATA_OUT_COPY_RNIA9FJ2[10]       OR3          C        In      -         6.799       -         
MYImplant.Inst_DATA_ACQUISITION_BLOCK.Inst_RHD_TESTMODULE.RHD_TEST_MOD_0.DATA_OUT_COPY_RNIA9FJ2[10]       OR3          Y        Out     1.804     8.603       -         
RHD_MISO_OUTSIDE_iv_12_4                                                                                  Net          -        -       0.773     -           1         
MYImplant.Inst_DATA_ACQUISITION_BLOCK.Inst_RHD_TESTMODULE.RHD_TEST_MOD_0.DATA_OUT_COPY_RNI2R9R4[12]       OR3          C        In      -         9.376       -         
MYImplant.Inst_DATA_ACQUISITION_BLOCK.Inst_RHD_TESTMODULE.RHD_TEST_MOD_0.DATA_OUT_COPY_RNI2R9R4[12]       OR3          Y        Out     1.804     11.180      -         
RHD_MISO_OUTSIDE_iv_12                                                                                    Net          -        -       3.420     -           6         
MYImplant.Inst_DATA_ACQUISITION_BLOCK.Inst_RHD_TESTMODULE.RHD_TEST_MOD_0.DATA_OUT_COPY_RNI46O5A_1[15]     OR3          C        In      -         14.600      -         
MYImplant.Inst_DATA_ACQUISITION_BLOCK.Inst_RHD_TESTMODULE.RHD_TEST_MOD_0.DATA_OUT_COPY_RNI46O5A_1[15]     OR3          Y        Out     1.804     16.404      -         
RHD_MISO_TEST_0[1]                                                                                        Net          -        -       5.329     -           17        
MYImplant.Inst_DATA_ACQUISITION_BLOCK.Inst_RHD_TESTMODULE.RHD_TEST_MOD_0.DATA_OUT_COPY_RNIUJTCA[15]       MX2          B        In      -         21.733      -         
MYImplant.Inst_DATA_ACQUISITION_BLOCK.Inst_RHD_TESTMODULE.RHD_TEST_MOD_0.DATA_OUT_COPY_RNIUJTCA[15]       MX2          Y        Out     1.374     23.107      -         
RHD_MISO_ARRAY[2]                                                                                         Net          -        -       3.420     -           6         
MYImplant.Inst_DATA_ACQUISITION_BLOCK.Inst_RHD_ARRAY.RHD2.DATAOUT_DATA_Copy_RNO_1[12]                     NOR3         C        In      -         26.528      -         
MYImplant.Inst_DATA_ACQUISITION_BLOCK.Inst_RHD_ARRAY.RHD2.DATAOUT_DATA_Copy_RNO_1[12]                     NOR3         Y        Out     1.804     28.332      -         
N_443                                                                                                     Net          -        -       0.773     -           1         
MYImplant.Inst_DATA_ACQUISITION_BLOCK.Inst_RHD_ARRAY.RHD2.DATAOUT_DATA_Copy_RNO[12]                       NOR3         C        In      -         29.104      -         
MYImplant.Inst_DATA_ACQUISITION_BLOCK.Inst_RHD_ARRAY.RHD2.DATAOUT_DATA_Copy_RNO[12]                       NOR3         Y        Out     1.641     30.746      -         
N_29                                                                                                      Net          -        -       0.773     -           1         
MYImplant.Inst_DATA_ACQUISITION_BLOCK.Inst_RHD_ARRAY.RHD2.DATAOUT_DATA_Copy[12]                           DFN1E0C0     D        In      -         31.518      -         
========================================================================================================================================================================
Total path delay (propagation time + setup) of 32.896 is 15.936(48.4%) logic and 16.960(51.6%) route.
Path delay compensated for clock skew. Clock skew is added to clock-to-out value, and is subtracted from setup time value


Path information for path number 4: 
      Requested Period:                      5.000
    - Setup time:                            1.378
    + Clock delay at ending point:           0.000 (ideal)
    = Required time:                         3.622

    - Propagation time:                      31.435
    - Clock delay at starting point:         0.000 (ideal)
    = Slack (non-critical) :                 -27.813

    Number of logic level(s):                8
    Starting point:                          MYImplant.Inst_DATA_ACQUISITION_BLOCK.Inst_RHD_TESTMODULE.RHD_TEST_MOD_0.SPIMACHINE_STATE_O[14] / Q
    Ending point:                            MYImplant.Inst_DATA_ACQUISITION_BLOCK.Inst_RHD_ARRAY.RHD0.DATAOUT_DATA_Copy[12] / D
    The start point is clocked by            IMPLANT_TOP|Clk_inferred_clock [falling] on pin CLK
    The end   point is clocked by            IMPLANT_TOP|Clk_inferred_clock [rising] on pin CLK

Instance / Net                                                                                                         Pin      Pin               Arrival     No. of    
Name                                                                                                      Type         Name     Dir     Delay     Time        Fan Out(s)
------------------------------------------------------------------------------------------------------------------------------------------------------------------------
MYImplant.Inst_DATA_ACQUISITION_BLOCK.Inst_RHD_TESTMODULE.RHD_TEST_MOD_0.SPIMACHINE_STATE_O[14]           DFN0C0       Q        Out     1.570     1.570       -         
SPIMACHINE_STATE_O[14]                                                                                    Net          -        -       0.927     -           2         
MYImplant.Inst_DATA_ACQUISITION_BLOCK.Inst_RHD_TESTMODULE.RHD_TEST_MOD_0.DATA_OUT_COPY_RNIEONL[1]         NOR2B        A        In      -         2.498       -         
MYImplant.Inst_DATA_ACQUISITION_BLOCK.Inst_RHD_TESTMODULE.RHD_TEST_MOD_0.DATA_OUT_COPY_RNIEONL[1]         NOR2B        Y        Out     1.236     3.734       -         
DATA_OUT_COPY_m[1]                                                                                        Net          -        -       0.773     -           1         
MYImplant.Inst_DATA_ACQUISITION_BLOCK.Inst_RHD_TESTMODULE.RHD_TEST_MOD_0.DATA_OUT_COPY_RNIL6KB1[8]        AO1          C        In      -         4.506       -         
MYImplant.Inst_DATA_ACQUISITION_BLOCK.Inst_RHD_TESTMODULE.RHD_TEST_MOD_0.DATA_OUT_COPY_RNIL6KB1[8]        AO1          Y        Out     1.520     6.026       -         
RHD_MISO_OUTSIDE_iv_12_1                                                                                  Net          -        -       0.773     -           1         
MYImplant.Inst_DATA_ACQUISITION_BLOCK.Inst_RHD_TESTMODULE.RHD_TEST_MOD_0.DATA_OUT_COPY_RNIA9FJ2[10]       OR3          C        In      -         6.799       -         
MYImplant.Inst_DATA_ACQUISITION_BLOCK.Inst_RHD_TESTMODULE.RHD_TEST_MOD_0.DATA_OUT_COPY_RNIA9FJ2[10]       OR3          Y        Out     1.804     8.603       -         
RHD_MISO_OUTSIDE_iv_12_4                                                                                  Net          -        -       0.773     -           1         
MYImplant.Inst_DATA_ACQUISITION_BLOCK.Inst_RHD_TESTMODULE.RHD_TEST_MOD_0.DATA_OUT_COPY_RNI2R9R4[12]       OR3          C        In      -         9.376       -         
MYImplant.Inst_DATA_ACQUISITION_BLOCK.Inst_RHD_TESTMODULE.RHD_TEST_MOD_0.DATA_OUT_COPY_RNI2R9R4[12]       OR3          Y        Out     1.804     11.180      -         
RHD_MISO_OUTSIDE_iv_12                                                                                    Net          -        -       3.420     -           6         
MYImplant.Inst_DATA_ACQUISITION_BLOCK.Inst_RHD_TESTMODULE.RHD_TEST_MOD_0.DATA_OUT_COPY_RNI46O5A_1[15]     OR3          C        In      -         14.600      -         
MYImplant.Inst_DATA_ACQUISITION_BLOCK.Inst_RHD_TESTMODULE.RHD_TEST_MOD_0.DATA_OUT_COPY_RNI46O5A_1[15]     OR3          Y        Out     1.804     16.404      -         
RHD_MISO_TEST_0[1]                                                                                        Net          -        -       5.329     -           17        
MYImplant.Inst_DATA_ACQUISITION_BLOCK.Inst_RHD_TESTMODULE.RHD_TEST_MOD_0.DATA_OUT_COPY_RNISHTCA[15]       MX2          B        In      -         21.733      -         
MYImplant.Inst_DATA_ACQUISITION_BLOCK.Inst_RHD_TESTMODULE.RHD_TEST_MOD_0.DATA_OUT_COPY_RNISHTCA[15]       MX2          Y        Out     1.374     23.107      -         
RHD_MISO_ARRAY[0]                                                                                         Net          -        -       3.420     -           6         
MYImplant.Inst_DATA_ACQUISITION_BLOCK.Inst_RHD_ARRAY.RHD0.DATAOUT_DATA_Copy_RNO_1[12]                     NOR3A        C        In      -         26.528      -         
MYImplant.Inst_DATA_ACQUISITION_BLOCK.Inst_RHD_ARRAY.RHD0.DATAOUT_DATA_Copy_RNO_1[12]                     NOR3A        Y        Out     1.721     28.248      -         
N_179                                                                                                     Net          -        -       0.773     -           1         
MYImplant.Inst_DATA_ACQUISITION_BLOCK.Inst_RHD_ARRAY.RHD0.DATAOUT_DATA_Copy_RNO[12]                       NOR3         C        In      -         29.021      -         
MYImplant.Inst_DATA_ACQUISITION_BLOCK.Inst_RHD_ARRAY.RHD0.DATAOUT_DATA_Copy_RNO[12]                       NOR3         Y        Out     1.641     30.662      -         
N_61                                                                                                      Net          -        -       0.773     -           1         
MYImplant.Inst_DATA_ACQUISITION_BLOCK.Inst_RHD_ARRAY.RHD0.DATAOUT_DATA_Copy[12]                           DFN1E0C0     D        In      -         31.435      -         
========================================================================================================================================================================
Total path delay (propagation time + setup) of 32.813 is 15.853(48.3%) logic and 16.960(51.7%) route.
Path delay compensated for clock skew. Clock skew is added to clock-to-out value, and is subtracted from setup time value


Path information for path number 5: 
      Requested Period:                      5.000
    - Setup time:                            1.378
    + Clock delay at ending point:           0.000 (ideal)
    = Required time:                         3.622

    - Propagation time:                      31.334
    - Clock delay at starting point:         0.000 (ideal)
    = Slack (non-critical) :                 -27.713

    Number of logic level(s):                8
    Starting point:                          MYImplant.Inst_DATA_ACQUISITION_BLOCK.Inst_RHD_TESTMODULE.RHD_TEST_MOD_0.SPIMACHINE_STATE_O[14] / Q
    Ending point:                            MYImplant.Inst_DATA_ACQUISITION_BLOCK.Inst_RHD_ARRAY.RHD6.DATAOUT_DATA_Copy[9] / D
    The start point is clocked by            IMPLANT_TOP|Clk_inferred_clock [falling] on pin CLK
    The end   point is clocked by            IMPLANT_TOP|Clk_inferred_clock [rising] on pin CLK

Instance / Net                                                                                                         Pin      Pin               Arrival     No. of    
Name                                                                                                      Type         Name     Dir     Delay     Time        Fan Out(s)
------------------------------------------------------------------------------------------------------------------------------------------------------------------------
MYImplant.Inst_DATA_ACQUISITION_BLOCK.Inst_RHD_TESTMODULE.RHD_TEST_MOD_0.SPIMACHINE_STATE_O[14]           DFN0C0       Q        Out     1.570     1.570       -         
SPIMACHINE_STATE_O[14]                                                                                    Net          -        -       0.927     -           2         
MYImplant.Inst_DATA_ACQUISITION_BLOCK.Inst_RHD_TESTMODULE.RHD_TEST_MOD_0.DATA_OUT_COPY_RNIEONL[1]         NOR2B        A        In      -         2.498       -         
MYImplant.Inst_DATA_ACQUISITION_BLOCK.Inst_RHD_TESTMODULE.RHD_TEST_MOD_0.DATA_OUT_COPY_RNIEONL[1]         NOR2B        Y        Out     1.236     3.734       -         
DATA_OUT_COPY_m[1]                                                                                        Net          -        -       0.773     -           1         
MYImplant.Inst_DATA_ACQUISITION_BLOCK.Inst_RHD_TESTMODULE.RHD_TEST_MOD_0.DATA_OUT_COPY_RNIL6KB1[8]        AO1          C        In      -         4.506       -         
MYImplant.Inst_DATA_ACQUISITION_BLOCK.Inst_RHD_TESTMODULE.RHD_TEST_MOD_0.DATA_OUT_COPY_RNIL6KB1[8]        AO1          Y        Out     1.520     6.026       -         
RHD_MISO_OUTSIDE_iv_12_1                                                                                  Net          -        -       0.773     -           1         
MYImplant.Inst_DATA_ACQUISITION_BLOCK.Inst_RHD_TESTMODULE.RHD_TEST_MOD_0.DATA_OUT_COPY_RNIA9FJ2[10]       OR3          C        In      -         6.799       -         
MYImplant.Inst_DATA_ACQUISITION_BLOCK.Inst_RHD_TESTMODULE.RHD_TEST_MOD_0.DATA_OUT_COPY_RNIA9FJ2[10]       OR3          Y        Out     1.804     8.603       -         
RHD_MISO_OUTSIDE_iv_12_4                                                                                  Net          -        -       0.773     -           1         
MYImplant.Inst_DATA_ACQUISITION_BLOCK.Inst_RHD_TESTMODULE.RHD_TEST_MOD_0.DATA_OUT_COPY_RNI2R9R4[12]       OR3          C        In      -         9.376       -         
MYImplant.Inst_DATA_ACQUISITION_BLOCK.Inst_RHD_TESTMODULE.RHD_TEST_MOD_0.DATA_OUT_COPY_RNI2R9R4[12]       OR3          Y        Out     1.804     11.180      -         
RHD_MISO_OUTSIDE_iv_12                                                                                    Net          -        -       3.420     -           6         
MYImplant.Inst_DATA_ACQUISITION_BLOCK.Inst_RHD_TESTMODULE.RHD_TEST_MOD_0.DATA_OUT_COPY_RNI46O5A_1[15]     OR3          C        In      -         14.600      -         
MYImplant.Inst_DATA_ACQUISITION_BLOCK.Inst_RHD_TESTMODULE.RHD_TEST_MOD_0.DATA_OUT_COPY_RNI46O5A_1[15]     OR3          Y        Out     1.804     16.404      -         
RHD_MISO_TEST_0[1]                                                                                        Net          -        -       5.329     -           17        
MYImplant.Inst_DATA_ACQUISITION_BLOCK.Inst_RHD_TESTMODULE.RHD_TEST_MOD_0.DATA_OUT_COPY_RNI2OTCA[15]       MX2          B        In      -         21.733      -         
MYImplant.Inst_DATA_ACQUISITION_BLOCK.Inst_RHD_TESTMODULE.RHD_TEST_MOD_0.DATA_OUT_COPY_RNI2OTCA[15]       MX2          Y        Out     1.374     23.107      -         
RHD_MISO_ARRAY[6]                                                                                         Net          -        -       3.420     -           6         
MYImplant.Inst_DATA_ACQUISITION_BLOCK.Inst_RHD_ARRAY.RHD6.DATAOUT_DATA_Copy_RNO_2[9]                      NOR3B        B        In      -         26.528      -         
MYImplant.Inst_DATA_ACQUISITION_BLOCK.Inst_RHD_ARRAY.RHD6.DATAOUT_DATA_Copy_RNO_2[9]                      NOR3B        Y        Out     1.458     27.985      -         
N_162                                                                                                     Net          -        -       0.773     -           1         
MYImplant.Inst_DATA_ACQUISITION_BLOCK.Inst_RHD_ARRAY.RHD6.DATAOUT_DATA_Copy_RNO[9]                        OR3          C        In      -         28.758      -         
MYImplant.Inst_DATA_ACQUISITION_BLOCK.Inst_RHD_ARRAY.RHD6.DATAOUT_DATA_Copy_RNO[9]                        OR3          Y        Out     1.804     30.562      -         
N_11_i                                                                                                    Net          -        -       0.773     -           1         
MYImplant.Inst_DATA_ACQUISITION_BLOCK.Inst_RHD_ARRAY.RHD6.DATAOUT_DATA_Copy[9]                            DFN1E0C0     D        In      -         31.334      -         
========================================================================================================================================================================
Total path delay (propagation time + setup) of 32.713 is 15.753(48.2%) logic and 16.960(51.8%) route.
Path delay compensated for clock skew. Clock skew is added to clock-to-out value, and is subtracted from setup time value


====================================
Detailed Report for Clock: IMPLANT_TOP|Clock_4MHz_inferred_clock
====================================


Starting Points with Worst Slack
********************************

                                                                        Starting                                                                          Arrival            
Instance                                                                Reference                                 Type         Pin     Net                Time        Slack  
                                                                        Clock                                                                                                
-----------------------------------------------------------------------------------------------------------------------------------------------------------------------------
MYImplant.Inst_CONTROL_NEXUS.Inst_Zarlink_SPI_Module.FSM_STATE[5]       IMPLANT_TOP|Clock_4MHz_inferred_clock     DFN1C0       Q       FSM_STATE[5]       1.771       -17.743
MYImplant.Inst_CONTROL_NEXUS.Inst_Zarlink_SPI_Module.FSM_STATE[2]       IMPLANT_TOP|Clock_4MHz_inferred_clock     DFN1C0       Q       FSM_STATE[2]       1.395       -17.567
MYImplant.Inst_CONTROL_NEXUS.Inst_Zarlink_SPI_Module.FSM_STATE[4]       IMPLANT_TOP|Clock_4MHz_inferred_clock     DFN1C0       Q       FSM_STATE[4]       1.771       -17.526
MYImplant.Inst_CONTROL_NEXUS.Inst_Zarlink_SPI_Module.FSM_STATE[0]       IMPLANT_TOP|Clock_4MHz_inferred_clock     DFN1C0       Q       FSM_STATE[0]       1.771       -17.108
MYImplant.Inst_CONTROL_NEXUS.Inst_Zarlink_SPI_Module.FSM_STATE[3]       IMPLANT_TOP|Clock_4MHz_inferred_clock     DFN1C0       Q       FSM_STATE[3]       1.771       -16.273
MYImplant.Inst_CONTROL_NEXUS.Inst_Zarlink_SPI_Module.FSM_STATE[1]       IMPLANT_TOP|Clock_4MHz_inferred_clock     DFN1C0       Q       FSM_STATE[1]       1.771       -14.586
MYImplant.Inst_CONTROL_NEXUS.Inst_Zarlink_SPI_Module.FSM_STATE_0[3]     IMPLANT_TOP|Clock_4MHz_inferred_clock     DFN1C0       Q       FSM_STATE_0[3]     1.771       -10.990
MYImplant.Inst_CONTROL_NEXUS.Inst_Zarlink_SPI_Module.FSM_STATE_0[4]     IMPLANT_TOP|Clock_4MHz_inferred_clock     DFN1C0       Q       FSM_STATE_0[4]     1.771       -9.887 
MYImplant.Inst_CONTROL_NEXUS.Inst_Zarlink_SPI_Module.Counter[0]         IMPLANT_TOP|Clock_4MHz_inferred_clock     DFN1E0C0     Q       Counter[0]         1.771       -8.113 
MYImplant.Inst_CONTROL_NEXUS.Inst_Zarlink_SPI_Module.Counter[2]         IMPLANT_TOP|Clock_4MHz_inferred_clock     DFN1E0C0     Q       Counter[2]         1.771       -7.319 
=============================================================================================================================================================================


Ending Points with Worst Slack
******************************

                                                                                   Starting                                                                                 Required            
Instance                                                                           Reference                                 Type         Pin     Net                       Time         Slack  
                                                                                   Clock                                                                                                        
------------------------------------------------------------------------------------------------------------------------------------------------------------------------------------------------
MYImplant.Inst_CONTROL_NEXUS.Inst_Zarlink_SPI_Module.SPI_SDI                       IMPLANT_TOP|Clock_4MHz_inferred_clock     DFN1C0       D       SPI_SDI_18                8.705        -17.743
MYImplant.Inst_CONTROL_NEXUS.Inst_Zarlink_SPI_Module.FSM_STATE[2]                  IMPLANT_TOP|Clock_4MHz_inferred_clock     DFN1C0       D       FSM_STATE_ns[2]           8.622        -14.903
MYImplant.Inst_CONTROL_NEXUS.Inst_Zarlink_SPI_Module.FSM_STATE[3]                  IMPLANT_TOP|Clock_4MHz_inferred_clock     DFN1C0       D       FSM_STATE_RNIGE8O5[4]     8.705        -14.465
MYImplant.Inst_CONTROL_NEXUS.Inst_Zarlink_SPI_Module.FSM_STATE_0[3]                IMPLANT_TOP|Clock_4MHz_inferred_clock     DFN1C0       D       FSM_STATE_RNIGE8O5[4]     8.705        -14.465
MYImplant.Inst_CONTROL_NEXUS.Inst_Zarlink_SPI_Module.FSM_STATE[4]                  IMPLANT_TOP|Clock_4MHz_inferred_clock     DFN1C0       D       FSM_STATE_ns[4]           8.705        -13.057
MYImplant.Inst_CONTROL_NEXUS.Inst_Zarlink_SPI_Module.FSM_STATE_0[4]                IMPLANT_TOP|Clock_4MHz_inferred_clock     DFN1C0       D       FSM_STATE_ns[4]           8.705        -13.057
MYImplant.Inst_CONTROL_NEXUS.Inst_Zarlink_SPI_Module.FSM_STATE[1]                  IMPLANT_TOP|Clock_4MHz_inferred_clock     DFN1C0       D       FSM_STATE_ns[1]           8.705        -12.435
MYImplant.Inst_CONTROL_NEXUS.Inst_Zarlink_SPI_Module.Zarlink_Data_In_Buffer[0]     IMPLANT_TOP|Clock_4MHz_inferred_clock     DFN1E1C0     E       un1_FSM_STATE_20          8.538        -11.800
MYImplant.Inst_CONTROL_NEXUS.Inst_Zarlink_SPI_Module.Zarlink_Data_In_Buffer[1]     IMPLANT_TOP|Clock_4MHz_inferred_clock     DFN1E1C0     E       un1_FSM_STATE_20          8.538        -11.800
MYImplant.Inst_CONTROL_NEXUS.Inst_Zarlink_SPI_Module.Zarlink_Data_In_Buffer[2]     IMPLANT_TOP|Clock_4MHz_inferred_clock     DFN1E1C0     E       un1_FSM_STATE_20          8.538        -11.800
================================================================================================================================================================================================


Worst Path Information
View Worst Path in Analyst
***********************


Path information for path number 1: 
      Requested Period:                      10.000
    - Setup time:                            1.295
    + Clock delay at ending point:           0.000 (ideal)
    = Required time:                         8.705

    - Propagation time:                      26.448
    - Clock delay at starting point:         0.000 (ideal)
    = Slack (non-critical) :                 -17.743

    Number of logic level(s):                6
    Starting point:                          MYImplant.Inst_CONTROL_NEXUS.Inst_Zarlink_SPI_Module.FSM_STATE[5] / Q
    Ending point:                            MYImplant.Inst_CONTROL_NEXUS.Inst_Zarlink_SPI_Module.SPI_SDI / D
    The start point is clocked by            IMPLANT_TOP|Clock_4MHz_inferred_clock [rising] on pin CLK
    The end   point is clocked by            IMPLANT_TOP|Clock_4MHz_inferred_clock [rising] on pin CLK

Instance / Net                                                                              Pin      Pin               Arrival     No. of    
Name                                                                             Type       Name     Dir     Delay     Time        Fan Out(s)
---------------------------------------------------------------------------------------------------------------------------------------------
MYImplant.Inst_CONTROL_NEXUS.Inst_Zarlink_SPI_Module.FSM_STATE[5]                DFN1C0     Q        Out     1.771     1.771       -         
FSM_STATE[5]                                                                     Net        -        -       5.926     -           24        
MYImplant.Inst_CONTROL_NEXUS.Inst_Zarlink_SPI_Module.FSM_STATE_RNIU82J[3]        OR2        B        In      -         7.697       -         
MYImplant.Inst_CONTROL_NEXUS.Inst_Zarlink_SPI_Module.FSM_STATE_RNIU82J[3]        OR2        Y        Out     1.554     9.251       -         
N_60                                                                             Net        -        -       3.667     -           7         
MYImplant.Inst_CONTROL_NEXUS.Inst_Zarlink_SPI_Module.FSM_STATE_RNIQF461_0[3]     NOR2A      B        In      -         12.917      -         
MYImplant.Inst_CONTROL_NEXUS.Inst_Zarlink_SPI_Module.FSM_STATE_RNIQF461_0[3]     NOR2A      Y        Out     0.977     13.895      -         
N_595_2                                                                          Net        -        -       3.938     -           8         
MYImplant.Inst_CONTROL_NEXUS.Inst_Zarlink_SPI_Module.SPI_SDI_RNO_20              AO1        B        In      -         17.833      -         
MYImplant.Inst_CONTROL_NEXUS.Inst_Zarlink_SPI_Module.SPI_SDI_RNO_20              AO1        Y        Out     1.361     19.194      -         
SPI_SDI_18_iv_0_0_5_tz                                                           Net        -        -       0.773     -           1         
MYImplant.Inst_CONTROL_NEXUS.Inst_Zarlink_SPI_Module.SPI_SDI_RNO_8               MX2C       B        In      -         19.967      -         
MYImplant.Inst_CONTROL_NEXUS.Inst_Zarlink_SPI_Module.SPI_SDI_RNO_8               MX2C       Y        Out     1.407     21.374      -         
SPI_SDI_RNO_8                                                                    Net        -        -       0.773     -           1         
MYImplant.Inst_CONTROL_NEXUS.Inst_Zarlink_SPI_Module.SPI_SDI_RNO_2               OR3A       A        In      -         22.147      -         
MYImplant.Inst_CONTROL_NEXUS.Inst_Zarlink_SPI_Module.SPI_SDI_RNO_2               OR3A       Y        Out     1.115     23.262      -         
SPI_SDI_18_iv_0_0_9                                                              Net        -        -       0.773     -           1         
MYImplant.Inst_CONTROL_NEXUS.Inst_Zarlink_SPI_Module.SPI_SDI_RNO                 OR3        C        In      -         24.034      -         
MYImplant.Inst_CONTROL_NEXUS.Inst_Zarlink_SPI_Module.SPI_SDI_RNO                 OR3        Y        Out     1.641     25.676      -         
SPI_SDI_18                                                                       Net        -        -       0.773     -           1         
MYImplant.Inst_CONTROL_NEXUS.Inst_Zarlink_SPI_Module.SPI_SDI                     DFN1C0     D        In      -         26.448      -         
=============================================================================================================================================
Total path delay (propagation time + setup) of 27.743 is 11.121(40.1%) logic and 16.622(59.9%) route.
Path delay compensated for clock skew. Clock skew is added to clock-to-out value, and is subtracted from setup time value


Path information for path number 2: 
      Requested Period:                      10.000
    - Setup time:                            1.378
    + Clock delay at ending point:           0.000 (ideal)
    = Required time:                         8.622

    - Propagation time:                      26.189
    - Clock delay at starting point:         0.000 (ideal)
    = Slack (non-critical) :                 -17.567

    Number of logic level(s):                6
    Starting point:                          MYImplant.Inst_CONTROL_NEXUS.Inst_Zarlink_SPI_Module.FSM_STATE[2] / Q
    Ending point:                            MYImplant.Inst_CONTROL_NEXUS.Inst_Zarlink_SPI_Module.SPI_SDI / D
    The start point is clocked by            IMPLANT_TOP|Clock_4MHz_inferred_clock [rising] on pin CLK
    The end   point is clocked by            IMPLANT_TOP|Clock_4MHz_inferred_clock [rising] on pin CLK

Instance / Net                                                                              Pin      Pin               Arrival     No. of    
Name                                                                             Type       Name     Dir     Delay     Time        Fan Out(s)
---------------------------------------------------------------------------------------------------------------------------------------------
MYImplant.Inst_CONTROL_NEXUS.Inst_Zarlink_SPI_Module.FSM_STATE[2]                DFN1C0     Q        Out     1.395     1.395       -         
FSM_STATE[2]                                                                     Net        -        -       5.788     -           22        
MYImplant.Inst_CONTROL_NEXUS.Inst_Zarlink_SPI_Module.FSM_STATE_RNIS62J_1[4]      NOR2A      B        In      -         7.183       -         
MYImplant.Inst_CONTROL_NEXUS.Inst_Zarlink_SPI_Module.FSM_STATE_RNIS62J_1[4]      NOR2A      Y        Out     0.927     8.110       -         
N_216                                                                            Net        -        -       3.667     -           7         
MYImplant.Inst_CONTROL_NEXUS.Inst_Zarlink_SPI_Module.FSM_STATE_RNIQF461_0[3]     NOR2A      A        In      -         11.777      -         
MYImplant.Inst_CONTROL_NEXUS.Inst_Zarlink_SPI_Module.FSM_STATE_RNIQF461_0[3]     NOR2A      Y        Out     1.508     13.285      -         
N_595_2                                                                          Net        -        -       3.938     -           8         
MYImplant.Inst_CONTROL_NEXUS.Inst_Zarlink_SPI_Module.SPI_SDI_RNO_20              AO1        B        In      -         17.223      -         
MYImplant.Inst_CONTROL_NEXUS.Inst_Zarlink_SPI_Module.SPI_SDI_RNO_20              AO1        Y        Out     1.437     18.660      -         
SPI_SDI_18_iv_0_0_5_tz                                                           Net        -        -       0.773     -           1         
MYImplant.Inst_CONTROL_NEXUS.Inst_Zarlink_SPI_Module.SPI_SDI_RNO_8               MX2C       B        In      -         19.432      -         
MYImplant.Inst_CONTROL_NEXUS.Inst_Zarlink_SPI_Module.SPI_SDI_RNO_8               MX2C       Y        Out     1.374     20.806      -         
SPI_SDI_RNO_8                                                                    Net        -        -       0.773     -           1         
MYImplant.Inst_CONTROL_NEXUS.Inst_Zarlink_SPI_Module.SPI_SDI_RNO_2               OR3A       A        In      -         21.579      -         
MYImplant.Inst_CONTROL_NEXUS.Inst_Zarlink_SPI_Module.SPI_SDI_RNO_2               OR3A       Y        Out     1.261     22.840      -         
SPI_SDI_18_iv_0_0_9                                                              Net        -        -       0.773     -           1         
MYImplant.Inst_CONTROL_NEXUS.Inst_Zarlink_SPI_Module.SPI_SDI_RNO                 OR3        C        In      -         23.613      -         
MYImplant.Inst_CONTROL_NEXUS.Inst_Zarlink_SPI_Module.SPI_SDI_RNO                 OR3        Y        Out     1.804     25.417      -         
SPI_SDI_18                                                                       Net        -        -       0.773     -           1         
MYImplant.Inst_CONTROL_NEXUS.Inst_Zarlink_SPI_Module.SPI_SDI                     DFN1C0     D        In      -         26.189      -         
=============================================================================================================================================
Total path delay (propagation time + setup) of 27.567 is 11.084(40.2%) logic and 16.484(59.8%) route.
Path delay compensated for clock skew. Clock skew is added to clock-to-out value, and is subtracted from setup time value


Path information for path number 3: 
      Requested Period:                      10.000
    - Setup time:                            1.378
    + Clock delay at ending point:           0.000 (ideal)
    = Required time:                         8.622

    - Propagation time:                      26.148
    - Clock delay at starting point:         0.000 (ideal)
    = Slack (non-critical) :                 -17.526

    Number of logic level(s):                6
    Starting point:                          MYImplant.Inst_CONTROL_NEXUS.Inst_Zarlink_SPI_Module.FSM_STATE[4] / Q
    Ending point:                            MYImplant.Inst_CONTROL_NEXUS.Inst_Zarlink_SPI_Module.SPI_SDI / D
    The start point is clocked by            IMPLANT_TOP|Clock_4MHz_inferred_clock [rising] on pin CLK
    The end   point is clocked by            IMPLANT_TOP|Clock_4MHz_inferred_clock [rising] on pin CLK

Instance / Net                                                                              Pin      Pin               Arrival     No. of    
Name                                                                             Type       Name     Dir     Delay     Time        Fan Out(s)
---------------------------------------------------------------------------------------------------------------------------------------------
MYImplant.Inst_CONTROL_NEXUS.Inst_Zarlink_SPI_Module.FSM_STATE[4]                DFN1C0     Q        Out     1.771     1.771       -         
FSM_STATE[4]                                                                     Net        -        -       4.790     -           12        
MYImplant.Inst_CONTROL_NEXUS.Inst_Zarlink_SPI_Module.FSM_STATE_RNIS62J_1[4]      NOR2A      A        In      -         6.561       -         
MYImplant.Inst_CONTROL_NEXUS.Inst_Zarlink_SPI_Module.FSM_STATE_RNIS62J_1[4]      NOR2A      Y        Out     1.508     8.069       -         
N_216                                                                            Net        -        -       3.667     -           7         
MYImplant.Inst_CONTROL_NEXUS.Inst_Zarlink_SPI_Module.FSM_STATE_RNIQF461_0[3]     NOR2A      A        In      -         11.735      -         
MYImplant.Inst_CONTROL_NEXUS.Inst_Zarlink_SPI_Module.FSM_STATE_RNIQF461_0[3]     NOR2A      Y        Out     1.508     13.243      -         
N_595_2                                                                          Net        -        -       3.938     -           8         
MYImplant.Inst_CONTROL_NEXUS.Inst_Zarlink_SPI_Module.SPI_SDI_RNO_20              AO1        B        In      -         17.181      -         
MYImplant.Inst_CONTROL_NEXUS.Inst_Zarlink_SPI_Module.SPI_SDI_RNO_20              AO1        Y        Out     1.437     18.618      -         
SPI_SDI_18_iv_0_0_5_tz                                                           Net        -        -       0.773     -           1         
MYImplant.Inst_CONTROL_NEXUS.Inst_Zarlink_SPI_Module.SPI_SDI_RNO_8               MX2C       B        In      -         19.390      -         
MYImplant.Inst_CONTROL_NEXUS.Inst_Zarlink_SPI_Module.SPI_SDI_RNO_8               MX2C       Y        Out     1.374     20.765      -         
SPI_SDI_RNO_8                                                                    Net        -        -       0.773     -           1         
MYImplant.Inst_CONTROL_NEXUS.Inst_Zarlink_SPI_Module.SPI_SDI_RNO_2               OR3A       A        In      -         21.537      -         
MYImplant.Inst_CONTROL_NEXUS.Inst_Zarlink_SPI_Module.SPI_SDI_RNO_2               OR3A       Y        Out     1.261     22.798      -         
SPI_SDI_18_iv_0_0_9                                                              Net        -        -       0.773     -           1         
MYImplant.Inst_CONTROL_NEXUS.Inst_Zarlink_SPI_Module.SPI_SDI_RNO                 OR3        C        In      -         23.571      -         
MYImplant.Inst_CONTROL_NEXUS.Inst_Zarlink_SPI_Module.SPI_SDI_RNO                 OR3        Y        Out     1.804     25.375      -         
SPI_SDI_18                                                                       Net        -        -       0.773     -           1         
MYImplant.Inst_CONTROL_NEXUS.Inst_Zarlink_SPI_Module.SPI_SDI                     DFN1C0     D        In      -         26.148      -         
=============================================================================================================================================
Total path delay (propagation time + setup) of 27.526 is 12.040(43.7%) logic and 15.486(56.3%) route.
Path delay compensated for clock skew. Clock skew is added to clock-to-out value, and is subtracted from setup time value


Path information for path number 4: 
      Requested Period:                      10.000
    - Setup time:                            1.378
    + Clock delay at ending point:           0.000 (ideal)
    = Required time:                         8.622

    - Propagation time:                      25.730
    - Clock delay at starting point:         0.000 (ideal)
    = Slack (non-critical) :                 -17.108

    Number of logic level(s):                6
    Starting point:                          MYImplant.Inst_CONTROL_NEXUS.Inst_Zarlink_SPI_Module.FSM_STATE[0] / Q
    Ending point:                            MYImplant.Inst_CONTROL_NEXUS.Inst_Zarlink_SPI_Module.SPI_SDI / D
    The start point is clocked by            IMPLANT_TOP|Clock_4MHz_inferred_clock [rising] on pin CLK
    The end   point is clocked by            IMPLANT_TOP|Clock_4MHz_inferred_clock [rising] on pin CLK

Instance / Net                                                                           Pin      Pin               Arrival     No. of    
Name                                                                          Type       Name     Dir     Delay     Time        Fan Out(s)
------------------------------------------------------------------------------------------------------------------------------------------
MYImplant.Inst_CONTROL_NEXUS.Inst_Zarlink_SPI_Module.FSM_STATE[0]             DFN1C0     Q        Out     1.771     1.771       -         
FSM_STATE[0]                                                                  Net        -        -       5.855     -           23        
MYImplant.Inst_CONTROL_NEXUS.Inst_Zarlink_SPI_Module.FSM_STATE_RNIR52J[0]     OR2B       B        In      -         7.626       -         
MYImplant.Inst_CONTROL_NEXUS.Inst_Zarlink_SPI_Module.FSM_STATE_RNIR52J[0]     OR2B       Y        Out     1.508     9.134       -         
N_58                                                                          Net        -        -       2.844     -           4         
MYImplant.Inst_CONTROL_NEXUS.Inst_Zarlink_SPI_Module.FSM_STATE_RNI77JS[1]     OR2A       B        In      -         11.978      -         
MYImplant.Inst_CONTROL_NEXUS.Inst_Zarlink_SPI_Module.FSM_STATE_RNI77JS[1]     OR2A       Y        Out     1.236     13.214      -         
N_525                                                                         Net        -        -       2.844     -           4         
MYImplant.Inst_CONTROL_NEXUS.Inst_Zarlink_SPI_Module.SPI_SDI_RNO_23           NOR3A      C        In      -         16.058      -         
MYImplant.Inst_CONTROL_NEXUS.Inst_Zarlink_SPI_Module.SPI_SDI_RNO_23           NOR3A      Y        Out     1.541     17.599      -         
N_596                                                                         Net        -        -       0.773     -           1         
MYImplant.Inst_CONTROL_NEXUS.Inst_Zarlink_SPI_Module.SPI_SDI_RNO_9            AO1        C        In      -         18.372      -         
MYImplant.Inst_CONTROL_NEXUS.Inst_Zarlink_SPI_Module.SPI_SDI_RNO_9            AO1        Y        Out     1.520     19.892      -         
SPI_SDI_18_iv_0_0_4                                                           Net        -        -       0.773     -           1         
MYImplant.Inst_CONTROL_NEXUS.Inst_Zarlink_SPI_Module.SPI_SDI_RNO_2            OR3A       B        In      -         20.664      -         
MYImplant.Inst_CONTROL_NEXUS.Inst_Zarlink_SPI_Module.SPI_SDI_RNO_2            OR3A       Y        Out     1.716     22.381      -         
SPI_SDI_18_iv_0_0_9                                                           Net        -        -       0.773     -           1         
MYImplant.Inst_CONTROL_NEXUS.Inst_Zarlink_SPI_Module.SPI_SDI_RNO              OR3        C        In      -         23.153      -         
MYImplant.Inst_CONTROL_NEXUS.Inst_Zarlink_SPI_Module.SPI_SDI_RNO              OR3        Y        Out     1.804     24.957      -         
SPI_SDI_18                                                                    Net        -        -       0.773     -           1         
MYImplant.Inst_CONTROL_NEXUS.Inst_Zarlink_SPI_Module.SPI_SDI                  DFN1C0     D        In      -         25.730      -         
==========================================================================================================================================
Total path delay (propagation time + setup) of 27.108 is 12.474(46.0%) logic and 14.634(54.0%) route.
Path delay compensated for clock skew. Clock skew is added to clock-to-out value, and is subtracted from setup time value


Path information for path number 5: 
      Requested Period:                      10.000
    - Setup time:                            1.378
    + Clock delay at ending point:           0.000 (ideal)
    = Required time:                         8.622

    - Propagation time:                      25.529
    - Clock delay at starting point:         0.000 (ideal)
    = Slack (non-critical) :                 -16.908

    Number of logic level(s):                6
    Starting point:                          MYImplant.Inst_CONTROL_NEXUS.Inst_Zarlink_SPI_Module.FSM_STATE[5] / Q
    Ending point:                            MYImplant.Inst_CONTROL_NEXUS.Inst_Zarlink_SPI_Module.SPI_SDI / D
    The start point is clocked by            IMPLANT_TOP|Clock_4MHz_inferred_clock [rising] on pin CLK
    The end   point is clocked by            IMPLANT_TOP|Clock_4MHz_inferred_clock [rising] on pin CLK

Instance / Net                                                                           Pin      Pin               Arrival     No. of    
Name                                                                          Type       Name     Dir     Delay     Time        Fan Out(s)
------------------------------------------------------------------------------------------------------------------------------------------
MYImplant.Inst_CONTROL_NEXUS.Inst_Zarlink_SPI_Module.FSM_STATE[5]             DFN1C0     Q        Out     1.771     1.771       -         
FSM_STATE[5]                                                                  Net        -        -       5.926     -           24        
MYImplant.Inst_CONTROL_NEXUS.Inst_Zarlink_SPI_Module.FSM_STATE_RNIR52J[0]     OR2B       A        In      -         7.697       -         
MYImplant.Inst_CONTROL_NEXUS.Inst_Zarlink_SPI_Module.FSM_STATE_RNIR52J[0]     OR2B       Y        Out     1.236     8.933       -         
N_58                                                                          Net        -        -       2.844     -           4         
MYImplant.Inst_CONTROL_NEXUS.Inst_Zarlink_SPI_Module.FSM_STATE_RNI77JS[1]     OR2A       B        In      -         11.777      -         
MYImplant.Inst_CONTROL_NEXUS.Inst_Zarlink_SPI_Module.FSM_STATE_RNI77JS[1]     OR2A       Y        Out     1.236     13.013      -         
N_525                                                                         Net        -        -       2.844     -           4         
MYImplant.Inst_CONTROL_NEXUS.Inst_Zarlink_SPI_Module.SPI_SDI_RNO_23           NOR3A      C        In      -         15.858      -         
MYImplant.Inst_CONTROL_NEXUS.Inst_Zarlink_SPI_Module.SPI_SDI_RNO_23           NOR3A      Y        Out     1.541     17.398      -         
N_596                                                                         Net        -        -       0.773     -           1         
MYImplant.Inst_CONTROL_NEXUS.Inst_Zarlink_SPI_Module.SPI_SDI_RNO_9            AO1        C        In      -         18.171      -         
MYImplant.Inst_CONTROL_NEXUS.Inst_Zarlink_SPI_Module.SPI_SDI_RNO_9            AO1        Y        Out     1.520     19.691      -         
SPI_SDI_18_iv_0_0_4                                                           Net        -        -       0.773     -           1         
MYImplant.Inst_CONTROL_NEXUS.Inst_Zarlink_SPI_Module.SPI_SDI_RNO_2            OR3A       B        In      -         20.464      -         
MYImplant.Inst_CONTROL_NEXUS.Inst_Zarlink_SPI_Module.SPI_SDI_RNO_2            OR3A       Y        Out     1.716     22.180      -         
SPI_SDI_18_iv_0_0_9                                                           Net        -        -       0.773     -           1         
MYImplant.Inst_CONTROL_NEXUS.Inst_Zarlink_SPI_Module.SPI_SDI_RNO              OR3        C        In      -         22.953      -         
MYImplant.Inst_CONTROL_NEXUS.Inst_Zarlink_SPI_Module.SPI_SDI_RNO              OR3        Y        Out     1.804     24.757      -         
SPI_SDI_18                                                                    Net        -        -       0.773     -           1         
MYImplant.Inst_CONTROL_NEXUS.Inst_Zarlink_SPI_Module.SPI_SDI                  DFN1C0     D        In      -         25.529      -         
==========================================================================================================================================
Total path delay (propagation time + setup) of 26.908 is 12.203(45.4%) logic and 14.705(54.6%) route.
Path delay compensated for clock skew. Clock skew is added to clock-to-out value, and is subtracted from setup time value


##### END OF TIMING REPORT #####]

--------------------------------------------------------------------------------
Target Part: AGLN250V2_VQFP100_STD
Report for cell IGLOO_TOP.igloo_top_arch
  Core Cell usage:
              cell count     area count*area
              AND2    16      1.0       16.0
              AND3     2      1.0        2.0
               AO1   122      1.0      122.0
              AO18     2      1.0        2.0
              AO1A    77      1.0       77.0
              AO1B     5      1.0        5.0
              AO1C    16      1.0       16.0
              AO1D    24      1.0       24.0
              AOI1    38      1.0       38.0
             AOI1B    15      1.0       15.0
               AX1     3      1.0        3.0
              AX1A     5      1.0        5.0
              AX1B     3      1.0        3.0
              AX1C    16      1.0       16.0
              AX1E     4      1.0        4.0
              AXO5     2      1.0        2.0
              AXO7     1      1.0        1.0
             AXOI5     1      1.0        1.0
            CLKINT     5      0.0        0.0
               GND    21      0.0        0.0
               INV    14      1.0       14.0
               MX2   537      1.0      537.0
              MX2A    20      1.0       20.0
              MX2B    33      1.0       33.0
              MX2C    13      1.0       13.0
             NAND2     2      1.0        2.0
            NAND2A     1      1.0        1.0
              NOR2   190      1.0      190.0
             NOR2A   441      1.0      441.0
             NOR2B   239      1.0      239.0
              NOR3    90      1.0       90.0
             NOR3A   139      1.0      139.0
             NOR3B   167      1.0      167.0
             NOR3C   126      1.0      126.0
               OA1    32      1.0       32.0
              OA1A    39      1.0       39.0
              OA1B    66      1.0       66.0
              OA1C    82      1.0       82.0
              OAI1     2      1.0        2.0
               OR2   209      1.0      209.0
              OR2A   131      1.0      131.0
              OR2B    52      1.0       52.0
               OR3   191      1.0      191.0
              OR3A    29      1.0       29.0
              OR3B    35      1.0       35.0
              OR3C    13      1.0       13.0
               VCC    21      0.0        0.0
               XA1    15      1.0       15.0
              XA1A    27      1.0       27.0
              XA1B     6      1.0        6.0
              XA1C    37      1.0       37.0
              XAI1     6      1.0        6.0
             XAI1A     2      1.0        2.0
             XNOR2    13      1.0       13.0
               XO1    13      1.0       13.0
              XO1A     1      1.0        1.0
              XOR2    56      1.0       56.0
              XOR3     1      1.0        1.0


            DFI1C0     1      1.0        1.0
            DFN0C0    71      1.0       71.0
            DFN0E1   128      1.0      128.0
          DFN0E1C0    30      1.0       30.0
            DFN0P0    10      1.0       10.0
            DFN1C0   318      1.0      318.0
          DFN1E0C0   547      1.0      547.0
            DFN1E1    20      1.0       20.0
          DFN1E1C0   681      1.0      681.0
          DFN1E1P0    12      1.0       12.0
            DFN1P0    34      1.0       34.0
          FIFO4K18     8      0.0        0.0
                   -----          ----------
             TOTAL  5329              5274.0


  IO Cell usage:
              cell count
            CLKBUF     1
             INBUF    10
            OUTBUF    27
                   -----
             TOTAL    38


Core Cells         : 5274 of 6144 (86%)
IO Cells           : 38

  RAM/ROM Usage Summary
Block Rams : 8 of 8 (100%)

Mapper successful!

At Mapper Exit (Real Time elapsed 0h:00m:24s; CPU Time elapsed 0h:00m:23s; Memory used current: 61MB peak: 195MB)

Process took 0h:00m:24s realtime, 0h:00m:23s cputime
# Tue Sep 02 15:34:52 2014

###########################################################]
``` ````
