# Supplementary material for: Implications for a Wireless, External Device System to Study Electrocorticography
Source: Sensors (Basel). 2017 Apr 4;17(4):761. doi: 10.3390/s17040761 (PMC5421721; doi:10.3390/s17040761)
Supplement: Supplementary file 1 [file sensors-17-00761-s001.zip › Supplement/RHD/IGLOO_RHD_02092014.zip!/IGLOO_RHD/synthesis/syntmp/statusReport.html]

 
Project Status Summary Page


|  |  |  |  |  |  |  |  |
| --- | --- | --- | --- | --- | --- | --- | --- |
| Project Settings | | | |||  |  |  |  |
| --- | --- | --- | --- |
| Project Name | IGLOO\_TOP\_syn | Implementation Name | synthesis |


| Top Module | work.IGLOO\_TOP | Retiming | 0 |
| Resource Sharing | 1 | Fanout Guide | 24 |
| Disable I/O Insertion | 0 | FSM Compiler | 1 |

  

| Run Status | | | | | | | | |
| --- | --- | --- | --- | --- | --- | --- | --- | --- |
| Job Name | Status |  |  |  | CPU Time | Real Time | Memory | Date/Time |
| Compile Input | Complete | 145 | 32 | 0 | - | 0m:03s | - | 9/2/2014 |
| Pre-mapping | Complete | 67 | 3 | 0 | 0m:00s | 0m:00s | 113MB | 9/2/2014 3:34:27 PM |
| Map & Optimize | Complete | 15 | 8 | 0 | 0m:23s | 0m:24s | 195MB | 9/2/2014 3:34:52 PM |

  

| Area Summary | | | |
| --- | --- | --- | --- |
|  |  |  |  |
| --- | --- | --- | --- |
|  | | | |
| Core Cells | 5274 | IO Cells | 38 |
| Block RAMs (v\_ram) | 8 |  |  |

  

| Timing Summary | | | |
| --- | --- | --- | --- |
|  |  |  |  |
| --- | --- | --- | --- |
|  | |  |  |
| Clock Name | Req Freq | Est Freq | Slack |
| --- | --- | --- | --- |
| IGLOO\_TOP\|Clk | 24.0 MHz | 81.3 MHz | 29.371 |
| IMPLANT\_TOP\|Clk\_inferred\_clock | 100.0 MHz | 15.2 MHz | -27.896 |
| IMPLANT\_TOP\|Clock\_4MHz\_inferred\_clock | 100.0 MHz | 36.0 MHz | -17.743 |

  

| Optimizations Summary | | | |
| --- | --- | --- | --- |
| Combined Clock Conversion | 1 / 2 |  |  |
